# Selection signatures in goats reveal a novel deletion mutant underlying cashmere yield and diameter

**DOI:** 10.1093/gigascience/giac107

**Published:** 2022-11-03

**Authors:** Hu Han, Man-Man Yang, Jiang Dan, Sheng-Yu Chao, Xing-Ju Zhang, Qiang Wei, Tao Chen, Qi-Ju Wang, Cheng-Ye Yang, Bater Wulan, Ting-Ting Zhang, Gang Gen, Mengkedala, Bin Li, Wei-Dong Deng, Ze-Pu Miao, Ran Wang, Qing-Feng Zhang, Lin Li, Ming Fang, Yong Li

**Affiliations:** Fisheries College, Jimei University, Xiamen, 361021, PR China; Animal breeding department, BGI Institute of Applied Agriculture, Shenzhen, 518120, PR China; Animal breeding department, BGI Institute of Applied Agriculture, Shenzhen, 518120, PR China; Shenzhen Engineering Laboratory for Genomics–Assisted Animal Breeding, BGI-Shenzhen, Shenzhen, 518083, PR China; Fisheries College, Jimei University, Xiamen, 361021, PR China; Animal Epidemic Disease Prevention and Control Center, Haixi Autonomous Prefecture of Qinghai Province, Delingha, Qinghai, 817099, PR China; Haixi Agricultural Technology Extension Service Center, Haixi Autonomous Prefecture of Qinghai Province, Delingha, Qinghai, 817099, PR China; Animal breeding department, BGI Institute of Applied Agriculture, Shenzhen, 518120, PR China; Shenzhen Engineering Laboratory for Genomics–Assisted Animal Breeding, BGI-Shenzhen, Shenzhen, 518083, PR China; Animal breeding department, BGI Institute of Applied Agriculture, Shenzhen, 518120, PR China; Shenzhen Engineering Laboratory for Genomics–Assisted Animal Breeding, BGI-Shenzhen, Shenzhen, 518083, PR China; Animal breeding department, BGI Institute of Applied Agriculture, Shenzhen, 518120, PR China; Shenzhen Engineering Laboratory for Genomics–Assisted Animal Breeding, BGI-Shenzhen, Shenzhen, 518083, PR China; Animal Epidemic Disease Prevention and Control Center, Haixi Autonomous Prefecture of Qinghai Province, Delingha, Qinghai, 817099, PR China; BGI Co. Ltd., Shenzhen, 518083, PR China; Haixi Agricultural Technology Extension Service Center, Haixi Autonomous Prefecture of Qinghai Province, Delingha, Qinghai, 817099, PR China; Animal breeding department, BGI Institute of Applied Agriculture, Shenzhen, 518120, PR China; Shenzhen Engineering Laboratory for Genomics–Assisted Animal Breeding, BGI-Shenzhen, Shenzhen, 518083, PR China; Haixi Agricultural Technology Extension Service Center, Haixi Autonomous Prefecture of Qinghai Province, Delingha, Qinghai, 817099, PR China; Haixi Agricultural Products Quality Safety Inspection and Testing Center, Haixi Autonomous Prefecture of Qinghai Province, Delingha, Qinghai, 817099, PR China; Animal breeding department, BGI Institute of Applied Agriculture, Shenzhen, 518120, PR China; Faculty of Animal Science and Technology, Yunnan Agricultural University, Kunming, 650201, PR China; Animal breeding department, BGI Institute of Applied Agriculture, Shenzhen, 518120, PR China; Animal breeding department, BGI Institute of Applied Agriculture, Shenzhen, 518120, PR China; The Enterprises Key Laboratory of Tianjin Mutton Sheep Genetics and Breeding, Tianjin Aoqun Animal Husbandry Pty. Ltd., Tianjin, 301607,PR China; Animal breeding department, BGI Institute of Applied Agriculture, Shenzhen, 518120, PR China; Fisheries College, Jimei University, Xiamen, 361021, PR China; Fisheries College, Jimei University, Xiamen, 361021, PR China; Animal breeding department, BGI Institute of Applied Agriculture, Shenzhen, 518120, PR China; Shenzhen Engineering Laboratory for Genomics–Assisted Animal Breeding, BGI-Shenzhen, Shenzhen, 518083, PR China; BGI Co. Ltd., Shenzhen, 518083, PR China

**Keywords:** *LHX2*, selective sweep, insulator, whole genome sequencing, cashmere trait

## Abstract

Cashmere traits were deployed for fiber yield and quality during the domestication of goats. However, the genetic alterations underlying cashmere trait selection are still unclear. We sequenced 120 Chinese native goats, including 2 cashmere goat breeds and 6 ordinary goat breeds. The genome-wide selective sweep of cashmere goat and ordinary goat revealed a novel set of candidate genes as well as pathways, such as nuclear factor–κB and Wnt signaling pathways. Of them, *LHX2*, regulating hair follicle development, was evident from the strongest selection signal when comparing the Uhumqin cashmere goat and ordinary goat. Interestingly, we identified a 582-bp deletion at 367 kb upstream of *LHX2* with higher frequency in cashmere goats and their ancient relatives. This mutation probably arose along with breeding procedures and is putatively responsible for cashmere production and diameter, as revealed by association studies. Luciferase assay showed that the 582-bp sequence, which acts as an insulator, restrains the expression of *LHX2* by interfering with its upstream enhancers. Our findings provide new insights into the genetic formation of cashmere and facilitate subsequent molecular breeding for cashmere goat improvement.

## Introduction

Cashmere contributes high economic value to the textile industry. Cashmere is made from the processing of the underwool of goats that is grown in high and cold regions of the world, such as the Tibetan plateau and Mongolia. China is the largest cashmere producer globally, accounting for approximately 75% of the world's supply [1]. Improvement in the quantity and quality of cashmere is an important breeding goal in goat farming.

The cashmere goats with a long fine underwool share a common ancestor with other ordinary goats that were domesticated from wild goat (bezoar) approximately 11,000 years ago in the Fertile Crescent of southwest Asia and adjacent areas [2, 3]. Almost all the breeds of goats can produce more or less cashmere fibers, but only the breeds with sufficiently fine hair are called cashmere goats (cashmere goats in China produce 250 to 500 g of fiber, while ordinary goats produce only 50 g) [4]. Previous research postulated that cashmere goats would not be domesticated locally, except several highland regions including the Himalayas, Mongolia, and Kirghizia [5]. This kind of domestication leads to selecting more and more cashmere wool to meet textile demands. Therefore, in contrast to cashmere fiber formation, cashmere production was pursued by intense artificial selection pressure during the domestication of cashmere goats [6–8].

Previous transcriptome studies in cashmere goats have already discovered many important pathways that are involved in cashmere fiber development, including *WNT,FGF5,BMP,TGF-β,NOTCH*, and*SHH*, and some of them may play important roles in secondary hair follicle development, such as *LHX2,FGF5*, and *TGF-β4* [9–15]. Meanwhile, some genes, such as *FGF5, SGK3, IGFBP7, OXTR, ROCK1, LHX2,FGF9,PRDM6*, and *WNT2*, were intensively selected in cashmere goats [6, 16, 17]. Recent studies have identified several causal genes for cashmere growth trait, including *FGF5* and *EDA2R* [7, 18]. *FGF5* is a regulator of the hair growth cycle, and the disruption of *FGF5* is associated with a long hair characteristic [19, 20]. Interestingly, the enhancer-absent *FGF5* was found only in domesticated goats rather than in the wild goats, indicating this causative mutation occurred in the domestication process [7, 21]. However, more research is necessary to elucidate the genetic basis of cashmere fiber formation in goats after divergence from sheep.

Here, we generated genomic data from 42 cashmere goats and 78 ordinary goats in China and conducted comprehensive population genomic analyses. Integrated with goat genomes, we identified genetic footprints under artificial selection during goat migration and domestication. We compared the ancient and wild goat, as well as investigated the origin and biological function of causative mutation for cashmere fiber formation.

## Results

### Population sequencing and genetic diversity

**Figure 1:**
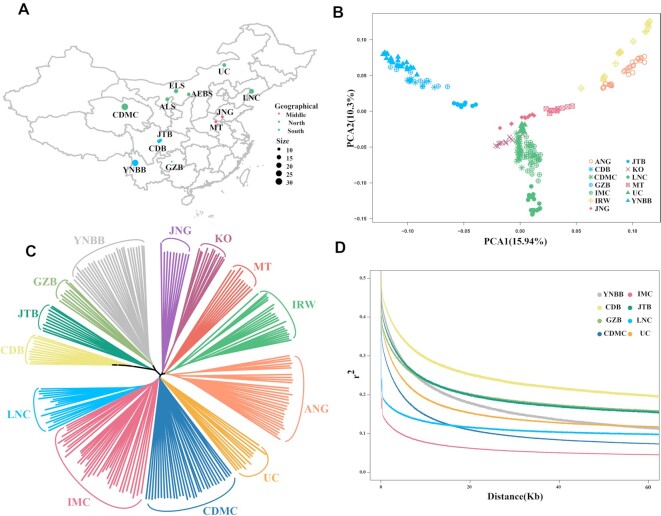
Geographic distribution and population genetic analyses of 13 goat breeds. (A) Geographical distribution of domestic goat breeds in China. Green, blue, and red represent northern, southwest, and middle regions, respectively. (B) Principal component (PC) plot of the first 2 components. The fraction of the variance explained is 15.94% and 10.3% for PC1 and PC2, respectively. (C) Neighbor-joining tree constructed using p-distances between different breeds, including LNC (Liaoning cashmere), IMC (Inner Mongolia cashmere), UC (Ujumqin cashmere), CDMC (Chaidamu cashmere), CDB (Chengdu brown), GZB (Guizhou black), YNBB (Yunnan black bone), JTB (Jintang black), MT (Matou), JNG (Jining gray), KO (Korean), IRW (Iranian wild), and ANG (Angora). (D) Linkage disequilibrium decay of goat populations measured by *r*^2^.

A total of 120 domestic goats representing 8 geographically diverse breeds in China were selected for genome resequencing (Supplementary Table S1). These datasets were also analyzed together with the published whole-genome sequencing (WGS) datasets of 116 individuals from 5 breeds (Supplementary Tables S2 and S3). Among the 10 domestic goat breeds, Liaoning cashmere goat (LNC), Inner Mongolia cashmere goat (IMC), Ujumqin cashmere goat (UC), and Chaidamu cashmere goat (CDMC) are the cashmere goat breeds located in the north of China; Chengdu brown goat (CDB), Guizhou black goat (GZB), Yunnan black bone goat (YNBB), and Jintang black goat (JTB) in the southwest and Matou goat (MT) and Jining gray goat (JNG) in the middle region are noncashmere goat breeds (Fig. 1a). The remaining goat breeds include Korea goat (KO), Iranian wild goat (IRW), and Angora goat (ANG) from Korea, Iran, and France, respectively.

A total of 8,448.92 Gb paired-end DNA sequence data were obtained from 236 goats (Supplementary Table S4). All goats were sequenced with an average 12-fold depth of the genome (4.17∼29.9×) and the average genome coverage of 97.82% (Supplementary Tables S1–S3). Of the reads, 99.2% were mapped to the latest goat reference genome ARS1 (GCA_001704415.1) (Supplementary Tables S1–S3). We detected 13,069,924 high-quality single-nucleotide polymorphisms (SNPs), with 0.605% (0.229 million) located in exonic regions (Supplementary Table S1). The average transition-to-transversion (Ti/Tv) ratio was 2.46 for all goat samples, which indicated relatively low potential random sequencing errors (Supplementary Table S1).

The principal component analysis (PCA) of the 236 goats revealed genetically distinct clusters according to their geographic locations. The clustering results of the northern cashmere goat populations (IMC, LNC, UC, and CDMC) and ordinary goat populations (YNBB, JTB, GZB, and CDB) were clearly separated (Fig. 1B). JNG and MT were divided into subgroups between cashmere goats and the ordinary southwest goats. Samples of IRW and ANG (France, South Africa, and Madagascar) were significantly different from those of Chinese native goats (Fig. 1B). This result was confirmed by the phylogenetic tree using the same SNPs (Fig. 1C). In the analysis of linkage disequilibrium (LD), 4 ordinary goat breeds from southwest China (YNBB, JTB, GZB, and CDB) showed an overall slower decay rate and a higher level of LD than the cashmere breeds from northern China (IMC, LNC, UC, and CDMC) (Fig. 1D).

**Figure 2:**
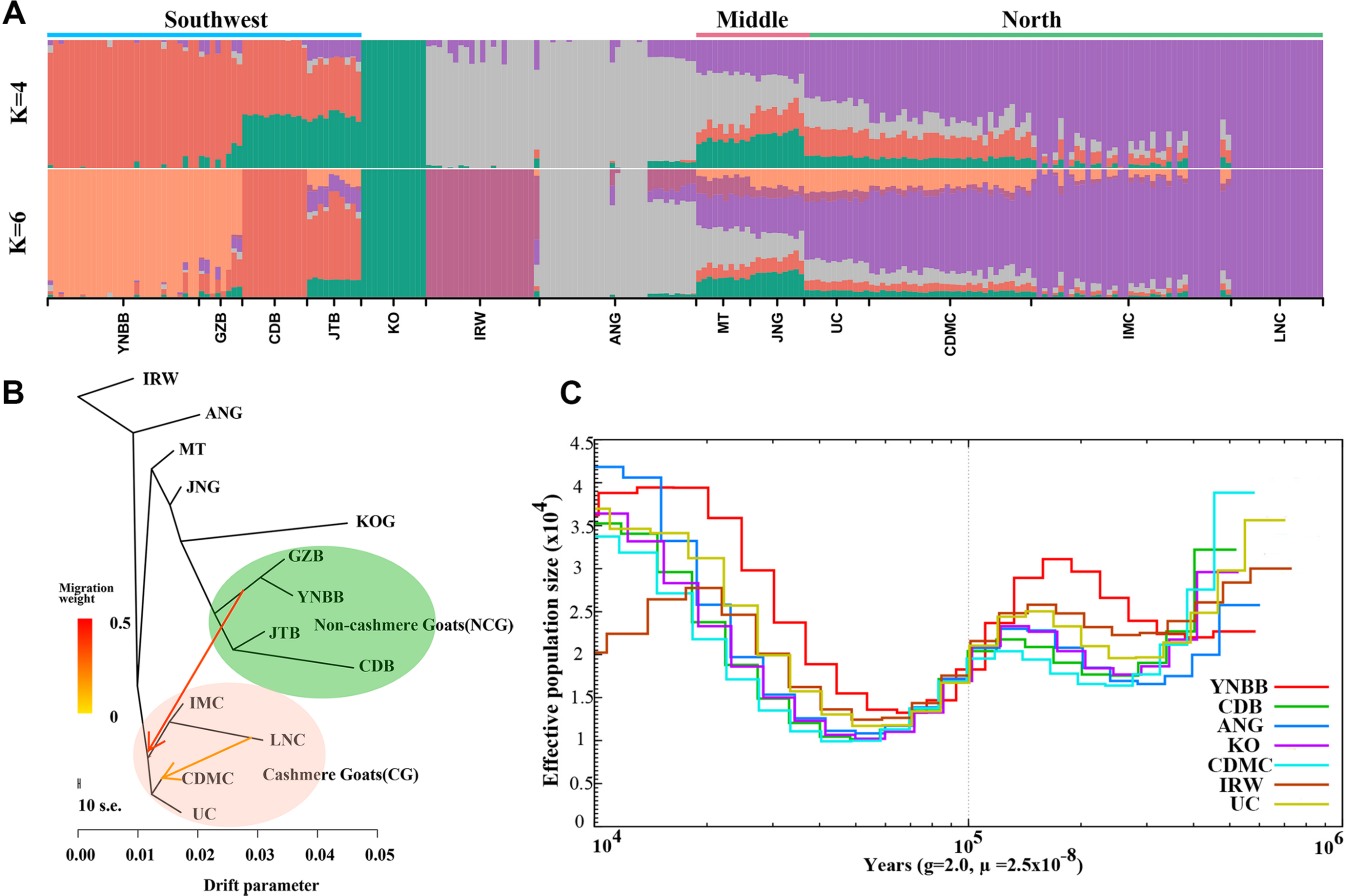
Population structure, gene flows, and effective population size of goat populations. (A) Model-based population assignment with ADMIXTURE analysis for *K* = 4 and 6, respectively. The population names are at the bottom of the figure, and the geographic locations are at the top. (B) Phylogenetic network of the inferred relationships among the 13 native breeds with 2 intergroup migration edges being identified. The branch length is proportional to the drift of each population. IRW (Iranian wild goat) was used as the out-group to root the tree. The colored regions in the phylogenetic tree represent 2 inferred genetic groups. Arrows indicate migration events, and a spectrum of heat colors indicates the migration weights of the migration events. (C) Pairwise sequentially Markovian coalescent analysis for the representative individuals sequenced at a high read coverage, exhibiting inferred variations in Ne over the past 10^6^ years. g (generation time) = 2 years; μ (neutral mutation rate per generation) = 2.5 × 10^−8^.

In the STRUCTURE analysis, when *K* = 4, we observed 5 separate clusters: IRW and ANG in west Asia; YNBB, GZB, JTB, and CDB in southwest China; cashmere goat in north China; MT and JNG in middle east China; and Korean goats in south Korea. At *K* = 6, goats in southwest China further split into 2 geographic subgroups: the Yunnan-Kweichow Plateau group, including YNBB and GZB goats, and the Chengdu Plain group, including CDM and JTB goats. Two west Asian goats (IRW and ANG) were also separated (Fig. 2a, Supplementary Fig. S2). Some cashmere goats showed evidence of admixture, which may be attributable to shared ancestral polymorphism and recent introgression events by crossbreeding with neighboring domestic goats. Interestingly, the genetic structure of the Chinese goat population revealed that UC and LNC goats represent 2 different types, and other cashmere goats are a mixture of these two types (Supplementary Fig. S3).

In TreeMix analysis, we found that cashmere goats and ordinary goats clustered into 2 groups (Fig. 2B). We observed 2 migration edges among clusters from LNC to CDMC and from YNBB and GZB to the cashmere goat (Fig. 2B), which can be explained by the fact that CDMC goats were crossbred between goats of the Qinghai-Tibet Plateau and LNC.

Seven high-coverage samples were chosen to infer the effective population size (Ne) over the past 10^6^ years using the pairwise sequential Markovian coalescent (PSMC) method [22]. PSMC analysis suggested that the goats suffered at least 2 bottlenecks (approximately 7,000 and 18,000 years ago), which resulted in a severe reduction in the effective population size (Fig. 2C). Interestingly, the results of the PSMC analysis of goats were like the demographic history of the sheep [23].

### Genome-wide selective sweeps

**Figure 3:**
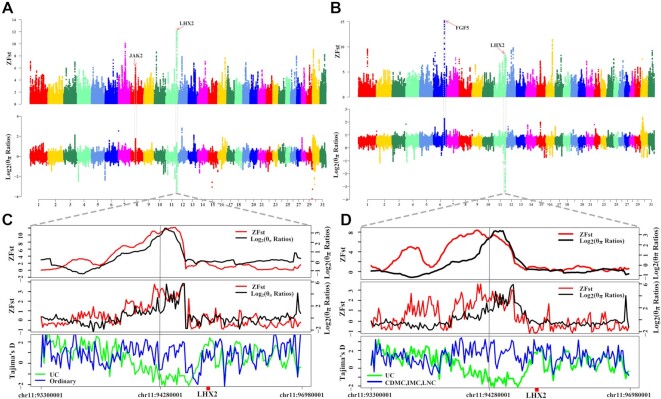
Genomic regions with selection sweep signals in domestic goats. (A) Manhattan plot of the genome-wide distribution of pairwise ZFst and log_2_(θ_π_ ratios) between UC and ordinary goats (YNBB, GZB, JTB, and CDB) using a 150-kb window size and a 10-kb step size. (B) Manhattan plot of the genome-wide distribution of pairwise ZFst and log_2_(θ_π_ ratios) between UC and other cashmere goats (CDMC, LNC, and IMC) using a 150-kb window size and a 10-kb step size. (C) Zoom of the peak signal on chromosome 11 between UC and ordinary goats (YNBB, GZB, JTB, and CDB). Log_2_(θ_π_ ratios), ZFst values, and Tajima's *D* values around the *LHX2* region (Fig. 3A) using a nonoverlapping 10-kb sliding window. The black and red lines represent log_2_(θ_π_ ratios) and ZFst values, respectively; the blue and green lines represent the ordinary goats and UC goats, respectively. (D) Zoom of the peak signal on chromosome 11 between UC and other cashmere goats (CDMC, LNC, and IMC). Log_2_(θ_π_ ratios), ZFst values, and Tajima's *D* values around the *LHX2* region (Fig. 3B) using a nonoverlapping 10-kb sliding window. The blue and green lines represent the other cashmere goats and UC goats, respectively.

**Figure 4:**
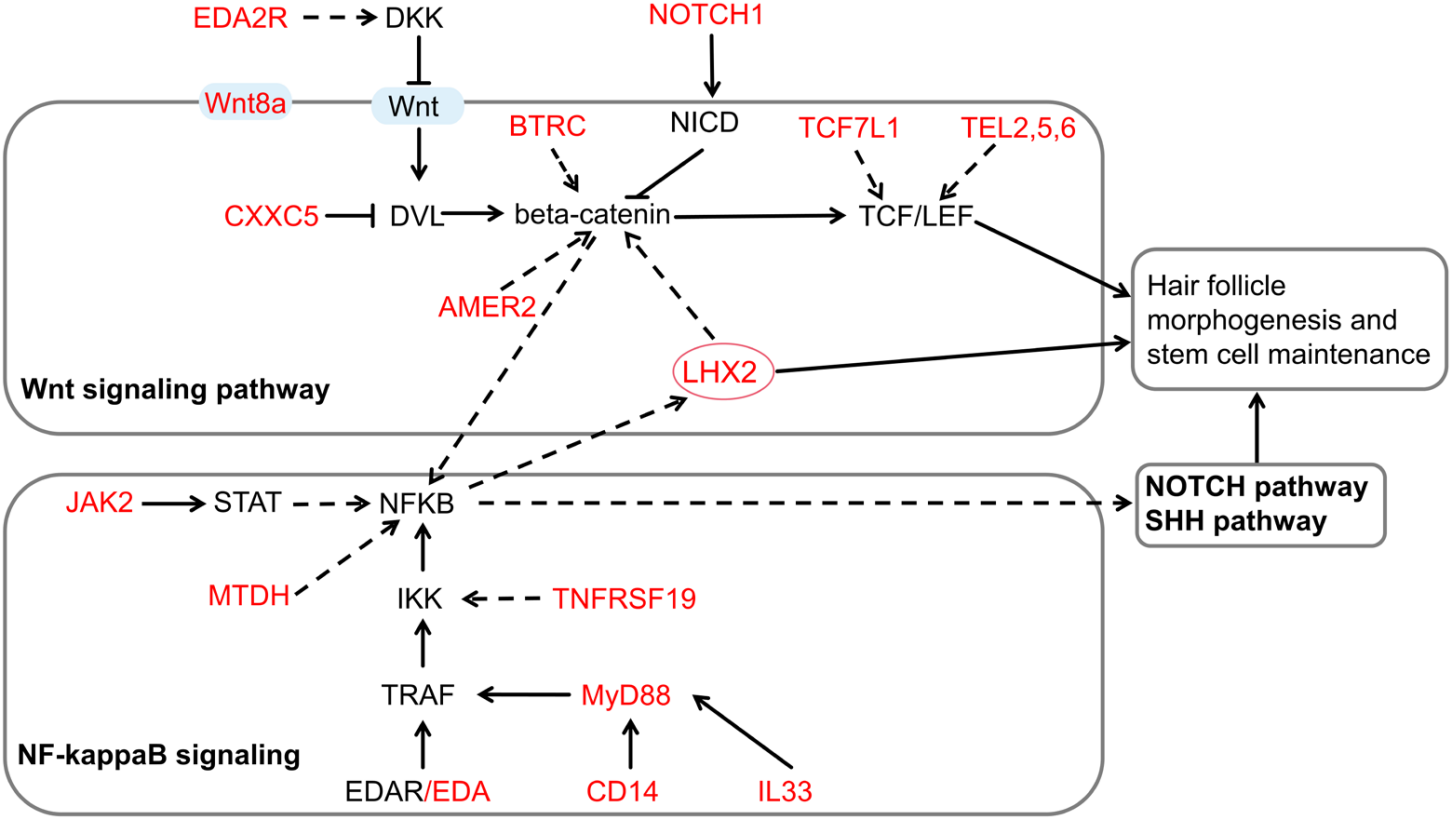
Schematic mechanisms of signaling pathways involved in cashmere fiber development. The names of the KEGG pathways are shown in bold. The candidate genes positively selected in the 2 methods of Fst and θπ ratio tests are shown in red. The solid block arrows represent direct effect, and the dashed black arrows indicate an indirect effect. The blunt-head arrow indicates an inhibition effect.

The results of the genetic structure analysis showed that the 2 cashmere goats (UC and CDMC) had apparent admixture with some other ordinary breeds. In contrast to CDMC bred through crossbreeding between Qinghai native goats and LNC, UC is originally from the Ujumuqin region and is continually selected for cashmere traits. Therefore, the distinct genetic background of UC raised the question of whether there is a different selective sweep region for the cashmere trait. We then performed a selective sweep analysis with the 12 UC and 58 ordinary goat genome sequences by estimating pairwise genetic differentiation (Fst) and nucleotide diversity differences (θπ) in 150-kb sliding windows along the genome. Using the top 1% of ZFst values and log_2_(θ_π_ ratios) cutoffs (ZFst > 4.01, the absolute value of log_2_(θ_π_ ratios) > 0.85), we identified 201 candidate genes associated with cashmere traits (Fig. 3A, Supplementary Table S2). We found that the most prominent ZFst signature was on chromosome 11 (Fig. 3A), spanning ∼1,100 kb region (93.6–94.7 Mb). This signature was also supported by the ZFst, log_2_(θ_π_ ratios), and Tajima's *D* statistics (Fig. 3C). However, we did not identify the selection signal harboring *FGF5*, which has been identified in previous reports [7]. We next used UC and other cashmere goats to perform selective sweep analysis and identified 263 genes corresponding to selective sweeps (Fig. 3B and D, Supplementary Table S3). Interestingly, both selection signals on chromosome 6 and *FGF5* were simultaneously identified in these populations. Among all the candidate genes, 9 (*EDA, MyD88, CD14, IL33, TNFRSF19, LHX2, AR, STK3*, and *JAK2*) were located in the nuclear factor (NF)–κB signaling pathway and 11 (*TCF7L1, WNT8B,BTRC, AMER2,TEL2, TEL5, TEL6, LHX2, CXXC5, NOTCH1*, and *VCAN*) were found in the Wnt/β-catenin signaling pathway (Fig. 4, Supplementary Fig. S6, Supplementary Tables S4 and S5). These signaling pathways play a central role in regulating hair follicle morphogenesis, stem cell differentiation, and hair cycle [24, 25]. The strongest selective sweep, localized on chromosome 11, harbored DENN domain-containing protein 1A (*DENND1A*), Crumbs cell polarity complex component 2 (*CRB2*), and LIM homeobox 2 (*LHX2*), in which *DENND1A* and *CRB2* are associated with polycystic ovary syndrome (PCOS) and maintenance of apicobasal polarity in the retinal pigment epithelium, respectively, but without evidence in hair development [26, 27]; another gene, *LHX2* near *DENND1A*, plays an important role in hair follicle development.

To confirm the reliability of our findings, we further used other cashmere goats (LNC, IMC, and CDMC) and ordinary goats to perform sweep selection analysis. The results showed that all previously detected functional genes for cashmere traits, including *FGF5, EDA2R*, and *STIM1*, were reidentified (Supplementary Fig. S7) [7, 21]. In addition to the cashmere trait, we also investigated the selection sweep for coat colors and confirmed previously reported genes *KIT, KITG, IRF4*, and *ASIP* for coat color using different goat breeds (Supplementary Fig. S8, Supplementary Fig. S9, and Supplementary Fig. S10). Furthermore, we also identified previously reported 100-kb copy number variants encompassing *KIT* (chr6: 70,859,258–70,959,918) and ∼154-kb copy number variants within *ASIP* (chr13: 63,226,824–63,381,501) with single base-pair resolution (Supplementary Fig. S11 and Supplementary Fig. S12).

### Plausible causative mutation near *LHX2*

**Figure 5:**
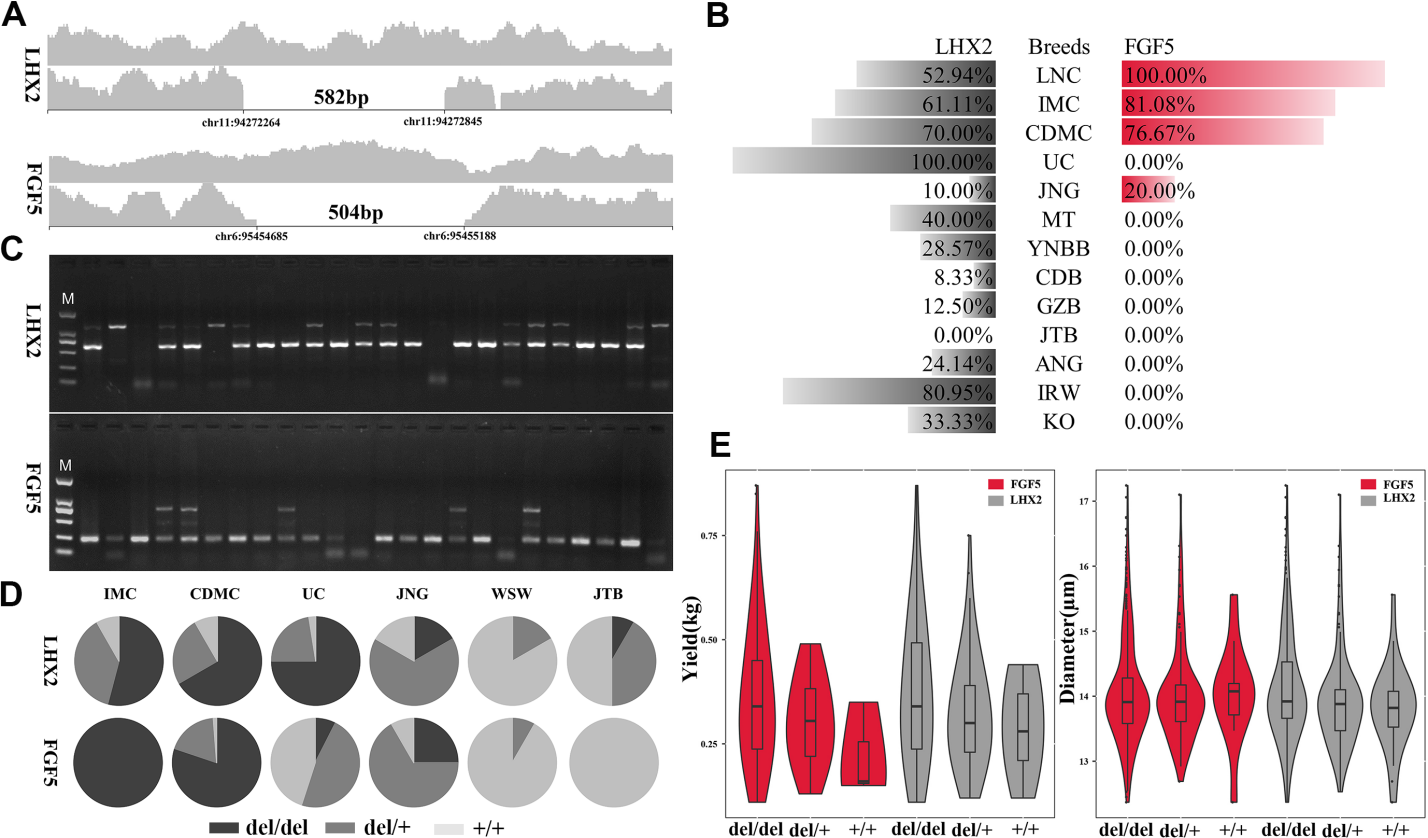
The *LHX2* and *FGF5* deletions in different goat breeds and their association with cashmere traits. (A) The identified 582-bp deletion near *LHX2* and the previously reported 504-bp deletion near *FGF5* based on read coverage. (B) Homozygous genotype frequency of the 2 deletions determined by the IGV viewer. (C) PCR amplification of the 2 deletion variants. (D) Distribution of the 582-bp deletion near *LHX2* and the 504-bp deletion of *FGF5* genotypes. del/del, deletion/deletion; del/+, deletion/wild type; +/+, wild type/wild type. WSW, Wushan white goat from southwest China. (E) The association of the 582-bp deletion near *LHX2* and 504-bp deletion of *FGF5* with cashmere yield and diameter in the CDMC goat population.

We inspected all variants within exons to identify the potential causal mutation around the *DENND1A-LHX2* locus; however, no coding variants were found (Supplementary Table S6). Strikingly, a 582-bp (chr11: 94,272,264–94,272,845 bp) deletion was identified in the 13th intron of *DENND1A* and 367 kb upstream of *LHX2* with the Integrative Genomics Viewer (IGV) (Fig. 5A). We tried to genotype the deletion variant of everyone in different populations, but it was not obvious to distinguish heterozygous type from homozygous wild type due to limited read counts with the IGV viewer, but it was possible to identify homozygous wild type. We then compared the frequencies of the homozygous deletion variant in different goat populations. The analysis results showed that the 582-bp deletion near *LHX2*(named 582del) had a higher frequency (52.9–100%) in cashmere goats as compared to 0.00% to 40.00% in ordinary goats (Fig. 5B and Supplementary Table S5). We also identified a 504-bp deletion (chr6: 95,454,685–95,455,188 bp) in the *FGF5* locus (named 504del), which is consistent with previous reports (Fig. 5a) [7]. However, the frequency of 582del in the ordinary goats was much higher than that of 504del (Fig. 5B and Supplementary Table S6). Intriguingly we found that 582del had a high frequency in the IRW population (80.9%), while 504del was absent, which is also consistent with previous research [7]. Since we could not accurately distinguish the heterozygous from wild genotypes using the IGV viewer, we designed PCR primers for detecting the heterozygosity of the 2 deletions in several goat populations. It showed that the frequency of homozygosity was consistent with that by the IGV viewer (Fig. 5C, Fig. 5D, Supplementary Table S7, and Supplementary Table S8), but some breeds had a higher frequency of heterozygosity, such as in UC (504del±: 47.5%) and JNG goats (504del±: 66.7%; 582del±: 66.7%). Finally, we detected 582del in several ancient goat samples (7/21) (Supplementary Table S9) and ibex samples (1/3) (Supplementary Fig. S13 and Supplementary Fig. S14), indicating that 582del is an older mutation that occurred much earlier than 504del. In addition, we compared the genotypes of the target genomic region for cashmere and ordinary goats. The result showed that the genotype patterns of cashmere goats were highly like that of wild goats but different from that of ordinary goats (Supplementary Fig. S15).

To further evaluate whether these 2 deletion variants were related to cashmere traits, we selected 235 CDMC goats with cashmere yield (Supplementary Fig. S16, Supplementary Table S10) and 581 CDMC goats with fiber diameter records (Supplementary Fig. S17, Supplementary Table S11) for association analysis. The association results showed that 582del and 504del were significantly associated with cashmere production (*P* = 0.0061 and *P* = 0.0113 for 582del and 504del, respectively). The 582del was also significantly associated with fiber diameter (*P* = 2.74e-04), but 504del was not (*P* = 0.348) (Fig. 5E). Interestingly, the interaction effects of 582del and 504del were significantly associated with fiber diameter (*P* = 6.1e-06), suggesting crosstalk between the 2 genes.

### Biological function of the 582-bp deletion

**Figure 6:**
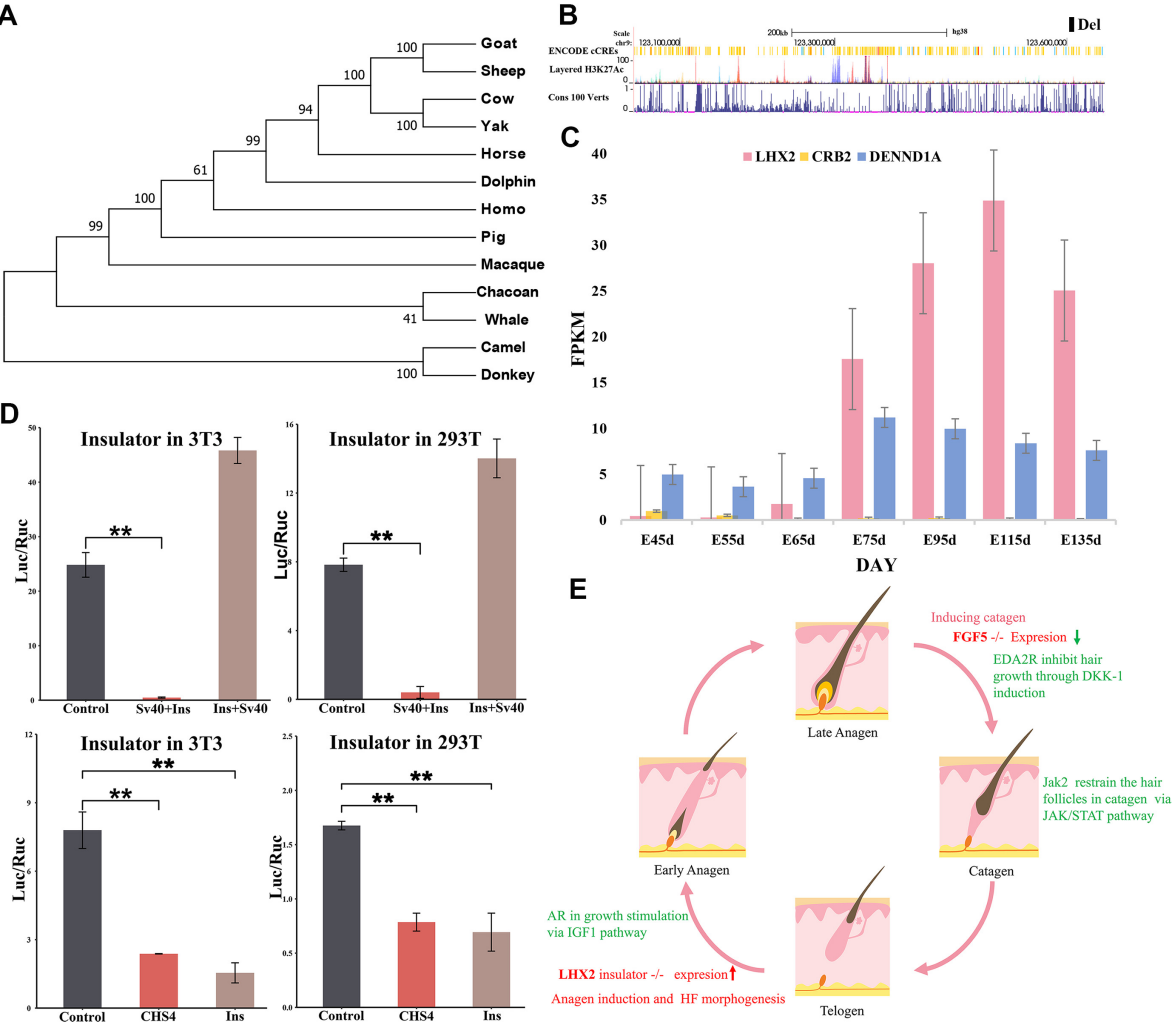
Effects of *LHX2* deletion sequence on reporter gene expression. (A) Phylogenetic tree of 13 primates and ungulates for the detection of the 582-bp deleted sequence. (B) Regulatory elements, epigenomic signals, and conservation scores on selective sweep region in chromosome 11. The black box represents the deletion variant location of *LHX2*. (C) The expression of *LHX2, CRB2*, and *DENND1A* in different stages of prenatal skin in cashmere goats. Boxes of different colors are used to represent genes. The expression data have been downloaded from a previous report [28]. (D) A dual-luciferase assay using NIH3T3 cell and 293T cell shows the *LHX2* upstream deletion sequence blocks the activity of luciferase. Data are shown as the mean ± standard error. The *P*value was calculated using the Student *t*test. (E) The molecular mechanisms of cashmere fiber development. In detail, the deletion of the *LHX2* insulator increases the expression of *LHX2* and promotes the cashmere fiber growth at the anagen stage, and *AR* could participate in hair growth at the anagen stage through the *IGF1* pathway [30], while the deletion of the 504-bp enhancer reduces the expression of *FGF5*, thereby inhibiting growth regression; *JAK2* and *EDA2R* may also participate in this stage via the JAK/STAT and Wnt pathways, respectively.

Analysis of the 582del region using the BLAST program revealed that it is not a highly conserved element but was found in the ancient genomes of primate and ungulate species (Fig. 6A). By checking the UCSC Genome Browser [70], we found many *cis*-regulatory elements and H3K27Ac marks (often enriched in enhancer regions) upstream of this deleted region (Fig. 6B). Interestingly, we identified many S/MAR sequences in the 582-bp sequence and a CTCF-binding site as well as 2 distal enhancer-like signatures that were close to it (Supplementary Sequences 1 and 2). Using previously reported RNA sequencing (RNA-seq) data [28], we found that the expression of *LHX2* in the different fetal stages of cashmere goats exhibited an upregulation pattern during hair follicle development (Fig. 6C, Supplementary Fig. S18). Furthermore, some functional enhancers of *LHX2* were also identified in the genome regulatory blocks of the *CRB2-LHX2* loci [29]. This evidence suggests that the 582-bp sequence may function as an insulator to block the *LHX2* enhancer function.

To confirm the insulator function of this sequence, we synthesized a 551-bp DNA fragment (named Ins, Supplementary Sequence 2) and subsequently inserted it downstream and upstream of the SV40 promoter in the pGL3 plasmid (Supplementary Fig. S17). The Ins vectors with the *Renilla*luciferase vector, phRL-TK, were transiently cotransfected into human 293T cells and mouse 3T3 cells. After 48 hours, the firefly and *Renilla* luciferase activities of the lysate were measured, and the ratio of firefly luciferase activity to *Renilla* luciferase activity was calculated for each sample. Our data showed that the Ins fragment decreased the expression of firefly luciferase by more than 90% in the downstream group of both cell types and increased expression in the upstream group (Fig. 6D). In addition, we inserted the Ins downstream of the SV40 enhancer in the pGL3 plasmid (Supplementary Fig. S19) and chose the well-known insulator cSH4 as a control to quantify the efficiency of the Ins. Our data showed that the Ins fragment decreased the expression of firefly luciferase by approximately 50% in human 293T cells, which was comparable to that of cSH4 (Fig. 6D). In addition, a similar result of the Ins activity was validated in the mouse 3T3 cells but decreased more sharply than the cSH4 group (Fig. 6D). These results suggested that the 551-bp DNA fragment played an enhancer-blocking function in mammary cells.

To describe the function mode of these selective sweep genes on the development cycle of cashmere, we downloaded and analyzed the transcriptome data of skin tissue during the prenatal stages and 1-year cashmere cycle. The transcriptome data showed that *AR* and *EDA2R* were not expressed in embryonic cashmere goat skin, and *JAK2* gene expression was opposite to that of *LHX2* (data from Wu et al. [28], Supplementary Fig. S20). Furthermore, *JAK2, EDA2R*, and *FGF5* exhibited similar expression patterns in the skin transcriptome data of different months of the 1-year cycle, while *LHX2* and *AR* had similar expression patterns (PRJNA470971) (Supplementary Figs. S20 and S21). It is known that cashmere fiber growth has 3 different periods in 1 year: a growth period (March–September), a regression period (September–December), and a resting period (December–March) [31]. Therefore, the deletion of the *LHX2* insulator increases the expression of *LHX2* and promotes cashmere fiber growth at the anagen stage, while deletion of the *FGF5* enhancer reduces the expression of *FGF5*, inhibiting the regression. Therefore, the 582-bp deletion disrupts the insulator and increases the expression of *LHX2*, as well as promotes cashmere fiber growth at the anagen stage, while deletion of the *FGF5* enhancer reduces the expression of *FGF5* and thus inhibits the regression of the hair follicle. Other candidate genes may affect cashmere fiber growth through uninvestigated regulation modes. Overall, we propose a possible molecular model for cashmere fiber formation: *LHX2* and *AR* may be involved in maintaining hair growth at the anagen stage, whereas *JAK2, EDA2R*, and *FGF5* function in the destructive phase (catagen) through the highest expression in September (Fig. 6E).

## Discussion

In this study, we resequenced the genomes of 42 cashmere goats and 78 ordinary goats. Population analyses revealed that goats in southwest China are different in the genome from goats in West Asia and North China, which is consistent with a recent study [7]. In the present study, we observed a genetic introgression from the LNC breed into the CDMC breed, which was confirmed by the origin and breed practice of CDMC (using Chaidamu goats as female parents and LNC as male parents to breed new cashmere goats, named CDMC) [32]. In addition, the introgression from the Yunnan-Kweichow Plateau into the cashmere goat breeds suggests that goats from the Qinghai-Tibet Plateau and Mongolian Plateau, as well as from the Yunnan-Kweichow Plateau, may have a common ancestor or a genetic admixture in their early years. Similar introgression results have also been reported in sheep populations from these regions [23]. In contrast, Angora goats have a signature of genetic admixture with some Chinese goats (Fig. 2A), which has also been reported previously [4]. In addition, although KO has large genome differences with other goat breeds [33], a clear signature of genetic admixture between KO and JT, JNG, and MT was observed, which may be related to the historical human migration between China and Korea. Finally, we observed CDMC and IMC goats presenting a genetic admixture of UC and LNC. LNC was bred in the 1980s from 6 counties in the eastern mountainous area of Liaoning Province in China, famous for its high cashmere yield. UC was originally bred from Ujumuqin white goat in the region of the Ujumuqin grassland for selecting the cashmere trait in 1994. Unlike LNC, UC has a closer genetic distance to goats in the middle region of China (Fig. 2A). These results indicate that UC may have a more unique genetic background than other cashmere goats.

By performing a whole-genome selection scan, we discovered a novel selective sweep region on chromosome 11 that appears to have undergone extremely strong selection in UC. This novel selective sweep region is located in a conserved linkage block, containing 3 genes: *DENND1A, CRB2*, and *LHX2*[29]. Among these genes, *LHX2* functions as a transcriptional activator in hair follicle stem cells and is an essential positive regulator of hair formation [34, 35]. Furthermore, the expression of *LHX2* was upregulated during hair follicle differentiation, and the expression abundance was constant throughout the development cycle in secondary hair follicles of cashmere goats [28, 31]. In contrast, *DENND1A* and *CRB2*, which function in endocytic trafficking to mediate the recycling of selective cargos and early embryonic development, respectively [36, 37], had low expression in the fetal stage of goat skin and showed very low expression during the cycle of adult cashmere fiber growth (Fig. 6C). These results indicate that *LHX2* is a functional gene in this selective sweep region.

We then reported an upstream deletion of *LHX2*, which carries a potential *cis*-regulatory insulator region (Fig. 6D). In contrast to the deletion allele of *FGF5* [7], the deletion allele of *LHX2* was found in all goats investigated, including wild and domesticated goats, but allele frequency was higher in northern and wild goats than in goats in other areas (Fig. 5B, D). Notably, this deletion was also found in the ancient goat and ibex samples (Supplementary Figs. S13 and S14). Ibex goats may also have dense underwool, like cashmere goats [4]. These results indicate that the deletion allele of *LHX2* occurred in the predomestication stage of goats and might be traced back to the early stage of goat speciation to cold climate adaptation. Further, we did not detect the 582-bp deletion in the same region of sheep, but we cannot rule out the possibility that we tested too few sheep (Supplementary Fig. S22). In contrast, the deletion allele of *FGF5* was not found in the Iranian wild goat (0/21), *Capra falconeri* and *Capra sibirica*(0/6), and ancient goats (0/21) (Supplementary Table S6), indicating that this deletion is most likely to occur during the domestication stage of goats to obtain higher cashmere production. Overall, it is possible that the deletion allele of *LHX2* is acquired in the wild goats at a very early stage to adapt to the cold environment and reaches a higher frequency in the north than in the south, while the *FGF5* enhancer deletion may have occurred during artificial selection or secondary domestication within the past 450 years [7] and fixed during the long-term artificial selection for cashmere trait. However, the origin and spread of cashmere-related mutations is just our inference based on available data, and there may be other possibilities worth further investigating.

Multiple sequence alignment revealed that the selective sweep region contains many CTCF binding sites and other *cis*-regulatory elements, as well as the H3K27ac epigenetic mark, located upstream of the 582-bp deletion. CTCF is a highly conserved zinc finger protein, required for insulator function in mammals [38], while H3K27ac is a marker for active enhancers and a great indicator of enhancer activity [39], suggesting that the selective sweep region contains some hair-related transcription regulatory elements. Our biological function experiments showed that the 582-bp deletion region contains an insulator for blocking the upstream enhancer role with higher efficiency in skin-related cells (NIH3T3 cells) than cSH4. An insulator is a long-range regulatory element that protects an expressing gene from its surroundings through 2 ways: blocking the action of a distal enhancer on a promoter and acting as “barriers” that prevent the advance of nearby condensed chromatin [38]. Previous reports described that those 4 of the 8 conserved noncoding elements (CNEs), approximately 222 to 619 kb upstream of human *LHX2*, functioned as tissue-specific enhancers in specific regions of the central nervous system and the dorsal root ganglia (DRG), recapitulating partial and overlapping expression patterns of *LHX2* and *CRB2* genes [29]. In our research, we found that the insulator is situated approximately 367 kb upstream of goat *LHX2* in the 13th intron of *DENND1A*. Therefore, we speculate that this insulator can regulate the expression of the *LHX2* gene by blocking the upstream enhancers, and the deletion of the insulator can increase the expression of the *LHX2* gene, thus increasing cashmere production. Subsequently, association analysis confirmed the significant relationship between the deletion genotype and cashmere traits, including cashmere production and fiber diameter (Fig. 5E). The interaction effect of the 2 deletion variants significantly affected the diameter of the cashmere fiber, indicating that the 2 genes have a synergistic effect on cashmere fiber development. *LHX2* is primarily expressed by precursor cells outside of the bulge region at the anagen stage and becomes undetectable when the HFs enter telogen [35]. FGF5 is highly expressed during the late anagen phase and promotes the transition from anagen to catagen. The inactivating mutations of *FGF5* display a long-haired phenotype through extending the anagen phase [40]. Therefore, *LHX2* would be expressed strongly and continuously at the prolonged anagen stage when an individual contains both deletion variants. Furthermore, the upregulated expression of the *LHX2* gene maintains the function of hair follicle stem cells and activates genes related to hair fiber structure and function [41]. Alternatively, hair matrix cells may have a finite capacity for proliferation and an improvement for hair follicle structure and function due to a lack of the enhancer of *FGF5* and the insulator of *LHX2* together. Overall, the downregulated expression of *FGF5* and the upregulated expression of *LHX2* gene may form an interaction effect in the same period, thus significantly affecting the development of cashmere fiber. In addition to *LHX2* and *FGF5*, we also identified a few positively selected genes related to cashmere traits, 4 genes (*AR, JAK2, EDA2R*, and *STK3*) affecting I-κB kinase/NF-κB signaling and 5 genes (*NOTCH1, TCF7L1, AR, VCAN*, and *WNT8B*) influencing the canonical WNT signaling pathway (Supplementary Table S5). We found that *NOTCH1, TCF7L1*, and *STK3* exhibited similar seasonal expression patterns with *JAK2, FGF5*, and *EDA2R* in the skin, but *VCAN* and *WNT8B* genes showed low expression (Supplementary Fig. S23; PRJNA470971). NF-κB and WNT signaling pathways play a central role in hair follicle development and regeneration [42–44]. Interestingly, *LHX2* is also regulated by NF-κB signaling to promote primary HF morphogenesis [45]. *EDA2R* is a divergent gene between cashmere goats (excluding UC goats) and ordinary goats, consistent with previous research [7]. *EDA2R* is highly expressed in the late anagen phase and may inhibit hair growth by inducing *DKK-1* expression [46]. Furthermore, the *AR* gene and *JAK2* are novel genes identified in cashmere traits. *JAK2* can restrain the hair follicles in catagen via the JAK/STAT pathway with upregulation in catagen and telogen stages [47, 48]. In contrast, AR has paradoxically different effects on hair follicles [49]. In cashmere goats, *AR* is expressed in the early anagen stage and may stimulate cashmere growth via the *IGF-1* pathway [50]. These positively selected genes may be involved in cashmere trait formation through the model described in Fig. 6E.

### Conclusions

Through population genomics and selective sweep analyses of cashmere and ordinary goats, we identified a novel causative mutation upstream of *LHX2* that functions as an insulator to block the enhancer of *LHX2*. In contrast to enhancer deletion of *FGF5*, this insulator deletion also retained high allele frequency in wild goats, ancient goats, and ibex. We also found that the insulator deletion was associated with 2 cashmere traits in the CDMC goat population. The positively selected genes were enriched in the NF-κB and Wnt pathways, which play a central role in hair follicle development. This study not only provides the first evidence of how cashmere fiber growth could be regulated during the evolution of goats but also offers valuable molecular markers for the genetic improvement of cashmere traits in goats.

## Materials and Methods

### Sample collection

We collected 120 domestic goats (*Capra hircus*), representing 8 geographically diverse breeds in China, which include 30 CDMC from Chaidamu City of Qinghai Province, 11 UC from Ujimqin Banner of Inner Mongolia Province, 8 GZB from Guiyang City of Guizhou Province, 11 YNBB from Lanping County of Yunnan Province, 10 MT from Shangqiu City of Henan Province, 10 JNG from Jining City of Shandong Province, 10 JTB from Jintang County of Sichuan Province, and 12 CDB from Chengdu City of Sichuan Province. Details of the samples used in this study are provided in Supplementary Table S1. All animal procedures were approved by the ethics and biological safety review committee of BGI (No. FT 18041) and were carried out in accordance with the approved guidelines.

### DNA extraction and sequencing to further evaluate whether these 2 deletion variants were related to cashmere

Ear or blood tissues of CDMC, UC, JT, GZB, JNG, YNBB, CDB, and MT were collected on-site and stored in an alcohol sampling tube or blood collection tube. A tissue DNA extraction kit was used to extract the genomic DNA from the samples, electrophoresis was used for integrity detection, and Qubit was used for concentration determination. Library construction for resequencing was performed with 1 to 3 µg genomic DNA using standard library preparation protocols and insert sizes from 200 to 400 bp. All 120 goats were sequenced on the BGISEQ-500 platforms, BGI Shenzhen, China (BGISEQ-500, RRID:SCR_017979) with PE100 (CDMC, UC, JT, YNBB, CDB, MT) and on the Illumina HiSeq2500 platforms (Illumina HiSeq 2500 System; Illumina, San Diego, CA, USA; RRID:SCR_016383) with PE150 (GZB, JNG). Furthermore, we downloaded published genomic data of modern, wild, and ancient goats from NCBI databases (Supplementary Table S2, Supplementary Table S3, and Supplementary Table S9).

### Read alignment and variant calling

We obtained raw data from a total of 236 goat samples, including 120 sequenced and 116 downloaded. All raw data were first filtered and trimmed using SOAPnuke (RRID:SCR_015025) v1.5.0 [51] if any of the following criteria were met: (i) reads containing adapter and poly-N, (ii) reads whose low-quality base ratio (base quality less than or equal to 5) is more than 50%, and (iii) reads whose unknown base (“N” base) ratio is more than 10%. Clean reads from all individuals were aligned to the goat reference genome ARS1 [52] by the Burrows–Wheeler Aligner (BWA version 0.7.12, RRID:SCR_010910). SAMtools [53] (RRID:SCR_002105) was used to convert the file format from SAM to BAM and filter the unmapped and nonunique reads. Picard (RRID:SCR_006525) v.1.54 was used to sort the BAM files and remove potential PCR duplications if multiple read pairs had identical external coordinates.

GATK (RRID:SCR_001876) v4.0.11 tools were used in the whole process of variant calling [54]. After mapping, the “HaplotypeCaller,” “CombineGVCFs,” and “GenotypeGVCFs” in GATK4 were used to detect SNPs and indels with default parameters. The output VCF file was then screened for SNPs using “SelectVariants function” of GATK4. SNPs and indels were separated using the GATK tool “SelectVariants” and subjected to rigorous processing to exclude false positives. To obtain high-quality SNPs, we carried out SNP filtering at 2 stages. SNP exclusion criteria [55] were as follows: (i) hard filtration with parameter “QD < 2.0 || ReadPosRankSum < −8.0 || FS > 60.0 || MQ < 40.0 || SOR > 3.0 || MQRankSum < −12.5 || QUAL < 30” and (ii) “–max-missing 0.9 –maf 0.05 –min-alleles 2 –max-alleles 2.” Finally, ∼13 million high-quality SNPs were obtained for further analysis.

### Population structure analysis

We used 12,861,877 high-quality (autosomal) SNPs for PCA analysis. PLINK (version 1.9, RRID:SCR_001757) [56] was used to calculate the principal components, using function “–vcf vcf –out pca –pca –chr-set 29 –allow-extra-chr.”

An individual-based NJ tree was constructed for the 236 goats based on the p-distance, with 1 outgroup (IRW) using the software VCF2Dis (RRID:SCR_022513) v1.09 and PHYLIP (RRID:SCR_006244) v.3.69 [57]. The Linux command line of converting to a matrix is “VCF2Dis -InPut vcf -OutPut p_dis.mat”; the Linux command line of constructing a phylogenetic tree is “fneighbor -datafile p_dis.mat -outfile goat.tree.txt -matrixtype s -treetype n -outtreefile goat.n.tree.tree.”

Population structure was analyzed using the ADMIXTURE (version 1.23, RRID:SCR_001263) [58] program, which implements a block-relaxation algorithm. To explore the convergence of individuals, we predefined the number of genetic clusters *K* from 3 to 7 and ran with cross-validation (CV) error procedure. Default methods and settings were used in admixture analysis. The analysis process includes the following 3 steps:

1. Converting VCF format to PLINK format. The Linux command line was “vcftools –vcf vcf –plink –out goat.plink.”
2. Using PLINK for further filtering with Linux command line “plink –noweb –file plink –geno 0.05 –maf 0.05 –hwe 0.0001 –chr-set 29 –make-bed –out QC”; after this step, we get the corresponding bed file.
3. ADMIXTURE v1.23 for population structure analysis with “admixture –cv QC.bed $k | tee log${k}.out”; then the results were plotted using R.

PopLDdecay(RRID:SCR_022509) [59] for linkage disequilibrium decay analysis was based on variant call format files.

### Demographic history

A population-level admixture analysis was conducted in the TreeMix (version 1.12, RRID:SCR_021636) [60]. The program inferred the (ML) Maximum Likelihood tree for 12 goat breeds (215 individuals) and an out-group (Bezoars, 21 individuals), and then the residuals matrix was used to identify pairs of populations that showed poor fits in the ML tree. These populations were regarded as candidates around which we added potential migration edges, and new arrangements of the ML tree accounting for migration events were generated [60]. From 1 to 20 migration events were gradually added to the ML tree until 98% of the variance between the breeds could be explained. The command was “-i input -bootstrap -k 500 -root IRW -o output.”

We used the PSMC (version 0.6.5, RRID:SCR_017229) [22] method to estimate changes in the effective population size (Ne) of goat over the past 1 million years. The PSMC analysis was implemented in the 7 samples sequenced at a high read depth (12.6∼26.94×) (Supplementary Table S10). The parameters of PSMC were set to -N25 -t15 -r5 -p “4+25*2+4+6,” where the parameters of generation time and mutation rate were set to 2.0 and 2.5e-8, respectively.

### Selective sweep analysis

In the selection sweep, we calculated the genome-wide distribution of Fst values and θ_π_ ratios (i.e., θ_π-UC_/θ_π-Ordinary_, θ_π-UC_/θ_π-CDMC.IMC.LNC_, θ_π-LNC.IMC_/θ_π-Ordinary_, θ_π-White_/θ_π-Brown_, and θ_π-White_/θ_π-Black_) for 5 control group pairs by VCFtools (RRID:SCR_001235) [61], which included the UC group versus the ordinary group (GZB, YNBB, JTB, CDB), the UC group versus the other cashmere group (CDMC, IMC, LNC), the other cashmere group (CDMC, IMC, LNC) versus the ordinary group (GZB, YNBB, JTB, CDB), the white coat color (UC, CDMC, IMC, LNC) versus the brown coat color (CDB), and the white coat color (IMC, LNC) versus the black coat color (YNBB, GZB) using a sliding-window approach (150-kb windows with 10-kb increments). The Fst values were Z-transformed, and the θπ ratios were log_2_-transformed. We considered the windows with the top 1% values for the ZFst and log_2_(θ_π_ ratios) simultaneously as the candidate outliers under strong selective sweeps. All of the outlier windows were assigned to corresponding SNPs and candidate genes. The signals were further confirmed by ZFst, log_2_(θ_π_ ratios), and Tajima's *D* with a 10-kb sliding window.

### Functional enrichment analyses

Candidate genes under selection were defined as those overlapping sweep regions or within 500 kb of the signals. The biological function of genes within candidate regions was annotated by analyzing Gene Ontology (GO) and KEGG pathways using Metascape [62]. Benjamini–Hochberg false discovery rate (FDR) was used for correcting the *P* values. The GO categories “Molecular Function,” “Biological Process,” and “Cellular Component” and Human Phenotype (HP) categories were used in these analyses.

### Genotypic and phenotypic analysis

***Genotyping**.* Genotypic and phenotypic analysis DNA was extracted from the ear tissue samples. The 582-bp deletion near *LHX2* (582del) and the 504-bp deletion of *FGF5* (504del) were genotyped by PCR amplification using the reaction condition of the 2-minute pre-denaturation at 95°C, 15-second denaturation at 95°C, 30-second annealing at 56°C, 45-second extension at 72°C for 30 cycles, and 5-minute extension at 72°C (the primers of the 582-bp deletion: 5′-CAGTACGGAGCAAGTAAACGG-3′ and 5′- ACCATTCCACTTGTCCACCT-3′, the primers of the 504-bp deletion: 5′-ACAGCGTGTGATCTTTTCTCTG-3′ and 5′-TCTTGGTCTGGCTGTGATCA-3′) and visualized on 2% agarose gels (Supplementary Table S11). The 582del and 540del were successfully genotyped in the extended population of 840 goats, including 752 CDMC, 12 IMC, 40 UC, 12 JNG, 12 Wushan white goats (from Chongqing in the southwest of China), and 12 JTB individuals.

***Phenotypic analysis***. All 581 cashmere samples were collected from the lateral body part of CDMC goats, and the fiber diameter was measured by an optical-based fiber diameter analyzer (OFDA2000). Sample preparation is the most critical part of obtaining accurate results from OFDA. Briefly, the cashmere fibers are roughly aligned in parallel and cut into 2 mm by the OFDA bench guillotine and then washed the fibers to remove grease. After drying, a spreader was used to evenly spread 10 to 15 mg of fibers on the glass slide. The slide was placed on the stage and then enlarged, and the snippets were scanned through the optical transmission microscope. The camera system collects the fiber image, and then the fiber in it is automatically identified through image analysis technology and the diameter is measured [63]. In addition, we measured cashmere yields from a subset of 351 CDMC goats in May 2020. The cashmere production was measured by hand-combing cashmere and weighing. First, a thin comb was used to comb the feces and other dirt along the direction of the hair, and then a density-comb was used to comb the direction of the hair repeatedly and finally comb the hair in the reverse direction until the fallen fibers were combed. The collected cashmere was weighed with an electronic scale (range, 0.3 to 5 kg, accuracy 0.1 g). Then, we tested if the identified deletion variants were associated with cashmere diameter and production, respectively. In total, 581 individuals were recorded; the linear model was expressed as y = b0 + bx + sex + batch + e, where y was the diameter; x was the deletion coding, with 0, 1, and 2 representing 0, 1, and 2 copies of the deletion sequence, respectively; b was the effect of deletion variant; sex was the sex effect; batch was the recorder effect, where 2 recorders were participated in the phenotyping; and e was the residual error, assumed to follow normal distribution. When the association between the deletion variants and the production was performed, 235 individuals were included, all females. The same linear model used above was employed to perform the association study for production. The association studies were performed with the lm function of R package [64].

### RNA-seq

RNA-seq data were downloaded from the NCBI database project ID SRP145408. Raw data were filtered by SOAPnuke (version 1.5.6), and read base N more than 5% and read base quality less than 20 were removed. Clean reads from the 12 individual samples were aligned to the reference genome (GCA_001704415.1 ARS1) using hisat2 (version 2.1.0, RRID:SCR_015530). Picard-tools-1.105 was used to sort BAM files. Based on the mapped reads and goat reference genome annotation in GFF (GCF_001704415.1), StringTie (version 1.3.3b, RRID:SCR_016323) was used to calculate the FPKM [65].

### Dual-luciferase reporter analysis

***Plasmid constructs***. A 563-bp DNA fragment containing “5′ end–KpnI/HindIII site–Ins sequence (551 bp)–XhoI/NcoI site” was synthesized by Tsingke Biotechnology Co., Ltd. (BeiJing,China), which was subsequently cloned downstream and upstream of the SV40 promoter in the pGL3 plasmid, named Insulator1 and Insulator2, respectively. A 796-bp DNA fragment containing “5′ end–BamHI site–Ins sequence (551 bp)–SalI site-sv40 enhancer sequence–PciI site–3′ end” (Supplementary Fig. S17) was synthesized by TsingKe Ltd., which was subsequently cloned downstream of Luc+ Poly (A) in the pGL3 promoter plasmid (Promega, Madison, WI, USA) and named Ens-E. Similarly, the well-known insulator cHS4, linked to the sv40 enhancer (Supplementary Fig. S19), was cloned into a pGL3 promoter plasmid and named cHS4-E.

***Cell culture, transfection, and luciferase reporter assay*.** The 3T3 cells and 293T cells were maintained in Dulbecco's modified Eagle's medium (Gibco, Waltham, MA, USA) with penicillin (100 U/ml), streptomycin (100 µg/ml), and 10% fetal bovine serum at 37°C in a humidified atmosphere of 95% air and 5% CO_2_. Before transfection, 3T3 and 293T cells were seeded into 24-well plates at 1.0 × 10^4^ cells/well. After overnight attachment, transfections were performed using Lipofectamine 2000 (Invitrogen, Carlsbad, CA, USA) according to the manufacturer's instructions. In this experiment, the 2 types of cells were divided into 8 groups, respectively. For each group, plasmid of pGL3-control (Promega), Insulator1 and Insulator2 or Ins-E, and cHS4-E were cotransfected with phRL-TK (Promega) at a ratio of 10:1 (0.8/0.08 µg) into the 3T3 and 293T cells, respectively. The cells were harvested 2 days after cotransfection, and luciferase activity was evaluated using the Dual-Luciferase Reporter Assay System (Promega, #E1910). Firefly luciferase data were normalized to *Renilla* luciferase activity. For this assay, pGL3-control was used as a positive control and Lipofectamine 2000 was used as a blank control. The cHS4 insulator was chosen as a reference to quantify the efficiency of the insulator function.

## Data Availability

All sequence data obtained in the current study were uploaded to the NCBI Sequence Read Archive (BioProject accession number PRJNA863495). The data that support the findings of this study have been also deposited into CNGB Sequence Archive (CNSA) [66] of China National GeneBank DataBase (CNGBdb) [67] with accession number CNP0001896. All supporting data and materials are available in the *GigaScience* GigaDB database [68].

### Abbreviations

ANG: Angora goat; BLAST: Basic Local Alignment Search Tool; BWA: Burrows–Wheeler Aligner; CDB: Chengdu brown goat; CDMC: Chaidamu cashmere goat; CNE: Conserved Noncoding Element; CTCF: CCCTC-Binding Factor; DRG: dorsal root ganglia; FDR: false discovery rate; FPKM: fragments per kilobase million; GATK: Genome Analysis Toolkit; GO: Gene Ontology; GZB: Guizhou black goat; HP: human phenotype; IGV: Integrative Genomics Viewer; IMC: Inner Mongolia cashmere goat; Ins: insulator; IRW: Iranian wild goat; JNG: Jining gray goat; JTB: Jintang black goat; KEGG: Kyoto Encyclopedia of Genes and Genomes; Kb: kilobase pairs; KO: Korea goat; LNC: Liaoning cashmere goat; MT: Matou goat; NCBI: National Center for Biotechnology Information; OFDA: optical-based fiber diameter analyze; PSMC: Pairwise Sequential Markovian Coalescent; RNA-seq: RNA sequencing; SNP: single-nucleotide polymorphism; SRA: Sequence Read Archive; UC: Ujumqin cashmere goat; WGS: whole-genome sequencing; WSW: Wushan white goat; YNBB: Yunnan black bone goat.

## Authors' Contributions

L.Y, F.M., and Z.S.Y. conceived, designed, and supervised the study. H.H., F.M., L.Y., J.D., C.T., and M.Z.P. performed the informatics analysis of the sequencing data. Z.S.Y., Y.M.M., W.Q., Y.C.Y., W.Q.J., W.L.B., G.G., Mengkedala, D.W.D., L.B., and Z.Q.F. obtained goat material and DNA for resequencing. Z.S.Y., Y.M.M., W.Q.J., W.L.B., G.G., Mengkedala, and L.B. performed phenotypic data collation and analysis. Y.M.M., W.R., J.D., W.Q., and Z.T.T. participated in the laboratory work. Z.X.J., Z.T.T., and L.L. performed dual-luciferase reporter analysis. C.T. analyzed the transcriptome. L.Y., H.H., Y.M.M., J.D., and Z.X.J. are the major contributors in writing the manuscript. All authors read, revised, and approved the final manuscript.

## Additional Files

Supplementary information accompanies this paper at Supplementary.docx and Supplementary table s.xlsx.

Supplementary Fig. 1. PCA analysis.

Supplementary Fig. 2. Results of genetic structure analysis of 13 goat breeds.

Supplementary Fig. 3. Results of genetic structure analysis of 10 Chinese goat breeds.

Supplementary Fig. 4. Phylogenetic tree of 10 Chinese native goat breeds.

Supplementary Fig. 5. Inferred goat tree of mixture events deduced by TreeMix.

Supplementary Fig. 6. Bar graph of enriched terms across positively selected genes with GO term enrichment analysis.

Supplementary Fig. 7. Analysis of the signatures of positive selection in the genome of cashmere and ordinary goat breeds.

Supplementary Fig. 8. Analysis of the signatures of positive selection in the genome of goat breeds with white and black coat color.

Supplementary Fig. 9. Analysis of the signatures of positive selection in the genome of goat breeds with white and brown coat color.

Supplementary Fig. 10. Ratios and ZFst values around the ASIP locus.

Supplementary Fig. 11. CNVs at the KIT locus.

Supplementary Fig. 12. CNVs at the ASIP locus.

Supplementary Fig. 13. Visualization of whole genome sequencing data identifies 582 bp deletion in the ancient goat.

Supplementary Fig. 14. Visualization of Whole Genome Sequencing Data identifies 582 bp deletion near LHX2 in the ibex goats.

Supplementary Fig. 15. The pattern of SNP genotypes near DEL among 13 goat breeds.

Supplementary Fig. 16. Distribution diagram of cashmere yield data.

Supplementary Fig. 17. Distribution of cashmere diameter measurements.

Supplementary Fig. 18. The expression of LHX2, JAK2 and Notch1 in different days (from 45 d to 135 d) of fetal skin.

Supplementary Fig. 19. Schematic drawing of the construction of DNA plasmids.

Supplementary Fig. 20. The expression of LHX2 and AR in different months of a year.

Supplementary Fig. 21. The expression of JAK2 and EDA2R as well as FGF5 in different months of a year.

Supplementary Fig. 22. The PCR identification of deletions in sheep.

Supplementary Fig. 23. The expression of STK2 and NOTCH1 as well as TCF7L1 in different months of a year.

Supplementary Table 1. Overview of sequencing information of 120 goats.

Supplementary Table 2. Sequencing information of 95 published goats used in this study.

Supplementary Table 3. Sequencing information of Wild goats used in this study.

Supplementary Table 4. Sequencing platforms for 236 goats of 13 goat breeds.

Supplementary Table 5. The genotype frequencies of the homozygous 582bp deletion (-/-) at LHX2 locus for 15 goat breeds, obtained from whole genome sequencing.

Supplementary Table 6. The genotype frequencies of the homozygous 504bp deletion (-/-) at FGF5 locus for 15 goat breeds, obtained from whole genome sequencing.

Supplementary Table 7. The frequencies of the 582 bp deletion near LHX2 with PCR amplification.

Supplementary Table 8. The frequencies of the 504 bp deletion near FGF5 with PCR amplification.

Supplementary Table 9. Published ancient samples used in this study.

Supplementary Table 10. Samples for PSMC analysis.

Supplementary Table 11. Primers used for amplification of deletion sequence.

Supplementary Table s1. Variant analysis.

Supplementary Table s2. The candidate genes in the selection sweep of UC goats and ordinary goats.

Supplementary Table s3. The candidate genes in the selection sweep of UC goats and other cashmere goats.

Supplementary Table s4. Annotation of candidate genes in UC goats and other cashmere goats to perform selective evolution analysis.

Supplementary Table s5. Enrichment of candidate genes in UC goats and other cashmere goats to perform selective evolution analysis.

Supplementary Table s6. The candidate genes in the selection sweep of other cashmere goats and ordinary goats.

Supplementary Table s7. The candidate genes in the selection sweep of white cashmere goats (CDMC, UC, IMC, ILC) and brown cashmere goats (CDB).

Supplementary Table s8. The candidate genes in the selection sweep of white cashmere goats (IMC, LNC) and black cashmere goats (YNBB, GZB).

Supplementary Table s9. Variant analysis of DENND1A and LHX2 gene.

Supplementary Table s10. Cashmere yield and Genotype.

Supplementary Table s11. Cashmere diameter and Genotype.

giac107_GIGA-D-22-00003_Original_Submission

giac107_GIGA-D-22-00003_Revision_1

giac107_GIGA-D-22-00003_Revision_2

giac107_GIGA-D-22-00003_Revision_3

giac107_GIGA-D-22-00003_Revision_4

giac107_Response_to_Reviewer_Comments_Revision_1

giac107_Response_to_Reviewer_Comments_Original_Submission

giac107_Response_to_Reviewer_Comments_Revision_2

giac107_Response_to_Reviewer_Comments_Revision_3

giac107_Reviewer_1_Report_Original_Submission Mahesh Neupane -- 2/7/2022 Reviewed

giac107_Reviewer_2_Report_Original_Submission Yixue Li -- 3/10/2022 Reviewed

giac107_Reviewer_3_Report_Original_Submission Yu Jiang, Ph.D -- 3/15/2022 Reviewed

giac107_Reviewer_3_Report_Revision_1 Yu Jiang, Ph.D -- 6/20/2022 Reviewed

giac107_Reviewer_3_Report_Revision_2 Yu Jiang, Ph.D -- 7/4/2022 Reviewed

giac107_Supplemental_Files

## Competing Interests

The authors declare no competing interests.

## Funding

This work was supported by the National Natural Science Foundation of China (31872560), the Science and Technology Innovation Strategy Projects of Guangdong Province (2019B020203002), the Basic Research Project of Haixi Agriculture and Animal Husbandry Bureau (Qinghai Caidamu Cashmere Goat Genomic Breeding project), and the Shenzhen Municipal Government of China (JCYJ20180307163440037).
