# Supplementary material for: Selection signatures in goats reveal a novel deletion mutant underlying cashmere yield and diameter
Source: Gigascience. 2022 Nov 3;11:giac107. doi: 10.1093/gigascience/giac107 (PMC9633279; doi:10.1093/gigascience/giac107)
Supplement: giac107_GIGA-D-22-00003_Original_Submission [file giac107_giga-d-22-00003_original_submission.pdf]

GigaScience

Selection signatures in goats reveal a novel deletion mutant underlying cashmere yield and diameter

--Manuscript Draft--

**Manuscript Number:**	GIGA-D-22-00003
**Full Title:**	Selection signatures in goats reveal a novel deletion mutant underlying cashmere yield and diameter
**Article Type:**	Research
**Funding Information:**	Science and Technology Innovation Strategy Projects of Guangdong Province (2019B020203002) — Doctor Yong Li<br>Innovative Research Group Project of the National Natural Science Foundation of China (31872560) — Professor Ming Fang<br>Innovative Research Group Project of the National Natural Science Foundation of China (31672399) — Professor Ming Fang<br>Shenzhen Municipal Human Resources and Social Security Bureau (JCYJ20180307163440037) — Doctor Yong Li
**Abstract:**	Cashmere traits were deployed for fiber yield and quality during the domestication of goats. However, the genetic alterations underlying cashmere trait selection are still unclear. We sequenced 120 Chinese native goats including two cashmere goat breeds and six ordinary goat breeds. The genome-wide selective sweep of cashmere goat and ordinary goat revealed a novel set of candidate genes as well as pathways, such as Nuclear factor kappa-B and Wnt Signaling pathways. Of them, the LHX2 gene regulating hair follicle development, was evident from the strongest selection signal when comparing the Uhumqin cashmere goat and ordinary goat. Interestingly, we identified a 582bp deletion at 367 kb upstream of LHX2 with higher frequency in cashmere goats and their ancient relatives. This mutation probably rises along the breeding procedures, and is putatively responsible for cashmere production and diameter, as revealed by association studies. Luciferase assay shows that the deletion, which acts as an insulator, restrains the expression of LHX2 by interfering its upstream enhancers. Our study discovers a novel insulator of the LHX2 involved in regulating cashmere production and diameter. Our findings also provide new insights into the genetic formation of cashmere, and facilitate subsequent molecular breeding for cashmere goat improvement.
**Corresponding Author:**	Yong Li<br>BGI-Shenzhen: BGI Group<br>Shenzhen, CHINA
**Corresponding Author Secondary Information:**	
**Corresponding Author's Institution:**	BGI-Shenzhen: BGI Group
**Corresponding Author's Secondary Institution:**	
**First Author:**	hu han
**First Author Secondary Information:**	
**Order of Authors:**	hu han
	Man-Man Yang
	Jiang Dan
	Xing-Ju Zhang

Powered by Editorial Manager® and ProduXion Manager® from Aries Systems Corporation

	Qiang Wei
	Tao Chen
	Qi-Ju Wang
	Cheng-Ye Yang
	Bater Wulan
	Ting-Ting Zhang
	Gang Gen
	Dala Mengke
	Bin Li
	Wei-Dong Deng
	Ze-Pu Miao
	Ran Wang
	Qing-Feng Zhang
	Lin Li
	Sheng-Yu Chao
	Ming Fang
	Yong Li
**Order of Authors Secondary Information:**	
**Additional Information:**	
**Question**	**Response**
Are you submitting this manuscript to a special series or article collection?	No
**Experimental design and statistics**<br><br>Full details of the experimental design and statistical methods used should be given in the Methods section, as detailed in our Minimum Standards Reporting Checklist. Information essential to interpreting the data presented should be made available in the figure legends.<br><br>Have you included all the information requested in your manuscript?	Yes
**Resources**<br><br>A description of all resources used, including antibodies, cell lines, animals and software tools, with enough information to allow them to be uniquely identified, should be included in the	Yes

Methods section. Authors are strongly encouraged to cite Research Resource Identifiers (RRIDs) for antibodies, model organisms and tools, where possible.<br><br>Have you included the information requested as detailed in our Minimum Standards Reporting Checklist?	
**Availability of data and materials**<br><br>All datasets and code on which the conclusions of the paper rely must be either included in your submission or deposited in publicly available repositories (where available and ethically appropriate), referencing such data using a unique identifier in the references and in the “Availability of Data and Materials” section of your manuscript.<br><br>Have you have met the above requirement as detailed in our Minimum Standards Reporting Checklist?	Yes

Click here to access/download;Manuscript;Manuscript.docx

Selection signatures in goats reveal a novel deletion mutant underlying cashmere yield and diameter

Hu Han[1,3]*, Man-Man Yang[1,3]*, Jiang Dan[2]*, Xing-Ju Zhang[1,3]*, Qiang Wei[1,3], Tao Chen[1,3], Qi-Ju Wang[4], Cheng-Ye Yang[3], Bater Wulan[5], Ting-Ting Zhang[1,3], Gang Gen[5], Mengkedala[6], Bin Li[1], Wei-Dong Deng[7], Ze-Pu Miao[1], Ran Wang[1], Qing-Feng Zhang[8], Lin Li[1], Sheng-Yu Chao[4,5#], Ming Fang[2#], Yong Li[1,3#]

1. BGI Institute of Applied Agriculture, Shenzhen, 518120, PR China.

2. Key Laboratory of Healthy Mariculture for the East China Sea, Ministry of Agriculture and Rural Affairs, Fisheries College, Jimei University, Xiamen, 361021, Fujian province, PR China

3. BGI-Shenzhen, Shenzhen, 518083, PR China.

4. Animal Epidemic Disease Prevention and Control Center, Haixi Autonomous Prefecture of Qinghai Province, Delingha, Qinghai, 817099, China.

5. Haixi Agricultural Technology Extension Service Center, Haixi Autonomous Prefecture of Qinghai Province, Delingha, Qinghai, 817099, China.

6. Haixi Agricultural Products Quality Safety Inspection and Testing Center, Haixi Autonomous Prefecture of Qinghai Province, Delingha, Qinghai, 817099, China.

7. Faculty of Animal Science and Technology, Yunnan Agricultural University, Kunming, 650201, PR China

8. The Enterprises Key Laboratory of Tianjin Mutton Sheep Genetics and breeding, Tianjin Aoqun Animal Husbandry Pty.Ltd，Tianjin，301607,PR China

Corresponding author:
Name: Yong Li
E-mail: liyong3@genomics.cn

Abstract

Cashmere traits were deployed for fiber yield and quality during the domestication of goats. However, the genetic alterations underlying cashmere trait selection are still unclear. We sequenced 120 Chinese native goats including two cashmere goat breeds and six ordinary goat breeds. The genome-wide selective sweep of cashmere goat and ordinary goat revealed a novel set of candidate genes as well as pathways, such as Nuclear factor kappa-B and Wnt Signaling pathways. Of them, the *LHX2* gene regulating hair follicle development, was evident from the strongest selection signal when comparing the Uhumqin cashmere goat and ordinary goat. Interestingly, we identified a 582bp deletion at 367 kb upstream of *LHX2* with higher frequency in cashmere goats and their ancient relatives. This mutation probably rises along the breeding procedures, and is putatively responsible for cashmere production and diameter, as revealed by association studies. Luciferase assay shows that the deletion, which acts as an insulator, restrains the expression of *LHX2* by interfering its upstream enhancers. Our study discovers a novel insulator of the *LHX2* involved in regulating cashmere production and diameter. Our findings also provide new insights into the genetic formation of cashmere, and facilitate subsequent molecular breeding for cashmere goat improvement.

Keywords: *LHX2*, selective sweep, insulator, whole genome sequencing, cashmere trait

Introduction

Cashmere contributes high economic value to the textile industry. Cashmere is made from the processing of the underwool of goats that are grown in high and cold regions of the world, such as the Tibetan plateau and Mongolia. China is the largest cashmere producer globally, accounting for approximately 75% of the world's supply(WaldronBrown and Komarek 2014). Improvement in the quantity and quality of cashmere is an important breeding goal in goat farming.

The cashmere goats with a long fine underwool, share a common ancestor with

other ordinary goats that were domesticated from wild goat (bezoar) approximately 11,000 years ago in the Fertile Crescent of southwest Asia and adjacent areas(Zeder 2008; Daly et al. 2018). Almost all the breeds of goats can produce more or less cashmere fibers, but only the breeds with sufficiently fine hair are called cashmere goats (cashmere goats in China produce 250 g to 500g of fiber, while only 50g in ordinary goats)(Ryder 1993). Previous research postulated that cashmere goats would not be domesticated locally, except several highland regions including Himalayas, Mongolia, and Kirghizia(Millar 1986). This kind of domestication leads to selecting more and more cashmere wool to meet textile demands. Therefore, in contrast to cashmere fiber formation, cashmere production was pursued by intense artificial selection pressure during the domestication of cashmere goats(Li et al. 2017; Cai et al. 2020; Jin et al. 2020).

Previous transcriptome studies in cashmere goats have already found many important pathways that are involved in cashmere fiber development, including *WNT, FGF5, BMP, TGF-β, NOTCH, SHH,* and some of them may play important roles in secondary hair follicle development, such as *LHX2, FGF5* and *TGF-β4*(GengYuan and Chen 2013; Geng et al. 2014; Gao et al. 2016; Su et al. 2018; Zhang et al. 2019; Dai et al. 2020; Zhang et al. 2020). Meanwhile, some genes, such as *FGF5, SGK3, IGFBP7, OXTR, ROCK1, LHX2, FGF9, PRDM6* and *WNT2* were intensively selected in cashmere goats(Wang et al. 2016; Li et al. 2017; Zhang et al. 2018). Recent studies have identified several causal genes for cashmere growth trait, including *FGF5* and *EDA2R*(Cai et al. 2020; Guo et al. 2020). *FGF5* is a regulator of the hair growth cycle, and the disruption of *FGF5* is associated with a long hair characteristic(Wang et al. 2016; Li et al. 2019). Interestingly, the enhancer-absent *FGF5* was only found in domesticated goats rather than in the wild goats, indicating this causative mutation occurred in domestication process(Cai et al. 2020; Li et al. 2020). However, more researches are necessary to elucidate the genetic basis of cashmere fiber formation in goats after divergence from sheep.

Here, we generated genomic data from 42 cashmere goats and 78 ordinary goats

in China and conducted comprehensive population genomic analyses. Integrated with goat genomes, we identified genetic footprints under artificial selection during goat migration and domestication. When compared the ancient and wild goat, we also investigate the origin and biological function of causative mutation for cashmere fiber formation.

Results

Population sequencing and genetic diversity

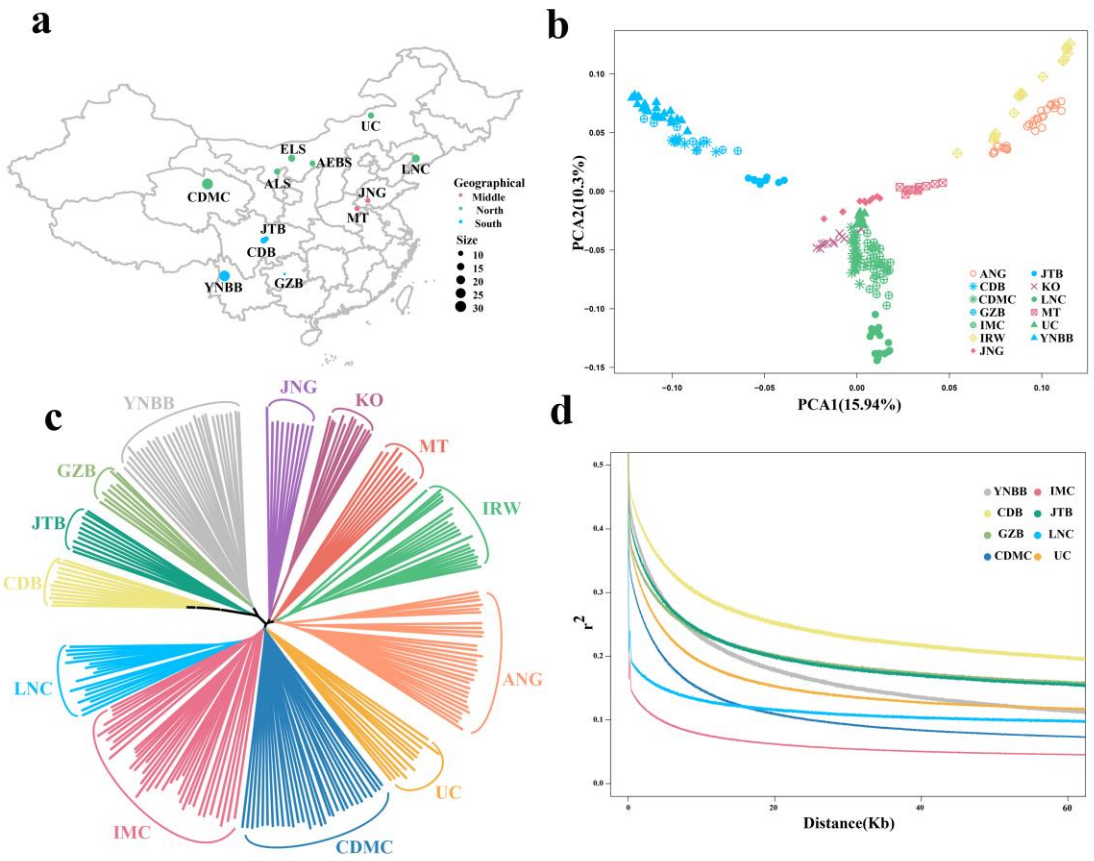

Fig. 1 Geographic distribution and population genetic analyses of 13 goat breeds.
(a) Geographical distribution of domestic goat breeds in China. Green, blue, red represents northern, southwest, middle regions, respectively. **(b)** Principal component (PC) plot of the first two components. The fraction of the variance explained is 15.94% and 10.3% for PC1 and PC2, respectively. **(c)** Neighbor-joining tree constructed using p-distances between different breeds, including LNC (Liaoning cashmere), IMC (Inner Mongolia cashmere), UC (Ujumqin cashmere), CDMC (Chaidamu cashmere), CDB (Chengdu brown), GZB (Guizhou Black), YNBB (Yunnan black bone), JTB (Jintang black), MT (Matou), JNG (Jining gray), KO (Korean), IRW (Iranian wild), and ANG (Angora). **(d)** Linkage disequilibrium decay of goat populations measured by r^2.

A total of 120 domestic goats representing eight geographically diverse breeds in China

were selected for genome resequencing (Supplementary Table 1). These datasets were also analyzed together with the published whole-genome sequencing (WGS) datasets of 116 individuals from five breeds (Supplementary Table 2 and 3). Among the ten domestic goat breeds, Liaoning cashmere goat (LNC), Inner Mongolia cashmere goat (IMC), Ujumqin cashmere goat (UC) and Chaidamu cashmere goat (CDMC) are the cashmere goat breeds located in the north of China; Chengdu brown goat (CDB), Guizhou Black goat (GZB), Yunnan black bone goat (YNBB) and Jintang black goat (JTB) in the southwest and Matou Goat (MT) and Jining gray goat (JNG) in the middle are non-cashmere goat breeds (Fig. 1a). The remaining goat breeds include Korea goat (KO), Iranian wild goat (IRW) and Angora goat (ANG) from Korea, Iran and France, respectively.

A total of 8448.92 Gb paired-end DNA sequence data were obtained from 236 goats. All goats were sequenced with an average 12-fold depth of the genome (4.17~29.9×) and the average genome coverage of 97.82% (Supplementary Tables 1-3). 99.2% reads were mapped to the latest goat reference genome ARS1(GCA_001704415.1) (Supplementary Tables 1-3). We totally detected 13,069,924 high-quality single nucleotide polymorphisms (SNPs), with 0.605% (0.229 million) located in exonic regions (Supplementary Table s10). The average transition-to-transversion (Ti/Tv) ratio was 2.46 for all goat samples, which indicated relatively low potential random sequencing errors (Supplementary Table s10).

The principal component analysis (PCA) of the 236 goats revealed genetically distinct clusters according to their geographic locations. The clustering results of the northern cashmere goat populations (IMC, LNC, UC and CDMC) and ordinary goat populations (YNBB, JTB, GZB and CDB) were clearly separated (Fig. 1b). JNG and MT were divided into subgroups between cashmere goats and the ordinary southwest goats. Samples of Iranian wild goats (IRW) and Angora goats (ANG) (France, South Africa, and Madagascar) were significantly different from those of Chinese native goats (Fig. 1b). This result was confirmed by the phylogenetic tree using the same SNPs (Fig. 1c). In the analysis of linkage disequilibrium (LD), four ordinary goat breeds from southwest China (YNBB, JTB, GZB and CDB) showed an overall slower decay rate

and a higher level of LD than the cashmere breeds from northern China (IMC, LNC, UC and CDMC) (Fig. 1d).

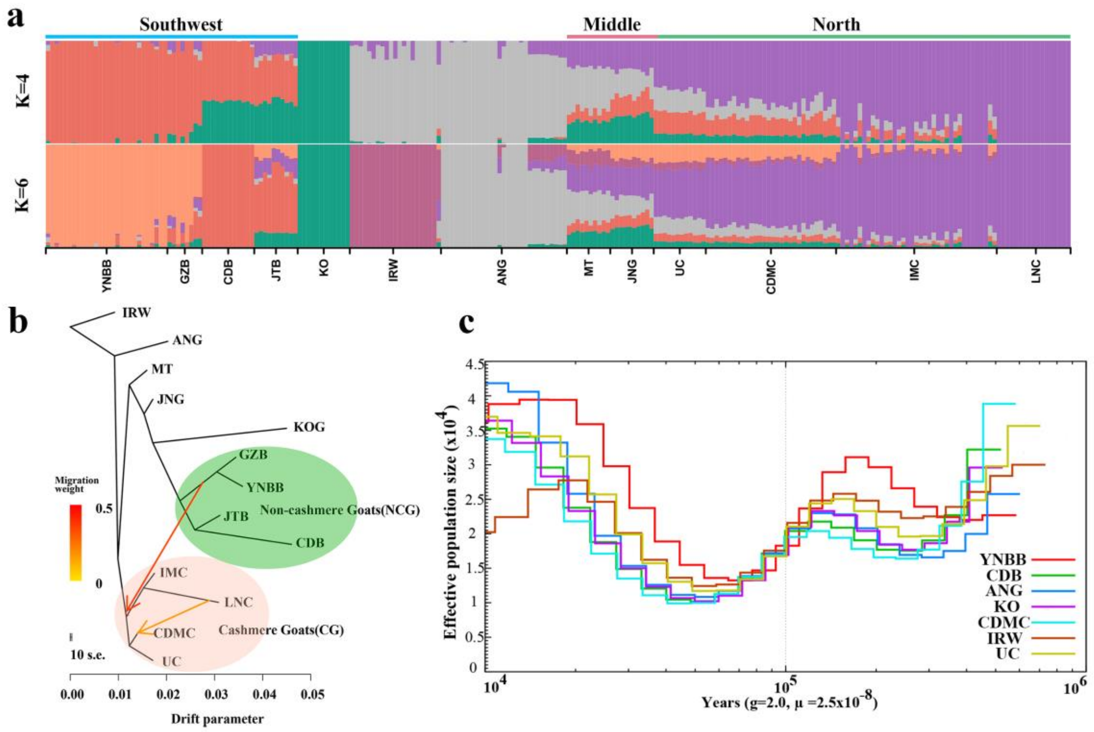

Fig. 2 Population structure, gene flows, and effective population size of goat populations. (a) Model-based population assignment with ADMIXTURE analysis for K = 4 and 6, respectively. The population names are at the bottom of the figure, and the geographic locations are at the top. **(b)** Phylogenetic network of the inferred relationships among the 13 native breeds with two inter-group migration edges being identified. The branch length is proportional to the drift of each population. IRW (Iranian wild goat) was used as the outgroup to root the tree. The colored regions in the phylogenetic tree represent two inferred genetic groups. Arrows indicate migration events, and a spectrum of heat colors indicates the migration weights of the migration events. **(c)** Pairwise sequentially Markovian coalescent analysis for the representative individuals sequenced at a high read coverage, exhibiting inferred variations in Ne over the last 10^6 years. g (generation time) = 2 years; μ (neutral mutation rate per generation) = 2.5× 10−8.

In the STRUCTURE analysis, When K = 4, we observed five separate clusters: IRWG and ANG in west Asia, YNBB, GZB, JTB and CDB in southwest China; cashmere goat in north China; MT and JNG in mddle east China; and Korean goats in south Korea. At K = 6, goats in the southwest China further split into two geographic

subgroups: the Yunnan-Kweichow Plateau group including YNBB and GZB goats, and the Chengdu Plain group including CDM and JTB goats. Two west Asian goats (IRW and ANG) were also separated (Fig. 2a). Some cashmere goats showed evidence of admixture, which may be attributable to shared ancestral polymorphism and recent introgression events by crossbreeding with neighboring domestic goats. Interestingly, the genetic structure of the Chinese goat population revealed that UC and LNC goats represent two different types, and other cashmere goats are a mixture of these two types (Supplementary Fig. 3).

In TreeMix analysis, we found that cashmere goats and ordinary goats clustered into two groups (Fig. 2b). We observed two migration edges among clusters from LNC to CDMC and from YNBB and GZB to the cashmere goat (Fig. 2b), which can be explained by the fact that CDMC goats were crossbred between goats of the Qinghai-Tibet Plateau and LNC.

Seven high-coverage samples were chosen to infer the effective population size (Ne) over the last 10^6 years using the pairwise sequential Markovian coalescent (PSMC) method(Li and Durbin 2011). PSMC analysis suggested that the goats suffered at least two bottlenecks (approximately 7000 and 18000 years ago), which resulted in a severe reduction in the effective population size (Fig. 2c). Interestingly, the results of the PSMC analysis of goats were like the demographic history of the sheep(Yang et al. 2016).

Genome-Wide Selective Sweeps

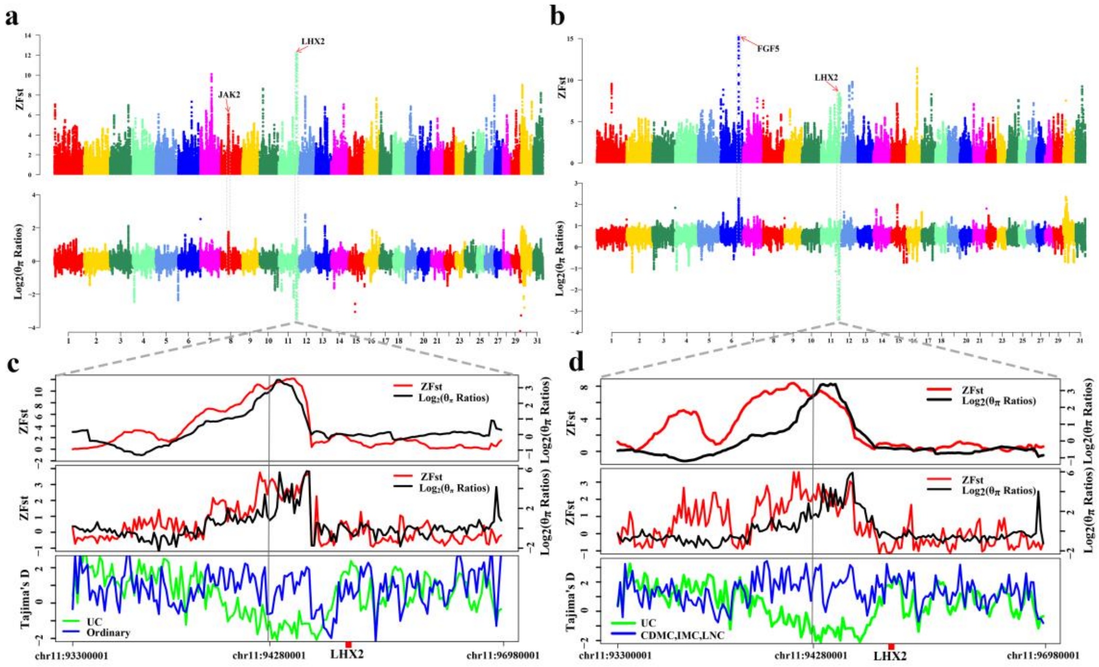

Fig. 3 Genomic regions with selection sweep signals in domestic goats. (a) Manhattan plot of the genome-wide distribution of pairwise ZFst and $\log_2(\theta_\pi$ Ratios) between UC and ordinary goats (YNBB, GZB, JTB and CDB) using a 150 kb window size and a 10 kb step size. **(b)** Manhattan plot of the genome-wide distribution of pairwise ZFst and $\log_2(\theta_\pi$ Ratios) between UC and other cashmere goats (CDMC, LNC and IMC) using a 150 kb window size and a 10 kb step size. **(c)** Zooming in on the peak signal region on chromosome 11. $\mathrm{Log}_2(\theta_\pi$ Ratios), ZFst values, and Tajima's D values around the *LHX2* region (Fig. 3a) using a nonoverlapping 10 kb sliding window. The black and red lines represent $\log_2(\theta_\pi$ Ratios) and ZFst values, respectively; the blue and green lines represent the ordinary goats and UC goats, respectively. All three methods indicate a strong selection signature around the *LHX2* region. **(d)** $\mathrm{Log}_2(\theta_\pi$ Ratios), ZFst values, and Tajima's D values around the *LHX2* region (Fig. 3b).

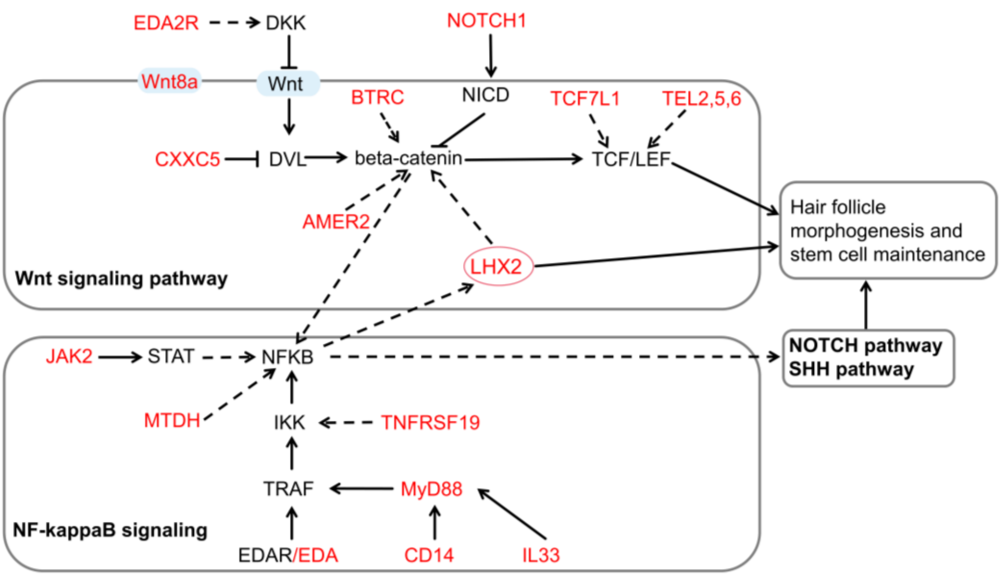

Fig. 4 Schematic mechanisms of signaling pathways involved in cashmere fiber development. The names of the KEGG pathways are shown in bold. The candidate genes positively selected in the two methods of Fst and θπ ratio tests are shown in red. The solid block arrows represent direct effect, and the dashed black arrows indicate an indirect effect. The blunt head arrow indicates an inhibition effect.

The results of the genetic structure analysis showed that the two cashmere goats (UC and CDMC) had apparent admixture with some other ordinary breeds. In contrast to CDMC bred through crossbreeding between Qinghai native goats and LNC, UC is originally from the Ujumuqin region and is continually selected for cashmere traits. Therefore, the distinct genetic background of UC raised the question of whether there is a different selective sweep region for the cashmere trait. We then performed a selective sweep analysis with the 12 UC and 58 ordinary goat genome sequences by estimating pairwise genetic differentiation (Fst) and nucleotide diversity differences (θπ) in 150 kb sliding windows along the genome. Using the top 1% of ZFst values and $\log_2(\theta_\pi$ Ratios) cutoffs (ZFst > 4.01, the absolute value of $\log_2(\theta_\pi$ Ratios) > 0.85), we identified 201 candidate genes associated with cashmere traits (Fig. 3a, Supplementary Table s1). We found that the most prominent ZFst signature was on chromosome 11 (Fig. 3a), spanning ~1,100 kb region (93.6–94.7 Mb). This signature was also supported by the ZFst, $\log_2(\theta_\pi$ Ratios), and Tajima's D statistics (Fig. 3c). However, we did not identify the selection signal harboring *FGF5*, which has been identified in previous

reports(Cai et al. 2020). We next used UC and other cashmere goats to perform selective sweep analysis and identified 263 genes corresponding to selective sweeps (Fig. 3b, Supplementary Table s2). Interestingly, both selection signals on chromosome 6 and *FGF5* were simultaneously identified in these populations. Among all the candidate genes, eight (*EDA*, *MyD88*, *CD14*, *IL33*, *TNFRSF19*, *LHX2*, *AR*, *STK3* and *JAK2*) were located in the NF-kappaB signaling pathway and eleven (*TCF7L1*, *WNT8B*, *BTRC*, *AMER2*, *TEL2*, *TEL5*, *TEL6*, *LHX2*, *CXXC5*, *NOTCH1* and *VCAN*) were found in the Wnt/ß-catenin signaling pathway (Fig. 4). These signaling pathways play a central role in regulating hair follicle morphogenesis, stem cell differentiation and hair cycle(Huelsken et al. 2001; Rishikaysh et al. 2014). The strongest selective sweep localized on chromosome 11, harbored DENN domain-containing protein 1A (*DENND1A*), Crumbs cell polarity complex component 2 (*CRB2*) and LIM homeobox 2 (*LHX2*), in which *DENND1A* and *CRB2* are associated with polycystic ovary syndrome (PCOS) and maintenance of apicobasal polarity in retinal pigment epithelium, respectively, but without evidence in hair development (McAllister et al. 2014; Paniagua et al. 2021); while another gene *LHX2* near *DENND1A*, plays important role in hair follicle development.

To confirm the reliability of our findings, we further used other cashmere goats (LNC, IMC and CDMC) and ordinary goats to perform sweep selection analysis. The results showed that all previously-detected functional genes for cashmere traits including *FGF5*, *EDA2R* and *STIM1*, were re-identified (Supplementary Fig. 6)(Cai et al. 2020; Li et al. 2020). In addition to the cashmere trait, we also investigated the selection sweep for coat colors and confirmed previously reported genes *KIT*, *KITG*, *IRF4* and *ASIP* for coat color using different goat breeds (Supplementary Fig. 7 and Supplementary Fig. 8). Furthermore, we also identified previously reported 100 kb copy number variants compassing *KIT* (chr6:70,859,258-70,959,918) and ~154 kb copy number variants within *ASIP* (chr13:63,226,824-63,381,501) with single base-pair resolution (Supplementary Fig. 10 and Supplementary Fig. 11).

Plausible Causative Mutation near *LHX2*

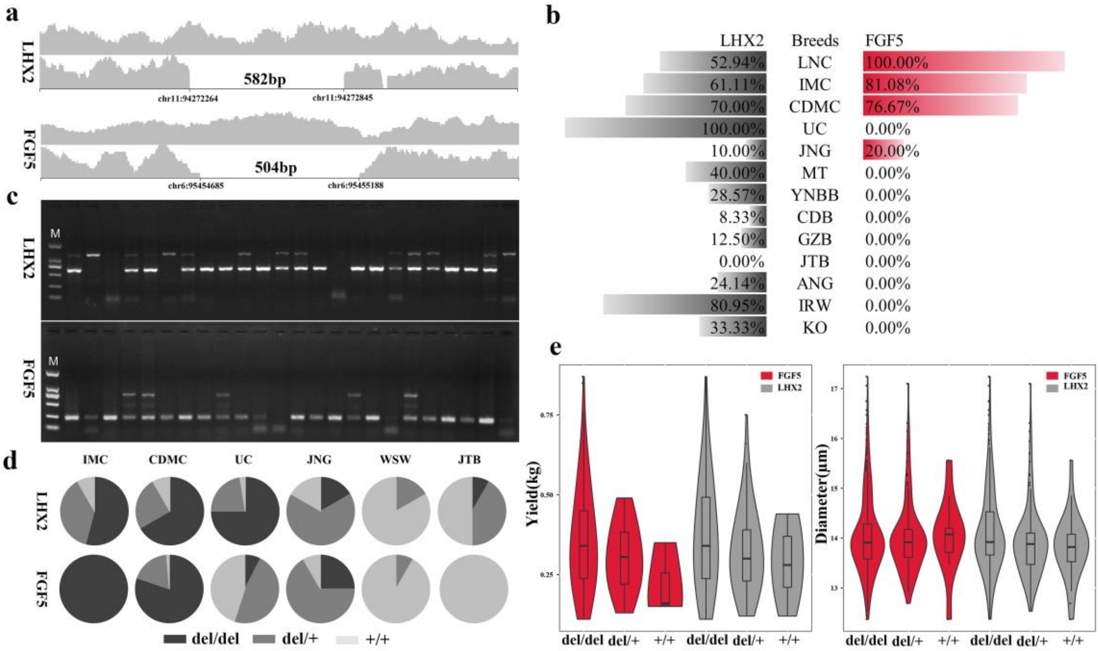

Fig. 5 The *LHX2* and *FGF5* deletions in different goat breeds and their association with cashmere traits in CDMC.

(a) The identified 582 bp deletion near *LHX2* and the previously reported 504 bp deletion near *FGF5* based on read coverage. **(b)** Homozygous genotype frequency of the two deletions determined by IGV viewer **(c)** PCR amplification of the two deletion variants. **(d)** Distribution of the 582 bp deletion near *LHX2* and the 504 bp deletion of *FGF5* genotypes. del/del, deletion/deletion; del/+, deletion/wild type; +/+, wild type/wild type. WSW (Wushan White goat from southwest China). **(e)** The association of the 582 bp deletion near *LHX2* and 504 bp deletion of *FGF5* with cashmere yield and diameter in the CDMC goat population.

We inspected all variants within exons to identify the potential causal mutation around the *DENND1A-LHX2* locus; however, no coding variants were found. Strikingly, a 582 bp (chr11: 94,272,264-94,272,845 bp) deletion was identified in the 13th intron of *DENND1A* and 367 kb upstream of *LHX2* with Integrative Genomics Viewer (IGV) (Fig. 5a). We tried to genotype of the deletion variant of everyone in different populations, but it is not obvious to distinguish heterozygous type from homozygous wide type due to limited read counts with IGV viewer, but it is quite confident to identify homozygous wide type. We then compared the frequencies of the homozygous deletion variant in different goat populations. The analysis results showed that the 582

bp deletion near *LHX2*(named 582del) had a higher frequency (52.9–100 %) in cashmere goats as compared 0.00%-40.00% in ordinary goats (Fig. 5b and Supplementary Table 6). We also identified a 504 bp deletion (chr6: 95,454,685-95,455,188 bp) in the *FGF5* locus (named 504del), which is consistent with previous reports (Fig. 5a) (Cai et al. 2020). However, the frequency of the 582del in the ordinary goats was much higher than that of the 504del (Fig. 5b, Supplementary Table 7). More interestingly, we found that the 582del has a high frequency in the IRWG population (80.9 %), while the 504del was absent, which is also consistent with previous research(Cai et al. 2020). Since we could not accurately distinguish the heterozygous from wild genotypes using IGV viewer, we designed PCR primers for detecting the heterozygosity of the two deletions in several goat populations. It showed that the frequency of homozygosity was consistent with that by IGV viewer (Fig. 5c, Fig. 5d, Supplementary Table 8, and Supplementary Table 9), but some breeds had a higher frequency of heterozygosity, such as in UC (504del+/-: 47.5 %) and JNG goats (504del+/-: 66.7 %; 582del+/-: 66.7 %). Finally, we detected the 582del in several ancient goat samples (7/21) and ibex samples (1/3) (Supplementary Fig. 13 and Supplementary Fig. 14), indicating that the 582del is an older mutation that occurred much earlier than the 504del. In addition, we compared the genotypes of the target genomic region for cashmere and ordinary goats. The result showed that the genotype patterns of cashmere goats were highly like that of wild goats, but different from that of ordinary goats (Supplementary Fig. 15).

To further evaluate whether these two deletion variants were related to cashmere traits, we selected 235 CDMC goatswith cashmere yield (Supplementary Fig. 22, Supplementary Table s8) and 581 CDMC goats with fiber diameter records (Supplementary Fig. 23, Supplementary Table s9) for association analysis. The association results showed that the 582del and the 504del was significantly associated with cashmere production (*P* value = 0.0061 and *P* value = 0.0113 for the 582del and the 504del, respectively). The 582del was also significantly associated with fiber diameter (*P* value = 2.74e-04), but the 504del was not (Fig. 5e, Supplementary Table s8, and Supplementary Table s9). Interestingly, the interactions effect of the 582del and

504del were significantly associated with fiber diameter (*P* value = 6.1e-06) (Supplementary Table s9), suggesting crosstalk between the two genes.

Biological Function of the 582 bp Deletion

Analysis of the 582del deletion region using the BLAST program revealed that it is not a highly conserved element but was found in the genomes of primate and ungulate species (Fig. 6a). By checking the UCSC Genome Browser (https://genome.ucsc.edu/), we found many cis-regulatory elements and H3K27Ac marks (often enriched in enhancer regions) upstream of this deleted region (Fig. 6b). Interestingly, we identified a CTCF-binding site and two distal enhancer-like signatures, which were close to the 582bp deletion sequence (Supplementary sequence 1). Using previously reported RNA-seq data(Wu et al. 2020), we found that the expression of *LHX2* in the different fetal stages of cashmere goats exhibited an up-regulation pattern during hair follicle development (Fig. 6c, Supplementary Fig. 16). Furthermore, some functional enhancers of *LHX2* were also identified in the genome regulatory blocks of the *CRB2-LHX2* loci(LeeBrenner and Venkatesh 2011). This evidence suggests that the deletion sequence may function as an insulator to block the *LHX2* enhancer function.

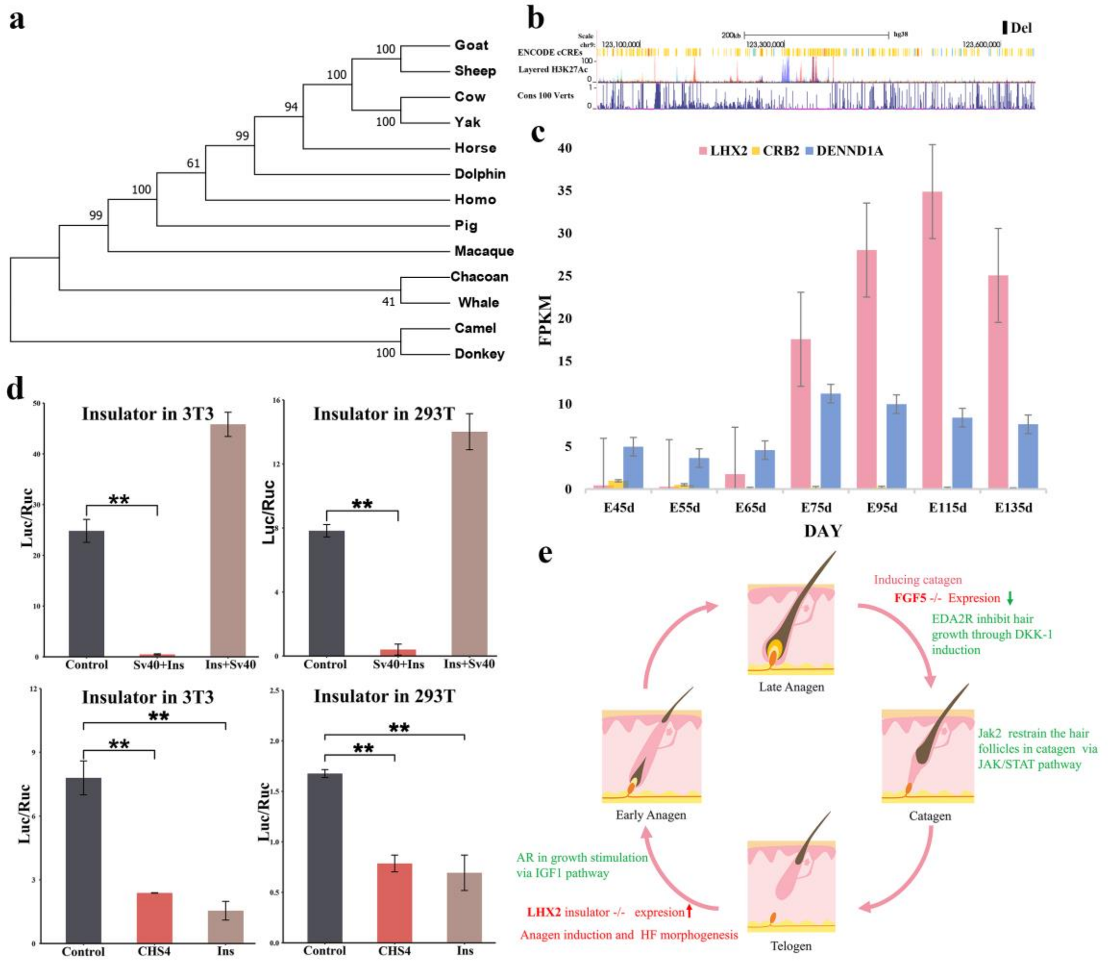

Fig. 6 Effects of *LHX2* deletion sequence on reporter gene expression in mouse NIH3T3 cell line and human 293T cell line. (a) Phylogenetic tree of 13 primates and ungulates for the detection of the 582 bp deleted sequence. **(b)** Regulatory elements, epigenomic signals, and conservation scores on selective sweep region in chromosome 11. The black box represents the deletion variant location of *LHX2*. **(c)** The expression of *LHX2*, *CRB2* and *DENND1A* in different stages of prenatal skin in cashmere goats. Boxes of different colors are used to represent genes. The expression data is downloaded from a previous report(Wu et al. 2020). **(d)** A dual-luciferase assay using NIH3T3 cell and 293T cell shows the *LHX2* upstream deletion sequence blocks the activity of luciferase. Data are shown as the mean ± standard error. The P-value was calculated using Student's *t*-test. **(e)** The molecular mechanisms of cashmere fiber development. In detail, the deletion of the *LHX2* insulator increases the expression of *LHX2* and promotes the cashmere fiber growth at the anagen stage, and *AR* could participate in hair growth at the anagen stage through the *IGF1* pathway(Grymowicz et al. 2020), while the deletion of the 504 bp enhancer reduces the expression of *FGF5*, thereby inhibiting growth regression; *JAK2* and *EDA2R* may also participate in this stage

via the JAK/STAT and Wnt pathways, respectively.

To confirm the insulator function of this sequence, we synthesized a 551 bp DNA fragment (named Ins, Supplementary sequence 2) and subsequently inserted it downstream and upstream of the SV40 promoter in the pGL3 plasmid (Supplementary Fig. 17). The Ins vectors with the Renilla luciferase vector, phRL-TK, were transiently co-transfected into human 293T cells and mouse 3T3 cells. After 48 h, the firefly and Renilla luciferase activities of the lysate were measured, and the ratio of firefly luciferase activity to Renilla luciferase activity was calculated for each sample. Our data showed that the Ins fragment decreased the expression of firefly luciferase by more than 90 % in the downstream group of both cell types and increased expression in the upstream group (Fig. 6d). In addition, we inserted the Ins downstream of the SV40 enhancer in the pGL3 plasmid (Supplementary Fig. 17) and chose the well-known insulator cSH4 as a control to quantify the efficiency of the Ins. Our data showed that the Ins fragment decreased the expression of firefly luciferase by approximately 50 % in human 293T cells, which was comparable to that of cSH4 (Fig. 6d). In addition, a similar result of the Ins activity was validated in the mouse 3T3 cells but decreased more sharply than the cSH4 group (Fig. 6d). These results suggested that the 551 bp DNA fragment played an enhancer-blocking function in mammary cells.

To describe the function mode of these selective sweep genes on the development cycle of cashmere, we downloaded and analyzed the transcriptome data of skin tissue during the prenatal stages and one-year cashmere cycle. The transcriptome data showed that *AR* and *EDA2R* were not expressed in embryonic cashmere goat skin, and *JAK2* gene expression was opposite to that of *LHX2* (data from Wu(Wu et al. 2020), Supplementary Fig. 18). Furthermore, *JAK2*, *EDA2R* and *FGF5* exhibited similar expression patterns in the skin transcriptome data of different months of the one-year cycle, while *LHX2* and *AR* had similar expression patterns (PRJNA470971) (Supplementary Fig. 18 and 19). It is known that cashmere fiber growth has three different periods in one year: a growth period (March–September), a regression period (September–December), and a resting period (December–

March)(Yang et al. 2020). Therefore, the deletion of the *LHX2* insulator increases the expression of *LHX2* and promotes cashmere fiber growth at the anagen stage, while deletion of the *FGF5* enhancer reduces the expression of *FGF5*, inhibiting the regression. The other candidate genes may affect cashmere fiber growth through uninvestigated regulation modes. Overall, we propose a possible molecular model for cashmere fiber formation: *LHX2* and *AR* may be involved in maintaining hair growth at the anagen stage, whereas *JAK2*, *EDA2R* and *FGF5* function in the destructive phase (catagen) through the highest expression in September (Fig. 6e).

Discussion

In this study, we resequenced the genomes of 42 cashmere goats and 78 ordinary goats. Population analyses revealed that goats in southwest China are different in the genome from goats in West Asia and North China, which is consistent with a recent study(Cai et al. 2020). In the present study, we observed a genetic introgression from the LNC breed into the CDMC breed, which was confirmed by the origin and breed practice of CDMC (using Chaidamu goats as female parents, and LNC as male parents to breed new cashmere goats, named CDMC)(Zheng-luZhaeng-kui and DARIQIBU 2003). In addition, the introgression from the Yunnan-Kweichow Plateau into the cashmere goat breeds suggests that goats from the Qinghai-Tibet Plateau and Mongolian Plateau, as well as from the Yunnan-Kweichow Plateau, may have a common ancestor or have a genetic admixture in their early years. Similar introgression results have also been reported in sheep populations from these regions(Yang et al. 2016). In contrast, Angora goats have a signature of genetic admixture with some Chinese goats (Fig. 2a), which has also been reported previously(Ryder 1993). In addition, although KO has large genome differences with other goat breeds(Kim et al. 2019), a clear signature of genetic admixture between KO and JT, JNG, and MT was observed, which may be related to the historical human migration between China and Korea. Finally, we observed CDMC and IMC goats presenting a genetic admixture of UC and LNC. LNC was bred in the 1980s from six counties in the eastern mountainous area of Liaoning province in China, famous for

its high cashmere yield. UC was originally bred from Ujumuqin white goat in the region of Ujumuqin grassland for selecting the cashmere trait in 1994. Unlike LNC, UC has a closer genetic distance to goats in the middle region of China (Fig. 2a). These results indicate that UC may have a more unique genetic background than other cashmere goats.

By performing a whole-genome selection scan, we discovered a novel selective sweep region on chromosome 11 that appears to have undergone extremely strong selection in UC. This novel selective sweep region is located in a conserved linkage block, containing three genes: *DENND1A*, *CRB2* and *LHX2*(LeeBrenner and Venkatesh 2011). Among these genes, *LHX2* functions as a transcriptional activator in hair follicle stem cells and is an essential positive regulator of hair formation(RheePolak and Fuchs 2006; Tornqvist et al. 2010). Furthermore, the expression of *LHX2* was upregulated during hair follicle differentiation, and the expression abundance was constant throughout the development cycle in secondary hair follicles of cashmere goats(Wu et al. 2020; Yang et al. 2020). In contrast, *DENND1A* and *CRB2*, which function in endocytic trafficking to mediate the recycling of selective cargos and early embryonic development, respectively(Xiao et al. 2011; Shi et al. 2019), were expressed lowly in the fetal stage of goat skin and showed very low expression during the cycle of adult cashmere fiber growth (Fig. 6c). These results indicate that *LHX2* is a functional gene in this selective sweep region.

We then reported an upstream deletion of *LHX2*, which carries a potential cis-regulatory insulator region (Fig. 6d). In contrast to the deletion allele of *FGF5*(Cai et al. 2020), the deletion allele of *LHX2* was found in all goats investigated, including wild and domesticated goats, but allele frequency was higher in northern and wild goats than in goats in other areas (Fig. 5b and Fig. 5d). Notably, this deletion was also found in the ancient goat and ibex samples (Supplementary Figs. 13 and 14). Ibex goats may also have dense underwool, like cashmere goats(Ryder 1993). These results indicate that the deletion allele of *LHX2* occurred in the pre-domestication stage of goats and might be traced back to the early stage of goat speciation to cold climate adaptation. Further, we did not detect the 582 bp deletion in the same region

of sheep, but we cannot rule out the possibility that we tested too few sheep (Supplementary Fig. 20). In contrast, the deletion allele of *FGF5* is most likely to occur during the domestication stage of goats to obtain higher cashmere production. Overall, we described a possible model for the origin and diffusion of cashmere-related mutations during the selection evolution of cashmere goats, helping us understand the molecular basis of cashmere trait formation (Fig. 7).

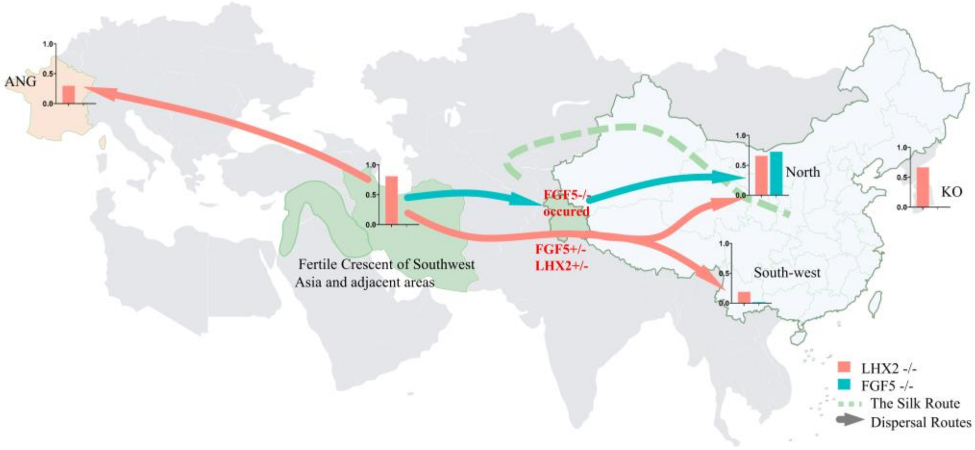

Fig. 7 Origin and diffusion of causative mutations for *LHX2* and *FGF5* in wild and domesticated goats.

Some wild goats acquired the deletion allele of *LHX2* at a very early stage. Because this deletion is beneficial to the development of cashmere to adapt to the cold environment, goats obtained higher genotype frequencies in the north than in the south. The *FGF5* enhancer deletion may have occurred during artificial selection or secondary domestication in the Kashmir region of Afghanistan and gradually migrated north of the Mongolian Plateau. This enhancer deletion was fixed during the long-term artificial selection. The low frequency of the *FGF5* enhancer deletion genotypes in the southwestern region may be related to the genetic admixture between the Qinghai-Tibet Plateau and the Yunnan-Kweichow Plateau goats.

Multiple sequence alignment revealed that the selective sweep region contains many CTCF binding sites and other cis-regulatory elements, as well as the H3K27ac epigenetic mark, located upstream of the 582bp deletion. CTCF is a highly conserved zinc finger protein, required for insulator function in mammals(WestGaszner and Felsenfeld 2002), while H3K27ac is a marker for active enhancers and a great indicator of enhancer activity(Creyghton et al. 2010), suggesting that the selective

sweep region contains some hair-related transcription regulatory elements. Our biological function experiments showed that the 582bp deletion region contains an insulator for blocking the upstream enhancer role with higher efficiency in skin-related cells (NIH3T3 cells) than cSH4. An insulator is a long-range regulatory element, which protect an expressing gene from its surroundings through two ways: blocking the action of a distal enhancer on a promoter and acting as “barriers”that prevent the advance of nearby condensed chromatin (WestGaszner and Felsenfeld 2002) . Previous report described that four of the eight conserved noncoding elements (CNEs), approximately 222 kb to 619 kb upstream of human *LHX2*, functioned as tissue-specific enhancers in specific regions of the central nervous system and the dorsal root ganglia (DRG), recapitulating partial and overlapping expression patterns of *LHX2* and *CRB2* genes (LeeBrenner and Venkatesh 2011). In our research, we found that the insulator is situated approximately 367 kb upstream of goat *LHX2* in the 13th intron of *DENND1A*. Therefore, we speculate that this insulator can regulate the expression of the *LHX2* gene by blocking the upstream enhancers, and the deletion of the insulator can increase the expression of the *LHX2* gene, thus increasing cashmere production. Subsequently, association analysis confirmed the significant relationship between the deletion genotype and cashmere traits, including cashmere production and fiber diameter (Fig. 5e). The interaction effect of the two deletion variants significantly affected the diameter of the cashmere fiber, indicating that the two genes have a synergistic effect on cashmere fiber development.

In addition to *LHX2* and *FGF5*, we also identified a few positively selected genes related to cashmere traits, four genes (*AR*, *JAK2*, *EDA2R* and *STK3*) affecting I-kappaB kinase/NF-kappaB signaling, and five genes (*NOTCH1*, *TCF7L1*, *AR*, *VCAN* and *WNT8B*) influencing the canonical WNT signaling pathway (Supplementary Table s7). We found that *NOTCH1*, *TCF7L1* and *STK3* exhibited similar seasonal expression patterns with *JAK2*, *FGF5* and *EDA2R* in the skin, but *VCAN* and *WNT8B* genes showed low expression (Supplementary Fig. 21; PRJNA470971). NF-kappaB and WNT signaling pathways play a central role in hair follicle development and

regeneration(KishimotoBurgeson and Morgan 2000; Krieger et al. 2018; Choi 2020). Interestingly, *LHX2* is also regulated by NF-κB signaling to promote primary HF morphogenesis(Tomann et al. 2016). *EDA2R* is a divergent gene between cashmere goats (excluding UC goats) and ordinary goats, consistent with previous research(Cai et al. 2020). *EDA2R* is highly expressed in the late anagen phase and may inhibit hair growth by inducing *DKK-1* expression(Kwack et al. 2020). Furthermore, the *AR* gene and *JAK2* are novel genes identified in cashmere traits. *JAK2* can restrain the hair follicles in catagen via the JAK/STAT pathway with up-regulation in catagen and telogen stages(Harel et al. 2015; Tao et al. 2020). In contrast, AR has paradoxically different effects on hair follicles(Randall 2008). In cashmere goats, *AR* is expressed in the early anagen stage and may stimulate cashmere growth via the *IGF-1* pathway(Inui and Itami 2013). These positively selected genes may be involved in cashmere trait formation through the model described in Fig. 6e.

Conclusions

Through population genomics and selective sweep analyses of cashmere and ordinary goats, we identified a novel causative mutation upstream of *LHX2* that functions as an insulator to block the enhancer of *LHX2*. In contrast to enhancer deletion of *FGF5*, this insulator deletion also retained high allele frequency in wild goats, ancient goats, and ibex. We also found that the insulator deletion was associated with two cashmere traits in the CDMC goat population. The positively selected genes were enriched in the NF-κB and Wnt pathways, which play a central role in hair follicle development. This study not only provides the first evidence of how cashmere fiber growth could be regulated during the evolution of goats but also offers valuable molecular markers for the genetic improvement of cashmere traits in goats.

MATERIALS AND METHODS

Samples Collection

We collected 120 domestic goats (C. hircus), representing eight geographically diverse breeds in China, which include 30 CDMC from Chaidamu City of Qinghai Province;

11 UC from Ujimqin Banner of Inner Mongolia Province; 8 GZB from Guiyang City of Guizhou Province; 11 YNBB from Lanping County of Yunnan Province; 10 MT from Shangqiu City of Henan Province; 10 JNG from Jining City of Shandong Province; 10 JTB from Jintang County of Sichuan Province; 12 CDB from Chengdu City of Sichuan Province.. Details of the samples used in this study are givened in Supplementary Table 1. All animal procedures were approved by the life ethics and biological safety review committee of BGI (NO. FT 18041), and were carried out in accordance with the approved guidelines.

DNA Extraction and Sequencing

The ear or blood tissues of CDMC, UC, JT, GZB, JNG, YNBB, CDB and MT were collected on-site and stored in an alcohol sampling tube or blood collection tube. A tissue DNA extraction kit was used to extract the genomic DNA from the samples, electrophoresis was used for integrity detection, and Qubit was used for concentration determination. Library construction for resequencing was performed with 1-3 μg of genomic DNA using standard library preparation protocols and insert sizes from 200–400 base pairs (bp). All 120 goats were sequenced on the BGISEQ-500 platforms with PE100 (CDMC, UC, JT, YNBB, CDB, MT) and on the Illumina HiSeq2500 platforms with PE150 (GZB, JNG). Besides, we downloaded published genomic data of modern, wild and ancient goats form the NCBI deposit (Supplementary Table 2, Supplementary Table 1).

Read Alignment and Variant Calling

We obtained raw data from a total of 236 goat samples, including 120 sequenced and 116 downloaded. All raw data were first filtered and trimmed using SoapNuke (version 1.5.0) (https://github.com/BGI-flexlab/SOAPnuke) (Chen et al. 2018), if any of the following criteria were met: (1) reads containing adapter and poly-N; (2) reads whose low-quality base ratio (base quality less than or equal to 5) is more than 50%; (3) reads whose unknown base ('N' base) ratio is more than 10%. Clean reads from all individuals were aligned to the goat reference genome ARS1 (https://www.ncbi.nlm.nih.gov/assembly/GCF_001704415.1/)(Hassanin et al. 2010) by the Burrows-Wheeler Aligner (BWA version 0.7.12). SAMtools(Li et al. 2009) was

used to convert the file format from SAM to BAM and filter the unmapped and non-unique reads. Picard (version 1.54, http://broadinstitute.github.io/picard/) was used to sort the BAM files and remove potential PCR duplications if multiple read pairs had identical external coordinates.

GATK (version 4.0.11, https://github.com/broadinstitute/gatk) tools were used in the whole process of variant calling. After mapping, the “HaplotypeCaller”, “CombineGVCFs” and “GenotypeGVCFs” in GATK4 were used to detect SNPs and Indels with default parameters. The output VCF File was then screened for SNPs using "SelectVariants function" of GATK4. SNPs and Indels were separated using the GATK tool “SelectVariants” and subjected to rigorous processing to exclude false positives. To obtain high-quality SNPs, we carried out SNP filtering at two stages. SNPs exclusion criteria(Choi et al. 2015) were as follows: (1) hard filtration with parameter “QD < 2.0 || ReadPosRankSum < -8.0 || FS > 60.0 || MQ < 40.0 || SOR > 3.0 || MQRankSum < -12.5 || QUAL < 30;” (2) “--max-missing 0.9 --maf 0.05 --min-alleles 2 --max-alleles 2.” Finally, ~13 million high-quality SNPs were remained for further analysis.

Population Structure Analysis

We used 12,861,877 high-quality SNPs (autosome) to PCA analysis. PLINK (version 1.9)(Purcell et al. 2007) was used to calculate the principal components, using function "--vcf vcf --out pca –pca --chr-set 29 --allow-extra-chr".

An individual-based NJ tree was constructed for the 236 goats based on the p-distance, with one outgroup (IRW, Iranian wild goat) using the software VCF2Dis (version 1.09, https://github.com/BGI-shenzhen/VCF2Dis) and PHYLIP (version 3.69) (http://evolution.genetics.washington.edu/phylip.html). The Linux command line of converting to a matrix is "VCF2Dis -InPut vcf -OutPut p_dis.mat"; the Linux command line of constructing a phylogenetic tree is " fneighbor -datafile p_dis.mat -outfile goat.tree.txt -matrixtype s -treetype n -outtreefile goat.n.tree.tree".

Population structure was analyzed using the ADMIXTURE (version 1.23)(AlexanderNovembre and Lange 2009; Alexander and Lange 2011) program which implement a block-relaxation algorithm. To explore the convergence of

individuals, we predefined the number of genetic clusters K from 3–7 and ran with cross-validation error (CV) procedure. Default methods and settings were used in Admixture analysis. The analysis process includes the following three steps:

(1) Converting VCF format to PLINK format. The Linux command line was "vcftools --vcf vcf --plink --out goat.plink";

(2) Using PLINK for further filtering with Linux command line "plink --noweb --file plink --geno 0.05 --maf 0.05 --hwe 0.0001 --chr-set 29 --make-bed --out QC"; after this step we get the corresponding bed file.

(3) ADMIXTURE v1.23 for population structure analysis with "admixture --cv QC.bed $k | tee log${k}.out"; then the results were plotted using R.

PopLDdecay (https://github.com/BGI-shenzhen/PopLDdecay)(Zhang et al. 2019) for linkage disequilibrium decay analysis was based on variant call format files.

Demographic History

A population-level admixture analysis was conducted in the TreeMix (version 1.12)(Pickrell and Pritchard 2012). The program inferred the ML tree for 12 goat breeds (215 individuals) and an outgroup (Bezoars,21 individuals), then the residuals matrix was used to identify pairs of populations that showed poor fits in the ML tree. These populations were regarded as candidates around which we added potential migration edges, and new arrangements of the ML tree accounting for migration events were generated (Pickrell and Pritchard 2012). From one to 20 migration events were gradually added to the ML tree until 98% of the variance between the breeds could be explained. The command was '-i input -bootstrap -k 500 -root IRW -o output'.

We used the PSMC (version 0.6.5) (Li and Durbin 2011) method to estimate changes in the effective population size (Ne) of goat over the last one million years. The PSMC analysis was implemented in the seven samples sequenced at a high read depth (12.6~ 26.94×) (Supplementary Table 11).The parameters of PSMC were set to -N25 -t15 -r5 -p "4+25*2+4+6", where the parameters of generation time and mutation rate were set to 2.0 and 2.5e-8, respectively.

Selective Sweep Analysis

In the selection sweep, we calculated the genome-wide distribution of Fst values and θ_π Ratios (Danecek et al. 2011) (i.e., $\theta_{\pi\text{-UC}}/\theta_{\pi\text{-Ordinary}}$, $\theta_{\pi\text{-UC}}/\theta_{\pi\text{-CDMC.IMC.LNC}}$, $\theta_{\pi\text{-LNC.IMC}}/\theta_{\pi\text{-Ordinary}}$, $\theta_{\pi\text{-White}}/\theta_{\pi\text{-Brown}}$ and $\theta_{\pi\text{-White}}/\theta_{\pi\text{-Black}}$) for five control group pairs, which included the UC group versus the ordinary group (GZB, YNBB, JTB, CDB), the UC group versus other cashmere (CDMC, IMC, LNC), the other cashmere group (CDMC, IMC, LNC) versus the ordinary group (GZB, YNBB, JTB, CDB), the white coat color(UC,CDMC, IMC, LNC) versus the brown coat color(CDB), the white coat color(IMC, LNC) versus the black coat color(YNBB, GZB) using a sliding-window approach (150-kb windows with 10-kb increments). The Fst values were Z-transformed, and the $\theta\pi$ Ratios were $\log_2$-transformed. We considered the windows with the top 1% values for the ZFst and $\log_2(\theta_\pi$ Ratios) simultaneously as the candidate outliers under strong selective sweeps. All of the outlier windows were assigned to corresponding SNPs and candidate genes. The signals were further confirmed by ZFst, $\mathrm{Log}_2(\theta_\pi$ Ratios), and Tajima's D with a 10 kb sliding window.

Functional Enrichment Analyses

Candidate genes under selection were defined as those overlapping sweep regions or within 500 kb of the signals. The biological function of genes within candidate regions was annotated by analyzing Gene Ontology (GO) and Kyoto Encyclopedia of Genes and Genomes (KEGG) pathways using Metascape(http://metascape.org/). Benjamini–Hochberg FDR (false discovery rate) was used for correcting the P values. The Gene Ontology categories "Molecular Function," "Biological Process" and "Cellular Component", and Human Phenotype (HP) categories were used in these analyses.

Genotypic and phenotypic analysis

Genotyping. Genotypic and phenotypic analysis DNA was extracted from the ear-tissue samples. The 582 bp deletion near *LHX2* (582del) and the 504 bp deletion of *FGF5* (504del) were genotyped by PCR amplification using the reaction condition of the 2-min pre-denaturation at 95 ℃, 15s-denaturation at 95 ℃, 30s-annealing at 56 ℃, 45s-extension at 72 ℃ for 30 cycle, and 5-min extension at 72 ℃ (the primers of 582 bp

deletion: 5’-CAGTACGGAGCAAGTAAACGG-3’ and 5’-ACCATTCCACTTGTCCACCT-3’, the primers of 504 bp deletion: 5’-ACAGCGTGTGATCTTTTCTCTG-3’ and 5’-TCTTGGTCTGGCTGTGATCA-3’) and visualized on 2% agarose gels. The 582del and 540del were successfully genotyped in the extended population of 840 goats, including 752 CDMC, 12 IMC, 40 UC, 12 JNG, 12 Wushan white goats (from Chongqing in the southwest of China) and 12 JTB individuals.

***Phenotypic analysis*.** All 581 cashmere samples were collected from the lateral body part of CDMC goats, and the fiber diameter was measured by an optical-based fiber diameter analyzer (OFDA2000). Sample preparation is the most critical part of obtaining accurate results from OFDA. Briefly, the cashmere fibers are roughly aligned in parallel and cut into 2 mm by the OFDA bench guillotine, and then washed the fibers to remove grease. After drying, use a spreader to evenly spread 10-15 mg of fibers on the glass slide. Place the slide on the stage, then enlarge and scan the snippets through the optical transmission microscope. The camera system collects the fiber image, and then the fiber in it is automatically identified through image analysis technology and the diameter is measured. In addition, we measured cashmere yield from a subset of 351 CDMC goats in May 2020. The cashmere production is measured by hand-combing cashmere and weighing. First, use a thin comb to comb the feces and other dirt along the direction of the hair, then use a density-comb to comb the direction of the hair repeatedly and finally comb the hair in the reverse direction until the fallen fibers are combed. The collected cashmere is weighed with an electronic scale (range 0.3g-5kg, accuracy 0.1g). Then, we tested if the identified deletion variants were associated with cashmere diameter and production, respectively. Totally, 581 individuals were recorded, the linear model was expressed as, $y=b0+bx+sex+batch+e$, where, y was the diameter; x was the deletion coding with 0, 1 and 2 representing 0, 1 and 2 copies of deletion sequence, respectively; b was the effect of deletion variant; sex was the sex effect; batch was the recorder effects, where 2 recorders were participated in the phenotyping; e was the residual error, assumed to follow normal distribution. When performed the association between the deletion variants and the production, 235 individuals were

included, all were females, the linear model for the association was expressed as y=b0+bx +e. The association studies were performed with lm function of R package.

RNAseq

RNAseq data were downloaded from the NCBI database project id SRP145408. Raw data were filtered by SOAPnuke (version 1.5.6), read base N more than 5%, and read base quality less than 20 was removed. Clean reads from the 12 individual samples were aligned to the reference genome (GCA_001704415.1 ARS1) using hisat2 (version 2.1.0). Picard-tools-1.105 was used to sort BAM files. Based on the mapped reads and goat reference genome annotation in GFF (GCF_001704415.1), StringTie (version,1.3.3b) was used to calculate the FPKM(Pertea et al. 2016).

Dual-Luciferase Reporter Analysis

Plasmid Constructs, a 563 bp DNA fragment containing “5’ end - KpnI/HindIII site-Ins sequence (551bp)-XhoI/NcoI site was synthesized by TsingKe Ltd, which was subsequently cloned downstream and upstream of the SV40 promoter in the pGL3 plasmid, named as Insulator1 and Insulator2, respectively. A 796 bp DNA fragment containing “5’ end - BamHI site-Ins sequence(551bp)-SalI site-sv40 enhancer sequence- PciI site – 3’ end” (Supplementary Fig. 17) was synthesized by TsingKe Ltd, which was subsequently cloned downstream of Luc+ Poly (A) in the pGL3 promoter plasmid (Promega) and named as Ens-E. Similarly, the well-known insulator cHS4 linked to the sv40 enhancer (Supplementary Fig. 17) was cloned into a pGL3 promoter plasmid and named cHS4-E.

Cell Culture, Transfection, and Luciferase Reporter Assay. The 3T3 cells and 293T cells were maintained in Dulbecco’s modified Eagle’s medium (Gibco, Waltham, MA, USA) with penicillin (100 units/ml), streptomycin (100 μg/ml), and 10 % fetal bovine serum at 37 °C in a humidified atmosphere of 95 % air and 5 % CO_2. Before transfection, 3T3 and 293T cells were seeded into 24-well plates at 1.0 × 104 cells/well. After overnight attachment, transfections were performed using Lipofectamine 2000 (Invitrogen, CA, USA) according to the manufacturer’s instructions. In this experiment, the two types of cells were divided into eight groups, respectively. For each group,

plasmid of pGL3-control (Promega), Insulator1 and Insulator2 or Ins-E and cHS4-E were co-transfected with phRL-TK (Promega) at a ratio of 10:1 (0.8 ug: 0.08 ug) into the 3T3 and 293T cells, respectively. The cells were harvested two days after co-transfection, and luciferase activity was evaluated using the Dual-Luciferase Reporter Assay System (Promega, #E1910). Firefly luciferase data were normalized to Renilla luciferase activity. For this assay, pGL3-control was used as a positive control and Lipofectamine 2000 was used as a blank control. The cHS4 insulator was chosen as a reference to quantify the efficiency of the insulator function.

Data availability.

The data that support the findings of this study have been deposited into CNGB Sequence Archive (CNSA)(Guo et al. 2020) of China National GeneBank DataBase (CNGBdb)(Chen et al. 2020) with accession number CNP0001896 （https://db.cngb.org/cnsa/project/CNP0001896/reviewlink/）.

Acknowledgements

This work was supported by the Science and Technology Innovation Strategy Projects of Guangdong Province (2019B020203002), the Basic Research Project of Haixi Agriculture and Animal Husbandry Bureau (Qinghai Caidamu Cashmere Goat Genomic Breeding project), the National Natural Science Foundation of China (31872560 and 31672399), the Shenzhen Municipal Government of China (JCYJ20180307163440037).

We thank Bayaertu & Yong quan from Dongwuzhumuqin Banner Aogali Animal Husbandry Co., Ltd and Hu Yeyong from Henan yudong animal husbandry co. Ltd for providing UC and MT goat materials, respectively. We thank Shancen Zhao for helpful discussion and revising the manuscript.

Author contributions

L.Y, F.M, Z.S.Y. conceived, designed, and supervised the study. H. H., F. M., L.Y., C.T. and M.Z.P performed the informatics analysis of the sequencing data. Z.S.Y., Y.M.M., W.Q., Y.C.Y., W.Q.J., WL.B., G.G., Mengkedala., D.W.D., L.B., Z.Q.F. obtained goat material and DNA for re-sequencing. Z.S.Y., Y.M.M., W.Q.J., WL.B., G.G., Mengkedala. and L.B. performed phenotypic data collation and analysis. Y.M.M., W.R., J.D., W.Q. and Z.T.T. participated in the laboratory work. Z.X.J., Z.T.T. and L.L. performed dual-Luciferase Reporter analysis. C.T analyzed the transcriptome. L.Y., H.H., Y.M.M., J.D. and Z.X.J are the major contributors in writing the manuscript. All authors read, revised, and approved the final manuscript.

Additional information

Supplementary Information accompanies this paper at Supplementary.docx and Supplementary table s.xlsx.

Competing interests: The authors declare no competing interests.

References:

Alexander, D. H., and K. Lange. 2011. Enhancements to the ADMIXTURE algorithm for individual ancestry estimation. BMC BIOINFORMATICS **12**:246.

Cai, Y., W. Fu, D. Cai, R. Heller, Z. Zheng, J. Wen, H. Li, X. Wang, A. Alshawi, Z. Sun, S. Zhu, J. Wang, M. Yang, S. Hu, Y. Li, Z. Yang, M. Gong, Y. Hou, T. Lan, K. Wu, Y. Chen, Y. Jiang, and X. Wang. 2020. Ancient Genomes Reveal the Evolutionary History and Origin of Cashmere-Producing Goats in China. MOLECULAR BIOLOGY AND EVOLUTION **37**:2099-2109.

Chen, F. Z., L. J. You, F. Yang, L. N. Wang, X. Q. Guo, F. Gao, C. Hua, C. Tan, L. Fang, R. Q. Shan, W. J. Zeng, B. Wang, R. Wang, X. Xu, and X. F. Wei. 2020. CNGBdb: China National GeneBank DataBase. Yi Chuan **42**:799-809.

Chen, Y., Y. Chen, C. Shi, Z. Huang, Y. Zhang, S. Li, Y. Li, J. Ye, C. Yu, Z. Li, X. Zhang, J. Wang, H. Yang, L. Fang, and Q. Chen. 2018. SOAPnuke: a MapReduce acceleration-supported software for integrated quality control and preprocessing of high-throughput sequencing data. GigaScience **7**:1-6.

Choi, B. Y. 2020. Targeting Wnt/beta-Catenin Pathway for Developing Therapies for Hair Loss. INTERNATIONAL JOURNAL OF MOLECULAR SCIENCES **21**.

Choi, J. W., B. H. Choi, S. H. Lee, S. S. Lee, H. C. Kim, D. Yu, W. H. Chung, K. T. Lee, H. H. Chai, Y. M. Cho, and D. Lim. 2015. Whole-Genome Resequencing Analysis of Hanwoo and Yanbian Cattle to Identify Genome-Wide SNPs and Signatures of Selection. MOLECULES AND CELLS **38**:466-473.

Creyghton, M. P., A. W. Cheng, G. G. Welstead, T. Kooistra, B. W. Carey, E. J. Steine, J. Hanna, M. A. Lodato, G. M. Frampton, P. A. Sharp, L. A. Boyer, R. A. Young, and R. Jaenisch. 2010. Histone H3K27ac separates active from poised enhancers and predicts developmental state. Proc Natl Acad Sci U S A **107**:21931-21936.

Dai, B., F. Hao, T. Xu, B. Zhu, L. Q. Ren, X. Y. Han, and D. J. Liu. 2020. Thymosin beta4 Identified by Transcriptomic Analysis from HF Anagen to Telogen Promotes Proliferation of SHF-DPCs in Albas Cashmere Goat. INTERNATIONAL JOURNAL OF MOLECULAR SCIENCES **21**.

Daly, K. G., D. P. Maisano, V. E. Mullin, A. Scheu, V. Mattiangeli, M. D. Teasdale, A. J. Hare, J. Burger, M. P. Verdugo, M. J. Collins, R. Kehati, C. M. Erek, G. Bar-Oz, F. Pompanon, T. Cumer, C. Cakirlar, A. F. Mohaseb, D. Decruyenaere, H. Davoudi, O. Cevik, G. Rollefson, J. D. Vigne, R. Khazaeli, H. Fathi, S. B. Doost, S. R. Rahimi, A. A. Vahdati, E. W. Sauer, K. H. Azizi, S. Maziar, B. Gasparian, R. Pinhasi, L. Martin, D. Orton, B. S. Arbuckle, N. Benecke, A. Manica, L. K. Horwitz, M. Mashkour, and D. G. Bradley. 2018. Ancient goat genomes reveal mosaic domestication in the Fertile Crescent. SCIENCE **361**:85-88.

Danecek, P., A. Auton, G. Abecasis, C. A. Albers, E. Banks, M. A. DePristo, R. E. Handsaker, G. Lunter, G. T. Marth, S. T. Sherry, G. McVean, and R. Durbin. 2011. The variant call format and VCFtools. BIOINFORMATICS **27**:2156-2158.

Gao, Y., X. Wang, H. Yan, J. Zeng, S. Ma, Y. Niu, G. Zhou, Y. Jiang, and Y. Chen. 2016. Comparative Transcriptome Analysis of Fetal Skin Reveals Key Genes Related to Hair Follicle Morphogenesis in Cashmere Goats. PLoS One **11**:e151118.

Geng, R.C. Yuan, and Y. Chen. 2013. Exploring differentially expressed genes by RNA-Seq in cashmere goat (Capra hircus) skin during hair follicle development and cycling. PLoS One **8**:e62704.

Geng, R., L. Wang, X. Wang, and Y. Chen. 2014. Cyclic expression of Lhx2 is involved in secondary hair follicle development in cashmere goat. GENE EXPRESSION PATTERNS **16**:31-35.

Grymowicz, M., E. Rudnicka, A. Podfigurna, P. Napierala, R. Smolarczyk, K. Smolarczyk, and B. Meczekalski. 2020. Hormonal effects on hair follicles. INTERNATIONAL JOURNAL OF MOLECULAR SCIENCES **21**:5342.

Guo, J., J. Zhong, G. E. Liu, L. Yang, L. Li, G. Chen, T. Song, and H. Zhang. 2020. Identification and population genetic analyses of copy number variations in six domestic goat breeds and Bezoar ibexes using next-generation sequencing. BMC GENOMICS **21**:840.

Guo, X., F. Chen, F. Gao, L. Li, K. Liu, L. You, C. Hua, F. Yang, W. Liu, C. Peng, L. Wang, X. Yang, F. Zhou, J. Tong, J. Cai, Z. Li, B. Wan, L. Zhang, T. Yang, M. Zhang, L. Yang, Y. Yang, W. Zeng, B. Wang, X. Wei, and X. Xu. 2020. CNSA: a data repository for archiving omics data. Database (Oxford) **2020**.

Harel, S., C. A. Higgins, J. E. Cerise, Z. Dai, J. C. Chen, R. Clynes, and A. M. Christiano. 2015. Pharmacologic inhibition of JAK-STAT signaling promotes hair growth. Science Advances **1**:e1500973.

Hassanin, A., C. Bonillo, B. X. Nguyen, and C. Cruaud. 2010. Comparisons between mitochondrial genomes of domestic goat (Capra hircus) reveal the presence of numts and multiple sequencing errors. Mitochondrial DNA **21**:68-76.

Huelsken, J., R. Vogel, B. Erdmann, G. Cotsarelis, and W. Birchmeier. 2001. beta-Catenin controls hair follicle morphogenesis and stem cell differentiation in the skin. CELL **105**:533-545.

Inui, S., and S. Itami. 2013. Androgen actions on the human hair follicle: perspectives. EXPERIMENTAL DERMATOLOGY **22**:168-171.

Jin, M., J. Lu, X. Fei, Z. Lu, K. Quan, Y. Liu, M. Chu, Di R, H. Wang, and C. Wei. 2020. Genetic Signatures of Selection for Cashmere Traits in Chinese Goats. Animals (Basel) **10**.

Kim, J. Y., S. Jeong, K. H. Kim, W. J. Lim, H. Y. Lee, and N. Kim. 2019. Discovery of Genomic Characteristics and Selection Signatures in Korean Indigenous Goats Through Comparison of 10 Goat Breeds. Frontiers in Genetics **10**:699.

Kishimoto, J.R. E. Burgeson, and B. A. Morgan. 2000. Wnt signaling maintains the hair-inducing activity of the dermal papilla. GENES & DEVELOPMENT **14**:1181-1185.

Krieger, K., S. E. Millar, N. Mikuda, I. Krahn, J. E. Kloepper, M. Bertolini, C. Scheidereit, R. Paus, and R. Schmidt-Ullrich. 2018. NF-kappaB Participates in Mouse Hair Cycle Control and Plays Distinct Roles in the Various Pelage Hair Follicle Types. JOURNAL OF INVESTIGATIVE DERMATOLOGY **138**:256-264.

Kwack, M. H., M. S. Jun, Y. K. Sung, J. C. Kim, and M. K. Kim. 2020. Ectodysplasin-A2 induces dickkopf 1 expression in human balding dermal papilla cells overexpressing the ectodysplasin A2 receptor. Biochem Biophys Res Commun **529**:766-772.

Lee, A. P.S. Brenner, and B. Venkatesh. 2011. Mouse transgenesis identifies conserved functional

enhancers and cis-regulatory motif in the vertebrate LIM homeobox gene Lhx2 locus. PLoS One **6**:e20088.

Li, G., S. Zhou, C. Li, B. Cai, H. Yu, B. Ma, Y. Huang, Y. Ding, Y. Liu, Q. Ding, C. He, J. Zhou, Y. Wang, G. Zhou, Y. Li, Y. Yan, J. Hua, B. Petersen, Y. Jiang, T. Sonstegard, X. Huang, Y. Chen, and X. Wang. 2019. Base pair editing in goat: nonsense codon introgression into FGF5 results in longer hair. FEBS Journal **286**:4675-4692.

Li, H., B. Handsaker, A. Wysoker, T. Fennell, J. Ruan, N. Homer, G. Marth, G. Abecasis, and R. Durbin. 2009. The Sequence Alignment/Map format and SAMtools. BIOINFORMATICS **25**:2078-2079.

Li, H., and R. Durbin. 2011. Inference of human population history from individual whole-genome sequences. NATURE **475**:493-496.

Li, X., R. Su, W. Wan, W. Zhang, H. Jiang, X. Qiao, Y. Fan, Y. Zhang, R. Wang, Z. Liu, Z. Wang, B. Liu, Y. Ma, H. Zhang, Q. Zhao, T. Zhong, Di R, Y. Jiang, W. Chen, W. Wang, Y. Dong, and J. Li. 2017. Identification of selection signals by large-scale whole-genome resequencing of cashmere goats. Sci Rep **7**:15142.

Li, Y., S. Song, X. Liu, Y. Zhang, D. Wang, X. He, Q. Zhao, Y. Pu, W. Guan, Y. Ma, and L. Jiang. 2020. Deletion of an enhancer in FGF5 is associated with ectopic expression in goat hair follicle s and the cashmere growth phenotype. bioRxiv.

McAllister, J. M., B. Modi, B. A. Miller, J. Biegler, R. Bruggeman, R. S. Legro, and J. R. Strauss. 2014. Overexpression of a DENND1A isoform produces a polycystic ovary syndrome theca phenotype. Proc Natl Acad Sci U S A **111**:E1519-E1527.

Millar, P. 1986. The performance of cashmere goats. Pp. 181-199. Animal Breeding Abstracts.

Paniagua, A. E., A. Segurado, J. F. Dolon, J. Esteve-Rudd, A. Velasco, D. S. Williams, and C. Lillo. 2021. Key Role for CRB2 in the Maintenance of Apicobasal Polarity in Retinal Pigment Epithelial Cells. Front Cell Dev Biol **9**:701853.

Pertea, M., D. Kim, G. M. Pertea, J. T. Leek, and S. L. Salzberg. 2016. Transcript-level expression analysis of RNA-seq experiments with HISAT, StringTie and Ballgown. Nature Protocols **11**:1650-1667.

Pickrell, J. K., and J. K. Pritchard. 2012. Inference of population splits and mixtures from genome-wide allele frequency data. PLoS Genetics **8**:e1002967.

Purcell, S., B. Neale, K. Todd-Brown, L. Thomas, M. A. Ferreira, D. Bender, J. Maller, P. Sklar, P. I. de Bakker, M. J. Daly, and P. C. Sham. 2007. PLINK: a tool set for whole-genome association and population-based linkage analyses. AMERICAN JOURNAL OF HUMAN GENETICS **81**:559-575.

Randall, V. A. 2008. The endocrine control of the hair follicle. Pp. 23-39. Hair Growth and Disorders. Springer.

Rhee, H.L. Polak, and E. Fuchs. 2006. Lhx2 maintains stem cell character in hair follicles. SCIENCE **312**:1946-1949.

Rishikaysh, P., K. Dev, D. Diaz, W. M. Qureshi, S. Filip, and J. Mokry. 2014. Signaling involved in hair follicle morphogenesis and development. INTERNATIONAL JOURNAL OF MOLECULAR SCIENCES **15**:1647-1670.

Ryder, M. L. 1993. The use of goat hair: an introductory historical review. Anthropozoologica.

Shi, J., Q. Gao, Y. Cao, and J. Fu. 2019. Dennd1a, a susceptibility gene for polycystic ovary syndrome, is essential for mouse embryogenesis. Dev Dyn **248**:351-362.

Su, R., Y. Fan, X. Qiao, X. Li, L. Zhang, C. Li, and J. Li. 2018. Transcriptomic analysis reveals critical genes for the hair follicle of Inner Mongolia cashmere goat from catagen to telogen. PLoS One **13**:e204404.

Tao, Y., X. Zhou, Z. Liu, X. Zhang, Y. Nie, X. Zheng, S. Li, X. Hu, G. Yang, Q. Zhao, and C. Mou. 2020. Expression patterns of three JAK-STAT pathway genes in feather follicle development during chicken embryogenesis. GENE EXPRESSION PATTERNS **35**:119078.

Tomann, P., R. Paus, S. E. Millar, C. Scheidereit, and R. Schmidt-Ullrich. 2016. Lhx2 is a direct NF-kappaB target gene that promotes primary hair follicle placode down-growth. DEVELOPMENT **143**:1512-1522.

Tornqvist, G., A. Sandberg, A. C. Hagglund, and L. Carlsson. 2010. Cyclic expression of lhx2 regulates hair formation. PLoS Genetics **6**:e1000904.

Waldron, S.C. Brown, and A. M. Komarek. 2014. The Chinese Cashmere Industry: A Global Value Chain Analysis. Development Policy Review **32**:589-610.

Wang, X., B. Cai, J. Zhou, H. Zhu, Y. Niu, B. Ma, H. Yu, A. Lei, H. Yan, Q. Shen, L. Shi, X. Zhao, J. Hua, X. Huang, L. Qu, and Y. Chen. 2016. Disruption of FGF5 in Cashmere Goats Using CRISPR/Cas9 Results in More Secondary Hair Follicles and Longer Fibers. PLoS One **11**:e164640.

Wang, X., J. Liu, G. Zhou, J. Guo, H. Yan, Y. Niu, Y. Li, C. Yuan, R. Geng, X. Lan, X. An, X. Tian, H. Zhou, J. Song, Y. Jiang, and Y. Chen. 2016. Whole-genome sequencing of eight goat populations for the detection of selection signatures underlying production and adaptive traits. Sci Rep **6**:38932.

West, A. G.M. Gaszner, and G. Felsenfeld. 2002. Insulators: many functions, many mechanisms. Genes Dev **16**:271-288.

Wu, Z., E. Hai, Di Z, R. Ma, F. Shang, Y. Wang, M. Wang, L. Liang, Y. Rong, J. Pan, W. Wu, R. Su, Z. Wang, R. Wang, Y. Zhang, and J. Li. 2020. Using WGCNA (weighted gene co-expression network analysis) to identify the hub genes of skin hair follicle development in fetus stage of Inner Mongolia cashmere goat. PLoS One **15**:e243507.

Xiao, Z., J. Patrakka, M. Nukui, L. Chi, D. Niu, C. Betsholtz, T. Pikkarainen, S. Vainio, and K. Tryggvason. 2011. Deficiency in Crumbs homolog 2 (Crb2) affects gastrulation and results in embryonic lethality in mice. Dev Dyn **240**:2646-2656.

Yang, F., Z. Liu, M. Zhao, Q. Mu, T. Che, Y. Xie, L. Ma, L. Mi, J. Li, and Y. Zhao. 2020. Skin transcriptome reveals the periodic changes in genes underlying cashmere (ground hair) follicle transition in cashmere goats. BMC GENOMICS **21**:392.

Yang, J., W. R. Li, F. H. Lv, S. G. He, S. L. Tian, W. F. Peng, Y. W. Sun, Y. X. Zhao, X. L. Tu, M. Zhang, X. L. Xie, Y. T. Wang, J. Q. Li, Y. G. Liu, Z. Q. Shen, F. Wang, G. J. Liu, H. F. Lu, J. Kantanen, J. L. Han, M. H. Li, and M. J. Liu. 2016. Whole-Genome Sequencing of Native Sheep Provides Insights into Rapid Adaptations to Extreme Environments. MOLECULAR BIOLOGY AND EVOLUTION **33**:2576-2592.

Zeder, M. A. 2008. Domestication and early agriculture in the Mediterranean Basin: Origins, diffusion, and impact. Proc Natl Acad Sci U S A **105**:11597-11604.

Zhang, B., L. Chang, X. Lan, N. Asif, F. Guan, D. Fu, B. Li, C. Yan, H. Zhang, X. Zhang, Y. Huang, H. Chen, J. Yu, and S. Li. 2018. Genome-wide definition of selective sweeps reveals molecular evidence of trait-driven domestication among elite goat (Capra species) breeds for the production of dairy, cashmere, and meat. GigaScience **7**.

Zhang, C., S. S. Dong, J. Y. Xu, W. M. He, and T. L. Yang. 2019. PopLDdecay: a fast and effective tool for linkage disequilibrium decay analysis based on variant call format files. BIOINFORMATICS **35**:1786-1788.

Zhang, Y., K. Wu, L. Wang, Z. Wang, W. Han, D. Chen, Y. Wei, R. Su, R. Wang, Z. Liu, Y. Zhao, Z. Wang, L. Zhan, Y. Zhang, and J. Li. 2020. Comparative study on seasonal hair follicle cycling by analysis

of the transcriptomes from cashmere and milk goats. GENOMICS **112**:332-345.
Zhang, Y., L. Wang, Z. Li, D. Chen, W. Han, Z. Wu, F. Shang, E. Hai, Y. Wei, R. Su, Z. Liu, R. Wang, Z. Wang, Y. Zhao, Z. Wang, Y. Zhang, and J. Li. 2019. Transcriptome profiling reveals transcriptional and alternative splicing regulation in the early embryonic development of hair follicles in the cashmere goat. Sci Rep **9**:17735.
Zheng-lu, H.Y. Zhaeng-kui, and DARIQIBU. 2003. The Breeding of Chai Da-mu Cashmere Coat in Wulan County of Qinghai Province. ECOLOGY OF DOMESTIC ANIMAL:76-78.

Manuscript

Click here to access/download;Manuscript;Supplementary table s.pdf

Supplementary Table s1:The candidate genes in the selection sweep of UC goats and ordinary goats

Gene	Chr	Bin_start	Bin_end	Gene_Start	Gene_end	ZFst	log 2 (θπ ratio)
CBLB	chr1	50270001	50420001	50156574	50379147	4.045866	1.026640173
ALCAM	chr1	50270001	50420001	49834499	50068116	4.045866	1.026640173
BCHE	chr1	100750001	100900001	101031428	101110937	4.739633	1.231168682
WARS2	chr3	96550001	96700001	96453280	96556143	6.977931	2.118194997
HAO2	chr3	96650001	96800001	96792486	96828594	4.421209	1.249012327
TBX15	chr3	96550001	96700001	96299835	96424984	6.977931	2.118194997
WARS2	chr3	96560001	96710001	96453280	96556143	6.901183	2.098734355
HAO2	chr3	96550001	96700001	96792486	96828594	6.977931	2.118194997
3BHSD	chr3	96550001	96700001	96839059	96848990	6.977931	2.118194997
ZNF697	chr3	96550001	96700001	96860774	96894430	6.977931	2.118194997
PHGDH	chr3	96550001	96700001	96957379	96988400	6.977931	2.118194997
HMGCS2	chr3	96550001	96700001	96993773	97024594	6.977931	2.118194997
REG4	chr3	96550001	96700001	97053866	97080492	6.977931	2.118194997
NOTCH2	chr3	96600001	96750001	97171972	97351176	6.674734	1.652744876
PIK3CG	chr4	72590001	72740001	72257537	72291605	4.318878	1.01187
CCDC71L	chr4	72590001	72740001	72481791	72486442	4.318878	1.01187
NAMPT	chr4	72590001	72740001	72928492	72971902	4.318878	1.01187
SYPL1	chr4	72590001	72740001	73117696	73148219	4.318878	1.01187
CYSTM1	chr7	59130001	59280001	59064021	59134162	5.469975	1.253373931
IGIP	chr7	59140001	59290001	59183771	59183983	5.847594	1.193806765
PURA	chr7	59140001	59290001	59193011	59204429	5.847594	1.193806765
NRG2	chr7	59140001	59290001	59276390	59474026	5.847594	1.193806765
HARS	chr7	59120001	59270001	58682131	58695144	4.910462	1.15632183
DND1	chr7	59120001	59270001	58695237	58698549	4.910462	1.15632183
WDR55	chr7	59120001	59270001	58698546	58703385	4.910462	1.15632183
IK	chr7	59140001	59290001	58705364	58718109	5.847594	1.193806765
NDUFA2	chr7	59140001	59290001	58718219	58720374	5.847594	1.193806765
TMCO6	chr7	59140001	59290001	58720332	58725924	5.847594	1.193806765
CD14	chr7	59140001	59290001	58731680	58735123	5.847594	1.193806765
SLC35A4	chr7	59140001	59290001	58754826	58759575	5.847594	1.193806765
APBB3	chr7	59140001	59290001	58759676	58766097	5.847594	1.193806765
SRA1	chr7	59140001	59290001	58766299	58773099	5.847594	1.193806765
EIF4EBP3	chr7	59140001	59290001	58773687	58774464	5.847594	1.193806765
SLC4A9	chr7	59140001	59290001	58927092	58941009	5.847594	1.193806765
HBEGF	chr7	59140001	59290001	58936015	58967277	5.847594	1.193806765
PFDN1	chr7	59140001	59290001	58990749	59062745	5.847594	1.193806765
CYSTM1	chr7	59140001	59290001	59064021	59134162	5.847594	1.193806765
NRG2	chr7	59120001	59270001	59276390	59474026	4.910462	1.15632183
PSD2	chr7	59140001	59290001	59477094	59549870	5.847594	1.193806765
CXXC5	chr7	59140001	59290001	59617412	59651710	5.847594	1.193806765
UBE2D2	chr7	59140001	59290001	59671314	59714771	5.847594	1.193806765
KIAA2026	chr8	38560001	38710001	38513727	38622147	4.466058	1.379238809
MLANA	chr8	38560001	38710001	38632729	38645056	4.466058	1.379238809
ERMP1	chr8	38570001	38720001	38711983	38774165	4.407573	1.360757448
RCL1	chr8	39570001	39720001	39520371	39583801	6.049661	1.584187032
AK3	chr8	39590001	39740001	39635494	39656272	6.08271	1.760158455
CDC37L1	chr8	39590001	39740001	39660101	39678648	6.08271	1.760158455
PLPP6	chr8	39590001	39740001	39700133	39703413	6.08271	1.760158455
SPATA6L	chr8	39590001	39740001	39734093	39756809	6.08271	1.760158455
SLC1A1	chr8	39630001	39780001	39770036	39845247	4.571571	1.466562188
GLDC	chr8	38560001	38710001	38074650	38160199	4.466058	1.379238809
UHRF2	chr8	38560001	38710001	38178911	38250733	4.466058	1.379238809

TPD52L3	chr8	38560001	38710001	38297532	38298263	4.466058	1.379238809
IL33	chr8	38560001	38710001	38337407	38408297	4.466058	1.379238809
RANBP6	chr8	38560001	38710001	38506959	38511581	4.466058	1.379238809
ERMP1	chr8	38560001	38710001	38711983	38774165	4.466058	1.379238809
RIC1	chr8	38560001	38710001	38776219	38919131	4.466058	1.379238809
PDCD1LG2	chr8	38560001	38710001	38979921	39046980	4.466058	1.379238809
CD274	chr8	38560001	38710001	39077983	39095141	4.466058	1.379238809
PLGRKT	chr8	39570001	39720001	39111116	39155487	6.049661	1.584187032
INSL6	chr8	39590001	39740001	39210866	39227365	6.08271	1.760158455
JAK2	chr8	39590001	39740001	39245187	39360411	6.08271	1.760158455
RCL1	chr8	39590001	39740001	39520371	39583801	6.08271	1.760158455
CDC37L1	chr8	39510001	39660001	39660101	39678648	4.064055	0.981399549
PLPP6	chr8	39550001	39700001	39700133	39703413	5.256231	1.300527496
SPATA6L	chr8	39570001	39720001	39734093	39756809	6.049661	1.584187032
SLC1A1	chr8	39590001	39740001	39770036	39845247	6.08271	1.760158455
GLIS3	chr8	39590001	39740001	40049592	40544666	6.08271	1.760158455
NCOA1	chr11	74200001	74350001	74153980	74372125	5.76972	0.93259616
RNAC-AC	chr11	74190001	74340001	74190725	74190796	5.679361	0.895461035
CRB2	chr11	94010001	94160001	94006350	94030821	6.979376	0.85927
DENND1A	chr11	94370001	94520001	94030061	94571614	12.1586	3.09584
RNAC-GC	chr11	94370001	94520001	93961937	93962008	12.1586	3.09584
DNMT3A	chr11	74200001	74350001	73679835	73784188	5.76972	0.93259616
POMC	chr11	74200001	74350001	73833491	73836904	5.76972	0.93259616
EFR3B	chr11	74200001	74350001	73838760	73935019	5.76972	0.93259616
DNAJC27	chr11	74200001	74350001	73977548	74015566	5.76972	0.93259616
ADCY3	chr11	74200001	74350001	74037527	74125235	5.76972	0.93259616
CENPO	chr11	74200001	74350001	74124434	74138988	5.76972	0.93259616
PTRHD1	chr11	74200001	74350001	74138657	74142150	5.76972	0.93259616
RNAC-AC	chr11	74200001	74350001	74190725	74190796	5.76972	0.93259616
ITSN2	chr11	74200001	74350001	74499640	74622540	5.76972	0.93259616
FAM228A	chr11	74200001	74350001	74624305	74636502	5.76972	0.93259616
FAM228B	chr11	74200001	74350001	74637060	74685661	5.76972	0.93259616
PFN4	chr11	74200001	74350001	74669150	74674491	5.76972	0.93259616
TP53I3	chr11	74200001	74350001	74678510	74685183	5.76972	0.93259616
SF3B6	chr11	74200001	74350001	74685654	74693957	5.76972	0.93259616
FKBP1B	chr11	74200001	74350001	74698136	74708853	5.76972	0.93259616
WDCP	chr11	74200001	74350001	74711378	74726672	5.76972	0.93259616
MFSD2B	chr11	74200001	74350001	74729851	74742391	5.76972	0.93259616
UBXN2A	chr11	74200001	74350001	74745868	74769527	5.76972	0.93259616
ATAD2B	chr11	74210001	74360001	74782987	74910341	5.494236	0.859224999
RC3H2	chr11	94000001	94150001	93525713	93577732	6.821547	0.851769
ZBTB6	chr11	94010001	94160001	93579682	93587389	6.979376	0.85927
ZBTB26	chr11	94010001	94160001	93589694	93604487	6.979376	0.85927
RABGAP1	chr11	94190001	94340001	93604558	93766020	9.854278	1.6232
GPR21	chr11	94120001	94270001	93690990	93697311	7.600753	1.10254
STRBP	chr11	94340001	94490001	93779449	93924823	11.85756	3.23961
RNAC-GC	chr11	94370001	94520001	93961937	93962008	12.1586	3.09584
CRB2	chr11	94370001	94520001	94006350	94030821	12.1586	3.09584
LHX2	chr11	94370001	94520001	94640045	94659024	12.1586	3.09584
NEK6	chr11	94370001	94520001	94837125	94922060	12.1586	3.09584
PSMB7	chr11	94370001	94520001	94922900	94979454	12.1586	3.09584
RNAC-GC	chr11	94370001	94520001	93961937	93962008	12.1586	3.09584
MYCBP2	chr12	33710001	33860001	33616605	33885679	7.85467	2.367480164
FBXL3	chr12	33750001	33900001	33896229	33919253	7.12068	1.664834787
CLN5	chr12	33780001	33930001	33920524	33931904	6.754983	1.452723271
ACOD1	chr12	33810001	33960001	33959349	33966931	5.673926	1.15025561

SLAIN1	chr12	33640001	33790001	33165618	33217921	5.722692	2.793518952
SCEL	chr12	33710001	33860001	33247740	33372142	7.85467	2.367480164
FBXL3	chr12	33710001	33860001	33896229	33919253	7.85467	2.367480164
CLN5	chr12	33710001	33860001	33920524	33931904	7.85467	2.367480164
ACOD1	chr12	33710001	33860001	33959349	33966931	7.85467	2.367480164
KCTD12	chr12	33710001	33860001	34023639	34029762	7.85467	2.367480164
RNF24	chr13	50590001	50740001	50520829	50637289	6.667194	1.797467437
RNAE-UU	chr13	50450001	50600001	50530729	50530800	5.724039	1.019706585
PANK2	chr13	50590001	50740001	50640389	50669701	6.667194	1.797467437
MIR103	chr13	50590001	50740001	50646831	50646916	6.667194	1.797467437
MAVS	chr13	50590001	50740001	50694748	50707107	6.667194	1.797467437
AP5S1	chr13	50590001	50740001	50734433	50738294	6.667194	1.797467437
CDC25B	chr13	50610001	50760001	50751016	50761010	5.106432	1.334935462
CENPB	chr13	50630001	50780001	50772192	50775019	4.681956	1.153658808
SPEF1	chr13	50630001	50780001	50775106	50781210	4.681956	1.153658808
:13H20orf2	chr13	50660001	50810001	50785603	50799453	4.643325	1.074449067
HSPA12B	chr13	50660001	50810001	50799840	50816831	4.643325	1.074449067
BPIFB4	chr13	61720001	61870001	61704744	61730572	4.250796	0.93728656
HAO1	chr13	50450001	50600001	50024601	50086221	5.724039	1.019706585
ADRA1D	chr13	50590001	50740001	50264597	50293882	6.667194	1.797467437
SMOX	chr13	50590001	50740001	50305402	50340974	6.667194	1.797467437
RNF24	chr13	50660001	50810001	50520829	50637289	4.643325	1.074449067
RNAE-UU	chr13	50590001	50740001	50530729	50530800	6.667194	1.797467437
PANK2	chr13	50450001	50600001	50640389	50669701	5.724039	1.019706585
MIR103	chr13	50450001	50600001	50646831	50646916	5.724039	1.019706585
MAVS	chr13	50450001	50600001	50694748	50707107	5.724039	1.019706585
AP5S1	chr13	50580001	50730001	50734433	50738294	5.896336	1.920141848
CDC25B	chr13	50590001	50740001	50751016	50761010	6.667194	1.797467437
CENPB	chr13	50590001	50740001	50772192	50775019	6.667194	1.797467437
SPEF1	chr13	50590001	50740001	50775106	50781210	6.667194	1.797467437
:13H20orf2	chr13	50590001	50740001	50785603	50799453	6.667194	1.797467437
HSPA12B	chr13	50590001	50740001	50799840	50816831	6.667194	1.797467437
SIGLEC1	chr13	50590001	50740001	50842091	50860436	6.667194	1.797467437
ADAM33	chr13	50590001	50740001	50865505	50879346	6.667194	1.797467437
GFRA4	chr13	50590001	50740001	50882036	50886744	6.667194	1.797467437
ATRN	chr13	50590001	50740001	50894660	51079673	6.667194	1.797467437
13H20orf1!	chr13	50590001	50740001	51139649	51312804	6.667194	1.797467437
RNAS-GG	chr13	50680001	50830001	51246448	51246520	4.139824	1.161308758
NOL4L	chr13	61720001	61870001	61202695	61335042	4.250796	0.93728656
RNAR-CC	chr13	61720001	61870001	61427319	61427391	4.250796	0.93728656
COMMD7	chr13	61720001	61870001	61430474	61455479	4.250796	0.93728656
DNMT3B	chr13	61720001	61870001	61467790	61502090	4.250796	0.93728656
MAPRE1	chr13	61720001	61870001	61508458	61541861	4.250796	0.93728656
SUN5	chr13	61720001	61870001	61602832	61626958	4.250796	0.93728656
BPIFB2	chr13	61720001	61870001	61632481	61650156	4.250796	0.93728656
BPIFB6	chr13	61720001	61870001	61657835	61672584	4.250796	0.93728656
BPIFB3	chr13	61720001	61870001	61681607	61696489	4.250796	0.93728656
BPIFA2	chr13	61720001	61870001	61962034	61971556	4.250796	0.93728656
BPIFA3	chr13	61730001	61880001	62295723	62306454	4.02234	1.045072322
RNAE-UU	chr13	50590001	50740001	50530729	50530800	6.667194	1.797467437
NMNAT2	chr16	63130001	63280001	62912702	63133011	5.708738	1.539642138
SMG7	chr16	63180001	63330001	63132818	63225482	7.661123	1.737211663
NCF2	chr16	63180001	63330001	63224955	63263716	7.661123	1.737211663
ARPC5	chr16	63180001	63330001	63300052	63310232	7.661123	1.737211663
APOBEC4	chr16	63180001	63330001	63322257	63323411	7.661123	1.737211663
LAMC1	chr16	63180001	63330001	62678983	62800726	7.661123	1.737211663

LAMC2	chr16	63180001	63330001	62840547	62910467	7.661123	1.737211663
NMNAT2	chr16	63180001	63330001	62912702	63133011	7.661123	1.737211663
SMG7	chr16	63230001	63380001	63132818	63225482	5.912934	1.123261708
ARPC5	chr16	63150001	63300001	63300052	63310232	6.389922	1.490857194
APOBEC4	chr16	63170001	63320001	63322257	63323411	7.484859	1.564476752
RGL1	chr16	63180001	63330001	63416372	63587678	7.661123	1.737211663
COLGALT	chr16	63180001	63330001	63596114	63715051	7.661123	1.737211663
TSEN15	chr16	63180001	63330001	63723676	63754234	7.661123	1.737211663
LDLRAD4	chr24	43800001	43950001	43647271	43808160	4.197746	1.20640782
FAM210A	chr24	43800001	43950001	43802788	43825139	4.197746	1.20640782
RNMT	chr24	43800001	43950001	43825214	43852257	4.197746	1.20640782
MC5R	chr24	43800001	43950001	43868653	43873520	4.197746	1.20640782
MC2R	chr24	43800001	43950001	43894659	43910455	4.197746	1.20640782
SPIRE1	chr24	43800001	43950001	43245485	43386532	4.197746	1.20640782
CEP76	chr24	43800001	43950001	43395941	43413346	4.197746	1.20640782
PSMG2	chr24	43800001	43950001	43413556	43422907	4.197746	1.20640782
PTPN2	chr24	43800001	43950001	43436686	43502642	4.197746	1.20640782
SEH1L	chr24	43800001	43950001	43534245	43554790	4.197746	1.20640782
CEP192	chr24	43800001	43950001	43556637	43630586	4.197746	1.20640782
CPXM2	chr26	8820001	8970001	8404960	8540659	5.681956	0.886241492
GPR26	chr26	8820001	8970001	8587125	8615287	5.681956	0.886241492
RNAG-GC	chr26	8820001	8970001	9008923	9008995	5.681956	0.886241492
BUB3	chr26	8820001	8970001	9070381	9082657	5.681956	0.886241492
HMX2	chr26	8820001	8970001	9085905	9088891	5.681956	0.886241492
HMX3	chr26	8820001	8970001	9099008	9101028	5.681956	0.886241492
ACADSB	chr26	8820001	8970001	9179479	9214221	5.681956	0.886241492
IKZF5	chr26	8820001	8970001	9214257	9232272	5.681956	0.886241492
PSTK	chr26	8820001	8970001	9240462	9253956	5.681956	0.886241492
RNAR-UC	chr26	8820001	8970001	9364714	9364785	5.681956	0.886241492
HELLS	chr26	35320001	35470001	35557819	35594336	5.244505	0.858559442
TBC1D12	chr26	35320001	35470001	35600390	35696027	5.244505	0.858559442
NOC3L	chr26	35320001	35470001	35718792	35745826	5.244505	0.858559442
PLCE1	chr26	35320001	35470001	35749803	36126653	5.244505	0.858559442
RNAH-AU	chr26	35320001	35470001	35860914	35860985	5.244505	0.858559442
IPMK	chr26	51130001	51280001	50971027	51025641	6.955776	0.875704506
CISD1	chr26	51130001	51280001	51026850	51045714	6.955776	0.875704506
UBE2D1	chr26	51130001	51280001	51089559	51128547	6.955776	0.875704506

Supplementary Table s2:The candidate genes in the selection sweep of UC goats and other cashmere goats

Gene	Chr	Bin start	Bin end	Gene Start	Gene end	ZFst	g 2 (θπ ratio)
INSIG2	chr2	66620001	66770001	66374212	66386544	4.717596	1.113071
CCDC93	chr2	66620001	66770001	66492663	66600576	4.717596	1.113071
DDX18	chr2	66620001	66770001	66651336	66667824	4.717596	1.113071
RASSF9	chr5	15610001	15760001	15329017	15361750	5.800002	1.053702
NTS	chr5	15610001	15760001	15391221	15402965	5.800002	1.053702
MGAT4C	chr5	15610001	15760001	15533616	15603737	5.800002	1.053702
PLEKHG7	chr5	22830001	22980001	22348785	22417547	6.329986	1.176884
EEA1	chr5	22830001	22980001	22421664	22546190	6.329986	1.176884
'RNAE-CU	chr5	22830001	22980001	22660968	22661040	6.329986	1.176884
NUDT4	chr5	22830001	22980001	22964342	22980670	6.329986	1.176884
UBE2N	chr5	22830001	22980001	22984934	23027066	6.329986	1.176884
MRPL42	chr5	22830001	22980001	23041060	23070803	6.329986	1.176884
SOCS2	chr5	22830001	22980001	23129217	23135562	6.329986	1.176884
CRADD	chr5	22830001	22980001	23232979	23425649	6.329986	1.176884
ALPK1	chr6	13970001	14120001	13456823	13570751	8.838069	1.599863
TIFA	chr6	13970001	14120001	13580947	13589288	8.838069	1.599863
AP1AR	chr6	13970001	14120001	13595155	13624310	8.838069	1.599863
C6H4orf32	chr6	13970001	14120001	13672177	13715799	8.838069	1.599863
PDLIM5	chr6	30790001	30940001	30440595	30679977	5.065607	1.07677
HPGDS	chr6	30790001	30940001	30819572	30845549	5.065607	1.07677
SMARCAD	chr6	30790001	30940001	30850057	30940436	5.065607	1.07677
ATOH1	chr6	30790001	30940001	31242153	31244357	5.065607	1.07677
'RNAS-GG	chr6	30790001	30940001	31293473	31293544	5.065607	1.07677
GRID2	chr6	30790001	30940001	31299697	32930194	5.065607	1.07677
'RNAE-UU	chr6	30790001	30940001	31327448	31327519	5.065607	1.07677
ANTXR2	chr6	95410001	95560001	95056743	95230556	15.15942	2.245108
PRDM8	chr6	95410001	95560001	95336875	95356821	15.15942	2.245108
FGF5	chr6	95410001	95560001	95418992	95440124	15.15942	2.245108
C6H4orf22	chr6	95410001	95560001	95489192	96199327	15.15942	2.245108
'RNAG-CC	chr6	95410001	95560001	95616042	95616114	15.15942	2.245108
VCAN	chr7	27580001	27730001	27133454	27253501	5.027904	1.273321
XRCC4	chr7	27580001	27730001	27358825	27646398	5.027904	1.273321
TMEM167A	chr7	27580001	27730001	27646443	27691570	5.027904	1.273321
TMCO6	chr7	59150001	59300001	58720332	58725924	4.480137	1.398587
CD14	chr7	59150001	59300001	58731680	58735123	4.480137	1.398587
SLC35A4	chr7	59150001	59300001	58754826	58759575	4.480137	1.398587
APBB3	chr7	59150001	59300001	58759676	58766097	4.480137	1.398587
SRA1	chr7	59150001	59300001	58766299	58773099	4.480137	1.398587
EIF4EBP3	chr7	59150001	59300001	58773687	58774464	4.480137	1.398587
'RNAC-GC	chr11	94180001	94330001	93961937	93962008	8.360215	1.08025
SLC4A9	chr7	59150001	59300001	58927092	58941009	4.480137	1.398587
HBEGF	chr7	59150001	59300001	58936015	58967277	4.480137	1.398587
PFDN1	chr7	59150001	59300001	58990749	59062745	4.480137	1.398587
CYSTM1	chr7	59150001	59300001	59064021	59134162	4.480137	1.398587
IGIP	chr7	59150001	59300001	59183771	59183983	4.480137	1.398587
PURA	chr7	59150001	59300001	59193011	59204429	4.480137	1.398587
NRG2	chr7	59150001	59300001	59276390	59474026	4.480137	1.398587
PSD2	chr7	59150001	59300001	59477094	59549870	4.480137	1.398587
CXXC5	chr7	59150001	59300001	59617412	59651710	4.480137	1.398587
UBE2D2	chr7	59150001	59300001	59671314	59714771	4.480137	1.398587
DTWD2	chr7	75830001	75980001	75660851	75734513	7.784391	1.432332
DMXL1	chr7	75830001	75980001	75824075	75958422	7.784391	1.432332
TNFAIP8	chr7	75830001	75980001	75975505	76118465	7.784391	1.432332

HSD17B4	chr7	75830001	75980001	76164451	76264055	7.784391	1.432332
FAM170A	chr7	75830001	75980001	76361601	76365214	7.784391	1.432332
RNAW-CC	chrX2	33520001	33670001	33182054	33182125	9.241732	1.111889
PRR16	chr7	76720001	76870001	77276296	77554872	5.303108	1.117342
TLE6	chr7	90200001	90350001	89777457	89789932	4.951197	1.235683
TLE2	chr7	90200001	90350001	89790761	89811070	4.951197	1.235683
AES	chr7	90200001	90350001	89825491	89833731	4.951197	1.235683
GNA11	chr7	90200001	90350001	89858038	89875620	4.951197	1.235683
GNA15	chr7	90200001	90350001	89884034	89904169	4.951197	1.235683
'RNAV-CA	chr7	90200001	90350001	89889797	89889869	4.951197	1.235683
S1PR4	chr7	90200001	90350001	89915136	89919050	4.951197	1.235683
NCLN	chr7	90200001	90350001	89923720	89937645	4.951197	1.235683
CELF5	chr7	90200001	90350001	89948056	89998459	4.951197	1.235683
NFIC	chr7	90200001	90350001	90052700	90125061	4.951197	1.235683
SMIM24	chr7	90200001	90350001	90128780	90131715	4.951197	1.235683
DOHH	chr7	90200001	90350001	90141286	90150323	4.951197	1.235683
FZR1	chr7	90200001	90350001	90164317	90182907	4.951197	1.235683
C7H19orf71	chr7	90200001	90350001	90183012	90188234	4.951197	1.235683
MFSD12	chr7	90200001	90350001	90188702	90197934	4.951197	1.235683
HMG20B	chr7	90200001	90350001	90209112	90215077	4.951197	1.235683
GIPC3	chr7	90200001	90350001	90218057	90221461	4.951197	1.235683
TBXA2R	chr7	90200001	90350001	90224431	90235848	4.951197	1.235683
CACTIN	chr7	90200001	90350001	90239897	90252116	4.951197	1.235683
PIP5K1C	chr7	90200001	90350001	90253792	90301886	4.951197	1.235683
TJP3	chr7	90200001	90350001	90309335	90339632	4.951197	1.235683
APBA3	chr7	90200001	90350001	90339711	90347136	4.951197	1.235683
MRPL54	chr7	90200001	90350001	90348993	90352086	4.951197	1.235683
RAX2	chr7	90200001	90350001	90352183	90354653	4.951197	1.235683
MATK	chr7	90200001	90350001	90358237	90366369	4.951197	1.235683
ZFR2	chr7	90200001	90350001	90382609	90427427	4.951197	1.235683
ATCAY	chr7	90200001	90350001	90440653	90480830	4.951197	1.235683
NMRK2	chr7	90200001	90350001	90489144	90493587	4.951197	1.235683
DAPK3	chr7	90200001	90350001	90504689	90517977	4.951197	1.235683
EEF2	chr7	90200001	90350001	90521523	90530393	4.951197	1.235683
PIAS4	chr7	90200001	90350001	90552914	90577026	4.951197	1.235683
ZBTB7A	chr7	90200001	90350001	90579097	90599740	4.951197	1.235683
MAP2K2	chr7	90200001	90350001	90625518	90646901	4.951197	1.235683
CREB3L3	chr7	90200001	90350001	90675700	90687529	4.951197	1.235683
SIRT6	chr7	90200001	90350001	90687710	90696547	4.951197	1.235683
ANKRD24	chr7	90200001	90350001	90709138	90726622	4.951197	1.235683
EBI3	chr7	90200001	90350001	90726730	90732953	4.951197	1.235683
CCDC94	chr7	90200001	90350001	90735131	90749262	4.951197	1.235683
SHD	chr7	90200001	90350001	90755834	90764186	4.951197	1.235683
TMIGD2	chr7	90200001	90350001	90764906	90772249	4.951197	1.235683
FSD1	chr7	90200001	90350001	90774022	90786104	4.951197	1.235683
STAP2	chr7	90230001	90380001	90786651	90798263	4.463907	1.271817
MPND	chr7	90230001	90380001	90803788	90813427	4.463907	1.271817
SH3GL1	chr7	90240001	90390001	90813591	90840745	4.314688	1.25121
MFSD14B	chr8	300001	450001	194748	313390	7.516694	1.212537
ANXA10	chr8	300001	450001	366581	454508	7.516694	1.212537
DDX60	chr8	300001	450001	469234	525201	7.516694	1.212537
PALLD	chr8	300001	450001	571937	953942	7.516694	1.212537
BCL11A	chr11	43260001	43410001	42802900	42902566	4.657959	1.311925
PAPOLG	chr11	43260001	43410001	43112732	43144983	4.657959	1.311925
REL	chr11	43260001	43410001	43228119	43265438	4.657959	1.311925
PUS10	chr11	43260001	43410001	43279025	43357451	4.657959	1.311925

PEX13	chr11	43260001	43410001	43357512	43391701	4.657959	1.311925
'RNAC-GC.	chr11	94180001	94330001	93961937	93962008	8.360215	1.08025
KIAA1841	chr11	43260001	43410001	43411369	43468576	4.657959	1.311925
GCFC2	chr11	43260001	43410001	43518809	43594785	4.657959	1.311925
MRPL19	chr11	43260001	43410001	43620250	43631033	4.657959	1.311925
EVA1A	chr11	43260001	43410001	43700750	43781436	4.657959	1.311925
PTCD3	chr11	48770001	48920001	48322191	48350270	4.394665	1.127641
POLR1A	chr11	48830001	48980001	48350620	48437096	6.623518	1.305807
ST3GAL5	chr11	48830001	48980001	48575055	48635258	6.623518	1.305807
ATOH8	chr11	48830001	48980001	48683976	48725280	6.623518	1.305807
GNLY	chr11	48830001	48980001	48786756	48789216	6.623518	1.305807
SFTPB	chr11	48830001	48980001	48852391	48866415	6.623518	1.305807
USP39	chr11	48830001	48980001	48878107	48915123	6.623518	1.305807
C11H2orf68	chr11	48830001	48980001	48918344	48924302	6.623518	1.305807
TMEM150A	chr11	48830001	48980001	48926643	48931720	6.623518	1.305807
RNF181	chr11	48830001	48980001	48931843	48944939	6.623518	1.305807
VAMP5	chr11	48830001	48980001	48936693	48945008	6.623518	1.305807
VAMP8	chr11	48830001	48980001	48947446	48950828	6.623518	1.305807
GGCX	chr11	48830001	48980001	48970438	48983617	6.623518	1.305807
MAT2A	chr11	48830001	48980001	48985904	48992675	6.623518	1.305807
SH2D6	chr11	48830001	48980001	49079744	49087050	6.623518	1.305807
CAPG	chr11	48830001	48980001	49108549	49126635	6.623518	1.305807
ELMOD3	chr11	48830001	48980001	49129367	49162874	6.623518	1.305807
'RNAG-CC	chr6	95410001	95560001	95616042	95616114	15.15942	2.245108
RETSAT	chr11	48830001	48980001	49163287	49178905	6.623518	1.305807
TGOLN2	chr11	48830001	48980001	49179390	49187399	6.623518	1.305807
TCF7L1	chr11	48830001	48980001	49197164	49389243	6.623518	1.305807
PDCL	chr11	93730001	93880001	93502797	93511273	5.034236	1.190971
RC3H2	chr11	93730001	93880001	93525713	93577732	5.034236	1.190971
ZBTB6	chr11	93730001	93880001	93579682	93587389	5.034236	1.190971
ZBTB26	chr11	93730001	93880001	93589694	93604487	5.034236	1.190971
RABGAP1	chr11	94180001	94330001	93604558	93766020	8.360215	1.08025
GPR21	chr11	93730001	93880001	93690990	93697311	5.034236	1.190971
STRBP	chr11	94180001	94330001	93779449	93924823	8.360215	1.08025
'RNAC-GC.	chr11	94180001	94330001	93961937	93962008	8.360215	1.08025
CRB2	chr11	94180001	94330001	94006350	94030821	8.360215	1.08025
DENND1A	chr11	94180001	94330001	94030061	94571614	8.360215	1.08025
LHX2	chr11	94180001	94330001	94640045	94659024	8.360215	1.08025
NEK6	chr11	94310001	94460001	94837125	94922060	7.432019	3.3312
PSMB7	chr11	94350001	94500001	94922900	94979454	6.539472	3.39406
PAEP	chr11	103080001	103230001	102722000	102727131	7.864747	1.268077
GLT6D1	chr11	103080001	103230001	102736918	102750984	7.864747	1.268077
LCN9	chr11	103080001	103230001	102752525	102755707	7.864747	1.268077
SOHLH1	chr11	103080001	103230001	102765983	102770191	7.864747	1.268077
KCNT1	chr11	103080001	103230001	102773986	102845015	7.864747	1.268077
CAMSAP1	chr11	103080001	103230001	102853679	102899665	7.864747	1.268077
UBAC1	chr11	103080001	103230001	102921461	102940501	7.864747	1.268077
NACC2	chr11	103080001	103230001	102971729	103052853	7.864747	1.268077
C11H9orf69	chr11	103080001	103230001	103069579	103073716	7.864747	1.268077
CCDC187	chr11	103080001	103230001	103179116	103224725	7.864747	1.268077
GPSM1	chr11	103080001	103230001	103233280	103236379	7.864747	1.268077
DNLZ	chr11	103080001	103230001	103240018	103241932	7.864747	1.268077
CARD9	chr11	103080001	103230001	103242044	103250512	7.864747	1.268077
SNAPC4	chr11	103080001	103230001	103250772	103272873	7.864747	1.268077
SDCCAG3	chr11	103080001	103230001	103275335	103281712	7.864747	1.268077
PMPCA	chr11	103080001	103230001	103281684	103292475	7.864747	1.268077

INPP5E	chr11	103080001	103230001	103293035	103303634	7.864747	1.268077
SEC16A	chr11	103080001	103230001	103304047	103334563	7.864747	1.268077
NOTCH1	chr11	103080001	103230001	103349994	103395722	7.864747	1.268077
EGFL7	chr11	103080001	103230001	103493921	103505661	7.864747	1.268077
MIR126	chr11	103080001	103230001	103503998	103504079	7.864747	1.268077
AGPAT2	chr11	103080001	103230001	103505961	103518463	7.864747	1.268077
FAM69B	chr11	103080001	103230001	103537747	103545952	7.864747	1.268077
ABO	chr11	103080001	103230001	103593328	103629857	7.864747	1.268077
SURF6	chr11	103080001	103230001	103647731	103654912	7.864747	1.268077
MED22	chr11	103090001	103240001	103655791	103662423	7.745723	1.268608
RPL7A	chr11	103090001	103240001	103662472	103665820	7.745723	1.268608
SURF2	chr11	103100001	103250001	103669990	103675502	7.724125	1.264893
SURF4	chr11	103100001	103250001	103673865	103685466	7.724125	1.264893
STKLD1	chr11	103130001	103280001	103693166	103708795	7.556998	1.226179
REXO4	chr11	103140001	103290001	103708896	103717819	7.464061	1.229936
ADAMTS1[illegible]	chr11	103150001	103300001	103722208	103755062	7.183195	1.212109
CACFD1	chr11	103190001	103340001	103755819	103764723	6.049875	1.116282
TNFRSF19	chr12	52760001	52910001	52252542	52348993	4.981813	1.192716
MIPEP	chr12	52760001	52910001	52377621	52457448	4.981813	1.192716
C1QTNF9	chr12	52760001	52910001	52462240	52469963	4.981813	1.192716
SPATA13	chr12	52760001	52910001	52473037	52579821	4.981813	1.192716
AMER2	chr12	52760001	52910001	52766430	52768477	4.981813	1.192716
MTMR6	chr12	52760001	52910001	52804352	52824861	4.981813	1.192716
NUP58	chr12	52760001	52910001	52830226	52862819	4.981813	1.192716
[illegible]RNAG-UC	chr12	52760001	52910001	52877526	52877598	4.981813	1.192716
ATP8A2	chr12	52760001	52910001	52884246	53350170	4.981813	1.192716
MTDH	chr14	16010001	16160001	15663731	15721499	4.696795	1.75102
LAPTM4B	chr14	16010001	16160001	15788488	15854656	4.696795	1.75102
MATN2	chr14	16010001	16160001	15872116	16041603	4.696795	1.75102
RPL30	chr14	16010001	16160001	16048416	16051780	4.696795	1.75102
ERICH5	chr14	16010001	16160001	16063489	16086802	4.696795	1.75102
RIDA	chr14	16010001	16160001	16096003	16104730	4.696795	1.75102
POP1	chr14	16010001	16160001	16104883	16139688	4.696795	1.75102
NIPAL2	chr14	16010001	16160001	16173131	16256193	4.696795	1.75102
KCNS2	chr14	16010001	16160001	16394213	16400041	4.696795	1.75102
STK3	chr14	16010001	16160001	16433649	16744347	4.696795	1.75102
DCLK3	chr22	11010001	11160001	10587039	10629524	4.713277	1.356235
GOLGA4	chr22	11010001	11160001	10653112	10757227	4.713277	1.356235
ITGA9	chr22	11010001	11160001	10780757	11147531	4.713277	1.356235
CTDSPL	chr22	11010001	11160001	11187226	11311455	4.713277	1.356235
MIR26A	chr22	11010001	11160001	11298067	11298156	4.713277	1.356235
VILL	chr22	11010001	11160001	11316379	11337078	4.713277	1.356235
PLCD1	chr22	11010001	11160001	11337411	11359212	4.713277	1.356235
DLEC1	chr22	11010001	11160001	11369750	11460169	4.713277	1.356235
ACAA1	chr22	11010001	11160001	11460323	11484652	4.713277	1.356235
MYD88	chr22	11010001	11160001	11484772	11490413	4.713277	1.356235
OXSR1	chr22	11010001	11160001	11513751	11598867	4.713277	1.356235
BTRC	chr26	29820001	29970001	29360210	29542837	5.365387	1.075789
LBX1	chr26	29820001	29970001	29649006	29652810	5.365387	1.075789
TLX1	chr26	29820001	29970001	29716272	29721774	5.365387	1.075789
KAZALD1	chr26	29820001	29970001	29780565	29784209	5.365387	1.075789
SFXN3	chr26	29820001	29970001	29805091	29812297	5.365387	1.075789
PDZD7	chr26	29820001	29970001	29813473	29833314	5.365387	1.075789
LZTS2	chr26	29820001	29970001	29833983	29844452	5.365387	1.075789
C26H10orf[illegible]	chr26	29820001	29970001	29846333	29851711	5.365387	1.075789
MRPL43	chr26	29820001	29970001	29851736	29853327	5.365387	1.075789

SEMA4G	chr26	29820001	29970001	29853719	29867663	5.365387	1.075789
SLF2	chr26	29820001	29970001	29872724	29914824	5.365387	1.075789
PAX2	chr26	29820001	29970001	29995533	30085588	5.365387	1.075789
HIF1AN	chr26	29820001	29970001	30240811	30255353	5.365387	1.075789
NDUFB8	chr26	29820001	29970001	30260956	30266363	5.365387	1.075789
SEC31B	chr26	29820001	29970001	30269322	30300136	5.365387	1.075789
WNT8B	chr26	29820001	29970001	30304225	30335373	5.365387	1.075789
SCD	chr26	29830001	29980001	30404780	30415917	5.226066	1.074017
PKD2L1	chr26	30220001	30370001	30438465	30482082	4.767839	1.182486
CWF19L1	chr26	30220001	30370001	30497374	30521868	4.767839	1.182486
CHUK	chr26	30220001	30370001	30524704	30565615	4.767839	1.182486
ERLIN1	chr26	30220001	30370001	30568325	30606628	4.767839	1.182486
CPN1	chr26	30220001	30370001	30664670	30693243	4.767839	1.182486
DNMBP	chr26	30220001	30370001	30708273	30822313	4.767839	1.182486
RNAC-GC	chr11	94180001	94330001	93961937	93962008	8.360215	1.08025
KIF4A	chrX1	11720001	11870001	11177125	11304219	4.702624	1.582698
PDZD11	chrX1	11720001	11870001	11304300	11307103	4.702624	1.582698
ARR3	chrX1	11740001	11890001	11313524	11326636	5.109391	1.465974
P2RY4	chrX1	11740001	11890001	11336935	11338033	5.109391	1.465974
AWAT1	chrX1	11740001	11890001	11348658	11355477	5.109391	1.465974
DGAT2L6	chrX1	11740001	11890001	11403406	11425115	5.109391	1.465974
IGBP1	chrX1	11740001	11890001	11436412	11481694	5.109391	1.465974
OTUD6A	chrX1	11740001	11890001	11521708	11522580	5.109391	1.465974
AWAT2	chrX1	11740001	11890001	11544904	11556261	5.109391	1.465974
EDA	chrX1	11740001	11890001	11557022	11895599	5.109391	1.465974
CDX4	chrX1	11740001	11890001	12054961	12063592	5.109391	1.465974
FAM155B	chrX1	11740001	11890001	12074820	12103927	5.109391	1.465974
PJA1	chrX1	11740001	11890001	12110252	12115011	5.109391	1.465974
AR	chrX1	13800001	13950001	13729623	13934065	7.526382	2.276666
RNAC-GC	chr11	94180001	94330001	93961937	93962008	8.360215	1.08025
GRIA3	chrX2	15570001	15720001	15984771	16285966	5.73919	1.063011
TRPC5	chrX2	32390001	32540001	32157960	32499654	4.689455	1.056817
TRPC5OS	chrX2	32390001	32540001	32198714	32199046	4.689455	1.056817
RNAE-UU	chr6	30790001	30940001	31327448	31327519	5.065607	1.07677
DCX	chrX2	32390001	32540001	32724034	32763972	4.689455	1.056817
CAPN6	chrX2	32390001	32540001	32796791	32821641	4.689455	1.056817
PAK3	chrX2	32390001	32540001	32843960	32971791	4.689455	1.056817
RNAW-CC	chrX2	33520001	33670001	33182054	33182125	9.241732	1.111889
RGAG1	chrX2	33520001	33670001	33623413	33629514	9.241732	1.111889
ACSL4	chrX2	33520001	33670001	33813892	33887538	9.241732	1.111889
KCNE5	chrX2	33520001	33670001	33907229	33908705	9.241732	1.111889
GPR119	chrX2	33530001	33680001	34098328	34099849	8.907226	1.092282

Supplementary Table s3:The candidate genes in the selection sweep of other cashmere goats and ordinary goats

Gene	Chr	Bin start	Bin end	Gene Start	Gene end	ZFst	log 2 (θπ ratio)
EPHA6	chr1	39410001	39560001	39786137	40756386	4.174788	1.277423091
TRNAG-CCC	chr6	95430001	95580001	95616042	95616114	12.25061	2.255890527
MFSD1	chr1	108680001	108830001	108264279	108290226	4.786555	1.14244988
RARRES1	chr1	108680001	108830001	108362584	108403869	4.786555	1.14244988
GFM1	chr1	108680001	108830001	108407040	108459141	4.786555	1.14244988
LXN	chr1	108680001	108830001	108421618	108429033	4.786555	1.14244988
MLF1	chr1	108680001	108830001	108466500	108497214	4.786555	1.14244988
RSRC1	chr1	108680001	108830001	108525105	108973792	4.786555	1.14244988
SHOX2	chr1	108680001	108830001	108977758	108987010	4.786555	1.14244988
FCRL5	chr3	106900001	107050001	107178895	107253732	8.608819	1.496014054
FCRL4	chr3	106900001	107050001	107264514	107288861	8.608819	1.496014054
FCRL3	chr3	106900001	107050001	107353691	107414661	8.608819	1.496014054
FCRL1	chr3	106900001	107050001	107426215	107442566	8.608819	1.496014054
CCDC71L	chr4	72840001	72990001	72481791	72486442	6.168006	1.338863099
NAMPT	chr4	72840001	72990001	72928492	72971902	6.168006	1.338863099
SYPL1	chr4	72840001	72990001	73117696	73148219	6.168006	1.338863099
CDHR3	chr4	72840001	72990001	73197557	73250292	6.168006	1.338863099
ATXN7L1	chr4	72840001	72990001	73341808	73600324	6.168006	1.338863099
PLEKHG7	chr5	22820001	22970001	22348785	22417547	8.296934	1.22204778
EEA1	chr5	22820001	22970001	22421664	22546190	8.296934	1.22204778
TRNAE-CUC	chr5	22820001	22970001	22660968	22661040	8.296934	1.22204778
NUDT4	chr5	22820001	22970001	22964342	22980670	8.296934	1.22204778
UBE2N	chr5	22820001	22970001	22984934	23027066	8.296934	1.22204778
MRPL42	chr5	22820001	22970001	23041060	23070803	8.296934	1.22204778
SOCS2	chr5	22820001	22970001	23129217	23135562	8.296934	1.22204778
CRADD	chr5	22820001	22970001	23232979	23425649	8.296934	1.22204778
ALPK1	chr6	13970001	14120001	13456823	13570751	8.782973	1.80273668
TIFA	chr6	13970001	14120001	13580947	13589288	8.782973	1.80273668
AP1AR	chr6	13970001	14120001	13595155	13624310	8.782973	1.80273668
C6H4orf32	chr6	13970001	14120001	13672177	13715799	8.782973	1.80273668
ANTXR2	chr6	95430001	95580001	95056743	95230556	12.25061	2.255890527
PRDM8	chr6	95430001	95580001	95336875	95356821	12.25061	2.255890527
FGF5	chr6	95430001	95580001	95418992	95440124	12.25061	2.255890527
C6H4orf22	chr6	95430001	95580001	95489192	96199327	12.25061	2.255890527
TRNAG-CCC	chr6	95430001	95580001	95616042	95616114	12.25061	2.255890527
IDUA	chr6	117640001	117790001	117245541	117260545	5.777367	1.9243
SLC26A1	chr6	117640001	117790001	117251803	117259744	5.777367	1.9243
DGKQ	chr6	117640001	117790001	117272335	117280603	5.777367	1.9243
CPLX1	chr6	117640001	117790001	117359637	117405415	5.777367	1.9243
PCGF3	chr6	117640001	117790001	117420955	117474202	5.777367	1.9243
MFSD7	chr6	117640001	117790001	117500584	117509668	5.777367	1.9243
ATP5I	chr6	117640001	117790001	117511862	117513678	5.777367	1.9243
PDE6B	chr6	117640001	117790001	117515512	117543217	5.777367	1.9243
PIGG	chr6	117640001	117790001	117580428	117620781	5.777367	1.9243
POC5	chr10	94330001	94480001	94124060	94156199	6.237345	2.273909881
ANKDD1B	chr10	94330001	94480001	94157042	94234857	6.237345	2.273909881
POLK	chr10	94330001	94480001	94240022	94323047	6.237345	2.273909881
COL4A3BP	chr10	94330001	94480001	94322997	94463887	6.237345	2.273909881
HMGCR	chr10	94330001	94480001	94469555	94491458	6.237345	2.273909881
ANKRD31	chr10	94330001	94480001	94562310	94696787	6.237345	2.273909881
GCNT4	chr10	94330001	94480001	94707551	94736446	6.237345	2.273909881
MTDH	chr14	16000001	16150001	15663731	15721499	4.630596	1.435265938
LAPTM4B	chr14	16000001	16150001	15788488	15854656	4.630596	1.435265938

MATN2	chr14	16000001	16150001	15872116	16041603	4.630596	1.435265938
RPL30	chr14	16470001	16620001	16048416	16051780	4.7791	1.435572797
ERICH5	chr14	16490001	16640001	16063489	16086802	5.414622	1.519189002
RIDA	chr14	16490001	16640001	16096003	16104730	5.414622	1.519189002
POP1	chr14	16490001	16640001	16104883	16139688	5.414622	1.519189002
NIPAL2	chr14	16490001	16640001	16173131	16256193	5.414622	1.519189002
KCNS2	chr14	16490001	16640001	16394213	16400041	5.414622	1.519189002
STK3	chr14	16490001	16640001	16433649	16744347	5.414622	1.519189002
OSR2	chr14	16490001	16640001	16881475	16889328	5.414622	1.519189002
VPS13B	chr14	16490001	16640001	16964332	17751028	5.414622	1.519189002
NCAPD3	chr15	1	150001	8858	69851	5.772881	1.176254531
MS4A13	chr15	1	150001	162114	183809	5.772881	1.176254531
MS4A1	chr15	1	150001	236250	251536	5.772881	1.176254531
MS4A2	chr15	1	150001	361337	370421	5.772881	1.176254531
MS4A3	chr15	1	150001	377886	381698	5.772881	1.176254531
OOSP2	chr15	1	150001	389169	394027	5.772881	1.176254531
TCN1	chr15	1	150001	437880	452872	5.772881	1.176254531
GIF	chr15	1	150001	464132	483135	5.772881	1.176254531
MRPL16	chr15	1	150001	496901	501008	5.772881	1.176254531
STX3	chr15	1	150001	501448	547877	5.772881	1.176254531
PLD5	chr16	33580001	33730001	32961659	33377109	4.750542	1.448461405
BECN2	chr16	33580001	33730001	33433023	33434318	4.750542	1.448461405
MAP1LC3C	chr16	33580001	33730001	33461574	33467259	4.750542	1.448461405
EXO1	chr16	33580001	33730001	33479386	33524373	4.750542	1.448461405
WDR64	chr16	33580001	33730001	33534070	33669850	4.750542	1.448461405
CHML	chr16	33580001	33730001	33677364	33686223	4.750542	1.448461405
OPN3	chr16	33580001	33730001	33707072	33715141	4.750542	1.448461405
KMO	chr16	33580001	33730001	33714087	33781937	4.750542	1.448461405
FH	chr16	33580001	33730001	33797989	33822750	4.750542	1.448461405
RGS7	chr16	33580001	33730001	33938680	34410674	4.750542	1.448461405
SLC43A2	chr19	22930001	23080001	22491187	22530698	5.518331	1.190832045
SCARF1	chr19	22930001	23080001	22536624	22548607	5.518331	1.190832045
RILP	chr19	22930001	23080001	22549902	22553181	5.518331	1.190832045
PRPF8	chr19	22930001	23080001	22553692	22584787	5.518331	1.190832045
TLCD2	chr19	22930001	23080001	22602284	22605673	5.518331	1.190832045
MIR22	chr19	22930001	23080001	22609247	22609347	5.518331	1.190832045
WDR81	chr19	22930001	23080001	22617109	22629107	5.518331	1.190832045
SERPINF2	chr19	22930001	23080001	22629867	22637599	5.518331	1.190832045
SERPINF1	chr19	22930001	23080001	22644651	22656868	5.518331	1.190832045
SMYD4	chr19	22930001	23080001	22657588	22696454	5.518331	1.190832045
RPA1	chr19	22930001	23080001	22696549	22740986	5.518331	1.190832045
RTN4RL1	chr19	22930001	23080001	22769076	22848363	5.518331	1.190832045
DPH1	chr19	22930001	23080001	22852077	22862418	5.518331	1.190832045
OVCA2	chr19	22930001	23080001	22860860	22862418	5.518331	1.190832045
HIC1	chr19	22930001	23080001	22871476	22876247	5.518331	1.190832045
SMG6	chr19	22930001	23080001	22876399	23077179	5.518331	1.190832045
SRR	chr19	22930001	23080001	23077186	23091990	5.518331	1.190832045
TSR1	chr19	22930001	23080001	23090732	23101110	5.518331	1.190832045
SGSM2	chr19	22930001	23080001	23102223	23140042	5.518331	1.190832045
TRNAG-CCC	chr6	95430001	95580001	95616042	95616114	12.25061	2.255890527
MNT	chr19	22930001	23080001	23146013	23162285	5.518331	1.190832045
METTL16	chr19	22930001	23080001	23172365	23237617	5.518331	1.190832045
PAFAH1B1	chr19	22930001	23080001	23302330	23368013	5.518331	1.190832045
CLUH	chr19	22930001	23080001	23373646	23395027	5.518331	1.190832045
RAP1GAP2	chr19	22930001	23080001	23437609	23652991	5.518331	1.190832045
SETD5	chr22	17450001	17600001	16950600	17030658	5.127566	1.810611887

THUMPD3	chr22	17460001	17610001	17048771	17070472	5.382906	1.849405564
SRGAP3	chr22	17460001	17610001	17156766	17404110	5.382906	1.849405564
RAD18	chr22	17460001	17610001	17414359	17523990	5.382906	1.849405564
OXTR	chr22	17460001	17610001	17594776	17607958	5.382906	1.849405564
CAV3	chr22	17460001	17610001	17610114	17622648	5.382906	1.849405564
SSUH2	chr22	17460001	17610001	17670175	17692990	5.382906	1.849405564
LMCD1	chr22	17460001	17610001	17740028	17796768	5.382906	1.849405564
CEP192	chr24	44000001	44150001	43556637	43630586	4.659627	1.688602001
LDLRAD4	chr24	44000001	44150001	43647271	43808160	4.659627	1.688602001
FAM210A	chr24	44000001	44150001	43802788	43825139	4.659627	1.688602001
RNMT	chr24	44000001	44150001	43825214	43852257	4.659627	1.688602001
MC5R	chr24	44000001	44150001	43868653	43873520	4.659627	1.688602001
MC2R	chr24	44000001	44150001	43894659	43910455	4.659627	1.688602001
SETBP1	chr24	44020001	44170001	44577303	44983204	4.595879	1.707368914
DUSP8	chr29	51330001	51480001	50962017	50977940	5.511603	2.942207197
MOB2	chr29	51330001	51480001	50987206	51039264	5.511603	2.942207197
BRSK2	chr29	51330001	51480001	51047154	51097037	5.511603	2.942207197
TOLLIP	chr29	51330001	51480001	51162053	51177156	5.511603	2.942207197
MUC5AC	chr29	51330001	51480001	51246526	51276626	5.511603	2.942207197
MUC2	chr29	51330001	51480001	51317872	51331926	5.511603	2.942207197
EDA	chrX1	12290001	12440001	11557022	11895599	4.182243	1.523792496
CDX4	chrX1	12290001	12440001	12054961	12063592	4.182243	1.523792496
FAM155B	chrX1	12290001	12440001	12074820	12103927	4.182243	1.523792496
PJA1	chrX1	12290001	12440001	12110252	12115011	4.182243	1.523792496
STARD8	chrX1	12290001	12440001	12400560	12415129	4.182243	1.523792496
YIPF6	chrX1	12290001	12440001	12585345	12617792	4.182243	1.523792496
OPHN1	chrX1	12290001	12440001	12692459	13271064	4.182243	1.523792496
AR	chrX1	13840001	13990001	13729623	13934065	6.234787	3.444346479
MAGED1	chrX1	14400001	14550001	14646763	14730609	4.432149	2.270950898
RNAW-CC	chrX1	14400001	14550001	14663110	14663181	4.432149	2.270950898
GSPT2	chrX1	14400001	14550001	14796387	14798923	4.432149	2.270950898
EDA2R	chrX1	18150001	18300001	18412588	18508471	4.786207	2.591552102

Supplementary Table s4:The candidate genes in the selection sweep of white cashmere goats(CDMC,UC,IMC,ILC) and brown cashmere goats(CDB)

Gene	Chr	Bin_start	Bin_end	Gene_Start	Gene_end	ZFst	log 2 (θπ ratio)
TIAM1	chr1	3140001	3290001	2425188	2887657	2.830128008	-1.098814506
KRTAP11-1	chr1	3140001	3290001	3134832	3135697	2.830128008	-1.098814506
KAP8	chr1	3140001	3290001	3215311	3215883	2.830128008	-1.098814506
TMPRSS15	chr1	17450001	17600001	17151749	17305379	4.408351838	-1.197269104
CHODL	chr1	17450001	17600001	17307773	17331943	4.408351838	-1.197269104
C1H21orf91	chr1	17450001	17600001	17836541	17868814	4.408351838	-1.197269104
BTG3	chr1	17460001	17610001	18029148	18048638	4.304015171	-1.176576709
TRNAK-UUU	chr1	17480001	17630001	18036604	18036676	4.2556205	-1.15392797
CXADR	chr1	17480001	17630001	18049029	18115289	4.2556205	-1.15392797
CLDND1	chr1	42740001	42890001	42586817	42594123	3.816634766	-1.145286177
GPR15	chr1	42740001	42890001	42603291	42606012	3.816634766	-1.145286177
CPOX	chr1	42740001	42890001	42654291	42669722	3.816634766	-1.145286177
ST3GAL6	chr1	42740001	42890001	42800705	42887578	3.816634766	-1.145286177
DCBLD2	chr1	42740001	42890001	42886443	42968414	3.816634766	-1.145286177
TRNAE-UUC	chrX1	46010001	46160001	46250912	46250984	5.493789747	1.676718744
BCHE	chr1	101340001	101490001	101031428	101110937	3.238363492	-0.910773058
SLITRK3	chr1	101340001	101490001	101714521	101725766	3.238363492	-0.910773058
SI	chr1	101340001	101490001	101811454	101920451	3.238363492	-0.910773058
TRNAG-CCC	chrX2	10370001	10520001	10239324	10239396	3.767169191	1.355072662
MFSD1	chr1	108580001	108730001	108264279	108290226	2.760128269	1.185676287
RARRES1	chr1	108580001	108730001	108362584	108403869	2.760128269	1.185676287
GFM1	chr1	108580001	108730001	108407040	108459141	2.760128269	1.185676287
LXN	chr1	108580001	108730001	108421618	108429033	2.760128269	1.185676287
MLF1	chr1	108580001	108730001	108466500	108497214	2.760128269	1.185676287
RSRC1	chr1	108580001	108730001	108525105	108973792	2.760128269	1.185676287
SHOX2	chr1	108580001	108730001	108977758	108987010	2.760128269	1.185676287
RAP2B	chr1	114630001	114780001	114205077	114214900	2.765992745	-1.981081018
P2RY1	chr1	114690001	114840001	114567590	114574104	3.362163418	-1.376694972
MBNL1	chr1	114690001	114840001	114974326	115192808	3.362163418	-1.376694972
SUCNR1	chr1	115610001	115760001	115626920	115636639	2.554276682	-1.711700505
C1H3orf58	chr1	124370001	124520001	124443207	124467469	3.343312104	-1.165207895
SLC9A9	chr1	124370001	124520001	124629179	125280384	3.343312104	-1.165207895
TRNAK-UUU	chr1	17480001	17630001	18036604	18036676	4.2556205	-1.15392797
FOXL2	chr1	130100001	130250001	129771906	129773370	3.33967443	-1.104076998
PIK3CB	chr1	130100001	130250001	129881817	130060986	3.33967443	-1.104076998
FAIM	chr1	130100001	130250001	130073736	130089218	3.33967443	-1.104076998
CEP70	chr1	130100001	130250001	130089234	130186513	3.33967443	-1.104076998
ESYT3	chr1	130100001	130250001	130189979	130245320	3.33967443	-1.104076998
MRAS	chr1	130100001	130250001	130284683	130352520	3.33967443	-1.104076998
NME9	chr1	130100001	130250001	130372691	130395986	3.33967443	-1.104076998
ARMC8	chr1	130100001	130250001	130401143	130507389	3.33967443	-1.104076998
DBR1	chr1	130100001	130250001	130521468	130533958	3.33967443	-1.104076998
A4GNT	chr1	130100001	130250001	130549744	130585303	3.33967443	-1.104076998
DZIP1L	chr1	130100001	130250001	130589968	130649459	3.33967443	-1.104076998
TRNAW-CCA	chrX1	14400001	14550001	14663110	14663181	4.407790888	1.145351386
CPNE4	chr1	138000001	138150001	137317163	137966558	3.755695217	-1.016833084
MRPL3	chr1	138000001	138150001	138002275	138059429	3.755695217	-1.016833084
NUDT16	chr1	138000001	138150001	138166503	138168104	3.755695217	-1.016833084
NEK11	chr1	138000001	138150001	138180436	138467344	3.755695217	-1.016833084
ASTE1	chr1	138000001	138150001	138467431	138489609	3.755695217	-1.016833084
ATP2C1	chr1	138000001	138150001	138497975	138651086	3.755695217	-1.016833084
ARMC9	chr2	17120001	17270001	16579176	16761996	2.860810263	-0.907251314
PSMD1	chr2	17120001	17270001	16782175	16865843	2.860810263	-0.907251314
HTR2B	chr2	17120001	17270001	16821118	16836903	2.860810263	-0.907251314
C2H2orf72	chr2	17120001	17270001	16874291	16884102	2.860810263	-0.907251314
TRNAW-CCA	chrX1	14400001	14550001	14663110	14663181	4.407790888	1.145351386
GPR55	chr2	17120001	17270001	17012406	17013648	2.860810263	-0.907251314
ITM2C	chr2	17120001	17270001	17046174	17060341	2.860810263	-0.907251314
CAB39	chr2	17120001	17270001	17092152	17190120	2.860810263	-0.907251314
SP140	chr2	17120001	17270001	17416819	17496056	2.860810263	-0.907251314
TRNAS-GGA	chr4	52950001	53100001	53090579	53090650	4.326759136	-0.932085986
SP110	chr2	17120001	17270001	17496060	17548527	2.860810263	-0.907251314
SLC16A14	chr2	17120001	17270001	17618698	17652976	2.860810263	-0.907251314
TRIP12	chr2	17190001	17340001	17763215	17916591	2.508788751	-1.061601281
RAB3GAP1	chr2	74440001	74590001	73996763	74121959	3.406274472	-1.30293792
ZRANB3	chr2	74440001	74590001	74145804	74436792	3.406274472	-1.30293792
R3HDM1	chr2	74440001	74590001	74437490	74600543	3.406274472	-1.30293792

MIR128	chr2	74440001	74590001	74560517	74560648	3.406274472	-1.30293792
UBXN4	chr2	74440001	74590001	74619014	74648704	3.406274472	-1.30293792
LCT	chr2	74440001	74590001	74649213	74699787	3.406274472	-1.30293792
MCM6	chr2	74440001	74590001	74708434	74745046	3.406274472	-1.30293792
DARS	chr2	74440001	74590001	74780841	74845981	3.406274472	-1.30293792
CXCR4	chr2	74440001	74590001	74976699	74980389	3.406274472	-1.30293792
TRNAT-AGU	chr2	75320001	75470001	75422127	75422200	2.619907812	-0.932636633
TRNAC-GCA	chr16	62530001	62680001	62935749	62935820	6.204615165	-0.939260842
THSD7B	chr2	75320001	75470001	75653642	76691998	2.619907812	-0.932636633
GALNT5	chr2	97410001	97560001	97082918	97124602	4.62275366	-1.02385636
ERMN	chr2	97410001	97560001	97130255	97137921	4.62275366	-1.02385636
CYTIP	chr2	97410001	97560001	97216183	97246728	4.62275366	-1.02385636
ACVR1C	chr2	97410001	97560001	97335789	97391095	4.62275366	-1.02385636
ACVR1	chr2	97410001	97560001	97581866	97719746	4.62275366	-1.02385636
UPP2	chr2	97410001	97560001	97918654	97959126	4.62275366	-1.02385636
CCDC148	chr2	97420001	97570001	97989039	98211940	4.620254884	-1.054392297
BAZ2B	chr2	99810001	99960001	99134862	99464817	3.174041245	-0.995678394
7-Mar	chr2	99810001	99960001	99549258	99596850	3.174041245	-0.995678394
PLA2R1	chr2	99810001	99960001	99763166	99888468	3.174041245	-0.995678394
ITGB6	chr2	99810001	99960001	99910920	100053272	3.174041245	-0.995678394
RBMS1	chr2	99810001	99960001	100074882	100296249	3.174041245	-0.995678394
ARL4C	chr3	6780001	6930001	6672190	6676211	2.521384625	-0.999423037
SPP2	chr3	6780001	6930001	7052998	7080042	2.521384625	-0.999423037
TRPM8	chr3	6780001	6930001	7101606	7208432	2.521384625	-0.999423037
HJURP	chr3	6780001	6930001	7253797	7266899	2.521384625	-0.999423037
MROH2A	chr3	6780001	6930001	7287456	7345980	2.521384625	-0.999423037
ZC3H12A	chr3	12760001	12910001	12397169	12407546	4.078904307	0.909581116
MEAF6	chr3	12760001	12910001	12415872	12440541	4.078904307	0.909581116
SNIP1	chr3	12760001	12910001	12453766	12469774	4.078904307	0.909581116
DNALI1	chr3	12760001	12910001	12470869	12479526	4.078904307	0.909581116
GNL2	chr3	12760001	12910001	12479488	12503646	4.078904307	0.909581116
RSPO1	chr3	12760001	12910001	12519202	12541600	4.078904307	0.909581116
C3H1orf109	chr3	12760001	12910001	12591206	12598018	4.078904307	0.909581116
CDCA8	chr3	12760001	12910001	12598174	12613416	4.078904307	0.909581116
EPHA10	chr3	12760001	12910001	12615370	12659007	4.078904307	0.909581116
MANEAL	chr3	12760001	12910001	12685596	12694130	4.078904307	0.909581116
YRDC	chr3	12760001	12910001	12693862	12697616	4.078904307	0.909581116
MTF1	chr3	12760001	12910001	12698723	12744794	4.078904307	0.909581116
INPP5B	chr3	12760001	12910001	12745894	12797875	4.078904307	0.909581116
SF3A3	chr3	12760001	12910001	12807257	12831210	4.078904307	0.909581116
FHL3	chr3	12760001	12910001	12835146	12842774	4.078904307	0.909581116
UTP11	chr3	12760001	12910001	12849439	12860707	4.078904307	0.909581116
POU3F1	chr3	12760001	12910001	12879313	12881446	4.078904307	0.909581116
RLF	chr3	15250001	15400001	14836369	14922369	3.789029236	-0.943971461
TMCO2	chr3	15250001	15400001	14929793	14932608	3.789029236	-0.943971461
ZMPSTE24	chr3	15250001	15400001	14950764	14993348	3.789029236	-0.943971461
COL9A2	chr3	15250001	15400001	15000986	15016479	3.789029236	-0.943971461
SMAP2	chr3	15250001	15400001	15053632	15102158	3.789029236	-0.943971461
ZFP69B	chr3	15250001	15400001	15120282	15144459	3.789029236	-0.943971461
ZFP69	chr3	15250001	15400001	15148977	15165365	3.789029236	-0.943971461
RIMS3	chr3	15250001	15400001	15250367	15295283	3.789029236	-0.943971461
NFYC	chr3	15250001	15400001	15315404	15381493	3.789029236	-0.943971461
MIR30E	chr3	15250001	15400001	15365532	15365637	3.789029236	-0.943971461
MIR30C	chr3	15250001	15400001	15368653	15368757	3.789029236	-0.943971461
KCNQ4	chr3	15250001	15400001	15393310	15452416	3.789029236	-0.943971461
CITED4	chr3	15250001	15400001	15475017	15476378	3.789029236	-0.943971461
CTPS1	chr3	15250001	15400001	15594936	15625865	3.789029236	-0.943971461
SLFNL1	chr3	15250001	15400001	15628164	15633250	3.789029236	-0.943971461
SCMH1	chr3	15250001	15400001	15637457	15855355	3.789029236	-0.943971461
FOXO6	chr3	15540001	15690001	15982854	16004565	2.97141937	-1.083446923
EDN2	chr3	15540001	15690001	16110938	16116874	2.97141937	-1.083446923
HIVEP3	chr3	15570001	15720001	16143582	16659535	2.918248126	-1.282438723
GUCA2B	chr3	17120001	17270001	16750513	16753710	2.800074696	-1.238544507
GUCA2A	chr3	17120001	17270001	16757271	16760306	2.800074696	-1.238544507
FOXJ3	chr3	17120001	17270001	16769526	16920659	2.800074696	-1.238544507
RIMKLA	chr3	17120001	17270001	16994111	17027435	2.800074696	-1.238544507
ZMYND12	chr3	17120001	17270001	17048117	17083139	2.800074696	-1.238544507
PPCS	chr3	17120001	17270001	17083553	17224415	2.800074696	-1.238544507
PPIH	chr3	17120001	17270001	17227464	17244672	2.800074696	-1.238544507
YBX1	chr3	17120001	17270001	17249139	17268236	2.800074696	-1.238544507
CLDN19	chr3	17120001	17270001	17281990	17287819	2.800074696	-1.238544507

P3H1	chr3	17120001	17270001	17294547	17313795	2.800074696	-1.238544507
C3H1orf50	chr3	17120001	17270001	17313998	17323506	2.800074696	-1.238544507
SVBP	chr3	17120001	17270001	17346323	17352490	2.800074696	-1.238544507
ERMAP	chr3	17120001	17270001	17372886	17384417	2.800074696	-1.238544507
ZNF691	chr3	17120001	17270001	17386195	17391582	2.800074696	-1.238544507
SLC2A1	chr3	17120001	17270001	17438803	17472481	2.800074696	-1.238544507
OLFM3	chr3	79040001	79190001	79015350	79240851	3.670736817	-2.021511467
COL11A1	chr3	79980001	80130001	79996781	80226730	5.910728453	-1.398969131
RNPC3	chr3	80100001	80250001	80656949	80688529	5.258819765	-1.359194004
VIPR2	chr4	330001	480001	171628	239456	3.831389447	-1.102217455
WDR60	chr4	330001	480001	293177	346902	3.831389447	-1.102217455
ESYT2	chr4	330001	480001	366433	449121	3.831389447	-1.102217455
NCAPG2	chr4	330001	480001	470768	542701	3.831389447	-1.102217455
PTPRN2	chr4	1470001	1620001	621406	1167657	4.347021324	-1.311148256
DNAJB6	chr4	1470001	1620001	1221408	1266789	4.347021324	-1.311148256
UBE3C	chr4	1470001	1620001	1312658	1426090	4.347021324	-1.311148256
MNX1	chr4	1470001	1620001	1503421	1508316	4.347021324	-1.311148256
NOM1	chr4	1470001	1620001	1526709	1539384	4.347021324	-1.311148256
LMBR1	chr4	1470001	1620001	1584560	1720496	4.347021324	-1.311148256
RNF32	chr4	1470001	1620001	1734604	1770786	4.347021324	-1.311148256
SHH	chr4	2630001	2780001	2388104	2398833	2.938255336	-0.924125133
RBM33	chr4	2630001	2780001	2421360	2530454	2.938255336	-0.924125133
CNPY1	chr4	2630001	2780001	2618556	2628346	2.938255336	-0.924125133
EN2	chr4	2630001	2780001	2659430	2666542	2.938255336	-0.924125133
INSIG1	chr4	2630001	2780001	2747017	2757803	2.938255336	-0.924125133
HTR5A	chr4	2630001	2780001	2878884	2895668	2.938255336	-0.924125133
PAXIP1	chr4	2630001	2780001	2922811	2963751	2.938255336	-0.924125133
DPP6	chr4	2630001	2780001	2979751	3954235	2.938255336	-0.924125133
MIR148A	chr4	50470001	50620001	50269709	50269793	3.043985879	-1.03209363
NFE2L3	chr4	50890001	51040001	50434030	50467648	5.68135097	-1.211564852
HNRNPA2B1	chr4	50890001	51040001	50469472	50479267	5.68135097	-1.211564852
CBX3	chr4	50890001	51040001	50480214	50491070	5.68135097	-1.211564852
SNX10	chr4	50890001	51040001	50549549	50616271	5.68135097	-1.211564852
SKAP2	chr4	50890001	51040001	50900784	51072547	5.68135097	-1.211564852
HOXA1	chr4	50890001	51040001	51262987	51265727	5.68135097	-1.211564852
HOXA2	chr4	50890001	51040001	51270010	51272952	5.68135097	-1.211564852
HOXA3	chr4	50890001	51040001	51276661	51283467	5.68135097	-1.211564852
HOXA4	chr4	50890001	51040001	51297397	51299705	5.68135097	-1.211564852
HOXA5	chr4	50890001	51040001	51309920	51312896	5.68135097	-1.211564852
HOXA6	chr4	50890001	51040001	51314374	51319005	5.68135097	-1.211564852
HOXA7	chr4	50890001	51040001	51323653	51329798	5.68135097	-1.211564852
HOXA9	chr4	50890001	51040001	51331211	51337701	5.68135097	-1.211564852
MIR196B	chr4	50890001	51040001	51338288	51338372	5.68135097	-1.211564852
HOXA10	chr4	50890001	51040001	51339407	51343166	5.68135097	-1.211564852
HOXA11	chr4	50890001	51040001	51350542	51354278	5.68135097	-1.211564852
HOXA13	chr4	50890001	51040001	51366007	51369167	5.68135097	-1.211564852
EVX1	chr4	50890001	51040001	51410813	51414731	5.68135097	-1.211564852
HIBADH	chr4	51990001	52140001	51633820	51746218	3.728038691	-1.226003675
TAX1BP1	chr4	51990001	52140001	51817207	51906819	3.728038691	-1.226003675
JAZF1	chr4	51990001	52140001	51907478	52239310	3.728038691	-1.226003675
CREB5	chr4	52950001	53100001	52482298	52922180	4.326759136	-0.932085986
TRIL	chr4	52950001	53100001	53029117	53034303	4.326759136	-0.932085986
CPVL	chr4	52950001	53100001	53082853	53188759	4.326759136	-0.932085986
TRNAS-GGA	chr4	52950001	53100001	53090579	53090650	4.326759136	-0.932085986
CHN2	chr4	53650001	53800001	53262143	53602288	4.41615414	-0.960159735
PRR15	chr4	53650001	53800001	53620856	53624213	4.41615414	-0.960159735
WIPF3	chr4	53650001	53800001	53725612	53809373	4.41615414	-0.960159735
SCRN1	chr4	53650001	53800001	53813414	53877817	4.41615414	-0.960159735
FKBP14	chr4	53650001	53800001	53892767	53904759	4.41615414	-0.960159735
PLEKHA8	chr4	53650001	53800001	53907086	53976470	4.41615414	-0.960159735
MTURN	chr4	53650001	53800001	54025518	54060461	4.41615414	-0.960159735
ZNRF2	chr4	53650001	53800001	54186584	54278606	4.41615414	-0.960159735
TRNAC-ACA	chr16	62530001	62680001	62246657	62246728	6.204615165	-0.939260842
HGF	chr4	81210001	81360001	81306673	81397853	3.031220021	-2.544560985
CACNA2D1	chr4	81210001	81360001	81665818	82201273	3.031220021	-2.544560985
LRRIQ1	chr5	15070001	15220001	14530561	14741764	2.690213523	-1.00433459
ALX1	chr5	15070001	15220001	14780433	14802313	2.690213523	-1.00433459
RASSF9	chr5	15070001	15220001	15329017	15361750	2.690213523	-1.00433459
NTS	chr5	15070001	15220001	15391221	15402965	2.690213523	-1.00433459
MGAT4C	chr5	15070001	15220001	15533616	15603737	2.690213523	-1.00433459
LRIG3	chr5	53650001	53800001	53854406	53905944	3.579403986	-1.141461934

ALPK1	chr6	13960001	14110001	13456823	13570751	4.433424595	1.429374937
TIFA	chr6	13960001	14110001	13580947	13589288	4.433424595	1.429374937
AP1AR	chr6	13960001	14110001	13595155	13624310	4.433424595	1.429374937
C6H4orf32	chr6	13960001	14110001	13672177	13715799	4.433424595	1.429374937
LEF1	chr6	17940001	18090001	17398506	17521097	3.559532764	-1.271600541
HADH	chr6	17980001	18130001	17529001	17573045	3.613077974	-1.044538396
SGMS2	chr6	18010001	18160001	17613593	17710656	3.67707725	-0.971713757
PAPSS1	chr6	18010001	18160001	17802392	17916090	3.67707725	-0.971713757
DKK2	chr6	18010001	18160001	18461852	18590125	3.67707725	-0.971713757
NPNT	chr6	20000001	20150001	19517104	19594712	2.838304277	-1.015374195
GSTCD	chr6	20040001	20190001	19640852	19780384	2.9174152	-0.921390165
INTS12	chr6	20040001	20190001	19780400	19801936	2.9174152	-0.921390165
ARHGEF38	chr6	20040001	20190001	19807399	19954771	2.9174152	-0.921390165
PPA2	chr6	20040001	20190001	20029174	20144590	2.9174152	-0.921390165
TET2	chr6	20040001	20190001	20216813	20352863	2.9174152	-0.921390165
PPARGC1A	chr6	43930001	44080001	43798769	43919972	3.061341326	-0.942307133
DHX15	chr6	43990001	44140001	44553729	44609199	2.949219355	-0.968604804
TRNAW-CCA	chrX1	14400001	14550001	14663110	14663181	4.407790888	1.145351386
APBB2	chr6	60980001	61130001	60285849	60665198	2.653700789	-0.911044318
UCHL1	chr6	60980001	61130001	60715873	60727733	2.653700789	-0.911044318
LIMCH1	chr6	60980001	61130001	60794267	61154327	2.653700789	-0.911044318
PHOX2B	chr6	60980001	61130001	61216690	61220345	2.653700789	-0.911044318
TMEM33	chr6	60980001	61130001	61387898	61407481	2.653700789	-0.911044318
SLC30A9	chr6	60980001	61130001	61429091	61509809	2.653700789	-0.911044318
BEND4	chr6	60980001	61130001	61544757	61574269	2.653700789	-0.911044318
GABRG1	chr6	65110001	65260001	65127653	65214852	3.843747341	-0.923577725
GABRA2	chr6	65110001	65260001	65342451	65518411	3.843747341	-0.923577725
TRNAW-CCA	chrX1	14400001	14550001	14663110	14663181	4.407790888	1.145351386
TRNAY-GUA	chr6	79390001	79540001	79799916	79799987	3.084561249	0.999567127
EPHA5	chr6	81310001	81460001	81441793	81853316	3.613876903	-1.1824586
ANKRD17	chr6	88780001	88930001	88712562	88880682	4.571690228	0.941481964
ALB	chr6	88780001	88930001	89001769	89019686	4.571690228	0.941481964
AFP	chr6	88780001	88930001	89027050	89048856	4.571690228	0.941481964
AFM	chr6	88780001	88930001	89058533	89081033	4.571690228	0.941481964
RASSF6	chr6	88780001	88930001	89142702	89210900	4.571690228	0.941481964
CXCL8	chr6	88780001	88930001	89335401	89338881	4.571690228	0.941481964
ANTXR2	chr6	95410001	95560001	95056743	95230556	3.540035508	1.369494402
PRDM8	chr6	95410001	95560001	95336875	95356821	3.540035508	1.369494402
FGF5	chr6	95410001	95560001	95418992	95440124	3.540035508	1.369494402
C6H4orf22	chr6	95410001	95560001	95489192	96199327	3.540035508	1.369494402
TRNAG-CCC	chrX2	10370001	10520001	10239324	10239396	3.767169191	1.355072662
CAST	chr7	14990001	15140001	14434087	14568155	3.733767179	-0.932912035
TRNAW-CCA	chrX1	14400001	14550001	14663110	14663181	4.407790888	1.145351386
PCSK1	chr7	15000001	15150001	14805715	14858039	3.850053777	-0.973979029
ELL2	chr7	15000001	15150001	15292347	15367401	3.850053777	-0.973979029
GLRX	chr7	15000001	15150001	15436081	15445521	3.850053777	-0.973979029
RHOBTB3	chr7	15000001	15150001	15465965	15527868	3.850053777	-0.973979029
SPATA9	chr7	15000001	15150001	15554156	15584327	3.850053777	-0.973979029
RFESD	chr7	15000001	15150001	15559116	15595714	3.850053777	-0.973979029
TRNAC-GCA	chr16	62530001	62680001	62935749	62935820	6.204615165	-0.939260842
HAND1	chr7	44960001	45110001	44621033	44624336	2.54278571	0.960734102
SAP30L	chr7	44960001	45110001	44638848	44652660	2.54278571	0.960734102
GALNT10	chr7	44960001	45110001	44665723	44897352	2.54278571	0.960734102
MFAP3	chr7	44960001	45110001	45031201	45048036	2.54278571	0.960734102
FAM114A2	chr7	44960001	45110001	45048066	45084637	2.54278571	0.960734102
GRIA1	chr7	44960001	45110001	45259914	45615155	2.54278571	0.960734102
ARAP3	chr7	58160001	58310001	57741258	57769308	3.609644281	-1.373327247
FCHSD1	chr7	58160001	58310001	57771763	57784020	3.609644281	-1.373327247
RELL2	chr7	58160001	58310001	57782218	57786366	3.609644281	-1.373327247
HDAC3	chr7	58160001	58310001	57786492	57799552	3.609644281	-1.373327247
DIAPH1	chr7	58160001	58310001	57801641	57918084	3.609644281	-1.373327247
TAF7	chr7	58160001	58310001	58114795	58117019	3.609644281	-1.373327247
PCDHB14	chr7	58160001	58310001	58185242	58189234	3.609644281	-1.373327247
PCDHB16	chr7	58160001	58310001	58214667	58217524	3.609644281	-1.373327247
PCDHB7	chr7	58160001	58310001	58225893	58228883	3.609644281	-1.373327247
PCDHB1	chr7	58160001	58310001	58350170	58352697	3.609644281	-1.373327247
TRNAC-GCA	chr16	62530001	62680001	62935749	62935820	6.204615165	-0.939260842
ZMAT2	chr7	58160001	58310001	58668572	58674067	3.609644281	-1.373327247
HARS2	chr7	58160001	58310001	58674889	58682059	3.609644281	-1.373327247
HARS	chr7	58160001	58310001	58682131	58695144	3.609644281	-1.373327247
DND1	chr7	58160001	58310001	58695237	58698549	3.609644281	-1.373327247

WDR55	chr7	58160001	58310001	58698546	58703385	3.609644281	-1.373327247
IK	chr7	58160001	58310001	58705364	58718109	3.609644281	-1.373327247
NDUFA2	chr7	58160001	58310001	58718219	58720374	3.609644281	-1.373327247
TMCO6	chr7	58160001	58310001	58720332	58725924	3.609644281	-1.373327247
CD14	chr7	58160001	58310001	58731680	58735123	3.609644281	-1.373327247
SLC35A4	chr7	58220001	58370001	58754826	58759575	3.463253376	-1.252913417
APBB3	chr7	58220001	58370001	58759676	58766097	3.463253376	-1.252913417
SRA1	chr7	58220001	58370001	58766299	58773099	3.463253376	-1.252913417
EIF4EBP3	chr7	58220001	58370001	58773687	58774464	3.463253376	-1.252913417
CXXC5	chr7	60010001	60160001	59617412	59651710	3.18617816	-1.009843833
UBE2D2	chr7	60010001	60160001	59671314	59714771	3.18617816	-1.009843833
TMEM173	chr7	60010001	60160001	59759510	59765656	3.18617816	-1.009843833
ECSCR	chr7	60010001	60160001	59772140	59780273	3.18617816	-1.009843833
DNAJC18	chr7	60010001	60160001	59788431	59817336	3.18617816	-1.009843833
SPATA24	chr7	60010001	60160001	59822027	59827252	3.18617816	-1.009843833
PROB1	chr7	60010001	60160001	59828342	59833722	3.18617816	-1.009843833
MZB1	chr7	60010001	60160001	59834329	59836445	3.18617816	-1.009843833
SLC23A1	chr7	60010001	60160001	59837143	59862223	3.18617816	-1.009843833
PAIP2	chr7	60010001	60160001	59849786	59867040	3.18617816	-1.009843833
MATR3	chr7	60010001	60160001	59873045	59911585	3.18617816	-1.009843833
SIL1	chr7	60010001	60160001	59972174	60233205	3.18617816	-1.009843833
CTNNA1	chr7	60010001	60160001	60240857	60414279	3.18617816	-1.009843833
LRRTM2	chr7	60010001	60160001	60304287	60307637	3.18617816	-1.009843833
TRNAS-GGA	chr4	52950001	53100001	53090579	53090650	4.326759136	-0.932085986
TRNAC-GCA	chr16	62530001	62680001	62935749	62935820	6.204615165	-0.939260842
HSPA9	chr7	60010001	60160001	60577216	60593487	3.18617816	-1.009843833
PDLIM4	chr7	88850001	89000001	88413050	88427472	2.875513948	0.916935177
SLC22A4	chr7	88860001	89010001	88444408	88487123	2.945071726	0.934780673
SLC22A5	chr7	88860001	89010001	88511443	88537601	2.945071726	0.934780673
IRF1	chr7	88860001	89010001	88630486	88637730	2.945071726	0.934780673
IL5	chr7	88860001	89010001	88684078	88686626	2.945071726	0.934780673
RAD50	chr7	88860001	89010001	88702532	88815957	2.945071726	0.934780673
TRNAC-GCA	chr16	62530001	62680001	62935749	62935820	6.204615165	-0.939260842
IL13	chr7	88860001	89010001	88859199	88862094	2.945071726	0.934780673
IL4	chr7	88860001	89010001	88878436	88886254	2.945071726	0.934780673
KIF3A	chr7	88860001	89010001	88891761	88973815	2.945071726	0.934780673
8-Sep	chr7	88860001	89010001	88981804	89009199	2.945071726	0.934780673
MKNK2	chr7	88860001	89010001	89073549	89085155	2.945071726	0.934780673
MOB3A	chr7	88860001	89010001	89102714	89121739	2.945071726	0.934780673
IZUMO4	chr7	88860001	89010001	89121955	89125128	2.945071726	0.934780673
AP3D1	chr7	88860001	89010001	89126591	89159963	2.945071726	0.934780673
JSRP1	chr7	88860001	89010001	89165304	89169175	2.945071726	0.934780673
AMH	chr7	88860001	89010001	89169334	89172804	2.945071726	0.934780673
SF3A2	chr7	88860001	89010001	89172888	89181602	2.945071726	0.934780673
PLEKHJ1	chr7	88860001	89010001	89181731	89184374	2.945071726	0.934780673
DOT1L	chr7	88860001	89010001	89184931	89236978	2.945071726	0.934780673
TRNAG-CCC	chrX2	10370001	10520001	10239324	10239396	3.767169191	1.355072662
OAZ1	chr7	88860001	89010001	89278872	89282376	2.945071726	0.934780673
C7H19orf35	chr7	88860001	89010001	89283752	89289772	2.945071726	0.934780673
LINGO3	chr7	88860001	89010001	89295581	89311797	2.945071726	0.934780673
LSM7	chr7	88860001	89010001	89322318	89326916	2.945071726	0.934780673
SPPL2B	chr7	88860001	89010001	89327012	89341777	2.945071726	0.934780673
TMPRSS9	chr7	88860001	89010001	89368992	89400432	2.945071726	0.934780673
TIMM13	chr7	88860001	89010001	89408589	89409818	2.945071726	0.934780673
LMNB2	chr7	88860001	89010001	89410232	89430556	2.945071726	0.934780673
RIC1	chr8	39340001	39490001	38776219	38919131	3.325735677	-0.984787915
PDCD1LG2	chr8	39350001	39500001	38979921	39046980	3.366685014	-1.092032594
CD274	chr8	39350001	39500001	39077983	39095141	3.366685014	-1.092032594
PLGRKT	chr8	39350001	39500001	39111116	39155487	3.366685014	-1.092032594
INSL6	chr8	39350001	39500001	39210866	39227365	3.366685014	-1.092032594
JAK2	chr8	39350001	39500001	39245187	39360411	3.366685014	-1.092032594
RCL1	chr8	39350001	39500001	39520371	39583801	3.366685014	-1.092032594
AK3	chr8	39350001	39500001	39635494	39656272	3.366685014	-1.092032594
CDC37L1	chr8	39350001	39500001	39660101	39678648	3.366685014	-1.092032594
PLPP6	chr8	39350001	39500001	39700133	39703413	3.366685014	-1.092032594
SPATA6L	chr8	39350001	39500001	39734093	39756809	3.366685014	-1.092032594
SLC1A1	chr8	39350001	39500001	39770036	39845247	3.366685014	-1.092032594
DNAI1	chr8	76080001	76230001	75600294	75669575	3.541395386	-1.144648099
ENHO	chr8	76080001	76230001	75669638	75671745	3.541395386	-1.144648099
CNTFR	chr8	76080001	76230001	75695438	75782519	3.541395386	-1.144648099
RPP25L	chr8	76080001	76230001	75801233	75803474	3.541395386	-1.144648099

DCTN3	chr8	76080001	76230001	75804882	75812788	3.541395386	-1.144648099
ARID3C	chr8	76080001	76230001	75814303	75821142	3.541395386	-1.144648099
SIGMAR1	chr8	76080001	76230001	75828160	75830978	3.541395386	-1.144648099
GALT	chr8	76080001	76230001	75837855	75841221	3.541395386	-1.144648099
IL11RA	chr8	76080001	76230001	75843232	75852734	3.541395386	-1.144648099
CCL27	chr8	76080001	76230001	75852804	75854562	3.541395386	-1.144648099
CCL19	chr8	76080001	76230001	75873840	75875825	3.541395386	-1.144648099
CCL21	chr8	76080001	76230001	75895977	75897160	3.541395386	-1.144648099
RASEF	chr8	76080001	76230001	76002648	76091226	3.541395386	-1.144648099
FRMD3	chr8	76080001	76230001	76202142	76554906	3.541395386	-1.144648099
TRNAC-ACA	chr16	62530001	62680001	62246657	62246728	6.204615165	-0.939260842
IDNK	chr8	76080001	76230001	76615850	76633688	3.541395386	-1.144648099
UBQLN1	chr8	76730001	76880001	76659014	76713744	3.108665093	-0.929885497
GKAP1	chr8	76730001	76880001	76763253	76861623	3.108665093	-0.929885497
KIF27	chr8	76730001	76880001	76879454	76969689	3.108665093	-0.929885497
C8H9orf64	chr8	76730001	76880001	76983174	76995044	3.108665093	-0.929885497
TRNAC-GCA	chr16	62530001	62680001	62935749	62935820	6.204615165	-0.939260842
HNRNPK	chr8	76730001	76880001	77011076	77023300	3.108665093	-0.929885497
MIR7	chr8	76730001	76880001	77012728	77012874	3.108665093	-0.929885497
RMI1	chr8	76730001	76880001	77023315	77038142	3.108665093	-0.929885497
SLC28A3	chr8	76730001	76880001	77279655	77345734	3.108665093	-0.929885497
GRIK2	chr9	36000001	36150001	35137287	35864369	2.917364205	-0.938431151
ASCC3	chr9	36000001	36150001	36344650	36689896	2.917364205	-0.938431151
PTPRK	chr9	53900001	54050001	53315015	53933095	2.50868676	-2.284897364
LAMA2	chr9	53900001	54050001	54306124	54983404	2.50868676	-2.284897364
AKAP7	chr9	56990001	57140001	56487185	56612712	3.440951371	1.066054738
ARG1	chr9	56990001	57140001	56815375	56830671	3.440951371	1.066054738
MED23	chr9	56990001	57140001	56831657	56873993	3.440951371	1.066054738
ENPP3	chr9	56990001	57140001	56897818	56987489	3.440951371	1.066054738
ENPP1	chr9	56990001	57140001	57045830	57117809	3.440951371	1.066054738
CTGF	chr9	56990001	57140001	57175950	57179282	3.440951371	1.066054738
MOXD1	chr9	56990001	57140001	57534766	57632301	3.440951371	1.066054738
TRNAC-ACA	chr16	62530001	62680001	62246657	62246728	6.204615165	-0.939260842
SPTB	chr10	26650001	26800001	26106676	26234517	2.892886394	-0.934841325
PLEKHG3	chr10	26680001	26830001	26235988	26281149	2.989233776	-0.9776677
PPP1R36	chr10	26680001	26830001	26407387	26430457	2.989233776	-0.9776677
HSPA2	chr10	26680001	26830001	26436107	26438017	2.989233776	-0.9776677
ZBTB1	chr10	26680001	26830001	26442455	26467963	2.989233776	-0.9776677
ZBTB25	chr10	26680001	26830001	26467957	26514294	2.989233776	-0.9776677
AKAP5	chr10	26680001	26830001	26500237	26505331	2.989233776	-0.9776677
MTHFD1	chr10	26680001	26830001	26510059	26572160	2.989233776	-0.9776677
ESR2	chr10	26680001	26830001	26669088	26714330	2.989233776	-0.9776677
SYNE2	chr10	26680001	26830001	26722712	27046703	2.989233776	-0.9776677
TRNAW-CCA	chrX1	14400001	14550001	14663110	14663181	4.407790888	1.145351386
SGPP1	chr10	26680001	26830001	27134837	27177851	2.989233776	-0.9776677
WDR89	chr10	26680001	26830001	27228084	27276570	2.989233776	-0.9776677
ZFHX2	chr10	80470001	80620001	80016831	80045551	5.355626106	1.20614307
THTPA	chr10	80470001	80620001	80048227	80052486	5.355626106	1.20614307
AP1G2	chr10	80480001	80630001	80051423	80059940	5.849465933	1.301821736
JPH4	chr10	80490001	80640001	80060027	80070268	6.479888542	1.486199493
CARMIL3	chr10	80560001	80710001	80346563	80364380	7.493252898	1.331705216
CPNE6	chr10	80560001	80710001	80365429	80372592	7.493252898	1.331705216
NRL	chr10	80560001	80710001	80374515	80388480	7.493252898	1.331705216
PCK2	chr10	80560001	80710001	80388956	80397146	7.493252898	1.331705216
DCAF11	chr10	80560001	80710001	80404031	80412030	7.493252898	1.331705216
FITM1	chr10	80560001	80710001	80413784	80416061	7.493252898	1.331705216
PSME1	chr10	80560001	80710001	80418160	80420956	7.493252898	1.331705216
EMC9	chr10	80560001	80710001	80420953	80423346	7.493252898	1.331705216
PSME2	chr10	80560001	80710001	80425928	80429833	7.493252898	1.331705216
RNF31	chr10	80560001	80710001	80429936	80440984	7.493252898	1.331705216
IRF9	chr10	80560001	80710001	80441154	80446438	7.493252898	1.331705216
REC8	chr10	80560001	80710001	80450920	80456724	7.493252898	1.331705216
IPO4	chr10	80560001	80710001	80456694	80465666	7.493252898	1.331705216
TM9SF1	chr10	80560001	80710001	80465618	80471145	7.493252898	1.331705216
TSSK4	chr10	80560001	80710001	80471219	80481243	7.493252898	1.331705216
CHMP4A	chr10	80560001	80710001	80481346	80485099	7.493252898	1.331705216
NEDD8	chr10	80560001	80710001	80487701	80496437	7.493252898	1.331705216
GMPR2	chr10	80560001	80710001	80496352	80502962	7.493252898	1.331705216
TINF2	chr10	80560001	80710001	80500910	80506489	7.493252898	1.331705216
TGM1	chr10	80560001	80710001	80514652	80529096	7.493252898	1.331705216
RABGGTA	chr10	80560001	80710001	80531344	80537374	7.493252898	1.331705216

DHRS1	chr10	80560001	80710001	80553019	80561411	7.493252898	1.331705216
NOP9	chr10	80560001	80710001	80561504	80567378	7.493252898	1.331705216
CIDEB	chr10	80560001	80710001	80567352	80573419	7.493252898	1.331705216
LTB4R2	chr10	80560001	80710001	80569575	80576366	7.493252898	1.331705216
LTB4R	chr10	80560001	80710001	80572744	80580288	7.493252898	1.331705216
ADCY4	chr10	80560001	80710001	80579805	80595382	7.493252898	1.331705216
RIPK3	chr10	80560001	80710001	80596354	80600577	7.493252898	1.331705216
NFATC4	chr10	80560001	80710001	80625155	80634456	7.493252898	1.331705216
NYNRIN	chr10	80560001	80710001	80658795	80680140	7.493252898	1.331705216
CBLN3	chr10	80560001	80710001	80681703	80691147	7.493252898	1.331705216
KHNYN	chr10	80560001	80710001	80683758	80701406	7.493252898	1.331705216
SDR39U1	chr10	80560001	80710001	80701422	80704767	7.493252898	1.331705216
TBC1D21	chr10	80560001	80710001	80730793	80744577	7.493252898	1.331705216
C10H15orf59	chr10	80560001	80710001	80874053	80887118	7.493252898	1.331705216
CD276	chr10	80560001	80710001	80911808	80946803	7.493252898	1.331705216
NPTN	chr10	80560001	80710001	80995331	81061660	7.493252898	1.331705216
REC114	chr10	80560001	80710001	81061570	81170567	7.493252898	1.331705216
ARIH1	chr10	82460001	82610001	81939607	82041082	2.533725521	1.098284796
HEXA	chr10	82460001	82610001	82112311	82143840	2.533725521	1.098284796
CELF6	chr10	82460001	82610001	82164246	82194306	2.533725521	1.098284796
PARP6	chr10	82460001	82610001	82205875	82232562	2.533725521	1.098284796
PKM	chr10	82460001	82610001	82238790	82265545	2.533725521	1.098284796
GRAMD2	chr10	82460001	82610001	82266642	82301212	2.533725521	1.098284796
SENP8	chr10	82460001	82610001	82315289	82335360	2.533725521	1.098284796
MYO9A	chr10	82460001	82610001	82335575	82577050	2.533725521	1.098284796
NR2E3	chr10	82460001	82610001	82590036	82594865	2.533725521	1.098284796
THSD4	chr10	82460001	82610001	82620551	83285979	2.533725521	1.098284796
SV2C	chr10	94290001	94440001	93649456	93866174	7.246893935	1.366699889
POC5	chr10	94340001	94490001	94124060	94156199	10.12898614	2.22891102
ANKDD1B	chr10	94340001	94490001	94157042	94234857	10.12898614	2.22891102
POLK	chr10	94340001	94490001	94240022	94323047	10.12898614	2.22891102
COL4A3BP	chr10	94340001	94490001	94322997	94463887	10.12898614	2.22891102
HMGCR	chr10	94340001	94490001	94469555	94491458	10.12898614	2.22891102
ANKRD31	chr10	94340001	94490001	94562310	94696787	10.12898614	2.22891102
GCNT4	chr10	94340001	94490001	94707551	94736446	10.12898614	2.22891102
MEMO1	chr11	14920001	15070001	14404200	14515722	5.078261916	1.631756201
DPY30	chr11	14920001	15070001	14524697	14538756	5.078261916	1.631756201
SPAST	chr11	14920001	15070001	14556017	14608600	5.078261916	1.631756201
SLC30A6	chr11	14920001	15070001	14620539	14666163	5.078261916	1.631756201
NLRC4	chr11	14920001	15070001	14669557	14716302	5.078261916	1.631756201
YIPF4	chr11	14920001	15070001	14738946	14782522	5.078261916	1.631756201
BIRC6	chr11	14920001	15070001	14814336	15024500	5.078261916	1.631756201
TTC27	chr11	14920001	15070001	15041424	15219539	5.078261916	1.631756201
LTBP1	chr11	14920001	15070001	15324388	15780520	5.078261916	1.631756201
CRIM1	chr11	18120001	18270001	18670767	18881417	2.998310964	-1.01187887
CRIPT	chr11	29080001	29230001	28648447	28659797	2.98348829	-0.937878288
SOCS5	chr11	29090001	29240001	28714825	28802754	3.20015091	-0.918115033
MCFD2	chr11	29090001	29240001	28943551	28953070	3.20015091	-0.918115033
TTC7A	chr11	29090001	29240001	28986941	29103619	3.20015091	-0.918115033
TRNAE-UUC	chrX1	46010001	46160001	46250912	46250984	5.493789747	1.676718744
C11H2orf61	chr11	29090001	29240001	29117484	29152416	3.20015091	-0.918115033
CALM2	chr11	29090001	29240001	29156107	29169684	3.20015091	-0.918115033
EPCAM	chr11	29090001	29240001	29347274	29359525	3.20015091	-0.918115033
MSH2	chr11	29090001	29240001	29370644	29455779	3.20015091	-0.918115033
KCNK12	chr11	29090001	29240001	29483068	29533843	3.20015091	-0.918115033
PLB1	chr11	71250001	71400001	70867451	71000091	3.372787468	1.157496503
FOSL2	chr11	71250001	71400001	71072128	71095051	3.372787468	1.157496503
BRE	chr11	71250001	71400001	71147448	71550180	3.372787468	1.157496503
TRNAC-GCA	chr16	62530001	62680001	62935749	62935820	6.204615165	-0.939260842
RBKS	chr11	71250001	71400001	71550250	71661409	3.372787468	1.157496503
MRPL33	chr11	71250001	71400001	71652999	71662442	3.372787468	1.157496503
SLC4A1AP	chr11	71250001	71400001	71732125	71753507	3.372787468	1.157496503
SUPT7L	chr11	71250001	71400001	71753582	71766351	3.372787468	1.157496503
GPN1	chr11	71250001	71400001	71768498	71796072	3.372787468	1.157496503
CCDC121	chr11	71250001	71400001	71796097	71799329	3.372787468	1.157496503
ZNF512	chr11	71250001	71400001	71803917	71833136	3.372787468	1.157496503
RFXAP	chr12	62300001	62450001	61869600	61896068	2.550401029	-0.988504361
SMAD9	chr12	62300001	62450001	61903330	61966268	2.550401029	-0.988504361
ALG5	chr12	62300001	62450001	61974895	62002230	2.550401029	-0.988504361
EXOSC8	chr12	62300001	62450001	62003540	62011362	2.550401029	-0.988504361
SUPT20H	chr12	62300001	62450001	62011274	62047300	2.550401029	-0.988504361

POSTN	chr12	62300001	62450001	62423932	62458253	2.550401029	-0.988504361
TRPC4	chr12	62300001	62450001	62497288	62709093	2.550401029	-0.988504361
RASSF2	chr13	46960001	47110001	46544095	46569158	2.603470282	-0.905899084
SLC23A2	chr13	47190001	47340001	46633841	46772996	2.676121784	-1.443006615
TMEM230	chr13	47210001	47360001	46856288	46864262	4.413791352	-1.506762581
PCNA	chr13	47210001	47360001	46864800	46870372	4.413791352	-1.506762581
CDS2	chr13	47210001	47360001	46876073	46921625	4.413791352	-1.506762581
PROKR2	chr13	47210001	47360001	46991225	47003919	4.413791352	-1.506762581
GPCPD1	chr13	47210001	47360001	47213879	47282392	4.413791352	-1.506762581
C13H20orf196	chr13	47210001	47360001	47418863	47527032	4.413791352	-1.506762581
CHGB	chr13	47210001	47360001	47569978	47587414	4.413791352	-1.506762581
TRMT6	chr13	47210001	47360001	47615544	47627266	4.413791352	-1.506762581
MCM8	chr13	47210001	47360001	47627290	47684838	4.413791352	-1.506762581
CRLS1	chr13	47210001	47360001	47713968	47736274	4.413791352	-1.506762581
LRRN4	chr13	47210001	47360001	47739527	47755422	4.413791352	-1.506762581
FERMT1	chr13	47210001	47360001	47778574	47837681	4.413791352	-1.506762581
HAO1	chr13	50420001	50570001	50024601	50086221	3.552393402	-1.493296513
ADRA1D	chr13	50420001	50570001	50264597	50293882	3.552393402	-1.493296513
SMOX	chr13	50420001	50570001	50305402	50340974	3.552393402	-1.493296513
RNF24	chr13	50420001	50570001	50520829	50637289	3.552393402	-1.493296513
TRNAE-UUC	chrX1	46010001	46160001	46250912	46250984	5.493789747	1.676718744
PANK2	chr13	50420001	50570001	50640389	50669701	3.552393402	-1.493296513
MIR103	chr13	50420001	50570001	50646831	50646916	3.552393402	-1.493296513
MAVS	chr13	50420001	50570001	50694748	50707107	3.552393402	-1.493296513
AP5S1	chr13	50420001	50570001	50734433	50738294	3.552393402	-1.493296513
CDC25B	chr13	50420001	50570001	50751016	50761010	3.552393402	-1.493296513
CENPB	chr13	50420001	50570001	50772192	50775019	3.552393402	-1.493296513
SPEF1	chr13	50420001	50570001	50775106	50781210	3.552393402	-1.493296513
C13H20orf27	chr13	50420001	50570001	50785603	50799453	3.552393402	-1.493296513
HSPA12B	chr13	50420001	50570001	50799840	50816831	3.552393402	-1.493296513
SIGLEC1	chr13	50420001	50570001	50842091	50860436	3.552393402	-1.493296513
ADAM33	chr13	50420001	50570001	50865505	50879346	3.552393402	-1.493296513
GFRA4	chr13	50420001	50570001	50882036	50886744	3.552393402	-1.493296513
ATRN	chr13	50420001	50570001	50894660	51079673	3.552393402	-1.493296513
C13H20orf194	chr13	50730001	50880001	51139649	51312804	2.534728431	-1.451662024
TRNAS-GGA	chr4	52950001	53100001	53090579	53090650	4.326759136	-0.932085986
RAE1	chr13	58510001	58660001	58079192	58097238	2.699562687	-0.934014171
SPO11	chr13	58510001	58660001	58104357	58119153	2.699562687	-0.934014171
BMP7	chr13	58510001	58660001	58174093	58260125	2.699562687	-0.934014171
TFAP2C	chr13	58970001	59120001	58657523	58667140	2.989182781	-2.602963759
RTFDC1	chr13	58970001	59120001	58735902	58783189	2.989182781	-2.602963759
GCNT7	chr13	58970001	59120001	58749848	58761271	2.989182781	-2.602963759
CASS4	chr13	58970001	59120001	58791766	58829365	2.989182781	-2.602963759
CSTF1	chr13	58970001	59120001	58833053	58845722	2.989182781	-2.602963759
AURKA	chr13	58970001	59120001	58845806	58862526	2.989182781	-2.602963759
FAM210B	chr13	58970001	59120001	58864336	58875132	2.989182781	-2.602963759
MC3R	chr13	59350001	59500001	58935095	58936185	2.998191975	-1.006651708
NSFL1C	chr13	59350001	59500001	59000052	59021299	2.998191975	-1.006651708
FKBP1A	chr13	59350001	59500001	59038273	59061754	2.998191975	-1.006651708
SDCBP2	chr13	59350001	59500001	59090093	59109110	2.998191975	-1.006651708
SNPH	chr13	59350001	59500001	59109662	59153612	2.998191975	-1.006651708
RAD21L1	chr13	59350001	59500001	59165671	59201265	2.998191975	-1.006651708
C13H20orf202	chr13	59350001	59500001	59230537	59243636	2.998191975	-1.006651708
TMEM74B	chr13	59350001	59500001	59244717	59249746	2.998191975	-1.006651708
PSMF1	chr13	59350001	59500001	59259919	59302429	2.998191975	-1.006651708
RSPO4	chr13	59350001	59500001	59419545	59456637	2.998191975	-1.006651708
ANGPT4	chr13	59350001	59500001	59493311	59543214	2.998191975	-1.006651708
FAM110A	chr13	59350001	59500001	59563554	59575513	2.998191975	-1.006651708
SLC52A3	chr13	59350001	59500001	59592813	59611003	2.998191975	-1.006651708
SCRT2	chr13	59350001	59500001	59693358	59707360	2.998191975	-1.006651708
SRXN1	chr13	59350001	59500001	59717581	59724941	2.998191975	-1.006651708
TCF15	chr13	59350001	59500001	59766065	59772269	2.998191975	-1.006651708
CSNK2A1	chr13	59350001	59500001	59825086	59880670	2.998191975	-1.006651708
TBC1D20	chr13	59350001	59500001	59896329	59914114	2.998191975	-1.006651708
RBCK1	chr13	59350001	59500001	59918875	59937057	2.998191975	-1.006651708
COMMD7	chr13	61860001	62010001	61430474	61455479	2.550622009	-1.20889443
DNMT3B	chr13	61860001	62010001	61467790	61502090	2.550622009	-1.20889443
MAPRE1	chr13	61860001	62010001	61508458	61541861	2.550622009	-1.20889443
SUN5	chr13	61860001	62010001	61602832	61626958	2.550622009	-1.20889443
BPIFB2	chr13	61860001	62010001	61632481	61650156	2.550622009	-1.20889443
BPIFB6	chr13	61860001	62010001	61657835	61672584	2.550622009	-1.20889443

BPIFB3	chr13	61860001	62010001	61681607	61696489	2.550622009	-1.20889443
BPIFB4	chr13	61860001	62010001	61704744	61730572	2.550622009	-1.20889443
BPIFA2	chr13	61860001	62010001	61962034	61971556	2.550622009	-1.20889443
BPIFA3	chr13	62530001	62680001	62295723	62306454	3.757446061	-2.189680297
BPIFA1	chr13	62530001	62680001	62313752	62321102	3.757446061	-2.189680297
BPIFB1	chr13	62530001	62680001	62330853	62371128	3.757446061	-2.189680297
CDK5RAP1	chr13	62530001	62680001	62434448	62470312	3.757446061	-2.189680297
SNTA1	chr13	62930001	63080001	62475263	62505018	4.836832511	-0.979373349
CBFA2T2	chr13	62930001	63080001	62545241	62683536	4.836832511	-0.979373349
NECAB3	chr13	62930001	63080001	62691011	62709617	4.836832511	-0.979373349
C13H20orf144	chr13	62930001	63080001	62697382	62699145	4.836832511	-0.979373349
ACTL10	chr13	62930001	63080001	62702196	62703536	4.836832511	-0.979373349
E2F1	chr13	62930001	63080001	62710990	62720515	4.836832511	-0.979373349
PXMP4	chr13	62930001	63080001	62730702	62747008	4.836832511	-0.979373349
ZNF341	chr13	62930001	63080001	62761999	62803765	4.836832511	-0.979373349
CHMP4B	chr13	62930001	63080001	62814614	62861236	4.836832511	-0.979373349
TRNAG-UCC	chr13	62930001	63080001	62912627	62912699	4.836832511	-0.979373349
**RALY**	chr13	62930001	63080001	62969108	63053806	4.836832511	-0.979373349
EIF2S2	chr13	62930001	63080001	63062854	63082442	4.836832511	-0.979373349
**ASIP**	chr13	62930001	63080001	63228709	63249542	4.836832511	-0.979373349
AHCY	chr13	62930001	63080001	63264059	63279821	4.836832511	-0.979373349
ITCH	chr13	62930001	63080001	63363104	63462630	4.836832511	-0.979373349
DYNLRB1	chr13	62930001	63080001	63465549	63483202	4.836832511	-0.979373349
MAP1LC3A	chr13	62930001	63080001	63498937	63500710	4.836832511	-0.979373349
PIGU	chr13	62930001	63080001	63500918	63594821	4.836832511	-0.979373349
MAFB	chr13	69200001	69350001	68957927	68961227	2.69527907	-1.363270987
TRNAW-CCA	chrX1	14400001	14550001	14663110	14663181	4.407790888	1.145351386
TOP1	chr13	69200001	69350001	69270713	69355431	2.69527907	-1.363270987
PLCG1	chr13	69200001	69350001	69366691	69399774	2.69527907	-1.363270987
ZHX3	chr13	69200001	69350001	69402826	69515922	2.69527907	-1.363270987
LPIN3	chr13	69200001	69350001	69534894	69553728	2.69527907	-1.363270987
EMILIN3	chr13	69200001	69350001	69553350	69559361	2.69527907	-1.363270987
TRNAE-CUC	chr13	69200001	69350001	69557976	69558047	2.69527907	-1.363270987
CHD6	chr13	69200001	69350001	69592893	69796343	2.69527907	-1.363270987
PTPRT	chr13	70370001	70520001	70261033	71417055	3.304130609	-2.01100636
TRNAC-GCA	chr16	62530001	62680001	62935749	62935820	6.204615165	-0.939260842
CBLN4	chr13	82650001	82800001	82836781	82844508	2.753447867	-0.964094616
MTDH	chr14	16030001	16180001	15663731	15721499	4.37579975	2.015176396
LAPTM4B	chr14	16030001	16180001	15788488	15854656	4.37579975	2.015176396
MATN2	chr14	16030001	16180001	15872116	16041603	4.37579975	2.015176396
RPL30	chr14	16470001	16620001	16048416	16051780	5.046304774	1.003314389
ERICH5	chr14	16500001	16650001	16063489	16086802	6.006871853	1.186563944
RIDA	chr14	16500001	16650001	16096003	16104730	6.006871853	1.186563944
POP1	chr14	16500001	16650001	16104883	16139688	6.006871853	1.186563944
NIPAL2	chr14	16500001	16650001	16173131	16256193	6.006871853	1.186563944
KCNS2	chr14	16500001	16650001	16394213	16400041	6.006871853	1.186563944
STK3	chr14	16500001	16650001	16433649	16744347	6.006871853	1.186563944
OSR2	chr14	16500001	16650001	16881475	16889328	6.006871853	1.186563944
VPS13B	chr14	16500001	16650001	16964332	17751028	6.006871853	1.186563944
TOX	chr14	57110001	57260001	56824099	57133493	3.412733895	-1.543298969
TRNAC-GCA	chr16	62530001	62680001	62935749	62935820	6.204615165	-0.939260842
NSMAF	chr14	57110001	57260001	57251301	57317215	3.412733895	-1.543298969
SDCBP	chr14	57110001	57260001	57317726	57350168	3.412733895	-1.543298969
UBXN2B	chr14	57110001	57260001	57451412	57481003	3.412733895	-1.543298969
FAM110B	chr14	57110001	57260001	57631544	57776493	3.412733895	-1.543298969
CLPB	chr15	31920001	32070001	31410696	31553755	3.401225924	1.743385708
PHOX2A	chr15	31920001	32070001	31580744	31585333	3.401225924	1.743385708
INPPL1	chr15	31920001	32070001	31585544	31600551	3.401225924	1.743385708
FOLR2	chr15	31920001	32070001	31602384	31605922	3.401225924	1.743385708
TRNAG-CCC	chrX2	10370001	10520001	10239324	10239396	3.767169191	1.355072662
FOLR1	chr15	31920001	32070001	31622904	31634159	3.401225924	1.743385708
ANAPC15	chr15	31920001	32070001	31688212	31691470	3.401225924	1.743385708
LAMTOR1	chr15	31920001	32070001	31697022	31703528	3.401225924	1.743385708
NUMA1	chr15	31920001	32070001	31718205	31787884	3.401225924	1.743385708
IL18BP	chr15	31920001	32070001	31788381	31790497	3.401225924	1.743385708
RNF121	chr15	31920001	32070001	31788430	31874308	3.401225924	1.743385708
ART5	chr15	31920001	32070001	31912716	31916296	3.401225924	1.743385708
ART1	chr15	31920001	32070001	31961266	31972443	3.401225924	1.743385708
NUP98	chr15	31920001	32070001	31985255	32073364	3.401225924	1.743385708
PGAP2	chr15	31920001	32070001	32081071	32094586	3.401225924	1.743385708
RHOG	chr15	31920001	32070001	32095244	32106242	3.401225924	1.743385708

STIM1	chr15	31920001	32070001	32117471	32313714	3.401225924	1.743385708
RRM1	chr15	31920001	32070001	32315709	32355581	3.401225924	1.743385708
TRNAG-CCC	chrX2	10370001	10520001	10239324	10239396	3.767169191	1.355072662
DUSP10	chr16	24020001	24170001	23718507	23758962	2.775749872	-1.027380861
TRNAT-UGU	chr16	24020001	24170001	24513444	24513516	2.775749872	-1.027380861
HHIPL2	chr16	24020001	24170001	24518158	24545971	2.775749872	-1.027380861
TAF1A	chr16	24020001	24170001	24552297	24583583	2.775749872	-1.027380861
CAPN2	chr16	26060001	26210001	25579186	25635659	2.854639815	-0.994240731
TP53BP2	chr16	26060001	26210001	25637185	25704284	2.854639815	-0.994240731
FBXO28	chr16	26160001	26310001	25797222	25826231	2.887990832	-1.367359524
DEGS1	chr16	26160001	26310001	25857736	25867324	2.887990832	-1.367359524
NVL	chr16	26160001	26310001	25907863	25991438	2.887990832	-1.367359524
CNIH4	chr16	26160001	26310001	26021184	26039178	2.887990832	-1.367359524
WDR26	chr16	26160001	26310001	26044756	26085641	2.887990832	-1.367359524
CNIH3	chr16	26160001	26310001	26233683	26368158	2.887990832	-1.367359524
TRNAD-GUC	chr16	26160001	26310001	26576818	26576889	2.887990832	-1.367359524
DNAH14	chr16	26160001	26310001	26600001	26998456	2.887990832	-1.367359524
ZBTB18	chr16	31960001	32110001	31676613	31685187	3.396534344	1.737427183
AKT3	chr16	31960001	32110001	31877151	32159430	3.396534344	1.737427183
SDCCAG8	chr16	31960001	32110001	32158344	32401912	3.396534344	1.737427183
CEP170	chr16	31960001	32110001	32402081	32538214	3.396534344	1.737427183
PLD5	chr16	33630001	33780001	32961659	33377109	4.25271376	1.306320576
BECN2	chr16	33630001	33780001	33433023	33434318	4.25271376	1.306320576
MAP1LC3C	chr16	33630001	33780001	33461574	33467259	4.25271376	1.306320576
EXO1	chr16	33630001	33780001	33479386	33524373	4.25271376	1.306320576
WDR64	chr16	33630001	33780001	33534070	33669850	4.25271376	1.306320576
CHML	chr16	33630001	33780001	33677364	33686223	4.25271376	1.306320576
OPN3	chr16	33630001	33780001	33707072	33715141	4.25271376	1.306320576
KMO	chr16	33630001	33780001	33714087	33781937	4.25271376	1.306320576
FH	chr16	33630001	33780001	33797989	33822750	4.25271376	1.306320576
RGS7	chr16	33630001	33780001	33938680	34410674	4.25271376	1.306320576
UBE4B	chr16	42420001	42570001	41875171	41997226	3.435239882	-1.080698059
RBP7	chr16	42420001	42570001	42034222	42057361	3.435239882	-1.080698059
NMNAT1	chr16	42420001	42570001	42070896	42099070	3.435239882	-1.080698059
LZIC	chr16	42420001	42570001	42098641	42108160	3.435239882	-1.080698059
CTNNBIP1	chr16	42420001	42570001	42125452	42172257	3.435239882	-1.080698059
CLSTN1	chr16	42420001	42570001	42200194	42276330	3.435239882	-1.080698059
PIK3CD	chr16	42420001	42570001	42276030	42337774	3.435239882	-1.080698059
TMEM201	chr16	42420001	42570001	42378250	42404636	3.435239882	-1.080698059
SLC25A33	chr16	42420001	42570001	42409266	42441068	3.435239882	-1.080698059
SPSB1	chr16	42420001	42570001	42602138	42672843	3.435239882	-1.080698059
H6PD	chr16	42420001	42570001	42698678	42738708	3.435239882	-1.080698059
MIR34A	chr16	42420001	42570001	42803786	42803892	3.435239882	-1.080698059
GPR157	chr16	42420001	42570001	42828376	42910835	3.435239882	-1.080698059
CA6	chr16	42430001	42580001	43004716	43034709	3.43022533	-1.053493701
GLUL	chr16	62530001	62680001	62094533	62105099	6.204615165	-0.939260842
TEDDM1	chr16	62530001	62680001	62112234	62113240	6.204615165	-0.939260842
RGSL1	chr16	62530001	62680001	62168698	62235170	6.204615165	-0.939260842
TRNAC-ACA	chr16	62530001	62680001	62246657	62246728	6.204615165	-0.939260842
RNASEL	chr16	62530001	62680001	62246769	62265717	6.204615165	-0.939260842
RGS16	chr16	62530001	62680001	62275493	62281296	6.204615165	-0.939260842
RGS8	chr16	62530001	62680001	62328361	62375835	6.204615165	-0.939260842
NPL	chr16	62530001	62680001	62472116	62514693	6.204615165	-0.939260842
DHX9	chr16	62530001	62680001	62524255	62569488	6.204615165	-0.939260842
SHCBP1L	chr16	62530001	62680001	62572702	62611679	6.204615165	-0.939260842
LAMC1	chr16	62530001	62680001	62678983	62800726	6.204615165	-0.939260842
LAMC2	chr16	62530001	62680001	62840547	62910467	6.204615165	-0.939260842
NMNAT2	chr16	62530001	62680001	62912702	63133011	6.204615165	-0.939260842
TRNAC-GCA	chr16	62530001	62680001	62935749	62935820	6.204615165	-0.939260842
NSL1	chr16	69880001	70030001	69415158	69456616	3.284191393	-0.924398915
BATF3	chr16	69900001	70050001	69498880	69512095	3.360106602	-0.925768606
FAM71A	chr16	69900001	70050001	69570276	69572494	3.360106602	-0.925768606
ATF3	chr16	69900001	70050001	69577756	69590608	3.360106602	-0.925768606
NENF	chr16	69900001	70050001	69779747	69787078	3.360106602	-0.925768606
TMEM206	chr16	69900001	70050001	69800710	69834107	3.360106602	-0.925768606
PPP2R5A	chr16	69900001	70050001	69835172	69902033	3.360106602	-0.925768606
DTL	chr16	69900001	70050001	70074829	70126246	3.360106602	-0.925768606
INTS7	chr16	69900001	70050001	70126144	70205853	3.360106602	-0.925768606
LPGAT1	chr16	69900001	70050001	70221573	70376075	3.360106602	-0.925768606
NEK2	chr16	69900001	70050001	70429263	70441739	3.360106602	-0.925768606
HHAT	chr16	72040001	72190001	71271943	71639497	3.90837556	-0.930160375

TRNAC-GCA	chr16	62530001	62680001	62935749	62935820	6.204615165	-0.939260842
SERTAD4	chr16	72070001	72220001	71719132	71733081	4.251727848	-0.917297411
SYT14	chr16	72070001	72220001	71798086	71934364	4.251727848	-0.917297411
DIEXF	chr16	72070001	72220001	72072998	72102018	4.251727848	-0.917297411
IRF6	chr16	72070001	72220001	72127836	72144263	4.251727848	-0.917297411
C16H1orf74	chr16	72070001	72220001	72147939	72150096	4.251727848	-0.917297411
TRAF3IP3	chr16	72070001	72220001	72150101	72173649	4.251727848	-0.917297411
HSD11B1	chr16	72070001	72220001	72192175	72232663	4.251727848	-0.917297411
G0S2	chr16	72070001	72220001	72264418	72265385	4.251727848	-0.917297411
LAMB3	chr16	72070001	72220001	72301952	72344457	4.251727848	-0.917297411
CAMK1G	chr16	72070001	72220001	72345416	72378212	4.251727848	-0.917297411
NEK7	chr16	75970001	76120001	75639146	75776800	2.644453616	-1.015957574
ATP6V1G3	chr16	75970001	76120001	75974525	75995529	2.644453616	-1.015957574
PTPRC	chr16	75970001	76120001	76103209	76232274	2.644453616	-1.015957574
PAFAH1B1	chr19	23760001	23910001	23302330	23368013	3.059012535	-1.238544507
CLUH	chr19	23760001	23910001	23373646	23395027	3.059012535	-1.238544507
RAP1GAP2	chr19	23760001	23910001	23437609	23652991	3.059012535	-1.238544507
SPATA22	chr19	23760001	23910001	23998431	24013174	3.059012535	-1.238544507
ASPA	chr19	23760001	23910001	24018930	24037078	3.059012535	-1.238544507
TRPV3	chr19	23760001	23910001	24042198	24076976	3.059012535	-1.238544507
TRPV1	chr19	23760001	23910001	24082035	24111002	3.059012535	-1.238544507
SHPK	chr19	23760001	23910001	24128599	24147165	3.059012535	-1.238544507
CTNS	chr19	23760001	23910001	24147314	24166212	3.059012535	-1.238544507
TAX1BP3	chr19	23760001	23910001	24166707	24172070	3.059012535	-1.238544507
EMC6	chr19	23760001	23910001	24171946	24173008	3.059012535	-1.238544507
P2RX5	chr19	23760001	23910001	24175226	24191058	3.059012535	-1.238544507
ITGAE	chr19	23760001	23910001	24198191	24258647	3.059012535	-1.238544507
GSG2	chr19	23760001	23910001	24204550	24207767	3.059012535	-1.238544507
NCBP3	chr19	23760001	23910001	24268865	24294295	3.059012535	-1.238544507
CAMKK1	chr19	23760001	23910001	24299517	24329180	3.059012535	-1.238544507
P2RX1	chr19	23760001	23910001	24333541	24352309	3.059012535	-1.238544507
FAM134B	chr20	56960001	57110001	56450873	56607068	4.249297065	-2.184424571
ZNF622	chr20	56960001	57110001	56614034	56627946	4.249297065	-2.184424571
11-Mar	chr20	56960001	57110001	56903699	57019253	4.249297065	-2.184424571
FBXL7	chr20	56960001	57110001	57170615	57623887	4.249297065	-2.184424571
ICE1	chr20	68100001	68250001	67619014	67689571	2.617341041	-1.236163541
ADAMTS16	chr20	68100001	68250001	67786480	67969514	2.617341041	-1.236163541
TRNAS-GGA	chr4	52950001	53100001	53090579	53090650	4.326759136	-0.932085986
IRX4	chr20	70920001	71070001	70764248	70772443	3.552427399	-0.963250524
NDUFS6	chr20	70920001	71070001	70826012	70831065	3.552427399	-0.963250524
MRPL36	chr20	70920001	71070001	70832013	70833718	3.552427399	-0.963250524
LPCAT1	chr20	70920001	71070001	70963369	71000848	3.552427399	-0.963250524
SLC6A3	chr20	70920001	71070001	71014677	71048556	3.552427399	-0.963250524
SLC12A7	chr20	70920001	71070001	71096461	71133473	3.552427399	-0.963250524
NKD2	chr20	70920001	71070001	71137228	71158931	3.552427399	-0.963250524
TRIP13	chr20	70920001	71070001	71206857	71222292	3.552427399	-0.963250524
BRD9	chr20	70920001	71070001	71222238	71240459	3.552427399	-0.963250524
TERT	chr20	70920001	71070001	71256520	71274686	3.552427399	-0.963250524
SLC6A18	chr20	70920001	71070001	71278518	71293544	3.552427399	-0.963250524
SLC6A19	chr20	70920001	71070001	71295262	71311801	3.552427399	-0.963250524
TRNAS-GGA	chr4	52950001	53100001	53090579	53090650	4.326759136	-0.932085986
TPPP	chr20	70920001	71070001	71380831	71402958	3.552427399	-0.963250524
CEP72	chr20	70920001	71070001	71405220	71445196	3.552427399	-0.963250524
TOPAZ1	chr22	16500001	16650001	16017411	16080362	3.489091065	-2.116535582
TCAIM	chr22	16510001	16660001	16082199	16119825	3.559447771	-2.492484414
ZNF445	chr22	16510001	16660001	16140516	16168275	3.559447771	-2.492484414
ZNF852	chr22	16510001	16660001	16189711	16202009	3.559447771	-2.492484414
ZNF502	chr22	16510001	16660001	16287427	16297792	3.559447771	-2.492484414
ZNF501	chr22	16510001	16660001	16309551	16310546	3.559447771	-2.492484414
KIAA1143	chr22	16510001	16660001	16318453	16327891	3.559447771	-2.492484414
KIF15	chr22	16510001	16660001	16327926	16386693	3.559447771	-2.492484414
TATDN2	chr22	16510001	16660001	16453967	16476805	3.559447771	-2.492484414
IRAK2	chr22	16920001	17070001	16479062	16533738	3.660350746	-1.567040593
VHL	chr22	16920001	17070001	16540685	16547653	3.660350746	-1.567040593
BRK1	chr22	16920001	17070001	16552011	16559676	3.660350746	-1.567040593
FANCD2OS	chr22	16920001	17070001	16561690	16565016	3.660350746	-1.567040593
FANCD2	chr22	16920001	17070001	16565691	16614581	3.660350746	-1.567040593
EMC3	chr22	16920001	17070001	16614568	16629878	3.660350746	-1.567040593
PRRT3	chr22	16920001	17070001	16637743	16644601	3.660350746	-1.567040593
CRELD1	chr22	16920001	17070001	16645360	16653853	3.660350746	-1.567040593
IL17RC	chr22	16920001	17070001	16653881	16665409	3.660350746	-1.567040593

IL17RE	chr22	16920001	17070001	16665446	16676596	3.660350746	-1.567040593
JAGN1	chr22	16920001	17070001	16682058	16685009	3.660350746	-1.567040593
CIDEC	chr22	16920001	17070001	16688728	16698787	3.660350746	-1.567040593
RPUSD3	chr22	16920001	17070001	16704276	16713792	3.660350746	-1.567040593
ARPC4	chr22	16920001	17070001	16739519	16749345	3.660350746	-1.567040593
TADA3	chr22	16920001	17070001	16749506	16760007	3.660350746	-1.567040593
CAMK1	chr22	16920001	17070001	16770761	16781794	3.660350746	-1.567040593
OGG1	chr22	16920001	17070001	16781738	16787900	3.660350746	-1.567040593
BRPF1	chr22	16920001	17070001	16788964	16804596	3.660350746	-1.567040593
CPNE9	chr22	16920001	17070001	16806646	16826679	3.660350746	-1.567040593
MTMR14	chr22	16920001	17070001	16827861	16871844	3.660350746	-1.567040593
LHFPL4	chr22	16920001	17070001	16905086	16938585	3.660350746	-1.567040593
SETD5	chr22	17430001	17580001	16950600	17030658	5.631137462	1.553606257
THUMPD3	chr22	17430001	17580001	17048771	17070472	5.631137462	1.553606257
SRGAP3	chr22	17430001	17580001	17156766	17404110	5.631137462	1.553606257
RAD18	chr22	17430001	17580001	17414359	17523990	5.631137462	1.553606257
OXTR	chr22	17430001	17580001	17594776	17607958	5.631137462	1.553606257
CAV3	chr22	17430001	17580001	17610114	17622648	5.631137462	1.553606257
SSUH2	chr22	17430001	17580001	17670175	17692990	5.631137462	1.553606257
LMCD1	chr22	17430001	17580001	17740028	17796768	5.631137462	1.553606257
EXOC2	chr23	680001	830001	237311	359937	2.901810596	-1.157463595
FOXQ1	chr23	680001	830001	641272	643823	2.901810596	-1.157463595
FOXF2	chr23	680001	830001	691668	696627	2.901810596	-1.157463595
FOXC1	chr23	680001	830001	780584	784092	2.901810596	-1.157463595
GMDS	chr23	680001	830001	792203	1217301	2.901810596	-1.157463595
RUNX2	chr23	30210001	30360001	29926106	30269961	3.097786066	1.272978695
TRNAE-UUC	chrX1	46010001	46160001	46250912	46250984	5.493789747	1.676718744
SUPT3H	chr23	30210001	30360001	30223937	30622509	3.097786066	1.272978695
C24H18orf25	chr24	46580001	46730001	46164903	46247071	3.014833486	-0.91893312
RNF165	chr24	46580001	46730001	46308979	46417069	3.014833486	-0.91893312
LOXHD1	chr24	46580001	46730001	46431005	46629483	3.014833486	-0.91893312
ST8SIA5	chr24	46580001	46730001	46648415	46718152	3.014833486	-0.91893312
PIAS2	chr24	46580001	46730001	46753797	46854848	3.014833486	-0.91893312
KATNAL2	chr24	46580001	46730001	46946336	46993318	3.014833486	-0.91893312
HDHD2	chr24	46580001	46730001	46998128	47043825	3.014833486	-0.91893312
IER3IP1	chr24	46580001	46730001	47046744	47064735	3.014833486	-0.91893312
SKOR2	chr24	46580001	46730001	47100548	47134783	3.014833486	-0.91893312
C25H16orf45	chr25	14210001	14360001	13735033	13881095	3.77634837	-1.064313965
KIAA0430	chr25	14210001	14360001	13900354	13940070	3.77634837	-1.064313965
NDE1	chr25	14210001	14360001	13944989	13981278	3.77634837	-1.064313965
MYH11	chr25	14210001	14360001	13981844	14109280	3.77634837	-1.064313965
FOPNL	chr25	14210001	14360001	14115214	14134357	3.77634837	-1.064313965
TRNAC-GCA	chr16	62530001	62680001	62935749	62935820	6.204615165	-0.939260842
ABCC1	chr25	14210001	14360001	14181754	14337412	3.77634837	-1.064313965
ABCC6	chr25	14210001	14360001	14342003	14407612	3.77634837	-1.064313965
PLPP4	chr26	11740001	11890001	11346975	11490960	2.687748744	-1.079173173
SEC23IP	chr26	11740001	11890001	11949606	11988858	2.687748744	-1.079173173
MCMBP	chr26	11740001	11890001	12003875	12042329	2.687748744	-1.079173173
INPP5F	chr26	11740001	11890001	12042910	12135028	2.687748744	-1.079173173
BAG3	chr26	11740001	11890001	12206500	12229905	2.687748744	-1.079173173
TIAL1	chr26	11740001	11890001	12276234	12298428	2.687748744	-1.079173173
TRNAC-ACA	chr16	62530001	62680001	62246657	62246728	6.204615165	-0.939260842
RGS10	chr26	11750001	11900001	12317355	12357120	2.61288744	-1.142735555
MBL2	chr26	45100001	45250001	45038445	45045199	3.624466955	-1.006941609
C28H10orf128	chr28	2760001	2910001	2312335	2343429	3.101117768	-1.654263005
FAM170B	chr28	2760001	2910001	2361936	2364098	3.101117768	-1.654263005
VSTM4	chr28	2760001	2910001	2380016	2459503	3.101117768	-1.654263005
WDFY4	chr28	2760001	2910001	2478311	2732087	3.101117768	-1.654263005
LRRC18	chr28	2760001	2910001	2518800	2536516	3.101117768	-1.654263005
ARHGAP22	chr28	3350001	3500001	2751853	2933545	3.535955873	-1.740335816
MAPK8	chr28	3380001	3530001	2941175	3039589	3.726831799	-1.784383523
FRMPD2	chr28	3380001	3530001	3087917	3155034	3.726831799	-1.784383523
PTPN20	chr28	3380001	3530001	3207633	3233696	3.726831799	-1.784383523
GDF10	chr28	3380001	3530001	3460297	3473327	3.726831799	-1.784383523
GDF2	chr28	3380001	3530001	3482208	3486651	3.726831799	-1.784383523
RBP3	chr28	3380001	3530001	3494829	3504269	3.726831799	-1.784383523
ZNF488	chr28	3380001	3530001	3511701	3521545	3.726831799	-1.784383523
ANTXRL	chr28	3380001	3530001	3727694	3772919	3.726831799	-1.784383523
TRNAA-AGC	chr28	3380001	3530001	3823011	3823082	3.726831799	-1.784383523
GPRIN2	chr28	3380001	3530001	3917031	3935587	3.726831799	-1.784383523
FAM35A	chr28	3400001	3550001	3966919	4070554	3.512684954	-1.904008087

DEAF1	chr29	46220001	46370001	45775415	45801060	3.148203557	1.184661154
DRD4	chr29	46230001	46380001	45804141	45806873	3.819541506	1.429803402
SCT	chr29	46230001	46380001	45817070	45818263	3.819541506	1.429803402
CDHR5	chr29	46230001	46380001	45820504	45827415	3.819541506	1.429803402
IRF7	chr29	46230001	46380001	45828394	45831518	3.819541506	1.429803402
PHRF1	chr29	46230001	46380001	45832047	45856007	3.819541506	1.429803402
RASSF7	chr29	46230001	46380001	45864757	45867360	3.819541506	1.429803402
LMNTD2	chr29	46230001	46380001	45870525	45876726	3.819541506	1.429803402
LRRC56	chr29	46230001	46380001	45876806	45890449	3.819541506	1.429803402
HRAS	chr29	46230001	46380001	45894564	45897458	3.819541506	1.429803402
RNH1	chr29	46230001	46380001	45921210	45936419	3.819541506	1.429803402
PTDSS2	chr29	46230001	46380001	45937562	45962964	3.819541506	1.429803402
ANO9	chr29	46230001	46380001	45977907	45999636	3.819541506	1.429803402
SIGIRR	chr29	46230001	46380001	46000666	46009408	3.819541506	1.429803402
PKP3	chr29	46230001	46380001	46010418	46015001	3.819541506	1.429803402
PGGHG	chr29	46230001	46380001	46035921	46042088	3.819541506	1.429803402
IFITM5	chr29	46230001	46380001	46044242	46046440	3.819541506	1.429803402
B4GALNT4	chr29	46230001	46380001	46147705	46150925	3.819541506	1.429803402
AP2A2	chr29	46230001	46380001	46174668	46242919	3.819541506	1.429803402
MUC6	chr29	46230001	46380001	46244530	46267909	3.819541506	1.429803402
GPR152	chr29	46230001	46380001	46389008	46392442	3.819541506	1.429803402
TMEM134	chr29	46230001	46380001	46402781	46407781	3.819541506	1.429803402
AIP	chr29	46230001	46380001	46417047	46423260	3.819541506	1.429803402
PITPNM1	chr29	46230001	46380001	46423853	46437448	3.819541506	1.429803402
CDK2AP2	chr29	46230001	46380001	46438685	46441018	3.819541506	1.429803402
CABP2	chr29	46230001	46380001	46446866	46451638	3.819541506	1.429803402
NDUFV1	chr29	46230001	46380001	46509096	46514891	3.819541506	1.429803402
NUDT8	chr29	46230001	46380001	46522488	46524991	3.819541506	1.429803402
TBX10	chr29	46230001	46380001	46526481	46535231	3.819541506	1.429803402
UNC93B1	chr29	46230001	46380001	46561947	46572020	3.819541506	1.429803402
ALDH3B1	chr29	46230001	46380001	46581621	46601665	3.819541506	1.429803402
NDUFS8	chr29	46230001	46380001	46603414	46607673	3.819541506	1.429803402
TCIRG1	chr29	46230001	46380001	46610608	46622361	3.819541506	1.429803402
CHKA	chr29	46230001	46380001	46624210	46681700	3.819541506	1.429803402
KMT5B	chr29	46230001	46380001	46706234	46764579	3.819541506	1.429803402
C29H11orf24	chr29	46230001	46380001	46800409	46809400	3.819541506	1.429803402
DUSP8	chr29	51330001	51480001	50962017	50977940	9.794303073	4.144870298
MOB2	chr29	51330001	51480001	50987206	51039264	9.794303073	4.144870298
BRSK2	chr29	51330001	51480001	51047154	51097037	9.794303073	4.144870298
TOLLIP	chr29	51330001	51480001	51162053	51177156	9.794303073	4.144870298
MUC5AC	chr29	51330001	51480001	51246526	51276626	9.794303073	4.144870298
MUC2	chr29	51330001	51480001	51317872	51331926	9.794303073	4.144870298
EDA	chrX1	12290001	12440001	11557022	11895599	4.385284901	1.74118651
CDX4	chrX1	12290001	12440001	12054961	12063592	4.385284901	1.74118651
FAM155B	chrX1	12290001	12440001	12074820	12103927	4.385284901	1.74118651
PJA1	chrX1	12290001	12440001	12110252	12115011	4.385284901	1.74118651
STARD8	chrX1	12290001	12440001	12400560	12415129	4.385284901	1.74118651
YIPF6	chrX1	12290001	12440001	12585345	12617792	4.385284901	1.74118651
OPHN1	chrX1	12290001	12440001	12692459	13271064	4.385284901	1.74118651
AR	chrX1	13830001	13980001	13729623	13934065	8.570174569	3.5854313
MAGED1	chrX1	14400001	14550001	14646763	14730609	4.407790888	1.145351386
TRNAW-CCA	chrX1	14400001	14550001	14663110	14663181	4.407790888	1.145351386
GSPT2	chrX1	14400001	14550001	14796387	14798923	4.407790888	1.145351386
EDA2R	chrX1	18150001	18300001	18412588	18508471	11.90741806	6.779108666
PCYT1B	chrX1	45930001	46080001	45429192	45570963	4.994867375	1.847796518
PDK3	chrX1	46010001	46160001	45589783	45669513	5.493789747	1.676718744
ZFX	chrX1	46010001	46160001	45877351	45913094	5.493789747	1.676718744
EIF2S3	chrX1	46010001	46160001	45967962	45984248	5.493789747	1.676718744
KLHL15	chrX1	46010001	46160001	46006667	46039781	5.493789747	1.676718744
CXHXorf58	chrX1	46010001	46160001	46086883	46109876	5.493789747	1.676718744
APOO	chrX1	46010001	46160001	46109971	46177359	5.493789747	1.676718744
SAT1	chrX1	46010001	46160001	46220673	46223754	5.493789747	1.676718744
TRNAE-UUC	chrX1	46010001	46160001	46250912	46250984	5.493789747	1.676718744
ACOT9	chrX1	46010001	46160001	46265173	46295513	5.493789747	1.676718744
PRDX4	chrX1	46010001	46160001	46307957	46319355	5.493789747	1.676718744
TRNAC-ACA	chr16	62530001	62680001	62246657	62246728	6.204615165	-0.939260842
PTCHD1	chrX1	46020001	46170001	46585313	46636740	5.255709043	1.73508887
ARHGEF6	chrX2	10370001	10520001	10085939	10202183	3.767169191	1.355072662
CD40LG	chrX2	10370001	10520001	10210614	10224367	3.767169191	1.355072662
TRNAG-CCC	chrX2	10370001	10520001	10239324	10239396	3.767169191	1.355072662
VGLL1	chrX2	10370001	10520001	10330275	10338073	3.767169191	1.355072662

ADGRG4	chrX2	10370001	10520001	10378603	10476767	3.767169191	1.355072662
BRS3	chrX2	10370001	10520001	10550184	10554622	3.767169191	1.355072662
HTATSF1	chrX2	10370001	10520001	10558643	10575514	3.767169191	1.355072662
MAP7D3	chrX2	10370001	10520001	10596017	10629199	3.767169191	1.355072662
TRNAW-CCA	chrX1	14400001	14550001	14663110	14663181	4.407790888	1.145351386
MOSPD1	chrX2	10370001	10520001	10638690	10662600	3.767169191	1.355072662
TRNAC-GCA	chr16	62530001	62680001	62935749	62935820	6.204615165	-0.939260842
FAM122B	chrX2	10370001	10520001	10745390	10769280	3.767169191	1.355072662
PLAC1	chrX2	10370001	10520001	10913401	10950105	3.767169191	1.355072662
PHF6	chrX2	10410001	10560001	10959652	11007335	3.554280234	1.348459062
HPRT1	chrX2	10470001	10620001	11036061	11066425	3.251792291	1.091665988

Supplementary Table s5:The candidate genes in the selection sweep of white cashmere goats(IMC,LNC) and black cashmere goats(YNBB,GZB)

Gene	Chr	Bin_start	Bin_end	Gene_Start	Gene_end	ZFst	log 2 (θπ ratio)
**DLG1**	chr1	71190001	71340001	71247382	71513739	4.077795841	1.519133746
**MELTF**	chr1	71190001	71340001	71209817	71236775	4.077795841	1.519133746
**DSCR3**	chr1	149200001	149350001	149344317	149378412	7.13579866	1.220067334
**TTC3**	chr1	149200001	149350001	149193226	149320070	7.13579866	1.220067334
**MAP2**	chr2	38530001	38680001	38304673	38603165	4.585173759	1.44606391
**ABI2**	chr2	44180001	44330001	44270238	44375595	4.118960347	0.831762887
**RAPH1**	chr2	44180001	44330001	44165839	44269635	4.118960347	0.831762887
**ARHGAP15**	chr2	82980001	83130001	82654446	83365942	4.683102607	2.895711311
**TLK1**	chr2	110870001	111020001	110847293	111023597	3.682082085	0.896106212
**EIF2B3**	chr3	19630001	19780001	19591301	19712010	4.647552384	5.444823593
**HECTD3**	chr3	19630001	19780001	19724747	19732726	4.647552384	5.444823593
**UROD**	chr3	19630001	19780001	19735233	19738693	4.647552384	5.444823593
**ZSWIM5**	chr3	19630001	19780001	19738773	19797291	4.647552384	5.444823593
**MIER1**	chr3	42510001	42660001	42524645	42592531	6.301259252	0.695709022
**SLC35D1**	chr3	42510001	42660001	42606930	42654238	6.301259252	0.695709022
**WDR78**	chr3	42510001	42660001	42418935	42512922	6.301259252	0.695709022
**DLD**	chr4	71290001	71440001	71309867	71338255	5.85101858	0.689512446
**LAMB1**	chr4	71290001	71440001	71228918	71305310	5.85101858	0.689512446
**A4GALT**	chr5	112530001	112680001	112509158	112533604	4.411586744	1.438761099
**ARFGAP3**	chr5	112530001	112680001	112591791	112642935	4.411586744	1.438761099
**LOC10863607**	chr5	112530001	112680001	112564962	112571285	4.411586744	1.438761099
**PACSIN2**	chr5	112530001	112680001	112659474	112771854	4.411586744	1.438761099
**LIMCH1**	chr6	60700001	60850001	60794267	61154327	5.006638638	0.979381613
**UCHL1**	chr6	60700001	60850001	60715873	60727733	5.006638638	0.979381613
**KIT**	chr6	70650001	70800001	70711232	70793908	6.418703432	1.269078511
**LOC10216853**	chr6	85400001	85550001	85372781	85417336	3.79432632	0.983364532
**LOC10217567**	chr6	85400001	85550001	85418698	85462330	3.79432632	0.983364532
**LOC10863319**	chr6	85400001	85550001	85478280	85511521	3.79432632	0.983364532
**C6H4orf22**	chr6	95410001	95560001	95489192	96199327	7.88154538	-5.892952164
**FGF5**	chr6	95410001	95560001	95418992	95440124	7.88154538	-5.892952164
**LOC10218583**	chr7	670001	820001	784018	785066	3.542595109	1.869047665
**LOC10863641**	chr7	670001	820001	792204	793583	3.542595109	1.869047665
**CSF1R**	chr7	48970001	49120001	49005729	49037330	5.331314099	1.689495915
**HMGXB3**	chr7	48970001	49120001	49031724	49099624	5.331314099	1.689495915
**SLC26A2**	chr7	48970001	49120001	49103643	49133638	5.331314099	1.689495915
**TIGD6**	chr7	48970001	49120001	49099687	49105290	5.331314099	1.689495915
**NR3C1**	chr7	56030001	56180001	56055609	56173859	3.993747325	0.465154886
**AK3**	chr8	39630001	39780001	39635494	39656272	4.275663078	1.256629115
**CDC37L1**	chr8	39630001	39780001	39660101	39678648	4.275663078	1.256629115
**LOC10863655**	chr8	39630001	39780001	39713646	39733463	4.275663078	1.256629115
**PLPP6**	chr8	39630001	39780001	39700133	39703413	4.275663078	1.256629115
**SLC1A1**	chr8	39630001	39780001	39770036	39845247	4.275663078	1.256629115
**SPATA6L**	chr8	39630001	39780001	39734093	39756809	4.275663078	1.256629115
**LOC10217060**	chr8	48300001	48450001	48103346	48358321	6.770352314	0.799970839
**ZFAND5**	chr8	48300001	48450001	48393269	48404753	6.770352314	0.799970839
**UBAP2**	chr8	75030001	75180001	75124772	75238885	4.991784611	0.696009002
**UBE2R2**	chr8	75030001	75180001	75023162	75123568	4.991784611	0.696009002
**SPACA1**	chr9	49090001	49240001	49173871	49199828	4.589731479	0.451627735
**WDR72**	chr10	46300001	46450001	46414060	46635609	7.339901688	1.225058174
**ATP5S**	chr10	59800001	59950001	59820875	59831656	8.16018786	2.062164527
**CDKL1**	chr10	59800001	59950001	59763696	59820944	8.16018786	2.062164527
**L2HGDH**	chr10	59800001	59950001	59831705	59883022	8.16018786	2.062164527
**SOS2**	chr10	59800001	59950001	59890326	59991899	8.16018786	2.062164527
**MYO9A**	chr10	82460001	82610001	82335575	82577050	3.77472812	0.82252203
**NR2E3**	chr10	82460001	82610001	82590036	82594865	3.77472812	0.82252203
**BIRC6**	chr11	14750001	14900001	14814336	15024500	5.654912281	3.241322629
**YIPF4**	chr11	14750001	14900001	14738946	14782522	5.654912281	3.241322629
**ATAD2B**	chr11	74680001	74830001	74782987	74910341	4.49953004	0.452310108
**FAM228B**	chr11	74680001	74830001	74637060	74685661	4.49953004	0.452310108
**FKBP1B**	chr11	74680001	74830001	74698136	74708853	4.49953004	0.452310108
**MFSD2B**	chr11	74680001	74830001	74729851	74742391	4.49953004	0.452310108
**SF3B6**	chr11	74680001	74830001	74685654	74693957	4.49953004	0.452310108
**TP53I3**	chr11	74680001	74830001	74678510	74685183	4.49953004	0.452310108
**UBXN2A**	chr11	74680001	74830001	74745868	74769527	4.49953004	0.452310108
**WDCP**	chr11	74680001	74830001	74711378	74726672	4.49953004	0.452310108
**MYCBP2**	chr12	33740001	33890001	33616605	33885679	3.765612678	0.581443334
**ARHGAP12**	chr13	32640001	32790001	32767221	32884670	5.861998544	0.544320516

**KIF5B**	chr13	32640001	32790001	32665942	32713201	5.861998544	0.544320516
**TFAP2C**	chr13	58580001	58730001	58657523	58667140	6.894591642	1.848139163
**FABP5**	chr14	37420001	37570001	37555681	37561958	3.87398285	0.868863963
**LOC10650286**	chr14	37420001	37570001	37565973	37578551	3.87398285	0.868863963
**ANGPTL7**	chr16	40820001	40970001	40944001	40950549	7.210068794	0.420092778
**MTOR**	chr16	40820001	40970001	40889820	41015393	7.210068794	0.420092778
**UBIAD1**	chr16	40820001	40970001	40869569	40881667	7.210068794	0.420092778
**LOC10217072**	chr19	34260001	34410001	34401249	34407219	4.023455378	0.49551399
**MIR33B**	chr19	34260001	34410001	34294280	34294373	4.023455378	0.49551399
**RAI1**	chr19	34260001	34410001	34296405	34400733	4.023455378	0.49551399
**SREBF1**	chr19	34260001	34410001	34288792	34295549	4.023455378	0.49551399
**LOC10863849**	chr21	830001	980001	828399	835280	4.44993375	0.533246485
**DUSP22**	chr23	50001	200001	111611	159290	8.136425561	1.59447982
**IRF4**	chr23	50001	200001	189741	204470	8.136425561	1.59447982
**LOC10218055**	chr23	50001	200001	103134	111587	8.136425561	1.59447982
**GDF10**	chr28	3470001	3620001	3460297	3473327	6.31818497	2.913213984
**GDF2**	chr28	3470001	3620001	3482208	3486651	6.31818497	2.913213984
**LOC10217750**	chr28	3470001	3620001	3521579	3535635	6.31818497	2.913213984
**LOC10863333**	chr28	3470001	3620001	3549189	3563373	6.31818497	2.913213984
**RBP3**	chr28	3470001	3620001	3494829	3504269	6.31818497	2.913213984
**ZNF488**	chr28	3470001	3620001	3511701	3521545	6.31818497	2.913213984
**GPR174**	chrX1	2430001	2580001	2445642	2450303	7.203812286	0.747097385
**CHM**	chrX1	5380001	5530001	5218529	5465965	8.47547856	2.522883125
**ZDHHC15**	chrX1	7490001	7640001	7543624	7656270	10.43542284	0.753121315
**ERCC6L**	chrX1	9690001	9840001	9758235	9789589	8.272432095	0.901394004
**PIN4**	chrX1	9690001	9840001	9797484	9806164	8.272432095	0.901394004
**RPS4X**	chrX1	9690001	9840001	9710947	9715286	8.272432095	0.901394004
GBE1	chr1	28550001	28700001	27823980	28134705	3.602363403	0.540637055
TRNAS-GGA	chr1	29730001	29880001	30237432	30237503	4.845461054	0.676332558
LOC108636831	chr1	50960001	51110001	51225661	51369420	5.766762895	1.904745972
BDH1	chr1	71190001	71340001	71702243	71739306	4.077795841	1.519133746
CEP19	chr1	71190001	71340001	70953940	70960688	4.077795841	1.519133746
FBXO45	chr1	71190001	71340001	70833297	70847080	4.077795841	1.519133746
LOC10650198	chr1	71190001	71340001	71739387	71740951	4.077795841	1.519133746
LOC108635601	chr1	71190001	71340001	71071562	71085250	4.077795841	1.519133746
NCBP2	chr1	71190001	71340001	71133340	71140105	4.077795841	1.519133746
NRROS	chr1	71190001	71340001	70890387	70916589	4.077795841	1.519133746
PAK2	chr1	71190001	71340001	70990569	71073583	4.077795841	1.519133746
PIGX	chr1	71190001	71340001	70957571	70985213	4.077795841	1.519133746
PIGZ	chr1	71190001	71340001	71144173	71175164	4.077795841	1.519133746
RNF168	chr1	71190001	71340001	70765125	70782692	4.077795841	1.519133746
SENP5	chr1	71190001	71340001	71085339	71133963	4.077795841	1.519133746
SMCO1	chr1	71190001	71340001	70784055	70790921	4.077795841	1.519133746
WDR53	chr1	71190001	71340001	70820545	70833184	4.077795841	1.519133746
APOD	chr1	71240001	71390001	71801594	71815214	4.007461921	2.422088645
LOC10650207	chr1	71240001	71390001	71796710	71801248	4.007461921	2.422088645
ACAP2	chr1	71400001	71550001	71889654	72051523	3.570252188	2.33243566
PPP1R2	chr1	71400001	71550001	71839912	71861039	3.570252188	2.33243566
CCDC50	chr1	75940001	76090001	75702789	75778927	3.845600679	0.41356136
GMNC	chr1	75940001	76090001	76146215	76153710	3.845600679	0.41356136
IL1RAP	chr1	75940001	76090001	76348425	76502431	3.845600679	0.41356136
LOC10650209	chr1	75940001	76090001	75696854	75700886	3.845600679	0.41356136
OSTN	chr1	75940001	76090001	75832686	75869245	3.845600679	0.41356136
TRNAY-GUA	chr1	75940001	76090001	75729168	75729239	3.845600679	0.41356136
UTS2B	chr1	75940001	76090001	75811187	75826115	3.845600679	0.41356136
CCDC39	chr1	86180001	86330001	86026741	86083878	5.039951434	0.628265047
DNAJC19	chr1	86180001	86330001	85751161	85756951	5.039951434	0.628265047
FXR1	chr1	86180001	86330001	85759719	85832648	5.039951434	0.628265047
LOC10217473	chr1	86180001	86330001	86008316	86009287	5.039951434	0.628265047
PEX5L	chr1	86180001	86330001	86709401	86990660	5.039951434	0.628265047
TTC14	chr1	86180001	86330001	86086130	86095985	5.039951434	0.628265047
DYRK1A	chr1	149200001	149350001	149441823	149586835	7.13579866	1.220067334
HLCS	chr1	149200001	149350001	148908000	149101181	7.13579866	1.220067334
KCNJ6	chr1	149200001	149350001	149701472	149797540	7.13579866	1.220067334
LOC10650247	chr1	149200001	149350001	148833940	148856302	7.13579866	1.220067334
LOC10863638	chr1	149200001	149350001	149442265	149444359	7.13579866	1.220067334
LOC10863660	chr1	149200001	149350001	148937056	148939730	7.13579866	1.220067334
PIGP	chr1	149200001	149350001	149181657	149193145	7.13579866	1.220067334
RIPPLY3	chr1	149200001	149350001	149137812	149154486	7.13579866	1.220067334
SIM2	chr1	149200001	149350001	148856421	148906415	7.13579866	1.220067334
RPE	chr2	38480001	38630001	38025649	38055619	3.757885269	1.703640266

LOC10863776	chr2	38490001	38640001	38096326	38103292	3.757885269	1.542400338
LOC10219100(	chr2	38530001	38680001	38809543	38811982	4.585173759	1.44606391
UNC80	chr2	38530001	38680001	38057219	38272802	4.585173759	1.44606391
PTH2R	chr2	38620001	38770001	39142168	39237314	3.52863191	0.445404986
CARF	chr2	44180001	44330001	44630924	44682273	4.118960347	0.831762887
CD28	chr2	44180001	44330001	43971113	44007195	4.118960347	0.831762887
CTLA4	chr2	44180001	44330001	43834650	43898636	4.118960347	0.831762887
ICA1L	chr2	44180001	44330001	44715754	44771738	4.118960347	0.831762887
ICOS	chr2	44180001	44330001	43742923	43767248	4.118960347	0.831762887
LOC10217656	chr2	44180001	44330001	44390930	44434022	4.118960347	0.831762887
NBEAL1	chr2	44180001	44330001	44445261	44596606	4.118960347	0.831762887
WDR12	chr2	44180001	44330001	44682367	44708633	4.118960347	0.831762887
FAM117B	chr2	44200001	44350001	44772823	44849910	3.721402786	1.125952097
BMPR2	chr2	44430001	44580001	44895151	45051641	3.520324427	0.695849086
GTDC1	chr2	82980001	83130001	83524315	83969888	4.683102607	2.895711311
KYNU	chr2	82980001	83130001	82455732	82573836	4.683102607	2.895711311
DCAF17	chr2	110870001	111020001	111270166	111296211	3.682082085	0.896106212
GAD1	chr2	110870001	111020001	110670201	110709366	3.682082085	0.896106212
GORASP2	chr2	110870001	111020001	110759622	110790395	3.682082085	0.896106212
LOC10086113(	chr2	110870001	111020001	111347663	111380487	3.682082085	0.896106212
LOC10650279(	chr2	110870001	111020001	110586596	110589425	3.682082085	0.896106212
METTL8	chr2	110870001	111020001	111185392	111270066	3.682082085	0.896106212
MYO3B	chr2	110870001	111020001	110071275	110525479	3.682082085	0.896106212
SP5	chr2	110870001	111020001	110577471	110649747	3.682082085	0.896106212
BEST4	chr3	19630001	19780001	19533282	19537923	4.647552384	5.444823593
BTBD19	chr3	19630001	19780001	19559512	19564955	4.647552384	5.444823593
C3H1orf228	chr3	19630001	19780001	19416581	19475118	4.647552384	5.444823593
HPDL	chr3	19630001	19780001	20038094	20039756	4.647552384	5.444823593
KIF2C	chr3	19630001	19780001	19497048	19515005	4.647552384	5.444823593
LOC10218365	chr3	19630001	19780001	20200128	20203776	4.647552384	5.444823593
LOC10650197	chr3	19630001	19780001	20011606	20034411	4.647552384	5.444823593
LOC10650197	chr3	19630001	19780001	19351455	19376896	4.647552384	5.444823593
LOC10863564(	chr3	19630001	19780001	19492744	19496898	4.647552384	5.444823593
MMACHC	chr3	19630001	19780001	20203755	20209102	4.647552384	5.444823593
MUTYH	chr3	19630001	19780001	20040325	20052659	4.647552384	5.444823593
PLK3	chr3	19630001	19780001	19552113	19557154	4.647552384	5.444823593
PTCH2	chr3	19630001	19780001	19570536	19586439	4.647552384	5.444823593
RNF220	chr3	19630001	19780001	19134220	19396425	4.647552384	5.444823593
RPS8	chr3	19630001	19780001	19528475	19531122	4.647552384	5.444823593
TCTEX1D4	chr3	19630001	19780001	19557154	19559385	4.647552384	5.444823593
TESK2	chr3	19630001	19780001	20056153	20195032	4.647552384	5.444823593
TMEM53	chr3	19630001	19780001	19398934	19416381	4.647552384	5.444823593
TOE1	chr3	19630001	19780001	20052442	20056227	4.647552384	5.444823593
C3H1orf141	chr3	42510001	42660001	42689958	42739898	6.301259252	0.695709022
IL12RB2	chr3	42510001	42660001	42898126	42970364	6.301259252	0.695709022
IL23R	chr3	42510001	42660001	42777642	42839433	6.301259252	0.695709022
INSL5	chr3	42510001	42660001	42399363	42402829	6.301259252	0.695709022
LOC10217925	chr3	42510001	42660001	42142579	42143074	6.301259252	0.695709022
SERBP1	chr3	42510001	42660001	42979045	42995109	6.301259252	0.695709022
SGIP1	chr3	42510001	42660001	42109904	42342473	6.301259252	0.695709022
TCTEX1D1	chr3	42510001	42660001	42350053	42378791	6.301259252	0.695709022
BCAP29	chr4	71290001	71440001	71630040	71677966	5.85101858	0.689512446
CBLL1	chr4	71290001	71440001	71501625	71518069	5.85101858	0.689512446
COG5	chr4	71290001	71440001	71695962	71978448	5.85101858	0.689512446
DUS4L	chr4	71290001	71440001	71679995	71695871	5.85101858	0.689512446
GPR22	chr4	71290001	71440001	71759431	71765488	5.85101858	0.689512446
LAMB4	chr4	71290001	71440001	71082894	71201291	5.85101858	0.689512446
NRCAM	chr4	71290001	71440001	70733249	71043018	5.85101858	0.689512446
SLC26A3	chr4	71290001	71440001	71471442	71497737	5.85101858	0.689512446
SLC26A4	chr4	71290001	71440001	71541010	71598634	5.85101858	0.689512446
HBP1	chr4	71570001	71720001	71979140	72010036	5.561603305	1.349252948
PRKAR2B	chr4	71570001	71720001	72019339	72129472	5.561603305	1.349252948
PIK3CG	chr4	71820001	71970001	72257537	72291605	5.430610264	0.454013285
AHR	chr4	94490001	94640001	94675280	94726287	4.224409435	0.741763151
SNX13	chr4	94490001	94640001	94080605	94228482	4.224409435	0.741763151
VSTM2A	chr4	119610001	119760001	119990280	120015338	6.90345848	0.588026912
**KITLG**	chr5	18400001	18550001	18044632	18151924	5.451347894	2.824501837
TRNAK-UUU	chr5	18400001	18550001	18931313	18931385	5.451347894	2.824501837
LOC10216900	chr5	112490001	112640001	112064706	112073351	3.662421734	0.490304594
LOC10217733	chr5	112490001	112640001	112046093	112073327	3.662421734	0.490304594
BIK	chr5	112530001	112680001	112857123	112876056	4.411586744	1.438761099

LOC10217242:	chr5	112530001	112680001	112386168	112391658	4.411586744	1.438761099
LOC10217364:	chr5	112530001	112680001	112448192	112473179	4.411586744	1.438761099
MCAT	chr5	112530001	112680001	112879941	112895630	4.411586744	1.438761099
NFAM1	chr5	112530001	112680001	112285233	112321727	4.411586744	1.438761099
POLDIP3	chr5	112530001	112680001	112423180	112445045	4.411586744	1.438761099
RRP7A	chr5	112530001	112680001	112412807	112419720	4.411586744	1.438761099
SCUBE1	chr5	112530001	112680001	112949603	113076465	4.411586744	1.438761099
SERHL2	chr5	112530001	112680001	112391941	112412844	4.411586744	1.438761099
TCF20	chr5	112530001	112680001	112076058	112171316	4.411586744	1.438761099
TSPO	chr5	112530001	112680001	112906645	112919429	4.411586744	1.438761099
TTLL12	chr5	112530001	112680001	112921455	112939130	4.411586744	1.438761099
TTLL1	chr5	112530001	112680001	112793919	112835018	4.411586744	1.438761099
APBB2	chr6	60700001	60850001	60285849	60665198	5.006638638	0.979381613
NSUN7	chr6	60700001	60850001	60236339	60284745	5.006638638	0.979381613
PHOX2B	chr6	60700001	60850001	61216690	61220345	5.006638638	0.979381613
KDR	chr6	70650001	70800001	71115093	71162022	6.418703432	1.269078511
LOC10650220:	chr6	70650001	70800001	71112345	71114702	6.418703432	1.269078511
LOC10863627	chr6	70650001	70800001	70941596	70959636	6.418703432	1.269078511
PDGFRA	chr6	70650001	70800001	70285928	70334333	6.418703432	1.269078511
LAGE3	chr6	85400001	85550001	85840653	85841323	3.79432632	0.983364532
LOC10216852:	chr6	85400001	85550001	85677862	85734370	3.79432632	0.983364532
LOC10216984(	chr6	85400001	85550001	85878003	85901026	3.79432632	0.983364532
LOC10217196	chr6	85400001	85550001	85100990	85116854	3.79432632	0.983364532
LOC10217243:	chr6	85400001	85550001	85137434	85152951	3.79432632	0.983364532
LOC10217308:	chr6	85400001	85550001	85181313	85204348	3.79432632	0.983364532
LOC10217355:	chr6	85400001	85550001	85246765	85270440	3.79432632	0.983364532
LOC10218314(	chr6	85400001	85550001	85556243	85588921	3.79432632	0.983364532
LOC10650395:	chr6	85400001	85550001	85319850	85353942	3.79432632	0.983364532
LOC10863324(	chr6	85400001	85550001	85029807	85052189	3.79432632	0.983364532
SULT1B1	chr6	85400001	85550001	85775305	85812886	3.79432632	0.983364532
TRNAH-GUG	chr6	85400001	85550001	85872259	85872330	3.79432632	0.983364532
CSN1S1	chr6	85410001	85560001	85978463	85995270	3.621360812	1.367069834
ANTXR2	chr6	95410001	95560001	95056743	95230556	7.88154538	-5.892952164
PRDM8	chr6	95410001	95560001	95336875	95356821	7.88154538	-5.892952164
TRNAG-CCC	chr6	95410001	95560001	95616042	95616114	7.88154538	-5.892952164
CAMK4	chr7	670001	820001	80527	352060	3.542595109	1.869047665
SLC25A46	chr7	670001	820001	913717	942491	3.542595109	1.869047665
TMEM232	chr7	670001	820001	970691	1229535	3.542595109	1.869047665
WDR36	chr7	670001	820001	469008	513272	3.542595109	1.869047665
ARHGEF37	chr7	48970001	49120001	49434451	49493265	5.331314099	1.689495915
ARSI	chr7	48970001	49120001	48771935	48779194	5.331314099	1.689495915
CAMK2A	chr7	48970001	49120001	48785985	48851816	5.331314099	1.689495915
CD74	chr7	48970001	49120001	48668848	48676702	5.331314099	1.689495915
CDX1	chr7	48970001	49120001	48887463	48904695	5.331314099	1.689495915
CSNK1A1	chr7	48970001	49120001	49514129	49562875	5.331314099	1.689495915
LOC10863634:	chr7	48970001	49120001	48616701	48647168	5.331314099	1.689495915
LOC10863645:	chr7	48970001	49120001	49397699	49417138	5.331314099	1.689495915
MIR378	chr7	48970001	49120001	49342624	49342733	5.331314099	1.689495915
NDST1	chr7	48970001	49120001	48527503	48611610	5.331314099	1.689495915
PDE6A	chr7	48970001	49120001	49146863	49224055	5.331314099	1.689495915
PDGFRB	chr7	48970001	49120001	48915000	48952725	5.331314099	1.689495915
PPARGC1B	chr7	48970001	49120001	49224063	49345243	5.331314099	1.689495915
RPS14	chr7	48970001	49120001	48647020	48650971	5.331314099	1.689495915
SLC6A7	chr7	48970001	49120001	48859670	48880389	5.331314099	1.689495915
TCOF1	chr7	48970001	49120001	48677826	48716462	5.331314099	1.689495915
ARHGAP26	chr7	56030001	56180001	56232839	56713995	3.993747325	0.465154886
CHSY3	chr7	86730001	86880001	86320405	86608841	4.602990304	1.300898854
HINT1	chr7	86730001	86880001	87299552	87306155	4.602990304	1.300898854
LYRM7	chr7	86740001	86890001	87312006	87337065	4.310861113	1.329492762
ACSL6	chr7	88170001	88320001	88024364	88089819	3.553575073	0.639018307
CSF2	chr7	88170001	88320001	88163476	88165829	3.553575073	0.639018307
FNIP1	chr7	88170001	88320001	87745848	87868406	3.553575073	0.639018307
IL5	chr7	88170001	88320001	88684078	88686626	3.553575073	0.639018307
IRF1	chr7	88170001	88320001	88630486	88637730	3.553575073	0.639018307
LOC10218911(	chr7	88170001	88320001	88358467	88394609	3.553575073	0.639018307
LOC10219050	chr7	88170001	88320001	88148538	88150765	3.553575073	0.639018307
LOC10650231	chr7	88170001	88320001	88166964	88168905	3.553575073	0.639018307
LOC10863638:	chr7	88170001	88320001	88557879	88636200	3.553575073	0.639018307
LOC10863638(	chr7	88170001	88320001	88537122	88559426	3.553575073	0.639018307
MEIKIN	chr7	88170001	88320001	87869145	88020686	3.553575073	0.639018307
PDLIM4	chr7	88170001	88320001	88413050	88427472	3.553575073	0.639018307

RAD50	chr7	88170001	88320001	88702532	88815957	3.553575073	0.639018307
SLC22A4	chr7	88170001	88320001	88444408	88487123	3.553575073	0.639018307
SLC22A5	chr7	88170001	88320001	88511443	88537601	3.553575073	0.639018307
GLIS3	chr8	39630001	39780001	40049592	40544666	4.275663078	1.256629115
INSL6	chr8	39630001	39780001	39210866	39227365	4.275663078	1.256629115
**JAK2**	**chr8**	**39630001**	**39780001**	**39245187**	**39360411**	**4.275663078**	**1.256629115**
RCL1	chr8	39630001	39780001	39520371	39583801	4.275663078	1.256629115
ABHD17B	chr8	48300001	48450001	47856656	47901320	6.770352314	0.799970839
ALDH1A1	chr8	48300001	48450001	48842634	48898290	6.770352314	0.799970839
C8H9orf85	chr8	48300001	48450001	47901641	47965410	6.770352314	0.799970839
GDA	chr8	48300001	48450001	48163890	48299507	6.770352314	0.799970839
TMC1	chr8	48300001	48450001	48656137	48790796	6.770352314	0.799970839
TRNAC-ACA	chr8	48300001	48450001	48452463	48452534	6.770352314	0.799970839
TRNAS-AGA	chr8	48300001	48450001	48491343	48491414	6.770352314	0.799970839
LOC10863660[illegible]	chr8	67610001	67760001	67546369	67550356	5.85940893	1.043592334
DNAJA1	chr8	75020001	75170001	74588289	74599055	4.706388417	0.652970977
AQP3	chr8	75030001	75180001	74981478	74987380	4.991784611	0.696009002
AQP7	chr8	75030001	75180001	74925841	74942904	4.991784611	0.696009002
B4GALT1	chr8	75030001	75180001	74661494	74711790	4.991784611	0.696009002
BAG1	chr8	75030001	75180001	74814221	74826505	4.991784611	0.696009002
C8H9orf24	chr8	75030001	75180001	75524268	75539047	4.991784611	0.696009002
CHMP5	chr8	75030001	75180001	74826490	74840085	4.991784611	0.696009002
DCAF12	chr8	75030001	75180001	75279762	75315413	4.991784611	0.696009002
DNAI1	chr8	75030001	75180001	75600294	75669575	4.991784611	0.696009002
FAM219A	chr8	75030001	75180001	75539143	75600107	4.991784611	0.696009002
KIAA1161	chr8	75030001	75180001	75514875	75522929	4.991784611	0.696009002
KIF24	chr8	75030001	75180001	75427275	75497830	4.991784611	0.696009002
LOC10216918[illegible]	chr8	75030001	75180001	75382353	75382839	4.991784611	0.696009002
LOC10218885[illegible]	chr8	75030001	75180001	74712045	74742654	4.991784611	0.696009002
NFX1	chr8	75030001	75180001	74850386	74915589	4.991784611	0.696009002
NOL6	chr8	75030001	75180001	74996129	75008583	4.991784611	0.696009002
NUDT2	chr8	75030001	75180001	75498047	75508885	4.991784611	0.696009002
SMU1	chr8	75030001	75180001	74602402	74634128	4.991784611	0.696009002
SPINK4	chr8	75030001	75180001	74799650	74807172	4.991784611	0.696009002
UBAP1	chr8	75030001	75180001	75361372	75427272	4.991784611	0.696009002
AKIRIN2	chr9	49090001	49240001	49535238	49559370	4.589731479	0.451627735
CNR1	chr9	49090001	49240001	49058598	49089157	4.589731479	0.451627735
ORC3	chr9	49090001	49240001	49563207	49636451	4.589731479	0.451627735
RARS2	chr9	49090001	49240001	49636516	49709248	4.589731479	0.451627735
TRNAE-UUC	chr9	49090001	49240001	49452430	49452502	4.589731479	0.451627735
EPM2A	chr9	69560001	69710001	69975816	70097860	4.546992488	2.052384367
UTRN	chr9	69560001	69710001	68700661	69257114	4.546992488	2.052384367
FBXO30	chr9	69570001	69720001	70139832	70157217	4.46087228	2.213053386
UNC13C	chr10	46300001	46450001	46857881	47536542	7.339901688	1.225058174
ARF6	chr10	59800001	59950001	60176242	60180754	8.16018786	2.062164527
ATL1	chr10	59800001	59950001	59534244	59620451	8.16018786	2.062164527
DNAAF2	chr10	59800001	59950001	60362975	60373391	8.16018786	2.062164527
KLHDC1	chr10	59800001	59950001	60292061	60331139	8.16018786	2.062164527
KLHDC2	chr10	59800001	59950001	60273935	60288929	8.16018786	2.062164527
LOC10863699[illegible]	chr10	59800001	59950001	60096105	60123543	8.16018786	2.062164527
MAP4K5	chr10	59800001	59950001	59620555	59737329	8.16018786	2.062164527
MGAT2	chr10	59800001	59950001	60373610	60376239	8.16018786	2.062164527
NEMF	chr10	59800001	59950001	60219279	60273568	8.16018786	2.062164527
NIN	chr10	59800001	59950001	59358669	59461224	8.16018786	2.062164527
POLE2	chr10	59800001	59950001	60333137	60362906	8.16018786	2.062164527
SAV1	chr10	59800001	59950001	59504372	59533641	8.16018786	2.062164527
VCPKMT	chr10	59800001	59950001	59992012	59998534	8.16018786	2.062164527
LRR1	chr10	59810001	59960001	60380171	60390404	7.436483937	1.554576324
RPL36AL	chr10	59810001	59960001	60376270	60377558	7.436483937	1.554576324
RPS29	chr10	59830001	59980001	60400653	60402483	6.701406427	1.208801374
ARIH1	chr10	82460001	82610001	81939607	82041082	3.77472812	0.82252203
CELF6	chr10	82460001	82610001	82164246	82194306	3.77472812	0.82252203
GRAMD2	chr10	82460001	82610001	82266642	82301212	3.77472812	0.82252203
HEXA	chr10	82460001	82610001	82112311	82143840	3.77472812	0.82252203
LOC10217412[illegible]	chr10	82460001	82610001	82079094	82093970	3.77472812	0.82252203
LOC10217439[illegible]	chr10	82460001	82610001	82063046	82067055	3.77472812	0.82252203
PARP6	chr10	82460001	82610001	82205875	82232562	3.77472812	0.82252203
PKM	chr10	82460001	82610001	82238790	82265545	3.77472812	0.82252203
SENP8	chr10	82460001	82610001	82315289	82335360	3.77472812	0.82252203
THSD4	chr10	82460001	82610001	82620551	83285979	3.77472812	0.82252203
DPY30	chr11	14750001	14900001	14524697	14538756	5.654912281	3.241322629

LOC108637104	chr11	14750001	14900001	15031517	15032456	5.654912281	3.241322629
LTBP1	chr11	14750001	14900001	15324388	15780520	5.654912281	3.241322629
MEMO1	chr11	14750001	14900001	14404200	14515722	5.654912281	3.241322629
NLRC4	chr11	14750001	14900001	14669557	14716302	5.654912281	3.241322629
SLC30A6	chr11	14750001	14900001	14620539	14666163	5.654912281	3.241322629
SPAST	chr11	14750001	14900001	14556017	14608600	5.654912281	3.241322629
TTC27	chr11	14750001	14900001	15041424	15219539	5.654912281	3.241322629
FAM228A	chr11	74680001	74830001	74624305	74636502	4.49953004	0.452310108
ITSN2	chr11	74680001	74830001	74499640	74622540	4.49953004	0.452310108
KLHL29	chr11	74680001	74830001	74938480	75270624	4.49953004	0.452310108
NCOA1	chr11	74680001	74830001	74153980	74372125	4.49953004	0.452310108
PFN4	chr11	74680001	74830001	74669150	74674491	4.49953004	0.452310108
ACOD1	chr12	33740001	33890001	33959349	33966931	3.765612678	0.581443334
CLN5	chr12	33740001	33890001	33920524	33931904	3.765612678	0.581443334
FBXL3	chr12	33740001	33890001	33896229	33919253	3.765612678	0.581443334
KCTD12	chr12	33740001	33890001	34023639	34029762	3.765612678	0.581443334
LOC108637272	chr12	33740001	33890001	33515996	33519521	3.765612678	0.581443334
SCEL	chr12	33740001	33890001	33247740	33372142	3.765612678	0.581443334
CACNB2	chr13	32640001	32790001	31915665	32336714	5.861998544	0.544320516
EPC1	chr13	32640001	32790001	32467923	32564799	5.861998544	0.544320516
NSUN6	chr13	32640001	32790001	32338249	32416247	5.861998544	0.544320516
ZEB1	chr13	32640001	32790001	33123676	33323855	5.861998544	0.544320516
ADRA1D	chr13	49740001	49890001	50264597	50293882	6.179858141	0.620295215
HAO1	chr13	49740001	49890001	50024601	50086221	6.179858141	0.620295215
SMOX	chr13	49740001	49890001	50305402	50340974	6.179858141	0.620295215
SPO11	chr13	58540001	58690001	58104357	58119153	3.523494115	0.507530025
AURKA	chr13	58580001	58730001	58845806	58862526	6.894591642	1.848139163
BMP7	chr13	58580001	58730001	58174093	58260125	6.894591642	1.848139163
CASS4	chr13	58580001	58730001	58791766	58829365	6.894591642	1.848139163
CSTF1	chr13	58580001	58730001	58833053	58845722	6.894591642	1.848139163
FAM210B	chr13	58580001	58730001	58864336	58875132	6.894591642	1.848139163
FKBP1A	chr13	58580001	58730001	59038273	59061754	6.894591642	1.848139163
GCNT7	chr13	58580001	58730001	58749848	58761271	6.894591642	1.848139163
LOC102171800	chr13	58580001	58730001	58963406	58973585	6.894591642	1.848139163
LOC102172077	chr13	58580001	58730001	58980392	58991467	6.894591642	1.848139163
LOC102178461	chr13	58580001	58730001	58731400	58732833	6.894591642	1.848139163
MC3R	chr13	58580001	58730001	58935095	58936185	6.894591642	1.848139163
NSFL1C	chr13	58580001	58730001	59000052	59021299	6.894591642	1.848139163
RTFDC1	chr13	58580001	58730001	58735902	58783189	6.894591642	1.848139163
SDCBP2	chr13	58580001	58730001	59090093	59109110	6.894591642	1.848139163
SNPH	chr13	58580001	58730001	59109662	59153612	6.894591642	1.848139163
C13H20orf202	chr13	58770001	58920001	59230537	59243636	5.191682105	1.502285353
LOC102173205	chr13	58770001	58920001	59204147	59213351	5.191682105	1.502285353
PSMF1	chr13	58770001	58920001	59259919	59302429	5.191682105	1.502285353
RAD21L1	chr13	58770001	58920001	59165671	59201265	5.191682105	1.502285353
TMEM74B	chr13	58770001	58920001	59244717	59249746	5.191682105	1.502285353
FABP9	chr14	37420001	37570001	37386749	37389927	3.87398285	0.868863963
LOC100861279	chr14	37420001	37570001	37370685	37375203	3.87398285	0.868863963
LOC102180330	chr14	37420001	37570001	37401185	37406098	3.87398285	0.868863963
LOC102181136	chr14	37420001	37570001	37261248	37304883	3.87398285	0.868863963
LOC102183983	chr14	37420001	37570001	37319293	37328976	3.87398285	0.868863963
MAL2	chr14	37420001	37570001	37054286	37083765	3.87398285	0.868863963
NOV	chr14	37420001	37570001	37205654	37213987	3.87398285	0.868863963
PAG1	chr14	37420001	37570001	37722835	37882011	3.87398285	0.868863963
ZNF704	chr14	37420001	37570001	37966963	38224513	3.87398285	0.868863963
ASPH	chr14	54670001	54820001	54919151	55109204	5.115941071	0.983812908
CLVS1	chr14	54670001	54820001	55108124	55272244	5.115941071	0.983812908
NKAIN3	chr14	54670001	54820001	54129546	54655604	5.115941071	0.983812908
CLCN6	chr16	40780001	40930001	40326558	40359834	5.273845382	0.478324923
MTHFR	chr16	40790001	40940001	40359958	40374676	6.15818825	0.551944071
AGTRAP	chr16	40820001	40970001	40414580	40434456	7.210068794	0.420092778
C16H1orf127	chr16	40820001	40970001	41145834	41161708	7.210068794	0.420092778
C16H1orf167	chr16	40820001	40970001	40375150	40397444	7.210068794	0.420092778
CASZ1	chr16	40820001	40970001	41300131	41455846	7.210068794	0.420092778
DISP3	chr16	40820001	40970001	40642918	40700187	7.210068794	0.420092778
DRAXIN	chr16	40820001	40970001	40450639	40462803	7.210068794	0.420092778
EXOSC10	chr16	40820001	40970001	41023287	41045517	7.210068794	0.420092778
FBXO2	chr16	40820001	40970001	40518717	40525066	7.210068794	0.420092778
LOC102183714	chr16	40820001	40970001	40489918	40510465	7.210068794	0.420092778
LOC102184360	chr16	40820001	40970001	40513567	40518656	7.210068794	0.420092778
LOC106502956	chr16	40820001	40970001	40793627	40798534	7.210068794	0.420092778

MAD2L2	chr16	40820001	40970001	40484534	40489828	7.210068794	0.420092778
MASP2	chr16	40820001	40970001	41080217	41096199	7.210068794	0.420092778
SRM	chr16	40820001	40970001	41062640	41066649	7.210068794	0.420092778
TARDBP	chr16	40820001	40970001	41097063	41103700	7.210068794	0.420092778
CPE	chr17	69480001	69630001	69249530	69400252	8.940987597	1.096762359
KLHL2	chr17	69480001	69630001	69018320	69180171	8.940987597	1.096762359
MSMO1	chr17	69480001	69630001	69190106	69207149	8.940987597	1.096762359
TLL1	chr17	69790001	69940001	70288534	70463775	3.940318406	0.51668781
ALKBH5	chr19	34260001	34410001	34083409	34101930	4.023455378	0.49551399
ATPAF2	chr19	34260001	34410001	34184989	34197673	4.023455378	0.49551399
COPS3	chr19	34260001	34410001	34532194	34550031	4.023455378	0.49551399
DRC3	chr19	34260001	34410001	34198124	34214173	4.023455378	0.49551399
DRG2	chr19	34260001	34410001	34158391	34167225	4.023455378	0.49551399
EPN2	chr19	34260001	34410001	33782295	33835695	4.023455378	0.49551399
FAM83G	chr19	34260001	34410001	33895436	33924274	4.023455378	0.49551399
FLCN	chr19	34260001	34410001	34562388	34578706	4.023455378	0.49551399
FLII	chr19	34260001	34410001	34048218	34060204	4.023455378	0.49551399
GID4	chr19	34260001	34410001	34170501	34184804	4.023455378	0.49551399
GRAP	chr19	34260001	34410001	33858368	33879666	4.023455378	0.49551399
LLGL1	chr19	34260001	34410001	34060165	34075010	4.023455378	0.49551399
LOC10217312(	chr19	34260001	34410001	34813392	34819216	4.023455378	0.49551399
LOC106503231	chr19	34260001	34410001	34702981	34708025	4.023455378	0.49551399
MED9	chr19	34260001	34410001	34469592	34475211	4.023455378	0.49551399
MIEF2	chr19	34260001	34410001	34043363	34048134	4.023455378	0.49551399
MPRIP	chr19	34260001	34410001	34594212	34689199	4.023455378	0.49551399
MYO15A	chr19	34260001	34410001	34106198	34152663	4.023455378	0.49551399
NT5M	chr19	34260001	34410001	34515432	34528305	4.023455378	0.49551399
PEMT	chr19	34260001	34410001	34427348	34463596	4.023455378	0.49551399
PLD6	chr19	34260001	34410001	34584886	34587673	4.023455378	0.49551399
PRPSAP2	chr19	34260001	34410001	33938685	33961376	4.023455378	0.49551399
RASD1	chr19	34260001	34410001	34466190	34468560	4.023455378	0.49551399
SHMT1	chr19	34260001	34410001	33985506	34012237	4.023455378	0.49551399
SLC5A10	chr19	34260001	34410001	33879678	33935296	4.023455378	0.49551399
SMCR8	chr19	34260001	34410001	34012105	34021030	4.023455378	0.49551399
TNFRSF13B	chr19	34260001	34410001	34749523	34772452	4.023455378	0.49551399
TOP3A	chr19	34260001	34410001	34021122	34041435	4.023455378	0.49551399
USP22	chr19	34260001	34410001	34778451	34797475	4.023455378	0.49551399
MKRN3	chr21	700001	850001	368224	369765	4.082146393	0.824422412
LOC10217575	chr21	830001	980001	1128292	1135469	4.44993375	0.533246485
LOC10218853	chr21	830001	980001	1135590	1283333	4.44993375	0.533246485
LOC10650333	chr21	830001	980001	1346787	1351080	4.44993375	0.533246485
MAGEL2	chr21	830001	980001	418902	423047	4.44993375	0.533246485
NDN	chr21	830001	980001	463679	465342	4.44993375	0.533246485
SNRPN	chr21	830001	980001	1109040	1115092	4.44993375	0.533246485
SNURF	chr21	830001	980001	1091977	1113163	4.44993375	0.533246485
ADAMTSL3	chr21	23100001	23250001	23559613	23931139	6.221830607	0.479996211
EFL1	chr21	23100001	23250001	23389905	23501720	6.221830607	0.479996211
LOC10218457	chr21	23100001	23250001	22654945	22773169	6.221830607	0.479996211
LOC10863845	chr21	23100001	23250001	22816979	22829042	6.221830607	0.479996211
LOC108638471	chr21	23100001	23250001	22879566	22880445	6.221830607	0.479996211
LOC10863851	chr21	23100001	23250001	23501768	23503984	6.221830607	0.479996211
LOC10863853	chr21	23100001	23250001	22853361	22858753	6.221830607	0.479996211
MEX3B	chr21	23100001	23250001	23314666	23317865	6.221830607	0.479996211
SAXO2	chr21	23100001	23250001	23508086	23519714	6.221830607	0.479996211
BNC1	chr21	23630001	23780001	24191923	24220410	4.600504274	0.428499799
SH3GL3	chr21	23630001	23780001	23947019	24053896	4.600504274	0.428499799
EXOC2	chr23	50001	200001	237311	359937	8.136425561	1.59447982
LOC10863344	chr23	50001	200001	567430	569485	8.136425561	1.59447982
FOXQ1	chr23	70001	220001	641272	643823	5.461001975	1.137823382
FOXF2	chr23	120001	270001	691668	696627	4.410447314	1.014177925
FOXC1	chr23	270001	420001	780584	784092	4.16598774	0.775201697
GMDS	chr23	270001	420001	792203	1217301	4.16598774	0.775201697
LOC10217961	chr23	270001	420001	765292	769039	4.16598774	0.775201697
MAPK8	chr28	3460001	3610001	2941175	3039589	5.084099176	2.742224133
ANTXRL	chr28	3470001	3620001	3727694	3772919	6.31818497	2.913213984
FAM35A	chr28	3470001	3620001	3966919	4070554	6.31818497	2.913213984
FRMPD2	chr28	3470001	3620001	3087917	3155034	6.31818497	2.913213984
GPRIN2	chr28	3470001	3620001	3917031	3935587	6.31818497	2.913213984
LOC10217243	chr28	3470001	3620001	3836198	3843754	6.31818497	2.913213984
LOC10217462	chr28	3470001	3620001	3937188	3948104	6.31818497	2.913213984
LOC10217649	chr28	3470001	3620001	3784690	3801075	6.31818497	2.913213984

LOC102188472	chr28	3470001	3620001	3948193	3957398	6.31818497	2.913213984
LOC108634139	chr28	3470001	3620001	3167684	3194936	6.31818497	2.913213984
PTPN20	chr28	3470001	3620001	3207633	3233696	6.31818497	2.913213984
TRNAA-AGC	chr28	3470001	3620001	3823011	3823082	6.31818497	2.913213984
ATRX	chrX1	2430001	2580001	2844617	3079823	7.203812286	0.747097385
CYSLTR1	chrX1	2430001	2580001	2639169	2709712	7.203812286	0.747097385
LOC102178713	chrX1	2430001	2580001	2763653	2793750	7.203812286	0.747097385
LOC102184043	chrX1	2430001	2580001	2403912	2428110	7.203812286	0.747097385
LOC102184234	chrX1	2430001	2580001	2184806	2204285	7.203812286	0.747097385
LPAR4	chrX1	2430001	2580001	2087559	2099113	7.203812286	0.747097385
CYLC1	chrX1	5380001	5530001	5770546	5816316	8.47547856	2.522883125
MIR361	chrX1	5380001	5530001	5296302	5296397	8.47547856	2.522883125
TRNAW-CCA	chrX1	5380001	5530001	5909913	5909985	8.47547856	2.522883125
ABCB7	chrX1	7490001	7640001	7666353	7809522	10.43542284	0.753121315
HDX	chrX1	7490001	7640001	7167355	7192928	10.43542284	0.753121315
KIAA2022	chrX1	7490001	7640001	7875317	8084555	10.43542284	0.753121315
MAGEE2	chrX1	7490001	7640001	7358153	7360444	10.43542284	0.753121315
RPS6KA6	chrX1	7490001	7640001	7095036	7148417	10.43542284	0.753121315
TRNAC-GCA	chrX1	7490001	7640001	8014720	8014791	10.43542284	0.753121315
NAP1L2	chrX1	9680001	9830001	9253597	9256075	8.272432095	0.772385717
CITED1	chrX1	9690001	9840001	9674906	9680321	8.272432095	0.901394004
FGF16	chrX1	9690001	9840001	10034151	10043086	8.272432095	0.901394004
HDAC8	chrX1	9690001	9840001	9335339	9343047	8.272432095	0.901394004
LOC108634317	chrX1	9690001	9840001	10190999	10195135	8.272432095	0.901394004
LOC108634345	chrX1	9690001	9840001	10068848	10075883	8.272432095	0.901394004
LOC108634356	chrX1	9690001	9840001	9621510	9654343	8.272432095	0.901394004
NHSL2	chrX1	9690001	9840001	9845027	9866306	8.272432095	0.901394004
PHKA1	chrX1	9690001	9840001	9352165	9527318	8.272432095	0.901394004
RGAG4	chrX1	9690001	9840001	9866637	9871155	8.272432095	0.901394004

Supplementary Table s6：Annotation of candidate genes in UC goats and other cashmere goats to perform selective evolution analysis

Input ID	Gene ID	Type	Tax ID	Homologer	Homologer	Gene Symt	Descripti	Biologica	Kinase Cl	Protein F	Subcellul	Drug (Dru	Canonical	Hallmark	GO:000724	GO:006007	GO:003509	R-HSA-400	GO:003025	GO:004362	GO:000689	R-HSA-533	GO:004858	hsa05215	GO:004566	GO:000734	GO:006068	R-HSA-886	R-HSA-886	GO:190101	hsa04730	GO:000170	GO:190157	GO:1900181 negative regulation of protein
INSIG2	51141	symbol	H. sapiens	51141	H. sapiens	INSIG2	insulin indu	GO:0060363 cranial suture morphogenesis;GO:0045717 negative regulation of							0.0	0.0	0.0	0.0	1.0	0.0	0.0	0.0	0.0	0.0	0.0	0.0	0.0	0.0	0.0	0.0	0.0	1.0	1.0	0.0
CCDC93	54520	symbol	H. sapiens	54520	H. sapiens	CCDC93	coiled-coil	GO:1990126 retrograde transport			Vesicles(Supported)Plasma membrane(Appro				0.0	0.0	0.0	0.0	0.0	0.0	1.0	0.0	0.0	0.0	0.0	0.0	0.0	0.0	0.0	0.0	0.0	0.0	0.0	0.0
DDX18	8886	symbol	H. sapiens	8886	H. sapiens	DDX18	DEAD-box	GO:0000463 maturatio		Enzymes/E	Nucleoli(Enhanced)		(M66)PID N	(M5928)HA	0.0	0.0	0.0	0.0	0.0	0.0	0.0	0.0	0.0	0.0	0.0	0.0	0.0	0.0	0.0	0.0	0.0	0.0	0.0	0.0
RASSF9	9182	symbol	H. sapiens	9182	H. sapiens	RASSF9	Ras associa	GO:0006605 protein targeting;GO:0016197 endosomal transport;GO:0006886 i							0.0	0.0	0.0	0.0	0.0	0.0	0.0	0.0	0.0	0.0	0.0	0.0	0.0	0.0	0.0	0.0	0.0	0.0	0.0	0.0
NTS	4922	symbol	H. sapiens	4922	H. sapiens	NTS	neurotensir	GO:0007186 G protein-coupled receptor signaling pathw					(M167)PID AP1 PATHW		0.0	0.0	0.0	0.0	0.0	0.0	0.0	0.0	0.0	0.0	0.0	0.0	0.0	0.0	0.0	0.0	0.0	0.0	0.0	0.0
MGAT4C	25834	symbol	H. sapiens	25834	H. sapiens	MGAT4C	MGAT4 fan	GO:0006487 protein N		Enzymes/ENZYME proteins/Transferases					0.0	0.0	0.0	0.0	0.0	0.0	0.0	0.0	0.0	0.0	0.0	0.0	0.0	0.0	0.0	0.0	0.0	0.0	0.0	0.0
PLEKHG7	440107	symbol	H. sapiens	440107	H. sapiens	PLEKHG7	pleckstrin homology and RhoGEF domain containing G7								0.0	0.0	0.0	0.0	0.0	0.0	0.0	0.0	0.0	0.0	0.0	0.0	0.0	0.0	0.0	0.0	0.0	0.0	0.0	0.0
EEA1	8411	symbol	H. sapiens	8411	H. sapiens	EEA1	early endos	GO:0016189 synaptic vesicle to er			Vesicles(Su	Inositol 1,3-Bisphosphate (Zinc io			1.0	0.0	0.0	0.0	0.0	0.0	0.0	0.0	0.0	0.0	0.0	0.0	0.0	0.0	0.0	0.0	0.0	0.0	0.0	0.0
TRNAE-CU	10834864	symbol	R. norvegicus			None	None	None	None	None	None	None	None	None	nan	nan	nan	nan	nan	nan	nan	nan	nan	nan	nan	nan	nan	nan	nan	nan	nan	nan	nan	nan
NUDT4	11163	symbol	H. sapiens	11163	H. sapiens	NUDT4	nudix hydr	GO:1901911 adenosin		Enzymes/E	Cytosol(Approved)			(M5945)HA	0.0	0.0	0.0	0.0	0.0	0.0	0.0	0.0	0.0	0.0	0.0	0.0	0.0	0.0	0.0	0.0	0.0	0.0	0.0	0.0
UBE2N	7334	symbol	H. sapiens	7334	H. sapiens	UBE2N	ubiquitin c	GO:0070423 nucleotic		Enzymes/E	Nucleoli fibrillar center		(M84)PID A	(M5923)HA	1.0	0.0	0.0	0.0	0.0	0.0	0.0	0.0	0.0	0.0	0.0	0.0	0.0	1.0	0.0	0.0	0.0	0.0	0.0	0.0
MRPL42	28977	symbol	H. sapiens	28977	H. sapiens	MRPL42	mitochond	GO:0070126 mitochor		Ribosomal	Mitochondria(Supported)Plasma membrane(A				0.0	0.0	0.0	0.0	0.0	1.0	0.0	0.0	0.0	0.0	0.0	0.0	0.0	0.0	0.0	0.0	0.0	0.0	0.0	0.0
SOCS2	8835	symbol	H. sapiens	8835	H. sapiens	SOCS2	suppressor	GO:0060396 growth hormone rec			Endoplasmic reticulum		(M122)PID	(M5947)HA	0.0	0.0	0.0	0.0	1.0	0.0	0.0	0.0	1.0	0.0	0.0	0.0	0.0	0.0	0.0	0.0	0.0	0.0	0.0	0.0
CRADD	8738	symbol	H. sapiens	8738	H. sapiens	CRADD	CASP2 and	GO:0006977 DNA damage respo			Cytosol;Nucleus(Appro		(M197)PID HIV NEF PA		0.0	0.0	0.0	0.0	0.0	0.0	0.0	0.0	0.0	0.0	0.0	1.0	0.0	0.0	0.0	0.0	0.0	0.0	0.0	0.0
ALPK1	80216	symbol	H. sapiens	80216	H. sapiens	ALPK1	alpha kinas	GO:00027	Atypical: Al	Enzymes/K	Centrosome(Approved)				1.0	0.0	0.0	0.0	0.0	0.0	0.0	0.0	0.0	0.0	0.0	0.0	0.0	0.0	0.0	0.0	0.0	0.0	0.0	0.0
TIFA	92610	symbol	H. sapiens	92610	H. sapiens	TIFA	TRAF intera	GO:0002753 cytoplasmic pattern			Cytosol;Nucleoli;Nucleus(Approved)				1.0	0.0	0.0	0.0	0.0	0.0	0.0	0.0	0.0	0.0	0.0	0.0	0.0	0.0	0.0	0.0	0.0	0.0	0.0	0.0
AP1AR	55435	symbol	H. sapiens	55435	H. sapiens	AP1AR	adaptor rel	GO:0034315 regulation of Arp2/3			Golgi apparatus(Supported)				0.0	0.0	0.0	0.0	0.0	0.0	1.0	0.0	1.0	0.0	0.0	0.0	0.0	0.0	0.0	0.0	0.0	0.0	0.0	0.0
C6H4orf32						None	None	None	None	None	None	None	None	None	nan	nan	nan	nan	nan	nan	nan	nan	nan	nan	nan	nan	nan	nan	nan	nan	nan	nan	nan	nan
PDLIM5	10611	symbol	H. sapiens	10611	H. sapiens	PDLIM5	PDZ and LI	GO:0061001 regulation of dendrit			Focal adhesion sites;Nucleoplasm			(M5908)HA	0.0	0.0	0.0	0.0	0.0	0.0	0.0	0.0	1.0	0.0	0.0	0.0	0.0	0.0	0.0	0.0	0.0	0.0	0.0	0.0
HPGDS	27306	symbol	H. sapiens	27306	H. sapiens	HPGDS	hematopoi	GO:0019371 cyclooxy		Enzymes/ENZYME pro		Glutathione (Protein homodimeriz			0.0	0.0	0.0	0.0	1.0	0.0	0.0	0.0	0.0	0.0	0.0	0.0	0.0	0.0	0.0	0.0	0.0	0.0	1.0	0.0
SMARCAD1	56916	symbol	H. sapiens	56916	H. sapiens	SMARCAD1	SWI/SNF-re	GO:0070933 histone H		Enzymes/E	Nucleoplasm(Enhanced)				0.0	0.0	0.0	0.0	0.0	0.0	0.0	0.0	0.0	0.0	0.0	0.0	0.0	0.0	0.0	0.0	0.0	0.0	0.0	0.0
ATOH1	474	symbol	H. sapiens	474	H. sapiens	ATOH1	atonal bHL	GO:0045609 positive r		Transcription factors/Basic domains					0.0	0.0	0.0	0.0	0.0	0.0	0.0	0.0	1.0	0.0	0.0	0.0	0.0	0.0	0.0	0.0	0.0	1.0	0.0	0.0
TRNAS-GG	10818056	symbol	D. rerio			None	None	None	None	None	None	None	None	None	nan	nan	nan	nan	nan	nan	nan	nan	nan	nan	nan	nan	nan	nan	nan	nan	nan	nan	nan	nan
GRID2	2895	symbol	H. sapiens	2895	H. sapiens	GRID2	glutamate	GO:0021707 cerebellar granule cell differentia				L-Glutamic Acid (Scaffo		(M5956)HA	0.0	1.0	0.0	0.0	0.0	0.0	0.0	0.0	0.0	0.0	0.0	0.0	0.0	0.0	0.0	0.0	1.0	0.0	0.0	0.0
TRNAE-UU	107985602	symbol	H. sapiens	107985602	H. sapiens	TRNAE-UU	transfer RNA glutamic acid (anticodon UUC)								0.0	0.0	0.0	0.0	0.0	0.0	0.0	0.0	0.0	0.0	0.0	0.0	0.0	0.0	0.0	0.0	0.0	0.0	0.0	0.0
ANTXR2	118429	symbol	H. sapiens	118429	H. sapiens	ANTXR2	ANTXR cell	GO:1901998 toxin transport;GO:0022414 reproductive p					(M267)PID	(M5892)HA	0.0	0.0	0.0	0.0	0.0	0.0	0.0	1.0	0.0	0.0	0.0	0.0	0.0	0.0	0.0	0.0	0.0	0.0	0.0	0.0
PRDM8	56978	symbol	H. sapiens	56978	H. sapiens	PRDM8	PR/SET dor	GO:0014003 oligoden		Transcriptic	Nuclear bodies(Approved)				0.0	0.0	0.0	0.0	0.0	0.0	0.0	0.0	0.0	0.0	0.0	0.0	0.0	0.0	0.0	0.0	0.0	0.0	0.0	0.0
FGF5	2250	symbol	H. sapiens	2250	H. sapiens	FGF5	fibroblast g	GO:0023019 signal tra	RAS pathw		Nucleoli fibrillar center		(M5883)NABA SECRET		0.0	0.0	0.0	0.0	0.0	0.0	0.0	0.0	0.0	0.0	0.0	0.0	0.0	1.0	0.0	0.0	0.0	0.0	0.0	0.0
C6H4orf22						None	None	None	None	None	None	None	None	None	nan	nan	nan	nan	nan	nan	nan	nan	nan	nan	nan	nan	nan	nan	nan	nan	nan	nan	nan	nan
TRNAG-CC	10798560	symbol	H. sapiens	10798560	H. sapiens	TRNAG-CC	transfer RNA glycine (anticodon CCC)								0.0	0.0	0.0	0.0	0.0	0.0	0.0	0.0	0.0	0.0	0.0	0.0	0.0	0.0	0.0	0.0	0.0	0.0	0.0	0.0
VCAN	1462	symbol	H. sapiens	1462	H. sapiens	VCAN	versican	GO:0030208 dermatan sulfate bic			Vesicles(Su	Hyaluronic	(M264)PID	(M5944)HA	1.0	1.0	0.0	0.0	0.0	0.0	0.0	0.0	0.0	0.0	0.0	0.0	0.0	0.0	0.0	0.0	0.0	0.0	1.0	0.0
XRCC4	7518	symbol	H. sapiens	7518	H. sapiens	XRCC4	X-ray repai	GO:0075713 establishment of inte			Nucleus(Su	S-(Dimethy	(M42)PID DNA PK PAT		0.0	0.0	0.0	0.0	0.0	0.0	0.0	0.0	0.0	0.0	0.0	0.0	0.0	0.0	0.0	0.0	0.0	0.0	0.0	0.0
TMEM167A	153339	symbol	H. sapiens	153339	H. sapiens	TMEM167A	transmemb	GO:0009306 protein secretion;GO:0002790 peptide secretion;GO:0032940 secr							0.0	0.0	0.0	0.0	0.0	0.0	0.0	0.0	0.0	0.0	0.0	0.0	0.0	0.0	0.0	0.0	0.0	0.0	0.0	0.0
TMCO6	55374	symbol	H. sapiens	55374	H. sapiens	TMCO6	transmemb	GO:0006606 protein import into r			Mitochondria;Nucleoplasm(Approved)				0.0	0.0	0.0	0.0	0.0	0.0	0.0	0.0	0.0	0.0	0.0	0.0	0.0	0.0	0.0	0.0	0.0	0.0	0.0	0.0
CD14	929	symbol	H. sapiens	929	H. sapiens	CD14	CD14 mole	GO:0034128 negative	CD marker		Vesicles(Supported)		(M264)PID	(M5897)HA	1.0	0.0	0.0	0.0	0.0	0.0	0.0	0.0	0.0	0.0	0.0	0.0	0.0	0.0	0.0	0.0	0.0	0.0	0.0	0.0
SLC35A4	113829	symbol	H. sapiens	113829	H. sapiens	SLC35A4	solute carri	GO:0090481 pyrimidir	Transporte		Nucleoplasm(Approved)				0.0	0.0	0.0	0.0	0.0	0.0	0.0	0.0	0.0	0.0	0.0	0.0	0.0	0.0	0.0	0.0	0.0	0.0	0.0	0.0
APBB3	10307	symbol	H. sapiens	10307	H. sapiens	APBB3	amyloid be	GO:0006355 regulation of transcri			Cytosol(Supported)Actin filaments(Approved)				0.0	0.0	0.0	0.0	0.0	0.0	0.0	0.0	0.0	0.0	0.0	0.0	0.0	0.0	0.0	0.0	0.0	0.0	0.0	0.0
SRA1	10011	symbol	H. sapiens	10011	H. sapiens	SRA1	steroid rec	GO:0045662 negative regulation c			Cytosol;Nucleoplasm(S		(M200)PID ERA GENO		0.0	0.0	0.0	0.0	0.0	0.0	0.0	0.0	0.0	0.0	1.0	1.0	0.0	0.0	0.0	0.0	0.0	0.0	0.0	0.0
EIF4EBP3	8637	symbol	H. sapiens	8637	H. sapiens	EIF4EBP3	eukaryotic	GO:0045947 negative regulation of translational initiation;GO:0006446 regulatio							0.0	0.0	0.0	0.0	0.0	0.0	0.0	0.0	0.0	0.0	0.0	0.0	0.0	0.0	0.0	0.0	0.0	0.0	0.0	0.0
TRNAC-GC	10798560	symbol	H. sapiens	10798560	H. sapiens	TRNAC-GC	transfer RNA cysteine (anticodon GCA)								0.0	0.0	0.0	0.0	0.0	0.0	0.0	0.0	0.0	0.0	0.0	0.0	0.0	0.0	0.0	0.0	0.0	0.0	0.0	0.0
SLC4A9	83697	symbol	H. sapiens	83697	H. sapiens	SLC4A9	solute carri	GO:0051453 regulatio	Transporters/Electrochemical Potential-driven transporte						0.0	0.0	0.0	0.0	0.0	0.0	0.0	0.0	0.0	0.0	0.0	0.0	0.0	0.0	0.0	0.0	0.0	0.0	0.0	0.0
HBEGF	1839	symbol	H. sapiens	1839	H. sapiens	HBEGF	heparin bir	GO:0051545 negative regulation of elastin biosynthetic p					(M201)PID	(M5932)HA	0.0	0.0	0.0	1.0	0.0	0.0	0.0	1.0	0.0	0.0	0.0	0.0	0.0	0.0	1.0	0.0	0.0	0.0	0.0	0.0
PFDN1	5201	symbol	H. sapiens	5201	H. sapiens	PFDN1	prefoldin s	GO:0006457 protein folding;GO:0			Cytosol;Nucleoli;Plasma membrane(Approve				0.0	0.0	0.0	0.0	0.0	0.0	0.0	0.0	0.0	0.0	0.0	0.0	0.0	0.0	0.0	0.0	0.0	0.0	0.0	0.0
CYSTM1	84418	symbol	H. sapiens	84418	H. sapiens	CYSTM1	cysteine ric	GO:0043312 neutrophil degranulation;GO:0002283 neutrophil activation involv							0.0	0.0	0.0	0.0	0.0	0.0	0.0	0.0	0.0	0.0	0.0	0.0	0.0	0.0	0.0	0.0	0.0	0.0	0.0	0.0
IGIP	492311	symbol	H. sapiens	492311	H. sapiens	IGIP	IgA inducing protein				Vesicles(Approved)				0.0	0.0	0.0	0.0	0.0	0.0	0.0	0.0	0.0	0.0	0.0	0.0	0.0	0.0	0.0	0.0	0.0	0.0	0.0	0.0
PURA	5813	symbol	H. sapiens	5813	H. sapiens	PURA	purine rich	GO:0098963 dendritic	Transcription factors/Yet undefined DNA-binc					(M5901)HA	0.0	0.0	0.0	0.0	0.0	0.0	0.0	0.0	0.0	0.0	0.0	0.0	1.0	0.0	0.0	0.0	0.0	0.0	0.0	0.0
NRG2	9542	symbol	H. sapiens	9542	H. sapiens	NRG2	neuregulin	GO:0038128 ERBB2 signaling pat			Nucleoli;Nucleus(Supp		(M201)PID ERBB NETW		0.0	0.0	0.0	0.0	0.0	0.0	0.0	0.0	0.0	0.0	0.0	0.0	0.0	0.0	1.0	0.0	0.0	0.0	0.0	0.0
PSD2	84249	symbol	H. sapiens	84249	H. sapiens	PSD2	pleckstrin a	GO:0032012 regulation of ARF protein signal transduction;GO:0032011 ARF prc							0.0	0.0	0.0	0.0	0.0	0.0	0.0	0.0	0.0	0.0	0.0	0.0	0.0	0.0	0.0	0.0	0.0	0.0	0.0	0.0
CXXC5	51523	symbol	H. sapiens	51523	H. sapiens	CXXC5	CXXC finge	GO:0043123 positive regulation o			Cytosol;Nucleoplasm(Enhanced)				1.0	0.0	0.0	0.0	0.0	0.0	0.0	0.0	0.0	0.0	0.0	0.0	0.0	0.0	0.0	0.0	0.0	0.0	0.0	0.0
UBE2D2	7322	symbol	H. sapiens	7322	H. sapiens	UBE2D2	ubiquitin c	GO:0035666 TRIF-dep		Enzymes/E	Cytosol;Pla	(R,R)-2,3-E	(M214)PID ERBB1 INTE		1.0	0.0	0.0	0.0	0.0	0.0	0.0	0.0	0.0	0.0	0.0	0.0	1.0	0.0	0.0	0.0	0.0	0.0	0.0	0.0
DTWD2	285605	symbol	H. sapiens	285605	H. sapiens	DTWD2	DTW domain containing 2				Nuclear bodies(Supported)				0.0	0.0	0.0	0.0	0.0	0.0	0.0	0.0	0.0	0.0	0.0	0.0	0.0	0.0	0.0	0.0	0.0	0.0	0.0	0.0
DMXL1	1657	symbol	H. sapiens	1657	H. sapiens	DMXL1	Dmx like 1	GO:0007035 vacuolar acidificatior			Cytosol;Nucleoli(Supported)				0.0	0.0	0.0	0.0	0.0	0.0	0.0	0.0	0.0	0.0	0.0	0.0	0.0	0.0	0.0	0.0	0.0	0.0	0.0	0.0
TNFAIP8	25816	symbol	H. sapiens	25816	H. sapiens	TNFAIP8	TNF alpha i	GO:0043154 negative regulation c			Nucleoplasm(Approved)			(M5908)HA	0.0	0.0	0.0	0.0	1.0	0.0	0.0	0.0	0.0	0.0	0.0	0.0	0.0	0.0	0.0	0.0	0.0	0.0	0.0	0.0
HSD17B4	3295	symbol	H. sapiens	3295	H. sapiens	HSD17B4	hydroxyste	GO:0036111 very long		Enzymes/E	Peroxisome	NADH (Receptor bindi		(M5949)HA	0.0	0.0	0.0	0.0	1.0	0.0	0.0	0.0	0.0	0.0	0.0	0.0	1.0	0.0	0.0	0.0	0.0	0.0	1.0	0.0
FAM170A	340069	symbol	H. sapiens	340069	H. sapiens	FAM170A	family with	GO:0045893 positive regulation of transcription, DNA-templated;GO:1903508 p							0.0	0.0	0.0	0.0	0.0	0.0	0.0	0.0	0.0	0.0	0.0	0.0	0.0	0.0	0.0	0.0	0.0	0.0	0.0	0.0
TRNAW-CC	10834863	symbol	R. norvegicus			None	None	None	None	None	None	None	None	None	nan	nan	nan	nan	nan	nan	nan	nan	nan	nan	nan	nan	nan	nan	nan	nan	nan	nan	nan	nan
PRR16	51334	symbol	H. sapiens	51334	H. sapiens	PRR16	proline rich	GO:0045793 positive regulation o			Cytosol;Midbody;Nucleus(Approved)				0.0	0.0	0.0	0.0	0.0	0.0	0.0	0.0	0.0	0.0	0.0	0.0	0.0	0.0	0.0	0.0	0.0	0.0	0.0	0.0
TLE6	79816	symbol	H. sapiens	79816	H. sapiens	TLE6	TLE family	GO:0060136 embryonic process involved in female pregnancy;GO:0090090 neg							0.0	1.0	0.0	0.0	0.0	0.0	0.0	0.0	0.0	0.0	0.0	0.0	0.0	0.0	0.0	0.0	0.0	0.0	0.0	0.0
TLE2	7089	symbol	H. sapiens	7089	H. sapiens	TLE2	TLE family	GO:1904837 beta-catenin-TCF cc			Nuclear bodies(Suppo		(M223)PID BETA CATE		0.0	1.0	0.0	0.0	0.0	0.0	0.0	0.0	0.0	0.0	0.0	0.0	0.0	0.0	0.0	0.0	0.0	0.0	0.0	0.0
AES	166	gene_synon	H. sapiens	166	H. sapiens	TLE5	TLE family	GO:2000210 positive regulation o			Nucleoplasm(Supporte		(M70)PID PS1 PATHWA		1.0	1.0	0.0	0.0	0.0	0.0	0.0	0.0	0.0	0.0	0.0	0.0	0.0	0.0	0.0	0.0	0.0	0.0	0.0	0.0
GNA11	2767	symbol	H. sapiens	2767	H. sapiens	GNA11	G protein s	GO:0045634 regulation of melanocyte differentiation;GO					(M138)PID THROMBIN		0.0	0.0	0.0	1.0	0.0	0.0	0.0	0.0	0.0	0.0	0.0	0.0	0.0	0.0	0.0	0.0	1.0	0.0	0.0	0.0
GNA15	2769	symbol	H. sapiens	2769	H. sapiens	GNA15	G protein s	GO:0007207 phospholipase C-activating G protein-cour					(M138)PID	(M5902)HA	0.0	0.0	0.0	1.0	0.0	0.0	0.0	0.0	0.0	0.0	0.0	0.0	0.0	0.0	0.0	0.0	0.0	0.0	0.0	0.0
TRNAV-CA	10798560	symbol	H. sapiens	10798560	H. sapiens	TRNAV-CA	transfer RNA valine (anticodon CAC)								0.0	0.0	0.0	0.0	0.0	0.0	0.0	0.0	0.0	0.0	0.0	0.0	0.0	0.0	0.0	0.0	0.0	0.0	0.0	0.0
S1PR4	8698	symbol	H. sapiens	8698	H. sapiens	S1PR4	sphingosin	GO:0007202 activatio	G-protein c		Mitochondria(Uncertai		(M64)PID S1P S1P4 PA		0.0	0.0	0.0	1.0	0.0	0.0	0.0	0.0	0.0	0.0	0.0	0.0	0.0	0.0	0.0	0.0	0.0	0.0	0.0	0.0
NCLN	56926	symbol	H. sapiens	56926	H. sapiens	NCLN	nicalin	GO:0061635 regulation of protein			Lipid droplets;Nucleoplasm(Uncertain)				0.0	0.0	0.0	0.0	0.0	0.0	0.0	0.0	0.0	0.0	0.0	0.0	0.0	0.0	0.0	0.0	0.0	0.0	0.0	0.0
CELF5	60680	symbol	H. sapiens	60680	H. sapiens	CELF5	CUGBP Ela	GO:0006376 mRNA splice site selection;GO:0000381 regulation of alternative m							0.0	0.0	0.0	0.0	0.0	0.0	0.0	0.0	0.0	0.0	0.0	0.0	0.0	0.0	0.0	0.0	0.0	0.0	0.0	0.0
NFIC	4782	symbol	H. sapiens	4782	H. sapiens	NFIC	nuclear fac	GO:0042475 odontog	Transcriptic		Nucleus(Enhanced)Nu		(M285)PID HNF3A PAT		0.0	0.0	0.0	0.0	0.0	0.0	0.0	0.0	0.0	0.0	0.0	0.0	0.0	0.0	0.0	0.0	0.0	0.0	0.0	0.0
SMIM24	284422	symbol	H. sapiens	284422	H. sapiens	SMIM24	small integ	GO:0008150 biological_process			Cytosol;Nucleoplasm(Approved)				0.0	0.0	0.0	0.0	0.0	0.0	0.0	0.0	0.0	0.0	0.0	0.0	0.0	0.0	0.0	0.0	0.0	0.0	0.0	0.0
DOHH	83475	symbol	H. sapiens	83475	H. sapiens	DOHH	deoxyhypu	GO:0008612 peptidyl-		Enzymes/E	Cytosol;Nucleus(Approved)				0.0	0.0	0.0	0.0	0.0	0.0	0.0	0.0	0.0	0.0	0.0	0.0	0.0	0.0	0.0	0.0	0.0	0.0	0.0	0.0
FZR1	51343	symbol	H. sapiens	51343	H. sapiens	FZR1	fizzy and ce	GO:0072425 signal transduction i			Nuclear membrane;Nu		(M242)PID AURORA A		1.0	0.0	0.0	0.0	0.0	0.0	0.0	0.0	0.0	0.0	0.0	1.0	0.0	0.0	0.0	0.0	0.0	0.0	0.0	0.0
C7H19orf71						None	None	None	None	None	None	None	None	None	nan	nan	nan	nan	nan	nan	nan	nan	nan	nan	nan	nan	nan	nan	nan	nan	nan	nan	nan	nan
MFSD12	126321	symbol	H. sapiens	126321	H. sapiens	MFSD12	major facili	GO:0048022 negative regulation c			Mitochondria;Nucleoplasm(Approved)				0.0	0.0	0.0	0.0	0.0	0.0	0.0	0.0	0.0	0.0	0.0	0.0	0.0	0.0	0.0	0.0	0.0	0.0	0.0	0.0
HMG20B	10362	symbol	H. sapiens	10362	H. sapiens	HMG20B	high mobil	GO:0033234 negative	Transcriptic		Nucleoplasm(Enhanced)				0.0	0.0	0.0	0.0	0.0	0.0	0.0	0.0	0.0	0.0	0.0	0.0	0.0	0.0	0.0	0.0	0.0	0.0	0.0	0.0
GIPC3	126326	symbol	H. sapiens	126326	H. sapiens	GIPC3	GIPC PDZ domain containing family member				Golgi apparatus;Nuclear bodies;Nucleoplasm				0.0	0.0	0.0	0.0	0.0	0.0	0.0	0.0	0.0	0.0	0.0	0.0	0.0	0.0	0.0	0.0	0.0	0.0	0.0	0.0
TBXA2R	6915	symbol	H. sapiens	6915	H. sapiens	TBXA2R	thromboxa	GO:0090051 negative	G-protein c		Plasma me	Ridogrel (T	(M12645)ST GA13 PAT		1.0	0.0	0.0	1.0	0.0	0.0	0.0	0.0	0.0	0.0	0.0	0.0	0.0	0.0	0.0	0.0	0.0	0.0	0.0	0.0
CACTIN	58509	symbol	H. sapiens	58509	H. sapiens	CACTIN	cactin, splic	GO:0031665 negative regulation c			Nuclear speckles;Nucleoplasm(Supported)Cyt				1.0	0.0	0.0	0.0	0.0	0.0	0.0	0.0	0.0	0.0	0.0	0.0	0.0	0.0	0.0	0.0	0.0	0.0	0.0	0.0
PIP5K1C	23396	symbol	H. sapiens	23396	H. sapiens	PIP5K1C	phosphatic	GO:0030593 neutroph		Enzymes/E	Cytosol;Nucleoplasm(A		(M72)PID NECTIN PAT		0.0	0.0	0.0	0.0	1.0	0.0	0.0	0.0	0.0	0.0	0.0	0.0	0.0	0.0	1.0	0.0	0.0	0.0	0.0	0.0
TJP3	27134	symbol	H. sapiens	27134	H. sapiens	TJP3	tight junction protein 3				Cell Junctions(Enhanced)Nucleopla			(M5906)HA	0.0	0.0	0.0	0.0	0.0	0.0	0.0	0.0	0.0	0.0	0.0	0.0	0.0	0.0	0.0	0.0	0.0	0.0	0.0	0.0
APBA3	9546	symbol	H. sapiens	9546	H. sapiens	APBA3	amyloid be	GO:0001701 in utero embryonic c			Vesicles(Approved)				0.0	0.0	0.0	0.0	0.0	0.0	0.0	0.0	0.0	0.0	0.0	0.0	0.0	0.0	0.0	0.0	0.0	0.0	0.0	0.0
MRPL54	116541	symbol	H. sapiens	116541	H. sapiens	MRPL54	mitochond	GO:0070126 mitochondrial transl			Mitochondria(Approved)				0.0	0.0	0.0	0.0	0.0	1.0	0.0	0.0	0.0	0.0	0.0	0.0	0.0	0.0	0.0	0.0	0.0	0.0	0.0	0.0
RAX2	84839	symbol	H. sapiens	84839	H. sapiens	RAX2	retina and a	GO:0007601 visual pe	Transcription factors/Helix-turn-helix domains						0.0	0.0	0.0	0.0	0.0	0.0	0.0	0.0	0.0	0.0	0.0	0.0	0.0	0.0	0.0	0.0	0.0	0.0	0.0	0.0
MATK	4145	symbol	H. sapiens	4145	H. sapiens	MATK	megakaryo	GO:003812	Tyr protein	Enzymes/E	ENZYME proteins/Trans		(M231)PID KIT PATHW		0.0	0.0	0.0	0.0	0.0	0.0	0.0	0.0	0.0	0.0	0.0	0.0	0.0	0.0	1.0	0.0	0.0	0.0	0.0	0.0
ZFR2	23217	symbol	H. sapiens	23217	H. sapiens	ZFR2	zinc finger RNA binding protein 2				Nucleoplasm(Uncertain)				0.0	0.0	0.0	0.0	0.0	0.0	0.0	0.0	0.0	0.0	0.0	0.0	0.0	0.0	0.0	0.0	0.0	0.0	0.0	0.0
ATCAY	85300	symbol	H. sapiens	85300	H. sapiens	ATCAY	ATCAY kin	GO:2000212 negative regulation of glutamate metabolic process;GO:2000211 r							0.0	0.0	0.0	0.0	0.0	0.0	0.0	0.0	0.0	0.0	0.0	0.0	0.0	0.0	0.0	0.0	0.0	0.0	0.0	0.0
NMRK2	27231	symbol	H. sapiens	27231	H. sapiens	NMRK2	nicotinami	GO:0009435 NAD bio		Enzymes/E	Nucleoplasm;Vesicles(Enhanced)				0.0	0.0	0.0	0.0	0.0	0.0	0.0	0.0	0.0	0.0	1.0	0.0	0.0	0.0	0.0	0.0	0.0	0.0	0.0	0.0
DAPK3	1613	symbol	H. sapiens	1613	H. sapiens	DAPK3	death asso	GO:004351	CAMK Ser/	Enzymes/E	Nucleus(Ap	2-(1,1-DIMETHYLETHYL)9-FLUOR			0.0	1.0	0.0	0.0	0.0	0.0	0.0	1.0	0.0	0.0	0.0	1.0	0.0	0.0	0.0	0.0	0.0	0.0	0.0	0.0
EEF2	1938	symbol	H. sapiens	1938	H. sapiens	EEF2	eukaryotic	GO:1990416 cellular response to I			Cytosol;Pla	Adenosine	(M121)PID	(M5922)HA	0.0	0.0	0.0	0.0	0.0	1.0	0.0	1.0	0.0	0.0	0.0	0.0	0.0	0.0	0.0	0.0	0.0	0.0	0.0	0.0
PIAS4	51588	symbol	H. sapiens	51588	H. sapiens	PIAS4	protein inh	GO:1902174 positive r		Enzymes/E	Nucleoplasm(Approve		(M228)PID SMAD2 3P/		1.0	1.0	0.0	0.0	1.0	0.0	0.0	0.0	0.0	0.0	0.0	0.0	0.0	0.0	0.0	0.0	0.0	0.0	0.0	0.0
ZBTB7A	51341	symbol	H. sapiens	51341	H. sapiens	ZBTB7A	zinc finger	GO:0060766 negative	Transcriptic		Nucleoplasm(Approved)				1.0	0.0	0.0	0.0	0.0	0.0	0.0	0.0	0.0	0.0	0.0	0.0	0.0	0.0	0.0	0.0	0.0	0.0	1.0	0.0
MAP2K2	5605	symbol	H. sapiens	5605	H. sapiens	MAP2K2	mitogen-a	GO:19038	STE Ser/Th	Enzymes/E	Cytosol(Su	Bosutinib (	(M18895)SA TRKA REC		1.0	0.0	0.0	0.0	0.0	0.0	0.0	1.0	0.0	1.0	0.0	1.0	0.0	0.0	0.0	0.0	1.0	0.0	0.0	0.0
CREB3L3	84699	symbol	H. sapiens	84699	H. sapiens	CREB3L3	cAMP resp	GO:1990440 positive r	Transcription factors/Basic domains						1.0	0.0	0.0	0.0	0.0	0.0	0.0	0.0	0.0	1.0	0.0	0.0	0.0	0.0	0.0	0.0	0.0	0.0	0.0	0.0
SIRT6	51548	symbol	H. sapiens	51548	H. sapiens	SIRT6	sirtuin 6	GO:1905549 positive regulation o			Nucleoplasm(Supporte		(M101)PID	(M5921)HA	0.0	0.0	0.0	0.0	0.0	0.0	0.0	0.0	1.0	0.0	0.0	0.0	1.0	0.0	0.0	0.0	0.0	0.0	1.0	0.0
ANKRD24	170961	symbol	H. sapiens	170961	H. sapiens	ANKRD24	ankyrin repeat domain 24				Cytosol;Microtubules(Uncertain)				0.0	0.0	0.0	0.0	0.0	0.0	0.0	0.0	0.0	0.0	0.0	0.0	0.0	0.0	0.0	0.0	0.0	0.0	0.0	0.0
EBI3	10148	symbol	H. sapiens	10148	H. sapiens	EBI3	Epstein-Ba	GO:0046641 positive regulation of alpha-beta T cell prol					(M36)PID IL	(M5897)HA	0.0	0.0	0.0	0.0	0.0	0.0	0.0	0.0	0.0	0.0	0.0	0.0	0.0	0.0	0.0	0.0	0.0	0.0	0.0	0.0
CCDC94	55702	gene_synon	H. sapiens	55702	H. sapiens	YJU2	YJU2 splici	GO:0043518 negative regulation c			Nucleoplasm(Approved)				0.0	0.0	0.0	0.0	0.0	0.0	0.0	0.0	0.0	0.0	0.0	0.0	0.0	0.0	0.0	0.0	0.0	0.0	0.0	0.0
SHD	56961	symbol	H. sapiens	56961	H. sapiens	SHD	Src homology 2 domain containing transform				Cytosol;Microtubule organizing center(Uncert				0.0	0.0	0.0	0.0	0.0	0.0	0.0	0.0	0.0	0.0	0.0	0.0	0.0	0.0	0.0	0.0	0.0	0.0	0.0	0.0
TMIGD2	126259	symbol	H. sapiens	126259	H. sapiens	TMIGD2	transmemb	GO:0042104 positive regulation of activated T cell proliferation;GO:0031295 T c							0.0	0.0	0.0	0.0	0.0	0.0	0.0	0.0	0.0	0.0	0.0	0.0	0.0	0.0	0.0	0.0	0.0	0.0	0.0	0.0
FSD1	79187	symbol	H. sapiens	79187	H. sapiens	FSD1	fibronectin	GO:0060236 regulation of mitotic spindle organization;GO:0090224 regulation							0.0	0.0	0.0	0.0	0.0	0.0	0.0	0.0	0.0	0.0	0.0	1.0	0.0	0.0	0.0	0.0	0.0	0.0	0.0	0.0
STAP2	55620	symbol	H. sapiens	55620	H. sapiens	STAP2	signal trans	GO:0042531 positive regulation of tyrosine phosphorylation of STAT protein;GC							0.0	0.0	0.0	0.0	0.0	0.0	0.0	0.0	0.0	0.0	0.0	0.0	0.0	0.0	0.0	0.0	0.0	0.0	0.0	0.0
MPND	84954	symbol	H. sapiens	84954	H. sapiens	MPND	MPN doma	GO:0006508 proteolys		Enzymes/P	Nucleoli;Nucleus(Approved)				0.0	0.0	0.0	0.0	0.0	0.0	0.0	0.0	0.0	0.0	0.0	0.0	0.0	0.0	0.0	0.0	0.0	0.0	0.0	0.0
SH3GL1	6455	symbol	H. sapiens	6455	H. sapiens	SH3GL1	SH3 domai	GO:0016191 synaptic vesicle unco			Cytosol(Enhanced)		(M281)PID FAK PATHW		0.0	1.0	0.0	0.0	0.0	1.0	0.0	0.0	0.0	0.0	0.0	0.0	0.0	0.0	0.0	0.0	0.0	0.0	0.0	0.0
MFSD14B	84641	symbol	H. sapiens	84641	H. sapiens	MFSD14B	major facili	GO:0055085 transmer	Transporte		Cytosol;Nucleoplasm(Approved)				0.0	0.0	0.0	0.0	0.0	0.0	0.0	0.0	0.0	0.0	0.0	0.0	0.0	0.0	0.0	0.0	0.0	0.0	0.0	0.0
ANXA10	11199	symbol	H. sapiens	11199	H. sapiens	ANXA10	annexin A10				Nucleoli;Nucleus;Vesic		(M5880)NA	(M5953)HA	0.0	0.0	0.0	0.0	0.0	0.0	0.0	0.0	0.0	0.0	0.0	0.0	0.0	0.0	0.0	0.0	0.0	0.0	0.0	0.0
DDX60	55601	symbol	H. sapiens	55601	H. sapiens	DDX60	DExD/H-bo	GO:1900245 positive r		Enzymes/E	Cytosol(Supported)Intermediate fi			(M5911)HA	1.0	0.0	0.0	0.0	0.0	0.0	0.0	0.0	0.0	0.0	0.0	0.0	0.0	0.0	0.0	0.0	0.0	0.0	0.0	0.0
PALLD	23022	symbol	H. sapiens	23022	H. sapiens	PALLD	palladin, cy	GO:0003334 keratinocyte develop			Actin filaments;Cytosol(Supported			(M5893)HA	0.0	0.0	0.0	0.0	0.0	0.0	0.0	0.0	1.0	0.0	0.0	0.0	0.0	0.0	0.0	0.0	0.0	0.0	0.0	0.0
BCL11A	53335	symbol	H. sapiens	53335	H. sapiens	BCL11A	BAF chrom	GO:1904800 negative	Transcriptic		Nucleoplasm(Enhanced)Nuclear bodies(Suppo				0.0	0.0	0.0	0.0	0.0	0.0	0.0	0.0	1.0	0.0	0.0	0.0	1.0	0.0	0.0	0.0	0.0	0.0	0.0	0.0
PAPOLG	64895	symbol	H. sapiens	64895	H. sapiens	PAPOLG	poly(A) pol	GO:0006378 mRNA po		Enzymes/E	Nucleoplasm(Enhanced)				0.0	0.0	0.0	0.0	0.0	0.0	0.0	0.0	0.0	0.0	0.0	0.0	0.0	0.0	0.0	0.0	0.0	0.0	0.0	0.0
REL	5966	symbol	H. sapiens	5966	H. sapiens	REL	REL proto-	GO:0032688 negative	RAS pathway related proteins;Trar				(M26)PID N	(M5890)HA	1.0	0.0	0.0	0.0	0.0	0.0	0.0	0.0	0.0	0.0	0.0	0.0	0.0	0.0	0.0	0.0	0.0	0.0	0.0	0.0
PUS10	150962	symbol	H. sapiens	150962	H. sapiens	PUS10	pseudouric	GO:0031119 tRNA pse		Enzymes/E	Mitochondria;Nuclear bodies;Nucleoplasm(Ap				0.0	0.0	0.0	0.0	0.0	0.0	0.0	0.0	0.0	0.0	0.0	0.0	0.0	0.0	0.0	0.0	0.0	0.0	0.0	0.0
PEX13	5194	symbol	H. sapiens	5194	H. sapiens	PEX13	peroxisom	GO:0016560 protein ir	Transporte		Vesicles(Supported)			(M5949)HA	0.0	0.0	0.0	0.0	1.0	0.0	0.0	0.0	0.0	0.0	0.0	0.0	0.0	1.0	0.0	0.0	0.0	0.0	1.0	0.0
KIAA1841	84542	symbol	H. sapiens	84542	H. sapiens	KIAA1841	KIAA1841				Nucleoli;Nucleus(Approved)				0.0	0.0	0.0	0.0	0.0	0.0	0.0	0.0	0.0	0.0	0.0	0.0	0.0	0.0	0.0	0.0	0.0	0.0	0.0	0.0
GCFC2	6936	symbol	H. sapiens	6936	H. sapiens	GCFC2	GC-rich se	GO:0000245 spliceosomal compl			Nucleoplasm(Supported)				0.0	0.0	0.0	0.0	0.0	0.0	0.0	0.0	0.0	0.0	0.0	0.0	0.0	0.0	0.0	0.0	0.0	0.0	0.0	0.0
MRPL19	9801	symbol	H. sapiens	9801	H. sapiens	MRPL19	mitochond	GO:0070126 mitochor		Ribosomal	Mitochondria(Supported)				0.0	0.0	0.0	0.0	0.0	1.0	0.0	0.0	0.0	0.0	0.0	0.0	0.0	0.0	0.0	0.0	0.0	0.0	0.0	0.0
EVA1A	84141	symbol	H. sapiens	84141	H. sapiens	EVA1A	eva-1 hom	GO:0043687 post-translational pr			Vesicles(Supported)Plasma membrane(Appro				0.0	0.0	0.0	0.0	0.0	0.0	0.0	0.0	0.0	0.0	0.0	0.0	0.0	0.0	0.0	0.0	0.0	0.0	0.0	0.0
PTCD3	55037	symbol	H. sapiens	55037	H. sapiens	PTCD3	pentatricop	GO:0070126 mitochor		Ribosomal	Mitochondria(Enhanced)			(M5905)HA	0.0	0.0	0.0	0.0	0.0	1.0	0.0	0.0	0.0	0.0	0.0	0.0	0.0	0.0	0.0	0.0	0.0	0.0	0.0	0.0
POLR1A	25885	symbol	H. sapiens	25885	H. sapiens	POLR1A	RNA polym	GO:1904750 negative		Enzymes/E	Nucleoli fibrillar center;Nucleus(Supported)				1.0	0.0	0.0	0.0	0.0	0.0	0.0	0.0	0.0	0.0	0.0	0.0	0.0	0.0	0.0	0.0	0.0	0.0	0.0	1.0
ST3GAL5	8869	symbol	H. sapiens	8869	H. sapiens	ST3GAL5	ST3 beta-g	GO:0001574 gangliosi		Enzymes/E	Vesicles(Ap	99mTc-14 F7 Mab (Sia		(M5913)HA	0.0	0.0	0.0	0.0	0.0	0.0	0.0	0.0	0.0	0.0	0.0	0.0	0.0	0.0	0.0	0.0	0.0	0.0	0.0	0.0
ATOH8	84913	symbol	H. sapiens	84913	H. sapiens	ATOH8	atonal bHL	GO:0045603 positive r	Transcriptic		Nucleoplasm(Supported)			(M5934)HA	0.0	0.0	0.0	0.0	0.0	0.0	0.0	0.0	0.0	0.0	0.0	0.0	1.0	0.0	0.0	0.0	0.0	1.0	0.0	0.0
GNLY	10578	symbol	H. sapiens	10578	H. sapiens	GNLY	granulysin	GO:0006968 cellular d	Transporters/Transport			3[N-Morpholino]Propane Sulfonic			0.0	0.0	0.0	0.0	0.0	0.0	0.0	0.0	0.0	0.0	0.0	0.0	0.0	0.0	0.0	0.0	0.0	0.0	0.0	0.0
SFTPB	6439	symbol	H. sapiens	6439	H. sapiens	SFTPB	surfactant p	GO:0007585 respiratory gaseous exchange;GO:0006665					(M5880)NABA ECM AF		0.0	0.0	0.0	0.0	0.0	0.0	0.0	0.0	0.0	0.0	0.0	0.0	0.0	0.0	0.0	0.0	0.0	0.0	0.0	0.0
USP39	10713	symbol	H. sapiens	10713	H. sapiens	USP39	ubiquitin s	GO:0000245 spliceosc		Enzymes/P	Nucleoplasm(Supported)				0.0	0.0	0.0	0.0	0.0	0.0	0.0	0.0	0.0	0.0	0.0	0.0	0.0	0.0	0.0	0.0	0.0	0.0	0.0	0.0
C11H2orf68						None	None	None	None	None	None	None	None	None	nan	nan	nan	nan	nan	nan	nan	nan	nan	nan	nan	nan	nan	nan	nan	nan	nan	nan	nan	nan
TMEM150A	129303	symbol	H. sapiens	129303	H. sapiens	TMEM150A	transmemb	GO:0046854 phosphatidylinositol			Nucleoplasm;Vesicles(Approved)				0.0	0.0	0.0	0.0	1.0	0.0	0.0	0.0	0.0	0.0	0.0	0.0	0.0	0.0	0.0	0.0	0.0	0.0	0.0	0.0
RNF181	51255	symbol	H. sapiens	51255	H. sapiens	RNF181	ring finger	GO:0051865 protein a		Enzymes/E	Cytosol;Nucleoplasm(Approved)				0.0	0.0	0.0	0.0	0.0	0.0	0.0	0.0	0.0	0.0	0.0	0.0	0.0	1.0	0.0	0.0	0.0	0.0	0.0	0.0
VAMP5	10791	symbol	H. sapiens	10791	H. sapiens	VAMP5	vesicle asso	GO:0043001 Golgi to plasma membrane protein transport;GO:0061						(M5913)HA	0.0	0.0	0.0	0.0	0.0	0.0	1.0	0.0	0.0	0.0	0.0	0.0	0.0	0.0	0.0	0.0	0.0	0.0	0.0	0.0
VAMP8	8673	symbol	H. sapiens	8673	H. sapiens	VAMP8	vesicle asso	GO:1903595 positive r	Transporters/Transporter channels and pores					(M5939)HA	1.0	0.0	0.0	0.0	0.0	1.0	1.0	0.0	0.0	0.0	0.0	0.0	0.0	0.0	0.0	0.0	0.0	0.0	0.0	0.0
GGCX	2677	symbol	H. sapiens	2677	H. sapiens	GGCX	gamma-gl	GO:0017187 peptidyl-		Enzymes/ENZYME pro		Coagulation factor VIIa Recombin			0.0	0.0	0.0	0.0	0.0	0.0	0.0	0.0	0.0	0.0	0.0	0.0	0.0	0.0	0.0	0.0	0.0	0.0	0.0	0.0
MAT2A	4144	symbol	H. sapiens	4144	H. sapiens	MAT2A	methionine	GO:0006556 S-adeno		Enzymes/E	Cytosol;Nu	S-Adenosy	(M195)PID CMYB PATH		0.0	0.0	0.0	0.0	0.0	0.0	0.0	0.0	0.0	0.0	0.0	0.0	0.0	0.0	0.0	0.0	0.0	0.0	0.0	0.0
SH2D6	284948	symbol	H. sapiens	284948	H. sapiens	SH2D6	SH2 domai	GO:0007169 transmembrane receptor protein tyrosine kinase signaling pathway							0.0	0.0	0.0	0.0	0.0	0.0	0.0	0.0	0.0	0.0	0.0	0.0	0.0	0.0	0.0	0.0	0.0	0.0	0.0	0.0
CAPG	822	symbol	H. sapiens	822	H. sapiens	CAPG	capping ac	GO:0051016 barbed-end actin fil			Nucleus(Enhanced)			(M5947)HA	0.0	0.0	0.0	0.0	0.0	1.0	0.0	0.0	0.0	0.0	0.0	0.0	0.0	0.0	0.0	0.0	0.0	0.0	0.0	0.0
ELMOD3	84173	symbol	H. sapiens	84173	H. sapiens	ELMOD3	ELMO dom	GO:0043547 positive regulation o			Mitochondria;Nucleoplasm(Appro			(M5905)HA	0.0	0.0	0.0	0.0	0.0	0.0	0.0	0.0	0.0	0.0	0.0	0.0	0.0	0.0	0.0	0.0	0.0	0.0	0.0	0.0
RETSAT	54884	symbol	H. sapiens	54884	H. sapiens	RETSAT	retinol satu	GO:0042572 retinol m		Enzymes/E	Nucleoli(Ap	Vitamin A (Oxidoreduc		(M5949)HA	0.0	0.0	0.0	0.0	0.0	0.0	0.0	0.0	0.0	0.0	0.0	0.0	0.0	0.0	0.0	0.0	0.0	0.0	0.0	0.0
TGOLN2	10618	symbol	H. sapiens	10618	H. sapiens	TGOLN2	trans-golgi	GO:0043687 post-translational pr			Golgi apparatus;Nucleoplasm(Enhanced)				0.0	0.0	0.0	0.0	0.0	0.0	0.0	0.0	0.0	0.0	0.0	0.0	0.0	0.0	0.0	0.0	0.0	0.0	0.0	0.0
TCF7L1	83439	symbol	H. sapiens	83439	H. sapiens	TCF7L1	transcriptic	GO:1904837 beta-cate	Transcriptic		Nucleoplasm(Supporte		(M236)PID	(M5956)HA	1.0	0.0	0.0	0.0	0.0	0.0	0.0	0.0	1.0	0.0	0.0	0.0	0.0	0.0	0.0	0.0	0.0	0.0	0.0	0.0
PDCL	5082	symbol	H. sapiens	5082	H. sapiens	PDCL	phosducin	GO:0061084 negative regulation c			Cytosol(Supported)				0.0	0.0	0.0	0.0	0.0	0.0	0.0	0.0	0.0	0.0	0.0	0.0	0.0	0.0	0.0	0.0	0.0	0.0	0.0	0.0
RC3H2	54542	symbol	H. sapiens	54542	H. sapiens	RC3H2	ring finger	GO:2000320 negative		Enzymes/E	Vesicles(Supported)				1.0	0.0	0.0	0.0	0.0	0.0	0.0	0.0	1.0	0.0	0.0	0.0	0.0	0.0	0.0	0.0	0.0	0.0	0.0	0.0
ZBTB6	10773	symbol	H. sapiens	10773	H. sapiens	ZBTB6	zinc finger	GO:0000122 negative	Transcriptic		Nucleoplasm(Supported)Mitochondria(Appro				0.0	0.0	0.0	0.0	0.0	0.0	0.0	0.0	0.0	0.0	0.0	0.0	0.0	0.0	0.0	0.0	0.0	0.0	0.0	0.0
ZBTB26	57684	symbol	H. sapiens	57684	H. sapiens	ZBTB26	zinc finger	GO:0000122 negative	Transcription factors/Zinc-coordinating DNA-binding dc						0.0	0.0	0.0	0.0	0.0	0.0	0.0	0.0	0.0	0.0	0.0	0.0	0.0	0.0	0.0	0.0	0.0	0.0	0.0	0.0
RABGAP1	23637	symbol	H. sapiens	23637	H. sapiens	RABGAP1	RAB GTPas	GO:1902017 regulation of cilium a			Cytosol(Enhanced)			(M5893)HA	0.0	0.0	0.0	0.0	0.0	0.0	0.0	0.0	0.0	0.0	0.0	0.0	0.0	0.0	0.0	0.0	0.0	0.0	0.0	0.0
GPR21	2844	symbol	H. sapiens	2844	H. sapiens	GPR21	G protein-c	GO:0046627 negative regulation c			Cytosol(Enhanced)				0.0	0.0	0.0	0.0	0.0	0.0	0.0	0.0	1.0	0.0	0.0	0.0	0.0	0.0	0.0	0.0	0.0	0.0	0.0	0.0
STRBP	55342	symbol	H. sapiens	55342	H. sapiens	STRBP	spermatid p	GO:0007286 spermatid development;GO:0007638 mechanosensory						(M5951)HA	0.0	0.0	0.0	0.0	0.0	0.0	0.0	0.0	0.0	0.0	0.0	0.0	0.0	0.0	0.0	0.0	0.0	0.0	0.0	0.0
CRB2	286204	symbol	H. sapiens	286204	H. sapiens	CRB2	crumbs cel	GO:0055111 ingression involved in gastrulation with mouth forming second;GC							0.0	1.0	0.0	0.0	0.0	0.0	0.0	0.0	0.0	0.0	0.0	0.0	0.0	0.0	0.0	0.0	0.0	1.0	0.0	0.0
DENND1A	57706	symbol	H. sapiens	57706	H. sapiens	DENND1A	DENN dom	GO:0032483 regulation of Rab pr			Vesicles(Supported)Cytosol;Nucleoplasm(App				0.0	0.0	0.0	0.0	0.0	0.0	0.0	0.0	0.0	0.0	0.0	0.0	0.0	0.0	0.0	0.0	0.0	0.0	0.0	0.0
LHX2	9355	symbol	H. sapiens	9355	H. sapiens	LHX2	LIM homec	GO:0045199 mainten	Transcriptic		Nucleoplasm(Approved)			(M5941)HA	0.0	1.0	0.0	0.0	0.0	0.0	0.0	0.0	1.0	0.0	0.0	0.0	1.0	0.0	0.0	0.0	0.0	1.0	0.0	0.0
NEK6	10783	symbol	H. sapiens	10783	H. sapiens	NEK6	NIMA relat	GO:00070	NEK Ser/Th	Enzymes/E	Cytosol;Microtubule organizing center;Nuclec				1.0	0.0	0.0	0.0	0.0	0.0	0.0	0.0	0.0	0.0	0.0	1.0	0.0	0.0	0.0	0.0	0.0	0.0	0.0	0.0
PSMB7	5695	symbol	H. sapiens	5695	H. sapiens	PSMB7	proteasom	GO:0002223 stimulatc		Enzymes/E	Cytosol;Nu	(3AR,6R,6AS)-6-((S)-((S)-CYCLOH			1.0	1.0	0.0	0.0	0.0	0.0	0.0	0.0	0.0	0.0	0.0	1.0	0.0	0.0	0.0	0.0	0.0	0.0	0.0	0.0
PAEP	5047	symbol	H. sapiens	5047	H. sapiens	PAEP	progestage	GO:1902491 negative regulation of sperm ca				12-Bromododecanoic Acid (Small			0.0	0.0	0.0	0.0	0.0	0.0	0.0	0.0	0.0	0.0	0.0	0.0	0.0	0.0	0.0	0.0	0.0	0.0	0.0	0.0
GLT6D1	360203	symbol	H. sapiens	360203	H. sapiens	GLT6D1	glycosyltra	GO:0030259 lipid glycosylation;GO:0030258				Uridine diphosphate glucose (Trar			0.0	0.0	0.0	0.0	1.0	0.0	0.0	0.0	0.0	0.0	0.0	0.0	0.0	0.0	0.0	0.0	0.0	0.0	0.0	0.0
LCN9	392399	symbol	H. sapiens	392399	H. sapiens	LCN9	lipocalin 9					2-Methoxy-3-Isopropylpyrazine (			0.0	0.0	0.0	0.0	0.0	0.0	0.0	0.0	0.0	0.0	0.0	0.0	0.0	0.0	0.0	0.0	0.0	0.0	0.0	0.0
SOHLH1	402381	symbol	H. sapiens	402381	H. sapiens	SOHLH1	spermatog	GO:0009994 oocyte d	Transcriptic		Nuclear speckles(Approved)				0.0	0.0	0.0	0.0	0.0	0.0	0.0	0.0	0.0	0.0	0.0	0.0	0.0	0.0	0.0	0.0	0.0	0.0	0.0	0.0
KCNT1	57582	symbol	H. sapiens	57582	H. sapiens	KCNT1	potassium	GO:0071805 potassiu	Transporters/Transporter channels and pores;Voltage-ga						0.0	0.0	0.0	0.0	0.0	0.0	0.0	0.0	0.0	0.0	0.0	0.0	0.0	0.0	0.0	0.0	0.0	0.0	0.0	0.0
CAMSAP1	157922	symbol	H. sapiens	157922	H. sapiens	CAMSAP1	calmodulin	GO:0007026 negative regulation c			Cytosol;Mitotic spindle(Supported)				0.0	0.0	0.0	0.0	0.0	1.0	0.0	0.0	0.0	0.0	0.0	0.0	0.0	0.0	0.0	0.0	0.0	0.0	0.0	0.0
UBAC1	10422	symbol	H. sapiens	10422	H. sapiens	UBAC1	UBA doma	GO:0016567 protein ubiquitinatio			Cytosol(Supported)Golgi apparatu			(M5945)HA	1.0	0.0	0.0	0.0	0.0	0.0	0.0	0.0	0.0	0.0	0.0	0.0	0.0	0.0	0.0	0.0	0.0	0.0	0.0	0.0
NACC2	138151	symbol	H. sapiens	138151	H. sapiens	NACC2	NACC fami	GO:1900477 negative regulation c			Mitochondria;Nucleoli(Approved)				0.0	0.0	0.0	0.0	0.0	0.0	0.0	0.0	0.0	0.0	0.0	1.0	0.0	0.0	0.0	0.0	0.0	0.0	0.0	0.0
C11H9orf69						None	None	None	None	None	None	None	None	None	nan	nan	nan	nan	nan	nan	nan	nan	nan	nan	nan	nan	nan	nan	nan	nan	nan	nan	nan	nan
CCDC187	399693	symbol	H. sapiens	399693	H. sapiens	CCDC187	coiled-coil	GO:0034453 microtubule anchoring;GO:0000226 microtubule cytoskeleton org							0.0	0.0	0.0	0.0	0.0	0.0	0.0	0.0	0.0	0.0	0.0	0.0	0.0	0.0	0.0	0.0	0.0	0.0	0.0	0.0
GPSM1	26086	symbol	H. sapiens	26086	H. sapiens	GPSM1	G protein s	GO:1905098 negative regulation c			Golgi apparatus(Supported)Nucleoplasm(App				0.0	0.0	0.0	0.0	0.0	0.0	0.0	0.0	0.0	0.0	0.0	0.0	0.0	0.0	0.0	0.0	0.0	0.0	0.0	0.0
DNLZ	728489	symbol	H. sapiens	728489	H. sapiens	DNLZ	DNL-type z	GO:0030150 protein import into r			Mitochondria(Enhanced)Nucleoplasm(Approv				0.0	0.0	0.0	0.0	0.0	0.0	0.0	0.0	0.0	0.0	0.0	0.0	0.0	0.0	0.0	0.0	0.0	0.0	0.0	0.0
CARD9	64170	symbol	H. sapiens	64170	H. sapiens	CARD9	caspase rec	GO:0002223 stimulatory C-type lectin receptor signaling pathway;GO:0002220							1.0	0.0	0.0	0.0	0.0	0.0	0.0	0.0	0.0	0.0	0.0	0.0	0.0	0.0	0.0	0.0	0.0	0.0	0.0	0.0
SNAPC4	6621	symbol	H. sapiens	6621	H. sapiens	SNAPC4	small nucle	GO:0042796 snRNA tr	Transcriptic		Nuclear membrane;Nucleoplasm(			(M5898)HA	0.0	0.0	0.0	0.0	0.0	0.0	0.0	0.0	0.0	0.0	0.0	0.0	0.0	0.0	0.0	0.0	0.0	0.0	0.0	0.0
SDCCAG3	10807	gene_synon	H. sapiens	10807	H. sapiens	ENTR1	endosome	GO:1903566 positive regulation of protein localization to cilium;GO:1903564 re							0.0	0.0	0.0	0.0	0.0	0.0	1.0	0.0	0.0	0.0	0.0	0.0	0.0	0.0	0.0	0.0	0.0	0.0	0.0	0.0
PMPCA	23203	symbol	H. sapiens	23203	H. sapiens	PMPCA	peptidase,	GO:0006627 protein p		Enzymes/E	Mitochondria(Enhanced)			(M5936)HA	0.0	0.0	0.0	0.0	0.0	0.0	0.0	0.0	0.0	0.0	0.0	0.0	0.0	1.0	0.0	0.0	0.0	0.0	0.0	0.0
INPP5E	56623	symbol	H. sapiens	56623	H. sapiens	INPP5E	inositol pol	GO:1903565 negative		Enzymes/ENZYME proteins/Hydrolases					0.0	0.0	0.0	0.0	1.0	0.0	0.0	0.0	0.0	0.0	0.0	0.0	0.0	0.0	0.0	0.0	0.0	0.0	0.0	0.0
SEC16A	9919	symbol	H. sapiens	9919	H. sapiens	SEC16A	SEC16 hom	GO:0043000 Golgi to plasma me			Endoplasmic reticulum;Golgi apparatus(Supp				1.0	0.0	0.0	0.0	0.0	0.0	1.0	0.0	0.0	0.0	0.0	0.0	0.0	0.0	0.0	0.0	0.0	0.0	0.0	0.0
NOTCH1	4851	symbol	H. sapiens	4851	H. sapiens	NOTCH1	notch rece	GO:0003270 Notch signaling pat			Nucleoplasm(Approve		(M70)PID P	(M5903)HA	1.0	1.0	0.0	0.0	0.0	0.0	0.0	0.0	1.0	0.0	1.0	0.0	1.0	0.0	0.0	0.0	0.0	1.0	0.0	0.0
EGFL7	51162	symbol	H. sapiens	51162	H. sapiens	EGFL7	EGF like do	GO:0001570 vasculogenesis;GO:0045746 negative regul					(M5883)NABA SECRET		0.0	0.0	0.0	0.0	0.0	0.0	0.0	0.0	0.0	0.0	0.0	0.0	1.0	0.0	0.0	0.0	0.0	0.0	0.0	0.0
MIR126	406913	symbol	H. sapiens	406913	H. sapiens	MIR126	microRNA	GO:1903589 positive regulation of blood vessel endothelial cell proliferation inv							1.0	0.0	0.0	0.0	0.0	0.0	0.0	0.0	0.0	0.0	0.0	0.0	1.0	0.0	1.0	0.0	0.0	0.0	0.0	0.0
AGPAT2	10555	symbol	H. sapiens	10555	H. sapiens	AGPAT2	1-acylglyc	GO:0016024 CDP-dia		Enzymes/E	Centrosome(Approved)				0.0	0.0	0.0	0.0	1.0	0.0	0.0	0.0	0.0	0.0	0.0	0.0	0.0	0.0	0.0	0.0	0.0	0.0	0.0	0.0
FAM69B	138311	gene_synon	H. sapiens	138311	H. sapiens	DIPK1B	divergent protein kinase domain 1B				Nuclear bodies;Nucleus;Vesicles(Approved)				0.0	0.0	0.0	0.0	0.0	0.0	0.0	0.0	0.0	0.0	0.0	0.0	0.0	0.0	0.0	0.0	0.0	0.0	0.0	0.0
ABO	28	symbol	H. sapiens	28	H. sapiens	ABO	ABO, alpha	GO:0030259 lipid glycosylation;GO:0006486				Uridine-Diphosphate-N-Acetylga			0.0	0.0	0.0	0.0	1.0	0.0	0.0	0.0	0.0	0.0	0.0	0.0	0.0	0.0	0.0	0.0	0.0	0.0	0.0	0.0
SURF6	6838	symbol	H. sapiens	6838	H. sapiens	SURF6	surfeit 6	GO:0042274 ribosomal small sub			Nucleoli(Enhanced)Nucleus(Approved)				0.0	0.0	0.0	0.0	0.0	0.0	0.0	0.0	0.0	0.0	0.0	0.0	0.0	0.0	0.0	0.0	0.0	0.0	0.0	0.0
MED22	6837	symbol	H. sapiens	6837	H. sapiens	MED22	mediator c	GO:0006357 regulation of transcri			Nucleoplasm(Approved)				0.0	0.0	0.0	0.0	1.0	0.0	0.0	0.0	0.0	0.0	0.0	0.0	0.0	0.0	0.0	0.0	0.0	0.0	0.0	0.0
RPL7A	6130	symbol	H. sapiens	6130	H. sapiens	RPL7A	ribosomal p	GO:0006614 SRP-dep	Ribosomal		Nucleoli;Vesicles(Approved)				0.0	0.0	0.0	0.0	0.0	1.0	0.0	0.0	0.0	0.0	0.0	0.0	0.0	0.0	0.0	0.0	0.0	0.0	0.0	0.0
SURF2	6835	symbol	H. sapiens	6835	H. sapiens	SURF2	surfeit 2	GO:0008150 biological_process			Nucleoli;Nucleus(Enhanced)				0.0	0.0	0.0	0.0	0.0	0.0	0.0	0.0	0.0	0.0	0.0	0.0	0.0	0.0	0.0	0.0	0.0	0.0	0.0	0.0
SURF4	6836	symbol	H. sapiens	6836	H. sapiens	SURF4	surfeit 4	GO:0034498 early end	Transporters						0.0	0.0	0.0	0.0	0.0	0.0	0.0	0.0	0.0	0.0	0.0	0.0	0.0	0.0	0.0	0.0	0.0	0.0	0.0	0.0
STKLD1	169436	symbol	H. sapiens	169436	H. sapiens	STKLD1	serine/thre	GO:00064	Other	Enzymes/Kinases					0.0	0.0	0.0	0.0	0.0	0.0	0.0	0.0	0.0	0.0	0.0	0.0	0.0	0.0	0.0	0.0	0.0	0.0	0.0	0.0
REXO4	57109	symbol	H. sapiens	57109	H. sapiens	REXO4	REX4 hom	GO:0000738 DNA catabolic proce			Nucleoli;Nucleus(Supported)				0.0	0.0	0.0	0.0	0.0	0.0	0.0	0.0	0.0	0.0	0.0	0.0	0.0	0.0	0.0	0.0	0.0	0.0	0.0	0.0
ADAMTS13	11093	symbol	H. sapiens	11093	H. sapiens	ADAMTS13	ADAM met	GO:0035864 response		Enzymes/ENZYME proteins/Hydro			(M3468)NABA ECM RE		1.0	0.0	0.0	0.0	0.0	0.0	0.0	0.0	0.0	0.0	0.0	0.0	0.0	0.0	0.0	0.0	0.0	0.0	0.0	0.0
CACFD1	11094	symbol	H. sapiens	11094	H. sapiens	CACFD1	calcium ch	GO:0016192 vesicle-m	Transporte		Nucleoplasm(Approved)				0.0	0.0	0.0	0.0	0.0	0.0	0.0	0.0	0.0	0.0	0.0	0.0	0.0	0.0	0.0	0.0	0.0	0.0	0.0	0.0
TNFRSF19	55504	symbol	H. sapiens	55504	H. sapiens	TNFRSF19	TNF recept	GO:0046330 positive regulation o			Mitochondria;Nucleoplasm(Approved)				1.0	0.0	0.0	0.0	0.0	0.0	0.0	0.0	0.0	0.0	0.0	0.0	0.0	0.0	0.0	0.0	0.0	0.0	0.0	0.0
MIPEP	4285	symbol	H. sapiens	4285	H. sapiens	MIPEP	mitochond	GO:0006627 protein p		Enzymes/E	Mitochondria(Enhanced)				0.0	0.0	0.0	0.0	0.0	0.0	0.0	0.0	0.0	0.0	0.0	0.0	0.0	0.0	0.0	0.0	0.0	0.0	0.0	0.0
C1QTNF9	338872	symbol	H. sapiens	338872	H. sapiens	C1QTNF9	C1q and TI	GO:0007165 signal transduction;GO:0023052 signaling;G					(M5880)NABA ECM AF		0.0	0.0	0.0	0.0	0.0	0.0	0.0	0.0	0.0	0.0	0.0	0.0	0.0	0.0	0.0	0.0	0.0	0.0	0.0	0.0
SPATA13	221178	symbol	H. sapiens	221178	H. sapiens	SPATA13	spermatog	GO:0030032 lamellipodium assen			Cytosol;Nucleoplasm(S		(M83)PID CDC42 REG		0.0	0.0	0.0	0.0	0.0	0.0	0.0	0.0	0.0	0.0	0.0	0.0	0.0	0.0	0.0	0.0	0.0	0.0	0.0	0.0
AMER2	219287	symbol	H. sapiens	219287	H. sapiens	AMER2	APC memb	GO:0090090 negative regulation of canonical Wnt signaling pathway;GO:00073							0.0	1.0	0.0	0.0	0.0	0.0	0.0	0.0	0.0	0.0	0.0	0.0	0.0	0.0	0.0	0.0	0.0	0.0	0.0	0.0
MTMR6	9107	symbol	H. sapiens	9107	H. sapiens	MTMR6	myotubula	GO:0046856 phosphatidylinositol dephosphorylation;GO:0006661 phosphatidy							0.0	0.0	0.0	0.0	1.0	0.0	0.0	0.0	0.0	0.0	0.0	0.0	0.0	0.0	0.0	0.0	0.0	0.0	0.0	0.0
NUP58	9818	symbol	H. sapiens	9818	H. sapiens	NUP58	nucleopori	GO:0006409 tRNA exp	Transporte		Cytosol;Mitochondria;Plasma mer			(M5941)HA	0.0	0.0	0.0	0.0	0.0	0.0	0.0	0.0	0.0	0.0	0.0	0.0	0.0	0.0	0.0	0.0	0.0	0.0	1.0	0.0
TRNAG-UC	108350327	symbol	R. norvegicus			None	None	None	None	None	None	None	None	None	nan	nan	nan	nan	nan	nan	nan	nan	nan	nan	nan	nan	nan	nan	nan	nan	nan	nan	nan	nan
ATP8A2	51761	symbol	H. sapiens	51761	H. sapiens	ATP8A2	ATPase ph	GO:0061092 positive r		Enzymes/E	Plasma membrane(Supported)Nucleoplasm(L				0.0	0.0	0.0	0.0	0.0	0.0	0.0	0.0	1.0	0.0	0.0	0.0	0.0	0.0	0.0	0.0	0.0	0.0	1.0	0.0
MTDH	92140	symbol	H. sapiens	92140	H. sapiens	MTDH	metadherin	GO:0031663 lipopolysaccharide-r			Endoplasmic reticulum		(M66)PID MYC ACTIV		1.0	0.0	0.0	0.0	0.0	0.0	0.0	0.0	0.0	0.0	0.0	0.0	0.0	1.0	0.0	0.0	0.0	0.0	0.0	0.0
LAPTM4B	55353	symbol	H. sapiens	55353	H. sapiens	LAPTM4B	lysosomal p	GO:1905166 negative	Transporte		Golgi apparatus(Approved)				[illegible]	[illegible]	[illegible]	[illegible]	[illegible]	[illegible]	[illegible]	[illegible]	[illegible]	[illegible]	[illegible]	[illegible]	[illegible]	[illegible]	[illegible]	[illegible]	[illegible]	[illegible]	[illegible]	[illegible]
MATN2	4147	symbol	H. sapiens	4147	H. sapiens	MATN2	matrilin 2	GO:0003429 growth plate cartilage chondrocyte morph					(M3008)NA	(M5930)HA	[illegible]	[illegible]	[illegible]	[illegible]	[illegible]	[illegible]	[illegible]	[illegible]	[illegible]	[illegible]	[illegible]	[illegible]	[illegible]	[illegible]	[illegible]	[illegible]	[illegible]	[illegible]	[illegible]	[illegible]
RPL30	6156	symbol	H. sapiens	6156	H. sapiens	RPL30	ribosomal p	GO:1904571 positive r	Ribosomal		Endoplasmic reticulum(Approved)				[illegible]	[illegible]	[illegible]	[illegible]	[illegible]	[illegible]	[illegible]	[illegible]	[illegible]	[illegible]	[illegible]	[illegible]	[illegible]	[illegible]	[illegible]	[illegible]	[illegible]	[illegible]	[illegible]	[illegible]
ERICH5	203111	symbol	H. sapiens	203111	H. sapiens	ERICH5	glutamate rich 5				Cytosol;Golgi apparatus;Nucleoplasm(Approved)				[illegible]	[illegible]	[illegible]	[illegible]	[illegible]	[illegible]	[illegible]	[illegible]	[illegible]	[illegible]	[illegible]	[illegible]	[illegible]	[illegible]	[illegible]	[illegible]	[illegible]	[illegible]	[illegible]	[illegible]
RIDA	10247	symbol	H. sapiens	10247	H. sapiens	RIDA	reactive int	GO:0019518 L-threonine catabolic process to				Benzoic Acid (Xenon atom bindin			[illegible]	[illegible]	[illegible]	[illegible]	[illegible]	[illegible]	[illegible]	[illegible]	[illegible]	[illegible]	[illegible]	[illegible]	[illegible]	[illegible]	[illegible]	[illegible]	[illegible]	[illegible]	[illegible]	[illegible]
POP1	10940	symbol	H. sapiens	10940	H. sapiens	POP1	POP1 hom	GO:0001682 tRNA 5'-l		Enzymes/E	Nucleoli;Nucleoplasm(Supported)				[illegible]	[illegible]	[illegible]	[illegible]	[illegible]	[illegible]	[illegible]	[illegible]	[illegible]	[illegible]	[illegible]	[illegible]	[illegible]	[illegible]	[illegible]	[illegible]	[illegible]	[illegible]	[illegible]	[illegible]
NIPAL2	79815	symbol	H. sapiens	79815	H. sapiens	NIPAL2	NIPA like d	GO:1903830 magnesium ion transmembrane transport;GO:0015693 magnesiu							[illegible]	[illegible]	[illegible]	[illegible]	[illegible]	[illegible]	[illegible]	[illegible]	[illegible]	[illegible]	[illegible]	[illegible]	[illegible]	[illegible]	[illegible]	[illegible]	[illegible]	[illegible]	[illegible]	[illegible]
KCNS2	3788	symbol	H. sapiens	3788	H. sapiens	KCNS2	potassium	GO:1902259 regulation of	Transporters/Transporter channels and pores;Voltage-ga						[illegible]	[illegible]	[illegible]	[illegible]	[illegible]	[illegible]	[illegible]	[illegible]	[illegible]	[illegible]	[illegible]	[illegible]	[illegible]	[illegible]	[illegible]	[illegible]	[illegible]	[illegible]	[illegible]	[illegible]
STK3	6788	symbol	H. sapiens	6788	H. sapiens	STK3	serine/thre	GO:00060	STE Ser/Th	Enzymes/E	Intermediate filaments;Rods & Rings(Approved)				[illegible]	[illegible]	[illegible]	[illegible]	[illegible]	[illegible]	[illegible]	[illegible]	[illegible]	[illegible]	[illegible]	[illegible]	[illegible]	[illegible]	[illegible]	[illegible]	[illegible]	[illegible]	[illegible]	[illegible]
DCLK3	85443	symbol	H. sapiens	85443	H. sapiens	DCLK3	doublecort	GO:19001	CAMK Ser/T	Enzymes/ENZYME proteins/Transferases,Kinases/CAMK					[illegible]	[illegible]	[illegible]	[illegible]	[illegible]	[illegible]	[illegible]	[illegible]	[illegible]	[illegible]	[illegible]	[illegible]	[illegible]	[illegible]	[illegible]	[illegible]	[illegible]	[illegible]	[illegible]	[illegible]
GOLGA4	2803	symbol	H. sapiens	2803	H. sapiens	GOLGA4	golgin A4	GO:0045773 positive regulation o			Cytosol;Golgi apparatus(Supported)			(M5910)HA	[illegible]	[illegible]	[illegible]	[illegible]	[illegible]	[illegible]	[illegible]	[illegible]	[illegible]	[illegible]	[illegible]	[illegible]	[illegible]	[illegible]	[illegible]	[illegible]	[illegible]	[illegible]	[illegible]	[illegible]
ITGA9	3680	symbol	H. sapiens	3680	H. sapiens	ITGA9	integrin sul	GO:0035993 neutrophil chemotax			Cell Junctions;Nucleoplasm		(M118)PID		[illegible]	[illegible]	[illegible]	[illegible]	[illegible]	[illegible]	[illegible]	[illegible]	[illegible]	[illegible]	[illegible]	[illegible]	[illegible]	[illegible]	[illegible]	[illegible]	[illegible]	[illegible]	[illegible]	[illegible]
CTDSPL	10217	symbol	H. sapiens	10217	H. sapiens	CTDSPL	CTD small	GO:2000134 negative regulation of		Enzymes/E	Nucleoplasm(Supported)		(M228)PID SMAD2 3P		[illegible]	[illegible]	[illegible]	[illegible]	[illegible]	[illegible]	[illegible]	[illegible]	[illegible]	[illegible]	[illegible]	[illegible]	[illegible]	[illegible]	[illegible]	[illegible]	[illegible]	[illegible]	[illegible]	[illegible]
MIR26A1	407015	gene_synon	H. sapiens	407015	H. sapiens	MIR26A1	microRNA 2	GO:1903409 negative regulation of inward rectifier potassium channel activity							[illegible]	[illegible]	[illegible]	[illegible]	[illegible]	[illegible]	[illegible]	[illegible]	[illegible]	[illegible]	[illegible]	[illegible]	[illegible]	[illegible]	[illegible]	[illegible]	[illegible]	[illegible]	[illegible]	[illegible]
VILL	50853	symbol	H. sapiens	50853	H. sapiens	VILL	villin like	GO:0051693 actin filament capping;GO:0030835 negative regulation of actin fil							[illegible]	[illegible]	[illegible]	[illegible]	[illegible]	[illegible]	[illegible]	[illegible]	[illegible]	[illegible]	[illegible]	[illegible]	[illegible]	[illegible]	[illegible]	[illegible]	[illegible]	[illegible]	[illegible]	[illegible]
PLCD1	5333	symbol	H. sapiens	5333	H. sapiens	PLCD1	phospholip	GO:0051209 release o		Enzymes/E	Nucleoplasm(Supported)	D-Myo-Inositol-1,4,5-Triphosphate			[illegible]	[illegible]	[illegible]	[illegible]	[illegible]	[illegible]	[illegible]	[illegible]	[illegible]	[illegible]	[illegible]	[illegible]	[illegible]	[illegible]	[illegible]	[illegible]	[illegible]	[illegible]	[illegible]	[illegible]
DLEC1	9940	symbol	H. sapiens	9940	H. sapiens	DLEC1	DLEC1 cilia	GO:0008285 negative regulation of cell population proliferation							[illegible]	[illegible]	[illegible]	[illegible]	[illegible]	[illegible]	[illegible]	[illegible]	[illegible]	[illegible]	[illegible]	[illegible]	[illegible]	[illegible]	[illegible]	[illegible]	[illegible]	[illegible]	[illegible]	[illegible]
ACAA1	30	symbol	H. sapiens	30	H. sapiens	ACAA1	acetyl-CoA	GO:0010124 phenylac		Enzymes/E	Peroxisome	Trimetazidine (Palmito		(M5949)HA	[illegible]	[illegible]	[illegible]	[illegible]	[illegible]	[illegible]	[illegible]	[illegible]	[illegible]	[illegible]	[illegible]	[illegible]	[illegible]	[illegible]	[illegible]	[illegible]	[illegible]	[illegible]	[illegible]	[illegible]
MYD88	4615	symbol	H. sapiens	4615	H. sapiens	MYD88	innate imm	GO:0034162 toll-like receptor 9 s			Cytosol(Supported)		(M264)PID	(M5897)HA	[illegible]	[illegible]	[illegible]	[illegible]	[illegible]	[illegible]	[illegible]	[illegible]	[illegible]	[illegible]	[illegible]	[illegible]	[illegible]	[illegible]	[illegible]	[illegible]	[illegible]	[illegible]	[illegible]	[illegible]
OXSR1	9943	symbol	H. sapiens	9943	H. sapiens	OXSR1	oxidative st	GO:0018105	STE Ser/Th	Enzymes/E	Cytosol;Nucleoplasm(Approved)				[illegible]	[illegible]	[illegible]	[illegible]	[illegible]	[illegible]	[illegible]	[illegible]	[illegible]	[illegible]	[illegible]	[illegible]	[illegible]	[illegible]	[illegible]	[illegible]	[illegible]	[illegible]	[illegible]	[illegible]
BTRC	8945	symbol	H. sapiens	8945	H. sapiens	BTRC	beta-transd	GO:0000223 stimulatory C-type		Enzymes/E			(M5493)W	(M5956)HA	[illegible]	[illegible]	[illegible]	[illegible]	[illegible]	[illegible]	[illegible]	[illegible]	[illegible]	[illegible]	[illegible]	[illegible]	[illegible]	[illegible]	[illegible]	[illegible]	[illegible]	[illegible]	[illegible]	[illegible]
LBX1	10660	symbol	H. sapiens	10660	H. sapiens	LBX1	ladybird ho	GO:0021920 regulatio	Transcriptic		Nuclear bodies;Nucleoplasm(Approved)				[illegible]	[illegible]	[illegible]	[illegible]	[illegible]	[illegible]	[illegible]	[illegible]	[illegible]	[illegible]	[illegible]	[illegible]	[illegible]	[illegible]	[illegible]	[illegible]	[illegible]	[illegible]	[illegible]	[illegible]
TLX1	3195	symbol	H. sapiens	3195	H. sapiens	TLX1	T cell leuke	GO:0045944 positive r	Transcription factors/Helix-turn-helix domains						[illegible]	[illegible]	[illegible]	[illegible]	[illegible]	[illegible]	[illegible]	[illegible]	[illegible]	[illegible]	[illegible]	[illegible]	[illegible]	[illegible]	[illegible]	[illegible]	[illegible]	[illegible]	[illegible]	[illegible]
KAZALD1	81621	symbol	H. sapiens	81621	H. sapiens	KAZALD1	Kazal type	GO:0030198 extracellular matrix organization;GO:0001501					(M3468)NABA ECM RI		[illegible]	[illegible]	[illegible]	[illegible]	[illegible]	[illegible]	[illegible]	[illegible]	[illegible]	[illegible]	[illegible]	[illegible]	[illegible]	[illegible]	[illegible]	[illegible]	[illegible]	[illegible]	[illegible]	[illegible]
SFXN3	81855	symbol	H. sapiens	81855	H. sapiens	SFXN3	sideroflexin	GO:0006730 one-carbon metabo			Mitochondria		(M254)PID MYC REPRE		[illegible]	[illegible]	[illegible]	[illegible]	[illegible]	[illegible]	[illegible]	[illegible]	[illegible]	[illegible]	[illegible]	[illegible]	[illegible]	[illegible]	[illegible]	[illegible]	[illegible]	[illegible]	[illegible]	[illegible]
PDZD7	79955	symbol	H. sapiens	79955	H. sapiens	PDZD7	PDZ doma	GO:0060088 auditory receptor cell stereocilium organization;GO:0002093 audit							[illegible]	[illegible]	[illegible]	[illegible]	[illegible]	[illegible]	[illegible]	[illegible]	[illegible]	[illegible]	[illegible]	[illegible]	[illegible]	[illegible]	[illegible]	[illegible]	[illegible]	[illegible]	[illegible]	[illegible]
LZTS2	84445	symbol	H. sapiens	84445	H. sapiens	LZTS2	leucine zip	GO:0060682 primary ureteric bud			Cytosol;Plasma membrane(Approved)				[illegible]	[illegible]	[illegible]	[illegible]	[illegible]	[illegible]	[illegible]	[illegible]	[illegible]	[illegible]	[illegible]	[illegible]	[illegible]	[illegible]	[illegible]	[illegible]	[illegible]	[illegible]	[illegible]	[illegible]
C26H10orf2						None	None	None	None	None	None	None	None	None	nan	nan	nan	nan	nan	nan	nan	nan	nan	nan	nan	nan	nan	nan	nan	nan	nan	nan	nan	nan
MRPL43	84545	symbol	H. sapiens	84545	H. sapiens	MRPL43	mitochond	GO:0070126 mitochondrial	Ribosomal		Mitochondria;Nucleoplasm(Supported)				[illegible]	[illegible]	[illegible]	[illegible]	[illegible]	[illegible]	[illegible]	[illegible]	[illegible]	[illegible]	[illegible]	[illegible]	[illegible]	[illegible]	[illegible]	[illegible]	[illegible]	[illegible]	[illegible]	[illegible]
SEMA4G	57715	symbol	H. sapiens	57715	H. sapiens	SEMA4G	semaphorin	GO:0048843 negative regulation			Lipid droplets;Vesicles(Supported)		(M5880)NABA ECM AF		[illegible]	[illegible]	[illegible]	[illegible]	[illegible]	[illegible]	[illegible]	[illegible]	[illegible]	[illegible]	[illegible]	[illegible]	[illegible]	[illegible]	[illegible]	[illegible]	[illegible]	[illegible]	[illegible]	[illegible]
SLF2	55719	symbol	H. sapiens	55719	H. sapiens	SLF2	SMC5-SMC	GO:0034184 positive regulation o			Nucleoplasm(Supported);Vesicles(Approved)				[illegible]	[illegible]	[illegible]	[illegible]	[illegible]	[illegible]	[illegible]	[illegible]	[illegible]	[illegible]	[illegible]	[illegible]	[illegible]	[illegible]	[illegible]	[illegible]	[illegible]	[illegible]	[illegible]	[illegible]
PAX2	5076	symbol	H. sapiens	5076	H. sapiens	PAX2	paired box	GO:2000594 positive r	Transcriptic		Nucleoplasm(Supported)				[illegible]	[illegible]	[illegible]	[illegible]	[illegible]	[illegible]	[illegible]	[illegible]	[illegible]	[illegible]	[illegible]	[illegible]	[illegible]	[illegible]	[illegible]	[illegible]	[illegible]	[illegible]	[illegible]	[illegible]
HIF1AN	55662	symbol	H. sapiens	55662	H. sapiens	HIF1AN	hypoxia inc	GO:0061428 negative r		Enzymes/E	Cytosol;Nu	D-tartaric	(M180)PID HIF1A PATHW		[illegible]	[illegible]	[illegible]	[illegible]	[illegible]	[illegible]	[illegible]	[illegible]	[illegible]	[illegible]	[illegible]	[illegible]	[illegible]	[illegible]	[illegible]	[illegible]	[illegible]	[illegible]	[illegible]	[illegible]
NDUFB8	4714	symbol	H. sapiens	4714	H. sapiens	NDUFB8	NADH:ubi	GO:0006120 mitochondrial electr				NADH (Nadh dehydro		(M5936)HA	[illegible]	[illegible]	[illegible]	[illegible]	[illegible]	[illegible]	[illegible]	[illegible]	[illegible]	[illegible]	[illegible]	[illegible]	[illegible]	[illegible]	[illegible]	[illegible]	[illegible]	[illegible]	[illegible]	[illegible]
SEC31B	25956	symbol	H. sapiens	25956	H. sapiens	SEC31B	SEC31 hom	GO:0090110 cargo loading into C			Cytosol;Nucleus;Vesicles(Approved)				[illegible]	[illegible]	[illegible]	[illegible]	[illegible]	[illegible]	[illegible]	[illegible]	[illegible]	[illegible]	[illegible]	[illegible]	[illegible]	[illegible]	[illegible]	[illegible]	[illegible]	[illegible]	[illegible]	[illegible]
WNT8B	7479	symbol	H. sapiens	7479	H. sapiens	WNT8B	Wnt family	GO:0048263 determination of dorsal identity;GO:0048					(M5883)NABA SECRET		[illegible]	[illegible]	[illegible]	[illegible]	[illegible]	[illegible]	[illegible]	[illegible]	[illegible]	[illegible]	[illegible]	[illegible]	[illegible]	[illegible]	[illegible]	[illegible]	[illegible]	[illegible]	[illegible]	[illegible]
SCD	6319	symbol	H. sapiens	6319	H. sapiens	SCD	stearoyl-Co	GO:0070542 response		Enzymes/E	Endoplasmic reticulum(Enhanced)			(M5892)HA	[illegible]	[illegible]	[illegible]	[illegible]	[illegible]	[illegible]	[illegible]	[illegible]	[illegible]	[illegible]	[illegible]	[illegible]	[illegible]	[illegible]	[illegible]	[illegible]	[illegible]	[illegible]	[illegible]	[illegible]
PKD2L1	9033	symbol	H. sapiens	9033	H. sapiens	PKD2L1	polycystin	GO:0001581 detection	Transporters/Transporter channels and pores;Voltage-ga						[illegible]	[illegible]	[illegible]	[illegible]	[illegible]	[illegible]	[illegible]	[illegible]	[illegible]	[illegible]	[illegible]	[illegible]	[illegible]	[illegible]	[illegible]	[illegible]	[illegible]	[illegible]	[illegible]	[illegible]
CWF19L1	55280	symbol	H. sapiens	55280	H. sapiens	CWF19L1	CWF19 like	GO:0000398 mRNA splicing, via s			Golgi apparatus;Nucleoplasm(Approved)				[illegible]	[illegible]	[illegible]	[illegible]	[illegible]	[illegible]	[illegible]	[illegible]	[illegible]	[illegible]	[illegible]	[illegible]	[illegible]	[illegible]	[illegible]	[illegible]	[illegible]	[illegible]	[illegible]	[illegible]
CHUK	1147	symbol	H. sapiens	1147	H. sapiens	CHUK	componen	GO:00356	Other	Enzymes/E	Cytosol;Nu	Aminosalic	(M37)PID	(M5905)HA	[illegible]	[illegible]	[illegible]	[illegible]	[illegible]	[illegible]	[illegible]	[illegible]	[illegible]	[illegible]	[illegible]	[illegible]	[illegible]	[illegible]	[illegible]	[illegible]	[illegible]	[illegible]	[illegible]	[illegible]
ERLIN1	10613	symbol	H. sapiens	10613	H. sapiens	ERLIN1	ER lipid raf	GO:0045541 negative regulation			Endoplasmic reticulum(Approved)				[illegible]	[illegible]	[illegible]	[illegible]	[illegible]	[illegible]	[illegible]	[illegible]	[illegible]	[illegible]	[illegible]	[illegible]	[illegible]	[illegible]	[illegible]	[illegible]	[illegible]	[illegible]	[illegible]	[illegible]
CPN1	1369	symbol	H. sapiens	1369	H. sapiens	CPN1	carboxype	GO:0010815 bradykinin		Enzymes/E				(M5946)HA	[illegible]	[illegible]	[illegible]	[illegible]	[illegible]	[illegible]	[illegible]	[illegible]	[illegible]	[illegible]	[illegible]	[illegible]	[illegible]	[illegible]	[illegible]	[illegible]	[illegible]	[illegible]	[illegible]	[illegible]
DNMBP	23268	symbol	H. sapiens	23268	H. sapiens	DNMBP	dynamin b	GO:0008360 regulation of cell sh			Golgi apparatus;Nucleoplasm(Supported)				[illegible]	[illegible]	[illegible]	[illegible]	[illegible]	[illegible]	[illegible]	[illegible]	[illegible]	[illegible]	[illegible]	[illegible]	[illegible]	[illegible]	[illegible]	[illegible]	[illegible]	[illegible]	[illegible]	[illegible]
KIF4A	24137	symbol	H. sapiens	24137	H. sapiens	KIF4A	kinesin fam	GO:0051256 mitotic spindle midz			Cytokinetic bridge;Nucleoplasm(E			(M5893)HA	[illegible]	[illegible]	[illegible]	[illegible]	[illegible]	[illegible]	[illegible]	[illegible]	[illegible]	[illegible]	[illegible]	[illegible]	[illegible]	[illegible]	[illegible]	[illegible]	[illegible]	[illegible]	[illegible]	[illegible]
PDZD11	51248	symbol	H. sapiens	51248	H. sapiens	PDZD11	PDZ domai	GO:0045199 maintenance of epithelial cell apical/basal polarity;GO:0006768 bio							[illegible]	[illegible]	[illegible]	[illegible]	[illegible]	[illegible]	[illegible]	[illegible]	[illegible]	[illegible]	[illegible]	[illegible]	[illegible]	[illegible]	[illegible]	[illegible]	[illegible]	[illegible]	[illegible]	[illegible]
ARR3	407	symbol	H. sapiens	407	H. sapiens	ARR3	arrestin 3	GO:0002031 G protein-coupled receptor internalization;					(M117)PID CONE PATH		[illegible]	[illegible]	[illegible]	[illegible]	[illegible]	[illegible]	[illegible]	[illegible]	[illegible]	[illegible]	[illegible]	[illegible]	[illegible]	[illegible]	[illegible]	[illegible]	[illegible]	[illegible]	[illegible]	[illegible]
P2RY4	5030	symbol	H. sapiens	5030	H. sapiens	P2RY4	pyrimidine	GO:0099509 regulation	G-protein coupled receptors			Adenosine		(M5956)HA	[illegible]	[illegible]	[illegible]	[illegible]	[illegible]	[illegible]	[illegible]	[illegible]	[illegible]	[illegible]	[illegible]	[illegible]	[illegible]	[illegible]	[illegible]	[illegible]	[illegible]	[illegible]	[illegible]	[illegible]
AWAT1	158833	symbol	H. sapiens	158833	H. sapiens	AWAT1	acyl-CoA w	GO:0010025 wax biosy		Enzymes/ENZYME proteins/Transferases					[illegible]	[illegible]	[illegible]	[illegible]	[illegible]	[illegible]	[illegible]	[illegible]	[illegible]	[illegible]	[illegible]	[illegible]	[illegible]	[illegible]	[illegible]	[illegible]	[illegible]	[illegible]	[illegible]	[illegible]
DGAT2L6	347516	symbol	H. sapiens	347516	H. sapiens	DGAT2L6	diacylglyce	GO:0036155 acylglycerol acyl-chain remodeling;GO:0006639 acylglycerol meta							[illegible]	[illegible]	[illegible]	[illegible]	[illegible]	[illegible]	[illegible]	[illegible]	[illegible]	[illegible]	[illegible]	[illegible]	[illegible]	[illegible]	[illegible]	[illegible]	[illegible]	[illegible]	[illegible]	[illegible]
IGBP1	3476	symbol	H. sapiens	3476	H. sapiens	IGBP1	immunoglob	GO:0043154 negative regulation c							[illegible]	[illegible]	[illegible]	[illegible]	[illegible]	[illegible]	[illegible]	[illegible]	[illegible]	[illegible]	[illegible]	[illegible]	[illegible]	[illegible]	[illegible]	[illegible]	[illegible]	[illegible]	[illegible]	[illegible]
OTUD6A	139562	symbol	H. sapiens	139562	H. sapiens	OTUD6A	OTU deubi	GO:1990167 protein K		Enzymes/ENZYME proteins/Hydrolases					[illegible]	[illegible]	[illegible]	[illegible]	[illegible]	[illegible]	[illegible]	[illegible]	[illegible]	[illegible]	[illegible]	[illegible]	[illegible]	[illegible]	[illegible]	[illegible]	[illegible]	[illegible]	[illegible]	[illegible]
AWAT2	158835	symbol	H. sapiens	158835	H. sapiens	AWAT2	acyl-CoA w	GO:0036155 acylglycerol		Enzymes/ENZYME proteins/Transferases					[illegible]	[illegible]	[illegible]	[illegible]	[illegible]	[illegible]	[illegible]	[illegible]	[illegible]	[illegible]	[illegible]	[illegible]	[illegible]	[illegible]	[illegible]	[illegible]	[illegible]	[illegible]	[illegible]	[illegible]
EDA	1896	symbol	H. sapiens	1896	H. sapiens	EDA	ectodyspla	GO:0061153 trachea gland develo			Vesicles(Supported);Lip		(M5880)NABA SECRETE		[illegible]	[illegible]	[illegible]	[illegible]	[illegible]	[illegible]	[illegible]	[illegible]	[illegible]	[illegible]	[illegible]	[illegible]	[illegible]	[illegible]	[illegible]	[illegible]	[illegible]	[illegible]	[illegible]	[illegible]

CDX4	1046	symbol	H. sapiens	1046	H. sapiens	CDX4	caudal type	GO:0060711	labyrinthi	Transcription factors/Helix-turn-h			(M223)PID BETA CATE		0.0	1.0	0.0	0.0	0.0	0.0	0.0	0.0	0.0	0.0	0.0	0.0	0.0	0.0	0.0	0.0	0.0	1.0	0.0	0.0
FAM155B	27112	symbol	H. sapiens	27112	H. sapiens	FAM155B	family with	GO:0098703	calcium ion import across plasma membrane;GO:1902656 calcium						0.0	0.0	0.0	0.0	0.0	0.0	0.0	0.0	0.0	0.0	0.0	0.0	0.0	0.0	0.0	0.0	0.0	0.0	0.0	0.0
PJA1	64219	symbol	H. sapiens	64219	H. sapiens	PJA1	praja ring f	GO:0016567	protein u	Enzymes/E	Nucleoli;Nucleus(Approved)				1.0	0.0	0.0	0.0	0.0	0.0	0.0	0.0	0.0	0.0	0.0	0.0	0.0	0.0	0.0	0.0	0.0	0.0	0.0	0.0
AR	367	symbol	H. sapiens	367	H. sapiens	AR	androgen r	GO:0060520	activatio	Nuclear rec	Mitochond	Levonorges	(M213)PID	(M5948)HA	1.0	1.0	0.0	1.0	0.0	0.0	0.0	0.0	1.0	1.0	0.0	0.0	1.0	0.0	0.0	0.0	0.0	1.0	0.0	0.0
GRIA3	2892	symbol	H. sapiens	2892	H. sapiens	GRIA3	glutamate	GO:2000310	regulatio	Transporters/Transport		L-Glutamic Acid (Extracellular-glu			0.0	0.0	0.0	0.0	0.0	0.0	0.0	0.0	0.0	0.0	0.0	0.0	0.0	0.0	0.0	0.0	1.0	0.0	0.0	0.0
TRPC5	7224	symbol	H. sapiens	7224	H. sapiens	TRPC5	transient re	GO:0050774	negative	Transporters/Transporter channels and pores;Voltage-ga					0.0	0.0	0.0	0.0	0.0	0.0	0.0	0.0	1.0	0.0	0.0	0.0	0.0	0.0	0.0	0.0	0.0	0.0	0.0	0.0
TRPC5OS	10032913[illegible]	symbol	H. sapiens	10032913[illegible]	H. sapiens	TRPC5OS	TRPC5 opposite strand								0.0	0.0	0.0	0.0	0.0	0.0	0.0	0.0	0.0	0.0	0.0	0.0	0.0	0.0	0.0	0.0	0.0	0.0	0.0	0.0
DCX	1641	symbol	H. sapiens	1641	H. sapiens	DCX	doublecort	GO:0042461	photoreceptor cell d		Cytosol(Approved)		(M163)PID	(M5957)HA	0.0	0.0	0.0	0.0	0.0	0.0	0.0	0.0	0.0	0.0	0.0	0.0	0.0	0.0	0.0	0.0	0.0	0.0	0.0	0.0
CAPN6	827	symbol	H. sapiens	827	H. sapiens	CAPN6	calpain 6	GO:0001578	microtub	Enzymes/P	Cytosol(Uncertain)				0.0	0.0	0.0	0.0	0.0	0.0	0.0	0.0	0.0	0.0	0.0	0.0	0.0	0.0	0.0	0.0	0.0	0.0	0.0	0.0
PAK3	5063	symbol	H. sapiens	5063	H. sapiens	PAK3	p21 (RAC1	GO:00610[illegible]	STE Ser/Thr	Enzymes/{E	Cytosol(Approved)		(M76)PID P	(M5957)HA	1.0	0.0	0.0	0.0	0.0	0.0	0.0	0.0	1.0	0.0	0.0	1.0	0.0	0.0	0.0	0.0	0.0	0.0	0.0	0.0
RGAG1	57529	gene_synor	H. sapiens	57529	H. sapiens	RTL9	retrotransposon Gag like 9								0.0	0.0	0.0	0.0	0.0	0.0	0.0	0.0	0.0	0.0	0.0	0.0	0.0	0.0	0.0	0.0	0.0	0.0	0.0	0.0
ACSL4	2182	symbol	H. sapiens	2182	H. sapiens	ACSL4	acyl-CoA s	GO:0035338	long-cha	Enzymes/E	Golgi appa	Icosapent (Very long-c		(M5949)HA	0.0	0.0	0.0	1.0	1.0	0.0	0.0	0.0	0.0	0.0	0.0	0.0	0.0	1.0	0.0	0.0	0.0	0.0	1.0	0.0
KCNE5	23630	symbol	H. sapiens	23630	H. sapiens	KCNE5	potassium	GO:1903765	negative regulation c		Cytosol(Uncertain)				0.0	0.0	0.0	0.0	0.0	0.0	0.0	0.0	0.0	0.0	0.0	0.0	0.0	0.0	0.0	1.0	0.0	0.0	0.0	0.0
GPR119	139760	symbol	H. sapiens	139760	H. sapiens	GPR119	G protein-c	GO:0030073	insulin se	G-protein coupled rec		APD668 (Phosphatidylcholine bin			0.0	0.0	0.0	0.0	0.0	0.0	0.0	0.0	0.0	0.0	0.0	0.0	0.0	0.0	0.0	0.0	0.0	0.0	0.0	0.0

Supplementary Table s7：Enrichment of candidate genes in UC goats and other cashmere goats to perform selective evolution analysis

GroupID	Category	Term	Description	LogP	Log(q-va	InTerm_I	Genes	Symbols
1_Summar	GO Biolog	GO:00072	I-kappaB kinase/NF-kappaB signaling	-6.84047	-2.725	15/279	929,1147,1	CD14,CHUK,EDA,MYD88,REL,UBE2N,BTRC,NEK6,CXXC5,TNFRSF19,CACTIN,CARD9,ALPK1,MTDH,TIFA,PAK3,PSMB7,UBE2D2,DDX60,VAMP8,CREB3L3,MIR126,VCAN,SEC16A,EEA1,RPL30,AR,ZBTB7A,TLE5,IGBP1,MAP2K2,STK3,OXSR1,UBAC1,FZR1,PJA1,PIAS4,MIR26A1,POLR1A,RC3H2,ADAMTS13,CPN1,NOTCH1,TBXA2R
1_Member	GO Biolog	GO:00072	I-kappaB kinase/NF-kappaB signaling	-6.84047	-2.725	15/279	929,1147,1	CD14,CHUK,EDA,MYD88,REL,UBE2N,BTRC,NEK6,CXXC5,TNFRSF19,CACTIN,CARD9,ALPK1,MTDH,TIFA
1_Member	GO Biolog	GO:00431	regulation of I-kappaB kinase/NF-kappaB signaling	-6.74309	-2.725	14/245	1147,1896.	CHUK,EDA,MYD88,REL,UBE2N,BTRC,NEK6,CXXC5,TNFRSF19,CACTIN,CARD9,ALPK1,MTDH,TIFA
1_Member	GO Biolog	GO:00431	positive regulation of I-kappaB kinase/NF-kappaB sig	-6.44435	-2.602	12/185	1147,1896.	CHUK,EDA,MYD88,REL,UBE2N,NEK6,CXXC5,TNFRSF19,CARD9,ALPK1,MTDH,TIFA
1_Member	GO Biolog	GO:00027	innate immune response-activating signal transducti	-4.96185	-1.245	13/301	929,1147,4	CD14,CHUK,MYD88,PAK3,PSMB7,UBE2D2,UBE2N,BTRC,DDX60,CACTIN,CARD9,ALPK1,TIFA
1_Member	GO Biolog	GO:00022	activation of innate immune response	-4.62509	-1.005	13/324	929,1147,4	CD14,CHUK,MYD88,PAK3,PSMB7,UBE2D2,UBE2N,BTRC,DDX60,CACTIN,CARD9,ALPK1,TIFA
1_Member	GO Biolog	GO:00313	positive regulation of defense response	-4.18227	-0.718	16/517	929,1147,4	CD14,CHUK,MYD88,PAK3,PSMB7,UBE2D2,UBE2N,VAMP8,BTRC,DDX60,CACTIN,CARD9,ALPK1,CREB3L3,TIFA,MIR126
1_Member	Canonical	M264	PID TOLL ENDOGENOUS PATHWAY	-3.99191	-0.718	4/25	929,1147,1	CD14,CHUK,VCAN,MYD88
1_Member	Reactome	R-HSA-93	IKK complex recruitment mediated by RIP1	-3.99191	-0.718	4/25	929,1147,7	CD14,CHUK,UBE2D2,UBE2N
1_Member	CORUM	CORUM:51	TNF-alpha/NF-kappa B signaling complex (SEC16A, C	-3.95755	-0.718	3/10	1147,5966.	CHUK,REL,SEC16A
1_Member	GO Biolog	GO:00450	positive regulation of innate immune response	-3.89867	-0.694	13/382	929,1147,4	CD14,CHUK,MYD88,PAK3,PSMB7,UBE2D2,UBE2N,BTRC,DDX60,CACTIN,CARD9,ALPK1,TIFA
1_Member	GO Biolog	GO:00022	pattern recognition receptor signaling pathway	-3.73458	-0.683	9/200	929,1147,4	CD14,CHUK,MYD88,UBE2D2,UBE2N,DDX60,CACTIN,ALPK1,TIFA
1_Member	CORUM	CORUM:51	TNF-alpha/NF-kappa B signaling complex (CHUK, BT	-3.7007	-0.683	3/12	1147,5966.	CHUK,REL,BTRC
1_Member	Reactome	R-HSA-16	Toll Like Receptor 9 (TLR9) Cascade	-3.37728	-0.427	6/97	929,1147,4	CD14,CHUK,MYD88,UBE2N,EEA1,BTRC
1_Member	Reactome	R-HSA-56	CLEC7A (Dectin-1) signaling	-3.2835	-0.411	6/101	1147,5695.	CHUK,PSMB7,UBE2D2,UBE2N,BTRC,CARD9
1_Member	Reactome	R-HSA-56	C-type lectin receptors (CLRs)	-3.23538	-0.411	7/143	1147,5063.	CHUK,PAK3,PSMB7,UBE2D2,UBE2N,BTRC,CARD9
1_Member	CORUM	CORUM:52	TNF-alpha/Nf-kappa B signaling complex (RPL6, RPL	-3.22656	-0.411	3/17	1147,5966.	CHUK,REL,RPL30
1_Member	GO Biolog	GO:00450	regulation of innate immune response	-3.20801	-0.411	13/451	929,1147,4	CD14,CHUK,MYD88,PAK3,PSMB7,UBE2D2,UBE2N,BTRC,DDX60,CACTIN,CARD9,ALPK1,TIFA
1_Member	GO Biolog	GO:00510	positive regulation of NF-kappaB transcription factor	-3.06182	-0.311	7/153	367,1147,1	AR,CHUK,EDA,MYD88,UBE2N,ZBTB7A,MTDH
1_Member	Reactome	R-HSA-16	TICAM1, RIP1-mediated IKK complex recruitment	-3.01172	-0.272	3/20	1147,7322.	CHUK,UBE2D2,UBE2N
1_Member	Reactome	R-HSA-16	Toll-like Receptor Cascades	-2.98021	-0.252	7/158	929,1147,4	CD14,CHUK,MYD88,UBE2D2,UBE2N,EEA1,BTRC
1_Member	GO Biolog	GO:00705	response to interleukin-1	-2.93307	-0.227	8/208	166,1147,3	TLE5,CHUK,IGBP1,MYD88,PSMB7,UBE2N,BTRC,CACTIN
1_Member	Reactome	R-HSA-20	TCR signaling	-2.89147	-0.220	6/120	1147,5063.	CHUK,PAK3,PSMB7,UBE2D2,UBE2N,BTRC
1_Member	GO Biolog	GO:00310	stress-activated protein kinase signaling cascade	-2.87354	-0.220	10/317	1147,3476.	CHUK,IGBP1,PAK3,MAP2K2,STK3,UBE2N,BTRC,OXSR1,TNFRSF19,CARD9
1_Member	Reactome	R-HSA-98	Class I MHC mediated antigen processing & presenta	-2.87323	-0.220	11/373	929,1147,4	CD14,CHUK,MYD88,PSMB7,UBE2D2,UBE2N,VAMP8,BTRC,UBAC1,FZR1,PJA1
1_Member	Reactome	R-HSA-28	FCERI mediated NF-kB activation	-2.86692	-0.220	5/82	1147,5695.	CHUK,PSMB7,UBE2D2,UBE2N,BTRC
1_Member	GO Biolog	GO:00510	regulation of DNA-binding transcription factor activit	-2.79832	-0.182	12/440	367,1147,1	AR,CHUK,EDA,MYD88,STK3,UBE2N,BTRC,ZBTB7A,PIAS4,CACTIN,MTDH,MIR26A1
1_Member	Reactome	R-HSA-12	ER-Phagosome pathway	-2.79738	-0.182	5/85	929,1147,4	CD14,CHUK,MYD88,PSMB7,VAMP8
1_Member	CORUM	CORUM:52	TNF-alpha/NF-kappa B signaling complex 5	-2.72279	-0.175	3/25	1147,5966.	CHUK,REL,POLR1A
1_Member	Reactome	R-HSA-16	Toll Like Receptor 4 (TLR4) Cascade	-2.69731	-0.171	6/131	929,1147,4	CD14,CHUK,MYD88,UBE2D2,UBE2N,BTRC
1_Member	Reactome	R-HSA-97	TRAF6 mediated induction of NFkB and MAP kinases	-2.64581	-0.151	5/92	929,1147,4	CD14,CHUK,MYD88,UBE2N,BTRC
1_Member	Reactome	R-HSA-16	Toll Like Receptor 7/8 (TLR7/8) Cascade	-2.62528	-0.151	5/93	929,1147,4	CD14,CHUK,MYD88,UBE2N,BTRC
1_Member	Reactome	R-HSA-97	MyD88 dependent cascade initiated on endosome	-2.62528	-0.151	5/93	929,1147,4	CD14,CHUK,MYD88,UBE2N,BTRC
1_Member	Reactome	R-HSA-52	Diseases of Immune System	-2.62474	-0.151	3/27	929,1147,4	CD14,CHUK,MYD88
1_Member	Reactome	R-HSA-56	Diseases associated with the TLR signaling cascade	-2.62474	-0.151	3/27	929,1147,4	CD14,CHUK,MYD88
1_Member	GO Biolog	GO:00380	NIK/NF-kappaB signaling	-2.56339	-0.133	7/187	929,1147,1	CD14,CHUK,EDA,PSMB7,REL,BTRC,RC3H2
1_Member	Reactome	R-HSA-16	MyD88:MAL(TIRAP) cascade initiated on plasma mem	-2.54571	-0.133	5/97	929,1147,4	CD14,CHUK,MYD88,UBE2N,BTRC
1_Member	Reactome	R-HSA-16	Toll Like Receptor TLR6:TLR2 Cascade	-2.54571	-0.133	5/97	929,1147,4	CD14,CHUK,MYD88,UBE2N,BTRC
1_Member	GO Biolog	GO:00356	TRIF-dependent toll-like receptor signaling pathway	-2.53443	-0.133	3/29	929,1147,7	CD14,CHUK,UBE2D2
1_Member	Reactome	R-HSA-20	Downstream TCR signaling	-2.52643	-0.133	5/98	1147,5695.	CHUK,PSMB7,UBE2D2,UBE2N,BTRC
1_Member	Reactome	R-HSA-16	MyD88-independent TLR4 cascade	-2.50738	-0.133	5/99	929,1147,7	CD14,CHUK,UBE2D2,UBE2N,BTRC
1_Member	Reactome	R-HSA-93	TRIF(TICAM1)-mediated TLR4 signaling	-2.50738	-0.133	5/99	929,1147,7	CD14,CHUK,UBE2D2,UBE2N,BTRC
1_Member	GO Biolog	GO:00704	interleukin-1-mediated signaling pathway	-2.48857	-0.133	5/100	1147,4615.	CHUK,MYD88,PSMB7,UBE2N,BTRC
1_Member	Reactome	R-HSA-16	Toll Like Receptor TLR1:TLR2 Cascade	-2.48857	-0.133	5/100	929,1147,4	CD14,CHUK,MYD88,UBE2N,BTRC
1_Member	Reactome	R-HSA-18	Toll Like Receptor 2 (TLR2) Cascade	-2.48857	-0.133	5/100	929,1147,4	CD14,CHUK,MYD88,UBE2N,BTRC
1_Member	Reactome	R-HSA-12	Antigen processing-Cross presentation	-2.46998	-0.119	5/101	929,1147,4	CD14,CHUK,MYD88,PSMB7,VAMP8
1_Member	Reactome	R-HSA-90	Interleukin-1 signaling	-2.43346	-0.106	5/103	1147,4615.	CHUK,MYD88,PSMB7,UBE2N,BTRC
1_Member	GO Biolog	GO:00027	MyD88-independent toll-like receptor signaling path	-2.37299	-0.083	3/33	929,1147,7	CD14,CHUK,UBE2D2
1_Member	GO Biolog	GO:00346	response to tumor necrosis factor	-2.35424	-0.077	9/315	929,1147,1	CD14,CHUK,EDA,IGBP1,PSMB7,ADAMTS13,PIAS4,TNFRSF19,CACTIN
1_Member	Reactome	R-HSA-11	Activation of NF-kappaB in B cells	-2.35078	-0.077	4/67	1147,5695.	CHUK,PSMB7,REL,BTRC
1_Member	Canonical	M110	PID IL1 PATHWAY	-2.33603	-0.077	3/34	1147,4615.	CHUK,MYD88,UBE2N
1_Member	GO Biolog	GO:00510	positive regulation of DNA-binding transcription fact	-2.27693	-0.050	8/266	367,1147,1	AR,CHUK,EDA,MYD88,STK3,UBE2N,ZBTB7A,MTDH
1_Member	GO Biolog	GO:00022	stimulatory C-type lectin receptor signaling pathway	-2.26296	-0.050	5/113	1147,5063.	CHUK,PAK3,PSMB7,BTRC,CARD9
1_Member	GO Biolog	GO:00027	immune response-activating signal transduction	-2.25895	-0.050	14/647	929,1147,4	CD14,CHUK,MYD88,PAK3,PSMB7,UBE2D2,UBE2N,BTRC,RC3H2,DDX60,CACTIN,CARD9,ALPK1,TIFA
1_Member	Reactome	R-HSA-16	NOD1/2 Signaling Pathway	-2.23208	-0.039	3/37	1147,7334.	CHUK,UBE2N,CARD9
1_Member	GO Biolog	GO:00022	innate immune response activating cell surface recep	-2.21539	-0.030	5/116	1147,5063.	CHUK,PAK3,PSMB7,BTRC,CARD9
1_Member	GO Biolog	GO:00022	activation of immune response	-2.17796	0.000	15/731	929,1147,1	CD14,CHUK,CPN1,MYD88,PAK3,PSMB7,UBE2D2,UBE2N,BTRC,RC3H2,DDX60,CACTIN,CARD9,ALPK1,TIFA
1_Member	GO Biolog	GO:00514	stress-activated MAPK cascade	-2.0857	0.000	8/287	1147,3476.	CHUK,IGBP1,MAP2K2,STK3,UBE2N,BTRC,TNFRSF19,CARD9
1_Member	GO Biolog	GO:00027	immune response-regulating signaling pathway	-2.07229	0.000	14/681	929,1147,4	CD14,CHUK,MYD88,PAK3,PSMB7,UBE2D2,UBE2N,BTRC,RC3H2,DDX60,CACTIN,CARD9,ALPK1,TIFA
1_Member	GO Biolog	GO:00324	regulation of type I interferon production	-2.05321	0.000	5/127	929,1147,4	CD14,CHUK,MYD88,REL,CACTIN
1_Member	GO Biolog	GO:00713	cellular response to interleukin-1	-2.02855	0.000	6/180	1147,4615.	CHUK,MYD88,PSMB7,UBE2N,BTRC,CACTIN
1_Member	GO Biolog	GO:00326	type I interferon production	-2.02561	0.000	5/129	929,1147,4	CD14,CHUK,MYD88,REL,CACTIN
1_Member	Reactome	R-HSA-11	Downstream signaling events of B Cell Receptor (BCR	-2.02432	0.000	4/83	1147,5695.	CHUK,PSMB7,REL,BTRC
1_Member	GO Biolog	GO:00022	response to molecule of bacterial origin	-2.00548	0.000	9/358	929,1147,4	CD14,CHUK,MYD88,NOTCH1,TBXA2R,ADAMTS13,CACTIN,CARD9,MTDH
2_Summar	GO Biolog	GO:00600	canonical Wnt signaling pathway	-4.40852	-0.868	13/340	166,1613,1	TLE5,DAPK3,EDA,NOTCH1,PSMB7,STK3,TLE2,WNT8B,BTRC,TLE6,TCF7L1,LZTS2,AMER2,AR,CDX4,VCAN,GRID2,SH3GL1,PIAS4
2_Member	GO Biolog	GO:00600	canonical Wnt signaling pathway	-4.40852	-0.868	13/340	166,1613,1	TLE5,DAPK3,EDA,NOTCH1,PSMB7,STK3,TLE2,WNT8B,BTRC,TLE6,TCF7L1,LZTS2,AMER2
2_Member	Canonical	M223	PID BETA CATENIN NUC PATHWAY	-3.83348	-0.683	6/80	166,367,1C	TLE5,AR,CDX4,VCAN,TLE2,TCF7L1
2_Member	GO Biolog	GO:00608	regulation of canonical Wnt signaling pathway	-3.73196	-0.683	11/294	166,1613,1	TLE5,DAPK3,EDA,NOTCH1,PSMB7,STK3,TLE2,BTRC,TLE6,LZTS2,AMER2
2_Member	Reactome	R-HSA-46	Repression of WNT target genes	-3.7007	-0.683	3/12	166,7089,8	TLE5,TLE2,TCF7L1
2_Member	GO Biolog	GO:00301	regulation of Wnt signaling pathway	-3.38835	-0.427	12/376	166,1613,1	TLE5,DAPK3,EDA,NOTCH1,PSMB7,STK3,TLE2,BTRC,TLE6,TCF7L1,LZTS2,AMER2
2_Member	GO Biolog	GO:00900	negative regulation of canonical Wnt signaling pathw	-3.26343	-0.411	8/185	166,4851,5	TLE5,NOTCH1,PSMB7,STK3,TLE2,TLE6,LZTS2,AMER2
2_Member	GO Biolog	GO:19051	cell surface receptor signaling pathway involved in ce	-3.21271	-0.411	16/631	166,1613,1	TLE5,DAPK3,EDA,GRID2,NOTCH1,PSMB7,SH3GL1,STK3,TLE2,WNT8B,BTRC,PIAS4,TLE6,TCF7L1,LZTS2,AMER2
2_Member	GO Biolog	GO:00160	Wnt signaling pathway	-3.08353	-0.311	14/525	166,1613,1	TLE5,DAPK3,EDA,NOTCH1,PSMB7,STK3,TLE2,WNT8B,BTRC,PIAS4,TLE6,TCF7L1,LZTS2,AMER2
2_Member	GO Biolog	GO:01987	cell-cell signaling by wnt	-3.06768	-0.311	14/527	166,1613,1	TLE5,DAPK3,EDA,NOTCH1,PSMB7,STK3,TLE2,WNT8B,BTRC,PIAS4,TLE6,TCF7L1,LZTS2,AMER2
2_Member	Reactome	R-HSA-19	Degradation of beta-catenin by the destruction comp	-2.84341	-0.206	5/83	166,5695,7	TLE5,PSMB7,TLE2,BTRC,TCF7L1
2_Member	GO Biolog	GO:00301	negative regulation of Wnt signaling pathway	-2.7788	-0.182	8/220	166,4851,5	TLE5,NOTCH1,PSMB7,STK3,TLE2,TLE6,LZTS2,AMER2
2_Member	Reactome	R-HSA-37	Deactivation of the beta-catenin transactivating com	-2.07831	0.000	3/42	7089,8945.	TLE2,BTRC,TCF7L1
3_Summar	GO Biolog	GO:00350	maintenance of apical/basal cell polarity	-3.95755	-0.718	3/10	9355,5124	LHX2,PDZD11,CRB2
3_Member	GO Biolog	GO:00350	maintenance of apical/basal cell polarity	-3.95755	-0.718	3/10	9355,5124	LHX2,PDZD11,CRB2
3_Member	GO Biolog	GO:00451	maintenance of epithelial cell apical/basal polarity	-3.95755	-0.718	3/10	9355,5124	LHX2,PDZD11,CRB2
3_Member	GO Biolog	GO:00300	maintenance of cell polarity	-3.22656	-0.411	3/17	9355,5124	LHX2,PDZD11,CRB2
4_Summar	Reactome	R-HSA-40	Free fatty acids regulate insulin secretion	-3.82244	-0.683	3/11	2182,2767.	ACSL4,GNA11,GNA15,S1PR4,TBXA2R,ARR3,AR,HBEGF
4_Member	Reactome	R-HSA-40	Free fatty acids regulate insulin secretion	-3.82244	-0.683	3/11	2182,2767.	ACSL4,GNA11,GNA15
4_Member	Canonical	M155	PID S1P META PATHWAY	-2.94795	-0.231	3/21	2767,2769.	GNA11,GNA15,S1PR4
4_Member	Reactome	R-HSA-42	Thromboxane signalling through TP receptor	-2.77513	-0.182	3/24	2767,2769.	GNA11,GNA15,TBXA2R
4_Member	Canonical	M99	PID TXA2PATHWAY	-2.60521	-0.139	4/57	407,2767,2	ARR3,GNA11,GNA15,TBXA2R
4_Member	Reactome	R-HSA-39	Signal amplification	-2.37299	-0.083	3/33	2767,2769.	GNA11,GNA15,TBXA2R
4_Member	Canonical	M19043	ST ADRENERGIC	-2.26564	-0.050	3/36	367,2767,2	AR,GNA11,GNA15
4_Member	Canonical	M41	PID ER NONGENOMIC PATHWAY	-2.10735	0.000	3/41	1839,2767.	HBEGF,GNA11,GNA15
5_Summar	GO Biolog	GO:00302	lipid modification	-3.74284	-0.683	10/245	28,30,3295	ABO,ACAA1,HSD17B4,PEX13,SOCS2,MTMR6,PIP5K1C,INPP5E,TMEM150A,GLT6D1,ACSL4,SCD,MED22,AGPAT2,TNFAIP8,HPGDS,INSIG2,PIAS4,AWAT1,AWAT2,DGAT2L6,PLCD1
5_Member	GO Biolog	GO:00302	lipid modification	-3.74284	-0.683	10/245	28,30,3295	ABO,ACAA1,HSD17B4,PEX13,SOCS2,MTMR6,PIP5K1C,INPP5E,TMEM150A,GLT6D1
5_Member	Reactome	R-HSA-55	Metabolism of lipids	-2.51242	-0.133	16/739	30,2182,32	ACAA1,ACSL4,HSD17B4,SCD,MED22,MTMR6,AGPAT2,PIP5K1C,TNFAIP8,HPGDS,INSIG2,PIAS4,INPP5E,AWAT1,AWAT2,DGAT2L6
5_Member	Reactome	R-HSA-14	Phospholipid metabolism	-2.26592	-0.050	7/212	9107,1055	MTMR6,AGPAT2,PIP5K1C,TNFAIP8,INPP5E,AWAT2,DGAT2L6
5_Member	KEGG Path	hsa00562	Inositol phosphate metabolism	-2.26113	-0.050	4/71	5333,9107.	PLCD1,MTMR6,PIP5K1C,INPP5E
5_Member	GO Biolog	GO:00464	glycerolipid metabolic process	-2.04454	0.000	10/416	2182,8835.	ACSL4,SOCS2,MTMR6,AGPAT2,PIP5K1C,INSIG2,INPP5E,TMEM150A,AWAT2,DGAT2L6
5_Member	Reactome	R-HSA-14	PI Metabolism	-2.00645	0.000	4/84	9107,2339	MTMR6,PIP5K1C,TNFAIP8,INPP5E
6_Summar	GO Biolog	GO:00436	cellular protein complex disassembly	-3.40024	-0.427	9/222	822,6455,9	CAPG,SH3GL1,MRPL19,MRPL42,VILL,PTCD3,MRPL43,MRPL54,CAMSAP1,EEF2,RPL30,WNT8B,VAMP8,RPL7A
6_Member	GO Biolog	GO:00436	cellular protein complex disassembly	-3.40024	-0.427	9/222	822,6455,9	CAPG,SH3GL1,MRPL19,MRPL42,VILL,PTCD3,MRPL43,MRPL54,CAMSAP1
6_Member	GO Biolog	GO:00064	translational elongation	-3.36597	-0.427	7/136	1938,6156.	EEF2,RPL30,MRPL19,MRPL42,PTCD3,MRPL43,MRPL54
6_Member	GO Biolog	GO:00329	protein-containing complex disassembly	-3.19869	-0.411	11/340	822,6455,7	CAPG,SH3GL1,WNT8B,VAMP8,MRPL19,MRPL42,VILL,PTCD3,MRPL43,MRPL54,CAMSAP1
6_Member	CORUM	CORUM:32	39S ribosomal subunit, mitochondrial	-2.88251	-0.220	4/48	9801,2897	MRPL19,MRPL42,MRPL43,MRPL54
6_Member	Reactome	R-HSA-53	Mitochondrial translation initiation	-2.75261	-0.182	5/87	9801,2897	MRPL19,MRPL42,PTCD3,MRPL43,MRPL54
6_Member	Reactome	R-HSA-53	Mitochondrial translation elongation	-2.75261	-0.182	5/87	9801,2897	MRPL19,MRPL42,PTCD3,MRPL43,MRPL54
6_Member	Reactome	R-HSA-54	Mitochondrial translation termination	-2.75261	-0.182	5/87	9801,2897	MRPL19,MRPL42,PTCD3,MRPL43,MRPL54
6_Member	GO Biolog	GO:00701	mitochondrial translational elongation	-2.70903	-0.175	5/89	9801,2897	MRPL19,MRPL42,PTCD3,MRPL43,MRPL54
6_Member	GO Biolog	GO:00701	mitochondrial translational termination	-2.70903	-0.175	5/89	9801,2897	MRPL19,MRPL42,PTCD3,MRPL43,MRPL54
6_Member	Reactome	R-HSA-53	Mitochondrial translation	-2.62528	-0.151	5/93	9801,2897	MRPL19,MRPL42,PTCD3,MRPL43,MRPL54
6_Member	GO Biolog	GO:00064	translational termination	-2.39779	-0.096	5/105	9801,2897	MRPL19,MRPL42,PTCD3,MRPL43,MRPL54
6_Member	CORUM	CORUM:32	55S ribosome, mitochondrial	-2.13724	0.000	4/77	9801,2897	MRPL19,MRPL42,MRPL43,MRPL54
6_Member	Reactome	R-HSA-72	Translation	-2.0515	0.000	8/291	1938,6130.	EEF2,RPL7A,RPL30,MRPL19,MRPL42,PTCD3,MRPL43,MRPL54
7_Summar	GO Biolog	GO:00068	post-Golgi vesicle-mediated transport	-3.23824	-0.411	6/103	2803,8673.	GOLGA4,VAMP8,SEC16A,VAMP5,CCDC93,AP1AR,ENTR1
7_Member	GO Biolog	GO:00068	post-Golgi vesicle-mediated transport	-3.23824	-0.411	6/103	2803,8673.	GOLGA4,VAMP8,SEC16A,VAMP5,CCDC93,AP1AR
7_Member	GO Biolog	GO:00988	vesicle-mediated transport to the plasma membrane	-2.58499	-0.133	5/95	2803,9919.	GOLGA4,SEC16A,VAMP5,ENTR1,CCDC93
7_Member	GO Biolog	GO:00068	Golgi to plasma membrane transport	-2.49766	-0.133	4/61	2803,9919.	GOLGA4,SEC16A,VAMP5,CCDC93
7_Member	GO Biolog	GO:00430	Golgi to plasma membrane protein transport	-2.10735	0.000	3/41	2803,9919.	GOLGA4,SEC16A,VAMP5
8_Summar	Reactome	R-HSA-53	Uptake and actions of bacterial toxins	-3.22617	-0.411	4/39	1839,1938.	HBEGF,EEF2,MAP2K2,ANTXR2,DAPK3
8_Member	Reactome	R-HSA-53	Uptake and actions of bacterial toxins	-3.22617	-0.411	4/39	1839,1938.	HBEGF,EEF2,MAP2K2,ANTXR2
8_Member	KEGG Path	hsa05219	Bladder cancer	-2.10735	0.000	3/41	1613,1839.	DAPK3,HBEGF,MAP2K2
9_Summar	GO Biolog	GO:00485	developmental growth	-2.87306	-0.220	16/680	367,2803,2	AR,GOLGA4,GPR21,MATN2,NOTCH1,STK3,TRPC5,SOCS2,LHX2,PDLIM5,SIRT6,ATP8A2,BCL11A,RC3H2,SEMA4G,LZTS2,ATOH1,PAK3,PAX2,PALLD,PDZD7,AP1AR
9_Member	GO Biolog	GO:00485	developmental growth	-2.87306	-0.220	16/680	367,2803,2	AR,GOLGA4,GPR21,MATN2,NOTCH1,STK3,TRPC5,SOCS2,LHX2,PDLIM5,SIRT6,ATP8A2,BCL11A,RC3H2,SEMA4G,LZTS2
9_Member	GO Biolog	GO:00605	developmental growth involved in morphogenesis	-2.60077	-0.139	8/235	2803,4851.	GOLGA4,NOTCH1,TRPC5,LHX2,SIRT6,BCL11A,SEMA4G,LZTS2
9_Member	GO Biolog	GO:00486	regulation of developmental growth	-2.55996	-0.133	10/350	367,2803,2	AR,GOLGA4,GPR21,NOTCH1,STK3,TRPC5,SOCS2,ATP8A2,BCL11A,SEMA4G
9_Member	GO Biolog	GO:00488	neuron projection morphogenesis	-2.19679	-0.014	14/658	474,2803,4	ATOH1,GOLGA4,MATN2,NOTCH1,PAK3,PAX2,STK3,TRPC5,LHX2,PDLIM5,PALLD,ATP8A2,BCL11A,SEMA4G
9_Member	GO Biolog	GO:00486	cell morphogenesis involved in neuron differentiation	-2.14918	0.000	13/598	474,2803,4	ATOH1,GOLGA4,MATN2,NOTCH1,PAK3,PAX2,TRPC5,LHX2,PDLIM5,PALLD,ATP8A2,SEMA4G,PDZD7
9_Member	GO Biolog	GO:01200	plasma membrane bounded cell projection morphog	-2.12015	0.000	14/672	474,2803,4	ATOH1,GOLGA4,MATN2,NOTCH1,PAK3,PAX2,STK3,TRPC5,LHX2,PDLIM5,PALLD,ATP8A2,BCL11A,SEMA4G
9_Member	GO Biolog	GO:00488	cell projection morphogenesis	-2.09875	0.000	14/676	474,2803,4	ATOH1,GOLGA4,MATN2,NOTCH1,PAK3,PAX2,STK3,TRPC5,LHX2,PDLIM5,PALLD,ATP8A2,BCL11A,SEMA4G
9_Member	GO Biolog	GO:00074	axonogenesis	-2.06484	0.000	11/478	474,2803,4	ATOH1,GOLGA4,MATN2,NOTCH1,PAK3,PAX2,TRPC5,LHX2,PALLD,ATP8A2,SEMA4G
9_Member	GO Biolog	GO:00009	cell morphogenesis involved in differentiation	-2.05104	0.000	15/756	367,474,28	AR,ATOH1,GOLGA4,MATN2,NOTCH1,PAK3,PAX2,TRPC5,LHX2,PDLIM5,PALLD,ATP8A2,AP1AR,SEMA4G,PDZD7
9_Member	GO Biolog	GO:00485	developmental cell growth	-2.02137	0.000	7/236	2803,7224.	GOLGA4,TRPC5,LHX2,PDLIM5,SIRT6,BCL11A,SEMA4G
10_Summa	KEGG Path	hsa05215	Prostate cancer	-2.75261	-0.182	5/87	367,1147,5	AR,CHUK,MAP2K2,TCF7L1,CREB3L3
10_Membe	KEGG Path	hsa05215	Prostate cancer	-2.75261	-0.182	5/87	367,1147,5	AR,CHUK,MAP2K2,TCF7L1,CREB3L3
11_Summa	GO Biolog	GO:00456	negative regulation of myoblast differentiation	-2.72279	-0.175	3/25	4851,1001.	NOTCH1,SRA1,NMRK2,HIF1AN
11_Membe	GO Biolog	GO:00456	negative regulation of myoblast differentiation	-2.72279	-0.175	3/25	4851,1001.	NOTCH1,SRA1,NMRK2
11_Membe	GO Biolog	GO:00456	regulation of myoblast differentiation	-2.72179	-0.175	4/53	4851,1001.	NOTCH1,SRA1,NMRK2,HIF1AN
12_Summa	GO Biolog	GO:00073	regulation of mitotic cell cycle	-2.63524	-0.151	16/718	1613,5063.	DAPK3,PAK3,MAP2K2,PSMB7,STK3,CRADD,BTRC,OXSR1,SRA1,CTDSPL,NEK6,FZR1,SLF2,FSD1,NACC2,MIR26A1
12_Membe	GO Biolog	GO:00073	regulation of mitotic cell cycle	-2.63524	-0.151	16/718	1613,5063.	DAPK3,PAK3,MAP2K2,PSMB7,STK3,CRADD,BTRC,OXSR1,SRA1,CTDSPL,NEK6,FZR1,SLF2,FSD1,NACC2,MIR26A1
13_Summa	GO Biolog	GO:00060	regulation of morphogenesis of a branching structure	-2.63346	-0.151	4/56	367,5076,5	AR,PAX2,SIRT6,BCL11A,EDA,NOTCH1,STK3,LHX2,ATOH8,BTRC,LBX1,LZTS2,PURA,RIDA,EGFL7,MIR126,MIR26A1
13_Membe	GO Biolog	GO:00060	regulation of morphogenesis of a branching structure	-2.63346	-0.151	4/56	367,5076,5	AR,PAX2,SIRT6,BCL11A
13_Membe	GO Biolog	GO:00351	tube formation	-2.40525	-0.096	6/150	1896,4851.	EDA,NOTCH1,PAX2,STK3,LHX2,ATOH8
13_Membe	GO Biolog	GO:19053	positive regulation of morphogenesis of an epitheliur	-2.26564	-0.050	3/36	367,5076,5	AR,PAX2,SIRT6
13_Membe	GO Biolog	GO:00605	epithelial tube morphogenesis	-2.25027	-0.045	9/327	367,4851,5	AR,NOTCH1,PAX2,STK3,BTRC,LHX2,LBX1,SIRT6,LZTS2
13_Membe	GO Biolog	GO:00506	epithelial cell proliferation	-2.21763	-0.030	11/455	367,4851,5	AR,NOTCH1,PAX2,PURA,BTRC,RIDA,EGFL7,SIRT6,ATOH8,MIR126,MIR26A1
14_Summa	Reactome	R-HSA-88	E3 ubiquitin ligases ubiquitinate target proteins	-2.55039	-0.133	4/59	5194,7322.	PEX13,UBE2D2,UBE2N,RNF181,ACAA1,HSD17B4,NDUFB8,PMPCA,ACSL4
14_Membe	Reactome	R-HSA-88	E3 ubiquitin ligases ubiquitinate target proteins	-2.55039	-0.133	4/59	5194,7322.	PEX13,UBE2D2,UBE2N,RNF181
14_Membe	Reactome	R-HSA-90	Peroxisomal protein import	-2.4469	-0.106	4/63	30,3295,51	ACAA1,HSD17B4,PEX13,UBE2D2
14_Membe	GO Biolog	GO:00066	protein targeting to peroxisome	-2.32779	-0.077	4/68	30,3295,51	ACAA1,HSD17B4,PEX13,UBE2D2
14_Membe	GO Biolog	GO:00726	protein localization to peroxisome	-2.32779	-0.077	4/68	30,3295,51	ACAA1,HSD17B4,PEX13,UBE2D2
14_Membe	GO Biolog	GO:00726	establishment of protein localization to peroxisome	-2.32779	-0.077	4/68	30,3295,51	ACAA1,HSD17B4,PEX13,UBE2D2
14_Membe	GO Biolog	GO:00435	peroxisomal transport	-2.30519	-0.062	4/69	30,3295,51	ACAA1,HSD17B4,PEX13,UBE2D2
14_Membe	Reactome	R-HSA-96	Protein localization	-2.23104	-0.039	6/163	30,3295,47	ACAA1,HSD17B4,NDUFB8,PEX13,UBE2D2,PMPCA
14_Membe	Reactome	R-HSA-88	Protein ubiquitination	-2.09847	0.000	4/79	5194,7322.	PEX13,UBE2D2,UBE2N,RNF181
14_Membe	GO Biolog	GO:00070	peroxisome organization	-2.06085	0.000	4/81	30,3295,51	ACAA1,HSD17B4,PEX13,UBE2D2
14_Membe	KEGG Path	hsa04146	Peroxisome	-2.02432	0.000	4/83	30,2182,32	ACAA1,ACSL4,HSD17B4,PEX13
15_Summa	Reactome	R-HSA-88	Downregulation of ERBB2 signaling	-2.53443	-0.133	3/29	1839,4145.	HBEGF,MATK,NRG2,FGF5,MYD88,PIP5K1C,STK3,MTDH,MIR126
15_Membe	Reactome	R-HSA-88	Downregulation of ERBB2 signaling	-2.53443	-0.133	3/29	1839,4145.	HBEGF,MATK,NRG2
15_Membe	Reactome	R-HSA-68	PI5P, PP2A and IER3 Regulate PI3K/AKT Signaling	-2.43346	-0.106	5/103	1839,2250.	HBEGF,FGF5,MYD88,NRG2,PIP5K1C
15_Membe	GO Biolog	GO:00381	ERBB2 signaling pathway	-2.41122	-0.096	3/32	1839,4145.	HBEGF,MATK,NRG2
15_Membe	Reactome	R-HSA-19	Negative regulation of the PI3K/AKT network	-2.31211	-0.065	5/110	1839,2250.	HBEGF,FGF5,MYD88,NRG2,PIP5K1C
15_Membe	GO Biolog	GO:00518	positive regulation of protein kinase B signaling	-2.06242	0.000	6/177	1839,2250.	HBEGF,FGF5,STK3,NRG2,MTDH,MIR126
16_Summa	GO Biolog	GO:19010	negative regulation of potassium ion transmembrane	-2.53443	-0.133	3/29	9943,2363	OXSR1,KCNE5,MIR26A1,KCNS2
16_Membe	GO Biolog	GO:19010	negative regulation of potassium ion transmembrane	-2.53443	-0.133	3/29	9943,2363	OXSR1,KCNE5,MIR26A1
16_Membe	GO Biolog	GO:19010	regulation of potassium ion transmembrane transpor	-2.39798	-0.096	4/65	3788,9943.	KCNS2,OXSR1,KCNE5,MIR26A1
16_Membe	GO Biolog	GO:19013	negative regulation of potassium ion transmembrane	-2.37299	-0.083	3/33	9943,2363	OXSR1,KCNE5,MIR26A1
16_Membe	GO Biolog	GO:00432	negative regulation of potassium ion transport	-2.10735	0.000	3/41	9943,2363	OXSR1,KCNE5,MIR26A1
17_Summa	KEGG Path	hsa04730	Long-term depression	-2.52377	-0.133	4/60	2767,2892.	GNA11,GRIA3,GRID2,MAP2K2
17_Membe	KEGG Path	hsa04730	Long-term depression	-2.52377	-0.133	4/60	2767,2892.	GNA11,GRIA3,GRID2,MAP2K2
18_Summa	GO Biolog	GO:00017	cell fate specification	-2.48857	-0.133	5/100	367,474,48	AR,ATOH1,NOTCH1,PAX2,LBX1,INSIG2,ATP8A2,PDZD7,ATOH8,CDX4,PLCD1,STK3,CRB2,WNT8B,LHX2
18_Membe	GO Biolog	GO:00017	cell fate specification	-2.48857	-0.133	5/100	367,474,48	AR,ATOH1,NOTCH1,PAX2,LBX1
18_Membe	GO Biolog	GO:00424	inner ear morphogenesis	-2.43346	-0.106	5/103	474,5076,5	ATOH1,PAX2,INSIG2,ATP8A2,PDZD7
18_Membe	GO Biolog	GO:00308	positive regulation of epithelial cell differentiation	-2.32779	-0.077	4/68	474,4851,5	ATOH1,NOTCH1,PAX2,ATOH8
18_Membe	GO Biolog	GO:00485	embryonic organ development	-2.28784	-0.050	11/445	474,1046,4	ATOH1,CDX4,NOTCH1,PAX2,PLCD1,STK3,LBX1,INSIG2,ATP8A2,PDZD7,CRB2
18_Membe	GO Biolog	GO:00424	inner ear auditory receptor cell differentiation	-2.26564	-0.050	3/36	474,4851,7	ATOH1,NOTCH1,PDZD7
18_Membe	GO Biolog	GO:00485	embryonic morphogenesis	-2.13762	0.000	13/600	367,474,48	AR,ATOH1,NOTCH1,PAX2,STK3,WNT8B,LHX2,LBX1,INSIG2,ATP8A2,PDZD7,ATOH8,CRB2
18_Membe	GO Biolog	GO:00424	ear morphogenesis	-2.11008	0.000	5/123	474,5076,5	ATOH1,PAX2,INSIG2,ATP8A2,PDZD7
18_Membe	GO Biolog	GO:00017	cell fate determination	-2.07831	0.000	3/42	474,5076,1	ATOH1,PAX2,LBX1
18_Membe	GO Biolog	GO:00353	hair cell differentiation	-2.02253	0.000	3/44	474,4851,7	ATOH1,NOTCH1,PDZD7
19_Summa	GO Biolog	GO:19015	fatty acid derivative biosynthetic process	-2.48857	-0.133	5/100	2182,6319.	ACSL4,SCD,HPGDS,AWAT1,AWAT2,ACAA1,HSD17B4,VCAN,PEX13,NUP58,ERLIN1,INSIG2,PDZD11,ZBTB7A,SIRT6
19_Membe	GO Biolog	GO:19015	fatty acid derivative biosynthetic process	-2.48857	-0.133	5/100	2182,6319.	ACSL4,SCD,HPGDS,AWAT1,AWAT2
19_Membe	GO Biolog	GO:00016	long-chain fatty acid metabolic process	-2.32886	-0.077	5/109	30,2182,32	ACAA1,ACSL4,HSD17B4,HPGDS,AWAT1
19_Membe	GO Biolog	GO:00327	monocarboxylic acid metabolic process	-2.23615	-0.039	14/651	30,1462,21	ACAA1,VCAN,ACSL4,HSD17B4,PEX13,SCD,NUP58,ERLIN1,HPGDS,INSIG2,PDZD11,ZBTB7A,SIRT6,AWAT1
19_Membe	GO Biolog	GO:00335	unsaturated fatty acid metabolic process	-2.23108	-0.039	5/115	30,3295,63	ACAA1,HSD17B4,SCD,HPGDS,AWAT1
19_Membe	GO Biolog	GO:00723	monocarboxylic acid biosynthetic process	-2.13569	0.000	9/341	1462,3295.	VCAN,HSD17B4,SCD,NUP58,ERLIN1,HPGDS,INSIG2,ZBTB7A,SIRT6
19_Membe	GO Biolog	GO:19015	fatty acid derivative metabolic process	-2.12058	0.000	6/172	2182,3295.	ACSL4,HSD17B4,SCD,HPGDS,AWAT1,AWAT2
19_Membe	GO Biolog	GO:00353	fatty-acyl-CoA metabolic process	-2.10735	0.000	3/41	2182,3295.	ACSL4,HSD17B4,SCD
19_Membe	Reactome	R-HSA-89	Fatty acid metabolism	-2.06242	0.000	6/177	30,2182,32	ACAA1,ACSL4,HSD17B4,SCD,HPGDS,AWAT1
20_Summa	GO Biolog	GO:19001	negative regulation of protein localization to nucleus	-2.4508	-0.106	3/31	25885,844	POLR1A,LZTS2,DCLK3
20_Membe	GO Biolog	GO:19001	negative regulation of protein localization to nucleus	-2.4508	-0.106	3/31	25885,844	POLR1A,LZTS2,DCLK3

Supplementary Table s8:Cashmere yield and Genotype

DNA ID	Yield(kg)	FGF5	LXH2
HXR2061	0.26	del/del	del/del
HXR2169	0.39	del/del	del/del
HXR2802	0.2	del/del	del/del
HXR2315	0.21	del/del	del/del
HXR2227	0.3	del/del	del/del
HXR2791	0.35	del/del	del/del
HXR2779	0.18	del/del	del/del
HXR2798	0.28	del/del	del/del
HXR2190	0.31	del/del	del/del
HXR1926	0.4	del/del	del/del
HXR2788	0.23	del/del	del/del
HXR2063	0.23	del/del	del/del
HXR2219	0.29	del/del	del/del
HXR2163	0.37	del/del	del/del
HXR2346	0.5	del/del	del/del
HXR2482	0.31	del/del	del/del
HXR2827	0.21	del/del	del/del
HXR1957	0.26	del/del	del/del
HXR2787	0.39	del/del	del/del
HXR2444	0.29	del/del	del/del
HXR2476	0.38	del/del	del/del
HXR1454	0.23	del/del	del/del
HXR2082	0.23	del/del	del/del
HXR2090	0.48	del/del	del/del
HXR3125	0.2	del/del	del/del
HXR1067	0.39	del/del	del/del
HXR0736	0.41	del/del	del/del
HXR0550	0.31	del/del	del/del
HXR1927	0.36	del/del	del/del
HXR1821	0.38	del/del	del/del
HXR0716	0.41	del/del	del/del
HXR1717	0.45	del/del	del/del
HXR1933	0.5	del/del	del/del
HXR0690	0.51	del/del	del/del
HXR3139	0.19	del/del	del/del
HXR3214	0.21	del/del	del/del
HXR1143	0.24	del/del	del/del
HXR1477	0.24	del/del	del/del
HXR0960	0.28	del/del	del/del
HXR1798	0.31	del/del	del/del
HXR1130	0.33	del/del	del/del
HXR1068	0.42	del/del	del/del
HXR0979	0.45	del/del	del/del
HXR1189	0.33	del/del	del/del
HXR1392	0.34	del/del	del/del
HXR1237	0.29	del/del	del/del
HXR0954	0.38	del/del	del/del
HXR1398	0.44	del/del	del/del
HXR0825	0.31	del/del	del/del
HXR1812	0.34	del/del	del/del
HXR1046	0.32	del/del	del/del
HXR1833	0.32	del/del	del/del
HXR0879	0.4	del/del	del/del
HXR1581	0.42	del/del	del/del
HXR0900	0.22	del/del	del/del
HXR0889	0.26	del/del	del/del
HXR1830	0.26	del/del	del/del

HXR1841	0.33	del/del	del/del
HXR0850	0.38	del/del	del/del
HXR1100	0.43	del/del	del/del
HXR0888	0.45	del/del	del/del
HXR1121	0.3	del/del	del/del
HXR1129	0.42	del/del	del/del
HXR0186	0.13	del/del	del/del
HXR0370	0.59	del/del	del/del
HXR2781	0.15	del/del	del/del
HXR0981	0.45	del/del	del/del
HXR2752	0.15	del/del	del/del
HXR1321	0.53	del/del	del/del
HXR1630	0.34	del/del	del/del
HXR1319	0.87	del/del	del/del
HXR0775	0.85	del/del	del/del
HXR1092	0.76	del/del	del/del
HXR1583	0.76	del/del	del/del
HXR1574	0.71	del/del	del/del
HXR0765	0.66	del/del	del/del
HXR0909	0.62	del/del	del/del
HXR0882	0.62	del/del	del/del
HXR0750	0.62	del/del	del/del
HXR1802	0.61	del/del	del/del
HXR0971	0.59	del/del	del/del
HXR3135	0.58	del/del	del/del
HXR0880	0.58	del/del	del/del
HXR0864	0.57	del/del	del/del
HXR0770	0.57	del/del	del/del
HXR1559	0.56	del/del	del/del
HXR0970	0.56	del/del	del/del
HXR0792	0.56	del/del	del/del
HXR1387	0.56	del/del	del/del
HXR1199	0.56	del/del	del/del
HXR0777	0.56	del/del	del/del
HXR0881	0.55	del/del	del/del
HXR1313	0.54	del/del	del/del
HXR1318	0.53	del/del	del/del
HXR0836	0.52	del/del	del/del
HXR1796	0.52	del/del	del/del
HXR0949	0.51	del/del	del/del
HXR1158	0.19	del/del	del/del
HXR1928	0.16	del/del	del/del
HXR2815	0.14	del/del	del/del
HXR2776	0.14	del/del	del/del
HXR2762	0.13	del/del	del/del
HXR2766	0.13	del/del	del/del
HXR0851	0.13	del/del	del/del
HXR1627	0.13	del/del	del/del
HXR2893	0.11	del/del	del/del
HXR2771	0.3	del/del	del/+
HXR2806	0.24	del/del	del/+
HXR1939	0.26	del/del	del/+
HXR2196	0.31	del/del	del/+
HXR2006	0.36	del/del	del/+
HXR2769	0.2	del/del	del/+
HXR2200	0.24	del/del	del/+
HXR1937	0.38	del/del	del/+
HXR2370	0.23	del/del	del/+
HXR2210	0.19	del/del	del/+

HXR2166	0.21	del/del	del/+
HXR2758	0.22	del/del	del/+
HXR2606	0.26	del/del	del/+
HXR2573	0.28	del/del	del/+
HXR2612	0.27	del/del	del/+
HXR0963	0.37	del/del	del/+
HXR2091	0.43	del/del	del/+
HXR2372	0.43	del/del	del/+
HXR1571	0.25	del/del	del/+
HXR0962	0.48	del/del	del/+
HXR3120	0.34	del/del	del/+
HXR3162	0.14	del/del	del/+
HXR1438	0.22	del/del	del/+
HXR1309	0.25	del/del	del/+
HXR1070	0.33	del/del	del/+
HXR1378	0.47	del/del	del/+
HXR1069	0.23	del/del	del/+
HXR1326	0.29	del/del	del/+
HXR0887	0.3	del/del	del/+
HXR1233	0.33	del/del	del/+
HXR1123	0.35	del/del	del/+
HXR1192	0.35	del/del	del/+
HXR1236	0.35	del/del	del/+
HXR0941	0.37	del/del	del/+
HXR1091	0.43	del/del	del/+
HXR0937	0.45	del/del	del/+
HXR0739	0.27	del/del	del/+
HXR0925	0.26	del/del	del/+
HXR1555	0.27	del/del	del/+
HXR1122	0.42	del/del	del/+
HXR0878	0.51	del/del	del/+
HXR1566	0.29	del/del	del/+
HXR0867	0.35	del/del	del/+
HXR1082	0.36	del/del	del/+
HXR0875	0.37	del/del	del/+
HXR1557	0.38	del/del	del/+
HXR1769	0.42	del/del	del/+
HXR0837	0.43	del/del	del/+
HXR1317	0.5	del/del	del/+
HXR1560	0.23	del/del	del/+
HXR1111	0.27	del/del	del/+
HXR1572	0.36	del/del	del/+
HXR0127	0.29	del/del	del/+
HXR1104	0.75	del/del	del/+
HXR1314	0.66	del/del	del/+
HXR0806	0.6	del/del	del/+
HXR1103	0.58	del/del	del/+
HXR2804	0.55	del/del	del/+
HXR0781	0.54	del/del	del/+
HXR1248	0.19	del/del	del/+
HXR1093	0.18	del/del	del/+
HXR1308	0.18	del/del	del/+
HXR2811	0.17	del/del	del/+
HXR0812	0.16	del/del	del/+
HXR1112	0.16	del/del	del/+
HXR2777	0.14	del/del	del/+
HXR1468	0.13	del/del	del/+
HXR1133	0.12	del/del	del/+
HXR2199	0.21	del/del	+/+

HXR1924	0.36	del/del	+/+
HXR2234	0.28	del/del	+/+
HXR2639	0.25	del/del	+/+
HXR2619	0.35	del/del	+/+
HXR2814	0.21	del/del	+/+
HXR1792	0.25	del/del	+/+
HXR1459	0.37	del/del	+/+
HXR2363	0.43	del/del	+/+
HXR1304	0.42	del/del	+/+
HXR1079	0.43	del/del	+/+
HXR1591	0.2	del/del	+/+
HXR1653	0.18	del/del	+/+
HXR1651	0.12	del/del	+/+
HXR2395	0.38	del/+	del/del
HXR2261	0.13	del/+	del/del
HXR1974	0.22	del/+	del/del
HXR2168	0.19	del/+	del/del
HXR2551	0.24	del/+	del/del
HXR0439	0.39	del/+	del/del
HXR3226	0.26	del/+	del/del
HXR3196	0.32	del/+	del/del
HXR3165	0.3	del/+	del/del
HXR0890	0.34	del/+	del/del
HXR0760	0.42	del/+	del/del
HXR1466	0.32	del/+	del/del
HXR0766	0.33	del/+	del/del
HXR1828	0.2	del/+	del/del
HXR0844	0.28	del/+	del/del
HXR0835	0.47	del/+	del/del
HXR0866	0.49	del/+	del/del
HXR1787	0.28	del/+	del/del
HXR0904	0.19	del/+	del/del
HXR1993	0.17	del/+	del/del
HXR2179	0.22	del/+	del/+
HXR2160	0.26	del/+	del/+
HXR2794	0.41	del/+	del/+
HXR2367	0.31	del/+	del/+
HXR2379	0.34	del/+	del/+
HXR2170	0.32	del/+	del/+
HXR2193	0.22	del/+	del/+
HXR1200	0.21	del/+	del/+
HXR0758	0.35	del/+	del/+
HXR1196	0.21	del/+	del/+
HXR1136	0.24	del/+	del/+
HXR1128	0.27	del/+	del/+
HXR0870	0.35	del/+	del/+
HXR1108	0.35	del/+	del/+
HXR1097	0.4	del/+	del/+
HXR0936	0.42	del/+	del/+
HXR0892	0.44	del/+	del/+
HXR0883	0.48	del/+	del/+
HXR1110	0.48	del/+	del/+
HXR0877	0.26	del/+	del/+
HXR2377	0.18	del/+	del/+
HXR2368	0.17	del/+	del/+
HXR2783	0.44	del/+	+/+
HXR1569	0.19	del/+	+/+
HXR2770	0.16	+/+	del/del
HXR1389	0.15	+/+	del/+

HXR0746	0.35	+/+	+/+

Supplementary Table s9:Cashmere diameter and Gnotype

Sample	Diameter（um)	FGF5	LHX2
HXR3749	15.27	del/del	del/del
HXR3752	14.74	del/del	del/del
HXR3176	13.81	del/+	del/+
HXR3149	14.85	del/del	del/del
HXR3144	14.09	del/del	del/del
HXR3581	16.47	del/del	del/del
HXR3152	14.05	del/del	del/del
HXR2445	16.22	del/del	del/del
HXR3750	14.07	del/+	del/+
HXR3738	14.01	del/+	del/+
HXR3352	15.39	del/del	del/del
HXR3745	14.7	del/del	del/del
HXR3739	13.3	del/del	del/del
HXR3138	14.07	del/+	del/+
HXR3315	17.06	del/del	del/del
HXR3288	14.85	del/del	del/del
HXR3742	13.94	del/+	del/+
HXR3748	14.71	del/del	del/del
HXR2457	15.68	del/del	del/del
HXR1484	13.87	del/+	del/+
HXR2531	14.24	del/del	del/del
HXR3257	13.91	del/del	del/del
HXR3665	14.09	del/del	del/del
HXR3103	15.74	del/del	del/del
HXR3119	15.22	del/del	del/del
HXR3401	15.56	+/+	+/+
HXR3334	14.05	del/del	del/del
HXR3083	13.83	del/del	del/del
HXR3669	16.75	del/del	del/del
HXR3151	13.68	del/del	del/del
HXR3680	14.67	del/+	del/+
HXR3110	15.53	del/+	del/+
HXR3142	15.35	del/del	del/del
HXR3376	16.57	del/del	del/del
HXR3548	15.11	del/+	del/+
HXR3082	15.42	del/del	del/del
HXR3715	13.28	del/del	del/del
HXR3565	14.1	del/del	del/del
HXR3080	15.12	del/del	del/del
HXR3161	14.23	del/+	del/+
HXR3170	14.07	del/del	del/del
HXR3564	14.93	del/+	del/+
HXR3604	14.04	del/del	del/del
HXR3134	15.54	del/del	del/del
HXR3117	14.97	del/del	del/del
HXR3222	14.19	del/del	del/del
HXR3158	14.1	del/del	del/del
HXR3636	13.6	del/del	del/del
HXR3156	15.95	del/del	del/del
HXR3706	13.95	del/del	del/del
HXR3092	14.22	del/del	del/del
HXR3107	15.09	del/del	del/del
HXR3385	14.04	del/del	del/del
HXR3373	14.53	del/del	del/del
HXR3155	13.18	del/del	del/del
HXR3681	14.24	del/del	del/del
HXR3606	15.32	del/del	del/del

HXR3612	14.98	del/del	del/del
HXR3407	13.49	del/del	del/del
HXR3381	13.52	del/del	del/del
HXR3705	14.71	del/+	del/+
HXR3619	14.28	del/del	del/del
HXR3095	14.76	del/del	del/del
HXR3403	14.1	del/del	del/del
HXR3224	14.17	del/del	del/del
HXR3357	13.02	del/+	del/+
HXR3545	15.72	del/del	del/del
HXR3114	15.46	del/del	del/del
HXR3195	13.45	del/del	del/del
HXR3690	13.04	del/del	del/del
HXR3201	15.45	del/del	del/del
HXR3252	16.09	del/del	del/del
HXR3356	13.98	del/+	del/+
HXR3194	13.73	del/del	del/del
HXR3314	14.78	del/del	del/del
HXR3379	14.27	del/del	del/del
HXR3459	13.55	del/del	del/del
HXR3611	13.61	del/del	del/del
HXR3589	13.82	del/del	del/del
HXR3175	13.31	del/del	del/del
HXR3181	15.95	del/del	del/del
HXR3337	14.27	+/+	+/+
HXR3311	15.15	del/del	del/del
HXR3206	13.47	del/del	del/del
HXR3363	13.78	del/del	del/del
HXR3569	15.89	del/del	del/del
HXR3653	14.19	del/+	del/+
HXR3320	14.45	del/del	del/del
HXR3448	14.06	del/del	del/del
HXR3384	13.74	del/del	del/del
HXR3291	14.16	del/del	del/del
HXR3672	14.12	del/del	del/del
HXR3213	13.99	del/del	del/del
HXR3264	16.74	del/del	del/del
HXR3346	13.49	del/del	del/del
HXR3622	13.96	del/+	del/+
HXR3277	15.82	del/del	del/del
HXR3228	15.71	del/del	del/del
HXR3247	15.98	del/del	del/del
HXR3338	14.92	del/del	del/del
HXR3639	13.91	del/del	del/del
HXR3321	12.85	del/del	del/del
HXR3391	14.17	del/+	del/+
HXR3367	15.16	NaN	NaN
HXR3347	15.71	del/+	del/+
HXR3628	14.05	del/del	del/del
HXR3280	13.9	del/del	del/del
HXR3483	14.27	del/del	del/del
HXR3618	15.28	del/del	del/del
HXR3306	16.14	del/+	del/+
HXR3260	15.97	del/del	del/del
HXR3290	15.27	del/del	del/del
HXR3431	13.49	del/del	del/del
HXR3212	15.5	del/del	del/del
HXR3269	15.21	del/del	del/del
HXR3716	14.98	del/del	del/del

HXR3325	14.56	del/del	del/del
HXR3655	15.06	del/+	del/+
HXR3249	15.21	del/del	del/del
HXR3259	14.96	del/+	del/+
HXR3366	14.23	del/del	del/del
HXR3230	17.1	del/+	del/+
HXR3626	13.23	del/+	del/+
HXR3319	15.37	del/del	del/del
HXR3707	14.25	del/del	del/del
HXR3266	14.27	del/del	del/del
HXR3236	14.13	del/del	del/del
HXR3422	14.92	del/del	del/del
HXR3293	14.19	del/+	del/+
HXR3393	13.75	del/del	del/del
HXR3268	15.28	del/+	del/+
HXR3730	15.26	del/+	del/+
HXR3425	13.96	del/+	del/+
HXR3503	15.37	del/del	del/del
HXR3241	16.29	del/del	del/del
HXR3454	17.24	del/del	del/del
HXR3714	14.06	del/+	del/+
HXR3392	15.02	del/del	del/del
HXR3426	16.31	del/+	del/+
HXR3645	16.17	del/del	del/del
HXR3262	14.06	del/del	del/del
HXR3734	14.64	del/del	del/del
HXR3317	14.26	del/del	del/del
HXR3255	15	del/del	del/del
HXR3332	14.91	del/del	del/del
HXR3427	15.32	del/del	del/del
HXR3736	13.69	del/del	del/del
HXR3467	14.02	del/del	del/del
HXR3396	14.91	del/del	del/del
HXR3336	13.99	del/del	del/del
HXR3343	14.44	del/+	del/+
HXR3499	14.61	del/del	del/del
HXR3272	13.89	del/del	del/del
HXR3479	14.39	del/del	del/del
HXR3254	14.87	del/del	del/del
HXR3186	15.23	del/del	del/del
HXR3284	15.94	del/+	del/+
HXR2351	14.01	del/+	del/+
HXR3472	14.65	del/del	del/del
HXR3686	13.25	del/del	del/del
HXR3525	14.75	del/del	del/del
HXR3733	13.49	del/del	del/del
HXR3221	15	del/del	del/del
HXR3558	13.57	del/del	del/del
HXR3512	14.71	del/del	del/del
HXR3718	14.27	del/del	del/del
HXR3735	15.69	del/+	del/+
HXR3722	14.3	del/del	del/del
HXR3726	12.94	del/del	del/del
HXR3737	13.55	del/del	del/del
HXR3741	13.79	del/del	del/del
HXR3132	13.83	del/del	del/del
HXR3128	13.86	del/del	del/del
HXR3101	14.87	del/del	del/del
HXR3615	13.68	del/del	del/del

HXR3532	13.79	del/del	del/del
HXR3183	14.9	del/del	del/del
HXR3547	12.8	del/del	del/del
HXR3670	13.52	del/del	del/del
HXR3140	14.55	del/del	del/del
HXR3112	15.7	del/del	del/del
HXR3552	14.66	del/del	del/del
HXR3660	13.98	del/+	del/+
HXR3097	15.05	del/del	del/del
HXR3113	13.89	del/del	del/del
HXR3100	13.58	del/+	del/+
HXR3387	13.87	del/del	del/del
HXR3232	13.54	del/del	del/del
HXR3751	13.52	del/del	del/del
HXR3555	13.53	del/del	del/del
HXR3189	13.8	del/del	del/del
HXR3709	14.99	del/del	del/del
HXR3755	13.86	del/+	del/+
HXR3300	13.27	del/del	del/del
HXR3182	14.33	del/del	del/del
HXR3324	13.49	del/del	del/del
HXR3530	14.99	del/+	del/+
HXR3369	13.82	del/+	del/+
HXR3551	13.51	del/del	del/del
HXR3187	15.03	del/del	del/del
HXR3326	13.8	del/del	del/del
HXR3331	14.46	del/+	del/+
HXR3372	13.57	del/del	del/del
HXR3286	14.8	del/del	del/del
HXR3434	14.3	del/del	del/del
HXR3754	14.56	del/del	del/del
HXR3531	14.68	del/del	del/del
HXR3245	14.93	del/del	del/del
HXR3333	13.29	del/del	del/del
HXR3541	14.78	del/del	del/del
HXR3701	14.03	del/del	del/del
HXR3143	14.12	del/del	del/del
HXR3188	14.15	del/del	del/del
HXR3178	13.83	del/del	del/del
HXR3218	14.52	del/del	del/del
HXR3542	13.4	del/del	del/del
HXR3374	13.98	del/del	del/del
HXR3238	15.47	del/del	del/del
HXR3534	13.79	del/+	del/+
HXR3538	14.95	del/del	del/del
HXR3190	13.81	del/del	del/del
HXR3362	15.03	del/del	del/del
HXR3378	13.75	del/del	del/del
HXR3398	13.67	del/del	del/del
HXR3674	13.86	del/del	del/del
HXR3410	14.46	del/del	del/del
HXR3485	13.3	del/del	del/del
HXR3327	13.48	del/del	del/del
HXR3520	13.17	del/+	del/+
HXR3447	14.25	del/del	del/del
HXR3671	14.17	+/+	+/+
HXR3437	14.59	del/del	del/del
HXR3480	13.47	+/+	+/+
HXR3527	14.82	del/del	del/del

HXR3282	14.09	del/del	del/del
HXR3518	15.22	del/del	del/del
HXR3445	13.48	del/+	del/+
HXR3219	13.74	del/del	del/del
HXR3684	14.29	del/+	del/+
HXR3438	13.83	del/del	del/del
HXR3220	13.89	del/del	del/del
HXR3675	15.56	del/del	del/del
HXR3510	14.45	del/+	del/+
HXR3743	15.28	del/del	del/del
HXR3516	14.63	del/del	del/del
HXR3420	13.7	del/del	del/del
HXR3478	13.27	del/del	del/del
HXR3428	14.04	del/del	del/del
HXR3446	14.87	del/+	del/+
HXR3469	13.66	del/del	del/del
HXR3513	14.85	+/+	+/+
HXR3484	13.3	del/+	del/+
HXR3413	13.84	del/+	del/+
HXR3720	13.76	del/del	del/del
HXR3329	13.32	del/del	del/del
HXR3539	13.79	del/del	del/del
HXR3526	14.92	del/+	del/+
HXR3414	13.26	del/del	del/del
HXR3481	13.82	del/del	del/del
HXR3505	14.56	del/del	del/del
HXR3517	13.8	del/del	del/del
HXR3486	14.86	del/del	del/del
HXR3500	12.85	del/del	del/del
HXR3271	13.85	del/del	del/del
HXR0988	12.98	del/del	del/del
HXR3506	14.32	del/del	del/del
HXR3239	14.37	del/+	del/+
HXR1387	14.06	del/del	del/del
HXR0851	13.76	del/del	del/del
HXR0777	13.92	del/del	del/del
HXR1313	13.76	del/del	del/del
HXR0824	14.13	NaN	NaN
HXR0806	13.2	del/+	del/del
HXR0745	14.08	+/+	del/del
HXR0781	13.8	del/+	del/del
HXR1314	14.06	del/+	del/del
HXR0775	13.88	del/del	del/del
HXR0792	13.32	del/del	del/del
HXR0761	13.61	NaN	NaN
HXR1092	14	del/del	del/del
HXR0770	13.29	del/del	del/del
HXR1583	13.92	del/del	del/del
HXR1370	13.12	del/+	NaN
HXR1318	13.22	del/del	del/del
HXR1389	13.54	del/+	+/+
HXR1248	13.98	del/+	del/del
HXR1103	13.92	del/+	del/del
HXR1802	13.79	del/del	del/del
HXR0882	13.8	del/del	del/del
HXR1574	13.19	del/del	del/del
HXR1093	13.88	del/+	del/del
HXR1796	13.96	del/del	del/del
HXR1319	14.1	del/del	del/del

HXR0904	13.28	del/del	del/+
HXR1569	14.05	+/+	del/+
HXR1104	13.78	del/+	del/del
HXR0864	13.71	del/del	del/del
HXR0881	13.8	del/del	del/del
HXR0836	14.01	del/del	del/del
HXR0880	13.58	del/del	del/del
HXR0909	13.66	del/del	del/del
HXR0902	13.34	NaN	NaN
HXR1834	14.13	NaN	del/del
HXR2607	14.05	NaN	NaN
HXR2377	14.01	del/+	del/+
HXR2368	13.89	del/+	del/+
HXR2804	13.3	del/+	del/del
HXR1559	13.88	del/del	del/del
HXR0750	13.67	del/del	del/del
HXR0765	13.91	del/del	del/del
HXR2068	16.12	del/del	NaN
HXR0812	13.2	del/+	del/del
HXR0798	13.6	NaN	del/del
HXR1627	14.08	del/del	del/del
HXR1468	13.29	del/+	del/del
HXR1653	14.17	+/+	del/del
HXR1651	14.07	+/+	del/del
HXR0971	13.71	del/del	del/del
HXR1308	13.66	del/+	del/del
HXR1199	13.66	del/del	del/del
HXR0949	13.87	del/del	del/del
HXR1133	13.28	del/+	del/del
HXR1112	13.46	del/+	del/del
HXR2243	13.92	del/del	del/del
HXR2815	12.54	del/del	del/del
HXR2811	14.05	del/+	del/del
HXR2766	12.45	del/del	del/del
HXR2770	12.37	del/del	+/+
HXR2893	15.18	del/del	del/del
HXR2762	14	del/del	del/del
HXR2777	12.92	del/+	del/del
HXR2749	14.04	del/del	del/del
HXR1993	14.18	del/del	del/+
HXR2826	12.69	del/del	del/+
HXR2764	13.11	NaN	del/del
HXR3135	15.27	del/del	del/del
HXR3168	13.91	del/del	del/del
HXR2776	12.79	del/del	del/del
HXR0970	13.68	del/del	del/del
HXR1158	13.76	del/del	del/del
HXR1928	13.9	del/del	del/del
HXR0127	13.53	del/del	del/+
HXR0186	13.09	del/del	del/del
HXR0370	13.42	del/del	del/del
HXR1387	14.06	del/del	del/del
HXR1392	13.72	del/del	del/del
HXR1046	13.39	del/del	del/del
HXR3162	14.08	del/del	del/+
HXR1459	13.59	del/del	+/+
HXR0439	14.05	del/+	del/del
HXR0690	13.98	del/del	del/del
HXR0550	14.15	del/del	del/del

HXR0716	14.12	del/del	del/del
HXR0851	13.76	del/del	del/del
HXR0739	13.35	del/del	del/+
HXR0777	13.92	del/del	del/del
HXR0825	13.69	del/del	del/del
HXR1313	13.76	del/del	del/del
HXR0806	13.2	del/del	del/+
HXR0745	14.08	del/del	+/+
HXR0781	13.8	del/del	del/+
HXR0766	14	del/+	del/del
HXR1812	13.38	del/del	del/del
HXR1314	14.06	del/del	del/+
HXR0758	13.43	del/+	del/+
HXR1237	13.58	del/del	del/del
HXR0775	13.88	del/del	del/del
HXR0792	13.32	del/del	del/del
HXR1092	14	del/del	del/del
HXR1398	13.56	del/del	del/del
HXR0770	13.29	del/del	del/del
HXR0760	13.72	del/+	del/del
HXR1583	13.92	del/del	del/del
HXR1378	13.51	del/del	del/+
HXR1070	14.38	del/del	del/+
HXR1798	14.02	del/del	del/del
HXR1143	13.66	del/del	del/del
HXR0746	13.77	+/+	+/+
HXR1091	12.86	del/del	del/+
HXR0890	13.04	del/+	del/del
HXR0937	13.58	del/del	del/+
HXR1326	13.13	del/del	del/+
HXR1318	13.22	del/del	del/del
HXR1123	14.01	del/del	del/+
HXR1130	13.68	del/del	del/del
HXR1321	12.86	del/del	del/del
HXR0887	13.47	del/del	del/+
HXR1069	13.36	del/del	del/+
HXR1068	13.13	del/del	del/del
HXR1236	13.48	del/del	del/+
HXR1233	14.13	del/del	del/+
HXR1389	13.54	+/+	del/+
HXR1248	13.98	del/del	del/+
HXR1129	13.74	del/del	del/del
HXR1103	13.92	del/del	del/+
HXR1802	13.79	del/del	del/del
HXR1079	14.15	del/del	+/+
HXR1572	14.03	del/del	del/+
HXR1121	14.04	del/del	del/del
HXR1560	13.57	del/del	del/+
HXR1111	14.07	del/del	del/+
HXR1787	13.74	del/+	del/del
HXR0882	13.8	del/del	del/del
HXR1581	13.19	del/del	del/del
HXR0883	13.92	del/+	del/+
HXR1574	13.19	del/del	del/del
HXR1110	13.87	del/+	del/+
HXR1108	14	del/+	del/+
HXR1093	13.88	del/del	del/+
HXR1796	13.96	del/del	del/del
HXR0844	13.91	del/+	del/del

HXR0878	13.68	del/del	del/+
HXR0879	13.02	del/del	del/del
HXR1122	13.58	del/del	del/+
HXR1833	14.07	del/del	del/del
HXR1136	13.66	del/+	del/+
HXR0892	13.63	del/+	del/+
HXR1555	13.09	del/del	del/+
HXR1828	13.84	del/+	del/del
HXR0866	13.85	del/+	del/del
HXR1097	13.26	del/+	del/+
HXR0925	13.57	del/del	del/+
HXR0870	14.16	del/+	del/+
HXR0936	14.12	del/+	del/+
HXR1319	14.1	del/del	del/del
HXR0904	13.28	del/+	del/del
HXR0889	13.98	del/del	del/del
HXR1566	13.68	del/del	del/+
HXR1569	14.05	del/+	+/+
HXR1082	14.02	del/del	del/+
HXR0888	13.78	del/del	del/del
HXR1104	13.78	del/del	del/+
HXR1317	14.02	del/del	del/+
HXR1830	13.91	del/del	del/del
HXR0875	12.84	del/del	del/+
HXR0864	13.71	del/del	del/del
HXR0850	14.04	del/del	del/del
HXR0881	13.8	del/del	del/del
HXR0867	13.89	del/del	del/+
HXR0836	14.01	del/del	del/del
HXR0900	13.1	del/del	del/del
HXR0837	14.15	del/del	del/+
HXR1769	12.94	del/del	del/+
HXR1841	13.66	del/del	del/del
HXR1100	13.64	del/del	del/del
HXR0880	13.58	del/del	del/del
HXR0909	13.66	del/del	del/del
HXR0877	13.33	del/+	del/+
HXR2551	13.88	del/+	del/del
HXR2444	13.99	del/del	del/del
HXR2612	13.39	del/del	del/+
HXR2476	13.53	del/del	del/del
HXR2606	13.97	del/del	del/+
HXR2370	13.11	del/del	del/+
HXR2377	14.01	del/+	del/+
HXR2482	13.85	del/del	del/del
HXR2619	13.77	del/del	+/+
HXR2639	13.81	del/del	+/+
HXR2368	13.89	del/+	del/+
HXR2379	13.81	del/+	del/+
HXR2787	13.86	del/del	del/del
HXR2791	13.47	del/del	del/del
HXR2804	13.3	del/del	del/+
HXR2802	13.86	del/del	del/del
HXR2395	14.07	del/+	del/del
HXR2063	14.52	del/del	del/del
HXR2794	13.62	del/+	del/+
HXR2788	12.54	del/del	del/del
HXR2006	14.05	del/del	del/+
HXR2061	14.06	del/del	del/del

HXR2806	13.42	del/del	del/+
HXR2798	14.74	del/del	del/del
HXR0736	13.37	del/del	del/del
HXR1559	13.88	del/del	del/del
HXR1571	13.14	del/del	del/+
HXR1067	14.07	del/del	del/del
HXR0750	13.67	del/del	del/del
HXR2082	14.67	del/del	del/del
HXR0765	13.91	del/del	del/del
HXR2372	13.89	del/del	del/+
HXR0812	13.2	del/del	del/+
HXR2090	15.79	del/del	del/del
HXR1792	13.92	del/del	+/+
HXR2091	13.91	del/del	del/+
HXR1821	14.13	del/del	del/del
HXR1627	14.08	del/del	del/del
HXR1468	13.29	del/del	del/+
HXR1466	13.3	del/+	del/del
HXR1653	14.17	del/del	+/+
HXR0954	13.75	del/del	del/del
HXR1651	14.07	del/del	+/+
HXR1200	13.52	del/+	del/+
HXR0981	13.03	del/del	del/del
HXR1477	13.08	del/del	del/del
HXR1309	13.33	del/del	del/+
HXR1304	14	del/del	+/+
HXR0971	13.71	del/del	del/del
HXR1308	13.66	del/del	del/+
HXR0941	14.18	del/del	del/+
HXR1189	13.1	del/del	del/del
HXR0979	14.19	del/del	del/del
HXR1192	14.05	del/del	del/+
HXR1199	13.66	del/del	del/del
HXR0949	13.87	del/del	del/del
HXR1133	13.28	del/del	del/+
HXR1591	13.51	del/del	+/+
HXR1112	13.46	del/del	del/+
HXR1128	13.7	del/+	del/+
HXR1196	13.88	del/+	del/+
HXR0835	13.95	del/+	del/del
HXR1630	13.59	del/del	del/del
HXR1557	14.1	del/del	del/+
HXR2243	13.92	del/del	del/del
HXR2573	14.24	del/del	del/+
HXR2346	14.2	del/del	del/del
HXR2783	13.51	del/+	+/+
HXR2815	12.54	del/del	del/del
HXR2814	14.04	del/del	+/+
HXR2210	13.19	del/del	del/+
HXR2827	14.12	del/del	del/del
HXR2758	12.53	del/del	del/+
HXR2227	14.23	del/del	del/del
HXR2811	14.05	del/del	del/+
HXR2315	14.09	del/del	del/del
HXR2766	12.45	del/del	del/del
HXR2770	12.37	+/+	del/del
HXR2367	13.44	del/+	del/+
HXR2234	13.82	del/del	+/+
HXR1974	13.97	del/+	del/del

HXR2769	13.91	del/del	del/+
HXR2893	15.18	del/del	del/del
HXR2219	13.74	del/del	del/del
HXR2752	12.62	del/del	del/del
HXR2762	14	del/del	del/del
HXR2781	12.54	del/del	del/del
HXR2777	12.92	del/del	del/+
HXR2749	14.04	del/del	del/del
HXR1993	14.18	del/+	del/del
HXR2771	13.88	del/del	del/+
HXR2826	12.69	del/+	del/del
HXR2199	13.39	del/del	+/+
HXR2779	13.7	del/del	del/del
HXR3135	15.27	del/del	del/del
HXR3165	14.11	del/+	del/del
HXR3168	13.91	del/del	del/del
HXR3214	14.75	del/del	del/del
HXR2776	12.79	del/del	del/del
HXR3125	14.97	del/del	del/del
HXR2363	14.06	del/del	+/+
HXR0970	13.68	del/del	del/del
HXR1454	14.15	del/del	del/del
HXR0963	13.64	del/del	del/+
HXR1717	13.63	del/del	del/del
HXR0962	14.1	del/del	del/+
HXR1158	13.76	del/del	del/del
HXR1438	13.79	del/del	del/+
HXR0960	13.37	del/del	del/del
HXR1928	13.9	del/del	del/del
HXR2166	13.85	del/del	del/+
HXR1957	13.73	del/del	del/del
HXR2170	13.8	del/+	del/+
HXR2168	13.71	del/+	del/del
HXR1941	12.69	del/del	+/+
HXR1937	13.19	del/del	del/+
HXR2200	13.88	del/del	del/+
HXR2163	13.13	del/del	del/del
HXR1939	13.72	del/del	del/+
HXR2160	13.27	del/+	del/+
HXR2196	13.02	del/del	del/+
HXR1924	12.93	del/del	+/+
HXR2179	13.13	del/+	del/+
HXR2169	12.98	del/del	del/del
HXR2261	13.8	del/+	del/del
HXR1926	14.92	del/del	del/del
HXR2190	13.76	del/del	del/del
HXR3139	14.78	del/del	del/del
HXR3225	13.58	del/del	+/+
HXR2193	13.37	del/+	del/+
HXR3226	14.24	del/+	del/del
HXR1927	12.88	del/del	del/del
HXR1933	13.84	del/del	del/del

Variant analysis

Number of effects by type and region

Type (alphabetical order)	Count	Percent
3_prime_UTR_variant	143948	0.00379
UTR_premature_start_codon_ga	4579	0.00012
5_prime_UTR_variant	26854	0.00071
downstream_gene_variant	1861794	0.04906
initiator_codon_variant	8	0
intergenic_region	8165402	0.21517
intragenic_variant	150948	0.00398
intron_variant	12728720	0.33542
missense_variant	69410	0.00183
on_coding_transcript_exon_varia	47626	0.00126
non_coding_transcript_variant	12788241	0.33699
splice_acceptor_variant	214	0.00001
splice_donor_variant	294	0.00001
splice_region_variant	21247	0.00056
start_lost	80	0
stop_gained	720	0.00002
stop_lost	104	0
stop_retained_variant	79	0
synonymous_variant	114308	0.00301
upstream_gene_variant	1824134	0.04807

Type (alphabetical order)	Count	Percent
DOWNSTREAM	1,861,794	4.91%
EXON	229,487	0.61%
INTERGENIC	8,165,402	21.53%
INTRON	12,711,669	33.52%
SPLICE_SITE_ACCEPTOR	214	0.00%
SPLICE_SITE_DONOR	286	0.00%
SPLICE_SITE_REGION	19,970	0.05%
TRANSCRIPT	12,939,189	34.12%
UPSTREAM	1,824,134	4.81%
UTR_3_PRIME	143,948	0.38%
UTR_5_PRIME	31,433	0.08%

Transition, transversions,heterozygous,homozygous per sample

	Transitions	ansversion	Ts/Tv	Het	Hom	Missing
CMS0019	4837778	1965207	2.462	2814879	1994053	32720
CMS0026	4886197	1989777	2.456	2688936	2093519	25286
CMS0037	4873889	1982624	2.458	2812859	2021827	21409
CMS0053	4865412	1980185	2.457	2750591	2047503	27876
CMS0065	4750755	1933162	2.458	2678155	2002881	68267
CMS0084	4933740	2008058	2.457	2834852	2053473	11607
CMS0088	4918530	2000855	2.458	3064929	1927228	9696
CMS0100	4856598	1974396	2.46	2837910	1996542	35968
CMS0177	4896586	1991074	2.459	3236108	1825776	12688
CMS0330	4938887	2009693	2.458	2908936	2019822	14731
CMS0406	4807382	1955796	2.458	2615124	2074027	44354
CMS0431	4738301	1929768	2.455	2903727	1882171	65227
FANG0001	4679623	1901423	2.461	3087782	1746632	6758
FANG0002	4633702	1883595	2.46	2438695	2039301	27605
FANG0003	4225263	1713969	2.465	2296948	1821142	237898
FANG0004	4575046	1858633	2.462	3035373	1699153	24711
FANG0005	4601259	1871377	2.459	2827144	1822746	26259
FANG0006	4497697	1827831	2.461	2543452	1891038	69935
FANG0007	4581889	1861965	2.461	3000810	1721522	23577
FANG0008	4550894	1847958	2.463	2883732	1757560	43262
FANG0009	4569499	1857683	2.46	3008380	1709401	36287
FANG0010	4120058	1673279	2.462	2184041	1804648	376968
FANG0011	4677072	1900764	2.461	3194428	1691704	11439
FANG0012	4291664	1742635	2.463	2030787	2001756	230340
FANG0013	4681846	1906700	2.455	3317998	1635274	6493
FANG0014	4584488	1863026	2.461	2794838	1826338	19093
FANG0015	4626702	1883543	2.456	3267309	1621468	18420
FANG0016	4579646	1861035	2.461	2910423	1765129	31794
FANG0017	4655770	1893673	2.459	3172693	1688375	12136
FANG0018	4624320	1879983	2.46	3160291	1672006	18259
FANG0019	4593648	1869683	2.457	3166801	1648265	16421
FANG0020	4588270	1866890	2.458	2516200	1969480	32142
GHS0001	4744112	1929650	2.459	3101378	1786192	54803
GHS0004	4224831	1709827	2.471	2154020	1890319	484394
GHS0010	4723753	1915114	2.467	2799219	1919824	87696
GHS0013	4548471	1841757	2.47	2673266	1858481	183966
GHS0017	4858799	1975687	2.459	2752426	2041030	48388
GHS0025	4940434	2012178	2.455	3461350	1745631	7266
GHS0026	4809356	1956672	2.458	3275800	1745114	31239
GHS0027	4740159	1925521	2.462	3399626	1633027	46158
HXR0014	4707226	1909615	2.465	3237605	1689618	43177
HXR0022	4649005	1885056	2.466	3179415	1677323	76308
HXR0044	4731846	1924468	2.459	3449040	1603637	29380
HXR0045	4724387	1919715	2.461	3356530	1643786	40517
HXR0047	4774168	1940808	2.46	3414674	1650151	24005
HXR0126	4706621	1912074	2.462	3324957	1646869	50562
HXR0127	4837470	1967163	2.459	3555647	1624493	10981
HXR0138	4699357	1906388	2.465	3306475	1649635	47496
HXR0147	4785941	1944092	2.462	3437885	1646074	24510
HXR0157	4587457	1859423	2.467	3072226	1687327	97370
HXR0170	4682856	1900508	2.464	3306806	1638279	55143
HXR0176	4746635	1929385	2.46	3416500	1629760	29505
HXR0186	4690923	1904642	2.463	3279843	1657861	53991
HXR0370	4797141	1950052	2.46	3432493	1657350	19079
HXR0711	4814526	1957390	2.46	3514874	1628521	14772
HXR0908	4750305	1929275	2.462	3386930	1646325	33491
HXR097	4745697	1925699	2.464	3274562	1698417	34587
HXR0981	4797132	1946015	2.465	3461279	1640934	25599
HXR1166	4813587	1956855	2.46	3381110	1694666	19770
HXR1167	4800868	1950484	2.461	3263740	1743806	28313
HXR1188	4786503	1949039	2.456	3463664	1635939	24295
HXR1321	4707738	1901983	2.475	3254799	1677461	91669
HXR1630	4804837	1949751	2.464	3517192	1618698	15649
HXR171	4852817	1974535	2.458	3283786	1771783	15439
HXR1750	4682805	1896889	2.469	3021336	1779179	76604
HXR2752	4700454	1903400	2.47	3156036	1723909	60714
HXR2781	4803429	1956311	2.455	3422874	1668433	14666
HXR3030	4805300	1953801	2.459	3179613	1789744	22461
HXR3386	4793660	1948490	2.46	3407568	1667291	23739
HXR3388	4783978	1943524	2.461	3081354	1823074	26120
IMCG01	3665203	1472249	2.49	1554236	1791608	1383378
IMCG02	2288096	939987	2.434	624679	1301702	3410130
IMCG04	3398897	1357096	2.505	1148489	1803752	2175578
IMCG05	1760586	717809	2.453	348961	1064717	4827797
IMCG051	2752376	1094892	2.514	776810	1535229	3077657
IMCG10	4104033	1673747	2.452	2138786	1819497	515779
IMCG101	1977399	795493	2.486	458298	1157297	4169871
IMCG11	3502247	1417191	2.471	1820064	1549687	1217807
IMCG111	4292216	1743803	2.461	2635813	1700103	259809
IMCG12	4370129	1773584	2.464	2200781	1971466	341705
IMCG121	2030459	818514	2.481	471363	1188805	3867581
IMCG13	3784290	1519187	2.491	1759305	1772086	903840
IMCG14	4078266	1651810	2.469	2214732	1757672	473333
IMCG141	2519185	1020517	2.469	674552	1432575	3383217
IMCG15	3593329	1448565	2.481	1520236	1760829	1089893
IMCG19	3037349	1215380	2.499	991867	1630431	2486240
IMCG21	3049040	1217633	2.504	929993	1668340	1992930
IMCG213	2447982	985647	2.484	601969	1415830	3656287
IMCG25	3295914	1324699	2.488	1351153	1634730	1475115
IMCG27	2708139	1100209	2.461	829070	1489639	2771456
IMCG28	2701882	1060098	2.549	917000	1422490	2672987
IMCG284	2979143	1199292	2.484	973303	1602566	2464420
IMCG29	3183594	1295829	2.457	1192593	1643415	1765113
IMCG293	3141090	1263216	2.487	1042746	1680780	2392795
IMCG30	2965523	1208470	2.454	1051509	1561242	2161232
IMCG31	3824253	1555857	2.458	1873560	1753275	892758
IMCG311	1964636	769465	2.553	574965	1079568	4367256
IMCG313	3887200	1579170	2.462	1850000	1808185	1024549
IMCG33	3943823	1595554	2.472	2080719	1729329	616873
IMCG35	2323411	930568	2.497	593167	1330406	3374041
IMCG36	1963278	805482	2.437	374026	1197367	4367294
IMCG37	1802025	689202	2.615	373003	1059112	5124073
IMCG39	2286303	907292	2.52	602621	1295487	3344074
IMCG43	1548317	639237	2.422	290370	948592	5138864
IMCG47	2856397	1140882	2.504	841701	1577789	2537154
IMCG55	4265119	1726278	2.471	2520541	1735428	307276
IMCG710	3316472	1321069	2.51	1414463	1611539	1515295
IRWG01	4542648	1841177	2.467	2850381	1766722	82531
IRWG04	4596021	1868311	2.46	2856502	1803915	9354
IRWG06	4519506	1833045	2.466	2804883	1773834	71197

Variants rate details

Chromosome	Length	Variants	'ariants rate
NC_030808.1	157,403,528	859,183	183
NC_030809.1	136,510,947	692,713	197
NC_030810.1	120,038,259	614,182	195
NC_030811.1	120,734,966	651,557	185
NC_030812.1	119,020,588	613,281	194
NC_030813.1	117,642,375	667,668	176
NC_030814.1	108,433,636	552,138	196
NC_030815.1	112,672,867	589,473	191
NC_030816.1	91,568,626	470,259	194
NC_030817.1	101,087,560	520,544	194
NC_030818.1	106,225,002	518,441	204
NC_030819.1	87,277,232	494,126	176
NC_030820.1	83,034,183	381,712	217
NC_030821.1	94,672,733	498,882	189
NC_030822.1	81,904,557	457,195	179
NC_030823.1	79,370,172	400,298	198
NC_030824.1	71,137,785	375,534	189
NC_030825.1	67,275,902	324,020	207
NC_030826.1	62,516,450	275,063	227
NC_030827.1	71,784,255	403,218	178
NC_030828.1	69,425,955	365,269	190
NC_030829.1	60,283,066	275,428	218
NC_030830.1	48,866,549	280,335	174
NC_030831.1	62,310,066	314,334	198
NC_030832.1	42,858,509	195,942	218
NC_030833.1	51,421,553	283,460	181
NC_030834.1	44,709,034	240,694	185
NC_030835.1	44,672,302	267,592	166
NC_030836.1	51,332,696	279,336	183
W_017189516.	66,011,198	133,608	494
W_017189517.	49,932,331	74,439	670
Total	2,582,134,882	#######	197

IRWG26	4555609	1852561	2.459	2481608	1963281	10100
IRWG28	4208901	1709979	2.461	2247604	1835638	161872
IRWG29	4235301	1724238	2.456	1422833	2268353	249869
IRWG30	4582458	1863247	2.459	2829455	1808125	9002
IRWG31	4551620	1849249	2.461	2345357	2027756	10698
IRWG33	4314618	1748795	2.467	1713131	2175141	153190
IRWG34	4547623	1847524	2.461	2201841	2096653	11315
IRWG35	4364188	1773509	2.461	1855091	2141303	118266
IRWG36	4275071	1738165	2.46	1991756	2010740	133190
IRWG38	4558273	1854286	2.458	2646659	1882950	12249
IRWG40	4561493	1854091	2.46	2486242	1964671	10062
IRWG41	4140088	1682503	2.461	1519715	2151438	298446
IRWG42	4271602	1735043	2.462	1972433	2017106	138548
IRWG43	4385973	1780967	2.463	2155906	2005517	68339
IRWG44	4534733	1842463	2.461	2476038	1950579	13473
IRWG45	4549033	1850680	2.458	2338155	2030779	10851
IRWG47	4567203	1857772	2.458	2348501	2038237	12632
IRWG48	4558729	1852981	2.46	2740580	1835565	17612
JTY0001	4716406	1918437	2.458	3199843	1717500	44181
JTY0012	4790504	1947037	2.46	3191047	1773247	22187
JTY0022	4820776	1961206	2.458	3135348	1823317	28460
JTY0034	4799353	1951201	2.46	3329692	1710431	21065
JTY0038	4637031	1881566	2.464	2610453	1954072	109755
JTY0042	4734210	1924772	2.46	2900456	1879263	54319
JTY0045	4784910	1943188	2.462	2881812	1923143	40419
JTY0050	4839508	1966962	2.46	3336012	1735229	16151
JTY0062	4834459	1968386	2.456	3231399	1785723	26727
JTY0076	4700786	1911536	2.459	2840436	1885943	64273
KOG06	4758291	1934079	2.46	2475766	2108302	23393
KOG07	4738870	1924353	2.463	2958147	1852538	17891
KOG08	4751755	1933669	2.457	3103308	1791058	17460
KOG09	4732128	1925127	2.458	3139363	1758946	23104
KOG10	4817670	1957979	2.461	2442139	2166755	21375
KOG11	4817408	1958961	2.459	2508939	2133715	14449
KOG12	4786908	1948262	2.457	2888072	1923549	18136
KOG13	4745564	1930083	2.459	2746933	1964357	18256
KOG14	4764570	1934533	2.463	2741093	1979005	23541
KOG15	4777653	1941916	2.46	2658157	2030706	21666
KOG16	4709410	1912580	2.462	2733886	1944052	32976
KOG17	4797522	1948302	2.462	2522572	2111626	19429
LNC18	3434664	1396245	2.46	1234377	1798266	1539714
LNC21	3275129	1327134	2.468	1091213	1755525	1895583
LNC22	2158778	871105	2.478	480607	1274638	3894873
LNC24	3984894	1608438	2.477	1787632	1902850	754569
LNC31	2747995	1088785	2.524	771338	1532721	2985587
LNC33	4019888	1622353	2.478	1826325	1907958	827984
LNC34	2558267	1014874	2.521	594699	1489221	3529334
LNC35	2248355	906212	2.481	464295	1345136	4190303
LNC36	1893940	751498	2.52	325336	1160051	5155657
LNC37	3625684	1461432	2.481	1365888	1860614	1368749
LNC38	3143969	1269574	2.476	749111	1832216	2183372
LNC39	2611079	1046471	2.495	590516	1533517	3496411
LNC47	3940464	1597660	2.466	1787264	1875430	819542
LNC48	1903770	748854	2.542	380512	1136056	4587176
LNC52	3838749	1568098	2.448	1556071	1925388	1140955
LNC54	3610768	1468331	2.459	1433303	1822898	1307137
LNC56	3827018	1552754	2.465	1568246	1905763	991348
MANG0027	4582766	1860913	2.463	3098785	1672447	2028
MANG0028	4625256	1879124	2.461	3187576	1658402	2506
MANG0029	4621407	1877745	2.461	2932510	1783321	2447
MANG0030	4592110	1868085	2.458	3100031	1680082	3543
MANG0031	4578920	1861705	2.46	3018215	1711205	10096
MANG0033	4543865	1843396	2.465	3032111	1677575	10777
MTG0001	4535595	1846177	2.457	3200444	1590664	39284
MTG0002	4456174	1814047	2.456	3075661	1597280	76354
MTG0003	4659277	1896810	2.456	2596079	1980004	13664
MTG0005	4581905	1864881	2.457	3326290	1560248	16285
MTG0006	4624908	1880555	2.459	3407349	1549057	14458
MTG0007	4525517	1841363	2.458	3224568	1571156	10813
MTG0009	4611562	1878143	2.455	2560021	1964842	28250
MTG0010	4464875	1816356	2.458	3113995	1583618	45784
MTG0012	4514676	1836212	2.459	2935040	1707924	43708
MTG0013	4558975	1854355	2.459	3197404	1607963	33970
WMG0004	4873414	1983066	2.458	3656126	1600177	3607
WMG0005	4834260	1965391	2.46	3584497	1607577	8452
WMG0006	4862953	1978027	2.458	3242944	1799018	4569
WMG0007	4829670	1964856	2.458	3165590	1814468	12104
WMG0008	4859964	1978611	2.456	3673995	1582290	6294
WMG0009	4740918	1925555	2.462	3437403	1614535	25962
WMG0010	4733810	1919313	2.466	3398843	1627140	34402
WMG0011	4719686	1918904	2.46	3392736	1622927	31983
WMG0012	4821270	1962830	2.456	3606478	1588811	8219
WMG0013	4596650	1863401	2.467	3141519	1659266	82397
WMG0016	4767604	1936221	2.462	2589157	2057334	26568
WMG0017	4766888	1935541	2.463	3404999	1648715	25628
ZANG0374	4651480	1891010	2.46	2889016	1826737	7541
ZANG0388	4606987	1875985	2.456	2846172	1818400	14954
ZANG0397	4565134	1855066	2.461	2820810	1799695	27047
BBG97	5002011	2038281	2.454	3530052	1755120	1958
BBG274	5013774	2045020	2.452	3430110	1814342	3775
BBG3101	5050388	2058782	2.453	3480416	1814377	2535
BBG6106	5041750	2054840	2.454	3300100	1898245	3056
BBG7111	5056297	2060884	2.453	3496091	1810545	2277
BBG8111	5043197	2053634	2.456	3446209	1825311	2099
BBG974	5011988	2039363	2.458	3532721	1759315	2210
BBG10101	5027752	2048087	2.455	3112423	1981708	2957
BBG12142	5063532	2063337	2.454	3359123	1883873	4326
BBG1674	5024566	2045899	2.456	3372381	1849042	4168
BBG1790	5060370	2064735	2.451	3261049	1932028	3315
BBG1897	5022681	2045385	2.456	3464472	1801797	1655
BBG26101	5053938	2061724	2.451	3444188	1835737	2801
BBG28106	5032394	2052822	2.451	3425142	1830037	4939
BBG31107	5053298	2058487	2.455	2883535	2114125	2157
BBG3674	5032982	2052122	2.453	3363748	1860678	3274
BBG39133	5048593	2058965	2.452	3551144	1778207	2839
BBG8094	5051140	2058248	2.454	3403118	1853135	2779
BBG8166	5056106	2061424	2.453	3499620	1808955	1989
BBG8266	5032147	2050317	2.454	3448514	1816975	1737
BBG8396	5051364	2057716	2.455	3455500	1826790	2999
BBG8990	4995544	2035088	2.455	3584048	1723292	2792
BBG90100	5054221	2062650	2.45	3440873	1837999	2799
BBG9195	5034501	2051946	2.454	3410679	1837884	2071
BBG93108	4914437	2000158	2.457	3261999	1826298	22007

BBG9590	4992165	2035267	2.453	3587508	1719962	2618
BBG9896	5061646	2063397	2.453	3410865	1857089	2577
BBG99111	5039676	2052509	2.455	3466093	1813046	1865
GRG15	4814203	1955080	2.462	3657113	1556085	7399
GRG16	4898094	1987130	2.465	3686570	1599327	7793
GRG17	4793357	1947851	2.461	3588308	1576450	17269
GRG18	4807527	1950097	2.465	3675180	1541222	18967
GRG43	4856641	1975217	2.459	3708852	1561503	5184
GRG45	4741643	1922984	2.466	3550765	1556931	29580
GRG47	4794290	1941320	2.47	3646246	1544682	38496
GRG75	4830171	1958457	2.466	3619458	1584585	30379
GRG76	4753862	1923015	2.472	2450633	2113122	122240
GRG77	4758180	1929858	2.466	3665968	1511035	17263
Total	1.03E+09	4.16E+08	2.463			

Manuscript Click here to access/download;Table;Supplementary.pdf

Selection signatures in goats reveal a novel deletion mutant underlying cashmere yield and diameter

Content

Supplementary Notes 1 4
1.1 Sample information and sequencing........ 4
1.2 Quality control and reads mapping........ 4
1.3 Variant calling and annotation........ 5
Supplementary Notes 2: **Population structure and phylogenetic analysis** 5
2.1 Geographic distribution mapping 5
2.2 Principal component analysis (PCA)........ 5
2.3 ADMIXTURE software clustering........ 5
2.4 Phylogenetic tree 6
2.5 LD analysis........ 6
Supplementary Notes 3: Gene flows and effective population size 7
3.1 TreeMix analysis 7
3.2 PSMC analysis 7
Supplementary Notes 4: Selective sweep analysis 8
4.1 Detection of selection signatures........ 错误!未定义书签。
4.2 Genome-Wide Selective Sweep for hair color 8
4.3 Genome-Wide Selective Sweep between for cashmere 8
Supplementary Notes 5: Deletion positioning........ 9
Supplementary Figures........ 10
Supplementary Tables 28
Supplementary sequence 45
Supplementary Reference........ 49

Supplementary Figure List

Supplementary Fig. 1 PCA analysis. 10
Supplementary Fig. 2 Results of genetic structure analysis of 13 goat breeds. 11
Supplementary Fig. 3 Results of genetic structure analysis of 10 Chinese goat breeds. 12
Supplementary Fig. 4 Phylogenetic tree of 10 Chinese native goat breeds. 13
Supplementary Fig. 5 Inferred goat tree of mixture events deduced by TreeMix. 14
Supplementary Fig. 6 Analysis of the signatures of positive selection in the genome of cashmere and ordinary goat breeds. 15
Supplementary Fig. 7 Analysis of the signatures of positive selection in the genome of goat breeds with white and black coat color. 16
Supplementary Fig. 8 Analysis of the signatures of positive selection in the genome of goat breeds with white and brown coat color. 17
Supplementary Fig. 9 Ratios and ZFst values around the *ASIP* locus. 18
Supplementary Fig. 10 CNVs at the *KIT* locus. 19
Supplementary Fig. 11 CNVs at the ASIP locus. 20
Supplementary Fig. 12 Bar graph of enriched terms across positively selected genes with GO term enrichment analysis. 20
Supplementary Fig. 13 Visualization of whole genome sequencing data identifies 582 bp deletion in the ancient goat. 21
Supplementary Fig. 14 Visualization of Whole Genome Sequencing Data identifies 582 bp deletion near *LHX2* in the ibex goats. 22
Supplementary Fig. 15 The pattern of SNP genotypes near DEL among 13 goat breeds. 22
Supplementary Fig. 16 The expression of *LHX2*, *JAK2* and *Notch1* in different days (from 45 d to 135 d) of fetal skin. 23
Supplementary Fig. 17 Schematic drawing of the construction of DNA plasmids. 24
Supplementary Fig. 18 The expression of *LHX2* and *AR* in different months of a year. 25
Supplementary Fig. 19 The expression of *JAK2* and *EDA2R* as well as *FGF5* in different months of a year. 26
Supplementary Fig. 20 The PCR identification of deletions in sheep. 27
Supplementary Fig. 21 The expression of *STK2* and *NOTCH1* as well as *TCF7L1* in different months of a year. 28

Supplementary Table List

Supplementary Table 1 Overview of sequencing information of 120 goats 30
Supplementary Table 2 Sequencing information of 116 published goats used in this study... 33
Supplementary Table 3 Sequencing information of Wild goats used in this study 37
Supplementary Table 4 Sequencing platforms for 236 goats of 13 goat breeds. 38
Supplementary Table 5 Published ancient samples used in this study. 39
Supplementary Table 6 The genotype frequencies of the homozygous 582bp deletion (-/-) at *LHX2* locus for 13 goat breeds, obtained from whole genome sequencing. 40
Supplementary Table 7 The genotype frequencies of the homozygous 504bp deletion (-/-) at FGF5 locus for 13 goat breeds, obtained from whole genome sequencing. 41
Supplementary Table 8 The frequencies of the 582 bp deletion near *LHX2* with PCR amplification. 42
Supplementary Table 9 The frequencies of the 504 bp deletion near *FGF5* with PCR amplification. 43
Supplementary Table 10 Primers used for amplification of deletion seences 44

Supplementary Notes 1

1.1 Sample information and sequencing

All 120 goats of 8 breeds were used in this study, which includes 30 Chaidamu Cashmere Goats (CDMC) from Chaidamu City of Qinghai Province; 11 Ujumqin Cashmere goats (UC) from Ujimqin Banner of Inner Mongolia Province; 8 Guizhou Black goats (GZB) from Guiyang City of Guizhou Province; 11 Yunnan Black Bone Goats (YNBB) from Lanping County of Yunnan Province; 10 Matou Goats (MT) from Shangqiu City of Henan Province; 10 Jining Grey Goats(JNG) from Jining City of Shandong Province; 10 Jintang black goat(JTB) from Jintang County of Sichuan Province; 12 Chengdu brown goat(CDB) from Chengdu City of Sichuan Province.

Firstly, ear tissues or blood of goats were collected on-site and stored in alcohol sampling tubes or blood collection tubes; then genomic DNA was extracted by commercial kits, and samples with DNA integrity and concentration > 50ng/ul were used for library construction. Next, the standard circularization step of DNBSEQ was carried out to make DNA nanoballs. Finally, the libraries were sequenced using BGIseq500 with PE100[1].

1.2 Quality control and reads mapping

Quality control of all raw reads was performed using SOAPnuke1.5.0 (https://github.com/BGI-flexlab/SOAPnuke), with the following criteria: quality value ≤20; low-quality bases >30%; and N bases >5%. Clean reads were then mapped to the reference genome ARS1 (https://www.ncbi.nlm.nih.gov/genome/10731?genome_assembly_id=281266) using BWA-0.7.12[2] (https://sourceforge.net/projects/bio-bwa/) with default parameters. In the mapping process, a BAM file index was built using SamTools (https://github.com/samtools/samtools/releases/), followed by the bam file being sorted and removed for duplicated reads with picard-tools-1.105 (https://github.com/broadinstitute/picard/releases).

1.3 Variant calling and annotation

After mapping, the "HaplotypeCaller", "CombineGVCFs" and "GenotypeGVCFs" in GATK4 were used to detect SNPs and indels with default parameters. The output VCF File was then screened for SNPs using "SelectVariants function" of GATK4. We further reserved the SNPs using "VariantFiltration" of GATK4 using the following criteria: QD < 2.0; FS > 60.0; MQ < 40.0; and ReadPosRankSum < -8.0. Next, we used VCFtools to obtain high-quality SNPs combined with population information. SNPs that met at least one of the following standards were excluded: (i) minor allele frequency (MAF) <0.05; (ii) SNP call rate <90%; and (iii) P < 0.000001 for the Hardy–Weinberg equilibrium. Only the loci of two alleles were retained for the subsequent analyses. Finally, ~13 million SNPs were remained for further analysis.

Supplementary Notes 2: Population structure and phylogenetic analysis

2.1 Geographic distribution mapping

First, we obtained the longitude and dimension data of 10 goat sampling sites through Google map; then used package "mapdata" and "ggrepel in R for mapping; finally, the Photoshop software was used for organization and optimization.

2.2 Principal component analysis (PCA)

The PLINK was used for principle component analysis (PCA) with 120 goats based on the filtered SNPs using function "--vcf vcf --out pca –pca --chr-set 29 --allow-extra-chr". The contribution of PCA1 and PCA2 was 15.94% and 10.3% respectively. The PCA further reveals that the difference between North China (cashmere goats, JNG and MT) and South China (JTB, CDB, YNBB and GZB) is greater than that between cashmere goats and ordinary goats (**Supplementary Fig. 1**).

2.3 Population admixture

Population structure was inferred using a Bayesian-based approach via the software package Admixture_linux-1.23 (https://dalexander.github.io/admixture/index.html); then, the cross validation was performed to choose the optimal *k* value. The analysis

process includes the following three steps:

(1) Converting VCF format to PLINK format. The Linux command line was “vcftools --vcf vcf --plink --out goat.plink”;

(2) Using PLINK for further filtering with Linux command line “plink --noweb --file plink --geno 0.05 --maf 0.05 --hwe 0.0001 --chr-set 29 --make-bed --out QC”; after this step we get the corresponding bed file.

(3) Using ADMIXTURE v1.23 for population structure analysis with k values ranged from 3 to 7. Five-fold cross-validation was used to evaluate the fitness. For each k, we ran the ADMIXTURE for 20 times and calculated the corresponding cross-validation errors with the Linux command line “admixture --cv QC.bed $k | tee log${k}.out”; then the results were plotted using R.

2.4 Phylogenetic tree

The VCF files were converted into a matrix using VCF2Dis-1.09 (https://github.com/BGI-shenzhen/VCF2Dis), and tree files were formed using PHYLIPNEW-3.69.650 (https://evolution.genetics.washington.edu/phylip.html). Then a phylogenetic tree based on genetic distance was constructed via the neighbor-joining method by iTOL[3] (http://itol.embl.de). The Linux command line of converting to a matrix is "VCF2Dis -InPut vcf -OutPut p_dis.mat"; the Linux command line of constructing a phylogenetic tree is "PHYLIPNEW-3.69.650/bin/fneighbor -datafile p_dis.mat -outfile goat.tree.txt -matrixtype s -treetype n -outtreefile goat.n.tree.tre”. The neighbor-joining (NJ) tree revealed strong clustering of the Chinese native goat into three genetic groups that presented a low level of genetic differentiation, which recapitulated the finding in the PCA (Supplementary Fig. 4).

2.5 LD decay

PopLDdecay (https://github.com/BGI-shenzhen/PopLDdecay) was used to calculate the r^2 of linkage disequilibrium (LD) with command "pLDdecay -InVCF vcf -MaxDist 300 -OutStat LDdecay".

Supplementary Notes 3: Gene flows and effective population size

3.1 Gene flow

A population-level admixture analysis was carried out with TreeMix (version 1.12) program (Pickrell and Pritchard 2012). It uses genome-wide SNP sites to infer the Maximum Likelihood (ML) tree for the 12 goat breeds (236 individuals) and an outgroup (Bezoars), with the command '-i input -bootstrap -k 500 -root IRW -o output'. The covariance matrix of the ML tree was estimated to reflect the correspondence between the ML tree and the SNP data and to identify pairs of populations that showed poor fits in the ML tree. When the ML tree did not fully describe the data, an admixture analysis was performed by allowing migration events. The poor-fit populations were regarded as candidates for adding potential migration edges. Migration events are gradually added to the ML trees until the model can explain 98% of the variation in breeds. The command was '-i input -bootstrap -k 500 -m migration events -o output'. The maximum-likelihood (ML) tree without migration events inferred from the TreeMix analysis divided the 236 goats into three clusters, which is similar to the population structuring patterns identified from the phylogenetic tree, PCA and genetic structure analysis (**Supplementary Fig. 5**). Migration events were detected among the three clusters when potential migration edges were added to the ML tree (**Supplementary Fig. 5**).

3.2 Effective population size

PSMC software was used to infer historical dynamics of effective population size and the divergence timing of goats based on seven samples from four lineages with a high sequencing depth[4]. The whole-genome diploid consensus sequences for each sample were generated by SAMtools and BCFtools with the parameter C50. Sites with sequencing depths < 10 and > 100 (vcfutils.pl vcf2fq -d 10 -D 100) were removed to reduce the probability of false positives. The PSMC parameter (psmc -N25 -t15 -r5 -p "4+25*2+4+6") was used to infer the historical effective population size, where the parameters of generation time and mutation rate were set to 2.0 and $2.5e^{-8}$,

respectively.

Supplementary Notes 4: Selective sweep analysis

Fst and θπ were performed to evaluate fixation and differentiation. High-quality SNPs were chosen to calculate Fst using VCFtools based on sliding 150kb windows with a 10kb step length. Fst values were then standardized as ZFst by calculating Z-scores according to the formula $x^*=(\bar{x}-x)/\sigma$. We also calculated the θ_π ratios and $\log_2$-transformed, namely $Log_2(\theta_\pi$ Ratios). We annotated genes functions for those windows with larger ZFst and $Log_2(\theta_\pi$ Ratios) values (top 1%) [5,6]. Gene ontology and KEGG analyses were performed using Metascape[7] and compared to the human genome as background.

4.1 Genome-Wide Selective Sweep between cashmere and ordinary goat

We performed selective sweep analysis with genome sequences of 84 cashmere goats (IMC, CDMC, LNC) and 58 ordinary goats (YNBB, GZB, JTB and GZB) We choose those windows with top 1% of statistic (ZFst>4.042 and $\log_2(\theta_\pi$ Ratios)> 1.348) for candidate windows. Finally, we identified 141 candidate genes in and around these windows (500kb from both sides) (Supplementary Table s2 and Supplementary Fig. 6).

4.2 Genome-Wide Selective Sweep for hair color

First, we scanned for selection signatures for the white coat group (CDMC, UC, IMC, LNC) and the black coat group (YNBB, GZB). We identified signature regions harboring *KIT*, *KITG* and *IRF4*(Supplementary Fig.7 and Supplementary Table s4) and 100 kb copy number variants (CNVs) downstream the *KIT* (chr6: 70,859,258-70,959,918) (Supplementary Fig. 10) , which is consistent with the previously report [8]. We next searched for the selection signature in the white coat group (LNC, IMC, CDMC and UC) and the brown coat group (CDM),We also identified a striking signature harboring *ASIP*, *AHCY* and *ITCH* (Supplementary Fig. 8-9 and Supplementary Table s3) and ~154 kb CNV partly overlapping with *ASIP* (chr13:63,334,008-63,380,258) (Supplementary Fig. 11), which is consistent

with the previously reported [8].

Supplementary Notes 5: Deletion of breakpoints of *LHX2* and *FGF5*

To detect the heterozygous genotype of goats more accurately, we designed PCR primers to detect the deletions in *LHX2* and *FGF5* (Supplementary Table 8 and Supplementary Table 9). Also, we used the same primers to detect the deletions in several sheep breeds (Hu sheep, Ujumqin sheep, and Australian white sheep) (Supplementary Fig. 21).

Supplementary Figures

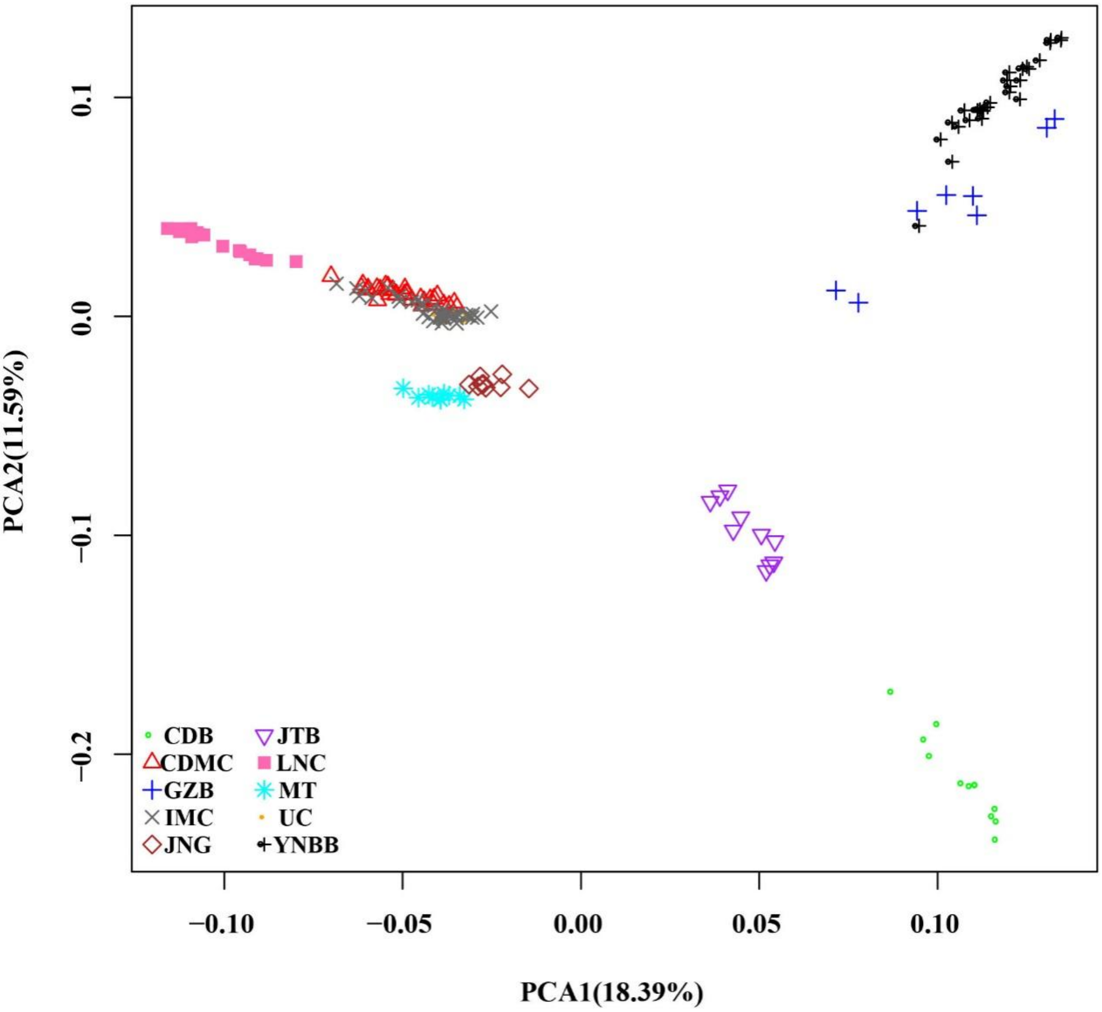

Supplementary Fig. 1 PCA analysis.

Principal components 1 and 2 of 10 local native goat breeds. The horizontal axis represents principal component 1 (PCA1), and the vertical axis represents principal component 2 (PCA2). Four cashmere goat breeds (CDMC, IMC, UC, LNC) were gathered; JNG and MTG breeds are close to the cashmere goats; The JTB, CDB, YNBB and GZB in Southwest China were all far from the cashmere goats and were divided into two small groups.

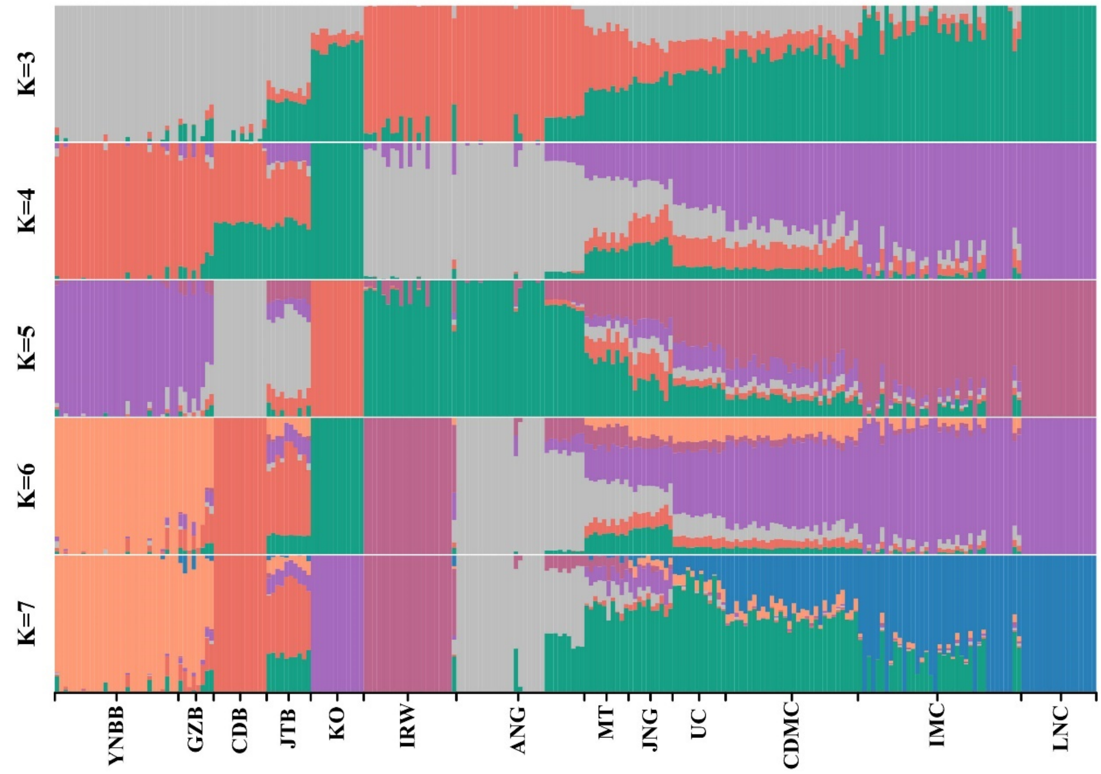

Supplementary Fig. 2 Results of genetic structure analysis of 13 goat breeds.

The length of each colored segment represents the proportion of the individual's genome inferred from K=3-7 ancestral populations. For the abbreviations of the individuals see supplementary table 3-5.

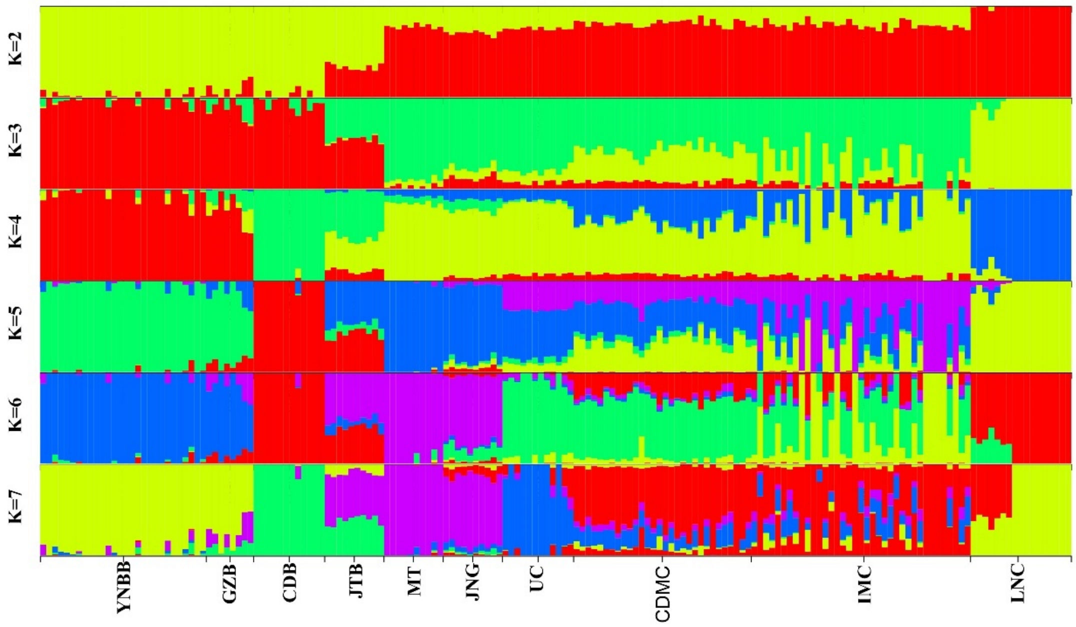

Supplementary Fig. 3 Results of genetic structure analysis of 10 Chinese goat breeds. The length of each colored segment represents the proportion of the individual's genome inferred from K=2-7 ancestral populations. For the abbreviations of the individuals see supplementary table 3-4.

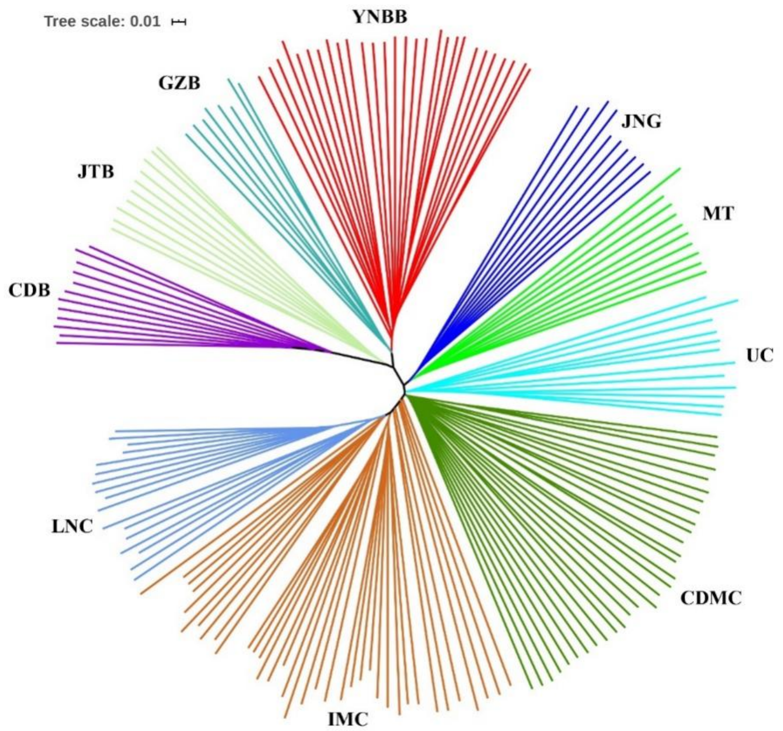

Supplementary Fig. 4 Phylogenetic tree of 10 Chinese native goat breeds.
Each color represents a breed, and each line represents an individual. Ten local goat breeds were clustered into two groups: four goat breeds in southwest China (CDB, JTB, GZB and YNBB) clustered for one group; LNC, IMC, CDMC, UC, MT and JNG gathered for another, JNG and MTG have clustered separately close to the cashmere goat subgroup.

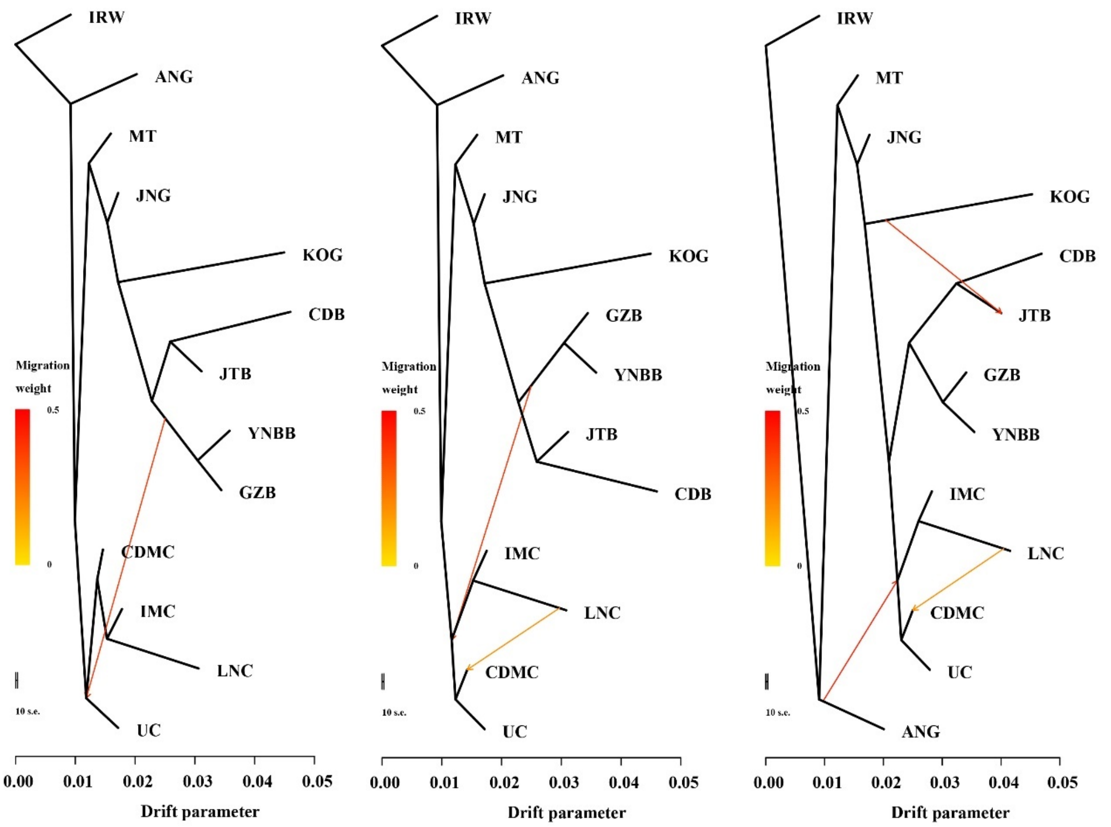

Supplementary Fig. 5 Inferred goat tree of mixture events deduced by TreeMix.

IRW samples were assigned as "outgroup" to root the tree. The graph inferred for the goat populations, allowing one, two and three migration events. Migration arrows are colored according to their weight. Horizontal branch lengths are proportional to the amount of genetic drift that has occurred on each branch. The scale bar shows ten times the average standard error of the entries in the sample covariance matrix.

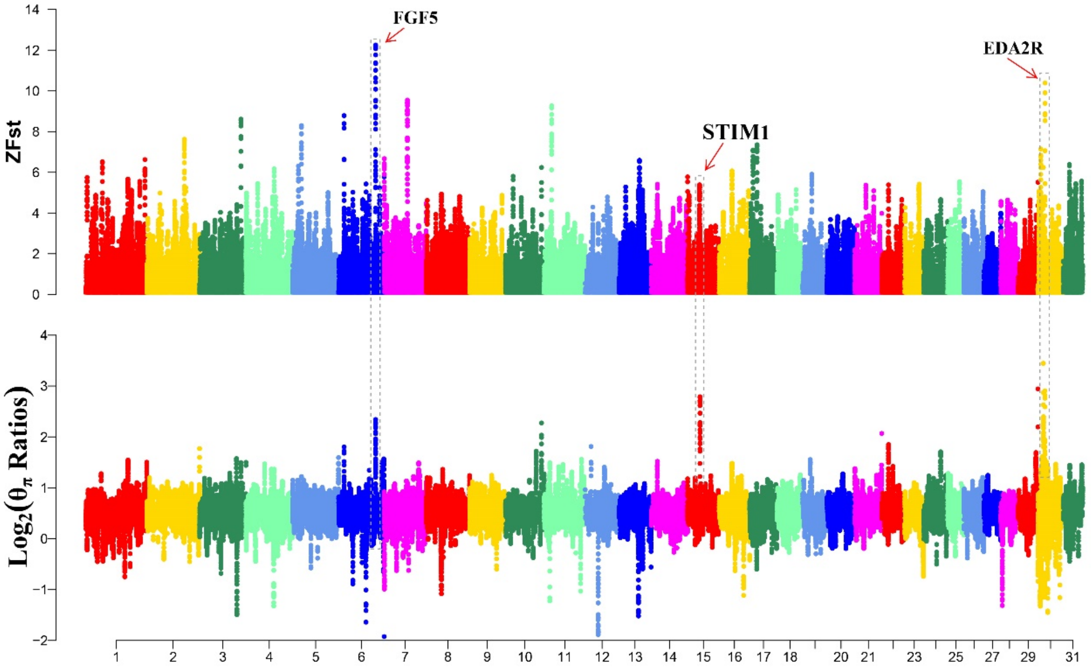

Supplementary Fig. 6 Analysis of the signatures of positive selection in the genome of cashmere and ordinary goat breeds.

Manhattan plot of the genome-wide distribution of pairwise ZFst and $\log_2(\theta_\pi$ Ratios) between cashmere goats (CDMC, IMC, LNC) and ordinary goats (YNBB, GZB, JTB, CDB) using a 150 kb window size and a 10 kb step size. The key candidate genes *FGF5, STIM1, KIT* related to cashmere were annotated at the significant signal. Red arrows and names indicate the top three candidate genes.

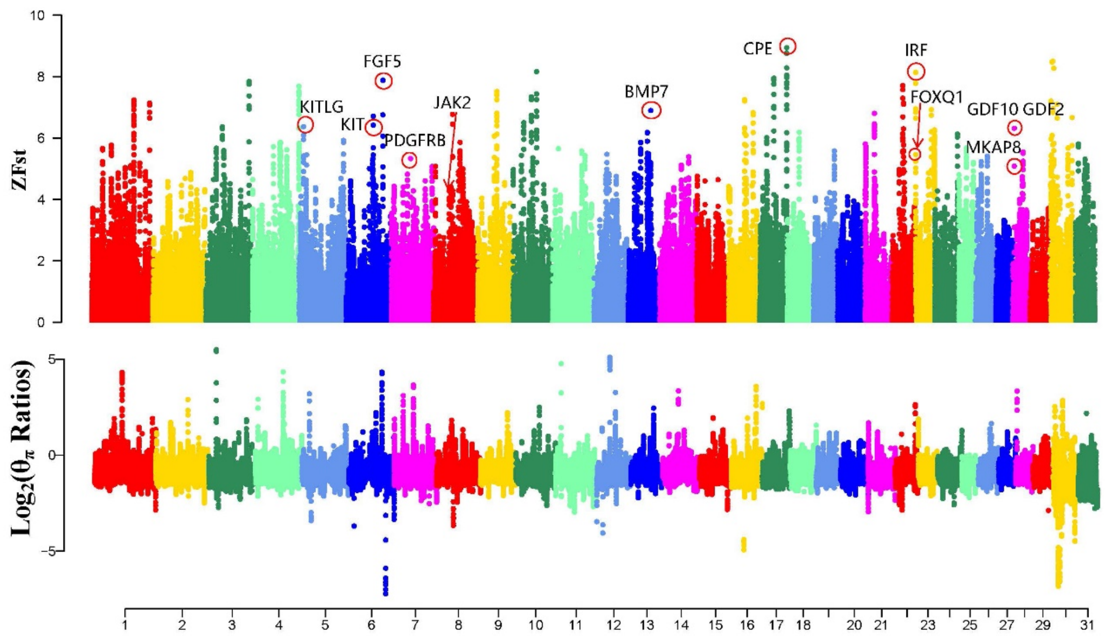

Supplementary Fig. 7 Analysis of the signatures of positive selection in the genome of goat breeds with white and black coat color.

Manhattan plot of the genome-wide distribution of pairwise ZFst and $\log_2(\theta_\pi$ Ratios) between white coat color goats (UC, CDMC, IMC, LNC) and black coat color goats (YNBB, GZB) using a 150 kb window size and a 10 kb step size. The key candidate genes *KIT*, *KITLG*, *FGF5*, *JAK2*, *FOXQ1* related to cashmere were annotated at the significant signal. Red circles and names indicate the top three candidate genes.

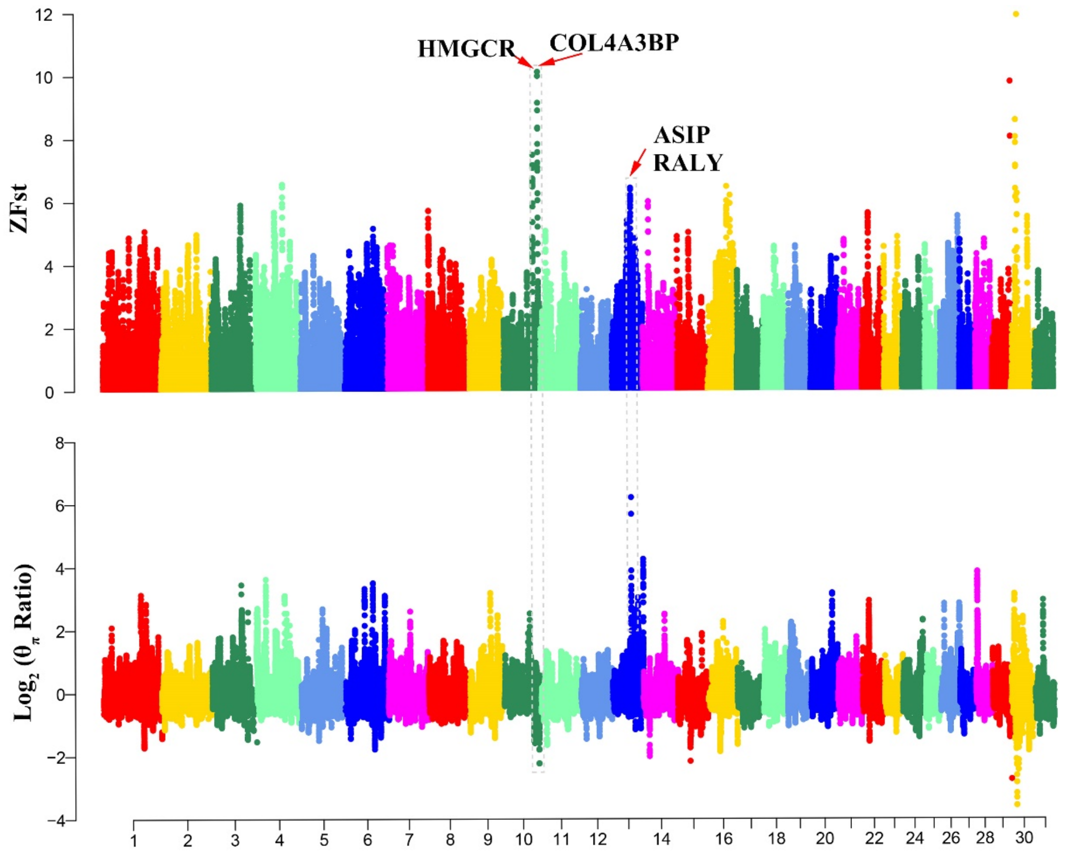

Supplementary Fig. 8 Analysis of the signatures of positive selection in the genome of goat breeds with white and brown coat color.

Manhattan plot of the genome-wide distribution of pairwise ZFst and log2(θ_π Ratios) between white coat color goats (UC, CDMC, IMC, LNC) and brown coat color goats (CDB) using a 150 kb window size and a 10 kb step size. The key candidate genes *ASIP*, *RALY*, *HMGCR*, *COL4A3BP* related to cashmere color were annotated at the significant signal. Red arrows and names indicate the top three candidate genes.

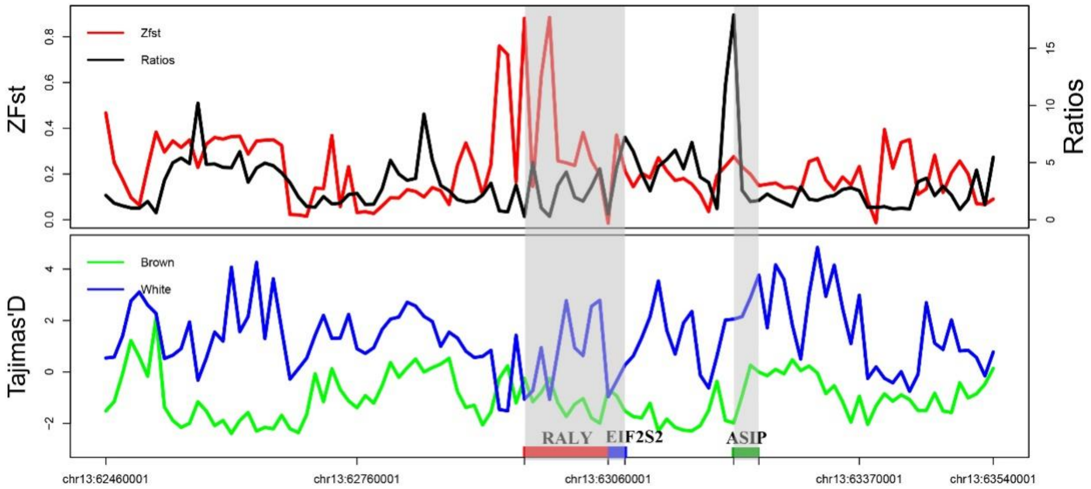

Supplementary Fig. 9 Ratios and ZFst values around the *ASIP* locus.

The black and red lines represent θπ Ratios and ZFst values, respectively. Tajima's D values around the *RALY*, *EIF2S2*, *ASIP* locus. The green and blue lines represent the brown coat color goats (CDB) and the white coat color goats (CDMC, IMC, UC, LNC).

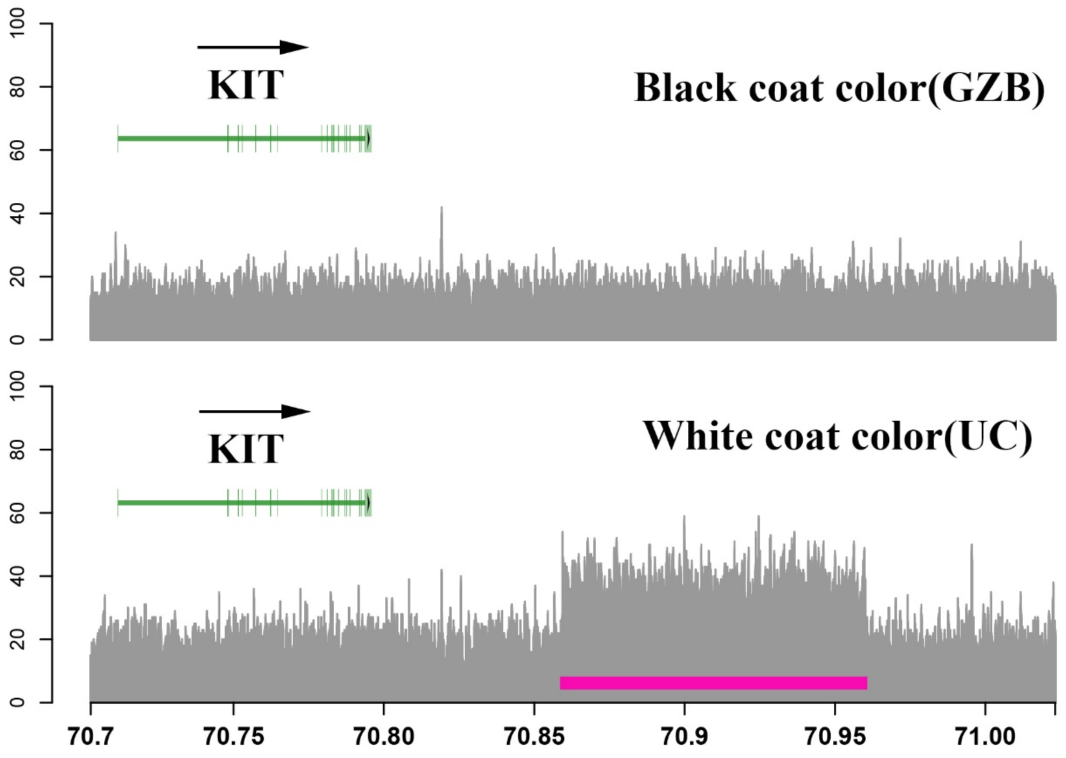

Supplementary Fig. 10 CNVs at the *KIT* locus.

The black coat color goat (GZB) does not show copy number variation (upper panel); however, in the white coat color goat (UC), the coverage plot shows a ~100 kb duplicated CNV (chr6:70859258-70959918) downstream of the *KIT* gene (lower panel).

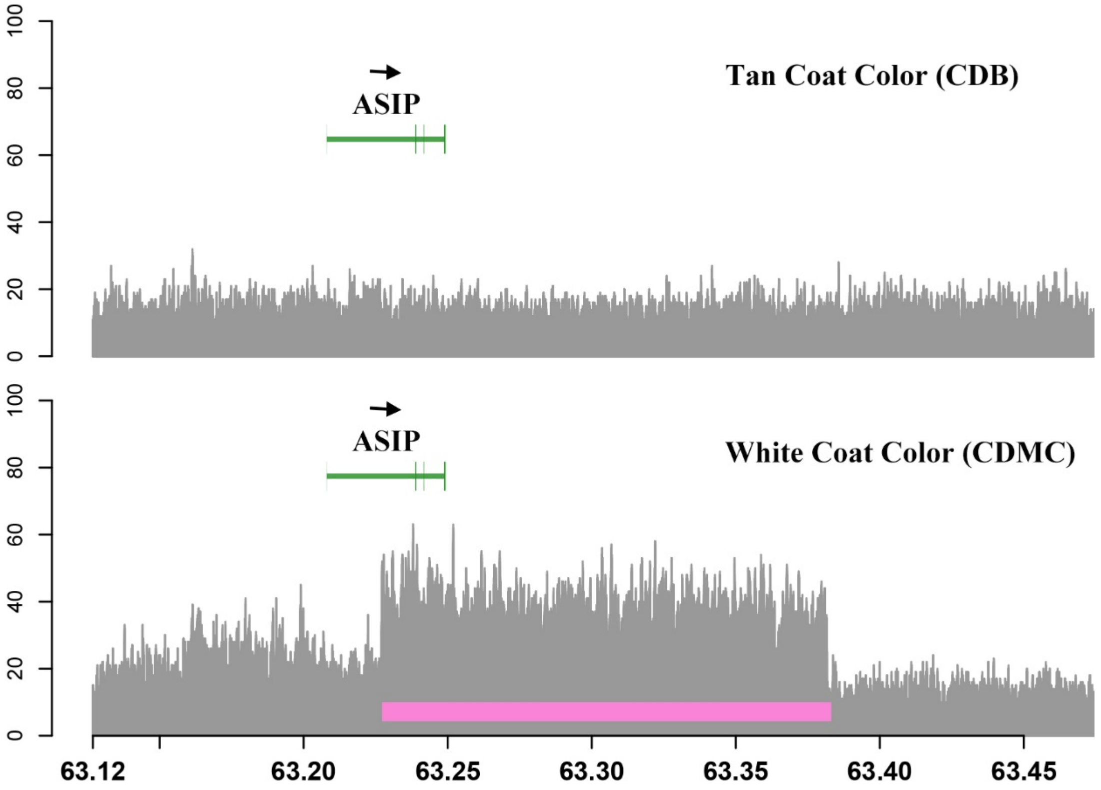

Supplementary Fig. 11 CNVs at the ASIP locus.

The black coat color goat (CDB) does not show any copy number variation. However, in the tan coat color goat (CDMC), the coverage plot shows a duplication of ~154 kb(chr13:63226824-63381501) downstream of the *KIT* gene.

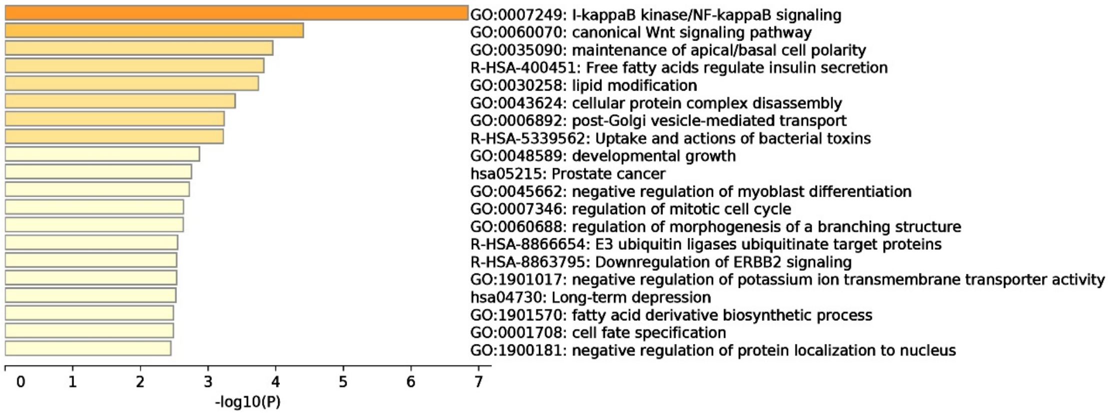

Supplementary Fig. 12 Bar graph of enriched terms across positively selected genes with GO term enrichment analysis.

X-axis represents the p-values.

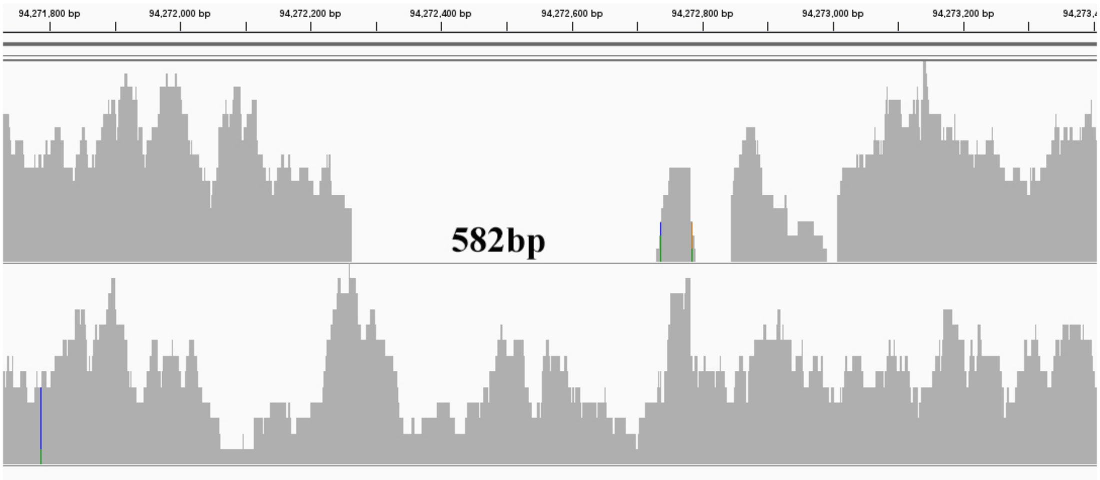

Supplementary Fig. 13 Visualization of whole genome sequencing data identifies 582 bp deletion in the ancient goat.

BAM files were visually inspected using the Integrative Genomics Viewer (IGV).

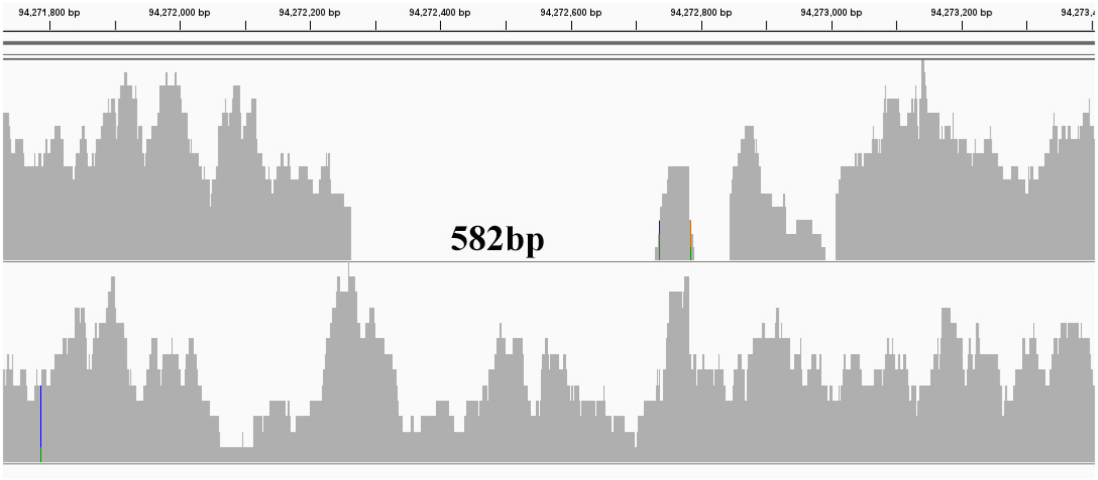

Supplementary Fig. 14 Visualization of Whole Genome Sequencing Data identifies 582 bp deletion near *LHX2* in the ibex goats.
Upper panel represents ibex goat carries 582bp deletion and lower panel not carries.

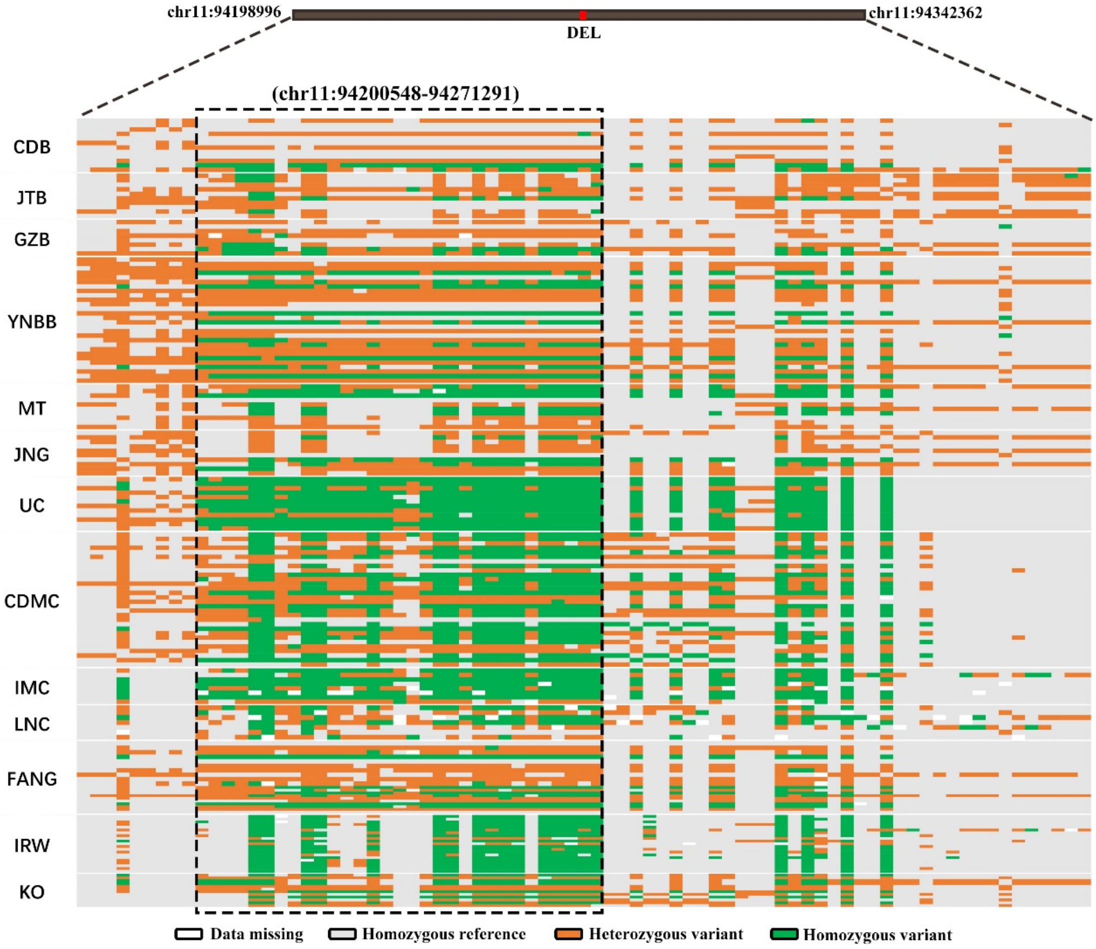

Supplementary Fig. 15 The pattern of SNP genotypes near DEL among 13 goat breeds.

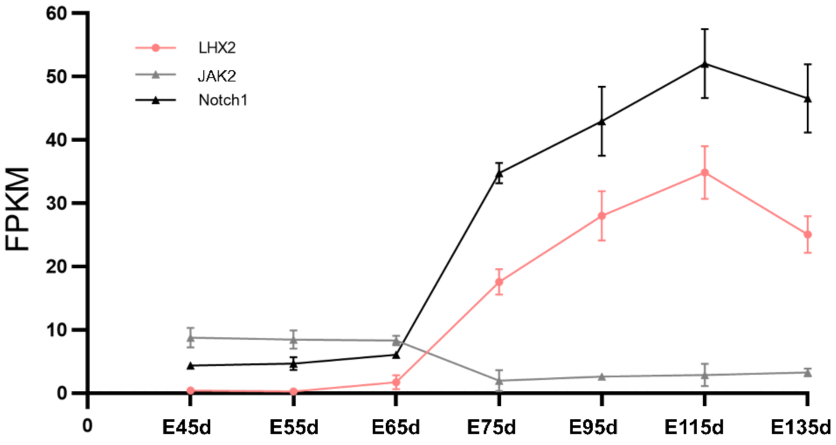

Supplementary Fig. 16 The expression of *LHX2*, *JAK2* and *Notch1* in different days (from 45 d to 135 d) of fetal skin.

E represents days of gestation. The expression data is downloaded from the published data[9].

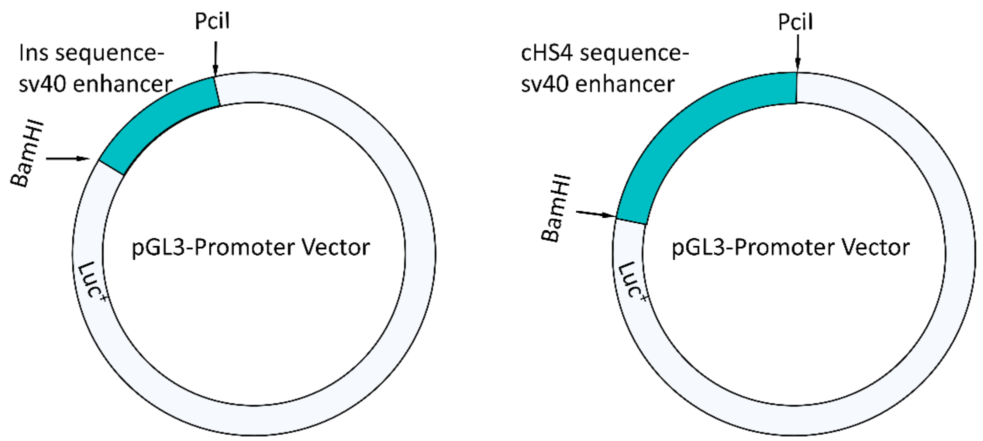

Supplementary Fig. 17 Schematic drawing of the construction of DNA plasmids.
The 796 bp DNA fragment (including 582 bp deletion sequence and some flanking sequence, named Ins and cHS4) and subsequently inserted downstream and upstream of the SV40 promoter in pGL3 plasmid.

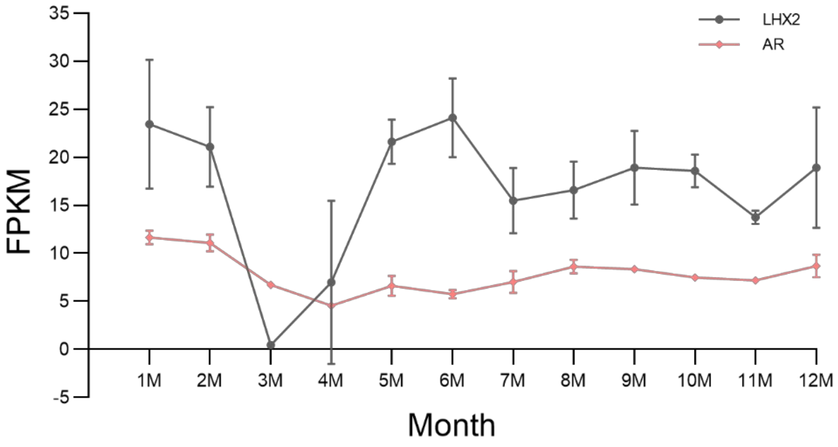

Supplementary Fig. 18 The expression of *LHX2* and *AR* in different months of a year. The expression data is downloaded from the NCBI SRA database[10] (PRJNA470971).

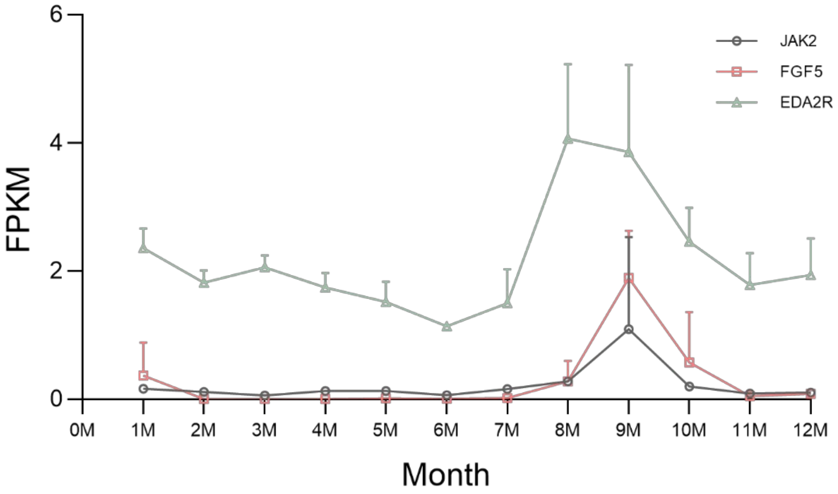

Supplementary Fig. 19 The expression of *JAK2* and *EDA2R* as well as *FGF5* in different months of a year.

The expression data is downloaded from the NCBI SRA database[10] (PRJNA470971).

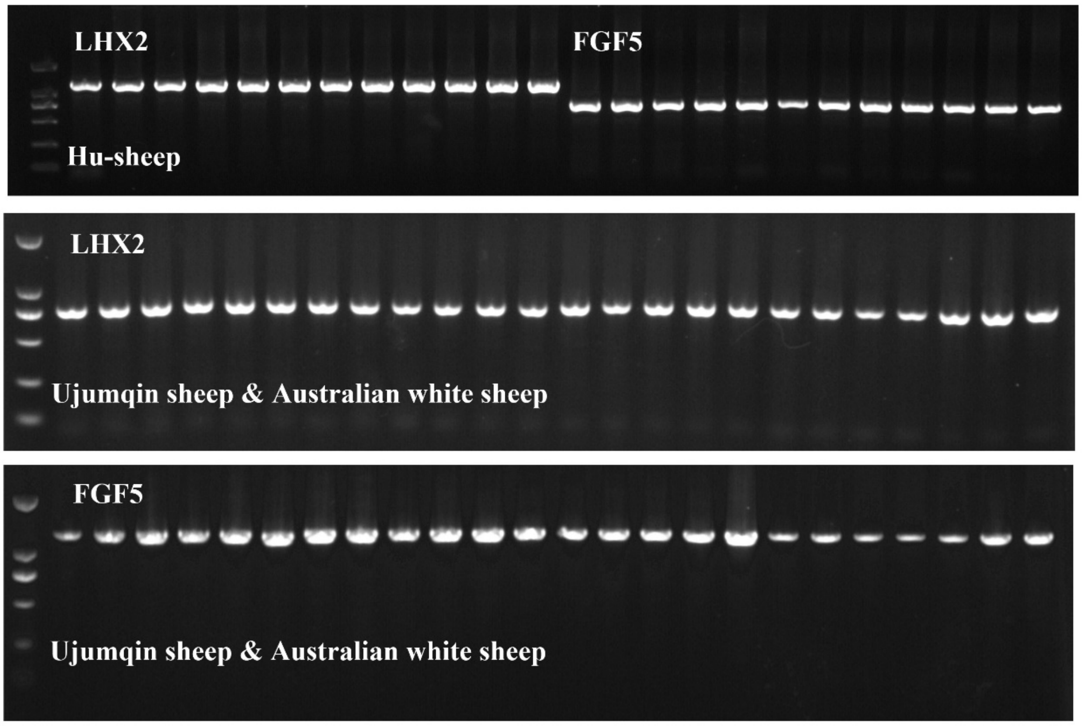

Supplementary Fig. 20 The PCR identification of deletions in sheep.

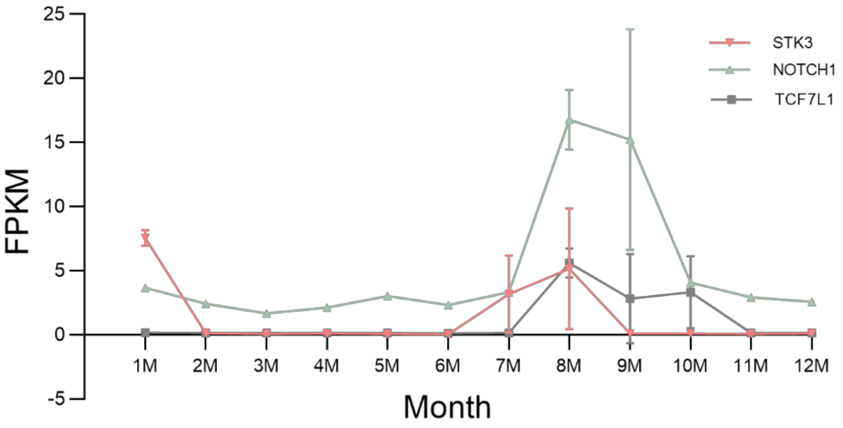

Supplementary Fig. 21 The expression of *STK2* and *NOTCH1* as well as *TCF7L1* in different months of a year.

The expression data is downloaded from the NCBI SRA database[10] (PRJNA470971).

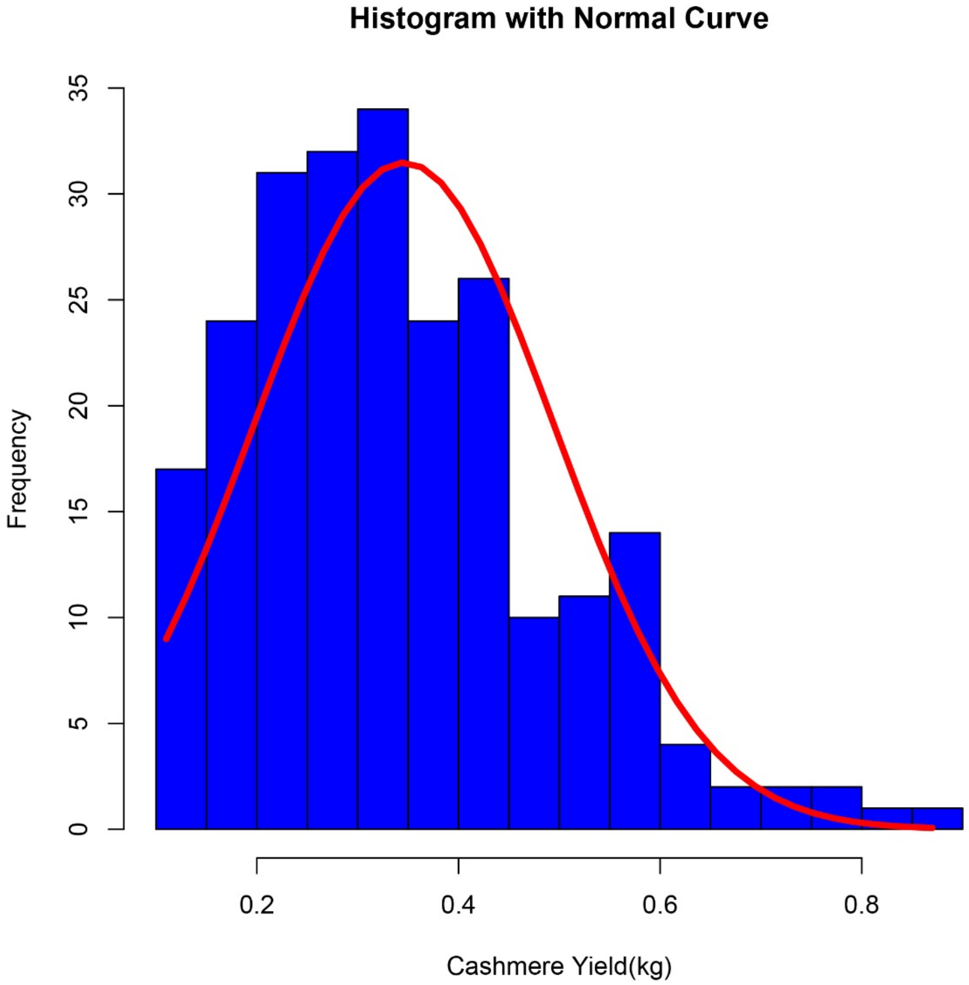

Supplementary Fig. 22 Distribution diagram of cashmere yield data

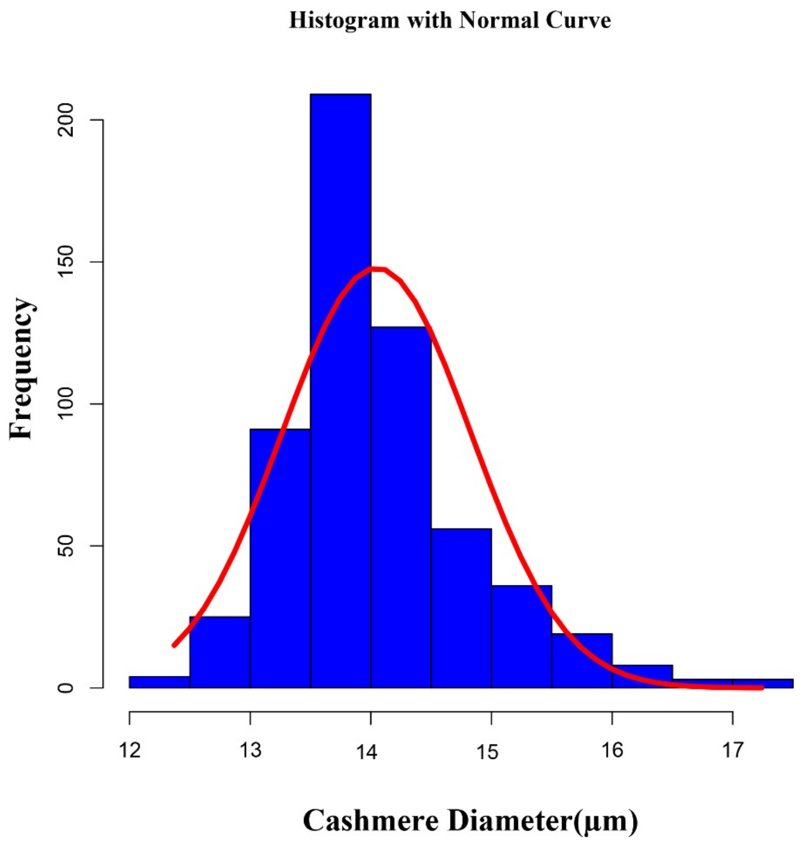

Supplementary Fig. 23 Distribution of cashmere diameter measurements

Supplementary Tables

Supplementary Table 1 Overview of sequencing information of 120 goats

Abbreviation	Sampling location	ID	Size_MB	Depth	Coverage	Mapping_rate(%)
CDMC	Chaidamu, Qinghai	HXR0157	24722.84	8.09	99.65%	99.19
		HXR0014	27535.99	9.33	99.70%	99.21
		HXR0138	28576.42	9.43	99.72%	99.2
		HXR0044	29417.06	10.04	99.71%	99.22
		HXR1167	29859.92	10.18	99.68%	99.36
		HXR0147	31318.85	10.51	99.72%	99.19
		HXR0047	31448.91	10.46	99.73%	99.2
		HXR1188	32735.31	11.07	99.71%	99.28
		HXR1166	32867.20	11.27	99.71%	99.3
		HXR171	34456.79	11.82	99.72%	99.26
		HXR0981	34904.03	11.86	99.69%	99.24
		HXR1321	30607.06	10.28	99.54%	99.17
		HXR1750	26659.97	9.09	99.55%	99.22
		HXR2752	28146.61	9.53	99.60%	99.22
		HXR0908	28744.20	9.94	99.66%	99.27
		HXR1630	33845.47	11.68	99.72%	99.09
		HXR3386	30860.89	10.67	99.65%	99.25
		HXR3388	30735.56	10.6	99.65%	99.27
		HXR0022	26253.44	8.7	99.58%	99.24
		HXR0045	29850.22	10.09	99.63%	99.3
		HXR0126	28236.77	9.4	99.67%	99.27
		HXR0170	28779.79	9.6	99.65%	99.28
		HXR0186	27155.72	9.06	99.64%	99.25
		HXR0370	33842.07	11.2	99.73%	99.18
		HXR0711	34055.78	11.64	99.71%	99.27
		HXR097	30425.80	10.06	99.68%	99.26
		HXR0127	36488.06	12.6	99.72%	99.27
		HXR0176	29880.23	10.19	99.65%	99.28
		HXR2781	35434.27	12.17	99.71%	99.28
		HXR3030	31065.93	10.6	99.68%	99.17
UC	Ujumqin,Inner Mongolia	WMG0010	29808.94	10.22	99.68%	99.22
		WMG0013	25158.27	8.53	99.58%	99.19
		WMG0016	32235.68	10.99	99.61%	99.33
		WMG0017	32123.61	11.03	99.70%	99.23
		WMG0004	45279.43	15.93	99.76%	99.23

		WMG0005	37758.79	13	99.73%	99.25
		WMG0006	43441.20	15.16	99.73%	99.22
		WMG0007	35369.19	12.18	99.73%	99.23
		WMG0008	39552.82	13.77	99.76%	99.24
		WMG0009	30251.41	10.41	99.71%	99.21
		WMG0011	29613.21	10.01	99.70%	99.23
		WMG0012	39691.25	13.55	99.74%	99.24
CDB	Chengdu,Sichuan	CMS0019	28356.30	9.82	99.66%	99.25
		CMS0084	34839.14	12.21	99.69%	99.27
		CMS0177	34653.57	12.04	99.69%	99.4
		CMS0330	32826.68	11.55	99.67%	99.38
		CMS0037	31909.39	10.83	99.69%	99.28
		CMS0088	39151.30	13.21	99.70%	99.26
		CMS0100	28772.64	9.75	99.61%	99.39
		CMS0406	27437.51	9.34	99.63%	99.28
		CMS0026	33172.37	10.83	99.66%	99.3
		CMS0053	31938.17	10.64	99.67%	99.32
		CMS0065	25939.66	8.68	99.58%	99.28
		CMS0431	28635.63	9.36	99.60%	99.33
GZB	Guiyang,Guizhou	GHS0004	16639.80	5.39	98.76%	98.98
		GHS0013	20755.17	6.93	99.46%	98.99
		GHS0010	24577.96	8.2	99.60%	98.56
		GHS0001	26279.23	8.89	99.65%	98.88
		GHS0027	27464.94	9.22	99.68%	99.18
		GHS0017	27865.07	9.4	99.64%	99.1
		GHS0026	29457.34	9.86	99.70%	99.22
		GHS0025	37752.37	13.01	99.75%	99.18
JTB	Jintang,Sichuan	JTY0038	22812.64	7.84	99.56%	99.2
		JTY0076	25960.19	8.93	99.66%	99.23
		JTY0042	26355.43	9.26	99.65%	99.25
		JTY0001	27932.10	9.67	99.62%	99.24
		JTY0045	29104.36	9.92	99.68%	99.21
		JTY0022	30724.89	10.59	99.70%	99.03
		JTY0062	30799.82	10.83	99.71%	99.21
		JTY0012	32126.81	10.9	99.71%	99.1
		JTY0034	32642.38	11.22	99.72%	99.09
		JTY0050	35249.33	11.95	99.75%	99.22
YNBB	Lanping,Yunnan	BBG97	68903.47	24.77	99.68%	99.49
		BBG274	53166.99	19	99.64%	99.54
		BBG3101	67090.35	23.88	99.72%	99.62
		BBG6106	57484.93	20.78	99.69%	99.56
		BBG7111	57943.70	20.91	99.67%	99.53
		BBG8111	57948.10	20.75	99.70%	99.51

		BBG974	56834.47	20.32	99.69%	99.47
		BBG10101	52573.69	18.74	99.69%	99.52
		BBG12142	67912.64	24.3	99.68%	99.61
		BBG1674	55152.12	19.84	99.65%	99.55
		BBG1790	61289.37	21.93	99.69%	99.62
		BBG1897	69885.29	24.88	99.72%	99.54
		BBG26101	55940.49	20.26	99.70%	99.46
		BBG28106	67676.59	24.27	99.68%	99.35
		BBG31107	74517.03	26.94	99.68%	99.54
		BBG3674	58077.22	20.82	99.66%	99.61
		BBG39133	67997.18	24.34	99.71%	99.64
		BBG8094	59123.55	21.11	99.68%	99.52
		BBG8166	59744.89	21.79	99.70%	99.6
		BBG8266	56908.26	20.7	99.70%	99.55
		BBG8396	67991.46	24.94	99.71%	99.53
		BBG8990	59468.12	21.62	99.70%	99.57
		BBG90100	64959.42	23.48	99.69%	99.66
		BBG9195	59900.10	21.55	99.68%	99.54
		BBG93108	64680.66	11.98	99.54%	99.67
		BBG9590	55525.50	20.04	99.70%	99.51
		BBG9896	60156.38	21.58	99.70%	99.63
		BBG99111	61696.89	22.19	99.69%	99.51
JNG	Jining,Shandong	GRG43	80147.53	27.97	99.69%	98.74
		GRG45	77785.36	26.92	99.57%	98.73
		GRG47	70586.37	24.5	99.55%	98.74
		GRG77	67499.73	23.42	99.64%	98.76
		GRG76	58886.59	20.43	99.10%	98.65
		GRG75	62095.07	21.69	99.58%	98.79
		GRG15	74124.89	26	99.70%	98.62
		GRG16	64099.62	23.07	99.68%	98.74
		GRG17	58185.73	20.13	99.66%	98.66
		GRG18	66355.72	22.34	99.65%	98.53
MT	Shangqiu,Henan	MTG0003	33073.79	12.05	99.71%	99.3
		MTG0005	31190.80	11.44	99.71%	99.29
		MTG0006	32001.89	11.67	99.72%	99.29
		MTG0007	36583.23	13.23	99.72%	99.3
		MTG0012	27846.19	10.16	99.62%	99.3
		MTG0013	28180.39	10.31	99.67%	99.26
		MTG0001	28026.49	10.39	99.66%	99.34
		MTG0002	23833.58	8.79	99.58%	99.28
		MTG0009	28787.71	10.5	99.67%	99.3
		MTG0010	27156.25	9.93	99.62%	99.29

Supplementary Table 2 Sequencing information of 95 published goats used in this study

Abbre viation	Sampling location	SRR	ID	Size_Mb	Depth(X)
IMC	Inner Mongolia	SRR4052649<br>SRR4096727	IMCG02	13352.12	5.15
		SRR4052677<br>SRR4096728	IMCG05	10378.24	5.92
		SRR4053244<br>SRR4096730	IMCG25	20533.36	7.12
		SRR4053410<br>SRR4096705	IMCG28	16367.42	6.06
		SRR4053460<br>SRR4096706	IMCG29	19661.99	6.95
		SRR4053519<br>SRR4096707	IMCG30	17400.57	6.37
		SRR4053642<br>SRR4096720	IMCG43	10480.34	4.17
		SRR4051874<br>SRR4101814	IMCG21	18138.44	6.33
		SRR4101803<br>SRR4051950	IMCG37	12121.82	4.73
		SRR4101805<br>SRR4051959	IMCG39	14024.62	5.1
		SRR4051960<br>SRR4051961	IMCG47	28440.14	9.08
		SRR4051919<br>SRR4101817	IMCG311	15150.43	5.29
		SRR4051941<br>SRR4101820	IMCG35	13081.32	5.05
		SRR4101821<br>SRR4051947	IMCG36	11200.72	4.55
		SRR4101735<br>SRR4052046	IMCG101	13212.90	4.64
		SRR4101737<br>SRR4052247	IMCG29	19661.99	6.95
		SRR4052353<br>SRR4101744	IMCG04	21710.11	7.55
		SRR4101745<br>SRR4052354	IMCG051	16082.35	4.36
		SRR4101749<br>SRR4052120	IMCG141	15530.00	5.54

		SRR4052177<br>SRR4101750	IMCG19	18957.64	6.5
		SRR4101751<br>SRR4052202	IMCG213	13769.74	5.3
		SRR4101747<br>SRR4052075	IMCG121	12505.18	4.68
		SRR4101753<br>SRR4052224	IMCG27	15395.76	5.75
		SRR4052234<br>SRR4101754	IMCG28	16367.42	6.06
		SRR4051772<br>SRR4096722	IMCG10	36865.38	11.57
		SRR4051866<br>SRR4101802	IMCG13	28479.47	8.98
		SRR4051917<br>SRR4101816	IMCG11	24991.59	12.38
		SRR4051928<br>SRR4101818	IMCG14	31556.88	10.34
		SRR4051939<br>SRR4101819	IMCG15	24113.19	8.04
		SRR4051964<br>SRR4101808	IMCG12	39729.48	12.8
		SRR4052103<br>SRR4101748	IMCG313	30156.49	9.86
		SRR4052272<br>SRR4101738	IMCG01	26447.13	8.33
		SRR4052346<br>SRR4101741	IMCG33	29215.81	9.71
		SRR4052349<br>SRR4101742	IMCG55	36349.01	11.9
		SRR4052525<br>SRR4096703	IMCG111	37597.81	8.58
		SRR4052590<br>SRR4096725	IMCG31	28631.05	9.39
		SRR4053363<br>SRR4096704	IMCG710	23137.77	7.63
LNC	Gaizhou, Liaoning	SRR4064255<br>SRR4107102	LNC18	19120.07	7.23
		SRR4064259<br>SRR4107114	LNC21	18442.79	6.96
		SRR4067780<br>SRR4107116	LNC24	25718.73	9.33
		SRR4067798	LNC33	25739.53	9.34

		SRR4107119			
		SRR4068049<br>SRR4107104	LNC37	22317.17	7.98
		SRR4068051<br>SRR4107105	LNC38	17313.81	6.45
		SRR4068053<br>SRR4107107	LNC47	26501.94	9.35
		SRR4068056<br>SRR4107110	LNC52	25383.53	9.16
		SRR4068057<br>SRR4107111	LNC54	22658.95	8.16
		SRR4068058<br>SRR4107112	LNC56	24913.93	8.91
		SRR4067779<br>SRR4107115	LNC22	11526.85	4.75
		SRR4067781<br>SRR4107117	LNC31	14464.54	5.68
		SRR4067799<br>SRR4107120	LNC34	13730.86	5.37
		SRR4067800<br>SRR4107121	LNC35	12068.03	4.93
		SRR4067801<br>SRR4107122	LNC36	10564.92	4.46
		SRR4068052<br>SRR4107106	LNC39	15629.86	5.53
		SRR4068054<br>SRR4107108	LNC48	11129.55	4.47
KO	Korean	SRR1265926	KOG06	45009.41	14.92
		SRR1265927	KOG07	44747.49	14.91
		SRR1265928	KOG08	45333.81	14.94
		SRR1265929	KOG09	44544.50	14.76
		SRR1265930	KOG10	43077.48	14.39
		SRR1265931	KOG11	45720.98	15.29
		SRR1265932	KOG12	44948.16	15.11
		SRR1265933	KOG13	46550.37	15.46
		SRR1265934	KOG14	47704.28	15.73
		SRR1265935	KOG15	45500.75	15.26
		SRR1265936	KOG16	43953.58	14.59
		SRR1265937	KOG17	43054.14	14.38
ANG	Evry, France;South Africa;Madagascar	ERR4133570	FANG0001	41597.54	13.93
		ERR4133551	FANG0002	35697.88	11.49
		ERR4133594	FANG0003	18590.68	6.53
		ERR4133600	FANG0004	33849.04	11.46

ERR4133602	FANG0005	31924.81	10.83
ERR4133549	FANG0006	37378.87	10.28
ERR4133646	FANG0007	34118.43	11.55
ERR4133649	FANG0008	29679.00	10.08
ERR4133643	FANG0009	37437.20	12.86
ERR4133597	FANG0010	16980.35	6.01
ERR4133588	FANG0011	39509.40	13.07
ERR4133619	FANG0012	18814.75	6.54
ERR4133615	FANG0013	39342.96	13.72
ERR4133622	FANG0014	33621.92	11.7
ERR4133550	FANG0015	42170.35	13.13
ERR4133591	FANG0016	32655.34	11.11
ERR4133618	FANG0017	40490.15	13.93
ERR4133606	FANG0018	35121.24	11.88
ERR4133625	FANG0019	37295.04	13.03
ERR4133628	FANG0020	34410.02	12.12
ERR3281402	MANG0027	103932.50	29.9
ERR3281403	MANG0028	68239.26	22.11
ERR3281404	MANG0029	80376.99	25.78
ERR3281405	MANG0030	79312.28	25.62
ERR3281406	MANG0031	62641.33	19.91
ERR3281407	MANG0033	76354.83	21.87
ERR4133525	ZANG0374	41438.84	14.24
ERR4133528	ZANG0388	34554.79	11.94
ERR4133517	ZANG0397	31028.87	10.73

Supplementary Table 3 Sequencing information of Wild goats used in this study

**Abbreviation**	**Sampling location**	SRR	**ID**	**Size_MB**	**depth**
IRW	Alamout, Iranian	ERR470100	IRWG01	45083.46	15.74
		ERR470104	IRWG04	43442.83	15.49
		ERR470106	IRWG06	45847.73	15.35
		ERR340426	IRWG26	37967.84	13.72
		ERR340328	IRWG28	19454.66	7.24
		ERR340329	IRWG29	17013.47	6.22
		ERR340330	IRWG30	37395.69	13.64
		ERR340331	IRWG31	34581.11	12.73
		ERR340333	IRWG33	19084.62	7
		ERR340334	IRWG34	37587.13	13.58
		ERR340335	IRWG35	19737.88	7.26
		ERR340336	IRWG36	19252.59	7.13
		ERR340338	IRWG38	34183.70	12.48
		ERR340340	IRWG40	36536.11	13.22
		ERR340341	IRWG41	15342.29	5.67
		ERR340342	IRWG42	19017.87	7.03
		ERR340343	IRWG43	21705.31	7.97
		ERR340344	IRWG44	33823.57	12.31
		ERR340345	IRWG45	35354.22	12.85
		ERR340347	IRWG47	37973.28	13.76
		ERR340348	IRWG48	32400.27	11.56
BS	Central Asia	SRR5803211	BS1	40343.35	14.58
		SRR5803192	BS5	34274.71	12.13
		SRR5803193	BS8	48985.98	17.08
FAG	Central Asia	SRR5803200	FAG02	27401.58	9.71
		SRR5803204	FAG05	90588.81	11.5
		SRR5803207	FAG04	32469.98	31.11

BS (Capra sibirica)

FAG (Capra falconeri)

Supplementary Table 4 Sequencing platforms for 236 goats of 13 goat breeds.

**Breed or Species**	**Abbreviation**	**Sequencing Platform**	**Length**	**Sample Size**
Chaidamu Cashmere Goat	CDMC	BGISEQ-500	100	30
Inner Mongolia Cashmere Goat	IMC	Illumina HiSeq2000	100	37
Ujumqin Cashmere Goat	UC	BGISEQ-500	100	12
Liaoning Cashmere Goat	LNC	Illumina HiSeq2000	100	17
Iranian Wild Goat	IRW	Illumina HiSeq2000	100	21
Matou Goat	MT	BGISEQ-500	100	10
Korean native goat	KO	Illumina HiSeq2000	100	12
Jining Grey Goats	JNG	Illumina Hiseq 2500	150	10
Jintang Black Goat	JTB	BGISEQ-500	100	10
Guizhou Black Goat	GZB	BGISEQ-500	100	8
Yunnan Black Bone Goat	YNBB	Illumina Hiseq 2500	150	28
Angora goat	ANG	Illumina HiSeq 4000	150	29
Chengdu Brown Goat	CDB	BGISEQ-500	100	12

Supplementary Table 5 Published ancient samples used in this study.

Sample	Period	Bioproject
Potterne1	Bronze Age	PRJEB26011
Qazvin1	Bronze Age	PRJEB26011
Bulak2	Bronze Age	PRJEB26011
Kohneh2	Bronze Age	PRJEB26011
Acem1	Bronze Age	PRJEB26011
Acem2	Bronze Age	PRJEB26011
Azer3	Bronze Age	PRJEB26011
Darre2	Chalcolithic	PRJEB26011
Fars4	Chalcolithic	PRJEB26011
Geor2	IronAge-Medieval	PRJEB26011
Kazbeg1	IronAge-Medieval	PRJEB26011
Azer4	IronAge-Medieval	PRJEB26011
Semnan1	Neolithic	PRJEB26011
Semnan10	Neolithic	PRJEB26011
Semnan13	Neolithic	PRJEB26011
Monjukli8	Neolithic	PRJEB26011
Blagotin3	Neolithic	PRJEB26011
Lur12	Neolithic	PRJEB26011
Blagotin1	Neolithic	PRJEB26011
Blagotin16	Neolithic	PRJEB26011
Blagotin2	Neolithic	PRJEB26011

Supplementary Table 6 The genotype frequencies of the homozygous 582bp deletion (-/-) at *LHX2* locus for 15 goat breeds, obtained from whole genome sequencing.

**Breed**	**LHX2(del/del)**	**Size**	**Genotype Frequency**
CDMC	21	30	70.00%
YNBB	8	28	28.57%
CDB	1	12	8.33%
JNG	1	10	10.00%
GZB	1	8	12.50%
IMC	22	36	61.11%
IRW	17	21	80.95%
JTB	0	10	0.00%
KO	4	12	33.33%
LNC	9	17	52.94%
MT	4	10	40.00%
UC	12	12	100.00%
ANG	7	29	24.14%
BS&FAG	1	6	16.66%
AG	7	21	33.33%

Supplementary Table 7 The genotype frequencies of the homozygous 504bp deletion (-/-) at FGF5 locus for 15 goat breeds, obtained from whole genome sequencing.

**Breed**	**FGF5(del/del)**	**Size**	**Genotype Frequency**
CDMC	23	30	76.67%
YNBB	0	28	0%
CDB	0	12	0%
JNG	2	10	20%
GZB	0	8	0%
IMC	30	37	81.08%
IRW	0	21	0%
JTB	0	10	0%
KO	0	12	0%
LNC	17	17	100%
MT	0	10	0%
UC	0	12	0%
ANG	1	29	3.45%
BS&FAG	0	6	0%
AG	0	21	0%

Supplementary Table 8 The frequencies of the 582 bp deletion near *LHX2* with PCR amplification.

Breed	**del/del**	**del/+**	+/+	**Sum**
CDMC	131(54.13%)	91(37.6%)	20(8.27%)	242
IMC	8(66.67%)	3(25%)	1(8.33%)	12
UC	30(75%)	9(22.5)	1(2.5%)	40
JNG	2(16.67%)	8(66.67%)	2(16.66)	12
WSW	0	2(16.67%)	10(83.33%)	12
JTB	1(8.33%)	5(41.67)	6(50%)	12

The frequencies of homozygous (del/del), heterozygous (del/+) and wide type of the 582 bp deletion near *LHX2*, obtained from PCR amplification.

Supplementary Table 9 The frequencies of the 504 bp deletion near *FGF5* with PCR amplification.

Breed	del/del	del/+	+/+	Sum
CDMC	194(80.16%)	45(18.6)	3(1.23%)	242
IMC	12(100%)	0	0	12
UC	3(7.5%)	19(47.5%)	18(45%)	40
JNG	3(25%)	8(66.67%)	1(8.33%)	12
WSW	0	1(8.33%)	11(91.67%)	12
JTB	0	0	12(100%)	12

The frequencies of homozygous (del/del), heterozygous (del/+) and wide type of the 504 bp deletion near *FGF5*, obtained from PCR amplification.

Supplementary Table 10 Primers used for amplification of deletion seences

Primer names	Sequence 5'-3'	Length (bp)
LHX2_del_F2	CAGTACGGAGCAAGTAAACGG	630 bp for del/del
LHX2_del_R2	ACCATTCCACTTGTCCACCT	1214 bp for +/+<br>630/1214 bp for del/+
FGF5_del_F1	ACAGCGTGTGATCTTTTCTCTG	235 bp for del/del
FGF5_del_R2	TCTTGGTCTGGCTGTGATCA	742 bp for +/+<br>235/742 bp for del/+

Supplementary Table 11 Samples for PSMC analysis

Sample	depth(X)
BBG31107	26.94
FANG0017	13.93
WMG0004	15.93
CMS0088	13.21
HXR0127	12.6
IRWG01	15.74
KOG14	15.73

Supplementary sequence 1

Distal enhancer-like signatures are shown in red, the CTCF binding site is displayed in bold, and the 582bp deletion sequence is underlined.

>goat:chromosome chromosome:ARS1:11:94266769:94278930:1
ATACGAGTTGCCATGACTATTCTGTTGCTTGGATTCATTCACCATCTACTCTCACCAGT
TGTACTAATTAAATCTTCAGAGCTCTTCCAGGATCTCTATCGCTTTCTTTGCTTCAGAA
AGTCAATCAGAGTTTATTGCCCCGAAAAGGTAGGATTCAGAGTCAACATTTACTAAA
TGAGTAGTATGTGTCCAGCAATGGGCTACTGGCTTTCATATCTTTCAAAAACCCTGAG
GGGAGTATTAACACTACTCTCTTCTTACTGAAAAGATTAAGTTGCCTAGGGCAACATG
GCTAGGGAGGATCAAGATACACAGGAAGAATTTCAACAAAACCATTATGACTTGAAG
CCTGGTGCTCTCTCCTCTTGTCCTTTTTCTTGGGAAAAGCTAGCAGAAGCACGGCTGT
CTGAATCATCTAGCACGGAAAACACATAAATATCAAGACAGCACAAGAGAAAAAAC
ATGAATTTTCACAAGGTCTATATGAACTCCTAATGATATAGTCTAACCTCACATCATA
CTTCATTCGCTCATCAGTGACCATGCTTTCAGTTTGCAGAAATAAAAAAAAAATTTGA
GTTCTTGACTTCTGACCACATAGTAGGTGCTCCAATTGTGTTAACAAAATCCACACAG
ATGTCTGCCAATGTGAGTGCCATTAGGGGTTTTTCAAGTATATTAAGATGTCAGTGAC
TTGGAAGGAAGTTATAACCAACCTAGATAGCATATTCAAAAGCAGAGACATTACTTT
GCCAACTAACACCCATCTAGTCAAGGGTGTGGTTTTTCCAGTAGTCATGTATGGATGT
GAGAGTTGGACTGTGAAGAAAGCTGAGTGCCGAAGAATTGATGCTTTTGAACTGTGG
TGTTGGAGAAGACTCTTGAGAGTCCCTTGGACTGCAAGGAGATCCAACCAGTCCATT
CTGAAGGAGATCAGTCCTGGGTGTTCTTTGGAAGGAATGATGCTAAAGCTGAAACTC
CAGTACTTTGGCCACCTCATGCAAAAAGTTGACTCACTGGAAAAGACTCTGATGCTG
GGAGGGGTTGGGAGCAGGAGGAAAAGGGGACGACAGAGGATGAGATGGCTGGATG
GCATCACCGACTCAATGGATGAGAGTTTGAGTGAACTCCGGACAGGGAGGCCTGGCA
TGTTGCAATTCATGGGGTCGCAAAGAGTCGGACAAGACTGAGTGACTGAACTGAACT
GAACTGACTTGAATATCACCTCTTAGTGTTTTTTGGTAGAGATCTGATTCTTAAAAGA
GTCATATAAAAAAGTTATCAATCCTATAATCTAAGAAACTGACTACACCACTAAAAT
AATACACAAGATACTCTGCCAATGTTCTCAATGCAATGGCTGAAAAGAAACATCACC
TCTCTAGATCCATTTATACAGATTTATTTAAAACTACCCTAGTGGACTACATCAGCAT
CCAATGGTACTGAGAGCTTAAATTACTCTTAGCTCTGTCCTGTTCTAGACTGTTGTTGC
TGCTGCTGCTAAGTTGCTTCAGTCATGTCTGATTCTTTGCGACCCCATAGACTCAGAG
CTCTTTCAGAAATATGGAAATAATTTTTAGTGCTAATCTGTACTCACAAATGGAGAAG
GAAATGGCAACCCACTCCAGAATTCTTGCTTAGAGAATACTGTGGACGGAGGAGCCT
GGTGGGCTGCTGTCCATAGGGCTGCACAGAGTCGGACACGACTGAAGCGACTTAGCA
CGCATGCATGCACCGGAGAAGGAAATGGCAGCCCACTCCAGTGTTCTTGACTGGATA
ATCCCAGGGATGGTGGAGCCTGGTGCGCTGCTGTCTATGGGGTCGCACAAAGTCGGA
CACGACTGAAGCAACTTGGCAGCAGCAGCAGCAGTACTCACAAATGCCTTCTAAACA
AACTTAGTCAGCCTTTATTATATAGTCTCAAGATGAAAAGGACTCTATTGATGCAAAA
TAATCAAAGAATAAATACTGTCTTAGAGAACTGGTCCTGAATCTGGTTATGTATTAGT
AATTTGGGAAACCTTAAAAAAATAGACATCCCACAGTCCCATCTTAGACTTTTTGAAT
TATTATTATAATAAAAATTTAAGTTCTTGGGTACTACCTCTACACATTCTGATTCAAAA

GGTCTAATTTTTATTTAAATTGGAGAATAATTAGTTTACAACATTGTGATGTTTTTAGC
CATACATCAACATGAATCAGCCACAGGTATACATTTGTCCCACCCCATCCTGAATCCC
CTCCCACCTCCTTCCTTACCCTATTCCTCTGCGGTTGTCCCAGAGCATCAGCTTTGAGT
GCCTTGCTTCATGCATCAAACTTGCACTGGTCATCTATTTTACACATAGTAATGTACAT
GTTTCAGTGCTATTGTCTCAAATCATCCCCTGCTCTCCTCCTCTCCCTCTGAGTCCAAA
AGTTTGTTCTTTACATCTGTGTCTCCTTTACTGCCCTGCATGTAGGATCATTGGTATTG
TCTTTCTAAATTCCATATATATGCGTTAATATATAGTATTTGTCTTTCTTTTCAACTTAT
TTCACTCTGTATAATAGGCTCCAGGTTCATCTACCTCATTAGAACTGACTCAAATGCA
TTCCTTTTTGTAGCTGAGAACTTAAATTTTTAATAAGCTCTCTGAGTGATTTCTAACAC
AACTAACTCAGGGATCAGCATTTGGGAATCACTAACAATCTGAATCCTCTGTCTAGTC
CAAAACATGTGCTCAATGTGTTGAAGCAAGAAGGGCAGGAGAGGAAGGTAAGAACT
AGTAGGGAGACCGTGATTGGTTGAAATCACAGCAATATTTAAAGTACTACAAATTAA
AGTGTTGTTACTCTATATATTATATAGCTTATACATTCTCTATAACATCAATGTTCTTC
ATTCTTATAGTCTAACATGAACAAGAGAAAAGGAACTCGCACATAATTAAATGAAGA
ACATAGTTCAAAAAGCAGCCAAGTGTATTTACATTTAAAGACCAGAGAAAAACCACA
TAAATAAAACATACCAATCTTTTCTATTTCCTGCCAATGGAAGTTCCTTTCATTTATTT
CTAACAGTTTTTAATATTCTCTGAGCCACAATAAAATTTTATGTGATGGAGATTAGAG
AGGCTATGCTATTTGAAAGTTTGGCTGCAGAGTTTGTATTTCATGCCACTTCATTACAT
TTTGTTAAGGATTTCCCTCTTCACCAAAAGGGATTAGTTTTCTTCTCACTTCTTTGAAT
ACTCTTCTTAGGTTGTTAGTGTGAACTAGGTTAATCTCTCTTTTAACATATAAAAGATG
AACAAACAAAATGTTCAATTTATTTTATTTTTTTAAATGGAACACTAAATTTAGTCTTA
GAATTCTCTGTTTGGTAAGTTTCCAGTTACAAGGTCTTGGCTTCCCTTGAGCACAGTTT
GAAACAAAATAATGCAGTTTGTTCTGATGGCTGGAACATGCAGAGTGGAGTGTGCAG
GCAAAGAGGCTCTGGCTCTGGTCCAGTATTTCAAACCTTCTTAAACTGCCACTCTCAT
CTAGGCTTCAAGGGCTCCAGATAAGCAAACAGGATTAGTTATTTCTAGCCCAGAAGA
GCTAGAAAAGCTTTATACACATCATTTAGGGACATAGCCCACTCTACAAGTCAGGGT
AGTCATGACCAGGTAGATCTGATCAGGAAAGAAGTAAAGATGAAGCTAAAGATCTG
AAACATTCCCTAGTCCACAAACTGACTCTTCTTAGGTCACAAAACCAGGTGGTGCTAA
GACACTGGATGCTGTGAATGGAAGTGGATAGGACAGGACTGGAGCAAGGTTGGGAA
GTTCCCCATCCCTTCATGCCAACACTCAAGTGTCTACTAAGGCCACAACTGAGGGTGC
TGGGGGGAAAAAGAAATACCACCCACCACTCTCCAGGCCATCAAGTCCTGCCCAAGG
CACATTTCTGGGACTAATTGGAAGAAATTGACGGAGAGATATTGGGCTTTTTTTTTT
TTTTTTCCTTCTCTACATGGTGTATTTGGAGAAGGAAACAGCAACCCACTCCAGTATG
CTTGCCTGGAAAATTTCATGGACAGGGAGCCTGGTGGGCTCTAGTCCATGGGGTCAC
AGAGTCAGACACGACTGAGTGATTAAATGACATAACATACTATTAGAAAAATAGTAA
TTCTTTTCATAGTTGTGGGTATCACACAAATAATTAGGTGTGGAGGCTCAATTAATTA
GGTAACAGCTTTGCCACCAAATTGTGCTTCCTTCCTAAGAACTTGTTTTAAAAAGCAT
CTCTTAAGTGTGACAGATATTCTTAACAGTGTGCCTCTGGTTCTCGTGACAGTTCCCA
CACAAGGATTCTGATGTTTGTTTGAGAGGTGACACCCAATCAGTGTAACCTGGTCCCC
ACAGCAAGCTCTTTGAAGGGGCCAAGATTCATCTGTGTAACTCCTGGTTGTTACTCCC
TTTGCTGAAGCCAAAGCCTCCCTTCCTCTGACACATCATCATAATGACCCACCTGCAT
GTCCTTTTCTTCTATTACGAGGGCATAACCTTGGGCAAGAGAACCCATCTCTCTGAGT
GGATGGATCATTCTGTAAAATGGGGATAATATTGCTCTTCTAGGAATTCTGTTGCAAT
TAGATGATATTATTTCTATAAAGGTTTTATTTTAGACCTACTTGCTATCCACAAGAGTA

ACCAAAAAACAATCTGAGTTAAACTCAGATTTGAAAATCTGGAAGAAAAATGGGGCC
TTTGTGCTAGAAGAAATCTCACTGAAGCATTTACATTAGAGAACAAGATGTAGTTAA
ATCTTCCCTATTTATCATTTATAAAGGAAAACAATCAATATACACTGCACAAAAAGAA
CAAAAAGTTAACATTTTTCTTACACAGAATATCACAACATTATTGACCGGGAAAGCCT
GAAATGAATATTACCTGACTCTGAACTAAACATGGCTGGGTTTGTCTCTATTGAC
ACACAGTCAGTTTTATGAAACATGGCACTCTGGGATGTTCAACAGCTTTCATCTT
TCAGATGCTTGATGGCTCTGAGAAGCATCAAGCCTACATCTTTATGAAACAAAAT
TGAGAAAAATGTGTTTTGTCAAGGTATGGAAGGCCACATCCGGATCTGTCAAGA
AAGGGTCATCAAAAACTTCAGGCTACAGTATTCTTTGCATTTTCCTTCATCAAAA
AGAAAATAAAAAGTCAGTACGGAGCAAGTAAACGGTAACAATTAGAAAGCTTTT
AAACTCTAAAATTGAAGAGGTCCAAAGGCTTGGATAAAAGGGATGTCTACTCAGTTA
CTTATCCCCAGAGAAACAACACAAAACAGATCAGGGTGTTGCTGATCCCCTATGAGG
TAAAAGAAGACCTTCTCCTTCCCTAAAGAGATATCCACCATCAATTAGTAAAACTTAG
AACTCAGTTAAGAATCAACTGTCTGTTTAAAAGTTTTTAAGTCAAAGTCATGTATTTA
TAGCCTGAAAAACAAAACAAAAAGTGTTAAAGGCTTATACTACTGACAAATAGCAGC
CCATCTCATGATATCCCTTACCTTCAATCAAAAGTAATCACATTCAACACAATATTTA
TCTCCTTATTTTTAAAAAGTATTTAATTTAAAAATTTAAACATTAATCTTTGACTTCCC
ATTATAGATATTAATAATTTATCAAGAACATATACATATACCTTATGCCAGACTGTTC
TAGGAGTTTTGCAACTCGTTTTATTTGACATGAGGTCACTGGTGACCAAACAAAAACT
GTTTCAGTGGAGGCAGGGGCTAAATCACAACACAAGACAGAGGAGAGAATGAGAAG
AGAGGAATTAGACAGTAAACAGAAGCAACTCTCTTGAGGAATTGCATTCTAAAAGGA
AGAAAAGAAATGGGATAAAAGAGATGTGTTTAAGGATGTTTTGTTTTGAAGTTTGGG
AGACACTATACTGTGCTAGTAAGTGCATAAGACTGTTCAGTTCAGTCGCTCAGTTGTG
TCCGACTCTTTGCGACCCCATGAATTACAGCACGCCAGGCCTGTACCAGTAAAGAAA
ACAAAAAACAAAAAATGATGCAGGAGAGAGAAAGCTCAAGGCTGGAGGAATTCCGT
AAGGTGACAAGAAGGATGCTGCAGTGTTAGCTTTCCCACAAGGCACTCCTTGGTCAT
CTAGCTAAACTGCAATCCCTGCTACACCAGTCGCCATCCACTCTCCTCCTCTGCTTAAT
TGTACTCCTTGTACTTAGAACTAGCTACTATTCTATATTTTCAACCCACCTCATTTATT
GATCTACTCGACTAGAACATAAGCTTCATATGAGGACAGGGACTTTTGCCTATTTTGT
TCACTGCTGTATCACCAGTACCTATGACAGTGCCACAAAAACTATTTGCTGAATGAAG
TCAGGTGGACAAGTGGAATGGTTGGCCTAGACAGAATGGACAGTTCATCCACAATGG
AGAAGGTGAGTGGAGGACTAGCTACCGCAGGTTGACAGGTGTGGTAGGGGCTGGTGC
CACTCTCTCCTGAGCGCTTTCATTAGATAAAGATGGAATTTTAGGTAAGAAATATTTT
TCCCTTAGAAGTTTAGGCACTGCTTCACTATCTTCTCGTTTCTAGAACTGCCACTGCGA
AATGTTGTGCAAGTCTGATTCTTAATGTTTATACAATGACTACTTTGTCTCTATCTCTG
TATTGTAAAATTACATGATAAAAAGGGCTTTTTAACTTTCAAAGATTTCCTGAAGCCA
AAACCTCCCTTCCCCTGACACTACATCATAACGACCAATGTGCCTGTCTCTTCTATGA
CTACCACGTGGCCTTAGGCAAGTTAACCCATGTATCTGAGTGAGTGGGCCATTCTGTA
AAATGGGGATAATATTGCTTTTTAGACTTTTAAAGAATTCTGAA

Supplementary sequence 2

Ins sequence(551bp)

GAAAAACAAAACAAAAAGTGTTAAAGGCTTATACTACTGACAAATAGCAGCCCATCTC
ATGATATCCCTTACCTTCAATCAAAAGTAATCACATTCAACACAATATTTATCTCCTTATT
TTTAAAAAGTATTTAATTTAAAAATTTAAACATTAATCTTTGACTTCCCATTATAGATATT
AATAATTTATCAAGAACATATACATATACCTTATGCCAGACTGTTCTAGGAGTTTTGCAA
CTCGTTTTATTTGACATGAGGTCACTGGTGACCAAACAAAAACTGTTTCAGTGGAGGC
AGGGGCTAAATCACAACACAAGACAGAGGAGAGAATGAGAAGAGAGGAATTAGACA
GTAAACAGAAGCAACTCTCTTGAGGAATTGCATTCTAAAAGGAAGAAAAGAAATGGG
ATAAAAGAGATGTGTTTAAGGATGTTTTGTTTTGAAGTTTGGGAGACACTATACTGTGC
TAGTAAGTGCATAAGACTGTTCAGTTCAGTCGCTCAGTTGTGTCCGACTCTTTGCGACC
CCATGAATTACAGCACGCCAGG

Supplementary Reference

1. Huang, J. et al. A reference human genome dataset of the BGISEQ-500 sequencer. *Gigascience*. 6, 1-9 (2017).
2. Li, H. & Durbin, R. Fast and accurate short read alignment with Burrows-Wheeler transform. *Bioinformatics*. 25, 1754-1760 (2009).
3. Letunic, I. & Bork, P. Interactive Tree Of Life (iTOL): an online tool for phylogenetic tree display and annotation. *Bioinformatics*. 23, 127-128 (2007).
4. Schiffels, S. & Durbin, R. Inferring human population size and separation history from multiple genome sequences. *Nat. Genet.* 46, 919-925 (2014).
5. Lai, F. N. et al. Whole-genome scanning for the litter size trait associated genes and SNPs under selection in dairy goat (Capra hircus). *Sci Rep*. 6, 38096 (2016).
6. Wang, L. et al. Understanding the Genetic Domestication History of the Jianchang Duck by Genotyping and Sequencing of Genomic Genes Under Selection. *G3 (Bethesda)*. 10, 1469-1476 (2020).
7. Zhou, Y. et al. Metascape provides a biologist-oriented resource for the analysis of systems-level datasets. *Nat. Commun.* 10, 1523 (2019).
8. Henkel, J. et al. Selection signatures in goats reveal copy number variants underlying breed-defining coat color phenotypes. *PLoS Genet.* 15, e1008536 (2019).
9. Wu, Z. et al. Using WGCNA (weighted gene co-expression network analysis) to identify the hub genes of skin hair follicle development in fetus stage of Inner Mongolia cashmere goat. *PLoS One*. 15, e243507 (2020).
10. Yang, F. et al. Skin transcriptome reveals the periodic changes in genes underlying cashmere (ground hair) follicle transition in cashmere goats. *BMC Genomics*. 21, 392 (2020).
