# Supplementary material for: Selection signatures in goats reveal a novel deletion mutant underlying cashmere yield and diameter
Source: Gigascience. 2022 Nov 3;11:giac107. doi: 10.1093/gigascience/giac107 (PMC9633279; doi:10.1093/gigascience/giac107)
Supplement: giac107_GIGA-D-22-00003_Revision_4 [file giac107_giga-d-22-00003_revision_4.pdf]

GigaScience

Selection signatures in goats reveal a novel deletion mutant underlying cashmere yield and diameter

--Manuscript Draft--

**Manuscript Number:**	GIGA-D-22-00003R4
**Full Title:**	Selection signatures in goats reveal a novel deletion mutant underlying cashmere yield and diameter
**Article Type:**	Research
**Funding Information:**	Science and Technology Innovation Strategy Projects of Guangdong Province (2019B020203002) — Doctor Yong Li<br>Innovative Research Group Project of the National Natural Science Foundation of China (31872560) — Professor Ming Fang<br>Shenzhen Municipal Human Resources and Social Security Bureau (JCYJ20180307163440037) — Doctor Yong Li
**Abstract:**	Cashmere traits were deployed for fiber yield and quality during the domestication of goats. However, the genetic alterations underlying cashmere trait selection are still unclear. We sequenced 120 Chinese native goats including two cashmere goat breeds and six ordinary goat breeds. The genome-wide selective sweep of cashmere goat and ordinary goat revealed a novel set of candidate genes as well as pathways, such as Nuclear factor kappa-B and Wnt Signaling pathways. Of them, LHX2 regulating hair follicle development, was evident from the strongest selection signal when comparing the Uhumqin cashmere goat and ordinary goat. Interestingly, we identified a 582-bp deletion at 367 kb upstream of LHX2 with higher frequency in cashmere goats and their ancient relatives. This mutation was probably arisen along the breeding procedures, and is putatively responsible for cashmere production and diameter, as revealed by association studies. Luciferase assay showed that the 582-bp sequence, which acts as an insulator, restrains the expression of LHX2 by interfering its upstream enhancers. Our findings provide new insights into the genetic formation of cashmere and facilitate subsequent molecular breeding for cashmere goat improvement.
**Corresponding Author:**	Yong Li<br>BGI-Shenzhen: BGI Group<br>Shenzhen, CHINA
**Corresponding Author Secondary Information:**	
**Corresponding Author's Institution:**	BGI-Shenzhen: BGI Group
**Corresponding Author's Secondary Institution:**	
**First Author:**	hu han
**First Author Secondary Information:**	
**Order of Authors:**	hu han
	Man-Man Yang
	Jiang Dan
	Sheng-Yu Chao
	Xing-Ju Zhang
	Qiang Wei
	Tao Chen
	Qi-Ju Wang

Powered by Editorial Manager® and ProduXion Manager® from Aries Systems Corporation

	Cheng-Ye Yang
	Bater Wulan
	Ting-Ting Zhang
	Gang Gen
	Dala Mengke
	Bin Li
	Wei-Dong Deng
	Ze-Pu Miao
	Ran Wang
	Qing-Feng Zhang
	Lin Li
	Ming Fang
	Yong Li
**Order of Authors Secondary Information:**	
**Response to Reviewers:**	1 GigaScience allows 3 joint first authors at most and 2 corresponding authors at most, please make sure the numbers meet our requirement.<br>Reply: We have revised the manuscript as requested.Yes, * represents Joint first author, and # represents Corresponding author.<br>2 Note that figures must be submitted as separate image files, not as part of the submitted manuscript file.<br>Reply: We have revised the manuscript as requested.At the time of this manuscript submission, the pictures were uploaded as required.<br>3 URL link shouldn't be included in the main text. please cite it as a reference.<br>Reply: Urls were removed from the manuscript and references were added.<br>4 Please add details here.<br>Reply: Reference were adde<br>5 List of abreferences were addedbreviations<br>Reply: A list of abbreviations was added to the manuscript and sorted.<br>6 Please add details here if available<br>Reply: Fund information has been refined in the manuscript.<br>7 All references, including URLs, must be numbered consecutively, in square brackets, in the order in which they are cited in the text, followed by any in tables or legends.<br>Reply: We have revised all the references in the manuscript according to the requirements of the GigaScience format.
**Additional Information:**	
**Question**	**Response**
Are you submitting this manuscript to a special series or article collection?	No
**Experimental design and statistics**<br><br>Full details of the experimental design and statistical methods used should be given in the Methods section, as detailed in our Minimum Standards Reporting Checklist. Information essential to interpreting the data presented should be made available in the figure legends.	Yes

Have you included all the information requested in your manuscript?	
**Resources**<br><br>A description of all resources used, including antibodies, cell lines, animals and software tools, with enough information to allow them to be uniquely identified, should be included in the Methods section. Authors are strongly encouraged to cite Research Resource Identifiers (RRIDs) for antibodies, model organisms and tools, where possible.<br><br>Have you included the information requested as detailed in our Minimum Standards Reporting Checklist?	Yes
**Availability of data and materials**<br><br>All datasets and code on which the conclusions of the paper rely must be either included in your submission or deposited in publicly available repositories (where available and ethically appropriate), referencing such data using a unique identifier in the references and in the “Availability of Data and Materials” section of your manuscript.<br><br>Have you have met the above requirement as detailed in our Minimum Standards Reporting Checklist?	Yes

Manuscript

Click here to access/download;Manuscript;Goat_Manuscript-revised_SCEV4-2.docx

Selection signatures in goats reveal a novel deletion mutant underlying cashmere yield and diameter

Hu Han[1,2]*, Man-Man Yang[2,3]*, Jiang Dan[1]*, Sheng-Yu Chao[4,6], Xing-Ju Zhang[2,3], Qiang Wei[2,3], Tao Chen[2,3], Qi-Ju Wang[4], Cheng-Ye Yang[5], Bater Wulan[6], Ting-Ting Zhang[2,3], Gang Gen[6], Mengkedala[7], Bin Li[2], Wei-Dong Deng[8], Ze-Pu Miao[2], Ran Wang[2], Qing-Feng Zhang[9], Lin Li[2], Ming Fang[1#], Yong Li [2,3,5#]

1. Fisheries College, Jimei University, Xiamen, 361021, Fujian province, PR China
2. BGI Institute of Applied Agriculture, Shenzhen, 518120, PR China.
3. Shenzhen Engineering Laboratory for Genomics–Assisted Animal Breeding, BGI-Shenzhen, Shenzhen, 518083, China.
4. Animal Epidemic Disease Prevention and Control Center, Haixi Autonomous Prefecture of Qinghai Province, Delingha, Qinghai, 817099, China.
5. BGI Co. Ltd., Shenzhen, 518083, China
6. Haixi Agricultural Technology Extension Service Center, Haixi Autonomous Prefecture of Qinghai Province, Delingha, Qinghai, 817099, China.
7. Haixi Agricultural Products Quality Safety Inspection and Testing Center, Haixi Autonomous Prefecture of Qinghai Province, Delingha, Qinghai, 817099, China.
8. Faculty of Animal Science and Technology, Yunnan Agricultural University, Kunming, 650201, PR China
9. The Enterprises Key Laboratory of Tianjin Mutton Sheep Genetics and breeding, Tianjin Aoqun Animal Husbandry Pty.Ltd，Tianjin，301607,PR China

*Joint first author
# Corresponding author
Name: Yong Li, Ming Fang

E-mail: liyong3@genomics.cn, fangming618@126.com
ORCID iDs
Hu han [0000-0002-3946-2101]; Man-Man Yang [0000-0003-4328-056X]; Jiang Dan [0000-0002-6985-4579]; Xing-Ju Zhang [0000-0003-0717-2510]; Qiang Wei [0000-0003-2478-7760]; Tao Chen [0000-0002-0730-5670]; Wei-Dong Deng [0000-0002-9620-2082]; Ran Wang [0000-0003-1782-3356]; Ming Fang [0000-0003-3066-4855]; Yong Li [0000-0002-9406-5010];

Abstract

Cashmere traits were deployed for fiber yield and quality during the domestication of goats. However, the genetic alterations underlying cashmere trait selection are still unclear. We sequenced 120 Chinese native goats including two cashmere goat breeds and six ordinary goat breeds. The genome-wide selective sweep of cashmere goat and ordinary goat revealed a novel set of candidate genes as well as pathways, such as Nuclear factor kappa-B and Wnt Signaling pathways. Of them, *LHX2* regulating hair follicle development, was evident from the strongest selection signal when comparing the Uhumqin cashmere goat and ordinary goat. Interestingly, we identified a 582-bp deletion at 367 kb upstream of *LHX2* with higher frequency in cashmere goats and their ancient relatives. This mutation was probably arisen along the breeding procedures, and is putatively responsible for cashmere production and diameter, as revealed by association studies. Luciferase assay showed that the 582-bp sequence, which acts as an insulator, restrains the expression of *LHX2* by interfering its upstream enhancers. Our findings provide new insights into the genetic formation of cashmere and facilitate subsequent molecular breeding for cashmere goat improvement.

Keywords: *LHX2*, selective sweep, insulator, whole genome sequencing, cashmere trait

Introduction

Cashmere contributes high economic value to the textile industry. Cashmere is made from the processing of the underwool of goats that are grown in high and cold regions of the world, such as the Tibetan plateau and Mongolia. China is the largest cashmere producer globally, accounting for approximately 75% of the world's supply[1].

Improvement in the quantity and quality of cashmere is an important breeding goal in goat farming.

The cashmere goats with a long fine underwool, share a common ancestor with other ordinary goats that were domesticated from wild goat (bezoar) approximately 11,000 years ago in the Fertile Crescent of southwest Asia and adjacent areas[2, 3]. Almost all the breeds of goats can produce more or less cashmere fibers, but only the breeds with sufficiently fine hair are called cashmere goats (cashmere goats in China produce 250 g to 500g of fiber, while only 50g in ordinary goats)[4]. Previous research postulated that cashmere goats would not be domesticated locally, except several highland regions including Himalayas, Mongolia, and Kirghizia[5]. This kind of domestication leads to selecting more and more cashmere wool to meet textile demands. Therefore, in contrast to cashmere fiber formation, cashmere production was pursued by intense artificial selection pressure during the domestication of cashmere goats[6-8].

Previous transcriptome studies in cashmere goats have already discovered many important pathways that are involved in cashmere fiber development, including *WNT, FGF5, BMP, TGF-β, NOTCH, SHH,* and some of them may play important roles in secondary hair follicle development, such as *LHX2*, *FGF5* and *TGF-β4*[9-15]. Meanwhile, some genes, such as *FGF5, SGK3, IGFBP7, OXTR, ROCK1, LHX2, FGF9, PRDM6* and *WNT2* were intensively selected in cashmere goats[6, 16, 17]. Recent studies have identified several causal genes for cashmere growth trait, including *FGF5* and *EDA2R*[7, 18]. *FGF5* is a regulator of the hair growth cycle, and the disruption of *FGF5* is associated with a long hair characteristic[19, 20]. Interestingly, the enhancer-absent *FGF5* was only found in domesticated goats rather than in the wild goats, indicating this causative mutation occurred in domestication process[7, 21]. However, more research is necessary to elucidate the genetic basis of cashmere fiber formation in goats after divergence from sheep.

Here, we generated genomic data from 42 cashmere goats and 78 ordinary goats in China and conducted comprehensive population genomic analyses. Integrated with goat genomes, we identified genetic footprints under artificial selection during goat

migration and domestication. When compared the ancient and wild goat, we also investigate the origin and biological function of causative mutation for cashmere fiber formation.

Results

Population sequencing and genetic diversity

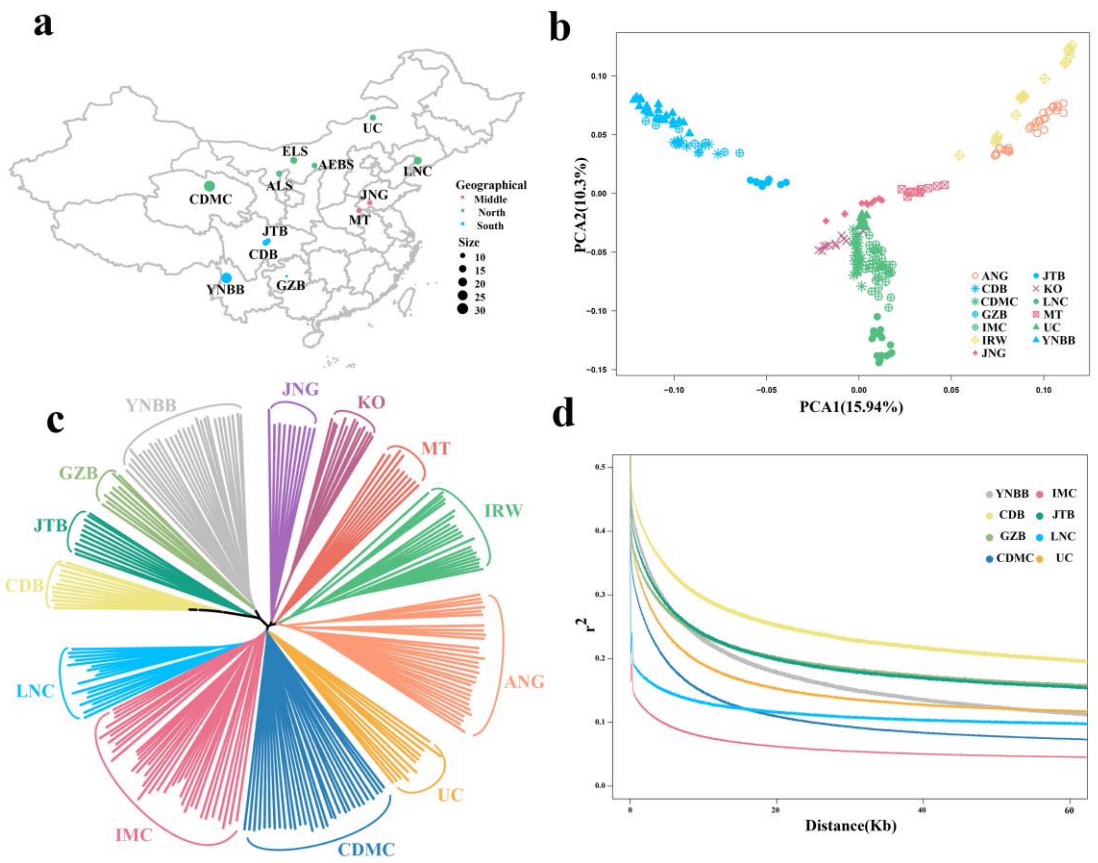

Fig. 1 Geographic distribution and population genetic analyses of 13 goat breeds.
(a) Geographical distribution of domestic goat breeds in China. Green, blue, red represents northern, southwest, middle regions, respectively. **(b)** Principal component (PC) plot of the first two components. The fraction of the variance explained is 15.94% and 10.3% for PC1 and PC2, respectively. **(c)** Neighbor-joining tree constructed using p-distances between different breeds, including LNC (Liaoning cashmere), IMC (Inner Mongolia cashmere), UC (Ujumqin cashmere), CDMC (Chaidamu cashmere), CDB (Chengdu brown), GZB (Guizhou Black), YNBB (Yunnan black bone), JTB (Jintang black), MT (Matou), JNG (Jining gray), KO (Korean), IRW (Iranian wild), and ANG (Angora). **(d)** Linkage disequilibrium decay of goat populations measured by r^2.

A total of 120 domestic goats representing eight geographically diverse breeds in China were selected for genome resequencing (Supplementary Table 1). These datasets were also analyzed together with the published whole-genome sequencing (WGS) datasets

of 116 individuals from five breeds (Supplementary Table 2 and 3). Among the ten domestic goat breeds, Liaoning cashmere goat (LNC), Inner Mongolia cashmere goat (IMC), Ujumqin cashmere goat (UC) and Chaidamu cashmere goat (CDMC) are the cashmere goat breeds located in the north of China; Chengdu brown goat (CDB), Guizhou Black goat (GZB), Yunnan black bone goat (YNBB) and Jintang black goat (JTB) in the southwest and Matou Goat (MT) and Jining gray goat (JNG) in the middle are non-cashmere goat breeds (Fig. 1a). The remaining goat breeds include Korea goat (KO), Iranian wild goat (IRW) and Angora goat (ANG) from Korea, Iran and France, respectively.

A total of 8448.92 Gb paired-end DNA sequence data were obtained from 236 goats (Supplementary Table 1). All goats were sequenced with an average 12-fold depth of the genome (4.17~29.9×) and the average genome coverage of 97.82% (Supplementary Tables 1-3). 99.2% reads were mapped to the latest goat reference genome ARS1(GCA_001704415.1) (Supplementary Tables 1-3). We totally detected 13,069,924 high-quality single nucleotide polymorphisms (SNPs), with 0.605% (0.229 million) located in exonic regions (Supplementary Table s1). The average transition-to-transversion (Ti/Tv) ratio was 2.46 for all goat samples, which indicated relatively low potential random sequencing errors (Supplementary Table s1).

The principal component analysis (PCA) of the 236 goats revealed genetically distinct clusters according to their geographic locations. The clustering results of the northern cashmere goat populations (IMC, LNC, UC and CDMC) and ordinary goat populations (YNBB, JTB, GZB and CDB) were clearly separated (Fig. 1b). JNG and MT were divided into subgroups between cashmere goats and the ordinary southwest goats. Samples of Iranian wild goats (IRW) and Angora goats (ANG) (France, South Africa, and Madagascar) were significantly different from those of Chinese native goats (Fig. 1b). This result was confirmed by the phylogenetic tree using the same SNPs (Fig. 1c). In the analysis of linkage disequilibrium (LD), four ordinary goat breeds from southwest China (YNBB, JTB, GZB and CDB) showed an overall slower decay rate and a higher level of LD than the cashmere breeds from northern China (IMC, LNC, UC and CDMC) (Fig. 1d).

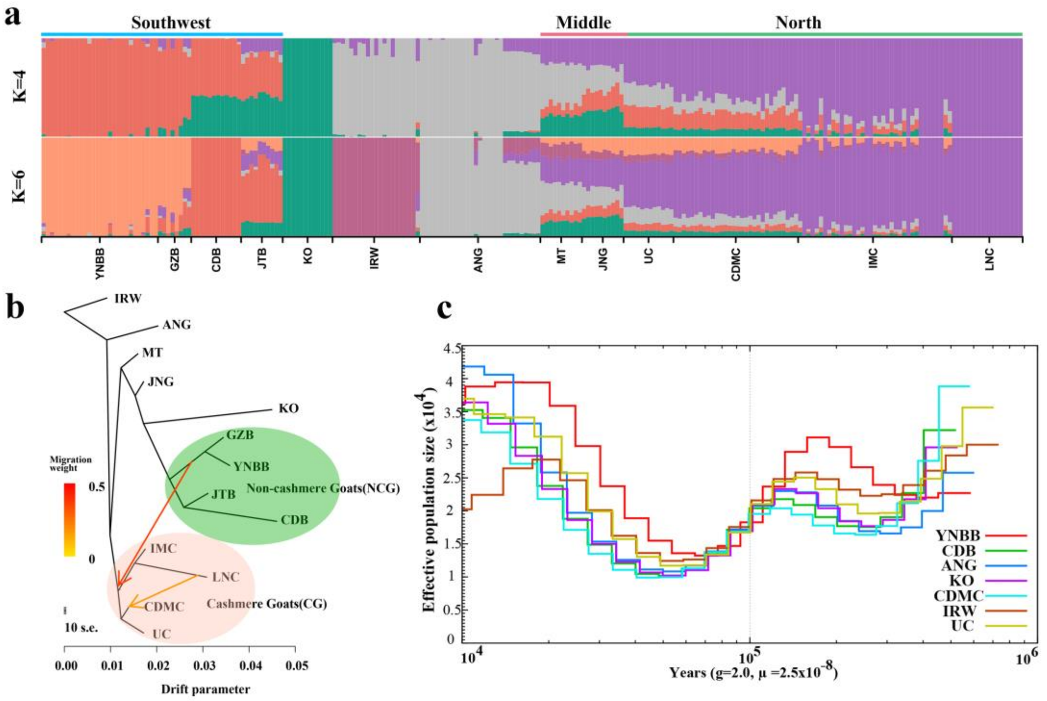

Fig. 2 Population structure, gene flows, and effective population size of goat populations. (a) Model-based population assignment with ADMIXTURE analysis for K = 4 and 6, respectively. The population names are at the bottom of the figure, and the geographic locations are at the top. **(b)** Phylogenetic network of the inferred relationships among the 13 native breeds with two inter-group migration edges being identified. The branch length is proportional to the drift of each population. IRW (Iranian wild goat) was used as the outgroup to root the tree. The colored regions in the phylogenetic tree represent two inferred genetic groups. Arrows indicate migration events, and a spectrum of heat colors indicates the migration weights of the migration events. **(c)** Pairwise sequentially Markovian coalescent analysis for the representative individuals sequenced at a high read coverage, exhibiting inferred variations in Ne over the last 10^6 years. g (generation time) = 2 years; μ (neutral mutation rate per generation) = 2.5×10^{-8}.

In the STRUCTURE analysis, When K = 4, we observed five separate clusters: IRW and ANG in west Asia, YNBB, GZB, JTB and CDB in southwest China; cashmere goat in north China; MT and JNG in middle east China; and Korean goats in south Korea. At K = 6, goats in the southwest China further split into two geographic

subgroups: the Yunnan-Kweichow Plateau group including YNBB and GZB goats, and the Chengdu Plain group including CDM and JTB goats. Two west Asian goats (IRW and ANG) were also separated (Fig. 2a, Supplementary Fig. 1). Some cashmere goats showed evidence of admixture, which may be attributable to shared ancestral polymorphism and recent introgression events by crossbreeding with neighboring domestic goats. Interestingly, the genetic structure of the Chinese goat population revealed that UC and LNC goats represent two different types, and other cashmere goats are a mixture of these two types (Supplementary Fig. 3).

In TreeMix analysis, we found that cashmere goats and ordinary goats clustered into two groups (Fig. 2b). We observed two migration edges among clusters from LNC to CDMC and from YNBB and GZB to the cashmere goat (Fig. 2b), which can be explained by the fact that CDMC goats were crossbred between goats of the Qinghai-Tibet Plateau and LNC.

Seven high-coverage samples were chosen to infer the effective population size (Ne) over the last 10^6 years using the pairwise sequential Markovian coalescent (PSMC) method[22]. PSMC analysis suggested that the goats suffered at least two bottlenecks (approximately 7000 and 18000 years ago), which resulted in a severe reduction in the effective population size (Fig. 2c). Interestingly, the results of the PSMC analysis of goats were like the demographic history of the sheep[23].

Genome-Wide Selective Sweeps

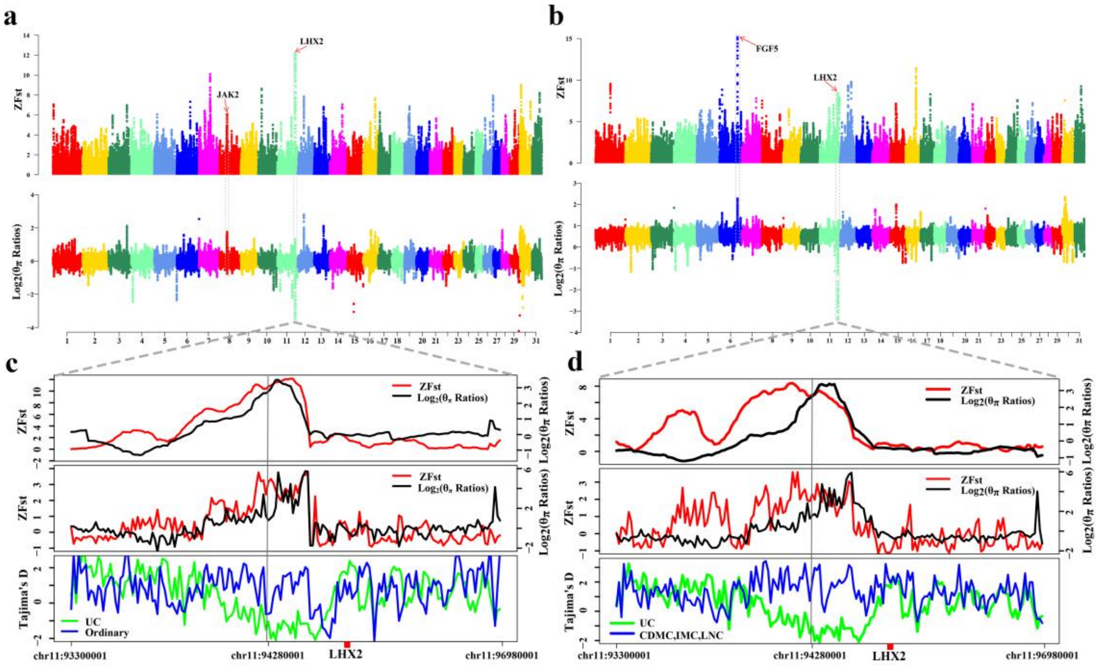

Fig. 3 Genomic regions with selection sweep signals in domestic goats. (a) Manhattan plot of the genome-wide distribution of pairwise ZFst and $\log_2(\theta_\pi$ Ratios) between UC and ordinary goats (YNBB, GZB, JTB and CDB) using a 150 kb window size and a 10 kb step size. **(b)** Manhattan plot of the genome-wide distribution of pairwise ZFst and $\log_2(\theta_\pi$ Ratios) between UC and other cashmere goats (CDMC, LNC and IMC) using a 150 kb window size and a 10 kb step size. **(c)** Zoom of the peak signal on chromosome 11 between UC and ordinary goats (YNBB, GZB, JTB and CDB). $\text{Log}_2(\theta_\pi$ Ratios), ZFst values, and Tajima's D values around the *LHX2* region (Fig. 3a) using a nonoverlapping 10 kb sliding window. The black and red lines represent $\log_2(\theta_\pi$ Ratios) and ZFst values, respectively; the blue and green lines represent the ordinary goats and UC goats, respectively. **(d)** Zoom of the peak signal on chromosome 11 between UC and other cashmere goats (CDMC, LNC and IMC). $\text{Log}_2(\theta_\pi$ Ratios), ZFst values, and Tajima's D values around the *LHX2* region (Fig. 3b) using a nonoverlapping 10 kb sliding window. The blue and green lines represent the other cashmere goats and UC goats, respectively.

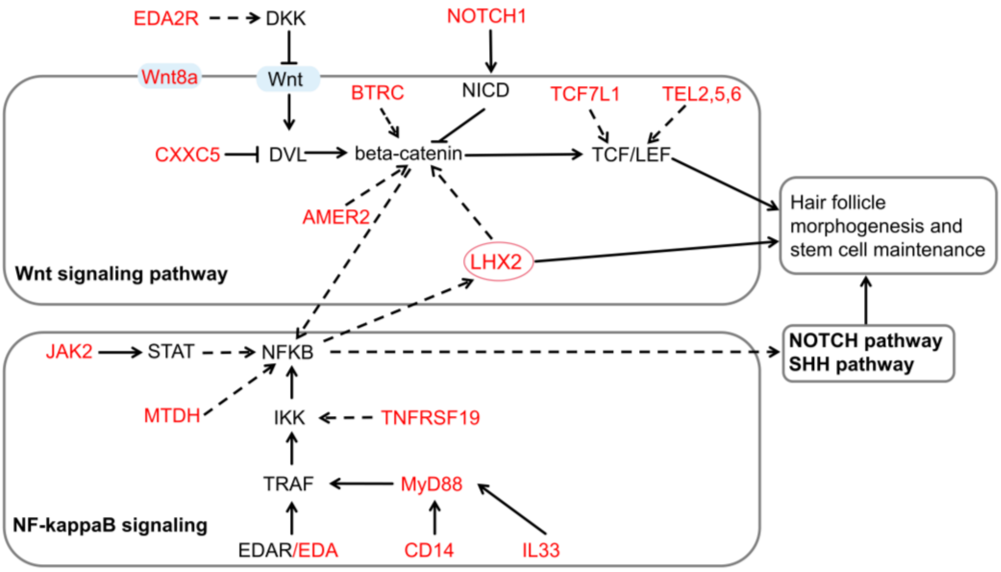

Fig. 4 Schematic mechanisms of signaling pathways involved in cashmere fiber development. The names of the KEGG pathways are shown in bold. The candidate genes positively selected in the two methods of Fst and θπ ratio tests are shown in red. The solid block arrows represent direct effect, and the dashed black arrows indicate an indirect effect. The blunt head arrow indicates an inhibition effect.

The results of the genetic structure analysis showed that the two cashmere goats (UC and CDMC) had apparent admixture with some other ordinary breeds. In contrast to CDMC bred through crossbreeding between Qinghai native goats and LNC, UC is originally from the Ujumuqin region and is continually selected for cashmere traits. Therefore, the distinct genetic background of UC raised the question of whether there is a different selective sweep region for the cashmere trait. We then performed a selective sweep analysis with the 12 UC and 58 ordinary goat genome sequences by estimating pairwise genetic differentiation (Fst) and nucleotide diversity differences (θπ) in 150 kb sliding windows along the genome. Using the top 1% of ZFst values and $\log_2(\theta_\pi$ Ratios) cutoffs (ZFst > 4.01, the absolute value of $\log_2(\theta_\pi$ Ratios) > 0.85), we identified 201 candidate genes associated with cashmere traits (Fig. 3a, Supplementary Table s2). We found that the most prominent ZFst signature was on chromosome 11 (Fig. 3a), spanning ~1,100 kb region (93.6–94.7 Mb). This signature was also supported by the ZFst, $\log_2(\theta_\pi$ Ratios), and Tajima's D statistics (Fig. 3c). However, we did not identify the selection signal harboring *FGF5*, which has been identified in previous

reports[7]. We next used UC and other cashmere goats to perform selective sweep analysis and identified 263 genes corresponding to selective sweeps (Fig. 3b and d, Supplementary Table s3). Interestingly, both selection signals on chromosome 6 and *FGF5* were simultaneously identified in these populations. Among all the candidate genes, eight (*EDA, MyD88, CD14, IL33, TNFRSF19, LHX2, AR, STK3* and *JAK2*) were located in the NF-kappaB signaling pathway and eleven (*TCF7L1*, *WNT8B*, *BTRC*, *AMER2*, *TEL2*, *TEL5*, *TEL6*, *LHX2*, *CXXC5*, *NOTCH1* and *VCAN*) were found in the Wnt/ß-catenin signaling pathway (Fig. 4, Supplementary Fig. 6, Supplementary Table s4 and s5). These signaling pathways play a central role in regulating hair follicle morphogenesis, stem cell differentiation and hair cycle[24, 25]. The strongest selective sweep localized on chromosome 11, harbored DENN domain-containing protein 1A (*DENND1A*), Crumbs cell polarity complex component 2 (*CRB2*) and LIM homeobox 2 (*LHX2*), in which *DENND1A* and *CRB2* are associated with polycystic ovary syndrome (PCOS) and maintenance of apicobasal polarity in retinal pigment epithelium, respectively, but without evidence in hair development [26, 27]; while another gene *LHX2* near *DENND1A*, plays important role in hair follicle development.

To confirm the reliability of our findings, we further used other cashmere goats (LNC, IMC and CDMC) and ordinary goats to perform sweep selection analysis. The results showed that all previously-detected functional genes for cashmere traits including *FGF5, EDA2R* and *STIM1*, were re-identified (Supplementary Fig. 7)[7, 21]. In addition to the cashmere trait, we also investigated the selection sweep for coat colors and confirmed previously reported genes *KIT*, *KITG*, *IRF4* and *ASIP* for coat color using different goat breeds (Supplementary Fig. 8, Supplementary Fig. 9 and Supplementary Fig. 10). Furthermore, we also identified previously reported 100 kb copy number variants compassing *KIT* (chr6:70,859,258-70,959,918) and ~154 kb copy number variants within *ASIP* (chr13:63,226,824-63,381,501) with single base-pair resolution (Supplementary Fig. 11 and Supplementary Fig. 12).

Plausible Causative Mutation near *LHX2*

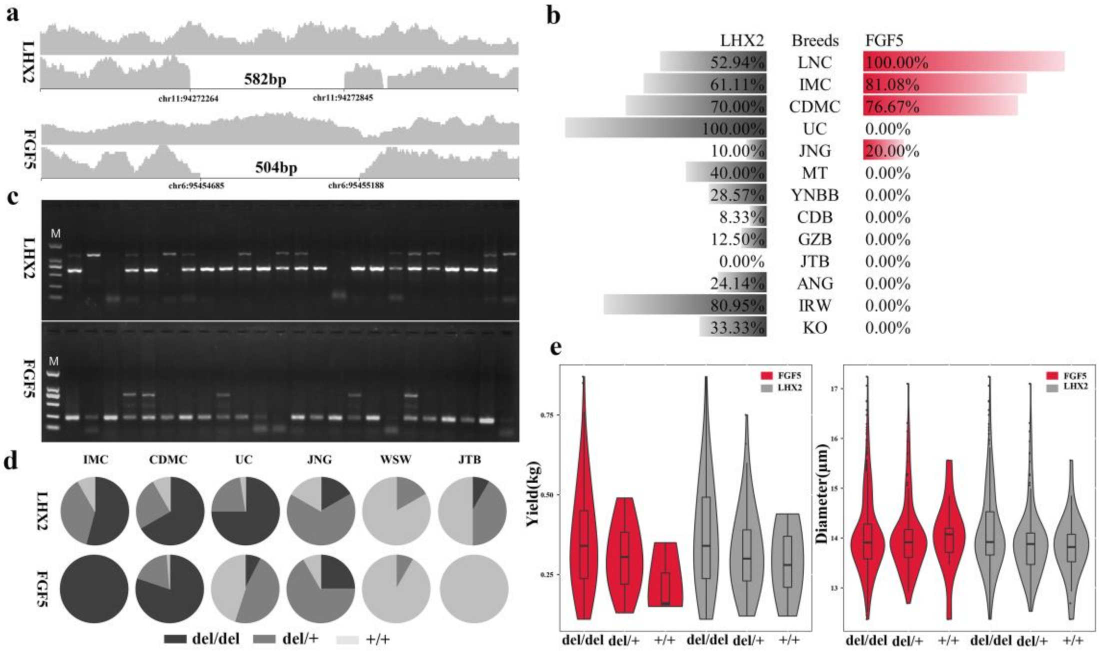

Fig. 5 The *LHX2* and *FGF5* deletions in different goat breeds and their association with cashmere traits.

(a) The identified 582 bp deletion near *LHX2* and the previously reported 504 bp deletion near *FGF5* based on read coverage. **(b)** Homozygous genotype frequency of the two deletions determined by IGV viewer **(c)** PCR amplification of the two deletion variants. **(d)** Distribution of the 582 bp deletion near *LHX2* and the 504 bp deletion of *FGF5* genotypes. del/del, deletion/deletion; del/+, deletion/wild type; +/+, wild type/wild type. WSW (Wushan White goat from southwest China). **(e)** The association of the 582 bp deletion near *LHX2* and 504 bp deletion of *FGF5* with cashmere yield and diameter in the CDMC goat population.

We inspected all variants within exons to identify the potential causal mutation around the *DENND1A*-*LHX2* locus; however, no coding variants were found (Supplementary Table s6). Strikingly, a 582 bp (chr11: 94,272,264-94,272,845 bp) deletion was identified in the 13th intron of *DENND1A* and 367 kb upstream of *LHX2* with Integrative Genomics Viewer (IGV) (Fig. 5a). We tried to genotype of the deletion variant of everyone in different populations, but it is not obvious to distinguish heterozygous type from homozygous wide type due to limited read counts with IGV viewer, but it is quite confident to identify homozygous wide type. We then compared the frequencies of the homozygous deletion variant in different goat populations. The

analysis results showed that the 582 bp deletion near *LHX2*(named 582del) had a higher frequency (52.9–100 %) in cashmere goats as compared 0.00%-40.00% in ordinary goats (Fig. 5b and Supplementary Table 5). We also identified a 504 bp deletion (chr6: 95,454,685-95,455,188 bp) in the *FGF5* locus (named 504del), which is consistent with previous reports (Fig. 5a) [7]. However, the frequency of the 582del in the ordinary goats was much higher than that of the 504del (Fig. 5b and Supplementary Table 6). More interestingly, we found that the 582del has a high frequency in the IRW population (80.9 %), while the 504del was absent, which is also consistent with previous research[7]. Since we could not accurately distinguish the heterozygous from wild genotypes using IGV viewer, we designed PCR primers for detecting the heterozygosity of the two deletions in several goat populations. It showed that the frequency of homozygosity was consistent with that by IGV viewer (Fig. 5c, Fig. 5d, Supplementary Table 7 and Supplementary Table 8), but some breeds had a higher frequency of heterozygosity, such as in UC (504del+/-: 47.5 %) and JNG goats (504del+/-: 66.7 %; 582del+/-: 66.7 %). Finally, we detected the 582del in several ancient goat samples (7/21) (Supplementary Table 9) and ibex samples (1/3) (Supplementary Fig. 13 and Supplementary Fig. 14), indicating that the 582del is an older mutation that occurred much earlier than the 504del. In addition, we compared the genotypes of the target genomic region for cashmere and ordinary goats. The result showed that the genotype patterns of cashmere goats were highly like that of wild goats, but different from that of ordinary goats (Supplementary Fig. 15).

To further evaluate whether these two deletion variants were related to cashmere traits, we selected 235 CDMC goats with cashmere yield (Supplementary Fig. 16, Supplementary Table s10) and 581 CDMC goats with fiber diameter records (Supplementary Fig. 17, Supplementary Table s11) for association analysis. The association results showed that the 582del and the 504del was significantly associated with cashmere production (*P* value = 0.0061 and *P* value = 0.0113 for the 582del and the 504del, respectively). The 582del was also significantly associated with fiber diameter (*P* value = 2.74e-04), but the 504del was not (*P* value =0.348) (Fig. 5e). Interestingly, the interactions effect of the 582del and 504del were significantly

associated with fiber diameter (*P* value = 6.1e-06), suggesting crosstalk between the two genes.

Biological Function of the 582 bp Deletion

Analysis of the 582del region using the BLAST program revealed that it is not a highly conserved element but was found in the ancient genomes of primate and ungulate species (Fig. 6a). By checking the UCSC Genome Browser (https://genome.ucsc.edu/), we found many cis-regulatory elements and H3K27Ac marks (often enriched in enhancer regions) upstream of this deleted region (Fig. 6b). Interestingly, we identified many S/MAR sequences in the 582bp sequence and a CTCF-binding site as well as two distal enhancer-like signatures which were close to it (Supplementary sequence 1 and 2). Using previously reported RNA-seq data[28], we found that the expression of *LHX2* in the different fetal stages of cashmere goats exhibited an up-regulation pattern during hair follicle development (Fig. 6c, Supplementary Fig. 18). Furthermore, some functional enhancers of *LHX2* were also identified in the genome regulatory blocks of the *CRB2-LHX2* loci[29]. This evidence suggests that the 582-bp sequence may function as an insulator to block the *LHX2* enhancer function.

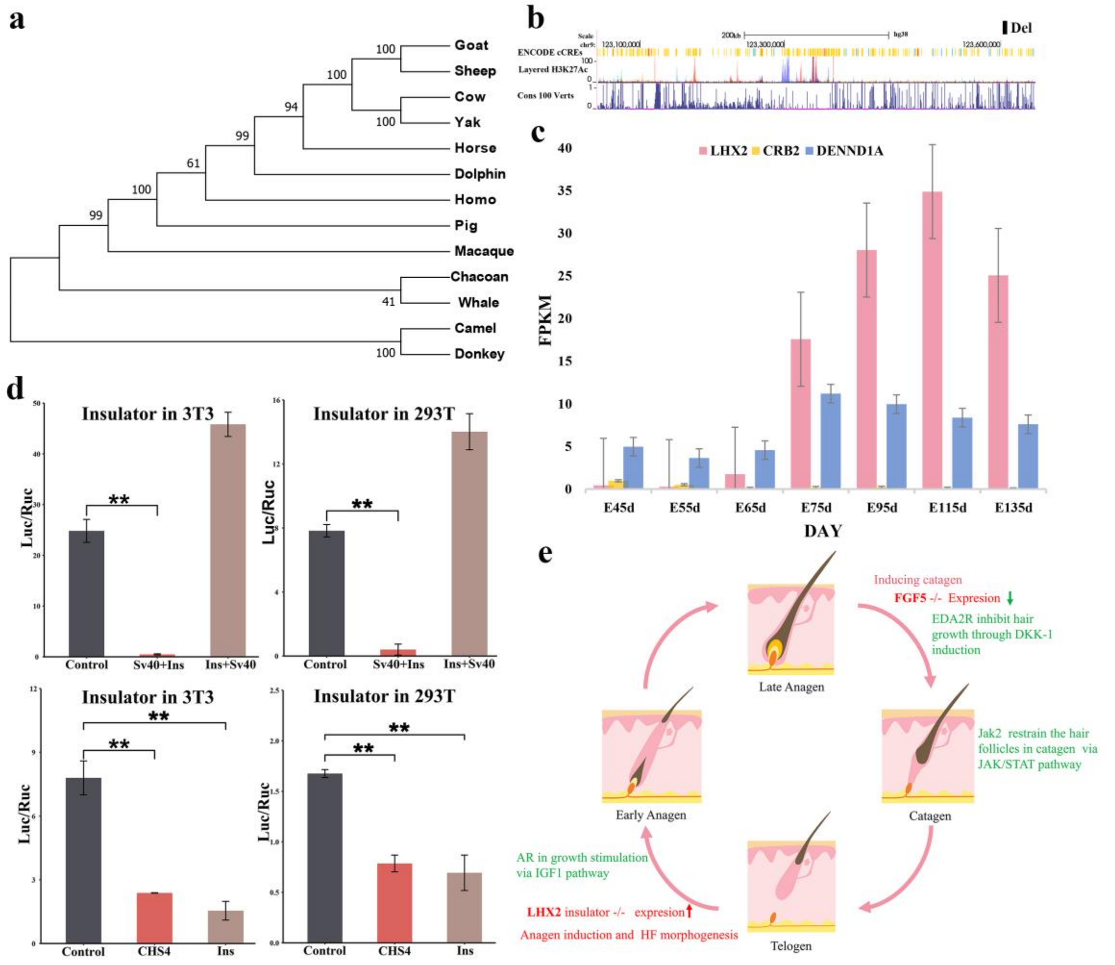

Fig. 6 Effects of *LHX2* deletion sequence on reporter gene expression. (a) Phylogenetic tree of 13 primates and ungulates for the detection of the 582 bp deleted sequence. **(b)** Regulatory elements, epigenomic signals, and conservation scores on selective sweep region in chromosome 11. The black box represents the deletion variant location of *LHX2*. **(c)** The expression of *LHX2*, *CRB2* and *DENND1A* in different stages of prenatal skin in cashmere goats. Boxes of different colors are used to represent genes. The expression data is downloaded from a previous report[28]. **(d)** A dual-luciferase assay using NIH3T3 cell and 293T cell shows the *LHX2* upstream deletion sequence blocks the activity of luciferase. Data are shown as the mean ± standard error. The P-value was calculated using Student's *t*-test. **(e)** The molecular mechanisms of cashmere fiber development. In detail, the deletion of the *LHX2* insulator increases the expression of *LHX2* and promotes the cashmere fiber growth at the anagen stage, and *AR* could participate in hair growth at the anagen stage through the *IGF1* pathway[30], while the deletion of the 504 bp enhancer reduces the expression of *FGF5*, thereby inhibiting growth regression; *JAK2* and *EDA2R* may also participate in this stage via the JAK/STAT and Wnt pathways, respectively.

To confirm the insulator function of this sequence, we synthesized a 551 bp DNA fragment (named Ins, Supplementary sequence 2) and subsequently inserted it downstream and upstream of the SV40 promoter in the pGL3 plasmid (Supplementary Fig. 17). The Ins vectors with the Renilla luciferase vector, phRL-TK, were transiently co-transfected into human 293T cells and mouse 3T3 cells. After 48 h, the firefly and Renilla luciferase activities of the lysate were measured, and the ratio of firefly luciferase activity to Renilla luciferase activity was calculated for each sample. Our data showed that the Ins fragment decreased the expression of firefly luciferase by more than 90 % in the downstream group of both cell types and increased expression in the upstream group (Fig. 6d). In addition, we inserted the Ins downstream of the SV40 enhancer in the pGL3 plasmid (Supplementary Fig. 19) and chose the well-known insulator cSH4 as a control to quantify the efficiency of the Ins. Our data showed that the Ins fragment decreased the expression of firefly luciferase by approximately 50 % in human 293T cells, which was comparable to that of cSH4 (Fig. 6d). In addition, a similar result of the Ins activity was validated in the mouse 3T3 cells but decreased more sharply than the cSH4 group (Fig. 6d). These results suggested that the 551 bp DNA fragment played an enhancer-blocking function in mammary cells.

To describe the function mode of these selective sweep genes on the development cycle of cashmere, we downloaded and analyzed the transcriptome data of skin tissue during the prenatal stages and one-year cashmere cycle. The transcriptome data showed that *AR* and *EDA2R* were not expressed in embryonic cashmere goat skin, and *JAK2* gene expression was opposite to that of *LHX2* (data from Wu[28], Supplementary Fig. 20). Furthermore, *JAK2*, *EDA2R* and *FGF5* exhibited similar expression patterns in the skin transcriptome data of different months of the one-year cycle, while *LHX2* and *AR* had similar expression patterns (PRJNA470971) (Supplementary Fig. 20 and 21). It is known that cashmere fiber growth has three different periods in one year: a growth period (March–September), a regression period (September–December), and a resting period (December–March)[31]. Therefore, the deletion of the *LHX2* insulator increases the expression of *LHX2* and promotes cashmere fiber growth at the anagen stage, while deletion of the

FGF5 enhancer reduces the expression of *FGF5*, inhibiting the regression. Therefore, the 582bp deletion disrupts the insulator and increases the expression of *LHX2*, and promotes cashmere fiber growth at the anagen stage, while deletion of the *FGF5* enhancer reduces the expression of *FGF5*, and thus inhibits the regression of the hair follicle. Other candidate genes may affect cashmere fiber growth through uninvestigated regulation modes. Overall, we propose a possible molecular model for cashmere fiber formation: *LHX2* and *AR* may be involved in maintaining hair growth at the anagen stage, whereas *JAK2*, *EDA2R* and *FGF5* function in the destructive phase (catagen) through the highest expression in September (Fig. 6e).

Discussion

In this study, we resequenced the genomes of 42 cashmere goats and 78 ordinary goats. Population analyses revealed that goats in southwest China are different in the genome from goats in West Asia and North China, which is consistent with a recent study[7]. In the present study, we observed a genetic introgression from the LNC breed into the CDMC breed, which was confirmed by the origin and breed practice of CDMC (using Chaidamu goats as female parents, and LNC as male parents to breed new cashmere goats, named CDMC)[32]. In addition, the introgression from the Yunnan-Kweichow Plateau into the cashmere goat breeds suggests that goats from the Qinghai-Tibet Plateau and Mongolian Plateau, as well as from the Yunnan-Kweichow Plateau, may have a common ancestor or have a genetic admixture in their early years. Similar introgression results have also been reported in sheep populations from these regions[23]. In contrast, Angora goats have a signature of genetic admixture with some Chinese goats (Fig. 2a), which has also been reported previously[4]. In addition, although KO has large genome differences with other goat breeds[33], a clear signature of genetic admixture between KO and JT, JNG, and MT was observed, which may be related to the historical human migration between China and Korea. Finally, we observed CDMC and IMC goats presenting a genetic admixture of UC and LNC. LNC was bred in the 1980s from six counties in the eastern mountainous area of Liaoning province in China, famous for its high cashmere yield. UC was

originally bred from Ujumuqin white goat in the region of Ujumuqin grassland for selecting the cashmere trait in 1994. Unlike LNC, UC has a closer genetic distance to goats in the middle region of China (Fig. 2a). These results indicate that UC may have a more unique genetic background than other cashmere goats.

By performing a whole-genome selection scan, we discovered a novel selective sweep region on chromosome 11 that appears to have undergone extremely strong selection in UC. This novel selective sweep region is located in a conserved linkage block, containing three genes: *DENND1A*, *CRB2* and *LHX2*[29]. Among these genes, *LHX2* functions as a transcriptional activator in hair follicle stem cells and is an essential positive regulator of hair formation[34, 35]. Furthermore, the expression of *LHX2* was upregulated during hair follicle differentiation, and the expression abundance was constant throughout the development cycle in secondary hair follicles of cashmere goats[28, 31]. In contrast, *DENND1A* and *CRB2*, which function in endocytic trafficking to mediate the recycling of selective cargos and early embryonic development, respectively[36, 37], were expressed lowly in the fetal stage of goat skin and showed very low expression during the cycle of adult cashmere fiber growth (Fig. 6c). These results indicate that *LHX2* is a functional gene in this selective sweep region.

We then reported an upstream deletion of *LHX2*, which carries a potential cis-regulatory insulator region (Fig. 6d). In contrast to the deletion allele of *FGF5*[7], the deletion allele of *LHX2* was found in all goats investigated, including wild and domesticated goats, but allele frequency was higher in northern and wild goats than in goats in other areas (Fig. 5b and Fig. 5d). Notably, this deletion was also found in the ancient goat and ibex samples (Supplementary Figs. 13 and 14). Ibex goats may also have dense underwool, like cashmere goats[4]. These results indicate that the deletion allele of *LHX2* occurred in the pre-domestication stage of goats and might be traced back to the early stage of goat speciation to cold climate adaptation. Further, we did not detect the 582 bp deletion in the same region of sheep, but we cannot rule out the possibility that we tested too few sheep (Supplementary Fig. 22). In contrast, the deletion allele of *FGF5* was not found in the Iranian Wild Goat (0/21), Capra

falconeri and Capra sibirica (0/6) as well as ancient goats (0/21) (Supplementary Table 6), indicating that this deletion is most likely to occur during the domestication stage of goats to obtain higher cashmere production. Overall, it is possible that the deletion allele of *LHX2* is acquired in the wild goats at a very early stage to adapt to the cold environment and reaches a higher frequency in the north than in the south, while the *FGF5* enhancer deletion may have occurred during artificial selection or secondary domestication within the last 450 years[7] and fixed during the long-term artificial selection for cashmere trait. However, the origin and spread of cashmere-related mutations is just our inference based on available data and there may be other possibilities worth further investigating.

Multiple sequence alignment revealed that the selective sweep region contains many CTCF binding sites and other cis-regulatory elements, as well as the H3K27ac epigenetic mark, located upstream of the 582bp deletion. CTCF is a highly conserved zinc finger protein, required for insulator function in mammals[38], while H3K27ac is a marker for active enhancers and a great indicator of enhancer activity[39], suggesting that the selective sweep region contains some hair-related transcription regulatory elements. Our biological function experiments showed that the 582bp deletion region contains an insulator for blocking the upstream enhancer role with higher efficiency in skin-related cells (NIH3T3 cells) than cSH4. An insulator is a long-range regulatory element, which protect an expressing gene from its surroundings through two ways: blocking the action of a distal enhancer on a promoter and acting as“barriers” that prevent the advance of nearby condensed chromatin[38] . Previous reports described those four of the eight conserved noncoding elements (CNEs), approximately 222 kb to 619 kb upstream of human *LHX2*, functioned as tissue-specific enhancers in specific regions of the central nervous system and the dorsal root ganglia (DRG), recapitulating partial and overlapping expression patterns of *LHX2* and *CRB2* genes[29]. In our research, we found that the insulator is situated approximately 367 kb upstream of goat *LHX2* in the 13th intron of *DENND1A*. Therefore, we speculate that this insulator can regulate

the expression of the *LHX2* gene by blocking the upstream enhancers, and the deletion of the insulator can increase the expression of the *LHX2* gene, thus increasing cashmere production. Subsequently, association analysis confirmed the significant relationship between the deletion genotype and cashmere traits, including cashmere production and fiber diameter (Fig. 5e). The interaction effect of the two deletion variants significantly affected the diameter of the cashmere fiber, indicating that the two genes have a synergistic effect on cashmere fiber development. *LHX2* is primarily expressed by precursor cells outside of the bulge region at the stage of anagen and becomes undetectable when the HFs enter telogen[35]. FGF5 is highly expressed during the late anagen phase and promotes the transition from anagen to catagen. The inactivating mutations of *FGF5* display a long-haired phenotype though extending anagen phase[40]. Therefore, the *LHX2* would be expressed strongly and continuously at the prolonged stage of anagen when an individual contains both deletion variants. Furthermore, the upregulated expression of the *LHX2* gene maintains the function of hair follicle stem cells and activates genes related to hair fiber structure and function[41]. Alternatively, hair matrix cells may have a finite capacity for proliferation and an improvement for hair follicle structure and function due to a lack of the enhancer of *FGF5* and the insulator of *LHX2* together. Overall, the down-regulated expression of *FGF5* and the up-regulated expression of *LHX2* gene may form an interaction effect in the same period, thus significantly affecting the development of cashmere fiber. In addition to *LHX2* and *FGF5*, we also identified a few positively selected genes related to cashmere traits, four genes (*AR*, *JAK2*, *EDA2R* and *STK3*) affecting I-kappaB kinase/NF-kappaB signaling, and five genes (*NOTCH1*, *TCF7L1*, *AR*, *VCAN* and *WNT8B*) influencing the canonical WNT signaling pathway (Supplementary Table s5). We found that *NOTCH1*, *TCF7L1* and *STK3* exhibited similar seasonal expression patterns with *JAK2*, *FGF5* and *EDA2R* in the skin, but *VCAN* and *WNT8B* genes showed low expression (Supplementary Fig. 23; PRJNA470971). NF-kappaB and WNT signaling pathways play a central role in hair follicle development and regeneration[42-44]. Interestingly, *LHX2* is also regulated by NF-κB signaling to promote primary HF morphogenesis[45]. *EDA2R* is

a divergent gene between cashmere goats (excluding UC goats) and ordinary goats, consistent with previous research[7]. *EDA2R* is highly expressed in the late anagen phase and may inhibit hair growth by inducing *DKK-1* expression[46]. Furthermore, the *AR* gene and *JAK2* are novel genes identified in cashmere traits. *JAK2* can restrain the hair follicles in catagen via the JAK/STAT pathway with up-regulation in catagen and telogen stages[47, 48]. In contrast, AR has paradoxically different effects on hair follicles[49]. In cashmere goats, *AR* is expressed in the early anagen stage and may stimulate cashmere growth via the *IGF-1* pathway[50]. These positively selected genes may be involved in cashmere trait formation through the model described in Fig. 6e.

Conclusions

Through population genomics and selective sweep analyses of cashmere and ordinary goats, we identified a novel causative mutation upstream of *LHX2* that functions as an insulator to block the enhancer of *LHX2*. In contrast to enhancer deletion of *FGF5*, this insulator deletion also retained high allele frequency in wild goats, ancient goats, and ibex. We also found that the insulator deletion was associated with two cashmere traits in the CDMC goat population. The positively selected genes were enriched in the NF-κB and Wnt pathways, which play a central role in hair follicle development. This study not only provides the first evidence of how cashmere fiber growth could be regulated during the evolution of goats but also offers valuable molecular markers for the genetic improvement of cashmere traits in goats.

MATERIALS AND METHODS

Samples Collection

We collected 120 domestic goats (*C. hircus*), representing eight geographically diverse breeds in China, which include 30 CDMC from Chaidamu City of Qinghai Province; 11 UC from Ujimqin Banner of Inner Mongolia Province; 8 GZB from Guiyang City of Guizhou Province; 11 YNBB from Lanping County of Yunnan Province; 10 MT from Shangqiu City of Henan Province; 10 JNG from Jining City of Shandong Province;

10 JTB from Jintang County of Sichuan Province; 12 CDB from Chengdu City of Sichuan Province. Details of the samples used in this study are provided in Supplementary Table 1. All animal procedures were approved by the ethics and biological safety review committee of BGI (NO. FT 18041), and were carried out in accordance with the approved guidelines.

DNA Extraction and Sequencing to further evaluate whether these two deletion variants were related to cashmere

Ear or blood tissues of CDMC, UC, JT, GZB, JNG, YNBB, CDB and MT were collected on-site and stored in an alcohol sampling tube or blood collection tube. A tissue DNA extraction kit was used to extract the genomic DNA from the samples, electrophoresis was used for integrity detection, and Qubit was used for concentration determination. Library construction for resequencing was performed with 1-3 μg of genomic DNA using standard library preparation protocols and insert sizes from 200–400 base pairs (bp). All 120 goats were sequenced on the BGISEQ-500 platforms (BGISEQ-500, RRID:SCR_017979) with PE100 (CDMC, UC, JT, YNBB, CDB, MT) and on the Illumina HiSeq2500 platforms (Illumina HiSeq 2500 System, RRID:SCR_016383) with PE150 (GZB, JNG). Furthermore we downloaded published genomic data of modern, wild and ancient goats from NCBI databases (Supplementary Table 2, Supplementary Table 2 and Supplementary Table 9).

Read Alignment and Variant Calling

We obtained raw data from a total of 236 goat samples, including 120 sequenced and 116 downloaded. All raw data were first filtered and trimmed using SOAPnuke (SOAPnuke, RRID:SCR_015025)v1.5.0 [51], if any of the following criteria were met: (1) reads containing adapter and poly-N; (2) reads whose low-quality base ratio (base quality less than or equal to 5) is more than 50%; (3) reads whose unknown base ('N' base) ratio is more than 10%. Clean reads from all individuals were aligned to the goat reference genome ARS1 [52] by the Burrows-Wheeler Aligner (BWA version 0.7.12, RRID:SCR_010910). SAMtools[53](RRID:SCR_002105) was used to convert the file format from SAM to BAM and filter the unmapped and non-unique reads. Picard (Picard, RRID:SCR_006525) v.1.54was used to sort the BAM files and remove

potential PCR duplications if multiple read pairs had identical external coordinates. GATK (GATK, RRID:SCR_001876) v4.0.11 tools were used in the whole process of variant calling[54]. After mapping, the “HaplotypeCaller”, “CombineGVCFs” and “GenotypeGVCFs” in GATK4 were used to detect SNPs and Indels with default parameters. The output VCF File was then screened for SNPs using "SelectVariants function" of GATK4. SNPs and Indels were separated using the GATK tool “SelectVariants” and subjected to rigorous processing to exclude false positives. To obtain high-quality SNPs, we carried out SNP filtering at two stages. SNPs exclusion criteria[55] were as follows: (1) hard filtration with parameter “QD < 2.0 || ReadPosRankSum < -8.0 || FS > 60.0 || MQ < 40.0 || SOR > 3.0 || MQRankSum < -12.5 || QUAL < 30;” (2) “--max-missing 0.9 --maf 0.05 --min-alleles 2 --max-alleles 2.” Finally, ~13 million high-quality SNPs were obtained for further analysis.

Population Structure Analysis

We used 12,861,877 high-quality (autosomal) SNPs for PCA analysis. PLINK (version 1.9, RRID:SCR_001757)[56] was used to calculate the principal components, using function "--vcf vcf --out pca –pca --chr-set 29 --allow-extra-chr".

An individual-based NJ tree was constructed for the 236 goats based on the p-distance, with one outgroup (IRW, Iranian wild goat) using the software VCF2Dis (VCF2Dis RRID:SCR_022513) v1.09 and PHYLIP (PHYLIP, RRID:SCR_006244) v.3.69[57]. The Linux command line of converting to a matrix is "VCF2Dis -InPut vcf -OutPut p_dis.mat"; the Linux command line of constructing a phylogenetic tree is " fneighbor -datafile p_dis.mat -outfile goat.tree.txt -matrixtype s -treetype n -outtreefile goat.n.tree.tree".

Population structure was analyzed using the ADMIXTURE (version 1.23, RRID:SCR_001263)[58] program which implement a block-relaxation algorithm. To explore the convergence of individuals, we predefined the number of genetic clusters K from 3–7 and ran with cross-validation error (CV) procedure. Default methods and settings were used in Admixture analysis. The analysis process includes the following three steps:

(1) Converting VCF format to PLINK format. The Linux command line was “vcftools --vcf vcf --plink --out goat.plink”;

(2) Using PLINK for further filtering with Linux command line “plink --noweb --file plink --geno 0.05 --maf 0.05 --hwe 0.0001 --chr-set 29 --make-bed --out QC”; after this step we get the corresponding bed file.

(3) ADMIXTURE v1.23 for population structure analysis with “admixture --cv QC.bed $k | tee log${k}.out”; then the results were plotted using R.

PopLDdecay(PopLDdecay, RRID:SCR_022509)[59] for linkage disequilibrium decay analysis was based on variant call format files.

Demographic History

A population-level admixture analysis was conducted in the TreeMix (version 1.12, RRID:SCR_021636)[60]. The program inferred the ML tree for 12 goat breeds (215 individuals) and an outgroup (Bezoars,21 individuals), then the residuals matrix was used to identify pairs of populations that showed poor fits in the ML tree. These populations were regarded as candidates around which we added potential migration edges, and new arrangements of the ML tree accounting for migration events were generated[60]. From one to 20 migration events were gradually added to the ML tree until 98% of the variance between the breeds could be explained. The command was ‘-i input -bootstrap -k 500 -root IRW -o output’.

We used the PSMC (version 0.6.5, RRID:SCR_017229)[22] method to estimate changes in the effective population size (Ne) of goat over the last one million years. The PSMC analysis was implemented in the seven samples sequenced at a high read depth (12.6~26.94×) (Supplementary Table 10).The parameters of PSMC were set to -N25 -t15 -r5 -p “4+25*2+4+6”, where the parameters of generation time and mutation rate were set to 2.0 and 2.5e-8, respectively.

Selective Sweep Analysis

In the selection sweep, we calculated the genome-wide distribution of Fst values and θ_{π} Ratios (i.e., $\theta_{\pi\text{-UC}}/\theta_{\pi\text{-Ordinary}}$, $\theta_{\pi\text{-UC}}/\theta_{\pi\text{-CDMC.IMC.LNC}}$, $\theta_{\pi\text{-LNC.IMC}}/\theta_{\pi\text{-Ordinary}}$, $\theta_{\pi\text{-White}}/\theta_{\pi\text{-Brown}}$ and $\theta_{\pi\text{-White}}/\theta_{\pi\text{-Black}}$) for five control group pairs by VCFtools (VCFtools,

RRID:SCR_001235)[61], which included the UC group versus the ordinary group (GZB, YNBB, JTB, CDB), the UC group versus other cashmere (CDMC, IMC, LNC), the other cashmere group (CDMC, IMC, LNC) versus the ordinary group (GZB, YNBB, JTB, CDB), the white coat color (UC, CDMC, IMC, LNC) versus the brown coat color(CDB), the white coat color(IMC, LNC) versus the black coat color(YNBB, GZB) using a sliding-window approach (150-kb windows with 10-kb increments). The Fst values were Z-transformed, and the θπ Ratios were $\log_2$-transformed. We considered the windows with the top 1% values for the ZFst and $\log_2(\theta_\pi$ Ratios) simultaneously as the candidate outliers under strong selective sweeps. All of the outlier windows were assigned to corresponding SNPs and candidate genes. The signals were further confirmed by ZFst, $\text{Log}_2(\theta_\pi$ Ratios), and Tajima's D with a 10 kb sliding window.

Functional Enrichment Analyses

Candidate genes under selection were defined as those overlapping sweep regions or within 500 kb of the signals. The biological function of genes within candidate regions was annotated by analyzing Gene Ontology (GO) and Kyoto Encyclopedia of Genes and Genomes (KEGG) pathways using Metascape[62]. Benjamini–Hochberg FDR (false discovery rate) was used for correcting the P values. The Gene Ontology categories "Molecular Function," "Biological Process" and "Cellular Component", and Human Phenotype (HP) categories were used in these analyses.

Genotypic and phenotypic analysis

Genotyping. Genotypic and phenotypic analysis DNA was extracted from the ear-tissue samples. The 582 bp deletion near *LHX2* (582del) and the 504 bp deletion of *FGF5* (504del) were genotyped by PCR amplification using the reaction condition of the 2-min pre-denaturation at 95 ℃, 15s-denaturation at 95 ℃, 30s-annealing at 56 ℃, 45s-extension at 72 ℃ for 30 cycle, and 5-min extension at 72 ℃ (the primers of 582 bp deletion: 5'-CAGTACGGAGCAAGTAAACGG-3' and 5'-ACCATTCCACTTGTCCACCT-3', the primers of 504 bp deletion: 5'-

ACAGCGTGTGATCTTTTCTCTG-3’ and 5’-TCTTGGTCTGGCTGTGATCA-3’) and visualized on 2% agarose gels (Supplementary Table 3). The 582del and 540del were successfully genotyped in the extended population of 840 goats, including 752 CDMC, 12 IMC, 40 UC, 12 JNG, 12 Wushan white goats (from Chongqing in the southwest of China) and 12 JTB individuals.

***Phenotypic analysis*.** All 581 cashmere samples were collected from the lateral body part of CDMC goats, and the fiber diameter was measured by an optical-based fiber diameter analyzer (OFDA2000). Sample preparation is the most critical part of obtaining accurate results from OFDA. Briefly, the cashmere fibers are roughly aligned in parallel and cut into 2 mm by the OFDA bench guillotine, and then washed the fibers to remove grease. After drying, a spreader was used to evenly spread 10-15 mg of fibers on the glass slide. The slide placed on the stage, then enlarged and the snippets scanned through the optical transmission microscope. The camera system collects the fiber image, and then the fiber in it is automatically identified through image analysis technology and the diameter is measured[63]. In addition, we measured cashmere yields from a subset of 351 CDMC goats in May 2020. The cashmere production was measured by hand-combing cashmere and weighing. Firstly, using a thin comb to comb the feces and other dirt along the direction of the hair, a density-comb was then used to comb the direction of the hair repeatedly and finally comb the hair in the reverse direction until the fallen fibers are combed. The collected cashmere is weighed with an electronic scale (range 0.3g-5kg, accuracy 0.1g). Then, we tested if the identified deletion variants were associated with cashmere diameter and production, respectively. In total, 581 individuals were recorded, the linear model was expressed as: y=b0+bx+sex+batch+e, where, y was the diameter; x was the deletion coding with 0, 1 and 2 representing 0, 1 and 2 copies of deletion sequence, respectively; b was the effect of deletion variant; sex was the sex effect; batch was the recorder effects, where 2 recorders were participated in the phenotyping; e was the residual error, assumed to follow normal distribution. When performed the association between the deletion variants and the production, 235 individuals were included, all were females, the same linear model used above was employed to perform the association study for production.

The association studies were performed with the lm function of R package[64].

RNAseq

RNAseq data were downloaded from the NCBI database project ID SRP145408. Raw data were filtered by SOAPnuke (version 1.5.6), read base N more than 5%, and read base quality less than 20 was removed. Clean reads from the 12 individual samples were aligned to the reference genome (GCA_001704415.1 ARS1) using hisat2 (version 2.1.0, RRID:SCR_015530). Picard-tools-1.105 was used to sort BAM files. Based on the mapped reads and goat reference genome annotation in GFF (GCF_001704415.1), StringTie (version,1.3.3b, RRID:SCR_016323) was used to calculate the FPKM[65].

Dual-Luciferase Reporter Analysis

Plasmid Constructs, a 563 bp DNA fragment containing "5' end - KpnI/HindIII site-Ins sequence (551bp)-XhoI/NcoI site was synthesized by TsingKe Ltd, which was subsequently cloned downstream and upstream of the SV40 promoter in the pGL3 plasmid, named as Insulator1 and Insulator2, respectively. A 796 bp DNA fragment containing "5' end - BamHI site-Ins sequence(551bp)-SalI site-sv40 enhancer sequence- PciI site – 3' end" (Supplementary Fig. 17) was synthesized by TsingKe Ltd, which was subsequently cloned downstream of Luc+ Poly (A) in the pGL3 promoter plasmid (Promega) and named as Ens-E. Similarly, the well-known insulator cHS4 linked to the sv40 enhancer (Supplementary Fig. 19) was cloned into a pGL3 promoter plasmid and named cHS4-E.

Cell Culture, Transfection, and Luciferase Reporter Assay. The 3T3 cells and 293T cells were maintained in Dulbecco's modified Eagle's medium (Gibco, Waltham, MA, USA) with penicillin (100 units/ml), streptomycin (100 µg/ml), and 10 % fetal bovine serum at 37 °C in a humidified atmosphere of 95 % air and 5 % CO_2. Before transfection, 3T3 and 293T cells were seeded into 24-well plates at 1.0 × 104 cells/well. After overnight attachment, transfections were performed using Lipofectamine 2000 (Invitrogen, CA, USA) according to the manufacturer's instructions. In this experiment, the two types of cells were divided into eight groups, respectively. For each group, plasmid of pGL3-control (Promega), Insulator1 and Insulator2 or Ins-E and cHS4-E

were co-transfected with phRL-TK (Promega) at a ratio of 10:1 (0.8 ug: 0.08 ug) into the 3T3 and 293T cells, respectively. The cells were harvested two days after co-transfection, and luciferase activity was evaluated using the Dual-Luciferase Reporter Assay System (Promega, #E1910). Firefly luciferase data were normalized to Renilla luciferase activity. For this assay, pGL3-control was used as a positive control and Lipofectamine 2000 was used as a blank control. The cHS4 insulator was chosen as a reference to quantify the efficiency of the insulator function.

Data availability.

All sequence data obtained in the current study were uploaded to the NCBI Sequence Read Archive (BioProject accession number PRJNA863495). The data that support the findings of this study have been also deposited into CNGB Sequence Archive (CNSA)[66] of China National GeneBank DataBase (CNGBdb)[67] with accession number CNP0001896. All supporting data and materials are available in the *GigaScience* GigaDB database[68].

List of abbreviations

ANG: Angora goat; BLAST: Basic Local Alignment Search Tool; BWA: Burrows-Wheeler Aligner; CDB: Chengdu brown goat; CDMC: Chaidamu cashmere goat; CNEs: Conserved Noncoding Elements; CTCF:CCCTC-Binding Factor; DRG: dorsal root ganglia; FDR: False Discovery Rate; FPKM: Fragments Per Kilobase Million; GATK: Genome Analysis Toolkit; GO: Gene Ontology; GZB: Guizhou Black goat; HP: Human Phenotype; IGV: Integrative Genomics Viewer; IMC: Inner Mongolia cashmere goat; Ins: insulator; IRW: Iranian wild goat; JNG: Jining gray goat; JTB: Jintang black goat; KEGG: yoto Encyclopedia of Genes and Genomes; Kb: kilobase pairs; KO: Korea goat; LNC: Liaoning cashmere goat; MT: Matou Goat; NCBI: National Center for Biotechnology Information; OFDA: optical-based fiber diameter analyze; PSMC: Pairwise Sequential Markovian Coalescent; RNA-seq: RNA sequencing; SNP: single-nucleotide polymorphism; SRA: Sequence Read Archive; UC: Ujumqin cashmere goat; WGS: Whole-Genome Sequencing; WSW: Wushan White goat; YNBB: Yunnan black bone goat.

Acknowledgements

We thank Bayaertu & Yong Quan from Dongwuzhumuqin Banner Aogali Animal Husbandry Co., Ltd and Hu Yeyong from Henan yudong animal husbandry co. Ltd for

providing UC and MT goat materials, respectively. We thank Shancen Zhao for helpful discussion and revising the manuscript.

Author contributions

L.Y, F.M, Z.S.Y. conceived, designed, and supervised the study. H. H., F. M., L.Y., J.D., C.T. and M.Z.P performed the informatics analysis of the sequencing data. Z.S.Y., Y.M.M., W.Q., Y.C.Y., W.Q.J., WL.B., G.G., Mengkedala., D.W.D., L.B., Z.Q.F. obtained goat material and DNA for re-sequencing. Z.S.Y., Y.M.M., W.Q.J., WL.B., G.G., Mengkedala. and L.B. performed phenotypic data collation and analysis. Y.M.M., W.R., J.D., W.Q. and Z.T.T. participated in the laboratory work. Z.X.J., Z.T.T. and L.L. performed dual-Luciferase Reporter analysis. C.T analyzed the transcriptome. L.Y., H.H., Y.M.M., J.D. and Z.X.J are the major contributors in writing the manuscript. All authors read, revised, and approved the final manuscript.

Additional information

Supplementary Information accompanies this paper at Supplementary.docx and Supplementary table s.xlsx.

Competing interests: The authors declare no competing interests.

Funding

This work was supported by the National Natural Science Foundation of China (31872560); the Science and Technology Innovation Strategy Projects of Guangdong Province (2019B020203002), the Basic Research Project of Haixi Agriculture and Animal Husbandry Bureau (Qinghai Caidamu Cashmere Goat Genomic Breeding project), the Shenzhen Municipal Government of China (JCYJ20180307163440037).

References:

1. Waldron, S, Brown, C, Komarek, AM. The Chinese Cashmere Industry: A Global Value Chain Analysis. *Dev Policy Rev* 2014; **32**(5):589-610.
2. Zeder, MA. Domestication and early agriculture in the Mediterranean Basin: Origins, diffusion, and impact. *Proc Natl Acad Sci USA* 2008; **105**(33):11597-11604.
3. Daly, KG, Maisano, DP, Mullin, VE, et al.. Ancient goat genomes reveal mosaic domestication in the Fertile Crescent. *Science* 2018; **361**(6397):85-88.
4. Ryder, ML. The use of goat hair: an introductory historical review. *Anthropozoologica* 1993.
5. Millar, P. The performance of cashmere goats. *In: Animal Breeding Abstracts:1986* 1986: 181-199.
6. Li, X, Su, R, Wan, W, et al.. Identification of selection signals by large-scale whole-genome resequencing of cashmere goats. *Sci Rep* 2017; **7** (1):15142.
7. Cai, Y, Fu, W, Cai, D, et al.. Ancient Genomes Reveal the Evolutionary History and Origin of Cashmere-Producing Goats in China. *Mol Biol Evol* 2020;**37**(7):2099-2109.
8. Jin, M, Lu, J, Fei, X, et al.. Genetic Signatures of Selection for Cashmere Traits in Chinese Goats. *Animals (Basel)* 2020; **10**(10).
9. Geng, R, Yuan, C, Chen, Y. Exploring differentially expressed genes by RNA-Seq in cashmere goat (Capra hircus) skin during hair follicle development and cycling. *Plos One* 2013; **8**(4):e62704.
10. Geng, R, Wang, L, Wang, X, et al.. Cyclic expression of Lhx2 is involved in secondary hair follicle development in cashmere goat. *Gene Expr Patterns* 2014; **16**(1):31-35.
11. Gao, Y, Wang, X, Yan, H, et al.. Comparative Transcriptome Analysis of Fetal Skin Reveals Key Genes Related to Hair Follicle Morphogenesis in Cashmere Goats. *Plos One* 2016; **11**(3):e151118.
12. Su, R, Fan, Y, Qiao, X, et al.. Transcriptomic analysis reveals critical genes for the hair follicle of Inner Mongolia cashmere goat from catagen to telogen. *Plos One* 2018; **13**(10):e204404.
13. Zhang, Y, Wang, L, Li, Z, et al.. Transcriptome profiling reveals transcriptional and alternative splicing regulation in the early embryonic development of hair follicles in the cashmere goat. *Sci Rep* 2019; **9**(1):17735.
14. Zhang Zhang, Y, Wu, K, Wang, L, et al.. Comparative study on seasonal hair follicle cycling by analysis of the transcriptomes from cashmere and milk goats. *Genomics* 2020; **112**(1):332-345.
15. Dai, B, Hao, F, Xu, T, et al.. Thymosin beta4 Identified by Transcriptomic Analysis from HF Anagen to Telogen Promotes Proliferation of SHF-DPCs in Albas Cashmere Goat. *Int J Mol Sci* 2020; **21**(7).
16. Wang, X, Liu, J, Zhou, G, et al.. Whole-genome sequencing of eight goat populations for the detection of selection signatures underlying production and adaptive traits. *Sci Rep* 2016; **6**:38932.
17. Zhang, B, Chang, L, Lan, X, et al.. Genome-wide definition of selective sweeps reveals molecular evidence of trait-driven domestication among elite goat (Capra species) breeds for the production of dairy, cashmere, and meat. *Gigascience* 2018; **7**(12).
18. Guo, J, Zhong, J, Liu, GE, et al.. Identification and population genetic analyses of copy number variations in six domestic goat breeds and Bezoar ibexes using next-generation sequencing. *BMC Genomics* 2020; **21**(1):840.
19. Wang, X, Cai, B, Zhou, J, et al.. Disruption of FGF5 in Cashmere Goats Using CRISPR/Cas9 Results in More Secondary Hair Follicles and Longer Fibers. *Plos One* 2016; **11**(10):e164640.
20. Li, G, Zhou, S, Li, C, et al.. Base pair editing in goat: nonsense codon introgression into FGF5 results in longer hair. *Febs J* 2019; **286**(23):4675-4692.

21. Li, Y, Song, S, Liu, X, et al.. Deletion of an enhancer in FGF5 is associated with ectopic expression in goat hair follicle s and the cashmere growth phenotype. *bioRxiv* 2020. https://doi.org/10.1101/2020.08.24.264754.
22. Li, H, Durbin, R. Inference of human population history from individual whole-genome sequences. *Nature* 2011; **475**(7357):493-496.
23. Yang, J, Li, WR, Lv, FH, et al.. Whole-Genome Sequencing of Native Sheep Provides Insights into Rapid Adaptations to Extreme Environments. *Mol Biol Evol* 2016; **33**(10):2576-2592.
24. Huelsken, J, Vogel, R, Erdmann, B, et al.. beta-Catenin controls hair follicle morphogenesis and stem cell differentiation in the skin. *Cell* 2001; **105**(4):533-545.
25. Rishikaysh, P, Dev, K, Diaz, D, et al.. Signaling involved in hair follicle morphogenesis and development. *Int J Mol Sci* 2014; **15**(1):1647-1670.
26. McAllister, JM, Modi, B, Miller, BA, et al.. Overexpression of a DENND1A isoform produces a polycystic ovary syndrome theca phenotype. *Proc Natl Acad Sci USA* 2014; **111**(15):e1519-e1527.
27. Paniagua, AE, Segurado, A, Dolon, JF,et al.. Key Role for CRB2 in the Maintenance of Apicobasal Polarity in Retinal Pigment Epithelial Cells. *Front Cell Dev Biol* 2021; **9**:701853.
28. Wu Z, Z, Hai, E, Di, Z, et al.. Using WGCNA (weighted gene co-expression network analysis) to identify the hub genes of skin hair follicle development in fetus stage of Inner Mongolia cashmere goat. *Plos One* 2020; **15**(12):e243507.
29. Lee, AP, Brenner, S, Venkatesh, B. Mouse transgenesis identifies conserved functional enhancers and cis-regulatory motif in the vertebrate LIM homeobox gene Lhx2 locus. *Plos One* 2011; **6**(5):e20088.
30. Grymowicz, M, Rudnicka, E, Podfigurna, A, et al.. Hormonal effects on hair follicles. *Int J Mol Sci* 2020; **6**(5):e20088.
30. Grymowicz, M, Rudnicka, E, Podfigurna, A, et al.. Hormonal effects on hair follicles. *Int J Mol Sci* 2020; **21**(15):5342.
31. Yang, F, Liu, Z, Zhao, M, et al.. Skin transcriptome reveals the periodic changes in genes underlying cashmere (ground hair) follicle transition in cashmere goats. *BMC Genomics* 2020; **21**(1):392.
32. Zheng-lu, H, Zhaeng-kui, Y, DARIQIBU. The Breeding of Chai Da-mu Cashmere Coat in Wulan County of Qinghai Province. *Ecology Of Domestic Animal* 2003; **24**:76-78.
33. Kim, JY, Jeong, S, Kim, KH, et al.. Discovery of Genomic Characteristics and Selection Signatures in Korean Indigenous Goats Through Comparison of 10 Goat Breeds. *Front Genet* 2019; **10**:699.
34. Rhee, H, Polak, L, Fuchs, E. Lhx2 maintains stem cell character in hair follicles. *Science* 2006; **312**(5782):1946-1949.
35. Tornqvist, G, Sandberg, A, Hagglund, AC, et al.. Cyclic expression of lhx2 regulates hair formation. *Plos Genet* 2010; **6**(4):e1000904.
36. Xiao, Z, Patrakka, J, Nukui, M, et al.. Deficiency in Crumbs homolog 2 (Crb2) affects gastrulation and results in embryonic lethality in mice. *Dev Dyn* 2011; **240**(12):2646-2656.
37. Shi, J, Gao, Q, Cao, Y, et al.. Dennd1a, a susceptibility gene for polycystic ovary syndrome, is essential for mouse embryogenesis. *Dev Dyn* 2019; **248**(5):351-362.
38. West, AG, Gaszner, M, Felsenfeld, G. Insulators: many functions, many mechanisms. *Genes Dev* 2002; **16**(3):271-288.
39. Creyghton, MP, Cheng, AW, Welstead, GG, et al.. Histone H3K27ac separates active from poised enhancers and predicts developmental state. *Proc Natl Acad Sci USA* 2010; **107**(50):21931-21936.
40. Higgins, CA, Petukhova, L, Harel, S, et al.. FGF5 is a crucial regulator of hair length in humans. *Proc Natl Acad Sci USA* 2014; **111**(29):10648-10653.

41. Folgueras, AR, Guo, X, Pasolli, HA, et al.. Architectural niche organization by LHX2 is linked to hair follicle stem cell function. *Cell Stem Cell* 2013; **13**(3):314-327.
42. Kishimoto, J, Burgeson, RE, Morgan, BA. Wnt signaling maintains the hair-inducing activity of the dermal papilla. *Gene Dev* 2000; **14**(10):1181-1185.
43. Krieger, K, Millar, SE, Mikuda, N, et al.. NF-kappaB Participates in Mouse Hair Cycle Control and Plays Distinct Roles in the Various Pelage Hair Follicle Types. *J Invest Dermatol* 2018; **138**(2):256-264.
44. Choi, BY. Targeting Wnt/beta-Catenin Pathway for Developing Therapies for Hair Loss. *Int J Mol Sci* 2020; **21**(14).
45. Tomann, P, Paus, R, Millar, et al.. Lhx2 is a direct NF-kappaB target gene that promotes primary hair follicle placode down-growth. *Development* 2016; **143**(9):1512-1522.
46. Kwack, MH, Jun, MS, Sung, YK, et al.. Ectodysplasin-A2 induces dickkopf 1 expression in human balding dermal papilla cells overexpressing the ectodysplasin A2 receptor. *Biochem Biophys Res Commun* 2020; **529**(3):766-772.
47. Harel, S, Higgins, CA, Cerise, JE, et al.. Pharmacologic inhibition of JAK-STAT signaling promotes hair growth. *Sci Adv* 2015; **1**(9):e1500973.
48. Tao, Y, Zhou, X, Liu, Z, et al.. Expression patterns of three JAK-STAT pathway genes in feather follicle development during chicken embryogenesis. *Gene Expr Patterns* 2020; **35**:119078.
49. Randall, VA. The endocrine control of the hair follicle. In: Hair Growth and Disorders. *Springer* 2008; 23-39.
50. Inui, S, Itami, S. Androgen actions on the human hair follicle: perspectives. *Exp Dermatol* 2013; **22**(3):168-171.
51. Chen, Y, Chen, Y, Shi, C, et al.. SOAPnuke: a MapReduce acceleration-supported software for integrated quality control and preprocessing of high-throughput sequencing data. *Gigascience* 2018; **7**(1):1-6.
52. Hassanin, A, Bonillo, C, Nguyen, BX, et al.. Comparisons between mitochondrial genomes of domestic goat (*Capra hircus*) reveal the presence of numts and multiple sequencing errors. *Mitochondr DNA* 2010; **21**(3-4):68-76.
53. Danecek P, Bonfield JK, Liddle J, Marshall J, Ohan V, Pollard MO, Whitwham A, Keane T, McCarthy SA, Davies RM, Li H. Twelve years of SAMtools and BCFtools. Gigascience. 2021 Feb 16;10(2):giab008. doi: 10.1093/gigascience/giab008.
54. McKenna, A, Hanna, M, Banks, E, et al.. The Genome Analysis Toolkit: a MapReduce framework for analyzing next-generation DNA sequencing data. *Genome Res* 2010; **20**(9):1297-1303.
55. Choi, JW, Choi, BH, Lee, SH, et al.. Whole-Genome Resequencing Analysis of Hanwoo and Yanbian Cattle to Identify Genome-Wide SNPs and Signatures of Selection. *Mol Cells* 2015; **38**(5):466-473.
56. Chang CC, Chow CC, Tellier LC, Vattikuti S, Purcell SM, Lee JJ. Second-generation PLINK: rising to the challenge of larger and richer datasets. Gigascience. 2015 Feb 25;4:7. doi: 10.1186/s13742-015-0047-8.
57. Retief, JD. Phylogenetic analysis using PHYLIP. *Methods Mol Biol* 2000; **132**:243-258.
58. , DH, Lange, K. Enhancements to the ADMIXTURE algorithm for individual ancestry estimation. *BMC Bioinformatics* 2011; **12**:246.
59. Zhang, C, Dong, SS, Xu, JY, et al.. PopLDdecay: a fast and effective tool for linkage disequilibrium decay analysis based on variant call format files. *Bioinformatics* 2019; **35**(10):1786-1788.
60. Pickrell, JK, Pritchard, JK. Inference of population splits and mixtures from genome-wide allele

frequency data. *Plos Genet* 2012; **8**(11):e1002967.
61. Danecek, P, Auton, A, Abecasis, G, et al.. The variant call format and VCFtools. *Bioinformatics* 2011; **27**(15):2156-2158.
62. Zhou, Y, Zhou, B, Pache, L, et al.. Metascape provides a biologist-oriented resource for the analysis of systems-level datasets. *Nat Commun* 2019; **10**(1):1523.
63. Brims, MA, Peterson, AD, Gherardi, SG. Introducing the OFDA2000 - For rapid measurement of diameter profile on greasy wool staples. *IWTO, Raw wool group report RWG04, Florence, Italy* 1999.
64. Core, TR. R: A language and environment for statistical computing. *R Foundation for Statistical Computing, Vienna, Austria* 2021.
65. Pertea, M, Kim, D, Pertea, GM, et al.. Transcript-level expression analysis of RNA-seq experiments with HISAT, StringTie and Ballgown. *Nat Protoc* 2016; **11**(9):1650-1667.
66. Guo, X, Chen, F, Gao, F, et al.. CNSA: a data repository for archiving omics data. *Database (Oxford)* 2020 2020.
67. Chen, FZ, You, LJ, Yang, F, et al.. CNGBdb: China National GeneBank DataBase. *Yi Chuan* 2020; **42**(8):799-809.
68. Han, H, Yang, M, Dan, J, et al.. Supporting data for “Selection signatures in goats reveal a novel deletion mutant underlying cashmere yield and diameter”. *Gigascience Database* 2022; http://dx.doi.org/10.5524/102245.

 Click here to access/download;Table;Revised-Supplementary tables .xlsx

Supplementary Table s1: Variant analysis

Number of effects by type and region

Type (alphabetical order)	Count	Percent
3_prime_UTR_variant	143948	0.00379
JTR_premature_start_codon_ga	4579	0.00012
5_prime_UTR_variant	26854	0.00071
downstream_gene_variant	1861794	0.04906
initiator_codon_variant	8	0
intergenic_region	8165402	0.21517
intragenic_variant	150948	0.00398
intron_variant	12728720	0.33542
missense_variant	69410	0.00183
n_coding_transcript_exon_varia	47626	0.00126
non_coding_transcript_variant	12788241	0.33699
splice_acceptor_variant	214	0.00001
splice_donor_variant	294	0.00001
splice_region_variant	21247	0.00056
start_lost	80	0
stop_gained	720	0.00002
stop_lost	104	0
stop_retained_variant	79	0
synonymous_variant	114308	0.00301
upstream_gene_variant	1824134	0.04807

Type (alphabetical order)	Count	Percent
DOWNSTREAM	1,861,794	4.91%
EXON	229,487	0.61%
INTERGENIC	8,165,402	21.53%
INTRON	12,711,669	33.52%
SPLICE_SITE_ACCEPTOR	214	0.00%
SPLICE_SITE_DONOR	286	0.00%
SPLICE_SITE_REGION	19,970	0.05%
TRANSCRIPT	12,939,189	34.12%
UPSTREAM	1,824,134	4.81%
UTR_3_PRIME	143,948	0.38%
UTR_5_PRIME	31,433	0.08%

Transition, transversions,heterozygou

	Transitions	ransversion	Ts/Tv
CMS0019	4837778	1965207	2.462
CMS0026	4886197	1989777	2.456
CMS0037	4873889	1982624	2.458
CMS0053	4865412	1980185	2.457
CMS0065	4750755	1933162	2.458
CMS0084	4933740	2008058	2.457
CMS0088	4918530	2000855	2.458
CMS0100	4856598	1974396	2.46
CMS0177	4896586	1991074	2.459
CMS0330	4938887	2009693	2.458
CMS0406	4807382	1955796	2.458
CMS0431	4738301	1929768	2.455
FANG0001	4679623	1901423	2.461
FANG0002	4633702	1883595	2.46
FANG0003	4225263	1713969	2.465
FANG0004	4575046	1858633	2.462
FANG0005	4601259	1871377	2.459
FANG0006	4497697	1827831	2.461
FANG0007	4581889	1861965	2.461
FANG0008	4550894	1847958	2.463
FANG0009	4569499	1857683	2.46
FANG0010	4120058	1673279	2.462
FANG0011	4677072	1900764	2.461
FANG0012	4291664	1742635	2.463
FANG0013	4681846	1906700	2.455
FANG0014	4584488	1863026	2.461
FANG0015	4626702	1883543	2.456
FANG0016	4579646	1861035	2.461
FANG0017	4655770	1893673	2.459
FANG0018	4624320	1879983	2.46
FANG0019	4593648	1869683	2.457
FANG0020	4588270	1866890	2.458
GHS0001	4744112	1929650	2.459
GHS0004	4224831	1709827	2.471
GHS0010	4723753	1915114	2.467
GHS0013	4548471	1841757	2.47
GHS0017	4858799	1975687	2.459
GHS0025	4940434	2012178	2.455
GHS0026	4809356	1956672	2.458
GHS0027	4740159	1925521	2.462
HXR0014	4707226	1909615	2.465
HXR0022	4649005	1885056	2.466
HXR0044	4731846	1924468	2.459
HXR0045	4724387	1919715	2.461

HXR0047	4774168	1940808	2.46
HXR0126	4706621	1912074	2.462
HXR0127	4837470	1967163	2.459
HXR0138	4699357	1906388	2.465
HXR0147	4785941	1944092	2.462
HXR0157	4587457	1859423	2.467
HXR0170	4682856	1900508	2.464
HXR0176	4746635	1929385	2.46
HXR0186	4690923	1904642	2.463
HXR0370	4797141	1950052	2.46
HXR0711	4814526	1957390	2.46
HXR0908	4750305	1929275	2.462
HXR097	4745697	1925699	2.464
HXR0981	4797132	1946015	2.465
HXR1166	4813587	1956855	2.46
HXR1167	4800868	1950484	2.461
HXR1188	4786503	1949039	2.456
HXR1321	4707738	1901983	2.475
HXR1630	4804837	1949751	2.464
HXR171	4852817	1974535	2.458
HXR1750	4682805	1896889	2.469
HXR2752	4700454	1903400	2.47
HXR2781	4803429	1956311	2.455
HXR3030	4805300	1953801	2.459
HXR3386	4793660	1948490	2.46
HXR3388	4783978	1943524	2.461
IMCG01	3665203	1472249	2.49
IMCG02	2288096	939987	2.434
IMCG04	3398897	1357096	2.505
IMCG05	1760586	717809	2.453
IMCG051	2752376	1094892	2.514
IMCG10	4104033	1673747	2.452
IMCG101	1977399	795493	2.486
IMCG11	3502247	1417191	2.471
IMCG111	4292216	1743803	2.461
IMCG12	4370129	1773584	2.464
IMCG121	2030459	818514	2.481
IMCG13	3784290	1519187	2.491
IMCG14	4078266	1651810	2.469
IMCG141	2519185	1020517	2.469
IMCG15	3593329	1448565	2.481
IMCG19	3037349	1215380	2.499
IMCG21	3049040	1217633	2.504
IMCG213	2447982	985647	2.484
IMCG25	3295914	1324699	2.488
IMCG27	2708139	1100209	2.461
IMCG28	2701882	1060098	2.549

IMCG284	2979143	1199292	2.484
IMCG29	3183594	1295829	2.457
IMCG293	3141090	1263216	2.487
IMCG30	2965523	1208470	2.454
IMCG31	3824253	1555857	2.458
IMCG311	1964636	769465	2.553
IMCG313	3887200	1579170	2.462
IMCG33	3943823	1595554	2.472
IMCG35	2323411	930568	2.497
IMCG36	1963278	805482	2.437
IMCG37	1802025	689202	2.615
IMCG39	2286303	907292	2.52
IMCG43	1548317	639237	2.422
IMCG47	2856397	1140882	2.504
IMCG55	4265119	1726278	2.471
IMCG710	3316472	1321069	2.51
IRWG01	4542648	1841177	2.467
IRWG04	4596021	1868311	2.46
IRWG06	4519506	1833045	2.466
IRWG26	4555609	1852561	2.459
IRWG28	4208901	1709979	2.461
IRWG29	4235301	1724238	2.456
IRWG30	4582458	1863247	2.459
IRWG31	4551620	1849249	2.461
IRWG33	4314618	1748795	2.467
IRWG34	4547623	1847524	2.461
IRWG35	4364188	1773509	2.461
IRWG36	4275071	1738165	2.46
IRWG38	4558273	1854286	2.458
IRWG40	4561493	1854091	2.46
IRWG41	4140088	1682503	2.461
IRWG42	4271602	1735043	2.462
IRWG43	4385973	1780967	2.463
IRWG44	4534733	1842463	2.461
IRWG45	4549033	1850680	2.458
IRWG47	4567203	1857772	2.458
IRWG48	4558729	1852981	2.46
JTY0001	4716406	1918437	2.458
JTY0012	4790504	1947037	2.46
JTY0022	4820776	1961206	2.458
JTY0034	4799353	1951201	2.46
JTY0038	4637031	1881566	2.464
JTY0042	4734210	1924772	2.46
JTY0045	4784910	1943188	2.462
JTY0050	4839508	1966962	2.46
JTY0062	4834459	1968386	2.456
JTY0076	4700786	1911536	2.459

KOG06	4758291	1934079	2.46
KOG07	4738870	1924353	2.463
KOG08	4751755	1933669	2.457
KOG09	4732128	1925127	2.458
KOG10	4817670	1957979	2.461
KOG11	4817408	1958961	2.459
KOG12	4786908	1948262	2.457
KOG13	4745564	1930083	2.459
KOG14	4764570	1934533	2.463
KOG15	4777653	1941916	2.46
KOG16	4709410	1912580	2.462
KOG17	4797522	1948302	2.462
LNC18	3434664	1396245	2.46
LNC21	3275129	1327134	2.468
LNC22	2158778	871105	2.478
LNC24	3984894	1608438	2.477
LNC31	2747995	1088785	2.524
LNC33	4019888	1622353	2.478
LNC34	2558267	1014874	2.521
LNC35	2248355	906212	2.481
LNC36	1893940	751498	2.52
LNC37	3625684	1461432	2.481
LNC38	3143969	1269574	2.476
LNC39	2611079	1046471	2.495
LNC47	3940464	1597660	2.466
LNC48	1903770	748854	2.542
LNC52	3838749	1568098	2.448
LNC54	3610768	1468331	2.459
LNC56	3827018	1552754	2.465
MANG0027	4582766	1860913	2.463
MANG0028	4625256	1879124	2.461
MANG0029	4621407	1877745	2.461
MANG0030	4592110	1868085	2.458
MANG0031	4578920	1861705	2.46
MANG0033	4543865	1843396	2.465
MTG0001	4535595	1846177	2.457
MTG0002	4456174	1814047	2.456
MTG0003	4659277	1896810	2.456
MTG0005	4581905	1864881	2.457
MTG0006	4624908	1880555	2.459
MTG0007	4525517	1841363	2.458
MTG0009	4611562	1878143	2.455
MTG0010	4464875	1816356	2.458
MTG0012	4514676	1836212	2.459
MTG0013	4558975	1854355	2.459
WMG0004	4873414	1983066	2.458
WMG0005	4834260	1965391	2.46

WMG0006	4862953	1978027	2.458
WMG0007	4829670	1964856	2.458
WMG0008	4859964	1978611	2.456
WMG0009	4740918	1925555	2.462
WMG0010	4733810	1919313	2.466
WMG0011	4719686	1918904	2.46
WMG0012	4821270	1962830	2.456
WMG0013	4596650	1863401	2.467
WMG0016	4767604	1936221	2.462
WMG0017	4766888	1935541	2.463
ZANG0374	4651480	1891010	2.46
ZANG0388	4606987	1875985	2.456
ZANG0397	4565134	1855066	2.461
BBG97	5002011	2038281	2.454
BBG274	5013774	2045020	2.452
BBG3101	5050388	2058782	2.453
BBG6106	5041750	2054840	2.454
BBG7111	5056297	2060884	2.453
BBG8111	5043197	2053634	2.456
BBG974	5011988	2039363	2.458
BBG10101	5027752	2048087	2.455
BBG12142	5063532	2063337	2.454
BBG1674	5024566	2045899	2.456
BBG1790	5060370	2064735	2.451
BBG1897	5022681	2045385	2.456
BBG26101	5053938	2061724	2.451
BBG28106	5032394	2052822	2.451
BBG31107	5053298	2058487	2.455
BBG3674	5032982	2052122	2.453
BBG39133	5048593	2058965	2.452
BBG8094	5051140	2058248	2.454
BBG8166	5056106	2061424	2.453
BBG8266	5032147	2050317	2.454
BBG8396	5051364	2057716	2.455
BBG8990	4995544	2035088	2.455
BBG90100	5054221	2062650	2.45
BBG9195	5034501	2051946	2.454
BBG93108	4914437	2000158	2.457
BBG9590	4992165	2035267	2.453
BBG9896	5061646	2063397	2.453
BBG99111	5039676	2052509	2.455
GRG15	4814203	1955080	2.462
GRG16	4898094	1987130	2.465
GRG17	4793357	1947851	2.461
GRG18	4807527	1950097	2.465
GRG43	4856641	1975217	2.459
GRG45	4741643	1922984	2.466

GRG47	4794290	1941320	2.47
GRG75	4830171	1958457	2.466
GRG76	4753862	1923015	2.472
GRG77	4758180	1929858	2.466
Total	1.03E+09	4.16E+08	2.463

s,homozygous per sample

Het	Hom	Missing
2814879	1994053	32720
2688936	2093519	25286
2812859	2021827	21409
2750591	2047503	27876
2678155	2002881	68267
2834852	2053473	11607
3064929	1927228	9696
2837910	1996542	35968
3236108	1825776	12688
2908936	2019822	14731
2615124	2074027	44354
2903727	1882171	65227
3087782	1746632	6758
2438695	2039301	27605
2296948	1821142	237898
3035373	1699153	24711
2827144	1822746	26259
2543452	1891038	69935
3000810	1721522	23577
2883732	1757560	43262
3008380	1709401	36287
2184041	1804648	376968
3194428	1691704	11439
2030787	2001756	230340
3317998	1635274	6493
2794838	1826338	19093
3267309	1621468	18420
2910423	1765129	31794
3172693	1688375	12136
3160291	1672006	18259
3166801	1648265	16421
2516200	1969480	32142
3101378	1786192	54803
2154020	1890319	484394
2799219	1919824	87696
2673266	1858481	183966
2752426	2041030	48388
3461350	1745631	7266
3275800	1745114	31239
3399626	1633027	46158
3237605	1689618	43177
3179415	1677323	76308
3449040	1603637	29380
3356530	1643786	40517

Variants rate details

Chromosome	Length	Variants	/ariants rate
NC_030808.1	157,403,528	859,183	183
NC_030809.1	136,510,947	692,713	197
NC_030810.1	120,038,259	614,182	195
NC_030811.1	120,734,966	651,557	185
NC_030812.1	119,020,588	613,281	194
NC_030813.1	117,642,375	667,668	176
NC_030814.1	108,433,636	552,138	196
NC_030815.1	112,672,867	589,473	191
NC_030816.1	91,568,626	470,259	194
NC_030817.1	101,087,560	520,544	194
NC_030818.1	106,225,002	518,441	204
NC_030819.1	87,277,232	494,126	176
NC_030820.1	83,034,183	381,712	217
NC_030821.1	94,672,733	498,882	189
NC_030822.1	81,904,557	457,195	179
NC_030823.1	79,370,172	400,298	198
NC_030824.1	71,137,785	375,534	189
NC_030825.1	67,275,902	324,020	207
NC_030826.1	62,516,450	275,063	227
NC_030827.1	71,784,255	403,218	178
NC_030828.1	69,425,955	365,269	190
NC_030829.1	60,283,066	275,428	218
NC_030830.1	48,866,549	280,335	174
NC_030831.1	62,310,066	314,334	198
NC_030832.1	42,858,509	195,942	218
NC_030833.1	51,421,553	283,460	181
NC_030834.1	44,709,034	240,694	185
NC_030835.1	44,672,302	267,592	166
NC_030836.1	51,332,696	279,336	183
IW_017189516.	66,011,198	133,608	494
IW_017189517.	49,932,331	74,439	670
Total	2,582,134,882	########	197

3414674	1650151	24005
3324957	1646869	50562
3555647	1624493	10981
3306475	1649635	47496
3437885	1646074	24510
3072226	1687327	97370
3306806	1638279	55143
3416500	1629760	29505
3279843	1657861	53991
3432493	1657350	19079
3514874	1628521	14772
3386930	1646325	33491
3274562	1698417	34587
3461279	1640934	25599
3381110	1694666	19770
3263740	1743806	28313
3463664	1635939	24295
3254799	1677461	91669
3517192	1618698	15649
3283786	1771783	15439
3021336	1779179	76604
3156036	1723909	60714
3422874	1668433	14666
3179613	1789744	22461
3407568	1667291	23739
3081354	1823074	26120
1554236	1791608	1383378
624679	1301702	3410130
1148489	1803752	2175578
348961	1064717	4827797
776810	1535229	3077657
2138786	1819497	515779
458298	1157297	4169871
1820064	1549687	1217807
2635813	1700103	259809
2200781	1971466	341705
471363	1188805	3867581
1759305	1772086	903840
2214732	1757672	473333
674552	1432575	3383217
1520236	1760829	1089893
991867	1630431	2486240
929993	1668340	1992930
601969	1415830	3656287
1351153	1634730	1475115
829070	1489639	2771456
917000	1422490	2672987

973303	1602566	2464420
1192593	1643415	1765113
1042746	1680780	2392795
1051509	1561242	2161232
1873560	1753275	892758
574965	1079568	4367256
1850000	1808185	1024549
2080719	1729329	616873
593167	1330406	3374041
374026	1197367	4367294
373003	1059112	5124073
602621	1295487	3344074
290370	948592	5138864
841701	1577789	2537154
2520541	1735428	307276
1414463	1611539	1515295
2850381	1766722	82531
2856502	1803915	9354
2804883	1773834	71197
2481608	1963281	10100
2247604	1835638	161872
1422833	2268353	249869
2829455	1808125	9002
2345357	2027756	10698
1713131	2175141	153190
2201841	2096653	11315
1855091	2141303	118266
1991756	2010740	133190
2646659	1882950	12249
2486242	1964671	10062
1519715	2151438	298446
1972433	2017106	138548
2155906	2005517	68339
2476038	1950579	13473
2338155	2030779	10851
2348501	2038237	12632
2740580	1835565	17612
3199843	1717500	44181
3191047	1773247	22187
3135348	1823317	28460
3329692	1710431	21065
2610453	1954072	109755
2900456	1879263	54319
2881812	1923143	40419
3336012	1735229	16151
3231399	1785723	26727
2840436	1885943	64273

2475766	2108302	23393
2958147	1852538	17891
3103308	1791058	17460
3139363	1758946	23104
2442139	2166755	21375
2508939	2133715	14449
2888072	1923549	18136
2746933	1964357	18256
2741093	1979005	23541
2658157	2030706	21666
2733886	1944052	32976
2522572	2111626	19429
1234377	1798266	1539714
1091213	1755525	1895583
480607	1274638	3894873
1787632	1902850	754569
771338	1532721	2985587
1826325	1907958	827984
594699	1489221	3529334
464295	1345136	4190303
325336	1160051	5155657
1365888	1860614	1368749
749111	1832216	2183372
590516	1533517	3496411
1787264	1875430	819542
380512	1136056	4587176
1556071	1925388	1140955
1433303	1822898	1307137
1568246	1905763	991348
3098785	1672447	2028
3187576	1658402	2506
2932510	1783321	2447
3100031	1680082	3543
3018215	1711205	10096
3032111	1677575	10777
3200444	1590664	39284
3075661	1597280	76354
2596079	1980004	13664
3326290	1560248	16285
3407349	1549057	14458
3224568	1571156	10813
2560021	1964842	28250
3113995	1583618	45784
2935040	1707924	43708
3197404	1607963	33970
3656126	1600177	3607
3584497	1607577	8452

3242944	1799018	4569
3165590	1814468	12104
3673995	1582290	6294
3437403	1614535	25962
3398843	1627140	34402
3392736	1622927	31983
3606478	1588811	8219
3141519	1659266	82397
2589157	2057334	26568
3404999	1648715	25628
2889016	1826737	7541
2846172	1818400	14954
2820810	1799695	27047
3530052	1755120	1958
3430110	1814342	3775
3480416	1814377	2535
3300100	1898245	3056
3496091	1810545	2277
3446209	1825311	2099
3532721	1759315	2210
3112423	1981708	2957
3359123	1883873	4326
3372381	1849042	4168
3261049	1932028	3315
3464472	1801797	1655
3444188	1835737	2801
3425142	1830037	4939
2883535	2114125	2157
3363748	1860678	3274
3551144	1778207	2839
3403118	1853135	2779
3499620	1808955	1989
3448514	1816975	1737
3455500	1826790	2999
3584048	1723292	2792
3440873	1837999	2799
3410679	1837884	2071
3261999	1826298	22007
3587508	1719962	2618
3410865	1857089	2577
3466093	1813046	1865
3657113	1556085	7399
3686570	1599327	7793
3588308	1576450	17269
3675180	1541222	18967
3708852	1561503	5184
3550765	1556931	29580

3646246	1544682	38496
3619458	1584585	30379
2450633	2113122	122240
3665968	1511035	17263

Supplementary Table s2:The candidate genes in the selection sweep of UC goats and ordinary goats

Gene	Chr	Bin_start	Bin_end	Gene_Start	Gene_end	ZFst	log 2 (θπ ratio)
CBLB	chr1	50270001	50420001	50156574	50379147	4.045866	1.026640173
ALCAM	chr1	50270001	50420001	49834499	50068116	4.045866	1.026640173
BCHE	chr1	1.01E+08	1.01E+08	1.01E+08	1.01E+08	4.739633	1.231168682
WARS2	chr3	96550001	96700001	96453280	96556143	6.977931	2.118194997
HAO2	chr3	96650001	96800001	96792486	96828594	4.421209	1.249012327
TBX15	chr3	96550001	96700001	96299835	96424984	6.977931	2.118194997
WARS2	chr3	96560001	96710001	96453280	96556143	6.901183	2.098734355
HAO2	chr3	96550001	96700001	96792486	96828594	6.977931	2.118194997
3BHSD	chr3	96550001	96700001	96839059	96848990	6.977931	2.118194997
ZNF697	chr3	96550001	96700001	96860774	96894430	6.977931	2.118194997
PHGDH	chr3	96550001	96700001	96957379	96988400	6.977931	2.118194997
HMGCS2	chr3	96550001	96700001	96993773	97024594	6.977931	2.118194997
REG4	chr3	96550001	96700001	97053866	97080492	6.977931	2.118194997
NOTCH2	chr3	96600001	96750001	97171972	97351176	6.674734	1.652744876
PIK3CG	chr4	72590001	72740001	72257537	72291605	4.318878	1.01187
CCDC71L	chr4	72590001	72740001	72481791	72486442	4.318878	1.01187
NAMPT	chr4	72590001	72740001	72928492	72971902	4.318878	1.01187
SYPL1	chr4	72590001	72740001	73117696	73148219	4.318878	1.01187
CYSTM1	chr7	59130001	59280001	59064021	59134162	5.469975	1.253373931
IGIP	chr7	59140001	59290001	59183771	59183983	5.847594	1.193806765
PURA	chr7	59140001	59290001	59193011	59204429	5.847594	1.193806765
NRG2	chr7	59140001	59290001	59276390	59474026	5.847594	1.193806765
HARS	chr7	59120001	59270001	58682131	58695144	4.910462	1.15632183
DND1	chr7	59120001	59270001	58695237	58698549	4.910462	1.15632183
WDR55	chr7	59120001	59270001	58698546	58703385	4.910462	1.15632183
IK	chr7	59140001	59290001	58705364	58718109	5.847594	1.193806765
NDUFA2	chr7	59140001	59290001	58718219	58720374	5.847594	1.193806765
TMCO6	chr7	59140001	59290001	58720332	58725924	5.847594	1.193806765
CD14	chr7	59140001	59290001	58731680	58735123	5.847594	1.193806765
SLC35A4	chr7	59140001	59290001	58754826	58759575	5.847594	1.193806765
APBB3	chr7	59140001	59290001	58759676	58766097	5.847594	1.193806765
SRA1	chr7	59140001	59290001	58766299	58773099	5.847594	1.193806765
EIF4EBP3	chr7	59140001	59290001	58773687	58774464	5.847594	1.193806765
SLC4A9	chr7	59140001	59290001	58927092	58941009	5.847594	1.193806765
HBEGF	chr7	59140001	59290001	58936015	58967277	5.847594	1.193806765
PFDN1	chr7	59140001	59290001	58990749	59062745	5.847594	1.193806765
CYSTM1	chr7	59140001	59290001	59064021	59134162	5.847594	1.193806765
NRG2	chr7	59120001	59270001	59276390	59474026	4.910462	1.15632183
PSD2	chr7	59140001	59290001	59477094	59549870	5.847594	1.193806765
CXXC5	chr7	59140001	59290001	59617412	59651710	5.847594	1.193806765
UBE2D2	chr7	59140001	59290001	59671314	59714771	5.847594	1.193806765
KIAA2026	chr8	38560001	38710001	38513727	38622147	4.466058	1.379238809
MLANA	chr8	38560001	38710001	38632729	38645056	4.466058	1.379238809

ERMP1	chr8	38570001	38720001	38711983	38774165	4.407573	1.360757448
RCL1	chr8	39570001	39720001	39520371	39583801	6.049661	1.584187032
AK3	chr8	39590001	39740001	39635494	39656272	6.08271	1.760158455
CDC37L1	chr8	39590001	39740001	39660101	39678648	6.08271	1.760158455
PLPP6	chr8	39590001	39740001	39700133	39703413	6.08271	1.760158455
SPATA6L	chr8	39590001	39740001	39734093	39756809	6.08271	1.760158455
SLC1A1	chr8	39630001	39780001	39770036	39845247	4.571571	1.466562188
GLDC	chr8	38560001	38710001	38074650	38160199	4.466058	1.379238809
UHRF2	chr8	38560001	38710001	38178911	38250733	4.466058	1.379238809
TPD52L3	chr8	38560001	38710001	38297532	38298263	4.466058	1.379238809
IL33	chr8	38560001	38710001	38337407	38408297	4.466058	1.379238809
RANBP6	chr8	38560001	38710001	38506959	38511581	4.466058	1.379238809
ERMP1	chr8	38560001	38710001	38711983	38774165	4.466058	1.379238809
RIC1	chr8	38560001	38710001	38776219	38919131	4.466058	1.379238809
PDCD1LG2	chr8	38560001	38710001	38979921	39046980	4.466058	1.379238809
CD274	chr8	38560001	38710001	39077983	39095141	4.466058	1.379238809
PLGRKT	chr8	39570001	39720001	39111116	39155487	6.049661	1.584187032
INSL6	chr8	39590001	39740001	39210866	39227365	6.08271	1.760158455
JAK2	chr8	39590001	39740001	39245187	39360411	6.08271	1.760158455
RCL1	chr8	39590001	39740001	39520371	39583801	6.08271	1.760158455
CDC37L1	chr8	39510001	39660001	39660101	39678648	4.064055	0.981399549
PLPP6	chr8	39550001	39700001	39700133	39703413	5.256231	1.300527496
SPATA6L	chr8	39570001	39720001	39734093	39756809	6.049661	1.584187032
SLC1A1	chr8	39590001	39740001	39770036	39845247	6.08271	1.760158455
GLIS3	chr8	39590001	39740001	40049592	40544666	6.08271	1.760158455
NCOA1	chr11	74200001	74350001	74153980	74372125	5.76972	0.93259616
RNAC-AC	chr11	74190001	74340001	74190725	74190796	5.679361	0.895461035
CRB2	chr11	94010001	94160001	94006350	94030821	6.979376	0.85927
DENND1A	chr11	94370001	94520001	94030061	94571614	12.1586	3.09584
RNAC-GC	chr11	94370001	94520001	93961937	93962008	12.1586	3.09584
DNMT3A	chr11	74200001	74350001	73679835	73784188	5.76972	0.93259616
POMC	chr11	74200001	74350001	73833491	73836904	5.76972	0.93259616
EFR3B	chr11	74200001	74350001	73838760	73935019	5.76972	0.93259616
DNAJC27	chr11	74200001	74350001	73977548	74015566	5.76972	0.93259616
ADCY3	chr11	74200001	74350001	74037527	74125235	5.76972	0.93259616
CENPO	chr11	74200001	74350001	74124434	74138988	5.76972	0.93259616
PTRHD1	chr11	74200001	74350001	74138657	74142150	5.76972	0.93259616
RNAC-AC	chr11	74200001	74350001	74190725	74190796	5.76972	0.93259616
ITSN2	chr11	74200001	74350001	74499640	74622540	5.76972	0.93259616
FAM228A	chr11	74200001	74350001	74624305	74636502	5.76972	0.93259616
FAM228B	chr11	74200001	74350001	74637060	74685661	5.76972	0.93259616
PFN4	chr11	74200001	74350001	74669150	74674491	5.76972	0.93259616
TP53I3	chr11	74200001	74350001	74678510	74685183	5.76972	0.93259616
SF3B6	chr11	74200001	74350001	74685654	74693957	5.76972	0.93259616
FKBP1B	chr11	74200001	74350001	74698136	74708853	5.76972	0.93259616
WDCP	chr11	74200001	74350001	74711378	74726672	5.76972	0.93259616
MFSD2B	chr11	74200001	74350001	74729851	74742391	5.76972	0.93259616

UBXN2A	chr11	74200001	74350001	74745868	74769527	5.76972	0.93259616
ATAD2B	chr11	74210001	74360001	74782987	74910341	5.494236	0.859224999
RC3H2	chr11	94000001	94150001	93525713	93577732	6.821547	0.851769
ZBTB6	chr11	94010001	94160001	93579682	93587389	6.979376	0.85927
ZBTB26	chr11	94010001	94160001	93589694	93604487	6.979376	0.85927
RABGAP1	chr11	94190001	94340001	93604558	93766020	9.854278	1.6232
GPR21	chr11	94120001	94270001	93690990	93697311	7.600753	1.10254
STRBP	chr11	94340001	94490001	93779449	93924823	11.85756	3.23961
RNAC-GC	chr11	94370001	94520001	93961937	93962008	12.1586	3.09584
CRB2	chr11	94370001	94520001	94006350	94030821	12.1586	3.09584
LHX2	chr11	94370001	94520001	94640045	94659024	12.1586	3.09584
NEK6	chr11	94370001	94520001	94837125	94922060	12.1586	3.09584
PSMB7	chr11	94370001	94520001	94922900	94979454	12.1586	3.09584
RNAC-GC	chr11	94370001	94520001	93961937	93962008	12.1586	3.09584
MYCBP2	chr12	33710001	33860001	33616605	33885679	7.85467	2.367480164
FBXL3	chr12	33750001	33900001	33896229	33919253	7.12068	1.664834787
CLN5	chr12	33780001	33930001	33920524	33931904	6.754983	1.452723271
ACOD1	chr12	33810001	33960001	33959349	33966931	5.673926	1.15025561
SLAIN1	chr12	33640001	33790001	33165618	33217921	5.722692	2.793518952
SCEL	chr12	33710001	33860001	33247740	33372142	7.85467	2.367480164
FBXL3	chr12	33710001	33860001	33896229	33919253	7.85467	2.367480164
CLN5	chr12	33710001	33860001	33920524	33931904	7.85467	2.367480164
ACOD1	chr12	33710001	33860001	33959349	33966931	7.85467	2.367480164
KCTD12	chr12	33710001	33860001	34023639	34029762	7.85467	2.367480164
RNF24	chr13	50590001	50740001	50520829	50637289	6.667194	1.797467437
RNAE-UU	chr13	50450001	50600001	50530729	50530800	5.724039	1.019706585
PANK2	chr13	50590001	50740001	50640389	50669701	6.667194	1.797467437
MIR103	chr13	50590001	50740001	50646831	50646916	6.667194	1.797467437
MAVS	chr13	50590001	50740001	50694748	50707107	6.667194	1.797467437
AP5S1	chr13	50590001	50740001	50734433	50738294	6.667194	1.797467437
CDC25B	chr13	50610001	50760001	50751016	50761010	5.106432	1.334935462
CENPB	chr13	50630001	50780001	50772192	50775019	4.681956	1.153658808
SPEF1	chr13	50630001	50780001	50775106	50781210	4.681956	1.153658808
13H20orf2	chr13	50660001	50810001	50785603	50799453	4.643325	1.074449067
HSPA12B	chr13	50660001	50810001	50799840	50816831	4.643325	1.074449067
BPIFB4	chr13	61720001	61870001	61704744	61730572	4.250796	0.93728656
HAO1	chr13	50450001	50600001	50024601	50086221	5.724039	1.019706585
ADRA1D	chr13	50590001	50740001	50264597	50293882	6.667194	1.797467437
SMOX	chr13	50590001	50740001	50305402	50340974	6.667194	1.797467437
RNF24	chr13	50660001	50810001	50520829	50637289	4.643325	1.074449067
RNAE-UU	chr13	50590001	50740001	50530729	50530800	6.667194	1.797467437
PANK2	chr13	50450001	50600001	50640389	50669701	5.724039	1.019706585
MIR103	chr13	50450001	50600001	50646831	50646916	5.724039	1.019706585
MAVS	chr13	50450001	50600001	50694748	50707107	5.724039	1.019706585
AP5S1	chr13	50580001	50730001	50734433	50738294	5.896336	1.920141848
CDC25B	chr13	50590001	50740001	50751016	50761010	6.667194	1.797467437
CENPB	chr13	50590001	50740001	50772192	50775019	6.667194	1.797467437

SPEF1	chr13	50590001	50740001	50775106	50781210	6.667194	1.797467437
13H20orf2	chr13	50590001	50740001	50785603	50799453	6.667194	1.797467437
HSPA12B	chr13	50590001	50740001	50799840	50816831	6.667194	1.797467437
SIGLEC1	chr13	50590001	50740001	50842091	50860436	6.667194	1.797467437
ADAM33	chr13	50590001	50740001	50865505	50879346	6.667194	1.797467437
GFRA4	chr13	50590001	50740001	50882036	50886744	6.667194	1.797467437
ATRN	chr13	50590001	50740001	50894660	51079673	6.667194	1.797467437
13H20orf1	chr13	50590001	50740001	51139649	51312804	6.667194	1.797467437
RNAS-GG	chr13	50680001	50830001	51246448	51246520	4.139824	1.161308758
NOL4L	chr13	61720001	61870001	61202695	61335042	4.250796	0.93728656
RNAR-CC	chr13	61720001	61870001	61427319	61427391	4.250796	0.93728656
COMMD7	chr13	61720001	61870001	61430474	61455479	4.250796	0.93728656
DNMT3B	chr13	61720001	61870001	61467790	61502090	4.250796	0.93728656
MAPRE1	chr13	61720001	61870001	61508458	61541861	4.250796	0.93728656
SUN5	chr13	61720001	61870001	61602832	61626958	4.250796	0.93728656
BPIFB2	chr13	61720001	61870001	61632481	61650156	4.250796	0.93728656
BPIFB6	chr13	61720001	61870001	61657835	61672584	4.250796	0.93728656
BPIFB3	chr13	61720001	61870001	61681607	61696489	4.250796	0.93728656
BPIFA2	chr13	61720001	61870001	61962034	61971556	4.250796	0.93728656
BPIFA3	chr13	61730001	61880001	62295723	62306454	4.02234	1.045072322
RNAE-UU	chr13	50590001	50740001	50530729	50530800	6.667194	1.797467437
NMNAT2	chr16	63130001	63280001	62912702	63133011	5.708738	1.539642138
SMG7	chr16	63180001	63330001	63132818	63225482	7.661123	1.737211663
NCF2	chr16	63180001	63330001	63224955	63263716	7.661123	1.737211663
ARPC5	chr16	63180001	63330001	63300052	63310232	7.661123	1.737211663
APOBEC4	chr16	63180001	63330001	63322257	63323411	7.661123	1.737211663
LAMC1	chr16	63180001	63330001	62678983	62800726	7.661123	1.737211663
LAMC2	chr16	63180001	63330001	62840547	62910467	7.661123	1.737211663
NMNAT2	chr16	63180001	63330001	62912702	63133011	7.661123	1.737211663
SMG7	chr16	63230001	63380001	63132818	63225482	5.912934	1.123261708
ARPC5	chr16	63150001	63300001	63300052	63310232	6.389922	1.490857194
APOBEC4	chr16	63170001	63320001	63322257	63323411	7.484859	1.564476752
RGL1	chr16	63180001	63330001	63416372	63587678	7.661123	1.737211663
COLGALT	chr16	63180001	63330001	63596114	63715051	7.661123	1.737211663
TSEN15	chr16	63180001	63330001	63723676	63754234	7.661123	1.737211663
LDLRAD4	chr24	43800001	43950001	43647271	43808160	4.197746	1.20640782
FAM210A	chr24	43800001	43950001	43802788	43825139	4.197746	1.20640782
RNMT	chr24	43800001	43950001	43825214	43852257	4.197746	1.20640782
MC5R	chr24	43800001	43950001	43868653	43873520	4.197746	1.20640782
MC2R	chr24	43800001	43950001	43894659	43910455	4.197746	1.20640782
SPIRE1	chr24	43800001	43950001	43245485	43386532	4.197746	1.20640782
CEP76	chr24	43800001	43950001	43395941	43413346	4.197746	1.20640782
PSMG2	chr24	43800001	43950001	43413556	43422907	4.197746	1.20640782
PTPN2	chr24	43800001	43950001	43436686	43502642	4.197746	1.20640782
SEH1L	chr24	43800001	43950001	43534245	43554790	4.197746	1.20640782
CEP192	chr24	43800001	43950001	43556637	43630586	4.197746	1.20640782
CPXM2	chr26	8820001	8970001	8404960	8540659	5.681956	0.886241492

GPR26	chr26	8820001	8970001	8587125	8615287	5.681956	0.886241492
RNAG-GC	chr26	8820001	8970001	9008923	9008995	5.681956	0.886241492
BUB3	chr26	8820001	8970001	9070381	9082657	5.681956	0.886241492
HMX2	chr26	8820001	8970001	9085905	9088891	5.681956	0.886241492
HMX3	chr26	8820001	8970001	9099008	9101028	5.681956	0.886241492
ACADSB	chr26	8820001	8970001	9179479	9214221	5.681956	0.886241492
IKZF5	chr26	8820001	8970001	9214257	9232272	5.681956	0.886241492
PSTK	chr26	8820001	8970001	9240462	9253956	5.681956	0.886241492
RNAR-UC	chr26	8820001	8970001	9364714	9364785	5.681956	0.886241492
HELLS	chr26	35320001	35470001	35557819	35594336	5.244505	0.858559442
TBC1D12	chr26	35320001	35470001	35600390	35696027	5.244505	0.858559442
NOC3L	chr26	35320001	35470001	35718792	35745826	5.244505	0.858559442
PLCE1	chr26	35320001	35470001	35749803	36126653	5.244505	0.858559442
RNAH-AU	chr26	35320001	35470001	35860914	35860985	5.244505	0.858559442
IPMK	chr26	51130001	51280001	50971027	51025641	6.955776	0.875704506
CISD1	chr26	51130001	51280001	51026850	51045714	6.955776	0.875704506
UBE2D1	chr26	51130001	51280001	51089559	51128547	6.955776	0.875704506

Supplementary Table s3:The candidate genes in the selection sweep of UC goats and other cashmere goats

Gene	Chr	Bin_start	Bin_end	Gene_Start	Gene_end	ZFst	g 2 (θπ ratio)
INSIG2	chr2	66620001	66770001	66374212	66386544	4.717596	1.113071
CCDC93	chr2	66620001	66770001	66492663	66600576	4.717596	1.113071
DDX18	chr2	66620001	66770001	66651336	66667824	4.717596	1.113071
RASSF9	chr5	15610001	15760001	15329017	15361750	5.800002	1.053702
NTS	chr5	15610001	15760001	15391221	15402965	5.800002	1.053702
MGAT4C	chr5	15610001	15760001	15533616	15603737	5.800002	1.053702
PLEKHG7	chr5	22830001	22980001	22348785	22417547	6.329986	1.176884
EEA1	chr5	22830001	22980001	22421664	22546190	6.329986	1.176884
TRNAE-CU	chr5	22830001	22980001	22660968	22661040	6.329986	1.176884
NUDT4	chr5	22830001	22980001	22964342	22980670	6.329986	1.176884
UBE2N	chr5	22830001	22980001	22984934	23027066	6.329986	1.176884
MRPL42	chr5	22830001	22980001	23041060	23070803	6.329986	1.176884
SOCS2	chr5	22830001	22980001	23129217	23135562	6.329986	1.176884
CRADD	chr5	22830001	22980001	23232979	23425649	6.329986	1.176884
ALPK1	chr6	13970001	14120001	13456823	13570751	8.838069	1.599863
TIFA	chr6	13970001	14120001	13580947	13589288	8.838069	1.599863
AP1AR	chr6	13970001	14120001	13595155	13624310	8.838069	1.599863
C6H4orf32	chr6	13970001	14120001	13672177	13715799	8.838069	1.599863
PDLIM5	chr6	30790001	30940001	30440595	30679977	5.065607	1.07677
HPGDS	chr6	30790001	30940001	30819572	30845549	5.065607	1.07677
SMARCAD	chr6	30790001	30940001	30850057	30940436	5.065607	1.07677
ATOH1	chr6	30790001	30940001	31242153	31244357	5.065607	1.07677
TRNAS-GG	chr6	30790001	30940001	31293473	31293544	5.065607	1.07677
GRID2	chr6	30790001	30940001	31299697	32930194	5.065607	1.07677
TRNAE-UU	chr6	30790001	30940001	31327448	31327519	5.065607	1.07677
ANTXR2	chr6	95410001	95560001	95056743	95230556	15.15942	2.245108
PRDM8	chr6	95410001	95560001	95336875	95356821	15.15942	2.245108
FGF5	chr6	95410001	95560001	95418992	95440124	15.15942	2.245108
C6H4orf22	chr6	95410001	95560001	95489192	96199327	15.15942	2.245108
TRNAG-CC	chr6	95410001	95560001	95616042	95616114	15.15942	2.245108
VCAN	chr7	27580001	27730001	27133454	27253501	5.027904	1.273321
XRCC4	chr7	27580001	27730001	27358825	27646398	5.027904	1.273321
TMEM167	chr7	27580001	27730001	27646443	27691570	5.027904	1.273321
TMCO6	chr7	59150001	59300001	58720332	58725924	4.480137	1.398587
CD14	chr7	59150001	59300001	58731680	58735123	4.480137	1.398587
SLC35A4	chr7	59150001	59300001	58754826	58759575	4.480137	1.398587
APBB3	chr7	59150001	59300001	58759676	58766097	4.480137	1.398587
SRA1	chr7	59150001	59300001	58766299	58773099	4.480137	1.398587
EIF4EBP3	chr7	59150001	59300001	58773687	58774464	4.480137	1.398587
TRNAC-GC	chr11	94180001	94330001	93961937	93962008	8.360215	1.08025
SLC4A9	chr7	59150001	59300001	58927092	58941009	4.480137	1.398587
HBEGF	chr7	59150001	59300001	58936015	58967277	4.480137	1.398587
PFDN1	chr7	59150001	59300001	58990749	59062745	4.480137	1.398587

CYSTM1	chr7	59150001	59300001	59064021	59134162	4.480137	1.398587
IGIP	chr7	59150001	59300001	59183771	59183983	4.480137	1.398587
PURA	chr7	59150001	59300001	59193011	59204429	4.480137	1.398587
NRG2	chr7	59150001	59300001	59276390	59474026	4.480137	1.398587
PSD2	chr7	59150001	59300001	59477094	59549870	4.480137	1.398587
CXXC5	chr7	59150001	59300001	59617412	59651710	4.480137	1.398587
UBE2D2	chr7	59150001	59300001	59671314	59714771	4.480137	1.398587
DTWD2	chr7	75830001	75980001	75660851	75734513	7.784391	1.432332
DMXL1	chr7	75830001	75980001	75824075	75958422	7.784391	1.432332
TNFAIP8	chr7	75830001	75980001	75975505	76118465	7.784391	1.432332
HSD17B4	chr7	75830001	75980001	76164451	76264055	7.784391	1.432332
FAM170A	chr7	75830001	75980001	76361601	76365214	7.784391	1.432332
RNAW-CC	chrX2	33520001	33670001	33182054	33182125	9.241732	1.111889
PRR16	chr7	76720001	76870001	77276296	77554872	5.303108	1.117342
TLE6	chr7	90200001	90350001	89777457	89789932	4.951197	1.235683
TLE2	chr7	90200001	90350001	89790761	89811070	4.951197	1.235683
AES	chr7	90200001	90350001	89825491	89833731	4.951197	1.235683
GNA11	chr7	90200001	90350001	89858038	89875620	4.951197	1.235683
GNA15	chr7	90200001	90350001	89884034	89904169	4.951197	1.235683
'RNAV-CA	chr7	90200001	90350001	89889797	89889869	4.951197	1.235683
S1PR4	chr7	90200001	90350001	89915136	89919050	4.951197	1.235683
NCLN	chr7	90200001	90350001	89923720	89937645	4.951197	1.235683
CELF5	chr7	90200001	90350001	89948056	89998459	4.951197	1.235683
NFIC	chr7	90200001	90350001	90052700	90125061	4.951197	1.235683
SMIM24	chr7	90200001	90350001	90128780	90131715	4.951197	1.235683
DOHH	chr7	90200001	90350001	90141286	90150323	4.951197	1.235683
FZR1	chr7	90200001	90350001	90164317	90182907	4.951197	1.235683
C7H19orf71	chr7	90200001	90350001	90183012	90188234	4.951197	1.235683
MFSD12	chr7	90200001	90350001	90188702	90197934	4.951197	1.235683
HMG20B	chr7	90200001	90350001	90209112	90215077	4.951197	1.235683
GIPC3	chr7	90200001	90350001	90218057	90221461	4.951197	1.235683
TBXA2R	chr7	90200001	90350001	90224431	90235848	4.951197	1.235683
CACTIN	chr7	90200001	90350001	90239897	90252116	4.951197	1.235683
PIP5K1C	chr7	90200001	90350001	90253792	90301886	4.951197	1.235683
TJP3	chr7	90200001	90350001	90309335	90339632	4.951197	1.235683
APBA3	chr7	90200001	90350001	90339711	90347136	4.951197	1.235683
MRPL54	chr7	90200001	90350001	90348993	90352086	4.951197	1.235683
RAX2	chr7	90200001	90350001	90352183	90354653	4.951197	1.235683
MATK	chr7	90200001	90350001	90358237	90366369	4.951197	1.235683
ZFR2	chr7	90200001	90350001	90382609	90427427	4.951197	1.235683
ATCAY	chr7	90200001	90350001	90440653	90480830	4.951197	1.235683
NMRK2	chr7	90200001	90350001	90489144	90493587	4.951197	1.235683
DAPK3	chr7	90200001	90350001	90504689	90517977	4.951197	1.235683
EEF2	chr7	90200001	90350001	90521523	90530393	4.951197	1.235683
PIAS4	chr7	90200001	90350001	90552914	90577026	4.951197	1.235683
ZBTB7A	chr7	90200001	90350001	90579097	90599740	4.951197	1.235683
MAP2K2	chr7	90200001	90350001	90625518	90646901	4.951197	1.235683

CREB3L3	chr7	90200001	90350001	90675700	90687529	4.951197	1.235683
SIRT6	chr7	90200001	90350001	90687710	90696547	4.951197	1.235683
ANKRD24	chr7	90200001	90350001	90709138	90726622	4.951197	1.235683
EBI3	chr7	90200001	90350001	90726730	90732953	4.951197	1.235683
CCDC94	chr7	90200001	90350001	90735131	90749262	4.951197	1.235683
SHD	chr7	90200001	90350001	90755834	90764186	4.951197	1.235683
TMIGD2	chr7	90200001	90350001	90764906	90772249	4.951197	1.235683
FSD1	chr7	90200001	90350001	90774022	90786104	4.951197	1.235683
STAP2	chr7	90230001	90380001	90786651	90798263	4.463907	1.271817
MPND	chr7	90230001	90380001	90803788	90813427	4.463907	1.271817
SH3GL1	chr7	90240001	90390001	90813591	90840745	4.314688	1.25121
MFSD14B	chr8	300001	450001	194748	313390	7.516694	1.212537
ANXA10	chr8	300001	450001	366581	454508	7.516694	1.212537
DDX60	chr8	300001	450001	469234	525201	7.516694	1.212537
PALLD	chr8	300001	450001	571937	953942	7.516694	1.212537
BCL11A	chr11	43260001	43410001	42802900	42902566	4.657959	1.311925
PAPOLG	chr11	43260001	43410001	43112732	43144983	4.657959	1.311925
REL	chr11	43260001	43410001	43228119	43265438	4.657959	1.311925
PUS10	chr11	43260001	43410001	43279025	43357451	4.657959	1.311925
PEX13	chr11	43260001	43410001	43357512	43391701	4.657959	1.311925
RNAC-GC	chr11	94180001	94330001	93961937	93962008	8.360215	1.08025
KIAA1841	chr11	43260001	43410001	43411369	43468576	4.657959	1.311925
GCFC2	chr11	43260001	43410001	43518809	43594785	4.657959	1.311925
MRPL19	chr11	43260001	43410001	43620250	43631033	4.657959	1.311925
EVA1A	chr11	43260001	43410001	43700750	43781436	4.657959	1.311925
PTCD3	chr11	48770001	48920001	48322191	48350270	4.394665	1.127641
POLR1A	chr11	48830001	48980001	48350620	48437096	6.623518	1.305807
ST3GAL5	chr11	48830001	48980001	48575055	48635258	6.623518	1.305807
ATOH8	chr11	48830001	48980001	48683976	48725280	6.623518	1.305807
GNLY	chr11	48830001	48980001	48786756	48789216	6.623518	1.305807
SFTPB	chr11	48830001	48980001	48852391	48866415	6.623518	1.305807
USP39	chr11	48830001	48980001	48878107	48915123	6.623518	1.305807
C11H2orf6	chr11	48830001	48980001	48918344	48924302	6.623518	1.305807
TMEM150	chr11	48830001	48980001	48926643	48931720	6.623518	1.305807
RNF181	chr11	48830001	48980001	48931843	48944939	6.623518	1.305807
VAMP5	chr11	48830001	48980001	48936693	48945008	6.623518	1.305807
VAMP8	chr11	48830001	48980001	48947446	48950828	6.623518	1.305807
GGCX	chr11	48830001	48980001	48970438	48983617	6.623518	1.305807
MAT2A	chr11	48830001	48980001	48985904	48992675	6.623518	1.305807
SH2D6	chr11	48830001	48980001	49079744	49087050	6.623518	1.305807
CAPG	chr11	48830001	48980001	49108549	49126635	6.623518	1.305807
ELMOD3	chr11	48830001	48980001	49129367	49162874	6.623518	1.305807
RNAG-CC	chr6	95410001	95560001	95616042	95616114	15.15942	2.245108
RETSAT	chr11	48830001	48980001	49163287	49178905	6.623518	1.305807
TGOLN2	chr11	48830001	48980001	49179390	49187399	6.623518	1.305807
TCF7L1	chr11	48830001	48980001	49197164	49389243	6.623518	1.305807
PDCL	chr11	93730001	93880001	93502797	93511273	5.034236	1.190971

RC3H2	chr11	93730001	93880001	93525713	93577732	5.034236	1.190971
ZBTB6	chr11	93730001	93880001	93579682	93587389	5.034236	1.190971
ZBTB26	chr11	93730001	93880001	93589694	93604487	5.034236	1.190971
RABGAP1	chr11	94180001	94330001	93604558	93766020	8.360215	1.08025
GPR21	chr11	93730001	93880001	93690990	93697311	5.034236	1.190971
STRBP	chr11	94180001	94330001	93779449	93924823	8.360215	1.08025
'RNAC-GC.	chr11	94180001	94330001	93961937	93962008	8.360215	1.08025
CRB2	chr11	94180001	94330001	94006350	94030821	8.360215	1.08025
DENND1A	chr11	94180001	94330001	94030061	94571614	8.360215	1.08025
LHX2	chr11	94180001	94330001	94640045	94659024	8.360215	1.08025
NEK6	chr11	94310001	94460001	94837125	94922060	7.432019	3.3312
PSMB7	chr11	94350001	94500001	94922900	94979454	6.539472	3.39406
PAEP	chr11	1.03E+08	1.03E+08	1.03E+08	1.03E+08	7.864747	1.268077
GLT6D1	chr11	1.03E+08	1.03E+08	1.03E+08	1.03E+08	7.864747	1.268077
LCN9	chr11	1.03E+08	1.03E+08	1.03E+08	1.03E+08	7.864747	1.268077
SOHLH1	chr11	1.03E+08	1.03E+08	1.03E+08	1.03E+08	7.864747	1.268077
KCNT1	chr11	1.03E+08	1.03E+08	1.03E+08	1.03E+08	7.864747	1.268077
CAMSAP1	chr11	1.03E+08	1.03E+08	1.03E+08	1.03E+08	7.864747	1.268077
UBAC1	chr11	1.03E+08	1.03E+08	1.03E+08	1.03E+08	7.864747	1.268077
NACC2	chr11	1.03E+08	1.03E+08	1.03E+08	1.03E+08	7.864747	1.268077
C11H9orf69	chr11	1.03E+08	1.03E+08	1.03E+08	1.03E+08	7.864747	1.268077
CCDC187	chr11	1.03E+08	1.03E+08	1.03E+08	1.03E+08	7.864747	1.268077
GPSM1	chr11	1.03E+08	1.03E+08	1.03E+08	1.03E+08	7.864747	1.268077
DNLZ	chr11	1.03E+08	1.03E+08	1.03E+08	1.03E+08	7.864747	1.268077
CARD9	chr11	1.03E+08	1.03E+08	1.03E+08	1.03E+08	7.864747	1.268077
SNAPC4	chr11	1.03E+08	1.03E+08	1.03E+08	1.03E+08	7.864747	1.268077
SDCCAG3	chr11	1.03E+08	1.03E+08	1.03E+08	1.03E+08	7.864747	1.268077
PMPCA	chr11	1.03E+08	1.03E+08	1.03E+08	1.03E+08	7.864747	1.268077
INPP5E	chr11	1.03E+08	1.03E+08	1.03E+08	1.03E+08	7.864747	1.268077
SEC16A	chr11	1.03E+08	1.03E+08	1.03E+08	1.03E+08	7.864747	1.268077
NOTCH1	chr11	1.03E+08	1.03E+08	1.03E+08	1.03E+08	7.864747	1.268077
EGFL7	chr11	1.03E+08	1.03E+08	1.03E+08	1.04E+08	7.864747	1.268077
MIR126	chr11	1.03E+08	1.03E+08	1.04E+08	1.04E+08	7.864747	1.268077
AGPAT2	chr11	1.03E+08	1.03E+08	1.04E+08	1.04E+08	7.864747	1.268077
FAM69B	chr11	1.03E+08	1.03E+08	1.04E+08	1.04E+08	7.864747	1.268077
ABO	chr11	1.03E+08	1.03E+08	1.04E+08	1.04E+08	7.864747	1.268077
SURF6	chr11	1.03E+08	1.03E+08	1.04E+08	1.04E+08	7.864747	1.268077
MED22	chr11	1.03E+08	1.03E+08	1.04E+08	1.04E+08	7.745723	1.268608
RPL7A	chr11	1.03E+08	1.03E+08	1.04E+08	1.04E+08	7.745723	1.268608
SURF2	chr11	1.03E+08	1.03E+08	1.04E+08	1.04E+08	7.724125	1.264893
SURF4	chr11	1.03E+08	1.03E+08	1.04E+08	1.04E+08	7.724125	1.264893
STKLD1	chr11	1.03E+08	1.03E+08	1.04E+08	1.04E+08	7.556998	1.226179
REXO4	chr11	1.03E+08	1.03E+08	1.04E+08	1.04E+08	7.464061	1.229936
ADAMTS13	chr11	1.03E+08	1.03E+08	1.04E+08	1.04E+08	7.183195	1.212109
CACFD1	chr11	1.03E+08	1.03E+08	1.04E+08	1.04E+08	6.049875	1.116282
TNFRSF19	chr12	52760001	52910001	52252542	52348993	4.981813	1.192716
MIPEP	chr12	52760001	52910001	52377621	52457448	4.981813	1.192716

C1QTNF9	chr12	52760001	52910001	52462240	52469963	4.981813	1.192716
SPATA13	chr12	52760001	52910001	52473037	52579821	4.981813	1.192716
AMER2	chr12	52760001	52910001	52766430	52768477	4.981813	1.192716
MTMR6	chr12	52760001	52910001	52804352	52824861	4.981813	1.192716
NUP58	chr12	52760001	52910001	52830226	52862819	4.981813	1.192716
RNAG-UC	chr12	52760001	52910001	52877526	52877598	4.981813	1.192716
ATP8A2	chr12	52760001	52910001	52884246	53350170	4.981813	1.192716
MTDH	chr14	16010001	16160001	15663731	15721499	4.696795	1.75102
LAPTM4B	chr14	16010001	16160001	15788488	15854656	4.696795	1.75102
MATN2	chr14	16010001	16160001	15872116	16041603	4.696795	1.75102
RPL30	chr14	16010001	16160001	16048416	16051780	4.696795	1.75102
ERICH5	chr14	16010001	16160001	16063489	16086802	4.696795	1.75102
RIDA	chr14	16010001	16160001	16096003	16104730	4.696795	1.75102
POP1	chr14	16010001	16160001	16104883	16139688	4.696795	1.75102
NIPAL2	chr14	16010001	16160001	16173131	16256193	4.696795	1.75102
KCNS2	chr14	16010001	16160001	16394213	16400041	4.696795	1.75102
STK3	chr14	16010001	16160001	16433649	16744347	4.696795	1.75102
DCLK3	chr22	11010001	11160001	10587039	10629524	4.713277	1.356235
GOLGA4	chr22	11010001	11160001	10653112	10757227	4.713277	1.356235
ITGA9	chr22	11010001	11160001	10780757	11147531	4.713277	1.356235
CTDSPL	chr22	11010001	11160001	11187226	11311455	4.713277	1.356235
MIR26A	chr22	11010001	11160001	11298067	11298156	4.713277	1.356235
VILL	chr22	11010001	11160001	11316379	11337078	4.713277	1.356235
PLCD1	chr22	11010001	11160001	11337411	11359212	4.713277	1.356235
DLEC1	chr22	11010001	11160001	11369750	11460169	4.713277	1.356235
ACAA1	chr22	11010001	11160001	11460323	11484652	4.713277	1.356235
MYD88	chr22	11010001	11160001	11484772	11490413	4.713277	1.356235
OXSR1	chr22	11010001	11160001	11513751	11598867	4.713277	1.356235
BTRC	chr26	29820001	29970001	29360210	29542837	5.365387	1.075789
LBX1	chr26	29820001	29970001	29649006	29652810	5.365387	1.075789
TLX1	chr26	29820001	29970001	29716272	29721774	5.365387	1.075789
KAZALD1	chr26	29820001	29970001	29780565	29784209	5.365387	1.075789
SFXN3	chr26	29820001	29970001	29805091	29812297	5.365387	1.075789
PDZD7	chr26	29820001	29970001	29813473	29833314	5.365387	1.075789
LZTS2	chr26	29820001	29970001	29833983	29844452	5.365387	1.075789
C26H10orf2	chr26	29820001	29970001	29846333	29851711	5.365387	1.075789
MRPL43	chr26	29820001	29970001	29851736	29853327	5.365387	1.075789
SEMA4G	chr26	29820001	29970001	29853719	29867663	5.365387	1.075789
SLF2	chr26	29820001	29970001	29872724	29914824	5.365387	1.075789
PAX2	chr26	29820001	29970001	29995533	30085588	5.365387	1.075789
HIF1AN	chr26	29820001	29970001	30240811	30255353	5.365387	1.075789
NDUFB8	chr26	29820001	29970001	30260956	30266363	5.365387	1.075789
SEC31B	chr26	29820001	29970001	30269322	30300136	5.365387	1.075789
WNT8B	chr26	29820001	29970001	30304225	30335373	5.365387	1.075789
SCD	chr26	29830001	29980001	30404780	30415917	5.226066	1.074017
PKD2L1	chr26	30220001	30370001	30438465	30482082	4.767839	1.182486
CWF19L1	chr26	30220001	30370001	30497374	30521868	4.767839	1.182486

CHUK	chr26	30220001	30370001	30524704	30565615	4.767839	1.182486
ERLIN1	chr26	30220001	30370001	30568325	30606628	4.767839	1.182486
CPN1	chr26	30220001	30370001	30664670	30693243	4.767839	1.182486
DNMBP	chr26	30220001	30370001	30708273	30822313	4.767839	1.182486
'RNAC-GC	chr11	94180001	94330001	93961937	93962008	8.360215	1.08025
KIF4A	chrX1	11720001	11870001	11177125	11304219	4.702624	1.582698
PDZD11	chrX1	11720001	11870001	11304300	11307103	4.702624	1.582698
ARR3	chrX1	11740001	11890001	11313524	11326636	5.109391	1.465974
P2RY4	chrX1	11740001	11890001	11336935	11338033	5.109391	1.465974
AWAT1	chrX1	11740001	11890001	11348658	11355477	5.109391	1.465974
DGAT2L6	chrX1	11740001	11890001	11403406	11425115	5.109391	1.465974
IGBP1	chrX1	11740001	11890001	11436412	11481694	5.109391	1.465974
OTUD6A	chrX1	11740001	11890001	11521708	11522580	5.109391	1.465974
AWAT2	chrX1	11740001	11890001	11544904	11556261	5.109391	1.465974
EDA	chrX1	11740001	11890001	11557022	11895599	5.109391	1.465974
CDX4	chrX1	11740001	11890001	12054961	12063592	5.109391	1.465974
FAM155B	chrX1	11740001	11890001	12074820	12103927	5.109391	1.465974
PJA1	chrX1	11740001	11890001	12110252	12115011	5.109391	1.465974
AR	chrX1	13800001	13950001	13729623	13934065	7.526382	2.276666
'RNAC-GC	chr11	94180001	94330001	93961937	93962008	8.360215	1.08025
GRIA3	chrX2	15570001	15720001	15984771	16285966	5.73919	1.063011
TRPC5	chrX2	32390001	32540001	32157960	32499654	4.689455	1.056817
TRPC5OS	chrX2	32390001	32540001	32198714	32199046	4.689455	1.056817
'RNAE-UU	chr6	30790001	30940001	31327448	31327519	5.065607	1.07677
DCX	chrX2	32390001	32540001	32724034	32763972	4.689455	1.056817
CAPN6	chrX2	32390001	32540001	32796791	32821641	4.689455	1.056817
PAK3	chrX2	32390001	32540001	32843960	32971791	4.689455	1.056817
RNAW-CC	chrX2	33520001	33670001	33182054	33182125	9.241732	1.111889
RGAG1	chrX2	33520001	33670001	33623413	33629514	9.241732	1.111889
ACSL4	chrX2	33520001	33670001	33813892	33887538	9.241732	1.111889
KCNE5	chrX2	33520001	33670001	33907229	33908705	9.241732	1.111889
GPR119	chrX2	33530001	33680001	34098328	34099849	8.907226	1.092282

Supplementary Table s4：Annotation of candidate genes in UC goats and other cashmere goats to pe

Input ID	Gene ID	Type	Tax ID	Homologen	Homologen	Gene Symb	Descripti	Biologica
INSIG2	51141	symbol	H. sapiens	51141	H. sapiens	INSIG2	insulin indu	GO:006036
CCDC93	54520	symbol	H. sapiens	54520	H. sapiens	CCDC93	coiled-coil	GO:199012
DDX18	8886	symbol	H. sapiens	8886	H. sapiens	DDX18	DEAD-box	GO:000046
RASSF9	9182	symbol	H. sapiens	9182	H. sapiens	RASSF9	Ras associa	GO:000660
NTS	4922	symbol	H. sapiens	4922	H. sapiens	NTS	neurotensi	GO:000718
MGAT4C	25834	symbol	H. sapiens	25834	H. sapiens	MGAT4C	MGAT4 fan	GO:000648
PLEKHG7	440107	symbol	H. sapiens	440107	H. sapiens	PLEKHG7	pleckstrin homology ar	
EEA1	8411	symbol	H. sapiens	8411	H. sapiens	EEA1	early endo:	GO:001618
TRNAE-CU(	108348643	symbol	R. norvegicus			None	None	None
NUDT4	11163	symbol	H. sapiens	11163	H. sapiens	NUDT4	nudix hydr	GO:190191
UBE2N	7334	symbol	H. sapiens	7334	H. sapiens	UBE2N	ubiquitin c	GO:007042
MRPL42	28977	symbol	H. sapiens	28977	H. sapiens	MRPL42	mitochond	GO:007012
SOCS2	8835	symbol	H. sapiens	8835	H. sapiens	SOCS2	suppressor	GO:006039
CRADD	8738	symbol	H. sapiens	8738	H. sapiens	CRADD	CASP2 and	GO:000697
ALPK1	80216	symbol	H. sapiens	80216	H. sapiens	ALPK1	alpha kinas	GO:000275
TIFA	92610	symbol	H. sapiens	92610	H. sapiens	TIFA	TRAF intera	GO:000275
AP1AR	55435	symbol	H. sapiens	55435	H. sapiens	AP1AR	adaptor rel	GO:003431
C6H4orf32						None	None	None
PDLIM5	10611	symbol	H. sapiens	10611	H. sapiens	PDLIM5	PDZ and LII	GO:006100
HPGDS	27306	symbol	H. sapiens	27306	H. sapiens	HPGDS	hematopoi	GO:001937
SMARCAD1	56916	symbol	H. sapiens	56916	H. sapiens	SMARCAD1	SWI/SNF-re	GO:007093
ATOH1	474	symbol	H. sapiens	474	H. sapiens	ATOH1	atonal bHL	GO:004560
TRNAS-GG/	108180566	symbol	D. rerio			None	None	None
GRID2	2895	symbol	H. sapiens	2895	H. sapiens	GRID2	glutamate i	GO:002170
TRNAE-UU(	107985602	symbol	H. sapiens	107985602	H. sapiens	TRNAE-UU(	transfer RNA glutamic a	
ANTXR2	118429	symbol	H. sapiens	118429	H. sapiens	ANTXR2	ANTXR cell	GO:190199
PRDM8	56978	symbol	H. sapiens	56978	H. sapiens	PRDM8	PR/SET dor	GO:001400
FGF5	2250	symbol	H. sapiens	2250	H. sapiens	FGF5	fibroblast g	GO:002301
C6H4orf22						None	None	None
TRNAG-CC(	107985600	symbol	H. sapiens	107985600	H. sapiens	TRNAG-CC(	transfer RNA glycine (a	
VCAN	1462	symbol	H. sapiens	1462	H. sapiens	VCAN	versican	GO:003020
XRCC4	7518	symbol	H. sapiens	7518	H. sapiens	XRCC4	X-ray repai	GO:007571
TMEM167/	153339	symbol	H. sapiens	153339	H. sapiens	TMEM167/	transmemk	GO:000930
TMCO6	55374	symbol	H. sapiens	55374	H. sapiens	TMCO6	transmemk	GO:000660
CD14	929	symbol	H. sapiens	929	H. sapiens	CD14	CD14 mole	GO:003412
SLC35A4	113829	symbol	H. sapiens	113829	H. sapiens	SLC35A4	solute carri	GO:009048
APBB3	10307	symbol	H. sapiens	10307	H. sapiens	APBB3	amyloid be	GO:000635
SRA1	10011	symbol	H. sapiens	10011	H. sapiens	SRA1	steroid rec	GO:004566
EIF4EBP3	8637	symbol	H. sapiens	8637	H. sapiens	EIF4EBP3	eukaryotic	GO:004594
TRNAC-GC/	107985606	symbol	H. sapiens	107985606	H. sapiens	TRNAC-GC/	transfer RNA cysteine (	
SLC4A9	83697	symbol	H. sapiens	83697	H. sapiens	SLC4A9	solute carri	GO:005145
HBEGF	1839	symbol	H. sapiens	1839	H. sapiens	HBEGF	heparin bir	GO:005154
PFDN1	5201	symbol	H. sapiens	5201	H. sapiens	PFDN1	prefoldin sı	GO:000645
CYSTM1	84418	symbol	H. sapiens	84418	H. sapiens	CYSTM1	cysteine ric	GO:004331
IGIP	492311	symbol	H. sapiens	492311	H. sapiens	IGIP	IgA inducing protein	

PURA	5813	symbol	H. sapiens	5813	H. sapiens	PURA	purine rich	GO:009896
NRG2	9542	symbol	H. sapiens	9542	H. sapiens	NRG2	neuregulin	GO:003812
PSD2	84249	symbol	H. sapiens	84249	H. sapiens	PSD2	pleckstrin a	GO:003201
CXXC5	51523	symbol	H. sapiens	51523	H. sapiens	CXXC5	CXXC finge	GO:004312
UBE2D2	7322	symbol	H. sapiens	7322	H. sapiens	UBE2D2	ubiquitin co	GO:003566
DTWD2	285605	symbol	H. sapiens	285605	H. sapiens	DTWD2	DTW domain containin	
DMXL1	1657	symbol	H. sapiens	1657	H. sapiens	DMXL1	Dmx like 1	GO:000703
TNFAIP8	25816	symbol	H. sapiens	25816	H. sapiens	TNFAIP8	TNF alpha i	GO:004315
HSD17B4	3295	symbol	H. sapiens	3295	H. sapiens	HSD17B4	hydroxyste	GO:003611
FAM170A	340069	symbol	H. sapiens	340069	H. sapiens	FAM170A	family with	GO:004589
TRNAW-CC	10834863C	symbol	R. norvegicus			None	None	None
PRR16	51334	symbol	H. sapiens	51334	H. sapiens	PRR16	proline rich	GO:004579
TLE6	79816	symbol	H. sapiens	79816	H. sapiens	TLE6	TLE family	GO:006013
TLE2	7089	symbol	H. sapiens	7089	H. sapiens	TLE2	TLE family	GO:190483
AES	166	gene_syno	H. sapiens	166	H. sapiens	TLE5	TLE family	GO:200021
GNA11	2767	symbol	H. sapiens	2767	H. sapiens	GNA11	G protein s	GO:004563
GNA15	2769	symbol	H. sapiens	2769	H. sapiens	GNA15	G protein s	GO:000720
TRNAV-CA(	107985608	symbol	H. sapiens	107985608	H. sapiens	TRNAV-CA(	transfer RNA valine (ar	
S1PR4	8698	symbol	H. sapiens	8698	H. sapiens	S1PR4	sphingosin	GO:000720
NCLN	56926	symbol	H. sapiens	56926	H. sapiens	NCLN	nicalin	GO:006163
CELF5	60680	symbol	H. sapiens	60680	H. sapiens	CELF5	CUGBP Ela	GO:000637
NFIC	4782	symbol	H. sapiens	4782	H. sapiens	NFIC	nuclear fac	GO:004247
SMIM24	284422	symbol	H. sapiens	284422	H. sapiens	SMIM24	small integ	GO:000815
DOHH	83475	symbol	H. sapiens	83475	H. sapiens	DOHH	deoxyhypu	GO:000861
FZR1	51343	symbol	H. sapiens	51343	H. sapiens	FZR1	fizzy and ce	GO:007242
C7H19orf71						None	None	None
MFSD12	126321	symbol	H. sapiens	126321	H. sapiens	MFSD12	major facili	GO:004802
HMG20B	10362	symbol	H. sapiens	10362	H. sapiens	HMG20B	high mobili	GO:003323
GIPC3	126326	symbol	H. sapiens	126326	H. sapiens	GIPC3	GIPC PDZ domain conta	
TBXA2R	6915	symbol	H. sapiens	6915	H. sapiens	TBXA2R	thromboxa	GO:009005
CACTIN	58509	symbol	H. sapiens	58509	H. sapiens	CACTIN	cactin, splic	GO:003166
PIP5K1C	23396	symbol	H. sapiens	23396	H. sapiens	PIP5K1C	phosphatid	GO:003059
TJP3	27134	symbol	H. sapiens	27134	H. sapiens	TJP3	tight junction protein 3	
APBA3	9546	symbol	H. sapiens	9546	H. sapiens	APBA3	amyloid be	GO:000170
MRPL54	116541	symbol	H. sapiens	116541	H. sapiens	MRPL54	mitochond	GO:007012
RAX2	84839	symbol	H. sapiens	84839	H. sapiens	RAX2	retina and	GO:000760
MATK	4145	symbol	H. sapiens	4145	H. sapiens	MATK	megakaryo	GO:003812
ZFR2	23217	symbol	H. sapiens	23217	H. sapiens	ZFR2	zinc finger RNA binding	
ATCAY	85300	symbol	H. sapiens	85300	H. sapiens	ATCAY	ATCAY kine	GO:200021
NMRK2	27231	symbol	H. sapiens	27231	H. sapiens	NMRK2	nicotinami	GO:000943
DAPK3	1613	symbol	H. sapiens	1613	H. sapiens	DAPK3	death asso	GO:004351
EEF2	1938	symbol	H. sapiens	1938	H. sapiens	EEF2	eukaryotic	GO:199041
PIAS4	51588	symbol	H. sapiens	51588	H. sapiens	PIAS4	protein inh	GO:190217
ZBTB7A	51341	symbol	H. sapiens	51341	H. sapiens	ZBTB7A	zinc finger	GO:006076
MAP2K2	5605	symbol	H. sapiens	5605	H. sapiens	MAP2K2	mitogen-ac	GO:19038C
CREB3L3	84699	symbol	H. sapiens	84699	H. sapiens	CREB3L3	cAMP resp	GO:199044
SIRT6	51548	symbol	H. sapiens	51548	H. sapiens	SIRT6	sirtuin 6	GO:190554

ANKRD24	170961	symbol	H. sapiens	170961	H. sapiens	ANKRD24	ankyrin repeat domain	
EBI3	10148	symbol	H. sapiens	10148	H. sapiens	EBI3	Epstein-Bar	GO:004664
CCDC94	55702	gene_syno	H. sapiens	55702	H. sapiens	YJU2	YJU2 splicir	GO:004351
SHD	56961	symbol	H. sapiens	56961	H. sapiens	SHD	Src homology 2 domair	
TMIGD2	126259	symbol	H. sapiens	126259	H. sapiens	TMIGD2	transmemb	GO:004210
FSD1	79187	symbol	H. sapiens	79187	H. sapiens	FSD1	fibronectin	GO:006023
STAP2	55620	symbol	H. sapiens	55620	H. sapiens	STAP2	signal trans	GO:004253
MPND	84954	symbol	H. sapiens	84954	H. sapiens	MPND	MPN doma	GO:000650
SH3GL1	6455	symbol	H. sapiens	6455	H. sapiens	SH3GL1	SH3 domai	GO:001619
MFSD14B	84641	symbol	H. sapiens	84641	H. sapiens	MFSD14B	major facili	GO:005508
ANXA10	11199	symbol	H. sapiens	11199	H. sapiens	ANXA10	annexin A10	
DDX60	55601	symbol	H. sapiens	55601	H. sapiens	DDX60	DExD/H-bo	GO:190024
PALLD	23022	symbol	H. sapiens	23022	H. sapiens	PALLD	palladin, cy	GO:000333
BCL11A	53335	symbol	H. sapiens	53335	H. sapiens	BCL11A	BAF chrom	GO:190480
PAPOLG	64895	symbol	H. sapiens	64895	H. sapiens	PAPOLG	poly(A) pol	GO:000637
REL	5966	symbol	H. sapiens	5966	H. sapiens	REL	REL proto-c	GO:003268
PUS10	150962	symbol	H. sapiens	150962	H. sapiens	PUS10	pseudouric	GO:003111
PEX13	5194	symbol	H. sapiens	5194	H. sapiens	PEX13	peroxisoma	GO:001656
KIAA1841	84542	symbol	H. sapiens	84542	H. sapiens	KIAA1841	KIAA1841	
GCFC2	6936	symbol	H. sapiens	6936	H. sapiens	GCFC2	GC-rich sec	GO:000024
MRPL19	9801	symbol	H. sapiens	9801	H. sapiens	MRPL19	mitochond	GO:007012
EVA1A	84141	symbol	H. sapiens	84141	H. sapiens	EVA1A	eva-1 hom	GO:004368
PTCD3	55037	symbol	H. sapiens	55037	H. sapiens	PTCD3	pentatricop	GO:007012
POLR1A	25885	symbol	H. sapiens	25885	H. sapiens	POLR1A	RNA polym	GO:190475
ST3GAL5	8869	symbol	H. sapiens	8869	H. sapiens	ST3GAL5	ST3 beta-ga	GO:000157
ATOH8	84913	symbol	H. sapiens	84913	H. sapiens	ATOH8	atonal bHL	GO:004560
GNLY	10578	symbol	H. sapiens	10578	H. sapiens	GNLY	granulysin	GO:000696
SFTPB	6439	symbol	H. sapiens	6439	H. sapiens	SFTPB	surfactant	GO:000758
USP39	10713	symbol	H. sapiens	10713	H. sapiens	USP39	ubiquitin sp	GO:000024
C11H2orf68						None	None	None
TMEM150A	129303	symbol	H. sapiens	129303	H. sapiens	TMEM150A	transmemb	GO:004685
RNF181	51255	symbol	H. sapiens	51255	H. sapiens	RNF181	ring finger	GO:005186
VAMP5	10791	symbol	H. sapiens	10791	H. sapiens	VAMP5	vesicle ass	GO:004300
VAMP8	8673	symbol	H. sapiens	8673	H. sapiens	VAMP8	vesicle ass	GO:190359
GGCX	2677	symbol	H. sapiens	2677	H. sapiens	GGCX	gamma-glu	GO:001718
MAT2A	4144	symbol	H. sapiens	4144	H. sapiens	MAT2A	methionine	GO:000655
SH2D6	284948	symbol	H. sapiens	284948	H. sapiens	SH2D6	SH2 domai	GO:000716
CAPG	822	symbol	H. sapiens	822	H. sapiens	CAPG	capping act	GO:005101
ELMOD3	84173	symbol	H. sapiens	84173	H. sapiens	ELMOD3	ELMO dom	GO:004354
RETSAT	54884	symbol	H. sapiens	54884	H. sapiens	RETSAT	retinol satu	GO:004257
TGOLN2	10618	symbol	H. sapiens	10618	H. sapiens	TGOLN2	trans-golgi	GO:004368
TCF7L1	83439	symbol	H. sapiens	83439	H. sapiens	TCF7L1	transcriptic	GO:190483
PDCL	5082	symbol	H. sapiens	5082	H. sapiens	PDCL	phosducin	GO:006108
RC3H2	54542	symbol	H. sapiens	54542	H. sapiens	RC3H2	ring finger	GO:200032
ZBTB6	10773	symbol	H. sapiens	10773	H. sapiens	ZBTB6	zinc finger	GO:000012
ZBTB26	57684	symbol	H. sapiens	57684	H. sapiens	ZBTB26	zinc finger	GO:000012
RABGAP1	23637	symbol	H. sapiens	23637	H. sapiens	RABGAP1	RAB GTPas	GO:190201

GPR21	2844	symbol	H. sapiens	2844	H. sapiens	GPR21	G protein-c	GO:004662
STRBP	55342	symbol	H. sapiens	55342	H. sapiens	STRBP	spermatid	GO:000728
CRB2	286204	symbol	H. sapiens	286204	H. sapiens	CRB2	crumbs cel	GO:005511
DENND1A	57706	symbol	H. sapiens	57706	H. sapiens	DENND1A	DENN dom	GO:003248
LHX2	9355	symbol	H. sapiens	9355	H. sapiens	LHX2	LIM homec	GO:004519
NEK6	10783	symbol	H. sapiens	10783	H. sapiens	NEK6	NIMA relat	GO:000707
PSMB7	5695	symbol	H. sapiens	5695	H. sapiens	PSMB7	proteasom	GO:000222
PAEP	5047	symbol	H. sapiens	5047	H. sapiens	PAEP	progestage	GO:190249
GLT6D1	360203	symbol	H. sapiens	360203	H. sapiens	GLT6D1	glycosyltra	GO:003025
LCN9	392399	symbol	H. sapiens	392399	H. sapiens	LCN9	lipocalin 9	
SOHLH1	402381	symbol	H. sapiens	402381	H. sapiens	SOHLH1	spermatog	GO:000999
KCNT1	57582	symbol	H. sapiens	57582	H. sapiens	KCNT1	potassium	GO:007180
CAMSAP1	157922	symbol	H. sapiens	157922	H. sapiens	CAMSAP1	calmodulin	GO:000702
UBAC1	10422	symbol	H. sapiens	10422	H. sapiens	UBAC1	UBA domai	GO:001656
NACC2	138151	symbol	H. sapiens	138151	H. sapiens	NACC2	NACC famil	GO:190047
C11H9orf69						None	None	None
CCDC187	399693	symbol	H. sapiens	399693	H. sapiens	CCDC187	coiled-coil	GO:003445
GPSM1	26086	symbol	H. sapiens	26086	H. sapiens	GPSM1	G protein s	GO:190509
DNLZ	728489	symbol	H. sapiens	728489	H. sapiens	DNLZ	DNL-type z	GO:003015
CARD9	64170	symbol	H. sapiens	64170	H. sapiens	CARD9	caspase rec	GO:000222
SNAPC4	6621	symbol	H. sapiens	6621	H. sapiens	SNAPC4	small nucle	GO:004279
SDCCAG3	10807	gene_syno	H. sapiens	10807	H. sapiens	ENTR1	endosome	GO:190356
PMPCA	23203	symbol	H. sapiens	23203	H. sapiens	PMPCA	peptidase,	GO:000662
INPP5E	56623	symbol	H. sapiens	56623	H. sapiens	INPP5E	inositol pol	GO:190356
SEC16A	9919	symbol	H. sapiens	9919	H. sapiens	SEC16A	SEC16 hom	GO:004300
NOTCH1	4851	symbol	H. sapiens	4851	H. sapiens	NOTCH1	notch rece	GO:000327
EGFL7	51162	symbol	H. sapiens	51162	H. sapiens	EGFL7	EGF like do	GO:000157
MIR126	406913	symbol	H. sapiens	406913	H. sapiens	MIR126	microRNA 1	GO:190358
AGPAT2	10555	symbol	H. sapiens	10555	H. sapiens	AGPAT2	1-acylglyce	GO:001602
FAM69B	138311	gene_syno	H. sapiens	138311	H. sapiens	DIPK1B	divergent protein kinas	
ABO	28	symbol	H. sapiens	28	H. sapiens	ABO	ABO, alpha	GO:003025
SURF6	6838	symbol	H. sapiens	6838	H. sapiens	SURF6	surfeit 6	GO:004227
MED22	6837	symbol	H. sapiens	6837	H. sapiens	MED22	mediator c	GO:000635
RPL7A	6130	symbol	H. sapiens	6130	H. sapiens	RPL7A	ribosomal	GO:000661
SURF2	6835	symbol	H. sapiens	6835	H. sapiens	SURF2	surfeit 2	GO:000815
SURF4	6836	symbol	H. sapiens	6836	H. sapiens	SURF4	surfeit 4	GO:003449
STKLD1	169436	symbol	H. sapiens	169436	H. sapiens	STKLD1	serine/thre	GO:000646
REXO4	57109	symbol	H. sapiens	57109	H. sapiens	REXO4	REX4 homc	GO:000073
ADAMTS13	11093	symbol	H. sapiens	11093	H. sapiens	ADAMTS13	ADAM met	GO:003586
CACFD1	11094	symbol	H. sapiens	11094	H. sapiens	CACFD1	calcium cha	GO:001619
TNFRSF19	55504	symbol	H. sapiens	55504	H. sapiens	TNFRSF19	TNF recept	GO:004633
MIPEP	4285	symbol	H. sapiens	4285	H. sapiens	MIPEP	mitochond	GO:000662
C1QTNF9	338872	symbol	H. sapiens	338872	H. sapiens	C1QTNF9	C1q and TN	GO:000716
SPATA13	221178	symbol	H. sapiens	221178	H. sapiens	SPATA13	spermatog	GO:003003
AMER2	219287	symbol	H. sapiens	219287	H. sapiens	AMER2	APC memb	GO:009009
MTMR6	9107	symbol	H. sapiens	9107	H. sapiens	MTMR6	myotubula	GO:004685
NUP58	9818	symbol	H. sapiens	9818	H. sapiens	NUP58	nucleopori	GO:000640

TRNAG-UC	108350327	symbol	R. norvegicus			None	None	None
ATP8A2	51761	symbol	H. sapiens	51761	H. sapiens	ATP8A2	ATPase pho	GO:006109
MTDH	92140	symbol	H. sapiens	92140	H. sapiens	MTDH	metadherin	GO:003166
LAPTM4B	55353	symbol	H. sapiens	55353	H. sapiens	LAPTM4B	lysosomal p	GO:190516
MATN2	4147	symbol	H. sapiens	4147	H. sapiens	MATN2	matrilin 2	GO:000342
RPL30	6156	symbol	H. sapiens	6156	H. sapiens	RPL30	ribosomal p	GO:190457
ERICH5	203111	symbol	H. sapiens	203111	H. sapiens	ERICH5	glutamate rich 5	
RIDA	10247	symbol	H. sapiens	10247	H. sapiens	RIDA	reactive int	GO:001951
POP1	10940	symbol	H. sapiens	10940	H. sapiens	POP1	POP1 homo	GO:000168
NIPAL2	79815	symbol	H. sapiens	79815	H. sapiens	NIPAL2	NIPA like d	GO:190383
KCNS2	3788	symbol	H. sapiens	3788	H. sapiens	KCNS2	potassium	GO:190225
STK3	6788	symbol	H. sapiens	6788	H. sapiens	STK3	serine/thre	GO:006080
DCLK3	85443	symbol	H. sapiens	85443	H. sapiens	DCLK3	doublecort	GO:190018
GOLGA4	2803	symbol	H. sapiens	2803	H. sapiens	GOLGA4	golgin A4	GO:004577
ITGA9	3680	symbol	H. sapiens	3680	H. sapiens	ITGA9	integrin sul	GO:003059
CTDSPL	10217	symbol	H. sapiens	10217	H. sapiens	CTDSPL	CTD small p	GO:200013
MIR26A	407015	gene_syno	H. sapiens	407015	H. sapiens	MIR26A1	microRNA 2	GO:190360
VILL	50853	symbol	H. sapiens	50853	H. sapiens	VILL	villin like	GO:005169
PLCD1	5333	symbol	H. sapiens	5333	H. sapiens	PLCD1	phospholip	GO:005120
DLEC1	9940	symbol	H. sapiens	9940	H. sapiens	DLEC1	DLEC1 cilia	GO:000828
ACAA1	30	symbol	H. sapiens	30	H. sapiens	ACAA1	acetyl-CoA	GO:001012
MYD88	4615	symbol	H. sapiens	4615	H. sapiens	MYD88	MYD88 inn	GO:003416
OXSR1	9943	symbol	H. sapiens	9943	H. sapiens	OXSR1	oxidative st	GO:001082
BTRC	8945	symbol	H. sapiens	8945	H. sapiens	BTRC	beta-transc	GO:000222
LBX1	10660	symbol	H. sapiens	10660	H. sapiens	LBX1	ladybird ho	GO:002192
TLX1	3195	symbol	H. sapiens	3195	H. sapiens	TLX1	T cell leuke	GO:004594
KAZALD1	81621	symbol	H. sapiens	81621	H. sapiens	KAZALD1	Kazal type s	GO:003019
SFXN3	81855	symbol	H. sapiens	81855	H. sapiens	SFXN3	sideroflexin	GO:000673
PDZD7	79955	symbol	H. sapiens	79955	H. sapiens	PDZD7	PDZ domai	GO:006008
LZTS2	84445	symbol	H. sapiens	84445	H. sapiens	LZTS2	leucine zip	GO:006068
C26H10orf2						None	None	None
MRPL43	84545	symbol	H. sapiens	84545	H. sapiens	MRPL43	mitochond	GO:007012
SEMA4G	57715	symbol	H. sapiens	57715	H. sapiens	SEMA4G	semaphorin	GO:004884
SLF2	55719	symbol	H. sapiens	55719	H. sapiens	SLF2	SMC5-SMC	GO:003418
PAX2	5076	symbol	H. sapiens	5076	H. sapiens	PAX2	paired box	GO:200059
HIF1AN	55662	symbol	H. sapiens	55662	H. sapiens	HIF1AN	hypoxia inc	GO:006142
NDUFB8	4714	symbol	H. sapiens	4714	H. sapiens	NDUFB8	NADH:ubiq	GO:000612
SEC31B	25956	symbol	H. sapiens	25956	H. sapiens	SEC31B	SEC31 hom	GO:009011
WNT8B	7479	symbol	H. sapiens	7479	H. sapiens	WNT8B	Wnt family	GO:004826
SCD	6319	symbol	H. sapiens	6319	H. sapiens	SCD	stearoyl-Co	GO:004694
PKD2L1	9033	symbol	H. sapiens	9033	H. sapiens	PKD2L1	polycystin 2	GO:000158
CWF19L1	55280	symbol	H. sapiens	55280	H. sapiens	CWF19L1	CWF19 like	GO:000039
CHUK	1147	symbol	H. sapiens	1147	H. sapiens	CHUK	component	GO:003566
ERLIN1	10613	symbol	H. sapiens	10613	H. sapiens	ERLIN1	ER lipid raft	GO:004554
CPN1	1369	symbol	H. sapiens	1369	H. sapiens	CPN1	carboxypep	GO:001081
DNMBP	23268	symbol	H. sapiens	23268	H. sapiens	DNMBP	dynamin bi	GO:000836
KIF4A	24137	symbol	H. sapiens	24137	H. sapiens	KIF4A	kinesin fam	GO:005125

PDZD11	51248	symbol	H. sapiens	51248	H. sapiens	PDZD11	PDZ domai	GO:004519
ARR3	407	symbol	H. sapiens	407	H. sapiens	ARR3	arrestin 3	GO:000203
P2RY4	5030	symbol	H. sapiens	5030	H. sapiens	P2RY4	pyrimidine	GO:009950
AWAT1	158833	symbol	H. sapiens	158833	H. sapiens	AWAT1	acyl-CoA w	GO:001002
DGAT2L6	347516	symbol	H. sapiens	347516	H. sapiens	DGAT2L6	diacylglyce	GO:003615
IGBP1	3476	symbol	H. sapiens	3476	H. sapiens	IGBP1	immunoglo	GO:004315
OTUD6A	139562	symbol	H. sapiens	139562	H. sapiens	OTUD6A	OTU deubi	GO:199016
AWAT2	158835	symbol	H. sapiens	158835	H. sapiens	AWAT2	acyl-CoA w	GO:003615
EDA	1896	symbol	H. sapiens	1896	H. sapiens	EDA	ectodyspla	GO:006115
CDX4	1046	symbol	H. sapiens	1046	H. sapiens	CDX4	caudal type	GO:006071
FAM155B	27112	symbol	H. sapiens	27112	H. sapiens	FAM155B	family with	GO:009870
PJA1	64219	symbol	H. sapiens	64219	H. sapiens	PJA1	praja ring f	GO:001656
AR	367	symbol	H. sapiens	367	H. sapiens	AR	androgen r	GO:006052
GRIA3	2892	symbol	H. sapiens	2892	H. sapiens	GRIA3	glutamate i	GO:200031
TRPC5	7224	symbol	H. sapiens	7224	H. sapiens	TRPC5	transient re	GO:005077
TRPC5OS	100329135	symbol	H. sapiens	100329135	H. sapiens	TRPC5OS	TRPC5 opposite strand	
DCX	1641	symbol	H. sapiens	1641	H. sapiens	DCX	doublecort	GO:004246
CAPN6	827	symbol	H. sapiens	827	H. sapiens	CAPN6	calpain 6	GO:000157
PAK3	5063	symbol	H. sapiens	5063	H. sapiens	PAK3	p21 (RAC1)	GO:006100
RGAG1	57529	gene_syno	H. sapiens	57529	H. sapiens	RTL9	retrotransposon Gag lil	
ACSL4	2182	symbol	H. sapiens	2182	H. sapiens	ACSL4	acyl-CoA sy	GO:003533
KCNE5	23630	symbol	H. sapiens	23630	H. sapiens	KCNE5	potassium	GO:190376
GPR119	139760	symbol	H. sapiens	139760	H. sapiens	GPR119	G protein-c	GO:003007

rform selective evolution analysis

Kinase Cl	Protein F	Subcellul	Drug (Dru	Canonical	Hallmark	GO:000724	GO:006007	GO:003509
3 cranial suture morphogenesis;GO:0045717 negative regulation of f						0.0	0.0	0.0
.6 retrograde transport		Vesicles(Supported)Plasma membrane(Appro				0.0	0.0	0.0
3 maturatio	Enzymes/E	Nucleoli(Enhanced)		(M66)PID N	(M5928)HA	0.0	0.0	0.0
5 protein targeting;GO:0016197 endosomal transport;GO:0006886 ir						0.0	0.0	0.0
6 G protein-coupled receptor signaling pathwa				(M167)PID AP1 PATHV		0.0	0.0	0.0
7 protein N	Enzymes/ENZYME proteins/Transferases					0.0	0.0	0.0
nd RhoGEF domain containing G7						0.0	0.0	0.0
9 synaptic vesicle to er		Vesicles(Su	Inositol 1,3-Bisphosphate (Zinc ior			1.0	0.0	0.0
None	None	None	None	None	None	nan	nan	nan
.1 adenosin	Enzymes/E	Cytosol(Approved)			(M5945)HA	0.0	0.0	0.0
.3 nucleotid	Enzymes/E	Nucleoli fibrillar cente		(M84)PID A	(M5923)HA	1.0	0.0	0.0
.6 mitochon	Ribosomal	Mitochondria(Supported)Plasma membrane(A				0.0	0.0	0.0
6 growth hormone rec		Endoplasmic reticulum		(M122)PID	(M5947)HA	0.0	0.0	0.0
7 DNA damage respons		Cytosol;Nucleus(Appro		(M197)PID HIV NEF PA		0.0	0.0	0.0
Atypical: Al	Enzymes/K	Centrosome(Approved)				1.0	0.0	0.0
3 cytoplasmic pattern		Cytosol;Nucleoli;Nucleus(Approved)				1.0	0.0	0.0
.5 regulation of Arp2/3		Golgi apparatus(Supported)				0.0	0.0	0.0
None	None	None	None	None	None	nan	nan	nan
1 regulation of dendrit		Focal adhesion sites;Nucleoplasm;			(M5908)HA	0.0	0.0	0.0
1 cyclooxyg	Enzymes/ENZYME pro		Glutathione (Protein homodimeriz			0.0	0.0	0.0
3 histone H	Enzymes/E	Nucleoplasm(Enhanced)				0.0	0.0	0.0
9 positive r	Transcription factors/Basic domains					0.0	0.0	0.0
None	None	None	None	None	None	nan	nan	nan
7 cerebellar granule cell differentia			L-Glutamic Acid (Scaffo		(M5956)HA	0.0	1.0	0.0
acid (anticodon UUC)						0.0	0.0	0.0
8 toxin transport;GO:0022414 reproductive p				(M267)PID	(M5892)HA	0.0	0.0	0.0
3 oligodend	Transcriptio	Nuclear bodies(Approved)				0.0	0.0	0.0
.9 signal tra	RAS pathwa	Nucleoli fibrillar cente		(M5883)NABA SECRET		0.0	0.0	0.0
None	None	None	None	None	None	nan	nan	nan
nticodon CCC)						0.0	0.0	0.0
8 dermatan sulfate bio		Vesicles(Su	Hyaluronic	(M264)PID	(M5944)HA	1.0	1.0	0.0
.3 establishment of inte		Nucleus(Su	S-(Dimethy	(M42)PID DNA PK PATI		0.0	0.0	0.0
6 protein secretion;GO:0002790 peptide secretion;GO:0032940 secre						0.0	0.0	0.0
6 protein import into n		Mitochondria;Nucleoplasm(Approved)				0.0	0.0	0.0
.8 negative	CD markers	Vesicles(Supported)		(M264)PID	(M5897)HA	1.0	0.0	0.0
1 pyrimidin	Transporte	Nucleoplasm(Approved)				0.0	0.0	0.0
5 regulation of transcri		Cytosol(Supported)Actin filaments(Approved)				0.0	0.0	0.0
2 negative regulation c		Cytosol;Nucleoplasm(S		(M200)PID ERA GENON		0.0	0.0	0.0
.7 negative regulation of translational initiation;GO:0006446 regulatio						0.0	0.0	0.0
(anticodon GCA)						0.0	0.0	0.0
3 regulatio	Transporters/Electrochemical Potential-driven transporte					0.0	0.0	0.0
.5 negative regulation of elastin biosynthetic p				(M201)PID	(M5932)HA	0.0	0.0	0.0
.7 protein folding;GO:0		Cytosol;Nucleoli;Plasma membrane(Approvec				0.0	0.0	0.0
.2 neutrophil degranulation;GO:0002283 neutrophil activation involve						0.0	0.0	0.0
		Vesicles(Approved)				0.0	0.0	0.0

3 dendritic Transcription factors/Yet undefined DNA-binc (M5901)HA	0.0	0.0	0.0
.8 ERBB2 signaling path Nucleoli;Nucleus(Supp (M201)PID ERBB NETW	0.0	0.0	0.0
.2 regulation of ARF protein signal transduction;GO:0032011 ARF prot	0.0	0.0	0.0
.3 positive regulation of Cytosol;Nucleoplasm(Enhanced)	1.0	0.0	0.0
6 TRIF-depe Enzymes/E Cytosol;Pla (R,R)-2,3-Bı (M214)PID ERBB1 INTE	1.0	0.0	0.0
g 2 Nuclear bodies(Supported)	0.0	0.0	0.0
5 vacuolar acidification Cytosol;Nucleoli(Supported)	0.0	0.0	0.0
4 negative regulation c Nucleoplasm(Approved) (M5908)HA	0.0	0.0	0.0
.1 very long Enzymes/E Peroxisome NADH (Receptor bindir (M5949)HA	0.0	0.0	0.0
3 positive regulation of transcription, DNA-templated;GO:1903508 p	0.0	0.0	0.0
None None None None None None	nan	nan	nan
3 positive regulation of Cytosol;Midbody;Nucleus(Approved)	0.0	0.0	0.0
6 embryonic process involved in female pregnancy;GO:0090090 nega	0.0	1.0	0.0
7 beta-catenin-TCF cor Nuclear bodies(Suppor (M223)PID BETA CATEI	0.0	1.0	0.0
.0 positive regulation of Nucleoplasm(Supporte (M70)PID PS1 PATHWA	1.0	1.0	0.0
4 regulation of melanocyte differentiation;GO (M138)PID THROMBIN	0.0	0.0	0.0
7 phospholipase C-activating G protein-couple (M138)PID (M5902)HA	0.0	0.0	0.0
nticodon CAC)	0.0	0.0	0.0
2 activation G-protein c Mitochondria(Uncerta (M64)PID S1P S1P4 PA	0.0	0.0	0.0
5 regulation of protein Lipid droplets;Nucleoplasm(Uncertain)	0.0	0.0	0.0
6 mRNA splice site selection;GO:0000381 regulation of alternative m	0.0	0.0	0.0
5 odontoge Transcriptio Nucleus(Enhanced)Nuc (M285)PID HNF3A PAT	0.0	0.0	0.0
0 biological_process Cytosol;Nucleoplasm(Approved)	0.0	0.0	0.0
.2 peptidyl-l Enzymes/E Cytosol;Nucleus(Approved)	0.0	0.0	0.0
.5 signal transduction ir Nuclear membrane;Nu (M242)PID AURORA A	1.0	0.0	0.0
None None None None None None	nan	nan	nan
.2 negative regulation c Mitochondria;Nucleoplasm(Approved)	0.0	0.0	0.0
4 negative Transcriptio Nucleoplasm(Enhanced)	0.0	0.0	0.0
aining family member 3 Golgi apparatus;Nuclear bodies;Nucleoplasm(	0.0	0.0	0.0
1 negative G-protein c Plasma me Ridogrel (T (M12645)ST GA13 PAT	1.0	0.0	0.0
5 negative regulation c Nuclear speckles;Nucleoplasm(Supported)Cyt	1.0	0.0	0.0
3 neutroph Enzymes/E Cytosol;Nucleoplasm(A (M72)PID NECTIN PATI	0.0	0.0	0.0
3 Cell Junctions(Enhanced)Nucleopla (M5906)HA	0.0	0.0	0.0
1 in utero embryonic d Vesicles(Approved)	0.0	0.0	0.0
.6 mitochondrial transla Mitochondria(Approved)	0.0	0.0	0.0
1 visual per Transcription factors/Helix-turn-helix domains	0.0	0.0	0.0
Tyr protein Enzymes/{ENZYME proteins/Trans (M231)PID KIT PATHW	0.0	0.0	0.0
g protein 2 Nucleoplasm(Uncertain)	0.0	0.0	0.0
.2 negative regulation of glutamate metabolic process;GO:2000211 re	0.0	0.0	0.0
5 NAD bios Enzymes/E Nucleoplasm;Vesicles(Enhanced)	0.0	0.0	0.0
CAMK Ser/ Enzymes/{E Nucleus(Ap 2-(1,1-DIMETHYLETHYL)9-FLUORO	0.0	1.0	0.0
.6 cellular response to k Cytosol;Pla Adenosine- (M121)PID (M5922)HA	0.0	0.0	0.0
4 positive r Enzymes/E Nucleoplasm(Approve (M228)PID SMAD2 3PA	1.0	1.0	0.0
6 negative Transcriptio Nucleoplasm(Approved)	1.0	0.0	0.0
STE Ser/Th Enzymes/{E Cytosol(Su Bosutinib (: (M18895)SA TRKA REC	1.0	0.0	0.0
.0 positive r Transcription factors/Basic domains	1.0	0.0	0.0
9 positive regulation of Nucleoplasm(Supporte (M101)PID (M5921)HA	0.0	0.0	0.0

24		Cytosol;Microtubules(Uncertain)				0.0	0.0	0.0
1 positive regulation of alpha-beta T cell prolif				(M36)PID II	(M5897)HA	0.0	0.0	0.0
8 negative regulation c		Nucleoplasm(Approved)				0.0	0.0	0.0
n containing transformi		Cytosol;Microtubule organizing center(Uncert				0.0	0.0	0.0
04 positive regulation of activated T cell proliferation;GO:0031295 T c						0.0	0.0	0.0
6 regulation of mitotic spindle organization;GO:0090224 regulation c						0.0	0.0	0.0
1 positive regulation of tyrosine phosphorylation of STAT protein;GO						0.0	0.0	0.0
08 proteolys	Enzymes/P	Nucleoli;Nucleus(Approved)				0.0	0.0	0.0
01 synaptic vesicle unco		Cytosol(Enhanced)		(M281)PID FAK PATHW		0.0	1.0	0.0
5 transmen	Transporte	Cytosol;Nucleoplasm(Approved)				0.0	0.0	0.0
		Nucleoli;Nucleus;Vesic		(M5880)NA	(M5953)HA	0.0	0.0	0.0
5 positive r	Enzymes/E	Cytosol(Supported)Intermediate fi			(M5911)HA	1.0	0.0	0.0
4 keratinocyte develop		Actin filaments;Cytosol(Supported			(M5893)HA	0.0	0.0	0.0
00 negative	Transcripti	Nucleoplasm(Enhanced)Nuclear bodies(Suppo				0.0	0.0	0.0
78 mRNA po	Enzymes/E	Nucleoplasm(Enhanced)				0.0	0.0	0.0
38 negative	RAS pathway related proteins;Tra			(M26)PID N	(M5890)HA	1.0	0.0	0.0
9 tRNA pse	Enzymes/E	Mitochondria;Nuclear bodies;Nucleoplasm(Ap				0.0	0.0	0.0
50 protein ir	Transporte	Vesicles(Supported)			(M5949)HA	0.0	0.0	0.0
		Nucleoli;Nucleus(Approved)				0.0	0.0	0.0
5 spliceosomal comple		Nucleoplasm(Supported)				0.0	0.0	0.0
6 mitochon	Ribosomal	Mitochondria(Supported)				0.0	0.0	0.0
57 post-translational pro		Vesicles(Supported)Plasma membrane(Appro				0.0	0.0	0.0
6 mitochon	Ribosomal	Mitochondria(Enhanced)			(M5905)HA	0.0	0.0	0.0
0 negative	Enzymes/E	Nucleoli fibrillar center;Nucleus(Supported)				1.0	0.0	0.0
74 gangliosi	Enzymes/E	Vesicles(Ap	99mTc-14 F7 Mab (Sial		(M5913)HA	0.0	0.0	0.0
03 positive r	Transcripti	Nucleoplasm(Supported)			(M5934)HA	0.0	0.0	0.0
58 cellular d	Transporters/Transpor		3[N-Morpholino]Propane Sulfonic			0.0	0.0	0.0
55 respiratory gaseous exchange;GO:0006665 s				(M5880)NABA ECM AF		0.0	0.0	0.0
5 spliceoso	Enzymes/P	Nucleoplasm(Supported)				0.0	0.0	0.0
None	None	None	None	None	None	nan	nan	nan
4 phosphatidylinositol		Nucleoplasm;Vesicles(Approved)				0.0	0.0	0.0
5 protein a	Enzymes/E	Cytosol;Nucleoplasm(Approved)				0.0	0.0	0.0
01 Golgi to plasma membrane protein transport;GO:0061					(M5913)HA	0.0	0.0	0.0
05 positive r	Transporters/Transporter channels and pores				(M5939)HA	1.0	0.0	0.0
57 peptidyl-g	Enzymes/ENZYME prot		Coagulation factor VIIa Recombina			0.0	0.0	0.0
6 S-adenos	Enzymes/E	Cytosol;Nu	S-Adenosyl	(M195)PID CMYB PATH		0.0	0.0	0.0
59 transmembrane receptor protein tyrosine kinase signaling pathway						0.0	0.0	0.0
6 barbed-end actin fila		Nucleus(Enhanced)			(M5947)HA	0.0	0.0	0.0
7 positive regulation of		Mitochondria;Nucleoplasm(Appro			(M5905)HA	0.0	0.0	0.0
72 retinol m	Enzymes/E	Nucleoli(Ap	Vitamin A (Oxidoreduc		(M5949)HA	0.0	0.0	0.0
57 post-translational pro		Golgi apparatus;Nucleoplasm(Enhanced)				0.0	0.0	0.0
57 beta-cate	Transcripti	Nucleoplasm(Supporte		(M236)PID	(M5956)HA	0.0	1.0	0.0
34 negative regulation c		Cytosol(Supported)				0.0	0.0	0.0
0 negative	Enzymes/E	Vesicles(Supported)				1.0	0.0	0.0
2 negative	Transcripti	Nucleoplasm(Supported)Mitochondria(Appro				0.0	0.0	0.0
2 negative	Transcription factors/Zinc-coordinating DNA-binding dom					0.0	0.0	0.0
7 regulation of cilium a		Cytosol(Enhanced)			(M5893)HA	0.0	0.0	0.0

.7 negative regulation c		Cytosol(Enhanced)				0.0	0.0	0.0
6 spermatid development;GO:0007638 mechanosensory					(M5951)HA	0.0	0.0	0.0
.1 ingression involved in gastrulation with mouth forming second;GO:						0.0	0.0	1.0
3 regulation of Rab prc		Vesicles(Supported)Cytosol;Nucleoplasm(App				0.0	0.0	0.0
9 maintena	Transcripti	Nucleoplasm(Approved)			(M5941)HA	0.0	0.0	1.0
NEK Ser/Th	Enzymes/{I	Cytosol;Microtubule organizing center;Nucleo				1.0	0.0	0.0
.3 stimulato	Enzymes/{I	Cytosol;Nu	(3AR,6R,6AS)-6-((S)-((S)-CYCLOHEX			1.0	1.0	0.0
1 negative regulation of sperm cap			12-Bromododecanoic Acid (Small			0.0	0.0	0.0
9 lipid glycosylation;GO:0030258 l			Uridine diphosphate glucose (Tran			0.0	0.0	0.0
			2-Methoxy-3-Isopropylpyrazine (T			0.0	0.0	0.0
4 oocyte di	Transcripti	Nuclear speckles(Approved)				0.0	0.0	0.0
5 potassiur	Transporters/Transporter channels and pores;Voltage-ga					0.0	0.0	0.0
.6 negative regulation c		Cytosol;Mitotic spindle(Supported)				0.0	0.0	0.0
7 protein ubiquitinatio		Cytosol(Supported)Golgi apparatu			(M5945)HA	1.0	0.0	0.0
7 negative regulation c		Mitochondria;Nucleoli(Approved)				0.0	0.0	0.0
None	None	None	None	None	None	nan	nan	nan
3 microtubule anchoring;GO:0000226 microtubule cytoskeleton orga						0.0	0.0	0.0
8 negative regulation c		Golgi apparatus(Supported)Nucleoplasm(App				0.0	0.0	0.0
0 protein import into n		Mitochondria(Enhanced)Nucleoplasm(Approv				0.0	0.0	0.0
.3 stimulatory C-type lectin receptor signaling pathway;GO:0002220 i						1.0	0.0	0.0
6 snRNA tra	Transcripti	Nuclear membrane;Nucleoplasm(/			(M5898)HA	0.0	0.0	0.0
6 positive regulation of protein localization to cilium;GO:1903564 reg						0.0	0.0	0.0
.7 protein p	Enzymes/{I	Mitochondria(Enhanced)			(M5936)HA	0.0	0.0	0.0
5 negative	Enzymes/ENZYME proteins/Hydrolases					0.0	0.0	0.0
0 Golgi to plasma mem		Endoplasmic reticulum;Golgi apparatus(Suppo				1.0	0.0	0.0
0 Notch signaling pathv		Nucleoplasm(Approve	(M70)PID F		(M5903)HA	1.0	1.0	0.0
0 vasculogenesis;GO:0045746 negative regula			(M5883)NABA SECRET			0.0	0.0	0.0
9 positive regulation of blood vessel endothelial cell proliferation inv						1.0	0.0	0.0
.4 CDP-diac	Enzymes/E	Centrosome(Approved)				0.0	0.0	0.0
se domain 1B		Nuclear bodies;Nucleus;Vesicles(Approved)				0.0	0.0	0.0
9 lipid glycosylation;GO:0006486 p			Uridine-Diphosphate-N-Acetylgala			0.0	0.0	0.0
4 ribosomal small subu		Nucleoli(Enhanced)Nucleus(Approved)				0.0	0.0	0.0
7 regulation of transcri		Nucleoplasm(Approved)				0.0	0.0	0.0
.4 SRP-depe	Ribosomal	Nucleoli;Vesicles(Approved)				0.0	0.0	0.0
0 biological_process		Nucleoli;Nucleus(Enhanced)				0.0	0.0	0.0
8 early end	Transporters					0.0	0.0	0.0
Other	Enzymes/Kinases					0.0	0.0	0.0
8 DNA catabolic proces		Nucleoli;Nucleus(Supported)				0.0	0.0	0.0
4 response	Enzymes/ENZYME proteins/Hydro		(M3468)NABA ECM RE			1.0	0.0	0.0
2 vesicle-m	Transporte	Nucleoplasm(Approved)				0.0	0.0	0.0
0 positive regulation o		Mitochondria;Nucleoplasm(Approved)				1.0	0.0	0.0
.7 protein p	Enzymes/{I	Mitochondria(Enhanced)				0.0	0.0	0.0
5 signal transduction;GO:0023052 signaling;G			(M5880)NABA ECM AF			0.0	0.0	0.0
2 lamellipodium assem		Cytosol;Nucleoplasm(S	(M83)PID CDC42 REG F			0.0	0.0	0.0
0 negative regulation of canonical Wnt signaling pathway;GO:00073						0.0	1.0	0.0
6 phosphatidylinositol dephosphorylation;GO:0006661 phosphatidyl						0.0	0.0	0.0
9 tRNA exp	Transporte	Cytosol;Mitochondria;Plasma mer			(M5941)HA	0.0	0.0	0.0

None	None	None	None	None	None	nan	nan	nan
2 positive r	Enzymes/E	Plasma membrane(Supported)Nucleoplasm(U				0.0	0.0	0.0
3 lipopolysaccharide-m		Endoplasmic reticulum	(M66)PID MYC ACTIV F			1.0	0.0	0.0
6 negative	Transporte	Golgi apparatus(Approved)				0.0	0.0	0.0
9 growth plate cartilage chondrocyte morpho			(M3008)NA	(M5930)HA		0.0	0.0	0.0
1 positive r	Ribosomal	Cytosol;Endoplasmic reticulum(Approved)				1.0	0.0	0.0
		Cytosol;Golgi apparatus;Nucleoplasm(Approv				0.0	0.0	0.0
8 L-threonine catabolic process to			Benzoic Acid (Xenon atom binding			0.0	0.0	0.0
2 tRNA 5'-le	Enzymes/E	Nucleoli(Supported)				0.0	0.0	0.0
0 magnesium ion transmembrane transport;GO:0015693 magnesium						0.0	0.0	0.0
9 regulatio	Transporters/Transporter channels and pores;Voltage-ga					0.0	0.0	0.0
STE Ser/Th	Enzymes/{E	Intermediate filaments;Rods & Rings(Approve				1.0	1.0	0.0
CAMK Ser/	Enzymes/{ENZYME proteins/Transferases,Kinases/CAMK					0.0	0.0	0.0
3 positive regulation o		Cytosol;Golgi apparatus(Supporte		(M5910)HA		0.0	0.0	0.0
3 neutrophil chemotax		Cell Junctions;Nucleop	(M118)PID	(M5915)HA		0.0	0.0	0.0
4 negative	Enzymes/E	Nucleoplasm;Vesicles(	(M228)PID SMAD2 3PA			0.0	0.0	0.0
9 negative regulation of inward rectifier potassium channel activity;G						1.0	0.0	0.0
3 actin filament capping;GO:0030835 negative regulation of actin fila						0.0	0.0	0.0
9 release o	Enzymes/E	Microtubul	D-Myo-Inositol-1,4,5-Triphosphate			0.0	0.0	0.0
5 negative regulation c		Cytosol(Supported)				0.0	0.0	0.0
4 phenylac	Enzymes/E	Peroxisome	Trimetazidine (Palmito	(M5949)HA		0.0	0.0	0.0
2 toll-like receptor 9 si		Cytosol(Supported)Mit	(M264)PID	(M5897)HA		1.0	0.0	0.0
STE Ser/Th	Enzymes/{E	Cytosol(Enhanced)		(M5938)HA		1.0	0.0	0.0
3 stimulatory C-type le		Cytosol;Nucleoplasm;F	(M5493)W	(M5945)HA		1.0	1.0	0.0
0 regulatio	Transcripti	Nuclear bodies;Nucleoplasm(Approved)				0.0	0.0	0.0
4 positive r	Transcription factors/Helix-turn-helix domains			(M5956)HA		0.0	0.0	0.0
8 extracellular matrix organization;GO:00015			(M3468)NABA ECM RE			0.0	0.0	0.0
0 one-carbon metaboli		Mitochondria(Enhance	(M254)PID MYC REPRE			0.0	0.0	0.0
8 auditory receptor cell stereocilium organization;GO:0002093 audit						0.0	0.0	0.0
2 primary ureteric bud		Cytosol;Plasma membrane(Approved)				0.0	1.0	0.0
None	None	None	None	None	None	nan	nan	nan
6 mitochon	Ribosomal	Mitochondria(Supported)Nucleoplasm(Uncer				0.0	0.0	0.0
3 negative regulation c		Lipid droplets;Vesicles	(M5880)NABA ECM AF			0.0	0.0	0.0
4 positive regulation o		Nucleus(Supported)Vesicles(Approved)				0.0	0.0	0.0
4 positive r	Transcripti	Nucleoli(Supported)Golgi apparatus(Uncertai				0.0	0.0	0.0
8 negative	Enzymes/E	Cytosol;Nu	D-tartaric a	(M180)PID HIF1A PATH		0.0	0.0	0.0
0 mitochondrial electr		Mitochond	NADH (Nadh dehydrog	(M5936)HA		0.0	0.0	0.0
0 cargo loading into CC		Cytosol;Nucleoli;Nucleus;Vesicles(Approved)				0.0	0.0	0.0
3 determination of dorsal identity;GO:190488			(M5883)NABA SECRET			0.0	1.0	0.0
9 fatty-acyl	Enzymes/E	Endoplasmic reticulum(Enhanced)		(M5892)HA		0.0	0.0	0.0
1 detection	Transporters/Transporter channels and pores;Voltage-ga					0.0	0.0	0.0
8 mRNA splicing, via sp		Golgi apparatus;Nucleoplasm(Approved)				0.0	0.0	0.0
Other	Enzymes/{E	Cytosol;Nu	Aminosalic	(M37)PID N	(M5905)HA	1.0	0.0	0.0
1 negative regulation c		Endoplasmic reticulum(Supported)				0.0	0.0	0.0
5 bradykini	Enzymes/{E	Nucleoli;Nucleus(Approved)		(M5946)HA		1.0	0.0	0.0
0 regulation of cell sha		Cytosol;Golgi apparatu	(M83)PID C	(M5953)HA		0.0	0.0	0.0
6 mitotic spindle midzo		Cytokinetic bridge;Nucleoplasm(E		(M5893)HA		0.0	0.0	0.0

9 maintenance of epithelial cell apical/basal polarity;GO:0006768 bic						0.0	0.0	1.0
1 G protein-coupled receptor internalization;(				(M117)PID CONE PATH		0.0	0.0	0.0
9 regulatio	G-protein coupled receptors/{Adenosine and				(M5956)HA	0.0	0.0	0.0
5 wax biosy	Enzymes/ENZYME proteins/Transferases					0.0	0.0	0.0
5 acylglycerol acyl-chain remodeling;GO:0006639 acylglycerol metab						0.0	0.0	0.0
4 negative regulation c		Microtubules(Approved)				1.0	0.0	0.0
7 protein K	Enzymes/ENZYME proteins/Hydrolases					0.0	0.0	0.0
5 acylglycer	Enzymes/ENZYME proteins/Transferases					0.0	0.0	0.0
3 trachea gland develo		Vesicles(Supported)Lip		(M5883)NABA SECRET		1.0	1.0	0.0
1 labyrinthi	Transcription factors/Helix-turn-h			(M223)PID BETA CATE		0.0	1.0	0.0
3 calcium ion import across plasma membrane;GO:1902656 calcium						0.0	0.0	0.0
7 protein u	Enzymes/E	Nucleoli;Nucleus(Approved)				1.0	0.0	0.0
0 activatio	Nuclear re	Mitochond	Levonorges	(M213)PID	(M5948)HA	1.0	1.0	0.0
0 regulatio	Transporters/Transpor		L-Glutamic Acid (Extracellular-glut			0.0	0.0	0.0
4 negative	Transporters/Transporter channels and pores;Voltage-ga					0.0	0.0	0.0
						0.0	0.0	0.0
1 photoreceptor cell d		Cytosol(Approved)		(M163)PID	(M5957)HA	0.0	0.0	0.0
8 microtub	Enzymes/P	Cytosol(Uncertain)				0.0	0.0	0.0
STE Ser/Th	Enzymes/{	Cytosol(Approved)		(M76)PID F	(M5957)HA	1.0	0.0	0.0
ke 9						0.0	0.0	0.0
8 long-chai	Enzymes/E	Golgi appa	Icosapent (Very long-c		(M5949)HA	0.0	0.0	0.0
5 negative regulation c		Cytosol(Uncertain)				0.0	0.0	0.0
3 insulin se	G-protein coupled rec		APD668 (Phosphatidylcholine binc			0.0	0.0	0.0

R-HSA-400	GO:003025	GO:004362	GO:000689	R-HSA-533	GO:004858	hsa05215	GO:004566	GO:000734
0.0	1.0	0.0	0.0	0.0	0.0	0.0	0.0	0.0
0.0	0.0	0.0	1.0	0.0	0.0	0.0	0.0	0.0
0.0	0.0	0.0	0.0	0.0	0.0	0.0	0.0	0.0
0.0	0.0	0.0	0.0	0.0	0.0	0.0	0.0	0.0
0.0	0.0	0.0	0.0	0.0	0.0	0.0	0.0	0.0
0.0	0.0	0.0	0.0	0.0	0.0	0.0	0.0	0.0
0.0	0.0	0.0	0.0	0.0	0.0	0.0	0.0	0.0
0.0	0.0	0.0	0.0	0.0	0.0	0.0	0.0	0.0
nan	nan	nan	nan	nan	nan	nan	nan	nan
0.0	0.0	0.0	0.0	0.0	0.0	0.0	0.0	0.0
0.0	0.0	0.0	0.0	0.0	0.0	0.0	0.0	0.0
0.0	0.0	1.0	0.0	0.0	0.0	0.0	0.0	0.0
0.0	1.0	0.0	0.0	0.0	1.0	0.0	0.0	0.0
0.0	0.0	0.0	0.0	0.0	0.0	0.0	0.0	1.0
0.0	0.0	0.0	0.0	0.0	0.0	0.0	0.0	0.0
0.0	0.0	0.0	0.0	0.0	0.0	0.0	0.0	0.0
0.0	0.0	0.0	1.0	0.0	1.0	0.0	0.0	0.0
nan	nan	nan	nan	nan	nan	nan	nan	nan
0.0	0.0	0.0	0.0	0.0	1.0	0.0	0.0	0.0
0.0	1.0	0.0	0.0	0.0	0.0	0.0	0.0	0.0
0.0	0.0	0.0	0.0	0.0	0.0	0.0	0.0	0.0
0.0	0.0	0.0	0.0	0.0	1.0	0.0	0.0	0.0
nan	nan	nan	nan	nan	nan	nan	nan	nan
0.0	0.0	0.0	0.0	0.0	0.0	0.0	0.0	0.0
0.0	0.0	0.0	0.0	0.0	0.0	0.0	0.0	0.0
0.0	0.0	0.0	0.0	1.0	0.0	0.0	0.0	0.0
0.0	0.0	0.0	0.0	0.0	0.0	0.0	0.0	0.0
0.0	0.0	0.0	0.0	0.0	0.0	0.0	0.0	0.0
nan	nan	nan	nan	nan	nan	nan	nan	nan
0.0	0.0	0.0	0.0	0.0	0.0	0.0	0.0	0.0
0.0	0.0	0.0	0.0	0.0	0.0	0.0	0.0	0.0
0.0	0.0	0.0	0.0	0.0	0.0	0.0	0.0	0.0
0.0	0.0	0.0	0.0	0.0	0.0	0.0	0.0	0.0
0.0	0.0	0.0	0.0	0.0	0.0	0.0	0.0	0.0
0.0	0.0	0.0	0.0	0.0	0.0	0.0	0.0	0.0
0.0	0.0	0.0	0.0	0.0	0.0	0.0	0.0	0.0
0.0	0.0	0.0	0.0	0.0	0.0	0.0	0.0	0.0
0.0	0.0	0.0	0.0	0.0	0.0	0.0	1.0	1.0
0.0	0.0	0.0	0.0	0.0	0.0	0.0	0.0	0.0
0.0	0.0	0.0	0.0	0.0	0.0	0.0	0.0	0.0
0.0	0.0	0.0	0.0	0.0	0.0	0.0	0.0	0.0
1.0	0.0	0.0	0.0	1.0	0.0	0.0	0.0	0.0
0.0	0.0	0.0	0.0	0.0	0.0	0.0	0.0	0.0
0.0	0.0	0.0	0.0	0.0	0.0	0.0	0.0	0.0
0.0	0.0	0.0	0.0	0.0	0.0	0.0	0.0	0.0

0.0	0.0	0.0	0.0	0.0	0.0	0.0	0.0	0.0
0.0	0.0	0.0	0.0	0.0	0.0	0.0	0.0	0.0
0.0	0.0	0.0	0.0	0.0	0.0	0.0	0.0	0.0
0.0	0.0	0.0	0.0	0.0	0.0	0.0	0.0	0.0
0.0	0.0	0.0	0.0	0.0	0.0	0.0	0.0	0.0
0.0	0.0	0.0	0.0	0.0	0.0	0.0	0.0	0.0
0.0	0.0	0.0	0.0	0.0	0.0	0.0	0.0	0.0
0.0	1.0	0.0	0.0	0.0	0.0	0.0	0.0	0.0
0.0	1.0	0.0	0.0	0.0	0.0	0.0	0.0	0.0
0.0	0.0	0.0	0.0	0.0	0.0	0.0	0.0	0.0
nan	nan	nan	nan	nan	nan	nan	nan	nan
0.0	0.0	0.0	0.0	0.0	0.0	0.0	0.0	0.0
0.0	0.0	0.0	0.0	0.0	0.0	0.0	0.0	0.0
0.0	0.0	0.0	0.0	0.0	0.0	0.0	0.0	0.0
0.0	0.0	0.0	0.0	0.0	0.0	0.0	0.0	0.0
1.0	0.0	0.0	0.0	0.0	0.0	0.0	0.0	0.0
1.0	0.0	0.0	0.0	0.0	0.0	0.0	0.0	0.0
0.0	0.0	0.0	0.0	0.0	0.0	0.0	0.0	0.0
1.0	0.0	0.0	0.0	0.0	0.0	0.0	0.0	0.0
0.0	0.0	0.0	0.0	0.0	0.0	0.0	0.0	0.0
0.0	0.0	0.0	0.0	0.0	0.0	0.0	0.0	0.0
0.0	0.0	0.0	0.0	0.0	0.0	0.0	0.0	0.0
0.0	0.0	0.0	0.0	0.0	0.0	0.0	0.0	0.0
0.0	0.0	0.0	0.0	0.0	0.0	0.0	0.0	0.0
0.0	0.0	0.0	0.0	0.0	0.0	0.0	0.0	1.0
nan	nan	nan	nan	nan	nan	nan	nan	nan
0.0	0.0	0.0	0.0	0.0	0.0	0.0	0.0	0.0
0.0	0.0	0.0	0.0	0.0	0.0	0.0	0.0	0.0
0.0	0.0	0.0	0.0	0.0	0.0	0.0	0.0	0.0
1.0	0.0	0.0	0.0	0.0	0.0	0.0	0.0	0.0
0.0	0.0	0.0	0.0	0.0	0.0	0.0	0.0	0.0
0.0	1.0	0.0	0.0	0.0	0.0	0.0	0.0	0.0
0.0	0.0	0.0	0.0	0.0	0.0	0.0	0.0	0.0
0.0	0.0	0.0	0.0	0.0	0.0	0.0	0.0	0.0
0.0	0.0	1.0	0.0	0.0	0.0	0.0	0.0	0.0
0.0	0.0	0.0	0.0	0.0	0.0	0.0	0.0	0.0
0.0	0.0	0.0	0.0	0.0	0.0	0.0	0.0	0.0
0.0	0.0	0.0	0.0	0.0	0.0	0.0	0.0	0.0
0.0	0.0	0.0	0.0	0.0	0.0	0.0	0.0	0.0
0.0	0.0	0.0	0.0	0.0	0.0	0.0	1.0	0.0
0.0	0.0	0.0	0.0	1.0	0.0	0.0	0.0	1.0
0.0	0.0	1.0	0.0	1.0	0.0	0.0	0.0	0.0
0.0	1.0	0.0	0.0	0.0	0.0	0.0	0.0	0.0
0.0	0.0	0.0	0.0	0.0	0.0	0.0	0.0	0.0
0.0	0.0	0.0	0.0	1.0	0.0	1.0	0.0	1.0
0.0	0.0	0.0	0.0	0.0	0.0	1.0	0.0	0.0
0.0	0.0	0.0	0.0	0.0	1.0	0.0	0.0	0.0

0.0	0.0	0.0	0.0	0.0	0.0	0.0	0.0	0.0
0.0	0.0	0.0	0.0	0.0	0.0	0.0	0.0	0.0
0.0	0.0	0.0	0.0	0.0	0.0	0.0	0.0	0.0
0.0	0.0	0.0	0.0	0.0	0.0	0.0	0.0	0.0
0.0	0.0	0.0	0.0	0.0	0.0	0.0	0.0	0.0
0.0	0.0	0.0	0.0	0.0	0.0	0.0	0.0	1.0
0.0	0.0	0.0	0.0	0.0	0.0	0.0	0.0	0.0
0.0	0.0	0.0	0.0	0.0	0.0	0.0	0.0	0.0
0.0	0.0	1.0	0.0	0.0	0.0	0.0	0.0	0.0
0.0	0.0	0.0	0.0	0.0	0.0	0.0	0.0	0.0
0.0	0.0	0.0	0.0	0.0	0.0	0.0	0.0	0.0
0.0	0.0	0.0	0.0	0.0	0.0	0.0	0.0	0.0
0.0	0.0	0.0	0.0	0.0	1.0	0.0	0.0	0.0
0.0	0.0	0.0	0.0	0.0	1.0	0.0	0.0	0.0
0.0	0.0	0.0	0.0	0.0	0.0	0.0	0.0	0.0
0.0	0.0	0.0	0.0	0.0	0.0	0.0	0.0	0.0
0.0	0.0	0.0	0.0	0.0	0.0	0.0	0.0	0.0
0.0	1.0	0.0	0.0	0.0	0.0	0.0	0.0	0.0
0.0	0.0	0.0	0.0	0.0	0.0	0.0	0.0	0.0
0.0	0.0	0.0	0.0	0.0	0.0	0.0	0.0	0.0
0.0	0.0	1.0	0.0	0.0	0.0	0.0	0.0	0.0
0.0	0.0	0.0	0.0	0.0	0.0	0.0	0.0	0.0
0.0	0.0	1.0	0.0	0.0	0.0	0.0	0.0	0.0
0.0	0.0	0.0	0.0	0.0	0.0	0.0	0.0	0.0
0.0	0.0	0.0	0.0	0.0	0.0	0.0	0.0	0.0
0.0	0.0	0.0	0.0	0.0	0.0	0.0	0.0	0.0
0.0	0.0	0.0	0.0	0.0	0.0	0.0	0.0	0.0
0.0	0.0	0.0	0.0	0.0	0.0	0.0	0.0	0.0
0.0	0.0	0.0	0.0	0.0	0.0	0.0	0.0	0.0
nan	nan	nan	nan	nan	nan	nan	nan	nan
0.0	1.0	0.0	0.0	0.0	0.0	0.0	0.0	0.0
0.0	0.0	0.0	0.0	0.0	0.0	0.0	0.0	0.0
0.0	0.0	0.0	1.0	0.0	0.0	0.0	0.0	0.0
0.0	0.0	1.0	1.0	0.0	0.0	0.0	0.0	0.0
0.0	0.0	0.0	0.0	0.0	0.0	0.0	0.0	0.0
0.0	0.0	0.0	0.0	0.0	0.0	0.0	0.0	0.0
0.0	0.0	0.0	0.0	0.0	0.0	0.0	0.0	0.0
0.0	0.0	1.0	0.0	0.0	0.0	0.0	0.0	0.0
0.0	0.0	0.0	0.0	0.0	0.0	0.0	0.0	0.0
0.0	0.0	0.0	0.0	0.0	0.0	0.0	0.0	0.0
0.0	0.0	0.0	0.0	0.0	0.0	0.0	0.0	0.0
0.0	0.0	0.0	0.0	0.0	0.0	1.0	0.0	0.0
0.0	0.0	0.0	0.0	0.0	0.0	0.0	0.0	0.0
0.0	0.0	0.0	0.0	0.0	1.0	0.0	0.0	0.0
0.0	0.0	0.0	0.0	0.0	0.0	0.0	0.0	0.0
0.0	0.0	0.0	0.0	0.0	0.0	0.0	0.0	0.0
0.0	0.0	0.0	0.0	0.0	0.0	0.0	0.0	0.0

0.0	0.0	0.0	0.0	0.0	1.0	0.0	0.0	0.0
0.0	0.0	0.0	0.0	0.0	0.0	0.0	0.0	0.0
0.0	0.0	0.0	0.0	0.0	0.0	0.0	0.0	0.0
0.0	0.0	0.0	0.0	0.0	0.0	0.0	0.0	0.0
0.0	0.0	0.0	0.0	0.0	1.0	0.0	0.0	0.0
0.0	0.0	0.0	0.0	0.0	0.0	0.0	0.0	1.0
0.0	0.0	0.0	0.0	0.0	0.0	0.0	0.0	1.0
0.0	0.0	0.0	0.0	0.0	0.0	0.0	0.0	0.0
0.0	1.0	0.0	0.0	0.0	0.0	0.0	0.0	0.0
0.0	0.0	0.0	0.0	0.0	0.0	0.0	0.0	0.0
0.0	0.0	0.0	0.0	0.0	0.0	0.0	0.0	0.0
0.0	0.0	0.0	0.0	0.0	0.0	0.0	0.0	0.0
0.0	0.0	1.0	0.0	0.0	0.0	0.0	0.0	0.0
0.0	0.0	0.0	0.0	0.0	0.0	0.0	0.0	0.0
0.0	0.0	0.0	0.0	0.0	0.0	0.0	0.0	1.0
nan	nan	nan	nan	nan	nan	nan	nan	nan
0.0	0.0	0.0	0.0	0.0	0.0	0.0	0.0	0.0
0.0	0.0	0.0	0.0	0.0	0.0	0.0	0.0	0.0
0.0	0.0	0.0	0.0	0.0	0.0	0.0	0.0	0.0
0.0	0.0	0.0	0.0	0.0	0.0	0.0	0.0	0.0
0.0	0.0	0.0	0.0	0.0	0.0	0.0	0.0	0.0
0.0	0.0	0.0	1.0	0.0	0.0	0.0	0.0	0.0
0.0	0.0	0.0	0.0	0.0	0.0	0.0	0.0	0.0
0.0	1.0	0.0	0.0	0.0	0.0	0.0	0.0	0.0
0.0	0.0	0.0	1.0	0.0	0.0	0.0	0.0	0.0
0.0	0.0	0.0	0.0	0.0	1.0	0.0	1.0	0.0
0.0	0.0	0.0	0.0	0.0	0.0	0.0	0.0	0.0
0.0	0.0	0.0	0.0	0.0	0.0	0.0	0.0	0.0
0.0	1.0	0.0	0.0	0.0	0.0	0.0	0.0	0.0
0.0	0.0	0.0	0.0	0.0	0.0	0.0	0.0	0.0
0.0	1.0	0.0	0.0	0.0	0.0	0.0	0.0	0.0
0.0	0.0	0.0	0.0	0.0	0.0	0.0	0.0	0.0
0.0	1.0	0.0	0.0	0.0	0.0	0.0	0.0	0.0
0.0	0.0	1.0	0.0	0.0	0.0	0.0	0.0	0.0
0.0	0.0	0.0	0.0	0.0	0.0	0.0	0.0	0.0
0.0	0.0	0.0	0.0	0.0	0.0	0.0	0.0	0.0
0.0	0.0	0.0	0.0	0.0	0.0	0.0	0.0	0.0
0.0	0.0	0.0	0.0	0.0	0.0	0.0	0.0	0.0
0.0	0.0	0.0	0.0	0.0	0.0	0.0	0.0	0.0
0.0	0.0	0.0	0.0	0.0	0.0	0.0	0.0	0.0
0.0	0.0	0.0	0.0	0.0	0.0	0.0	0.0	0.0
0.0	0.0	0.0	0.0	0.0	0.0	0.0	0.0	0.0
0.0	0.0	0.0	0.0	0.0	0.0	0.0	0.0	0.0
0.0	0.0	0.0	0.0	0.0	0.0	0.0	0.0	0.0
0.0	0.0	0.0	0.0	0.0	0.0	0.0	0.0	0.0
0.0	1.0	0.0	0.0	0.0	0.0	0.0	0.0	0.0
0.0	0.0	0.0	0.0	0.0	0.0	0.0	0.0	0.0

nan	nan	nan	nan	nan	nan	nan	nan	nan
0.0	0.0	0.0	0.0	0.0	1.0	0.0	0.0	0.0
0.0	0.0	0.0	0.0	0.0	0.0	0.0	0.0	0.0
0.0	0.0	0.0	0.0	0.0	0.0	0.0	0.0	0.0
0.0	0.0	0.0	0.0	0.0	1.0	0.0	0.0	0.0
0.0	0.0	1.0	0.0	0.0	0.0	0.0	0.0	0.0
0.0	0.0	0.0	0.0	0.0	0.0	0.0	0.0	0.0
0.0	0.0	0.0	0.0	0.0	0.0	0.0	0.0	0.0
0.0	0.0	0.0	0.0	0.0	0.0	0.0	0.0	0.0
0.0	0.0	0.0	0.0	0.0	0.0	0.0	0.0	0.0
0.0	0.0	0.0	0.0	0.0	0.0	0.0	0.0	0.0
0.0	0.0	0.0	0.0	0.0	1.0	0.0	0.0	1.0
0.0	0.0	0.0	0.0	0.0	0.0	0.0	0.0	0.0
0.0	0.0	0.0	1.0	0.0	1.0	0.0	0.0	0.0
0.0	0.0	0.0	0.0	0.0	0.0	0.0	0.0	0.0
0.0	0.0	0.0	0.0	0.0	0.0	0.0	0.0	1.0
0.0	0.0	0.0	0.0	0.0	0.0	0.0	0.0	1.0
0.0	0.0	1.0	0.0	0.0	0.0	0.0	0.0	0.0
0.0	1.0	0.0	0.0	0.0	0.0	0.0	0.0	0.0
0.0	0.0	0.0	0.0	0.0	0.0	0.0	0.0	0.0
0.0	1.0	0.0	0.0	0.0	0.0	0.0	0.0	0.0
0.0	0.0	0.0	0.0	0.0	0.0	0.0	0.0	0.0
0.0	0.0	0.0	0.0	0.0	0.0	0.0	0.0	1.0
0.0	0.0	0.0	0.0	0.0	0.0	0.0	0.0	1.0
0.0	0.0	0.0	0.0	0.0	0.0	0.0	0.0	0.0
0.0	0.0	0.0	0.0	0.0	0.0	0.0	0.0	0.0
0.0	0.0	0.0	0.0	0.0	0.0	0.0	0.0	0.0
0.0	0.0	0.0	0.0	0.0	0.0	0.0	0.0	0.0
0.0	0.0	0.0	0.0	0.0	1.0	0.0	0.0	0.0
0.0	0.0	0.0	0.0	0.0	1.0	0.0	0.0	0.0
nan	nan	nan	nan	nan	nan	nan	nan	nan
0.0	0.0	1.0	0.0	0.0	0.0	0.0	0.0	0.0
0.0	0.0	0.0	0.0	0.0	1.0	0.0	0.0	0.0
0.0	0.0	0.0	0.0	0.0	0.0	0.0	0.0	1.0
0.0	0.0	0.0	0.0	0.0	1.0	0.0	0.0	0.0
0.0	0.0	0.0	0.0	0.0	0.0	0.0	1.0	0.0
0.0	0.0	0.0	0.0	0.0	0.0	0.0	0.0	0.0
0.0	0.0	0.0	0.0	0.0	0.0	0.0	0.0	0.0
0.0	0.0	1.0	0.0	0.0	0.0	0.0	0.0	0.0
0.0	1.0	0.0	0.0	0.0	0.0	0.0	0.0	0.0
0.0	0.0	0.0	0.0	0.0	0.0	0.0	0.0	0.0
0.0	0.0	0.0	0.0	0.0	0.0	0.0	0.0	0.0
0.0	0.0	0.0	0.0	0.0	0.0	1.0	0.0	0.0
0.0	0.0	0.0	0.0	0.0	0.0	0.0	0.0	0.0
0.0	0.0	0.0	0.0	0.0	0.0	0.0	0.0	0.0
0.0	0.0	0.0	0.0	0.0	0.0	0.0	0.0	0.0
0.0	0.0	0.0	0.0	0.0	0.0	0.0	0.0	0.0

0.0	0.0	0.0	0.0	0.0	0.0	0.0	0.0	0.0
1.0	0.0	0.0	0.0	0.0	0.0	0.0	0.0	0.0
0.0	0.0	0.0	0.0	0.0	0.0	0.0	0.0	0.0
0.0	1.0	0.0	0.0	0.0	0.0	0.0	0.0	0.0
0.0	1.0	0.0	0.0	0.0	0.0	0.0	0.0	0.0
0.0	0.0	0.0	0.0	0.0	0.0	0.0	0.0	0.0
0.0	0.0	0.0	0.0	0.0	0.0	0.0	0.0	0.0
0.0	1.0	0.0	0.0	0.0	0.0	0.0	0.0	0.0
0.0	0.0	0.0	0.0	0.0	0.0	0.0	0.0	0.0
0.0	0.0	0.0	0.0	0.0	0.0	0.0	0.0	0.0
0.0	0.0	0.0	0.0	0.0	0.0	0.0	0.0	0.0
0.0	0.0	0.0	0.0	0.0	0.0	0.0	0.0	0.0
1.0	0.0	0.0	0.0	0.0	1.0	1.0	0.0	0.0
0.0	0.0	0.0	0.0	0.0	0.0	0.0	0.0	0.0
0.0	0.0	0.0	0.0	0.0	1.0	0.0	0.0	0.0
0.0	0.0	0.0	0.0	0.0	0.0	0.0	0.0	0.0
0.0	0.0	0.0	0.0	0.0	0.0	0.0	0.0	0.0
0.0	0.0	0.0	0.0	0.0	0.0	0.0	0.0	0.0
0.0	0.0	0.0	0.0	0.0	1.0	0.0	0.0	1.0
0.0	0.0	0.0	0.0	0.0	0.0	0.0	0.0	0.0
1.0	1.0	0.0	0.0	0.0	0.0	0.0	0.0	0.0
0.0	0.0	0.0	0.0	0.0	0.0	0.0	0.0	0.0
0.0	0.0	0.0	0.0	0.0	0.0	0.0	0.0	0.0

GO:006068	R-HSA-886	R-HSA-886	GO:190101	hsa04730	GO:000170	GO:190157	GO:1900181 negativ
0.0	0.0	0.0	0.0	0.0	1.0	1.0	0.0
0.0	0.0	0.0	0.0	0.0	0.0	0.0	0.0
0.0	0.0	0.0	0.0	0.0	0.0	0.0	0.0
0.0	0.0	0.0	0.0	0.0	0.0	0.0	0.0
0.0	0.0	0.0	0.0	0.0	0.0	0.0	0.0
0.0	0.0	0.0	0.0	0.0	0.0	0.0	0.0
0.0	0.0	0.0	0.0	0.0	0.0	0.0	0.0
0.0	0.0	0.0	0.0	0.0	0.0	0.0	0.0
nan	nan	nan	nan	nan	nan	nan	nan
0.0	0.0	0.0	0.0	0.0	0.0	0.0	0.0
0.0	1.0	0.0	0.0	0.0	0.0	0.0	0.0
0.0	0.0	0.0	0.0	0.0	0.0	0.0	0.0
0.0	0.0	0.0	0.0	0.0	0.0	0.0	0.0
0.0	0.0	0.0	0.0	0.0	0.0	0.0	0.0
0.0	0.0	0.0	0.0	0.0	0.0	0.0	0.0
0.0	0.0	0.0	0.0	0.0	0.0	0.0	0.0
0.0	0.0	0.0	0.0	0.0	0.0	0.0	0.0
nan	nan	nan	nan	nan	nan	nan	nan
0.0	0.0	0.0	0.0	0.0	0.0	0.0	0.0
0.0	0.0	0.0	0.0	0.0	0.0	1.0	0.0
0.0	0.0	0.0	0.0	0.0	0.0	0.0	0.0
0.0	0.0	0.0	0.0	0.0	1.0	0.0	0.0
nan	nan	nan	nan	nan	nan	nan	nan
0.0	0.0	0.0	0.0	1.0	0.0	0.0	0.0
0.0	0.0	0.0	0.0	0.0	0.0	0.0	0.0
0.0	0.0	0.0	0.0	0.0	0.0	0.0	0.0
0.0	0.0	0.0	0.0	0.0	0.0	0.0	0.0
0.0	0.0	1.0	0.0	0.0	0.0	0.0	0.0
nan	nan	nan	nan	nan	nan	nan	nan
0.0	0.0	0.0	0.0	0.0	0.0	0.0	0.0
0.0	0.0	0.0	0.0	0.0	0.0	1.0	0.0
0.0	0.0	0.0	0.0	0.0	0.0	0.0	0.0
0.0	0.0	0.0	0.0	0.0	0.0	0.0	0.0
0.0	0.0	0.0	0.0	0.0	0.0	0.0	0.0
0.0	0.0	0.0	0.0	0.0	0.0	0.0	0.0
0.0	0.0	0.0	0.0	0.0	0.0	0.0	0.0
0.0	0.0	0.0	0.0	0.0	0.0	0.0	0.0
0.0	0.0	0.0	0.0	0.0	0.0	0.0	0.0
0.0	0.0	0.0	0.0	0.0	0.0	0.0	0.0
0.0	0.0	0.0	0.0	0.0	0.0	0.0	0.0
0.0	0.0	0.0	0.0	0.0	0.0	0.0	0.0
0.0	0.0	1.0	0.0	0.0	0.0	0.0	0.0
0.0	0.0	0.0	0.0	0.0	0.0	0.0	0.0
0.0	0.0	0.0	0.0	0.0	0.0	0.0	0.0
0.0	0.0	0.0	0.0	0.0	0.0	0.0	0.0

1.0	0.0	0.0	0.0	0.0	0.0	0.0	0.0
0.0	0.0	1.0	0.0	0.0	0.0	0.0	0.0
0.0	0.0	0.0	0.0	0.0	0.0	0.0	0.0
0.0	0.0	0.0	0.0	0.0	0.0	0.0	0.0
0.0	1.0	0.0	0.0	0.0	0.0	0.0	0.0
0.0	0.0	0.0	0.0	0.0	0.0	0.0	0.0
0.0	0.0	0.0	0.0	0.0	0.0	0.0	0.0
0.0	0.0	0.0	0.0	0.0	0.0	0.0	0.0
0.0	1.0	0.0	0.0	0.0	0.0	1.0	0.0
0.0	0.0	0.0	0.0	0.0	0.0	0.0	0.0
nan	nan	nan	nan	nan	nan	nan	nan
0.0	0.0	0.0	0.0	0.0	0.0	0.0	0.0
0.0	0.0	0.0	0.0	0.0	0.0	0.0	0.0
0.0	0.0	0.0	0.0	0.0	0.0	0.0	0.0
0.0	0.0	0.0	0.0	0.0	0.0	0.0	0.0
0.0	0.0	0.0	0.0	1.0	0.0	0.0	0.0
0.0	0.0	0.0	0.0	0.0	0.0	0.0	0.0
0.0	0.0	0.0	0.0	0.0	0.0	0.0	0.0
0.0	0.0	0.0	0.0	0.0	0.0	0.0	0.0
0.0	0.0	0.0	0.0	0.0	0.0	0.0	0.0
0.0	0.0	0.0	0.0	0.0	0.0	0.0	0.0
0.0	0.0	0.0	0.0	0.0	0.0	0.0	0.0
0.0	0.0	0.0	0.0	0.0	0.0	0.0	0.0
0.0	0.0	0.0	0.0	0.0	0.0	0.0	0.0
0.0	0.0	0.0	0.0	0.0	0.0	0.0	0.0
nan	nan	nan	nan	nan	nan	nan	nan
0.0	0.0	0.0	0.0	0.0	0.0	0.0	0.0
0.0	0.0	0.0	0.0	0.0	0.0	0.0	0.0
0.0	0.0	0.0	0.0	0.0	0.0	0.0	0.0
0.0	0.0	0.0	0.0	0.0	0.0	0.0	0.0
0.0	0.0	0.0	0.0	0.0	0.0	0.0	0.0
0.0	0.0	1.0	0.0	0.0	0.0	0.0	0.0
0.0	0.0	0.0	0.0	0.0	0.0	0.0	0.0
0.0	0.0	0.0	0.0	0.0	0.0	0.0	0.0
0.0	0.0	0.0	0.0	0.0	0.0	0.0	0.0
0.0	0.0	0.0	0.0	0.0	0.0	0.0	0.0
0.0	0.0	1.0	0.0	0.0	0.0	0.0	0.0
0.0	0.0	0.0	0.0	0.0	0.0	0.0	0.0
0.0	0.0	0.0	0.0	0.0	0.0	0.0	0.0
0.0	0.0	0.0	0.0	0.0	0.0	0.0	0.0
0.0	0.0	0.0	0.0	0.0	0.0	0.0	0.0
0.0	0.0	0.0	0.0	0.0	0.0	0.0	0.0
0.0	0.0	0.0	0.0	0.0	0.0	0.0	0.0
0.0	0.0	0.0	0.0	0.0	0.0	1.0	0.0
0.0	0.0	0.0	0.0	1.0	0.0	0.0	0.0
0.0	0.0	0.0	0.0	0.0	0.0	0.0	0.0
1.0	0.0	0.0	0.0	0.0	0.0	1.0	0.0

0.0	0.0	0.0	0.0	0.0	0.0	0.0	0.0
0.0	0.0	0.0	0.0	0.0	0.0	0.0	0.0
0.0	0.0	0.0	0.0	0.0	0.0	0.0	0.0
0.0	0.0	0.0	0.0	0.0	0.0	0.0	0.0
0.0	0.0	0.0	0.0	0.0	0.0	0.0	0.0
0.0	0.0	0.0	0.0	0.0	0.0	0.0	0.0
0.0	0.0	0.0	0.0	0.0	0.0	0.0	0.0
0.0	0.0	0.0	0.0	0.0	0.0	0.0	0.0
0.0	0.0	0.0	0.0	0.0	0.0	0.0	0.0
0.0	0.0	0.0	0.0	0.0	0.0	0.0	0.0
0.0	0.0	0.0	0.0	0.0	0.0	0.0	0.0
0.0	0.0	0.0	0.0	0.0	0.0	0.0	0.0
0.0	0.0	0.0	0.0	0.0	0.0	0.0	0.0
1.0	0.0	0.0	0.0	0.0	0.0	0.0	0.0
0.0	0.0	0.0	0.0	0.0	0.0	0.0	0.0
0.0	0.0	0.0	0.0	0.0	0.0	0.0	0.0
0.0	0.0	0.0	0.0	0.0	0.0	0.0	0.0
0.0	1.0	0.0	0.0	0.0	0.0	1.0	0.0
0.0	0.0	0.0	0.0	0.0	0.0	0.0	0.0
0.0	0.0	0.0	0.0	0.0	0.0	0.0	0.0
0.0	0.0	0.0	0.0	0.0	0.0	0.0	0.0
0.0	0.0	0.0	0.0	0.0	0.0	0.0	0.0
0.0	0.0	0.0	0.0	0.0	0.0	0.0	0.0
0.0	0.0	0.0	0.0	0.0	0.0	0.0	1.0
0.0	0.0	0.0	0.0	0.0	0.0	0.0	0.0
1.0	0.0	0.0	0.0	0.0	1.0	0.0	0.0
0.0	0.0	0.0	0.0	0.0	0.0	0.0	0.0
0.0	0.0	0.0	0.0	0.0	0.0	0.0	0.0
0.0	0.0	0.0	0.0	0.0	0.0	0.0	0.0
nan	nan	nan	nan	nan	nan	nan	nan
0.0	0.0	0.0	0.0	0.0	0.0	0.0	0.0
0.0	1.0	0.0	0.0	0.0	0.0	0.0	0.0
0.0	0.0	0.0	0.0	0.0	0.0	0.0	0.0
0.0	0.0	0.0	0.0	0.0	0.0	0.0	0.0
0.0	0.0	0.0	0.0	0.0	0.0	0.0	0.0
0.0	0.0	0.0	0.0	0.0	0.0	0.0	0.0
0.0	0.0	0.0	0.0	0.0	0.0	0.0	0.0
0.0	0.0	0.0	0.0	0.0	0.0	0.0	0.0
0.0	0.0	0.0	0.0	0.0	0.0	0.0	0.0
0.0	0.0	0.0	0.0	0.0	0.0	0.0	0.0
0.0	0.0	0.0	0.0	0.0	0.0	0.0	0.0
0.0	0.0	0.0	0.0	0.0	0.0	0.0	0.0
0.0	0.0	0.0	0.0	0.0	0.0	0.0	0.0
0.0	0.0	0.0	0.0	0.0	0.0	0.0	0.0
0.0	0.0	0.0	0.0	0.0	0.0	0.0	0.0
0.0	0.0	0.0	0.0	0.0	0.0	0.0	0.0
0.0	0.0	0.0	0.0	0.0	0.0	0.0	0.0

0.0	0.0	0.0	0.0	0.0	0.0	0.0	0.0
0.0	0.0	0.0	0.0	0.0	0.0	0.0	0.0
0.0	0.0	0.0	0.0	0.0	1.0	0.0	0.0
0.0	0.0	0.0	0.0	0.0	0.0	0.0	0.0
1.0	0.0	0.0	0.0	0.0	1.0	0.0	0.0
0.0	0.0	0.0	0.0	0.0	0.0	0.0	0.0
0.0	0.0	0.0	0.0	0.0	0.0	0.0	0.0
0.0	0.0	0.0	0.0	0.0	0.0	0.0	0.0
0.0	0.0	0.0	0.0	0.0	0.0	0.0	0.0
0.0	0.0	0.0	0.0	0.0	0.0	0.0	0.0
0.0	0.0	0.0	0.0	0.0	0.0	0.0	0.0
0.0	0.0	0.0	0.0	0.0	0.0	0.0	0.0
0.0	0.0	0.0	0.0	0.0	0.0	0.0	0.0
0.0	0.0	0.0	0.0	0.0	0.0	0.0	0.0
0.0	0.0	0.0	0.0	0.0	0.0	0.0	0.0
nan	nan	nan	nan	nan	nan	nan	nan
0.0	0.0	0.0	0.0	0.0	0.0	0.0	0.0
0.0	0.0	0.0	0.0	0.0	0.0	0.0	0.0
0.0	0.0	0.0	0.0	0.0	0.0	0.0	0.0
0.0	0.0	0.0	0.0	0.0	0.0	0.0	0.0
0.0	0.0	0.0	0.0	0.0	0.0	0.0	0.0
0.0	0.0	0.0	0.0	0.0	0.0	0.0	0.0
0.0	1.0	0.0	0.0	0.0	0.0	0.0	0.0
0.0	0.0	0.0	0.0	0.0	0.0	0.0	0.0
0.0	0.0	0.0	0.0	0.0	0.0	0.0	0.0
1.0	0.0	0.0	0.0	0.0	1.0	0.0	0.0
1.0	0.0	0.0	0.0	0.0	0.0	0.0	0.0
1.0	0.0	1.0	0.0	0.0	0.0	0.0	0.0
0.0	0.0	0.0	0.0	0.0	0.0	0.0	0.0
0.0	0.0	0.0	0.0	0.0	0.0	0.0	0.0
0.0	0.0	0.0	0.0	0.0	0.0	0.0	0.0
0.0	0.0	0.0	0.0	0.0	0.0	0.0	0.0
0.0	0.0	0.0	0.0	0.0	0.0	0.0	0.0
0.0	0.0	0.0	0.0	0.0	0.0	0.0	0.0
0.0	0.0	0.0	0.0	0.0	0.0	0.0	0.0
0.0	0.0	0.0	0.0	0.0	0.0	0.0	0.0
0.0	0.0	0.0	0.0	0.0	0.0	0.0	0.0
0.0	0.0	0.0	0.0	0.0	0.0	0.0	0.0
0.0	0.0	0.0	0.0	0.0	0.0	0.0	0.0
0.0	0.0	0.0	0.0	0.0	0.0	0.0	0.0
0.0	0.0	0.0	0.0	0.0	0.0	0.0	0.0
0.0	0.0	0.0	0.0	0.0	0.0	0.0	0.0
0.0	0.0	0.0	0.0	0.0	0.0	0.0	0.0
0.0	0.0	0.0	0.0	0.0	0.0	0.0	0.0
0.0	0.0	0.0	0.0	0.0	0.0	0.0	0.0
0.0	0.0	0.0	0.0	0.0	0.0	0.0	0.0
0.0	0.0	0.0	0.0	0.0	0.0	1.0	0.0

nan	nan	nan	nan	nan	nan	nan	nan
0.0	0.0	0.0	0.0	0.0	1.0	0.0	0.0
0.0	0.0	1.0	0.0	0.0	0.0	0.0	0.0
0.0	0.0	0.0	0.0	0.0	0.0	0.0	0.0
0.0	0.0	0.0	0.0	0.0	0.0	0.0	0.0
0.0	0.0	0.0	0.0	0.0	0.0	0.0	0.0
0.0	0.0	0.0	0.0	0.0	0.0	0.0	0.0
1.0	0.0	0.0	0.0	0.0	0.0	0.0	0.0
0.0	0.0	0.0	0.0	0.0	0.0	0.0	0.0
0.0	0.0	0.0	0.0	0.0	0.0	0.0	0.0
0.0	0.0	0.0	1.0	0.0	0.0	0.0	0.0
1.0	0.0	1.0	0.0	0.0	1.0	0.0	0.0
0.0	0.0	0.0	0.0	0.0	0.0	0.0	1.0
0.0	0.0	0.0	0.0	0.0	0.0	0.0	0.0
0.0	0.0	0.0	0.0	0.0	0.0	0.0	0.0
0.0	0.0	0.0	0.0	0.0	0.0	0.0	0.0
1.0	0.0	0.0	1.0	0.0	0.0	0.0	0.0
0.0	0.0	0.0	0.0	0.0	0.0	0.0	0.0
0.0	0.0	0.0	0.0	0.0	1.0	0.0	0.0
0.0	0.0	0.0	0.0	0.0	0.0	0.0	0.0
0.0	1.0	0.0	0.0	0.0	0.0	1.0	0.0
0.0	0.0	1.0	0.0	0.0	0.0	0.0	0.0
0.0	0.0	0.0	1.0	0.0	0.0	0.0	0.0
1.0	0.0	0.0	0.0	0.0	0.0	0.0	0.0
1.0	0.0	0.0	0.0	0.0	1.0	0.0	0.0
0.0	0.0	0.0	0.0	0.0	0.0	0.0	0.0
0.0	0.0	0.0	0.0	0.0	0.0	0.0	0.0
0.0	0.0	0.0	0.0	0.0	0.0	0.0	0.0
0.0	0.0	0.0	0.0	0.0	1.0	0.0	0.0
1.0	0.0	0.0	0.0	0.0	0.0	0.0	1.0
nan	nan	nan	nan	nan	nan	nan	nan
0.0	0.0	0.0	0.0	0.0	0.0	0.0	0.0
0.0	0.0	0.0	0.0	0.0	0.0	0.0	0.0
0.0	0.0	0.0	0.0	0.0	0.0	0.0	0.0
1.0	0.0	0.0	0.0	0.0	1.0	0.0	0.0
0.0	0.0	0.0	0.0	0.0	0.0	0.0	0.0
0.0	1.0	0.0	0.0	0.0	0.0	0.0	0.0
0.0	0.0	0.0	0.0	0.0	0.0	0.0	0.0
0.0	0.0	0.0	0.0	0.0	1.0	0.0	0.0
0.0	0.0	0.0	0.0	0.0	0.0	1.0	0.0
0.0	0.0	0.0	0.0	0.0	0.0	0.0	0.0
0.0	0.0	0.0	0.0	0.0	0.0	0.0	0.0
0.0	0.0	0.0	0.0	0.0	0.0	0.0	0.0
0.0	0.0	0.0	0.0	0.0	0.0	1.0	0.0
0.0	0.0	0.0	0.0	0.0	0.0	0.0	0.0
0.0	0.0	0.0	0.0	0.0	0.0	0.0	0.0
0.0	0.0	0.0	0.0	0.0	0.0	0.0	0.0

0.0	0.0	0.0	0.0	0.0	0.0	1.0	0.0
0.0	0.0	0.0	0.0	0.0	0.0	0.0	0.0
0.0	0.0	0.0	0.0	0.0	0.0	0.0	0.0
0.0	0.0	0.0	0.0	0.0	0.0	1.0	0.0
0.0	0.0	0.0	0.0	0.0	0.0	0.0	0.0
0.0	0.0	0.0	0.0	0.0	0.0	0.0	0.0
0.0	0.0	0.0	0.0	0.0	0.0	0.0	0.0
0.0	0.0	0.0	0.0	0.0	0.0	1.0	0.0
1.0	0.0	0.0	0.0	0.0	0.0	0.0	0.0
0.0	0.0	0.0	0.0	0.0	1.0	0.0	0.0
0.0	0.0	0.0	0.0	0.0	0.0	0.0	0.0
0.0	0.0	0.0	0.0	0.0	0.0	0.0	0.0
1.0	0.0	0.0	0.0	0.0	1.0	0.0	0.0
0.0	0.0	0.0	0.0	1.0	0.0	0.0	0.0
0.0	0.0	0.0	0.0	0.0	0.0	0.0	0.0
0.0	0.0	0.0	0.0	0.0	0.0	0.0	0.0
0.0	0.0	0.0	0.0	0.0	0.0	0.0	0.0
0.0	0.0	0.0	0.0	0.0	0.0	0.0	0.0
0.0	0.0	0.0	0.0	0.0	0.0	0.0	0.0
0.0	0.0	0.0	0.0	0.0	0.0	0.0	0.0
0.0	1.0	0.0	0.0	0.0	0.0	1.0	0.0
0.0	0.0	0.0	1.0	0.0	0.0	0.0	0.0
0.0	0.0	0.0	0.0	0.0	0.0	0.0	0.0

e regulation of protein

Supplementary Table s5 : Enrichment of candidate genes in UC goats and other cashmere goats to perform sel

GroupID	Category	Term	Description	LogP	Log(q-val
1_Summar	GO Biologi	GO:000724	I-kappaB kinase/NF-kappaB signaling	-6.84047	-2.725
1_Member	GO Biologi	GO:000724	I-kappaB kinase/NF-kappaB signaling	-6.84047	-2.725
1_Member	GO Biologi	GO:004312	regulation of I-kappaB kinase/NF-kappaB signaling	-6.74309	-2.725
1_Member	GO Biologi	GO:004312	positive regulation of I-kappaB kinase/NF-kappaB signal	-6.44435	-2.602
1_Member	GO Biologi	GO:000275	innate immune response-activating signal transduction	-4.96185	-1.245
1_Member	GO Biologi	GO:000221	activation of innate immune response	-4.62509	-1.005
1_Member	GO Biologi	GO:003134	positive regulation of defense response	-4.18227	-0.718
1_Member	Canonical P	M264	PID TOLL ENDOGENOUS PATHWAY	-3.99191	-0.718
1_Member	Reactome	R-HSA-937	IKK complex recruitment mediated by RIP1	-3.99191	-0.718
1_Member	CORUM	CORUM:51	TNF-alpha/NF-kappa B signaling complex (SEC16A, CHU	-3.95755	-0.718
1_Member	GO Biologi	GO:004508	positive regulation of innate immune response	-3.89867	-0.694
1_Member	GO Biologi	GO:000222	pattern recognition receptor signaling pathway	-3.73458	-0.683
1_Member	CORUM	CORUM:51	TNF-alpha/NF-kappa B signaling complex (CHUK, BTRC,	-3.7007	-0.683
1_Member	Reactome	R-HSA-168	Toll Like Receptor 9 (TLR9) Cascade	-3.37728	-0.427
1_Member	Reactome	R-HSA-560	CLEC7A (Dectin-1) signaling	-3.2835	-0.411
1_Member	Reactome	R-HSA-562	C-type lectin receptors (CLRs)	-3.23538	-0.411
1_Member	CORUM	CORUM:52	TNF-alpha/Nf-kappa B signaling complex (RPL6, RPL30,	-3.22656	-0.411
1_Member	GO Biologi	GO:004508	regulation of innate immune response	-3.20801	-0.411
1_Member	GO Biologi	GO:005109	positive regulation of NF-kappaB transcription factor ac	-3.06182	-0.311
1_Member	Reactome	R-HSA-168	TICAM1, RIP1-mediated IKK complex recruitment	-3.01172	-0.272
1_Member	Reactome	R-HSA-168	Toll-like Receptor Cascades	-2.98021	-0.252
1_Member	GO Biologi	GO:007055	response to interleukin-1	-2.93307	-0.227
1_Member	Reactome	R-HSA-202	TCR signaling	-2.89147	-0.220
1_Member	GO Biologi	GO:003109	stress-activated protein kinase signaling cascade	-2.87354	-0.220
1_Member	Reactome	R-HSA-983	Class I MHC mediated antigen processing & presentatio	-2.87323	-0.220
1_Member	Reactome	R-HSA-287	FCERI mediated NF-kB activation	-2.86692	-0.220
1_Member	GO Biologi	GO:005109	regulation of DNA-binding transcription factor activity	-2.79832	-0.182
1_Member	Reactome	R-HSA-123	ER-Phagosome pathway	-2.79738	-0.182
1_Member	CORUM	CORUM:52	TNF-alpha/NF-kappa B signaling complex 5	-2.72279	-0.175
1_Member	Reactome	R-HSA-166	Toll Like Receptor 4 (TLR4) Cascade	-2.69731	-0.171
1_Member	Reactome	R-HSA-975	TRAF6 mediated induction of NFkB and MAP kinases up	-2.64581	-0.151
1_Member	Reactome	R-HSA-168	Toll Like Receptor 7/8 (TLR7/8) Cascade	-2.62528	-0.151
1_Member	Reactome	R-HSA-975	MyD88 dependent cascade initiated on endosome	-2.62528	-0.151
1_Member	Reactome	R-HSA-526	Diseases of Immune System	-2.62474	-0.151
1_Member	Reactome	R-HSA-560	Diseases associated with the TLR signaling cascade	-2.62474	-0.151
1_Member	GO Biologi	GO:003806	NIK/NF-kappaB signaling	-2.56339	-0.133
1_Member	Reactome	R-HSA-166	MyD88:MAL(TIRAP) cascade initiated on plasma memb	-2.54571	-0.133
1_Member	Reactome	R-HSA-168	Toll Like Receptor TLR6:TLR2 Cascade	-2.54571	-0.133
1_Member	GO Biologi	GO:003566	TRIF-dependent toll-like receptor signaling pathway	-2.53443	-0.133
1_Member	Reactome	R-HSA-202	Downstream TCR signaling	-2.52643	-0.133
1_Member	Reactome	R-HSA-166	MyD88-independent TLR4 cascade	-2.50738	-0.133
1_Member	Reactome	R-HSA-937	TRIF(TICAM1)-mediated TLR4 signaling	-2.50738	-0.133
1_Member	GO Biologi	GO:007049	interleukin-1-mediated signaling pathway	-2.48857	-0.133
1_Member	Reactome	R-HSA-168	Toll Like Receptor TLR1:TLR2 Cascade	-2.48857	-0.133
1_Member	Reactome	R-HSA-181	Toll Like Receptor 2 (TLR2) Cascade	-2.48857	-0.133

1_Member	Reactome (	R-HSA-123(	Antigen processing-Cross presentation		-2.46998	-0.119
1_Member	Reactome (	R-HSA-902(	Interleukin-1 signaling		-2.43346	-0.106
1_Member	GO Biologi	GO:000275	MyD88-independent toll-like receptor signaling pathwa		-2.37299	-0.083
1_Member	GO Biologi	GO:003461	response to tumor necrosis factor		-2.35424	-0.077
1_Member	Reactome (	R-HSA-116	Activation of NF-kappaB in B cells		-2.35078	-0.077
1_Member	Canonical F	M110	PID IL1 PATHWAY		-2.33603	-0.077
1_Member	GO Biologi	GO:005109	positive regulation of DNA-binding transcription factor a		-2.27693	-0.050
1_Member	GO Biologi	GO:000222	stimulatory C-type lectin receptor signaling pathway		-2.26296	-0.050
1_Member	GO Biologi	GO:000275	immune response-activating signal transduction		-2.25895	-0.050
1_Member	Reactome (	R-HSA-168(	NOD1/2 Signaling Pathway		-2.23208	-0.039
1_Member	GO Biologi	GO:000222	innate immune response activating cell surface recepto		-2.21539	-0.030
1_Member	GO Biologi	GO:000225	activation of immune response		-2.17796	0.000
1_Member	GO Biologi	GO:005140	stress-activated MAPK cascade		-2.0857	0.000
1_Member	GO Biologi	GO:000276	immune response-regulating signaling pathway		-2.07229	0.000
1_Member	GO Biologi	GO:003247	regulation of type I interferon production		-2.05321	0.000
1_Member	GO Biologi	GO:007134	cellular response to interleukin-1		-2.02855	0.000
1_Member	GO Biologi	GO:003260	type I interferon production		-2.02561	0.000
1_Member	Reactome (	R-HSA-116	Downstream signaling events of B Cell Receptor (BCR)		-2.02432	0.000
1_Member	GO Biologi	GO:000223	response to molecule of bacterial origin		-2.00548	0.000
2_Summar	GO Biologi	GO:006007	canonical Wnt signaling pathway		-4.40852	-0.868
2_Member	GO Biologi	GO:006007	canonical Wnt signaling pathway		-4.40852	-0.868
2_Member	Canonical F	M223	PID BETA CATENIN NUC PATHWAY		-3.83348	-0.683
2_Member	GO Biologi	GO:006082	regulation of canonical Wnt signaling pathway		-3.73196	-0.683
2_Member	Reactome (	R-HSA-464	Repression of WNT target genes		-3.7007	-0.683
2_Member	GO Biologi	GO:003011	regulation of Wnt signaling pathway		-3.38835	-0.427
2_Member	GO Biologi	GO:009009	negative regulation of canonical Wnt signaling pathway		-3.26343	-0.411
2_Member	GO Biologi	GO:190511	cell surface receptor signaling pathway involved in cell-c		-3.21271	-0.411
2_Member	GO Biologi	GO:001605	Wnt signaling pathway		-3.08353	-0.311
2_Member	GO Biologi	GO:019873	cell-cell signaling by wnt		-3.06768	-0.311
2_Member	Reactome (	R-HSA-195	Degradation of beta-catenin by the destruction comple:		-2.84341	-0.206
2_Member	GO Biologi	GO:003017	negative regulation of Wnt signaling pathway		-2.7788	-0.182
2_Member	Reactome (	R-HSA-376	Deactivation of the beta-catenin transactivating comple		-2.07831	0.000
3_Summar	GO Biologi	GO:003509	maintenance of apical/basal cell polarity		-3.95755	-0.718
3_Member	GO Biologi	GO:003509	maintenance of apical/basal cell polarity		-3.95755	-0.718
3_Member	GO Biologi	GO:004519	maintenance of epithelial cell apical/basal polarity		-3.95755	-0.718
3_Member	GO Biologi	GO:003001	maintenance of cell polarity		-3.22656	-0.411
4_Summar	Reactome (	R-HSA-400	Free fatty acids regulate insulin secretion		-3.82244	-0.683
4_Member	Reactome (	R-HSA-400	Free fatty acids regulate insulin secretion		-3.82244	-0.683
4_Member	Canonical F	M155	PID S1P META PATHWAY		-2.94795	-0.231
4_Member	Reactome (	R-HSA-428	Thromboxane signalling through TP receptor		-2.77513	-0.182
4_Member	Canonical F	M99	PID TXA2PATHWAY		-2.60521	-0.139
4_Member	Reactome (	R-HSA-392	Signal amplification		-2.37299	-0.083
4_Member	Canonical F	M19043	ST ADRENERGIC		-2.26564	-0.050
4_Member	Canonical F	M41	PID ER NONGENOMIC PATHWAY		-2.10735	0.000
5_Summar	GO Biologi	GO:003025	lipid modification		-3.74284	-0.683
5_Member	GO Biologi	GO:003025	lipid modification		-3.74284	-0.683
5_Member	Reactome (	R-HSA-556	Metabolism of lipids		-2.51242	-0.133

5_Member	Reactome (	R-HSA-148:	Phospholipid metabolism	-2.26592	-0.050
5_Member	KEGG Path	hsa00562	Inositol phosphate metabolism	-2.26113	-0.050
5_Member	GO Biologi	GO:004648	glycerolipid metabolic process	-2.04454	0.000
5_Member	Reactome (	R-HSA-148:	PI Metabolism	-2.00645	0.000
6_Summar	GO Biologi	GO:004362	cellular protein complex disassembly	-3.40024	-0.427
6_Member	GO Biologi	GO:004362	cellular protein complex disassembly	-3.40024	-0.427
6_Member	GO Biologi	GO:000641	translational elongation	-3.36597	-0.427
6_Member	GO Biologi	GO:003298	protein-containing complex disassembly	-3.19869	-0.411
6_Member	CORUM	CORUM:32	39S ribosomal subunit, mitochondrial	-2.88251	-0.220
6_Member	Reactome (	R-HSA-536	Mitochondrial translation initiation	-2.75261	-0.182
6_Member	Reactome (	R-HSA-538	Mitochondrial translation elongation	-2.75261	-0.182
6_Member	Reactome (	R-HSA-541	Mitochondrial translation termination	-2.75261	-0.182
6_Member	GO Biologi	GO:007012	mitochondrial translational elongation	-2.70903	-0.175
6_Member	GO Biologi	GO:007012	mitochondrial translational termination	-2.70903	-0.175
6_Member	Reactome (	R-HSA-536	Mitochondrial translation	-2.62528	-0.151
6_Member	GO Biologi	GO:000641	translational termination	-2.39779	-0.096
6_Member	CORUM	CORUM:32	55S ribosome, mitochondrial	-2.13724	0.000
6_Member	Reactome (	R-HSA-727	Translation	-2.0515	0.000
7_Summar	GO Biologi	GO:00068	post-Golgi vesicle-mediated transport	-3.23824	-0.411
7_Member	GO Biologi	GO:00068	post-Golgi vesicle-mediated transport	-3.23824	-0.411
7_Member	GO Biologi	GO:009887	vesicle-mediated transport to the plasma membrane	-2.58499	-0.133
7_Member	GO Biologi	GO:00068	Golgi to plasma membrane transport	-2.49766	-0.133
7_Member	GO Biologi	GO:00430	Golgi to plasma membrane protein transport	-2.10735	0.000
8_Summar	Reactome (	R-HSA-533	Uptake and actions of bacterial toxins	-3.22617	-0.411
8_Member	Reactome (	R-HSA-533	Uptake and actions of bacterial toxins	-3.22617	-0.411
8_Member	KEGG Path	hsa05219	Bladder cancer	-2.10735	0.000
9_Summar	GO Biologi	GO:004858	developmental growth	-2.87306	-0.220
9_Member	GO Biologi	GO:004858	developmental growth	-2.87306	-0.220
9_Member	GO Biologi	GO:006056	developmental growth involved in morphogenesis	-2.60077	-0.139
9_Member	GO Biologi	GO:004863	regulation of developmental growth	-2.55996	-0.133
9_Member	GO Biologi	GO:004881	neuron projection morphogenesis	-2.19679	-0.014
9_Member	GO Biologi	GO:004866	cell morphogenesis involved in neuron differentiation	-2.14918	0.000
9_Member	GO Biologi	GO:012003	plasma membrane bounded cell projection morphogen	-2.12015	0.000
9_Member	GO Biologi	GO:004885	cell projection morphogenesis	-2.09875	0.000
9_Member	GO Biologi	GO:00740	axonogenesis	-2.06484	0.000
9_Member	GO Biologi	GO:00090	cell morphogenesis involved in differentiation	-2.05104	0.000
9_Member	GO Biologi	GO:004858	developmental cell growth	-2.02137	0.000
10_Summa	KEGG Path	hsa05215	Prostate cancer	-2.75261	-0.182
10_Membe	KEGG Path	hsa05215	Prostate cancer	-2.75261	-0.182
11_Summa	GO Biologi	GO:004566	negative regulation of myoblast differentiation	-2.72279	-0.175
11_Membe	GO Biologi	GO:004566	negative regulation of myoblast differentiation	-2.72279	-0.175
11_Membe	GO Biologi	GO:004566	regulation of myoblast differentiation	-2.72179	-0.175
12_Summa	GO Biologi	GO:000734	regulation of mitotic cell cycle	-2.63524	-0.151
12_Membe	GO Biologi	GO:000734	regulation of mitotic cell cycle	-2.63524	-0.151
13_Summa	GO Biologi	GO:006068	regulation of morphogenesis of a branching structure	-2.63346	-0.151
13_Membe	GO Biologi	GO:006068	regulation of morphogenesis of a branching structure	-2.63346	-0.151
13_Membe	GO Biologi	GO:003514	tube formation	-2.40525	-0.096

13_Membe	GO Biologi	GO:190533	positive regulation of morphogenesis of an epithelium	-2.26564	-0.050
13_Membe	GO Biologi	GO:006056	epithelial tube morphogenesis	-2.25027	-0.045
13_Membe	GO Biologi	GO:005067	epithelial cell proliferation	-2.21763	-0.030
14_Summa	Reactome (	R-HSA-886(	E3 ubiquitin ligases ubiquitinate target proteins	-2.55039	-0.133
14_Membe	Reactome (	R-HSA-886(	E3 ubiquitin ligases ubiquitinate target proteins	-2.55039	-0.133
14_Membe	Reactome (	R-HSA-903:	Peroxisomal protein import	-2.4469	-0.106
14_Membe	GO Biologi	GO:000662	protein targeting to peroxisome	-2.32779	-0.077
14_Membe	GO Biologi	GO:007266	protein localization to peroxisome	-2.32779	-0.077
14_Membe	GO Biologi	GO:007266	establishment of protein localization to peroxisome	-2.32779	-0.077
14_Membe	GO Biologi	GO:004357	peroxisomal transport	-2.30519	-0.062
14_Membe	Reactome (	R-HSA-960!	Protein localization	-2.23104	-0.039
14_Membe	Reactome (	R-HSA-885:	Protein ubiquitination	-2.09847	0.000
14_Membe	GO Biologi	GO:000703	peroxisome organization	-2.06085	0.000
14_Membe	KEGG Path	hsa04146	Peroxisome	-2.02432	0.000
15_Summa	Reactome (	R-HSA-886:	Downregulation of ERBB2 signaling	-2.53443	-0.133
15_Membe	Reactome (	R-HSA-886:	Downregulation of ERBB2 signaling	-2.53443	-0.133
15_Membe	Reactome (	R-HSA-681:	PI5P, PP2A and IER3 Regulate PI3K/AKT Signaling	-2.43346	-0.106
15_Membe	GO Biologi	GO:003812	ERBB2 signaling pathway	-2.41122	-0.096
15_Membe	Reactome (	R-HSA-199	Negative regulation of the PI3K/AKT network	-2.31211	-0.065
15_Membe	GO Biologi	GO:005189	positive regulation of protein kinase B signaling	-2.06242	0.000
16_Summa	GO Biologi	GO:190101	negative regulation of potassium ion transmembrane tr	-2.53443	-0.133
16_Membe	GO Biologi	GO:190101	negative regulation of potassium ion transmembrane tr	-2.53443	-0.133
16_Membe	GO Biologi	GO:190101	regulation of potassium ion transmembrane transporte	-2.39798	-0.096
16_Membe	GO Biologi	GO:190138	negative regulation of potassium ion transmembrane tr	-2.37299	-0.083
16_Membe	GO Biologi	GO:004326	negative regulation of potassium ion transport	-2.10735	0.000
17_Summa	KEGG Path	hsa04730	Long-term depression	-2.52377	-0.133
17_Membe	KEGG Path	hsa04730	Long-term depression	-2.52377	-0.133
18_Summa	GO Biologi	GO:00017C	cell fate specification	-2.48857	-0.133
18_Membe	GO Biologi	GO:00017C	cell fate specification	-2.48857	-0.133
18_Membe	GO Biologi	GO:004247	inner ear morphogenesis	-2.43346	-0.106
18_Membe	GO Biologi	GO:003085	positive regulation of epithelial cell differentiation	-2.32779	-0.077
18_Membe	GO Biologi	GO:004856	embryonic organ development	-2.28784	-0.050
18_Membe	GO Biologi	GO:004249	inner ear auditory receptor cell differentiation	-2.26564	-0.050
18_Membe	GO Biologi	GO:004859	embryonic morphogenesis	-2.13762	0.000
18_Membe	GO Biologi	GO:004247	ear morphogenesis	-2.11008	0.000
18_Membe	GO Biologi	GO:00017C	cell fate determination	-2.07831	0.000
18_Membe	GO Biologi	GO:003531	hair cell differentiation	-2.02253	0.000
19_Summa	GO Biologi	GO:190157	fatty acid derivative biosynthetic process	-2.48857	-0.133
19_Membe	GO Biologi	GO:190157	fatty acid derivative biosynthetic process	-2.48857	-0.133
19_Membe	GO Biologi	GO:000167	long-chain fatty acid metabolic process	-2.32886	-0.077
19_Membe	GO Biologi	GO:003278	monocarboxylic acid metabolic process	-2.23615	-0.039
19_Membe	GO Biologi	GO:003355	unsaturated fatty acid metabolic process	-2.23108	-0.039
19_Membe	GO Biologi	GO:007233	monocarboxylic acid biosynthetic process	-2.13569	0.000
19_Membe	GO Biologi	GO:190156	fatty acid derivative metabolic process	-2.12058	0.000
19_Membe	GO Biologi	GO:003533	fatty-acyl-CoA metabolic process	-2.10735	0.000
19_Membe	Reactome (	R-HSA-897	Fatty acid metabolism	-2.06242	0.000
20_Summa	GO Biologi	GO:190018	negative regulation of protein localization to nucleus	-2.4508	-0.106

20_Membe	GO Biologic	GO:190018	negative regulation of protein localization to nucleus	-2.4508	-0.106

ective evolution analysis

InTerm_In	Genes	Symbols
15/279	929,1147,1	CD14,CHUK,EDA,MYD88,REL,UBE2N,BTRC,NEK6,CXXC5,TNFRSF19,CACTIN,CARD
15/279	929,1147,1	CD14,CHUK,EDA,MYD88,REL,UBE2N,BTRC,NEK6,CXXC5,TNFRSF19,CACTIN,CARD
14/245	1147,1896,	CHUK,EDA,MYD88,REL,UBE2N,BTRC,NEK6,CXXC5,TNFRSF19,CACTIN,CARD9,ALPK
12/185	1147,1896,	CHUK,EDA,MYD88,REL,UBE2N,NEK6,CXXC5,TNFRSF19,CARD9,ALPK1,MTDH,TIFA
13/301	929,1147,4	CD14,CHUK,MYD88,PAK3,PSMB7,UBE2D2,UBE2N,BTRC,DDX60,CACTIN,CARD9,A
13/324	929,1147,4	CD14,CHUK,MYD88,PAK3,PSMB7,UBE2D2,UBE2N,BTRC,DDX60,CACTIN,CARD9,A
16/517	929,1147,4	CD14,CHUK,MYD88,PAK3,PSMB7,UBE2D2,UBE2N,VAMP8,BTRC,DDX60,CACTIN,
4/25	929,1147,1	CD14,CHUK,VCAN,MYD88
4/25	929,1147,7	CD14,CHUK,UBE2D2,UBE2N
3/10	1147,5966,	CHUK,REL,SEC16A
13/382	929,1147,4	CD14,CHUK,MYD88,PAK3,PSMB7,UBE2D2,UBE2N,BTRC,DDX60,CACTIN,CARD9,A
9/200	929,1147,4	CD14,CHUK,MYD88,UBE2D2,UBE2N,DDX60,CACTIN,ALPK1,TIFA
3/12	1147,5966,	CHUK,REL,BTRC
6/97	929,1147,4	CD14,CHUK,MYD88,UBE2N,EEA1,BTRC
6/101	1147,5695,	CHUK,PSMB7,UBE2D2,UBE2N,BTRC,CARD9
7/143	1147,5063,	CHUK,PAK3,PSMB7,UBE2D2,UBE2N,BTRC,CARD9
3/17	1147,5966,	CHUK,REL,RPL30
13/451	929,1147,4	CD14,CHUK,MYD88,PAK3,PSMB7,UBE2D2,UBE2N,BTRC,DDX60,CACTIN,CARD9,A
7/153	367,1147,1	AR,CHUK,EDA,MYD88,UBE2N,ZBTB7A,MTDH
3/20	1147,7322,	CHUK,UBE2D2,UBE2N
7/158	929,1147,4	CD14,CHUK,MYD88,UBE2D2,UBE2N,EEA1,BTRC
8/208	166,1147,3	TLE5,CHUK,IGBP1,MYD88,PSMB7,UBE2N,BTRC,CACTIN
6/120	1147,5063,	CHUK,PAK3,PSMB7,UBE2D2,UBE2N,BTRC
10/317	1147,3476,	CHUK,IGBP1,PAK3,MAP2K2,STK3,UBE2N,BTRC,OXSR1,TNFRSF19,CARD9
11/373	929,1147,4	CD14,CHUK,MYD88,PSMB7,UBE2D2,UBE2N,VAMP8,BTRC,UBAC1,FZR1,PJA1
5/82	1147,5695,	CHUK,PSMB7,UBE2D2,UBE2N,BTRC
12/440	367,1147,1	AR,CHUK,EDA,MYD88,STK3,UBE2N,BTRC,ZBTB7A,PIAS4,CACTIN,MTDH,MIR26A1
5/85	929,1147,4	CD14,CHUK,MYD88,PSMB7,VAMP8
3/25	1147,5966,	CHUK,REL,POLR1A
6/131	929,1147,4	CD14,CHUK,MYD88,UBE2D2,UBE2N,BTRC
5/92	929,1147,4	CD14,CHUK,MYD88,UBE2N,BTRC
5/93	929,1147,4	CD14,CHUK,MYD88,UBE2N,BTRC
5/93	929,1147,4	CD14,CHUK,MYD88,UBE2N,BTRC
3/27	929,1147,4	CD14,CHUK,MYD88
3/27	929,1147,4	CD14,CHUK,MYD88
7/187	929,1147,1	CD14,CHUK,EDA,PSMB7,REL,BTRC,RC3H2
5/97	929,1147,4	CD14,CHUK,MYD88,UBE2N,BTRC
5/97	929,1147,4	CD14,CHUK,MYD88,UBE2N,BTRC
3/29	929,1147,7	CD14,CHUK,UBE2D2
5/98	1147,5695,	CHUK,PSMB7,UBE2D2,UBE2N,BTRC
5/99	929,1147,7	CD14,CHUK,UBE2D2,UBE2N,BTRC
5/99	929,1147,7	CD14,CHUK,UBE2D2,UBE2N,BTRC
5/100	1147,4615,	CHUK,MYD88,PSMB7,UBE2N,BTRC
5/100	929,1147,4	CD14,CHUK,MYD88,UBE2N,BTRC
5/100	929,1147,4	CD14,CHUK,MYD88,UBE2N,BTRC

5/101	929,1147,4	CD14,CHUK,MYD88,PSMB7,VAMP8
5/103	1147,4615,	CHUK,MYD88,PSMB7,UBE2N,BTRC
3/33	929,1147,7	CD14,CHUK,UBE2D2
9/315	929,1147,1	CD14,CHUK,EDA,IGBP1,PSMB7,ADAMTS13,PIAS4,TNFRSF19,CACTIN
4/67	1147,5695,	CHUK,PSMB7,REL,BTRC
3/34	1147,4615,	CHUK,MYD88,UBE2N
8/266	367,1147,1	AR,CHUK,EDA,MYD88,STK3,UBE2N,ZBTB7A,MTDH
5/113	1147,5063,	CHUK,PAK3,PSMB7,BTRC,CARD9
14/647	929,1147,4	CD14,CHUK,MYD88,PAK3,PSMB7,UBE2D2,UBE2N,BTRC,RC3H2,DDX60,CACTIN,C
3/37	1147,7334,	CHUK,UBE2N,CARD9
5/116	1147,5063,	CHUK,PAK3,PSMB7,BTRC,CARD9
15/731	929,1147,1	CD14,CHUK,CPN1,MYD88,PAK3,PSMB7,UBE2D2,UBE2N,BTRC,RC3H2,DDX60,CAC
8/287	1147,3476,	CHUK,IGBP1,MAP2K2,STK3,UBE2N,BTRC,TNFRSF19,CARD9
14/681	929,1147,4	CD14,CHUK,MYD88,PAK3,PSMB7,UBE2D2,UBE2N,BTRC,RC3H2,DDX60,CACTIN,C
5/127	929,1147,4	CD14,CHUK,MYD88,REL,CACTIN
6/180	1147,4615,	CHUK,MYD88,PSMB7,UBE2N,BTRC,CACTIN
5/129	929,1147,4	CD14,CHUK,MYD88,REL,CACTIN
4/83	1147,5695,	CHUK,PSMB7,REL,BTRC
9/358	929,1147,4	CD14,CHUK,MYD88,NOTCH1,TBXA2R,ADAMTS13,CACTIN,CARD9,MTDH
13/340	166,1613,1	TLE5,DAPK3,EDA,NOTCH1,PSMB7,STK3,TLE2,WNT8B,BTRC,TLE6,TCF7L1,LZTS2,A
13/340	166,1613,1	TLE5,DAPK3,EDA,NOTCH1,PSMB7,STK3,TLE2,WNT8B,BTRC,TLE6,TCF7L1,LZTS2,A
6/80	166,367,10	TLE5,AR,CDX4,VCAN,TLE2,TCF7L1
11/294	166,1613,1	TLE5,DAPK3,EDA,NOTCH1,PSMB7,STK3,TLE2,BTRC,TLE6,LZTS2,AMER2
3/12	166,7089,8	TLE5,TLE2,TCF7L1
12/376	166,1613,1	TLE5,DAPK3,EDA,NOTCH1,PSMB7,STK3,TLE2,BTRC,TLE6,TCF7L1,LZTS2,AMER2
8/185	166,4851,5	TLE5,NOTCH1,PSMB7,STK3,TLE2,TLE6,LZTS2,AMER2
16/631	166,1613,1	TLE5,DAPK3,EDA,GRID2,NOTCH1,PSMB7,SH3GL1,STK3,TLE2,WNT8B,BTRC,PIAS4,
14/525	166,1613,1	TLE5,DAPK3,EDA,NOTCH1,PSMB7,STK3,TLE2,WNT8B,BTRC,PIAS4,TLE6,TCF7L1,L
14/527	166,1613,1	TLE5,DAPK3,EDA,NOTCH1,PSMB7,STK3,TLE2,WNT8B,BTRC,PIAS4,TLE6,TCF7L1,L
5/83	166,5695,7	TLE5,PSMB7,TLE2,BTRC,TCF7L1
8/220	166,4851,5	TLE5,NOTCH1,PSMB7,STK3,TLE2,TLE6,LZTS2,AMER2
3/42	7089,8945,	TLE2,BTRC,TCF7L1
3/10	9355,5124	LHX2,PDZD11,CRB2
3/10	9355,5124	LHX2,PDZD11,CRB2
3/10	9355,5124	LHX2,PDZD11,CRB2
3/17	9355,5124	LHX2,PDZD11,CRB2
3/11	2182,2767,	ACSL4,GNA11,GNA15,S1PR4,TBXA2R,ARR3,AR,HBEGF
3/11	2182,2767,	ACSL4,GNA11,GNA15
3/21	2767,2769,	GNA11,GNA15,S1PR4
3/24	2767,2769,	GNA11,GNA15,TBXA2R
4/57	407,2767,2	ARR3,GNA11,GNA15,TBXA2R
3/33	2767,2769,	GNA11,GNA15,TBXA2R
3/36	367,2767,2	AR,GNA11,GNA15
3/41	1839,2767,	HBEGF,GNA11,GNA15
10/245	28,30,3295	ABO,ACAA1,HSD17B4,PEX13,SOCS2,MTMR6,PIP5K1C,INPP5E,TMEM150A,GLT6D
10/245	28,30,3295	ABO,ACAA1,HSD17B4,PEX13,SOCS2,MTMR6,PIP5K1C,INPP5E,TMEM150A,GLT6D
16/739	30,2182,32	ACAA1,ACSL4,HSD17B4,SCD,MED22,MTMR6,AGPAT2,PIP5K1C,TNFAIP8,HPGDS,I

7/212	9107,1055!	MTMR6,AGPAT2,PIP5K1C,TNFAIP8,INPP5E,AWAT2,DGAT2L6
4/71	5333,9107,	PLCD1,MTMR6,PIP5K1C,INPP5E
10/416	2182,8835,	ACSL4,SOCS2,MTMR6,AGPAT2,PIP5K1C,INSIG2,INPP5E,TMEM150A,AWAT2,DGA
4/84	9107,2339(	MTMR6,PIP5K1C,TNFAIP8,INPP5E
9/222	822,6455,9	CAPG,SH3GL1,MRPL19,MRPL42,VILL,PTCD3,MRPL43,MRPL54,CAMSAP1,EEF2,RF
9/222	822,6455,9	CAPG,SH3GL1,MRPL19,MRPL42,VILL,PTCD3,MRPL43,MRPL54,CAMSAP1
7/136	1938,6156,	EEF2,RPL30,MRPL19,MRPL42,PTCD3,MRPL43,MRPL54
11/340	822,6455,7	CAPG,SH3GL1,WNT8B,VAMP8,MRPL19,MRPL42,VILL,PTCD3,MRPL43,MRPL54,C/
4/48	9801,2897'	MRPL19,MRPL42,MRPL43,MRPL54
5/87	9801,2897'	MRPL19,MRPL42,PTCD3,MRPL43,MRPL54
5/87	9801,2897'	MRPL19,MRPL42,PTCD3,MRPL43,MRPL54
5/87	9801,2897'	MRPL19,MRPL42,PTCD3,MRPL43,MRPL54
5/89	9801,2897'	MRPL19,MRPL42,PTCD3,MRPL43,MRPL54
5/89	9801,2897'	MRPL19,MRPL42,PTCD3,MRPL43,MRPL54
5/93	9801,2897'	MRPL19,MRPL42,PTCD3,MRPL43,MRPL54
5/105	9801,2897'	MRPL19,MRPL42,PTCD3,MRPL43,MRPL54
4/77	9801,2897'	MRPL19,MRPL42,MRPL43,MRPL54
8/291	1938,6130,	EEF2,RPL7A,RPL30,MRPL19,MRPL42,PTCD3,MRPL43,MRPL54
6/103	2803,8673,	GOLGA4,VAMP8,SEC16A,VAMP5,CCDC93,AP1AR,ENTR1
6/103	2803,8673,	GOLGA4,VAMP8,SEC16A,VAMP5,CCDC93,AP1AR
5/95	2803,9919,	GOLGA4,SEC16A,VAMP5,ENTR1,CCDC93
4/61	2803,9919,	GOLGA4,SEC16A,VAMP5,CCDC93
3/41	2803,9919,	GOLGA4,SEC16A,VAMP5
4/39	1839,1938,	HBEGF,EEF2,MAP2K2,ANTXR2,DAPK3
4/39	1839,1938,	HBEGF,EEF2,MAP2K2,ANTXR2
3/41	1613,1839,	DAPK3,HBEGF,MAP2K2
16/680	367,2803,2	AR,GOLGA4,GPR21,MATN2,NOTCH1,STK3,TRPC5,SOCS2,LHX2,PDLIM5,SIRT6,ATF
16/680	367,2803,2	AR,GOLGA4,GPR21,MATN2,NOTCH1,STK3,TRPC5,SOCS2,LHX2,PDLIM5,SIRT6,ATF
8/235	2803,4851,	GOLGA4,NOTCH1,TRPC5,LHX2,SIRT6,BCL11A,SEMA4G,LZTS2
10/350	367,2803,2	AR,GOLGA4,GPR21,NOTCH1,STK3,TRPC5,SOCS2,ATP8A2,BCL11A,SEMA4G
14/658	474,2803,4	ATOH1,GOLGA4,MATN2,NOTCH1,PAK3,PAX2,STK3,TRPC5,LHX2,PDLIM5,PALLD,A
13/598	474,2803,4	ATOH1,GOLGA4,MATN2,NOTCH1,PAK3,PAX2,TRPC5,LHX2,PDLIM5,PALLD,ATP8A
14/672	474,2803,4	ATOH1,GOLGA4,MATN2,NOTCH1,PAK3,PAX2,STK3,TRPC5,LHX2,PDLIM5,PALLD,A
14/676	474,2803,4	ATOH1,GOLGA4,MATN2,NOTCH1,PAK3,PAX2,STK3,TRPC5,LHX2,PDLIM5,PALLD,A
11/478	474,2803,4	ATOH1,GOLGA4,MATN2,NOTCH1,PAK3,PAX2,TRPC5,LHX2,PALLD,ATP8A2,SEMA4
15/756	367,474,28	AR,ATOH1,GOLGA4,MATN2,NOTCH1,PAK3,PAX2,TRPC5,LHX2,PDLIM5,PALLD,ATI
7/236	2803,7224,	GOLGA4,TRPC5,LHX2,PDLIM5,SIRT6,BCL11A,SEMA4G
5/87	367,1147,5	AR,CHUK,MAP2K2,TCF7L1,CREB3L3
5/87	367,1147,5	AR,CHUK,MAP2K2,TCF7L1,CREB3L3
3/25	4851,1001:	NOTCH1,SRA1,NMRK2,HIF1AN
3/25	4851,1001:	NOTCH1,SRA1,NMRK2
4/53	4851,1001:	NOTCH1,SRA1,NMRK2,HIF1AN
16/718	1613,5063,	DAPK3,PAK3,MAP2K2,PSMB7,STK3,CRADD,BTRC,OXSR1,SRA1,CTDSPL,NEK6,FZR:
16/718	1613,5063,	DAPK3,PAK3,MAP2K2,PSMB7,STK3,CRADD,BTRC,OXSR1,SRA1,CTDSPL,NEK6,FZR:
4/56	367,5076,5	AR,PAX2,SIRT6,BCL11A,EDA,NOTCH1,STK3,LHX2,ATOH8,BTRC,LBX1,LZTS2,PURA,
4/56	367,5076,5	AR,PAX2,SIRT6,BCL11A
6/150	1896,4851,	EDA,NOTCH1,PAX2,STK3,LHX2,ATOH8

3/36	367,5076,5	AR,PAX2,SIRT6
9/327	367,4851,5	AR,NOTCH1,PAX2,STK3,BTRC,LHX2,LBX1,SIRT6,LZTS2
11/455	367,4851,5	AR,NOTCH1,PAX2,PURA,BTRC,RIDA,EGFL7,SIRT6,ATOH8,MIR126,MIR26A1
4/59	5194,7322,	PEX13,UBE2D2,UBE2N,RNF181,ACAA1,HSD17B4,NDUFB8,PMPCA,ACSL4
4/59	5194,7322,	PEX13,UBE2D2,UBE2N,RNF181
4/63	30,3295,51	ACAA1,HSD17B4,PEX13,UBE2D2
4/68	30,3295,51	ACAA1,HSD17B4,PEX13,UBE2D2
4/68	30,3295,51	ACAA1,HSD17B4,PEX13,UBE2D2
4/68	30,3295,51	ACAA1,HSD17B4,PEX13,UBE2D2
4/69	30,3295,51	ACAA1,HSD17B4,PEX13,UBE2D2
6/163	30,3295,47	ACAA1,HSD17B4,NDUFB8,PEX13,UBE2D2,PMPCA
4/79	5194,7322,	PEX13,UBE2D2,UBE2N,RNF181
4/81	30,3295,51	ACAA1,HSD17B4,PEX13,UBE2D2
4/83	30,2182,32	ACAA1,ACSL4,HSD17B4,PEX13
3/29	1839,4145,	HBEGF,MATK,NRG2,FGF5,MYD88,PIP5K1C,STK3,MTDH,MIR126
3/29	1839,4145,	HBEGF,MATK,NRG2
5/103	1839,2250,	HBEGF,FGF5,MYD88,NRG2,PIP5K1C
3/32	1839,4145,	HBEGF,MATK,NRG2
5/110	1839,2250,	HBEGF,FGF5,MYD88,NRG2,PIP5K1C
6/177	1839,2250,	HBEGF,FGF5,STK3,NRG2,MTDH,MIR126
3/29	9943,2363(	OXSR1,KCNE5,MIR26A1,KCNS2
3/29	9943,2363(	OXSR1,KCNE5,MIR26A1
4/65	3788,9943,	KCNS2,OXSR1,KCNE5,MIR26A1
3/33	9943,2363(	OXSR1,KCNE5,MIR26A1
3/41	9943,2363(	OXSR1,KCNE5,MIR26A1
4/60	2767,2892,	GNA11,GRIA3,GRID2,MAP2K2
4/60	2767,2892,	GNA11,GRIA3,GRID2,MAP2K2
5/100	367,474,48	AR,ATOH1,NOTCH1,PAX2,LBX1,INSIG2,ATP8A2,PDZD7,ATOH8,CDX4,PLCD1,STK3
5/100	367,474,48	AR,ATOH1,NOTCH1,PAX2,LBX1
5/103	474,5076,5	ATOH1,PAX2,INSIG2,ATP8A2,PDZD7
4/68	474,4851,5	ATOH1,NOTCH1,PAX2,ATOH8
11/445	474,1046,4	ATOH1,CDX4,NOTCH1,PAX2,PLCD1,STK3,LBX1,INSIG2,ATP8A2,PDZD7,CRB2
3/36	474,4851,7	ATOH1,NOTCH1,PDZD7
13/600	367,474,48	AR,ATOH1,NOTCH1,PAX2,STK3,WNT8B,LHX2,LBX1,INSIG2,ATP8A2,PDZD7,ATOH8
5/123	474,5076,5	ATOH1,PAX2,INSIG2,ATP8A2,PDZD7
3/42	474,5076,1	ATOH1,PAX2,LBX1
3/44	474,4851,7	ATOH1,NOTCH1,PDZD7
5/100	2182,6319,	ACSL4,SCD,HPGDS,AWAT1,AWAT2,ACAA1,HSD17B4,VCAN,PEX13,NUP58,ERLIN1
5/100	2182,6319,	ACSL4,SCD,HPGDS,AWAT1,AWAT2
5/109	30,2182,32	ACAA1,ACSL4,HSD17B4,HPGDS,AWAT1
14/651	30,1462,21	ACAA1,VCAN,ACSL4,HSD17B4,PEX13,SCD,NUP58,ERLIN1,HPGDS,INSIG2,PDZD11,
5/115	30,3295,63	ACAA1,HSD17B4,SCD,HPGDS,AWAT1
9/341	1462,3295,	VCAN,HSD17B4,SCD,NUP58,ERLIN1,HPGDS,INSIG2,ZBTB7A,SIRT6
6/172	2182,3295,	ACSL4,HSD17B4,SCD,HPGDS,AWAT1,AWAT2
3/41	2182,3295,	ACSL4,HSD17B4,SCD
6/177	30,2182,32	ACAA1,ACSL4,HSD17B4,SCD,HPGDS,AWAT1
3/31	25885,844	POLR1A,LZTS2,DCLK3

3/31	25885,844	POLR1A,LZTS2,DCLK3

9,ALPK1,MTDH,TIFA,PAK3,PSMB7,UBE2D2,DDX60,VAMP8,CREB3L3,MIR126,VCAN,SEC16A,EEA1,RPL30,A
9,ALPK1,MTDH,TIFA
K1,MTDH,TIFA

LPK1,TIFA
LPK1,TIFA
CARD9,ALPK1,CREB3L3,TIFA,MIR126

LPK1,TIFA

LPK1,TIFA

ARD9,ALPK1,TIFA

CTIN,CARD9,ALPK1,TIFA

ARD9,ALPK1,TIFA

MER2,AR,CDX4,VCAN,GRID2,SH3GL1,PIAS4
MER2

,TLE6,TCF7L1,LZTS2,AMER2
ZTS2,AMER2
ZTS2,AMER2

D1,ACSL4,SCD,MED22,AGPAT2,TNFAIP8,HPGDS,INSIG2,PIAS4,AWAT1,AWAT2,DGAT2L6,PLCD1
D1
NSIG2,PIAS4,INPP5E,AWAT1,AWAT2,DGAT2L6

.T2L6

PL30,WNT8B,VAMP8,RPL7A

AMSAP1

P8A2,BCL11A,RC3H2,SEMA4G,LZTS2,ATOH1,PAK3,PAX2,PALLD,PDZD7,AP1AR
P8A2,BCL11A,RC3H2,SEMA4G,LZTS2

ATP8A2,BCL11A,SEMA4G
A2,SEMA4G,PDZD7
ATP8A2,BCL11A,SEMA4G
ATP8A2,BCL11A,SEMA4G
4G
P8A2,AP1AR,SEMA4G,PDZD7

1,SLF2,FSD1,NACC2,MIR26A1
1,SLF2,FSD1,NACC2,MIR26A1
RIDA,EGFL7,MIR126,MIR26A1

,CRB2,WNT8B,LHX2

3,CRB2

,INSIG2,PDZD11,ZBTB7A,SIRT6

,ZBTB7A,SIRT6,AWAT1

AR,ZBTB7A,TLE5,IGBP1,MAP2K2,STK3,OXSR1,UBAC1,FZR1,PJA1,PIAS4,MIR26A1,POLR1A,RC3H2,ADAMTS

S13,CPN1,NOTCH1,TBXA2R

Supplementary Table s6:The candidate genes in the selection sweep of other cashmere goats and ordinary goats

Gene	Chr	Bin_start	Bin_end	Gene_Start	Gene_end	ZFst	log 2 (θπ ratio)
EPHA6	chr1	39410001	39560001	39786137	40756386	4.174788	1.277423091
TRNAG-CC	chr6	95430001	95580001	95616042	95616114	12.25061	2.255890527
MFSD1	chr1	108680001	108830001	108264279	108290226	4.786555	1.14244988
RARRES1	chr1	108680001	108830001	108362584	108403869	4.786555	1.14244988
GFM1	chr1	108680001	108830001	108407040	108459141	4.786555	1.14244988
LXN	chr1	108680001	108830001	108421618	108429033	4.786555	1.14244988
MLF1	chr1	108680001	108830001	108466500	108497214	4.786555	1.14244988
RSRC1	chr1	108680001	108830001	108525105	108973792	4.786555	1.14244988
SHOX2	chr1	108680001	108830001	108977758	108987010	4.786555	1.14244988
FCRL5	chr3	106900001	107050001	107178895	107253732	8.608819	1.496014054
FCRL4	chr3	106900001	107050001	107264514	107288861	8.608819	1.496014054
FCRL3	chr3	106900001	107050001	107353691	107414661	8.608819	1.496014054
FCRL1	chr3	106900001	107050001	107426215	107442566	8.608819	1.496014054
CCDC71L	chr4	72840001	72990001	72481791	72486442	6.168006	1.338863099
NAMPT	chr4	72840001	72990001	72928492	72971902	6.168006	1.338863099
SYPL1	chr4	72840001	72990001	73117696	73148219	6.168006	1.338863099
CDHR3	chr4	72840001	72990001	73197557	73250292	6.168006	1.338863099
ATXN7L1	chr4	72840001	72990001	73341808	73600324	6.168006	1.338863099
PLEKHG7	chr5	22820001	22970001	22348785	22417547	8.296934	1.22204778
EEA1	chr5	22820001	22970001	22421664	22546190	8.296934	1.22204778
TRNAE-CU	chr5	22820001	22970001	22660968	22661040	8.296934	1.22204778
NUDT4	chr5	22820001	22970001	22964342	22980670	8.296934	1.22204778
UBE2N	chr5	22820001	22970001	22984934	23027066	8.296934	1.22204778
MRPL42	chr5	22820001	22970001	23041060	23070803	8.296934	1.22204778
SOCS2	chr5	22820001	22970001	23129217	23135562	8.296934	1.22204778
CRADD	chr5	22820001	22970001	23232979	23425649	8.296934	1.22204778
ALPK1	chr6	13970001	14120001	13456823	13570751	8.782973	1.80273668
TIFA	chr6	13970001	14120001	13580947	13589288	8.782973	1.80273668
AP1AR	chr6	13970001	14120001	13595155	13624310	8.782973	1.80273668
C6H4orf32	chr6	13970001	14120001	13672177	13715799	8.782973	1.80273668
ANTXR2	chr6	95430001	95580001	95056743	95230556	12.25061	2.255890527
PRDM8	chr6	95430001	95580001	95336875	95356821	12.25061	2.255890527
FGF5	chr6	95430001	95580001	95418992	95440124	12.25061	2.255890527
C6H4orf22	chr6	95430001	95580001	95489192	96199327	12.25061	2.255890527
TRNAG-CC	chr6	95430001	95580001	95616042	95616114	12.25061	2.255890527
IDUA	chr6	117640001	117790001	117245541	117260545	5.777367	1.9243
SLC26A1	chr6	117640001	117790001	117251803	117259744	5.777367	1.9243
DGKQ	chr6	117640001	117790001	117272335	117280603	5.777367	1.9243
CPLX1	chr6	117640001	117790001	117359637	117405415	5.777367	1.9243
PCGF3	chr6	117640001	117790001	117420955	117474202	5.777367	1.9243
MFSD7	chr6	117640001	117790001	117500584	117509668	5.777367	1.9243
ATP5I	chr6	117640001	117790001	117511862	117513678	5.777367	1.9243
PDE6B	chr6	117640001	117790001	117515512	117543217	5.777367	1.9243

PIGG	chr6	117640001	117790001	117580428	117620781	5.777367	1.9243
POC5	chr10	94330001	94480001	94124060	94156199	6.237345	2.273909881
ANKDD1B	chr10	94330001	94480001	94157042	94234857	6.237345	2.273909881
POLK	chr10	94330001	94480001	94240022	94323047	6.237345	2.273909881
COL4A3BP	chr10	94330001	94480001	94322997	94463887	6.237345	2.273909881
HMGCR	chr10	94330001	94480001	94469555	94491458	6.237345	2.273909881
ANKRD31	chr10	94330001	94480001	94562310	94696787	6.237345	2.273909881
GCNT4	chr10	94330001	94480001	94707551	94736446	6.237345	2.273909881
MTDH	chr14	16000001	16150001	15663731	15721499	4.630596	1.435265938
LAPTM4B	chr14	16000001	16150001	15788488	15854656	4.630596	1.435265938
MATN2	chr14	16000001	16150001	15872116	16041603	4.630596	1.435265938
RPL30	chr14	16470001	16620001	16048416	16051780	4.7791	1.435572797
ERICH5	chr14	16490001	16640001	16063489	16086802	5.414622	1.519189002
RIDA	chr14	16490001	16640001	16096003	16104730	5.414622	1.519189002
POP1	chr14	16490001	16640001	16104883	16139688	5.414622	1.519189002
NIPAL2	chr14	16490001	16640001	16173131	16256193	5.414622	1.519189002
KCNS2	chr14	16490001	16640001	16394213	16400041	5.414622	1.519189002
STK3	chr14	16490001	16640001	16433649	16744347	5.414622	1.519189002
OSR2	chr14	16490001	16640001	16881475	16889328	5.414622	1.519189002
VPS13B	chr14	16490001	16640001	16964332	17751028	5.414622	1.519189002
NCAPD3	chr15	1	150001	8858	69851	5.772881	1.176254531
MS4A13	chr15	1	150001	162114	183809	5.772881	1.176254531
MS4A1	chr15	1	150001	236250	251536	5.772881	1.176254531
MS4A2	chr15	1	150001	361337	370421	5.772881	1.176254531
MS4A3	chr15	1	150001	377886	381698	5.772881	1.176254531
OOSP2	chr15	1	150001	389169	394027	5.772881	1.176254531
TCN1	chr15	1	150001	437880	452872	5.772881	1.176254531
GIF	chr15	1	150001	464132	483135	5.772881	1.176254531
MRPL16	chr15	1	150001	496901	501008	5.772881	1.176254531
STX3	chr15	1	150001	501448	547877	5.772881	1.176254531
PLD5	chr16	33580001	33730001	32961659	33377109	4.750542	1.448461405
BECN2	chr16	33580001	33730001	33433023	33434318	4.750542	1.448461405
MAP1LC3C	chr16	33580001	33730001	33461574	33467259	4.750542	1.448461405
EXO1	chr16	33580001	33730001	33479386	33524373	4.750542	1.448461405
WDR64	chr16	33580001	33730001	33534070	33669850	4.750542	1.448461405
CHML	chr16	33580001	33730001	33677364	33686223	4.750542	1.448461405
OPN3	chr16	33580001	33730001	33707072	33715141	4.750542	1.448461405
KMO	chr16	33580001	33730001	33714087	33781937	4.750542	1.448461405
FH	chr16	33580001	33730001	33797989	33822750	4.750542	1.448461405
RGS7	chr16	33580001	33730001	33938680	34410674	4.750542	1.448461405
SLC43A2	chr19	22930001	23080001	22491187	22530698	5.518331	1.190832045
SCARF1	chr19	22930001	23080001	22536624	22548607	5.518331	1.190832045
RILP	chr19	22930001	23080001	22549902	22553181	5.518331	1.190832045
PRPF8	chr19	22930001	23080001	22553692	22584787	5.518331	1.190832045
TLCD2	chr19	22930001	23080001	22602284	22605673	5.518331	1.190832045
MIR22	chr19	22930001	23080001	22609247	22609347	5.518331	1.190832045
WDR81	chr19	22930001	23080001	22617109	22629107	5.518331	1.190832045

SERPINF2	chr19	22930001	23080001	22629867	22637599	5.518331	1.190832045
SERPINF1	chr19	22930001	23080001	22644651	22656868	5.518331	1.190832045
SMYD4	chr19	22930001	23080001	22657588	22696454	5.518331	1.190832045
RPA1	chr19	22930001	23080001	22696549	22740986	5.518331	1.190832045
RTN4RL1	chr19	22930001	23080001	22769076	22848363	5.518331	1.190832045
DPH1	chr19	22930001	23080001	22852077	22862418	5.518331	1.190832045
OVCA2	chr19	22930001	23080001	22860860	22862418	5.518331	1.190832045
HIC1	chr19	22930001	23080001	22871476	22876247	5.518331	1.190832045
SMG6	chr19	22930001	23080001	22876399	23077179	5.518331	1.190832045
SRR	chr19	22930001	23080001	23077186	23091990	5.518331	1.190832045
TSR1	chr19	22930001	23080001	23090732	23101110	5.518331	1.190832045
SGSM2	chr19	22930001	23080001	23102223	23140042	5.518331	1.190832045
TRNAG-CCC	chr6	95430001	95580001	95616042	95616114	12.25061	2.255890527
MNT	chr19	22930001	23080001	23146013	23162285	5.518331	1.190832045
METTL16	chr19	22930001	23080001	23172365	23237617	5.518331	1.190832045
PAFAH1B1	chr19	22930001	23080001	23302330	23368013	5.518331	1.190832045
CLUH	chr19	22930001	23080001	23373646	23395027	5.518331	1.190832045
RAP1GAP2	chr19	22930001	23080001	23437609	23652991	5.518331	1.190832045
SETD5	chr22	17450001	17600001	16950600	17030658	5.127566	1.810611887
THUMPD3	chr22	17460001	17610001	17048771	17070472	5.382906	1.849405564
SRGAP3	chr22	17460001	17610001	17156766	17404110	5.382906	1.849405564
RAD18	chr22	17460001	17610001	17414359	17523990	5.382906	1.849405564
OXTR	chr22	17460001	17610001	17594776	17607958	5.382906	1.849405564
CAV3	chr22	17460001	17610001	17610114	17622648	5.382906	1.849405564
SSUH2	chr22	17460001	17610001	17670175	17692990	5.382906	1.849405564
LMCD1	chr22	17460001	17610001	17740028	17796768	5.382906	1.849405564
CEP192	chr24	44000001	44150001	43556637	43630586	4.659627	1.688602001
LDLRAD4	chr24	44000001	44150001	43647271	43808160	4.659627	1.688602001
FAM210A	chr24	44000001	44150001	43802788	43825139	4.659627	1.688602001
RNMT	chr24	44000001	44150001	43825214	43852257	4.659627	1.688602001
MC5R	chr24	44000001	44150001	43868653	43873520	4.659627	1.688602001
MC2R	chr24	44000001	44150001	43894659	43910455	4.659627	1.688602001
SETBP1	chr24	44020001	44170001	44577303	44983204	4.595879	1.707368914
DUSP8	chr29	51330001	51480001	50962017	50977940	5.511603	2.942207197
MOB2	chr29	51330001	51480001	50987206	51039264	5.511603	2.942207197
BRSK2	chr29	51330001	51480001	51047154	51097037	5.511603	2.942207197
TOLLIP	chr29	51330001	51480001	51162053	51177156	5.511603	2.942207197
MUC5AC	chr29	51330001	51480001	51246526	51276626	5.511603	2.942207197
MUC2	chr29	51330001	51480001	51317872	51331926	5.511603	2.942207197
EDA	chrX1	12290001	12440001	11557022	11895599	4.182243	1.523792496
CDX4	chrX1	12290001	12440001	12054961	12063592	4.182243	1.523792496
FAM155B	chrX1	12290001	12440001	12074820	12103927	4.182243	1.523792496
PJA1	chrX1	12290001	12440001	12110252	12115011	4.182243	1.523792496
STARD8	chrX1	12290001	12440001	12400560	12415129	4.182243	1.523792496
YIPF6	chrX1	12290001	12440001	12585345	12617792	4.182243	1.523792496
OPHN1	chrX1	12290001	12440001	12692459	13271064	4.182243	1.523792496
AR	chrX1	13840001	13990001	13729623	13934065	6.234787	3.444346479

MAGED1	chrX1	14400001	14550001	14646763	14730609	4.432149	2.270950898
TRNAW-CCA	chrX1	14400001	14550001	14663110	14663181	4.432149	2.270950898
GSPT2	chrX1	14400001	14550001	14796387	14798923	4.432149	2.270950898
EDA2R	chrX1	18150001	18300001	18412588	18508471	4.786207	2.591552102

**Supplementary Table s7:The candidate genes in the selection sweep of white cashm
and brown cashmere goats(CDB)**

Gene	Chr	Bin_start	Bin_end	Gene_Start	Gene_end
TIAM1	chr1	3140001	3290001	2425188	2887657
KRTAP11-1	chr1	3140001	3290001	3134832	3135697
KAP8	chr1	3140001	3290001	3215311	3215883
TMPRSS15	chr1	17450001	17600001	17151749	17305379
CHODL	chr1	17450001	17600001	17307773	17331943
C1H21orf91	chr1	17450001	17600001	17836541	17868814
BTG3	chr1	17460001	17610001	18029148	18048638
TRNAK-UUU	chr1	17480001	17630001	18036604	18036676
CXADR	chr1	17480001	17630001	18049029	18115289
CLDND1	chr1	42740001	42890001	42586817	42594123
GPR15	chr1	42740001	42890001	42603291	42606012
CPOX	chr1	42740001	42890001	42654291	42669722
ST3GAL6	chr1	42740001	42890001	42800705	42887578
DCBLD2	chr1	42740001	42890001	42886443	42968414
TRNAE-UUC	chrX1	46010001	46160001	46250912	46250984
BCHE	chr1	101340001	101490001	101031428	101110937
SLITRK3	chr1	101340001	101490001	101714521	101725766
SI	chr1	101340001	101490001	101811454	101920451
TRNAG-CCC	chrX2	10370001	10520001	10239324	10239396
MFSD1	chr1	108580001	108730001	108264279	108290226
RARRES1	chr1	108580001	108730001	108362584	108403869
GFM1	chr1	108580001	108730001	108407040	108459141
LXN	chr1	108580001	108730001	108421618	108429033
MLF1	chr1	108580001	108730001	108466500	108497214
RSRC1	chr1	108580001	108730001	108525105	108973792
SHOX2	chr1	108580001	108730001	108977758	108987010
RAP2B	chr1	114630001	114780001	114205077	114214900
P2RY1	chr1	114690001	114840001	114567590	114574104
MBNL1	chr1	114690001	114840001	114974326	115192808
SUCNR1	chr1	115610001	115760001	115626920	115636639
C1H3orf58	chr1	124370001	124520001	124443207	124467469
SLC9A9	chr1	124370001	124520001	124629179	125280384
TRNAK-UUU	chr1	17480001	17630001	18036604	18036676
FOXL2	chr1	130100001	130250001	129771906	129773370
PIK3CB	chr1	130100001	130250001	129881817	130060986
FAIM	chr1	130100001	130250001	130073736	130089218
CEP70	chr1	130100001	130250001	130089234	130186513
ESYT3	chr1	130100001	130250001	130189979	130245320
MRAS	chr1	130100001	130250001	130284683	130352520
NME9	chr1	130100001	130250001	130372691	130395986
ARMC8	chr1	130100001	130250001	130401143	130507389
DBR1	chr1	130100001	130250001	130521468	130533958
A4GNT	chr1	130100001	130250001	130549744	130585303

DZIP1L	chr1	130100001	130250001	130589968	130649459
TRNAW-CCA	chrX1	14400001	14550001	14663110	14663181
CPNE4	chr1	138000001	138150001	137317163	137966558
MRPL3	chr1	138000001	138150001	138002275	138059429
NUDT16	chr1	138000001	138150001	138166503	138168104
NEK11	chr1	138000001	138150001	138180436	138467344
ASTE1	chr1	138000001	138150001	138467431	138489609
ATP2C1	chr1	138000001	138150001	138497975	138651086
ARMC9	chr2	17120001	17270001	16579176	16761996
PSMD1	chr2	17120001	17270001	16782175	16865843
HTR2B	chr2	17120001	17270001	16821118	16836903
C2H2orf72	chr2	17120001	17270001	16874291	16884102
TRNAW-CCA	chrX1	14400001	14550001	14663110	14663181
GPR55	chr2	17120001	17270001	17012406	17013648
ITM2C	chr2	17120001	17270001	17046174	17060341
CAB39	chr2	17120001	17270001	17092152	17190120
SP140	chr2	17120001	17270001	17416819	17496056
TRNAS-GGA	chr4	52950001	53100001	53090579	53090650
SP110	chr2	17120001	17270001	17496060	17548527
SLC16A14	chr2	17120001	17270001	17618698	17652976
TRIP12	chr2	17190001	17340001	17763215	17916591
RAB3GAP1	chr2	74440001	74590001	73996763	74121959
ZRANB3	chr2	74440001	74590001	74145804	74436792
R3HDM1	chr2	74440001	74590001	74437490	74600543
MIR128	chr2	74440001	74590001	74560517	74560648
UBXN4	chr2	74440001	74590001	74619014	74648704
LCT	chr2	74440001	74590001	74649213	74699787
MCM6	chr2	74440001	74590001	74708434	74745046
DARS	chr2	74440001	74590001	74780841	74845981
CXCR4	chr2	74440001	74590001	74976699	74980389
TRNAT-AGU	chr2	75320001	75470001	75422127	75422200
TRNAC-GCA	chr16	62530001	62680001	62935749	62935820
THSD7B	chr2	75320001	75470001	75653642	76691998
GALNT5	chr2	97410001	97560001	97082918	97124602
ERMN	chr2	97410001	97560001	97130255	97137921
CYTIP	chr2	97410001	97560001	97216183	97246728
ACVR1C	chr2	97410001	97560001	97335789	97391095
ACVR1	chr2	97410001	97560001	97581866	97719746
UPP2	chr2	97410001	97560001	97918654	97959126
CCDC148	chr2	97420001	97570001	97989039	98211940
BAZ2B	chr2	99810001	99960001	99134862	99464817
7-Mar	chr2	99810001	99960001	99549258	99596850
PLA2R1	chr2	99810001	99960001	99763166	99888468
ITGB6	chr2	99810001	99960001	99910920	100053272
RBMS1	chr2	99810001	99960001	100074882	100296249
ARL4C	chr3	6780001	6930001	6672190	6676211
SPP2	chr3	6780001	6930001	7052998	7080042

TRPM8	chr3	6780001	6930001	7101606	7208432
HJURP	chr3	6780001	6930001	7253797	7266899
MROH2A	chr3	6780001	6930001	7287456	7345980
ZC3H12A	chr3	12760001	12910001	12397169	12407546
MEAF6	chr3	12760001	12910001	12415872	12440541
SNIP1	chr3	12760001	12910001	12453766	12469774
DNALI1	chr3	12760001	12910001	12470869	12479526
GNL2	chr3	12760001	12910001	12479488	12503646
RSPO1	chr3	12760001	12910001	12519202	12541600
C3H1orf109	chr3	12760001	12910001	12591206	12598018
CDCA8	chr3	12760001	12910001	12598174	12613416
EPHA10	chr3	12760001	12910001	12615370	12659007
MANEAL	chr3	12760001	12910001	12685596	12694130
YRDC	chr3	12760001	12910001	12693862	12697616
MTF1	chr3	12760001	12910001	12698723	12744794
INPP5B	chr3	12760001	12910001	12745894	12797875
SF3A3	chr3	12760001	12910001	12807257	12831210
FHL3	chr3	12760001	12910001	12835146	12842774
UTP11	chr3	12760001	12910001	12849439	12860707
POU3F1	chr3	12760001	12910001	12879313	12881446
RLF	chr3	15250001	15400001	14836369	14922369
TMCO2	chr3	15250001	15400001	14929793	14932608
ZMPSTE24	chr3	15250001	15400001	14950764	14993348
COL9A2	chr3	15250001	15400001	15000986	15016479
SMAP2	chr3	15250001	15400001	15053632	15102158
ZFP69B	chr3	15250001	15400001	15120282	15144459
ZFP69	chr3	15250001	15400001	15148977	15165365
RIMS3	chr3	15250001	15400001	15250367	15295283
NFYC	chr3	15250001	15400001	15315404	15381493
MIR30E	chr3	15250001	15400001	15365532	15365637
MIR30C	chr3	15250001	15400001	15368653	15368757
KCNQ4	chr3	15250001	15400001	15393310	15452416
CITED4	chr3	15250001	15400001	15475017	15476378
CTPS1	chr3	15250001	15400001	15594936	15625865
SLFNL1	chr3	15250001	15400001	15628164	15633250
SCMH1	chr3	15250001	15400001	15637457	15855355
FOXO6	chr3	15540001	15690001	15982854	16004565
EDN2	chr3	15540001	15690001	16110938	16116874
HIVEP3	chr3	15570001	15720001	16143582	16659535
GUCA2B	chr3	17120001	17270001	16750513	16753710
GUCA2A	chr3	17120001	17270001	16757271	16760306
FOXJ3	chr3	17120001	17270001	16769526	16920659
RIMKLA	chr3	17120001	17270001	16994111	17027435
ZMYND12	chr3	17120001	17270001	17048117	17083139
PPCS	chr3	17120001	17270001	17083553	17224415
PPIH	chr3	17120001	17270001	17227464	17244672
YBX1	chr3	17120001	17270001	17249139	17268236

CLDN19	chr3	17120001	17270001	17281990	17287819
P3H1	chr3	17120001	17270001	17294547	17313795
C3H1orf50	chr3	17120001	17270001	17313998	17323506
SVBP	chr3	17120001	17270001	17346323	17352490
ERMAP	chr3	17120001	17270001	17372886	17384417
ZNF691	chr3	17120001	17270001	17386195	17391582
SLC2A1	chr3	17120001	17270001	17438803	17472481
OLFM3	chr3	79040001	79190001	79015350	79240851
COL11A1	chr3	79980001	80130001	79996781	80226730
RNPC3	chr3	80100001	80250001	80656949	80688529
VIPR2	chr4	330001	480001	171628	239456
WDR60	chr4	330001	480001	293177	346902
ESYT2	chr4	330001	480001	366433	449121
NCAPG2	chr4	330001	480001	470768	542701
PTPRN2	chr4	1470001	1620001	621406	1167657
DNAJB6	chr4	1470001	1620001	1221408	1266789
UBE3C	chr4	1470001	1620001	1312658	1426090
MNX1	chr4	1470001	1620001	1503421	1508316
NOM1	chr4	1470001	1620001	1526709	1539384
LMBR1	chr4	1470001	1620001	1584560	1720496
RNF32	chr4	1470001	1620001	1734604	1770786
SHH	chr4	2630001	2780001	2388104	2398833
RBM33	chr4	2630001	2780001	2421360	2530454
CNPY1	chr4	2630001	2780001	2618556	2628346
EN2	chr4	2630001	2780001	2659430	2666542
INSIG1	chr4	2630001	2780001	2747017	2757803
HTR5A	chr4	2630001	2780001	2878884	2895668
PAXIP1	chr4	2630001	2780001	2922811	2963751
DPP6	chr4	2630001	2780001	2979751	3954235
MIR148A	chr4	50470001	50620001	50269709	50269793
NFE2L3	chr4	50890001	51040001	50434030	50467648
HNRNPA2B1	chr4	50890001	51040001	50469472	50479267
CBX3	chr4	50890001	51040001	50480214	50491070
SNX10	chr4	50890001	51040001	50549549	50616271
SKAP2	chr4	50890001	51040001	50900784	51072547
HOXA1	chr4	50890001	51040001	51262987	51265727
HOXA2	chr4	50890001	51040001	51270010	51272952
HOXA3	chr4	50890001	51040001	51276661	51283467
HOXA4	chr4	50890001	51040001	51297397	51299705
HOXA5	chr4	50890001	51040001	51309920	51312896
HOXA6	chr4	50890001	51040001	51314374	51319005
HOXA7	chr4	50890001	51040001	51323653	51329798
HOXA9	chr4	50890001	51040001	51331211	51337701
MIR196B	chr4	50890001	51040001	51338288	51338372
HOXA10	chr4	50890001	51040001	51339407	51343166
HOXA11	chr4	50890001	51040001	51350542	51354278
HOXA13	chr4	50890001	51040001	51366007	51369167

EVX1	chr4	50890001	51040001	51410813	51414731
HIBADH	chr4	51990001	52140001	51633820	51746218
TAX1BP1	chr4	51990001	52140001	51817207	51906819
JAZF1	chr4	51990001	52140001	51907478	52239310
CREB5	chr4	52950001	53100001	52482298	52922180
TRIL	chr4	52950001	53100001	53029117	53034303
CPVL	chr4	52950001	53100001	53082853	53188759
TRNAS-GGA	chr4	52950001	53100001	53090579	53090650
CHN2	chr4	53650001	53800001	53262143	53602288
PRR15	chr4	53650001	53800001	53620856	53624213
WIPF3	chr4	53650001	53800001	53725612	53809373
SCRN1	chr4	53650001	53800001	53813414	53877817
FKBP14	chr4	53650001	53800001	53892767	53904759
PLEKHA8	chr4	53650001	53800001	53907086	53976470
MTURN	chr4	53650001	53800001	54025518	54060461
ZNRF2	chr4	53650001	53800001	54186584	54278606
TRNAC-ACA	chr16	62530001	62680001	62246657	62246728
HGF	chr4	81210001	81360001	81306673	81397853
CACNA2D1	chr4	81210001	81360001	81665818	82201273
LRRIQ1	chr5	15070001	15220001	14530561	14741764
ALX1	chr5	15070001	15220001	14780433	14802313
RASSF9	chr5	15070001	15220001	15329017	15361750
NTS	chr5	15070001	15220001	15391221	15402965
MGAT4C	chr5	15070001	15220001	15533616	15603737
LRIG3	chr5	53650001	53800001	53854406	53905944
ALPK1	chr6	13960001	14110001	13456823	13570751
TIFA	chr6	13960001	14110001	13580947	13589288
AP1AR	chr6	13960001	14110001	13595155	13624310
C6H4orf32	chr6	13960001	14110001	13672177	13715799
LEF1	chr6	17940001	18090001	17398506	17521097
HADH	chr6	17980001	18130001	17529001	17573045
SGMS2	chr6	18010001	18160001	17613593	17710656
PAPSS1	chr6	18010001	18160001	17802392	17916090
DKK2	chr6	18010001	18160001	18461852	18590125
NPNT	chr6	20000001	20150001	19517104	19594712
GSTCD	chr6	20040001	20190001	19640852	19780384
INTS12	chr6	20040001	20190001	19780400	19801936
ARHGEF38	chr6	20040001	20190001	19807399	19954771
PPA2	chr6	20040001	20190001	20029174	20144590
TET2	chr6	20040001	20190001	20216813	20352863
PPARGC1A	chr6	43930001	44080001	43798769	43919972
DHX15	chr6	43990001	44140001	44553729	44609199
TRNAW-CCA	chrX1	14400001	14550001	14663110	14663181
APBB2	chr6	60980001	61130001	60285849	60665198
UCHL1	chr6	60980001	61130001	60715873	60727733
LIMCH1	chr6	60980001	61130001	60794267	61154327
PHOX2B	chr6	60980001	61130001	61216690	61220345

TMEM33	chr6	60980001	61130001	61387898	61407481
SLC30A9	chr6	60980001	61130001	61429091	61509809
BEND4	chr6	60980001	61130001	61544757	61574269
GABRG1	chr6	65110001	65260001	65127653	65214852
GABRA2	chr6	65110001	65260001	65342451	65518411
TRNAW-CCA	chrX1	14400001	14550001	14663110	14663181
TRNAY-GUA	chr6	79390001	79540001	79799916	79799987
EPHA5	chr6	81310001	81460001	81441793	81853316
ANKRD17	chr6	88780001	88930001	88712562	88880682
ALB	chr6	88780001	88930001	89001769	89019686
AFP	chr6	88780001	88930001	89027050	89048856
AFM	chr6	88780001	88930001	89058533	89081033
RASSF6	chr6	88780001	88930001	89142702	89210900
CXCL8	chr6	88780001	88930001	89335401	89338881
ANTXR2	chr6	95410001	95560001	95056743	95230556
PRDM8	chr6	95410001	95560001	95336875	95356821
FGF5	chr6	95410001	95560001	95418992	95440124
C6H4orf22	chr6	95410001	95560001	95489192	96199327
TRNAG-CCC	chrX2	10370001	10520001	10239324	10239396
CAST	chr7	14990001	15140001	14434087	14568155
TRNAW-CCA	chrX1	14400001	14550001	14663110	14663181
PCSK1	chr7	15000001	15150001	14805715	14858039
ELL2	chr7	15000001	15150001	15292347	15367401
GLRX	chr7	15000001	15150001	15436081	15445521
RHOBTB3	chr7	15000001	15150001	15465965	15527868
SPATA9	chr7	15000001	15150001	15554156	15584327
RFESD	chr7	15000001	15150001	15559116	15595714
TRNAC-GCA	chr16	62530001	62680001	62935749	62935820
HAND1	chr7	44960001	45110001	44621033	44624336
SAP30L	chr7	44960001	45110001	44638848	44652660
GALNT10	chr7	44960001	45110001	44665723	44897352
MFAP3	chr7	44960001	45110001	45031201	45048036
FAM114A2	chr7	44960001	45110001	45048066	45084637
GRIA1	chr7	44960001	45110001	45259914	45615155
ARAP3	chr7	58160001	58310001	57741258	57769308
FCHSD1	chr7	58160001	58310001	57771763	57784020
RELL2	chr7	58160001	58310001	57782218	57786366
HDAC3	chr7	58160001	58310001	57786492	57799552
DIAPH1	chr7	58160001	58310001	57801641	57918084
TAF7	chr7	58160001	58310001	58114795	58117019
PCDHB14	chr7	58160001	58310001	58185242	58189234
PCDHB16	chr7	58160001	58310001	58214667	58217524
PCDHB7	chr7	58160001	58310001	58225893	58228883
PCDHB1	chr7	58160001	58310001	58350170	58352697
TRNAC-GCA	chr16	62530001	62680001	62935749	62935820
ZMAT2	chr7	58160001	58310001	58668572	58674067
HARS2	chr7	58160001	58310001	58674889	58682059

HARS	chr7	58160001	58310001	58682131	58695144
DND1	chr7	58160001	58310001	58695237	58698549
WDR55	chr7	58160001	58310001	58698546	58703385
IK	chr7	58160001	58310001	58705364	58718109
NDUFA2	chr7	58160001	58310001	58718219	58720374
TMCO6	chr7	58160001	58310001	58720332	58725924
CD14	chr7	58160001	58310001	58731680	58735123
SLC35A4	chr7	58220001	58370001	58754826	58759575
APBB3	chr7	58220001	58370001	58759676	58766097
SRA1	chr7	58220001	58370001	58766299	58773099
EIF4EBP3	chr7	58220001	58370001	58773687	58774464
CXXC5	chr7	60010001	60160001	59617412	59651710
UBE2D2	chr7	60010001	60160001	59671314	59714771
TMEM173	chr7	60010001	60160001	59759510	59765656
ECSCR	chr7	60010001	60160001	59772140	59780273
DNAJC18	chr7	60010001	60160001	59788431	59817336
SPATA24	chr7	60010001	60160001	59822027	59827252
PROB1	chr7	60010001	60160001	59828342	59833722
MZB1	chr7	60010001	60160001	59834329	59836445
SLC23A1	chr7	60010001	60160001	59837143	59862223
PAIP2	chr7	60010001	60160001	59849786	59867040
MATR3	chr7	60010001	60160001	59873045	59911585
SIL1	chr7	60010001	60160001	59972174	60233205
CTNNA1	chr7	60010001	60160001	60240857	60414279
LRRTM2	chr7	60010001	60160001	60304287	60307637
TRNAS-GGA	chr4	52950001	53100001	53090579	53090650
TRNAC-GCA	chr16	62530001	62680001	62935749	62935820
HSPA9	chr7	60010001	60160001	60577216	60593487
PDLIM4	chr7	88850001	89000001	88413050	88427472
SLC22A4	chr7	88860001	89010001	88444408	88487123
SLC22A5	chr7	88860001	89010001	88511443	88537601
IRF1	chr7	88860001	89010001	88630486	88637730
IL5	chr7	88860001	89010001	88684078	88686626
RAD50	chr7	88860001	89010001	88702532	88815957
TRNAC-GCA	chr16	62530001	62680001	62935749	62935820
IL13	chr7	88860001	89010001	88859199	88862094
IL4	chr7	88860001	89010001	88878436	88886254
KIF3A	chr7	88860001	89010001	88891761	88973815
8-Sep	chr7	88860001	89010001	88981804	89009199
MKNK2	chr7	88860001	89010001	89073549	89085155
MOB3A	chr7	88860001	89010001	89102714	89121739
IZUMO4	chr7	88860001	89010001	89121955	89125128
AP3D1	chr7	88860001	89010001	89126591	89159963
JSRP1	chr7	88860001	89010001	89165304	89169175
AMH	chr7	88860001	89010001	89169334	89172804
SF3A2	chr7	88860001	89010001	89172888	89181602
PLEKHJ1	chr7	88860001	89010001	89181731	89184374

DOT1L	chr7	88860001	89010001	89184931	89236978
TRNAG-CCC	chrX2	10370001	10520001	10239324	10239396
OAZ1	chr7	88860001	89010001	89278872	89282376
C7H19orf35	chr7	88860001	89010001	89283752	89289772
LINGO3	chr7	88860001	89010001	89295581	89311797
LSM7	chr7	88860001	89010001	89322318	89326916
SPPL2B	chr7	88860001	89010001	89327012	89341777
TMPRSS9	chr7	88860001	89010001	89368992	89400432
TIMM13	chr7	88860001	89010001	89408589	89409818
LMNB2	chr7	88860001	89010001	89410232	89430556
RIC1	chr8	39340001	39490001	38776219	38919131
PDCD1LG2	chr8	39350001	39500001	38979921	39046980
CD274	chr8	39350001	39500001	39077983	39095141
PLGRKT	chr8	39350001	39500001	39111116	39155487
INSL6	chr8	39350001	39500001	39210866	39227365
JAK2	chr8	39350001	39500001	39245187	39360411
RCL1	chr8	39350001	39500001	39520371	39583801
AK3	chr8	39350001	39500001	39635494	39656272
CDC37L1	chr8	39350001	39500001	39660101	39678648
PLPP6	chr8	39350001	39500001	39700133	39703413
SPATA6L	chr8	39350001	39500001	39734093	39756809
SLC1A1	chr8	39350001	39500001	39770036	39845247
DNAI1	chr8	76080001	76230001	75600294	75669575
ENHO	chr8	76080001	76230001	75669638	75671745
CNTFR	chr8	76080001	76230001	75695438	75782519
RPP25L	chr8	76080001	76230001	75801233	75803474
DCTN3	chr8	76080001	76230001	75804882	75812788
ARID3C	chr8	76080001	76230001	75814303	75821142
SIGMAR1	chr8	76080001	76230001	75828160	75830978
GALT	chr8	76080001	76230001	75837855	75841221
IL11RA	chr8	76080001	76230001	75843232	75852734
CCL27	chr8	76080001	76230001	75852804	75854562
CCL19	chr8	76080001	76230001	75873840	75875825
CCL21	chr8	76080001	76230001	75895977	75897160
RASEF	chr8	76080001	76230001	76002648	76091226
FRMD3	chr8	76080001	76230001	76202142	76554906
TRNAC-ACA	chr16	62530001	62680001	62246657	62246728
IDNK	chr8	76080001	76230001	76615850	76633688
UBQLN1	chr8	76730001	76880001	76659014	76713744
GKAP1	chr8	76730001	76880001	76763253	76861623
KIF27	chr8	76730001	76880001	76879454	76969689
C8H9orf64	chr8	76730001	76880001	76983174	76995044
TRNAC-GCA	chr16	62530001	62680001	62935749	62935820
HNRNPK	chr8	76730001	76880001	77011076	77023300
MIR7	chr8	76730001	76880001	77012728	77012874
RMI1	chr8	76730001	76880001	77023315	77038142
SLC28A3	chr8	76730001	76880001	77279655	77345734

GRIK2	chr9	36000001	36150001	35137287	35864369
ASCC3	chr9	36000001	36150001	36344650	36689896
PTPRK	chr9	53900001	54050001	53315015	53933095
LAMA2	chr9	53900001	54050001	54306124	54983404
AKAP7	chr9	56990001	57140001	56487185	56612712
ARG1	chr9	56990001	57140001	56815375	56830671
MED23	chr9	56990001	57140001	56831657	56873993
ENPP3	chr9	56990001	57140001	56897818	56987489
ENPP1	chr9	56990001	57140001	57045830	57117809
CTGF	chr9	56990001	57140001	57175950	57179282
MOXD1	chr9	56990001	57140001	57534766	57632301
TRNAC-ACA	chr16	62530001	62680001	62246657	62246728
SPTB	chr10	26650001	26800001	26106676	26234517
PLEKHG3	chr10	26680001	26830001	26235988	26281149
PPP1R36	chr10	26680001	26830001	26407387	26430457
HSPA2	chr10	26680001	26830001	26436107	26438017
ZBTB1	chr10	26680001	26830001	26442455	26467963
ZBTB25	chr10	26680001	26830001	26467957	26514294
AKAP5	chr10	26680001	26830001	26500237	26505331
MTHFD1	chr10	26680001	26830001	26510059	26572160
ESR2	chr10	26680001	26830001	26669088	26714330
SYNE2	chr10	26680001	26830001	26722712	27046703
TRNAW-CCA	chrX1	14400001	14550001	14663110	14663181
SGPP1	chr10	26680001	26830001	27134837	27177851
WDR89	chr10	26680001	26830001	27228084	27276570
ZFHX2	chr10	80470001	80620001	80016831	80045551
THTPA	chr10	80470001	80620001	80048227	80052486
AP1G2	chr10	80480001	80630001	80051423	80059940
JPH4	chr10	80490001	80640001	80060027	80070268
CARMIL3	chr10	80560001	80710001	80346563	80364380
CPNE6	chr10	80560001	80710001	80365429	80372592
NRL	chr10	80560001	80710001	80374515	80388480
PCK2	chr10	80560001	80710001	80388956	80397146
DCAF11	chr10	80560001	80710001	80404031	80412030
FITM1	chr10	80560001	80710001	80413784	80416061
PSME1	chr10	80560001	80710001	80418160	80420956
EMC9	chr10	80560001	80710001	80420953	80423346
PSME2	chr10	80560001	80710001	80425928	80429833
RNF31	chr10	80560001	80710001	80429936	80440984
IRF9	chr10	80560001	80710001	80441154	80446438
REC8	chr10	80560001	80710001	80450920	80456724
IPO4	chr10	80560001	80710001	80456694	80465666
TM9SF1	chr10	80560001	80710001	80465618	80471145
TSSK4	chr10	80560001	80710001	80471219	80481243
CHMP4A	chr10	80560001	80710001	80481346	80485099
NEDD8	chr10	80560001	80710001	80487701	80496437
GMPR2	chr10	80560001	80710001	80496352	80502962

TINF2	chr10	80560001	80710001	80500910	80506489
TGM1	chr10	80560001	80710001	80514652	80529096
RABGGTA	chr10	80560001	80710001	80531344	80537374
DHRS1	chr10	80560001	80710001	80553019	80561411
NOP9	chr10	80560001	80710001	80561504	80567378
CIDEB	chr10	80560001	80710001	80567352	80573419
LTB4R2	chr10	80560001	80710001	80569575	80576366
LTB4R	chr10	80560001	80710001	80572744	80580288
ADCY4	chr10	80560001	80710001	80579805	80595382
RIPK3	chr10	80560001	80710001	80596354	80600577
NFATC4	chr10	80560001	80710001	80625155	80634456
NYNRIN	chr10	80560001	80710001	80658795	80680140
CBLN3	chr10	80560001	80710001	80681703	80691147
KHNYN	chr10	80560001	80710001	80683758	80701406
SDR39U1	chr10	80560001	80710001	80701422	80704767
TBC1D21	chr10	80560001	80710001	80730793	80744577
C10H15orf59	chr10	80560001	80710001	80874053	80887118
CD276	chr10	80560001	80710001	80911808	80946803
NPTN	chr10	80560001	80710001	80995331	81061660
REC114	chr10	80560001	80710001	81061570	81170567
ARIH1	chr10	82460001	82610001	81939607	82041082
HEXA	chr10	82460001	82610001	82112311	82143840
CELF6	chr10	82460001	82610001	82164246	82194306
PARP6	chr10	82460001	82610001	82205875	82232562
PKM	chr10	82460001	82610001	82238790	82265545
GRAMD2	chr10	82460001	82610001	82266642	82301212
SENP8	chr10	82460001	82610001	82315289	82335360
MYO9A	chr10	82460001	82610001	82335575	82577050
NR2E3	chr10	82460001	82610001	82590036	82594865
THSD4	chr10	82460001	82610001	82620551	83285979
SV2C	chr10	94290001	94440001	93649456	93866174
POC5	chr10	94340001	94490001	94124060	94156199
ANKDD1B	chr10	94340001	94490001	94157042	94234857
POLK	chr10	94340001	94490001	94240022	94323047
COL4A3BP	chr10	94340001	94490001	94322997	94463887
HMGCR	chr10	94340001	94490001	94469555	94491458
ANKRD31	chr10	94340001	94490001	94562310	94696787
GCNT4	chr10	94340001	94490001	94707551	94736446
MEMO1	chr11	14920001	15070001	14404200	14515722
DPY30	chr11	14920001	15070001	14524697	14538756
SPAST	chr11	14920001	15070001	14556017	14608600
SLC30A6	chr11	14920001	15070001	14620539	14666163
NLRC4	chr11	14920001	15070001	14669557	14716302
YIPF4	chr11	14920001	15070001	14738946	14782522
BIRC6	chr11	14920001	15070001	14814336	15024500
TTC27	chr11	14920001	15070001	15041424	15219539
LTBP1	chr11	14920001	15070001	15324388	15780520

CRIM1	chr11	18120001	18270001	18670767	18881417
CRIPT	chr11	29080001	29230001	28648447	28659797
SOCS5	chr11	29090001	29240001	28714825	28802754
MCFD2	chr11	29090001	29240001	28943551	28953070
TTC7A	chr11	29090001	29240001	28986941	29103619
TRNAE-UUC	chrX1	46010001	46160001	46250912	46250984
C11H2orf61	chr11	29090001	29240001	29117484	29152416
CALM2	chr11	29090001	29240001	29156107	29169684
EPCAM	chr11	29090001	29240001	29347274	29359525
MSH2	chr11	29090001	29240001	29370644	29455779
KCNK12	chr11	29090001	29240001	29483068	29533843
PLB1	chr11	71250001	71400001	70867451	71000091
FOSL2	chr11	71250001	71400001	71072128	71095051
BRE	chr11	71250001	71400001	71147448	71550180
TRNAC-GCA	chr16	62530001	62680001	62935749	62935820
RBKS	chr11	71250001	71400001	71550250	71661409
MRPL33	chr11	71250001	71400001	71652999	71662442
SLC4A1AP	chr11	71250001	71400001	71732125	71753507
SUPT7L	chr11	71250001	71400001	71753582	71766351
GPN1	chr11	71250001	71400001	71768498	71796072
CCDC121	chr11	71250001	71400001	71796097	71799329
ZNF512	chr11	71250001	71400001	71803917	71833136
RFXAP	chr12	62300001	62450001	61869600	61896068
SMAD9	chr12	62300001	62450001	61903330	61966268
ALG5	chr12	62300001	62450001	61974895	62002230
EXOSC8	chr12	62300001	62450001	62003540	62011362
SUPT20H	chr12	62300001	62450001	62011274	62047300
POSTN	chr12	62300001	62450001	62423932	62458253
TRPC4	chr12	62300001	62450001	62497288	62709093
RASSF2	chr13	46960001	47110001	46544095	46569158
SLC23A2	chr13	47190001	47340001	46633841	46772996
TMEM230	chr13	47210001	47360001	46856288	46864262
PCNA	chr13	47210001	47360001	46864800	46870372
CDS2	chr13	47210001	47360001	46876073	46921625
PROKR2	chr13	47210001	47360001	46991225	47003919
GPCPD1	chr13	47210001	47360001	47213879	47282392
C13H20orf196	chr13	47210001	47360001	47418863	47527032
CHGB	chr13	47210001	47360001	47569978	47587414
TRMT6	chr13	47210001	47360001	47615544	47627266
MCM8	chr13	47210001	47360001	47627290	47684838
CRLS1	chr13	47210001	47360001	47713968	47736274
LRRN4	chr13	47210001	47360001	47739527	47755422
FERMT1	chr13	47210001	47360001	47778574	47837681
HAO1	chr13	50420001	50570001	50024601	50086221
ADRA1D	chr13	50420001	50570001	50264597	50293882
SMOX	chr13	50420001	50570001	50305402	50340974
RNF24	chr13	50420001	50570001	50520829	50637289

TRNAE-UUC	chrX1	46010001	46160001	46250912	46250984
PANK2	chr13	50420001	50570001	50640389	50669701
MIR103	chr13	50420001	50570001	50646831	50646916
MAVS	chr13	50420001	50570001	50694748	50707107
AP5S1	chr13	50420001	50570001	50734433	50738294
CDC25B	chr13	50420001	50570001	50751016	50761010
CENPB	chr13	50420001	50570001	50772192	50775019
SPEF1	chr13	50420001	50570001	50775106	50781210
C13H20orf27	chr13	50420001	50570001	50785603	50799453
HSPA12B	chr13	50420001	50570001	50799840	50816831
SIGLEC1	chr13	50420001	50570001	50842091	50860436
ADAM33	chr13	50420001	50570001	50865505	50879346
GFRA4	chr13	50420001	50570001	50882036	50886744
ATRN	chr13	50420001	50570001	50894660	51079673
C13H20orf194	chr13	50730001	50880001	51139649	51312804
TRNAS-GGA	chr4	52950001	53100001	53090579	53090650
RAE1	chr13	58510001	58660001	58079192	58097238
SPO11	chr13	58510001	58660001	58104357	58119153
BMP7	chr13	58510001	58660001	58174093	58260125
TFAP2C	chr13	58970001	59120001	58657523	58667140
RTFDC1	chr13	58970001	59120001	58735902	58783189
GCNT7	chr13	58970001	59120001	58749848	58761271
CASS4	chr13	58970001	59120001	58791766	58829365
CSTF1	chr13	58970001	59120001	58833053	58845722
AURKA	chr13	58970001	59120001	58845806	58862526
FAM210B	chr13	58970001	59120001	58864336	58875132
MC3R	chr13	59350001	59500001	58935095	58936185
NSFL1C	chr13	59350001	59500001	59000052	59021299
FKBP1A	chr13	59350001	59500001	59038273	59061754
SDCBP2	chr13	59350001	59500001	59090093	59109110
SNPH	chr13	59350001	59500001	59109662	59153612
RAD21L1	chr13	59350001	59500001	59165671	59201265
C13H20orf202	chr13	59350001	59500001	59230537	59243636
TMEM74B	chr13	59350001	59500001	59244717	59249746
PSMF1	chr13	59350001	59500001	59259919	59302429
RSPO4	chr13	59350001	59500001	59419545	59456637
ANGPT4	chr13	59350001	59500001	59493311	59543214
FAM110A	chr13	59350001	59500001	59563554	59575513
SLC52A3	chr13	59350001	59500001	59592813	59611003
SCRT2	chr13	59350001	59500001	59693358	59707360
SRXN1	chr13	59350001	59500001	59717581	59724941
TCF15	chr13	59350001	59500001	59766065	59772269
CSNK2A1	chr13	59350001	59500001	59825086	59880670
TBC1D20	chr13	59350001	59500001	59896329	59914114
RBCK1	chr13	59350001	59500001	59918875	59937057
COMMD7	chr13	61860001	62010001	61430474	61455479
DNMT3B	chr13	61860001	62010001	61467790	61502090

MAPRE1	chr13	61860001	62010001	61508458	61541861
SUN5	chr13	61860001	62010001	61602832	61626958
BPIFB2	chr13	61860001	62010001	61632481	61650156
BPIFB6	chr13	61860001	62010001	61657835	61672584
BPIFB3	chr13	61860001	62010001	61681607	61696489
BPIFB4	chr13	61860001	62010001	61704744	61730572
BPIFA2	chr13	61860001	62010001	61962034	61971556
BPIFA3	chr13	62530001	62680001	62295723	62306454
BPIFA1	chr13	62530001	62680001	62313752	62321102
BPIFB1	chr13	62530001	62680001	62330853	62371128
CDK5RAP1	chr13	62530001	62680001	62434448	62470312
SNTA1	chr13	62930001	63080001	62475263	62505018
CBFA2T2	chr13	62930001	63080001	62545241	62683536
NECAB3	chr13	62930001	63080001	62691011	62709617
C13H20orf144	chr13	62930001	63080001	62697382	62699145
ACTL10	chr13	62930001	63080001	62702196	62703536
E2F1	chr13	62930001	63080001	62710990	62720515
PXMP4	chr13	62930001	63080001	62730702	62747008
ZNF341	chr13	62930001	63080001	62761999	62803765
CHMP4B	chr13	62930001	63080001	62814614	62861236
TRNAG-UCC	chr13	62930001	63080001	62912627	62912699
**RALY**	chr13	62930001	63080001	62969108	63053806
EIF2S2	chr13	62930001	63080001	63062854	63082442
**ASIP**	chr13	62930001	63080001	63228709	63249542
AHCY	chr13	62930001	63080001	63264059	63279821
ITCH	chr13	62930001	63080001	63363104	63462630
DYNLRB1	chr13	62930001	63080001	63465549	63483202
MAP1LC3A	chr13	62930001	63080001	63498937	63500710
PIGU	chr13	62930001	63080001	63500918	63594821
MAFB	chr13	69200001	69350001	68957927	68961227
TRNAW-CCA	chrX1	14400001	14550001	14663110	14663181
TOP1	chr13	69200001	69350001	69270713	69355431
PLCG1	chr13	69200001	69350001	69366691	69399774
ZHX3	chr13	69200001	69350001	69402826	69515922
LPIN3	chr13	69200001	69350001	69534894	69553728
EMILIN3	chr13	69200001	69350001	69553350	69559361
TRNAE-CUC	chr13	69200001	69350001	69557976	69558047
CHD6	chr13	69200001	69350001	69592893	69796343
PTPRT	chr13	70370001	70520001	70261033	71417055
TRNAC-GCA	chr16	62530001	62680001	62935749	62935820
CBLN4	chr13	82650001	82800001	82836781	82844508
MTDH	chr14	16030001	16180001	15663731	15721499
LAPTM4B	chr14	16030001	16180001	15788488	15854656
MATN2	chr14	16030001	16180001	15872116	16041603
RPL30	chr14	16470001	16620001	16048416	16051780
ERICH5	chr14	16500001	16650001	16063489	16086802
RIDA	chr14	16500001	16650001	16096003	16104730

POP1	chr14	16500001	16650001	16104883	16139688
NIPAL2	chr14	16500001	16650001	16173131	16256193
KCNS2	chr14	16500001	16650001	16394213	16400041
STK3	chr14	16500001	16650001	16433649	16744347
OSR2	chr14	16500001	16650001	16881475	16889328
VPS13B	chr14	16500001	16650001	16964332	17751028
TOX	chr14	57110001	57260001	56824099	57133493
TRNAC-GCA	chr16	62530001	62680001	62935749	62935820
NSMAF	chr14	57110001	57260001	57251301	57317215
SDCBP	chr14	57110001	57260001	57317726	57350168
UBXN2B	chr14	57110001	57260001	57451412	57481003
FAM110B	chr14	57110001	57260001	57631544	57776493
CLPB	chr15	31920001	32070001	31410696	31553755
PHOX2A	chr15	31920001	32070001	31580744	31585333
INPPL1	chr15	31920001	32070001	31585544	31600551
FOLR2	chr15	31920001	32070001	31602384	31605922
TRNAG-CCC	chrX2	10370001	10520001	10239324	10239396
FOLR1	chr15	31920001	32070001	31622904	31634159
ANAPC15	chr15	31920001	32070001	31688212	31691470
LAMTOR1	chr15	31920001	32070001	31697022	31703528
NUMA1	chr15	31920001	32070001	31718205	31787884
IL18BP	chr15	31920001	32070001	31788381	31790497
RNF121	chr15	31920001	32070001	31788430	31874308
ART5	chr15	31920001	32070001	31912716	31916296
ART1	chr15	31920001	32070001	31961266	31972443
NUP98	chr15	31920001	32070001	31985255	32073364
PGAP2	chr15	31920001	32070001	32081071	32094586
RHOG	chr15	31920001	32070001	32095244	32106242
STIM1	chr15	31920001	32070001	32117471	32313714
RRM1	chr15	31920001	32070001	32315709	32355581
TRNAG-CCC	chrX2	10370001	10520001	10239324	10239396
DUSP10	chr16	24020001	24170001	23718507	23758962
TRNAT-UGU	chr16	24020001	24170001	24513444	24513516
HHIPL2	chr16	24020001	24170001	24518158	24545971
TAF1A	chr16	24020001	24170001	24552297	24583583
CAPN2	chr16	26060001	26210001	25579186	25635659
TP53BP2	chr16	26060001	26210001	25637185	25704284
FBXO28	chr16	26160001	26310001	25797222	25826231
DEGS1	chr16	26160001	26310001	25857736	25867324
NVL	chr16	26160001	26310001	25907863	25991438
CNIH4	chr16	26160001	26310001	26021184	26039178
WDR26	chr16	26160001	26310001	26044756	26085641
CNIH3	chr16	26160001	26310001	26233683	26368158
TRNAD-GUC	chr16	26160001	26310001	26576818	26576889
DNAH14	chr16	26160001	26310001	26600001	26998456
ZBTB18	chr16	31960001	32110001	31676613	31685187
AKT3	chr16	31960001	32110001	31877151	32159430

SDCCAG8	chr16	31960001	32110001	32158344	32401912
CEP170	chr16	31960001	32110001	32402081	32538214
PLD5	chr16	33630001	33780001	32961659	33377109
BECN2	chr16	33630001	33780001	33433023	33434318
MAP1LC3C	chr16	33630001	33780001	33461574	33467259
EXO1	chr16	33630001	33780001	33479386	33524373
WDR64	chr16	33630001	33780001	33534070	33669850
CHML	chr16	33630001	33780001	33677364	33686223
OPN3	chr16	33630001	33780001	33707072	33715141
KMO	chr16	33630001	33780001	33714087	33781937
FH	chr16	33630001	33780001	33797989	33822750
RGS7	chr16	33630001	33780001	33938680	34410674
UBE4B	chr16	42420001	42570001	41875171	41997226
RBP7	chr16	42420001	42570001	42034222	42057361
NMNAT1	chr16	42420001	42570001	42070896	42099070
LZIC	chr16	42420001	42570001	42098641	42108160
CTNNBIP1	chr16	42420001	42570001	42125452	42172257
CLSTN1	chr16	42420001	42570001	42200194	42276330
PIK3CD	chr16	42420001	42570001	42276030	42337774
TMEM201	chr16	42420001	42570001	42378250	42404636
SLC25A33	chr16	42420001	42570001	42409266	42441068
SPSB1	chr16	42420001	42570001	42602138	42672843
H6PD	chr16	42420001	42570001	42698678	42738708
MIR34A	chr16	42420001	42570001	42803786	42803892
GPR157	chr16	42420001	42570001	42828376	42910835
CA6	chr16	42430001	42580001	43004716	43034709
GLUL	chr16	62530001	62680001	62094533	62105099
TEDDM1	chr16	62530001	62680001	62112234	62113240
RGSL1	chr16	62530001	62680001	62168698	62235170
TRNAC-ACA	chr16	62530001	62680001	62246657	62246728
RNASEL	chr16	62530001	62680001	62246769	62265717
RGS16	chr16	62530001	62680001	62275493	62281296
RGS8	chr16	62530001	62680001	62328361	62375835
NPL	chr16	62530001	62680001	62472116	62514693
DHX9	chr16	62530001	62680001	62524255	62569488
SHCBP1L	chr16	62530001	62680001	62572702	62611679
LAMC1	chr16	62530001	62680001	62678983	62800726
LAMC2	chr16	62530001	62680001	62840547	62910467
NMNAT2	chr16	62530001	62680001	62912702	63133011
TRNAC-GCA	chr16	62530001	62680001	62935749	62935820
NSL1	chr16	69880001	70030001	69415158	69456616
BATF3	chr16	69900001	70050001	69498880	69512095
FAM71A	chr16	69900001	70050001	69570276	69572494
ATF3	chr16	69900001	70050001	69577756	69590608
NENF	chr16	69900001	70050001	69779747	69787078
TMEM206	chr16	69900001	70050001	69800710	69834107
PPP2R5A	chr16	69900001	70050001	69835172	69902033

DTL	chr16	69900001	70050001	70074829	70126246
INTS7	chr16	69900001	70050001	70126144	70205853
LPGAT1	chr16	69900001	70050001	70221573	70376075
NEK2	chr16	69900001	70050001	70429263	70441739
HHAT	chr16	72040001	72190001	71271943	71639497
TRNAC-GCA	chr16	62530001	62680001	62935749	62935820
SERTAD4	chr16	72070001	72220001	71719132	71733081
SYT14	chr16	72070001	72220001	71798086	71934364
DIEXF	chr16	72070001	72220001	72072998	72102018
IRF6	chr16	72070001	72220001	72127836	72144263
C16H1orf74	chr16	72070001	72220001	72147939	72150096
TRAF3IP3	chr16	72070001	72220001	72150101	72173649
HSD11B1	chr16	72070001	72220001	72192175	72232663
G0S2	chr16	72070001	72220001	72264418	72265385
LAMB3	chr16	72070001	72220001	72301952	72344457
CAMK1G	chr16	72070001	72220001	72345416	72378212
NEK7	chr16	75970001	76120001	75639146	75776800
ATP6V1G3	chr16	75970001	76120001	75974525	75995529
PTPRC	chr16	75970001	76120001	76103209	76232274
PAFAH1B1	chr19	23760001	23910001	23302330	23368013
CLUH	chr19	23760001	23910001	23373646	23395027
RAP1GAP2	chr19	23760001	23910001	23437609	23652991
SPATA22	chr19	23760001	23910001	23998431	24013174
ASPA	chr19	23760001	23910001	24018930	24037078
TRPV3	chr19	23760001	23910001	24042198	24076976
TRPV1	chr19	23760001	23910001	24082035	24111002
SHPK	chr19	23760001	23910001	24128599	24147165
CTNS	chr19	23760001	23910001	24147314	24166212
TAX1BP3	chr19	23760001	23910001	24166707	24172070
EMC6	chr19	23760001	23910001	24171946	24173008
P2RX5	chr19	23760001	23910001	24175226	24191058
ITGAE	chr19	23760001	23910001	24198191	24258647
GSG2	chr19	23760001	23910001	24204550	24207767
NCBP3	chr19	23760001	23910001	24268865	24294295
CAMKK1	chr19	23760001	23910001	24299517	24329180
P2RX1	chr19	23760001	23910001	24333541	24352309
FAM134B	chr20	56960001	57110001	56450873	56607068
ZNF622	chr20	56960001	57110001	56614034	56627946
11-Mar	chr20	56960001	57110001	56903699	57019253
FBXL7	chr20	56960001	57110001	57170615	57623887
ICE1	chr20	68100001	68250001	67619014	67689571
ADAMTS16	chr20	68100001	68250001	67786480	67969514
TRNAS-GGA	chr4	52950001	53100001	53090579	53090650
IRX4	chr20	70920001	71070001	70764248	70772443
NDUFS6	chr20	70920001	71070001	70826012	70831065
MRPL36	chr20	70920001	71070001	70832013	70833718
LPCAT1	chr20	70920001	71070001	70963369	71000848

SLC6A3	chr20	70920001	71070001	71014677	71048556
SLC12A7	chr20	70920001	71070001	71096461	71133473
NKD2	chr20	70920001	71070001	71137228	71158931
TRIP13	chr20	70920001	71070001	71206857	71222292
BRD9	chr20	70920001	71070001	71222238	71240459
TERT	chr20	70920001	71070001	71256520	71274686
SLC6A18	chr20	70920001	71070001	71278518	71293544
SLC6A19	chr20	70920001	71070001	71295262	71311801
TRNAS-GGA	chr4	52950001	53100001	53090579	53090650
TPPP	chr20	70920001	71070001	71380831	71402958
CEP72	chr20	70920001	71070001	71405220	71445196
TOPAZ1	chr22	16500001	16650001	16017411	16080362
TCAIM	chr22	16510001	16660001	16082199	16119825
ZNF445	chr22	16510001	16660001	16140516	16168275
ZNF852	chr22	16510001	16660001	16189711	16202009
ZNF502	chr22	16510001	16660001	16287427	16297792
ZNF501	chr22	16510001	16660001	16309551	16310546
KIAA1143	chr22	16510001	16660001	16318453	16327891
KIF15	chr22	16510001	16660001	16327926	16386693
TATDN2	chr22	16510001	16660001	16453967	16476805
IRAK2	chr22	16920001	17070001	16479062	16533738
VHL	chr22	16920001	17070001	16540685	16547653
BRK1	chr22	16920001	17070001	16552011	16559676
FANCD2OS	chr22	16920001	17070001	16561690	16565016
FANCD2	chr22	16920001	17070001	16565691	16614581
EMC3	chr22	16920001	17070001	16614568	16629878
PRRT3	chr22	16920001	17070001	16637743	16644601
CRELD1	chr22	16920001	17070001	16645360	16653853
IL17RC	chr22	16920001	17070001	16653881	16665409
IL17RE	chr22	16920001	17070001	16665446	16676596
JAGN1	chr22	16920001	17070001	16682058	16685009
CIDEC	chr22	16920001	17070001	16688728	16698787
RPUSD3	chr22	16920001	17070001	16704276	16713792
ARPC4	chr22	16920001	17070001	16739519	16749345
TADA3	chr22	16920001	17070001	16749506	16760007
CAMK1	chr22	16920001	17070001	16770761	16781794
OGG1	chr22	16920001	17070001	16781738	16787900
BRPF1	chr22	16920001	17070001	16788964	16804596
CPNE9	chr22	16920001	17070001	16806646	16826679
MTMR14	chr22	16920001	17070001	16827861	16871844
LHFPL4	chr22	16920001	17070001	16905086	16938585
SETD5	chr22	17430001	17580001	16950600	17030658
THUMPD3	chr22	17430001	17580001	17048771	17070472
SRGAP3	chr22	17430001	17580001	17156766	17404110
RAD18	chr22	17430001	17580001	17414359	17523990
OXTR	chr22	17430001	17580001	17594776	17607958
CAV3	chr22	17430001	17580001	17610114	17622648

SSUH2	chr22	17430001	17580001	17670175	17692990
LMCD1	chr22	17430001	17580001	17740028	17796768
EXOC2	chr23	680001	830001	237311	359937
FOXQ1	chr23	680001	830001	641272	643823
FOXF2	chr23	680001	830001	691668	696627
FOXC1	chr23	680001	830001	780584	784092
GMDS	chr23	680001	830001	792203	1217301
RUNX2	chr23	30210001	30360001	29926106	30269961
TRNAE-UUC	chrX1	46010001	46160001	46250912	46250984
SUPT3H	chr23	30210001	30360001	30223937	30622509
C24H18orf25	chr24	46580001	46730001	46164903	46247071
RNF165	chr24	46580001	46730001	46308979	46417069
LOXHD1	chr24	46580001	46730001	46431005	46629483
ST8SIA5	chr24	46580001	46730001	46648415	46718152
PIAS2	chr24	46580001	46730001	46753797	46854848
KATNAL2	chr24	46580001	46730001	46946336	46993318
HDHD2	chr24	46580001	46730001	46998128	47043825
IER3IP1	chr24	46580001	46730001	47046744	47064735
SKOR2	chr24	46580001	46730001	47100548	47134783
C25H16orf45	chr25	14210001	14360001	13735033	13881095
KIAA0430	chr25	14210001	14360001	13900354	13940070
NDE1	chr25	14210001	14360001	13944989	13981278
MYH11	chr25	14210001	14360001	13981844	14109280
FOPNL	chr25	14210001	14360001	14115214	14134357
TRNAC-GCA	chr16	62530001	62680001	62935749	62935820
ABCC1	chr25	14210001	14360001	14181754	14337412
ABCC6	chr25	14210001	14360001	14342003	14407612
PLPP4	chr26	11740001	11890001	11346975	11490960
SEC23IP	chr26	11740001	11890001	11949606	11988858
MCMBP	chr26	11740001	11890001	12003875	12042329
INPP5F	chr26	11740001	11890001	12042910	12135028
BAG3	chr26	11740001	11890001	12206500	12229905
TIAL1	chr26	11740001	11890001	12276234	12298428
TRNAC-ACA	chr16	62530001	62680001	62246657	62246728
RGS10	chr26	11750001	11900001	12317355	12357120
MBL2	chr26	45100001	45250001	45038445	45045199
C28H10orf128	chr28	2760001	2910001	2312335	2343429
FAM170B	chr28	2760001	2910001	2361936	2364098
VSTM4	chr28	2760001	2910001	2380016	2459503
WDFY4	chr28	2760001	2910001	2478311	2732087
LRRC18	chr28	2760001	2910001	2518800	2536516
ARHGAP22	chr28	3350001	3500001	2751853	2933545
MAPK8	chr28	3380001	3530001	2941175	3039589
FRMPD2	chr28	3380001	3530001	3087917	3155034
PTPN20	chr28	3380001	3530001	3207633	3233696
GDF10	chr28	3380001	3530001	3460297	3473327
GDF2	chr28	3380001	3530001	3482208	3486651

RBP3	chr28	3380001	3530001	3494829	3504269
ZNF488	chr28	3380001	3530001	3511701	3521545
ANTXRL	chr28	3380001	3530001	3727694	3772919
TRNAA-AGC	chr28	3380001	3530001	3823011	3823082
GPRIN2	chr28	3380001	3530001	3917031	3935587
FAM35A	chr28	3400001	3550001	3966919	4070554
DEAF1	chr29	46220001	46370001	45775415	45801060
DRD4	chr29	46230001	46380001	45804141	45806873
SCT	chr29	46230001	46380001	45817070	45818263
CDHR5	chr29	46230001	46380001	45820504	45827415
IRF7	chr29	46230001	46380001	45828394	45831518
PHRF1	chr29	46230001	46380001	45832047	45856007
RASSF7	chr29	46230001	46380001	45864757	45867360
LMNTD2	chr29	46230001	46380001	45870525	45876726
LRRC56	chr29	46230001	46380001	45876806	45890449
HRAS	chr29	46230001	46380001	45894564	45897458
RNH1	chr29	46230001	46380001	45921210	45936419
PTDSS2	chr29	46230001	46380001	45937562	45962964
ANO9	chr29	46230001	46380001	45977907	45999636
SIGIRR	chr29	46230001	46380001	46000666	46009408
PKP3	chr29	46230001	46380001	46010418	46015001
PGGHG	chr29	46230001	46380001	46035921	46042088
IFITM5	chr29	46230001	46380001	46044242	46046440
B4GALNT4	chr29	46230001	46380001	46147705	46150925
AP2A2	chr29	46230001	46380001	46174668	46242919
MUC6	chr29	46230001	46380001	46244530	46267909
GPR152	chr29	46230001	46380001	46389008	46392442
TMEM134	chr29	46230001	46380001	46402781	46407781
AIP	chr29	46230001	46380001	46417047	46423260
PITPNM1	chr29	46230001	46380001	46423853	46437448
CDK2AP2	chr29	46230001	46380001	46438685	46441018
CABP2	chr29	46230001	46380001	46446866	46451638
NDUFV1	chr29	46230001	46380001	46509096	46514891
NUDT8	chr29	46230001	46380001	46522488	46524991
TBX10	chr29	46230001	46380001	46526481	46535231
UNC93B1	chr29	46230001	46380001	46561947	46572020
ALDH3B1	chr29	46230001	46380001	46581621	46601665
NDUFS8	chr29	46230001	46380001	46603414	46607673
TCIRG1	chr29	46230001	46380001	46610608	46622361
CHKA	chr29	46230001	46380001	46624210	46681700
KMT5B	chr29	46230001	46380001	46706234	46764579
C29H11orf24	chr29	46230001	46380001	46800409	46809400
DUSP8	chr29	51330001	51480001	50962017	50977940
MOB2	chr29	51330001	51480001	50987206	51039264
BRSK2	chr29	51330001	51480001	51047154	51097037
TOLLIP	chr29	51330001	51480001	51162053	51177156
MUC5AC	chr29	51330001	51480001	51246526	51276626

MUC2	chr29	51330001	51480001	51317872	51331926
EDA	chrX1	12290001	12440001	11557022	11895599
CDX4	chrX1	12290001	12440001	12054961	12063592
FAM155B	chrX1	12290001	12440001	12074820	12103927
PJA1	chrX1	12290001	12440001	12110252	12115011
STARD8	chrX1	12290001	12440001	12400560	12415129
YIPF6	chrX1	12290001	12440001	12585345	12617792
OPHN1	chrX1	12290001	12440001	12692459	13271064
AR	chrX1	13830001	13980001	13729623	13934065
MAGED1	chrX1	14400001	14550001	14646763	14730609
TRNAW-CCA	chrX1	14400001	14550001	14663110	14663181
GSPT2	chrX1	14400001	14550001	14796387	14798923
EDA2R	chrX1	18150001	18300001	18412588	18508471
PCYT1B	chrX1	45930001	46080001	45429192	45570963
PDK3	chrX1	46010001	46160001	45589783	45669513
ZFX	chrX1	46010001	46160001	45877351	45913094
EIF2S3	chrX1	46010001	46160001	45967962	45984248
KLHL15	chrX1	46010001	46160001	46006667	46039781
CXHXorf58	chrX1	46010001	46160001	46086883	46109876
APOO	chrX1	46010001	46160001	46109971	46177359
SAT1	chrX1	46010001	46160001	46220673	46223754
TRNAE-UUC	chrX1	46010001	46160001	46250912	46250984
ACOT9	chrX1	46010001	46160001	46265173	46295513
PRDX4	chrX1	46010001	46160001	46307957	46319355
TRNAC-ACA	chr16	62530001	62680001	62246657	62246728
PTCHD1	chrX1	46020001	46170001	46585313	46636740
ARHGEF6	chrX2	10370001	10520001	10085939	10202183
CD40LG	chrX2	10370001	10520001	10210614	10224367
TRNAG-CCC	chrX2	10370001	10520001	10239324	10239396
VGLL1	chrX2	10370001	10520001	10330275	10338073
ADGRG4	chrX2	10370001	10520001	10378603	10476767
BRS3	chrX2	10370001	10520001	10550184	10554622
HTATSF1	chrX2	10370001	10520001	10558643	10575514
MAP7D3	chrX2	10370001	10520001	10596017	10629199
TRNAW-CCA	chrX1	14400001	14550001	14663110	14663181
MOSPD1	chrX2	10370001	10520001	10638690	10662600
TRNAC-GCA	chr16	62530001	62680001	62935749	62935820
FAM122B	chrX2	10370001	10520001	10745390	10769280
PLAC1	chrX2	10370001	10520001	10913401	10950105
PHF6	chrX2	10410001	10560001	10959652	11007335
HPRT1	chrX2	10470001	10620001	11036061	11066425

ıere goats(CDMC,UC,IMC,ILC)

ZFst	log 2 (θπ ratio)
2.830128008	-1.098814506
2.830128008	-1.098814506
2.830128008	-1.098814506
4.408351838	-1.197269104
4.408351838	-1.197269104
4.408351838	-1.197269104
4.304015171	-1.176576709
4.2556205	-1.15392797
4.2556205	-1.15392797
3.816634766	-1.145286177
3.816634766	-1.145286177
3.816634766	-1.145286177
3.816634766	-1.145286177
3.816634766	-1.145286177
5.493789747	1.676718744
3.238363492	-0.910773058
3.238363492	-0.910773058
3.238363492	-0.910773058
3.767169191	1.355072662
2.760128269	1.185676287
2.760128269	1.185676287
2.760128269	1.185676287
2.760128269	1.185676287
2.760128269	1.185676287
2.760128269	1.185676287
2.760128269	1.185676287
2.765992745	-1.981081018
3.362163418	-1.376694972
3.362163418	-1.376694972
2.554276682	-1.711700505
3.343312104	-1.165207895
3.343312104	-1.165207895
4.2556205	-1.15392797
3.33967443	-1.104076998
3.33967443	-1.104076998
3.33967443	-1.104076998
3.33967443	-1.104076998
3.33967443	-1.104076998
3.33967443	-1.104076998
3.33967443	-1.104076998
3.33967443	-1.104076998
3.33967443	-1.104076998
3.33967443	-1.104076998

3.33967443	-1.104076998
4.407790888	1.145351386
3.755695217	-1.016833084
3.755695217	-1.016833084
3.755695217	-1.016833084
3.755695217	-1.016833084
3.755695217	-1.016833084
3.755695217	-1.016833084
2.860810263	-0.907251314
2.860810263	-0.907251314
2.860810263	-0.907251314
2.860810263	-0.907251314
4.407790888	1.145351386
2.860810263	-0.907251314
2.860810263	-0.907251314
2.860810263	-0.907251314
2.860810263	-0.907251314
4.326759136	-0.932085986
2.860810263	-0.907251314
2.860810263	-0.907251314
2.508788751	-1.061601281
3.406274472	-1.30293792
3.406274472	-1.30293792
3.406274472	-1.30293792
3.406274472	-1.30293792
3.406274472	-1.30293792
3.406274472	-1.30293792
3.406274472	-1.30293792
3.406274472	-1.30293792
3.406274472	-1.30293792
2.619907812	-0.932636633
6.204615165	-0.939260842
2.619907812	-0.932636633
4.62275366	-1.02385636
4.62275366	-1.02385636
4.62275366	-1.02385636
4.62275366	-1.02385636
4.62275366	-1.02385636
4.62275366	-1.02385636
4.620254884	-1.054392297
3.174041245	-0.995678394
3.174041245	-0.995678394
3.174041245	-0.995678394
3.174041245	-0.995678394
3.174041245	-0.995678394
2.521384625	-0.999423037
2.521384625	-0.999423037

2.521384625	-0.999423037
2.521384625	-0.999423037
2.521384625	-0.999423037
4.078904307	0.909581116
4.078904307	0.909581116
4.078904307	0.909581116
4.078904307	0.909581116
4.078904307	0.909581116
4.078904307	0.909581116
4.078904307	0.909581116
4.078904307	0.909581116
4.078904307	0.909581116
4.078904307	0.909581116
4.078904307	0.909581116
4.078904307	0.909581116
4.078904307	0.909581116
4.078904307	0.909581116
4.078904307	0.909581116
4.078904307	0.909581116
4.078904307	0.909581116
3.789029236	-0.943971461
3.789029236	-0.943971461
3.789029236	-0.943971461
3.789029236	-0.943971461
3.789029236	-0.943971461
3.789029236	-0.943971461
3.789029236	-0.943971461
3.789029236	-0.943971461
3.789029236	-0.943971461
3.789029236	-0.943971461
3.789029236	-0.943971461
3.789029236	-0.943971461
3.789029236	-0.943971461
3.789029236	-0.943971461
3.789029236	-0.943971461
3.789029236	-0.943971461
2.97141937	-1.083446923
2.97141937	-1.083446923
2.918248126	-1.282438723
2.800074696	-1.238544507
2.800074696	-1.238544507
2.800074696	-1.238544507
2.800074696	-1.238544507
2.800074696	-1.238544507
2.800074696	-1.238544507
2.800074696	-1.238544507
2.800074696	-1.238544507

2.800074696	-1.238544507
2.800074696	-1.238544507
2.800074696	-1.238544507
2.800074696	-1.238544507
2.800074696	-1.238544507
2.800074696	-1.238544507
2.800074696	-1.238544507
3.670736817	-2.021511467
5.910728453	-1.398969131
5.258819765	-1.359194004
3.831389447	-1.102217455
3.831389447	-1.102217455
3.831389447	-1.102217455
3.831389447	-1.102217455
4.347021324	-1.311148256
4.347021324	-1.311148256
4.347021324	-1.311148256
4.347021324	-1.311148256
4.347021324	-1.311148256
4.347021324	-1.311148256
4.347021324	-1.311148256
2.938255336	-0.924125133
2.938255336	-0.924125133
2.938255336	-0.924125133
2.938255336	-0.924125133
2.938255336	-0.924125133
2.938255336	-0.924125133
2.938255336	-0.924125133
2.938255336	-0.924125133
3.043985879	-1.03209363
5.68135097	-1.211564852
5.68135097	-1.211564852
5.68135097	-1.211564852
5.68135097	-1.211564852
5.68135097	-1.211564852
5.68135097	-1.211564852
5.68135097	-1.211564852
5.68135097	-1.211564852
5.68135097	-1.211564852
5.68135097	-1.211564852
5.68135097	-1.211564852
5.68135097	-1.211564852
5.68135097	-1.211564852
5.68135097	-1.211564852
5.68135097	-1.211564852
5.68135097	-1.211564852
5.68135097	-1.211564852

5.68135097	-1.211564852
3.728038691	-1.226003675
3.728038691	-1.226003675
3.728038691	-1.226003675
4.326759136	-0.932085986
4.326759136	-0.932085986
4.326759136	-0.932085986
4.326759136	-0.932085986
4.41615414	-0.960159735
4.41615414	-0.960159735
4.41615414	-0.960159735
4.41615414	-0.960159735
4.41615414	-0.960159735
4.41615414	-0.960159735
4.41615414	-0.960159735
4.41615414	-0.960159735
6.204615165	-0.939260842
3.031220021	-2.544560985
3.031220021	-2.544560985
2.690213523	-1.00433459
2.690213523	-1.00433459
2.690213523	-1.00433459
2.690213523	-1.00433459
2.690213523	-1.00433459
3.579403986	-1.141461934
4.433424595	1.429374937
4.433424595	1.429374937
4.433424595	1.429374937
4.433424595	1.429374937
3.559532764	-1.271600541
3.613077974	-1.044538396
3.67707725	-0.971713757
3.67707725	-0.971713757
3.67707725	-0.971713757
2.838304277	-1.015374195
2.9174152	-0.921390165
2.9174152	-0.921390165
2.9174152	-0.921390165
2.9174152	-0.921390165
2.9174152	-0.921390165
3.061341326	-0.942307133
2.949219355	-0.968604804
4.407790888	1.145351386
2.653700789	-0.911044318
2.653700789	-0.911044318
2.653700789	-0.911044318
2.653700789	-0.911044318

2.653700789	-0.911044318
2.653700789	-0.911044318
2.653700789	-0.911044318
3.843747341	-0.923577725
3.843747341	-0.923577725
4.407790888	1.145351386
3.084561249	0.999567127
3.613876903	-1.1824586
4.571690228	0.941481964
4.571690228	0.941481964
4.571690228	0.941481964
4.571690228	0.941481964
4.571690228	0.941481964
4.571690228	0.941481964
3.540035508	1.369494402
3.540035508	1.369494402
3.540035508	1.369494402
3.540035508	1.369494402
3.767169191	1.355072662
3.733767179	-0.932912035
4.407790888	1.145351386
3.850053777	-0.973979029
3.850053777	-0.973979029
3.850053777	-0.973979029
3.850053777	-0.973979029
3.850053777	-0.973979029
3.850053777	-0.973979029
6.204615165	-0.939260842
2.54278571	0.960734102
2.54278571	0.960734102
2.54278571	0.960734102
2.54278571	0.960734102
2.54278571	0.960734102
2.54278571	0.960734102
3.609644281	-1.373327247
3.609644281	-1.373327247
3.609644281	-1.373327247
3.609644281	-1.373327247
3.609644281	-1.373327247
3.609644281	-1.373327247
3.609644281	-1.373327247
3.609644281	-1.373327247
3.609644281	-1.373327247
3.609644281	-1.373327247
6.204615165	-0.939260842
3.609644281	-1.373327247
3.609644281	-1.373327247

3.609644281	-1.373327247
3.609644281	-1.373327247
3.609644281	-1.373327247
3.609644281	-1.373327247
3.609644281	-1.373327247
3.609644281	-1.373327247
3.609644281	-1.373327247
3.463253376	-1.252913417
3.463253376	-1.252913417
3.463253376	-1.252913417
3.463253376	-1.252913417
3.18617816	-1.009843833
3.18617816	-1.009843833
3.18617816	-1.009843833
3.18617816	-1.009843833
3.18617816	-1.009843833
3.18617816	-1.009843833
3.18617816	-1.009843833
3.18617816	-1.009843833
3.18617816	-1.009843833
3.18617816	-1.009843833
3.18617816	-1.009843833
3.18617816	-1.009843833
3.18617816	-1.009843833
3.18617816	-1.009843833
4.326759136	-0.932085986
6.204615165	-0.939260842
3.18617816	-1.009843833
2.875513948	0.916935177
2.945071726	0.934780673
2.945071726	0.934780673
2.945071726	0.934780673
2.945071726	0.934780673
2.945071726	0.934780673
6.204615165	-0.939260842
2.945071726	0.934780673
2.945071726	0.934780673
2.945071726	0.934780673
2.945071726	0.934780673
2.945071726	0.934780673
2.945071726	0.934780673
2.945071726	0.934780673
2.945071726	0.934780673
2.945071726	0.934780673
2.945071726	0.934780673
2.945071726	0.934780673
2.945071726	0.934780673

2.945071726	0.934780673
3.767169191	1.355072662
2.945071726	0.934780673
2.945071726	0.934780673
2.945071726	0.934780673
2.945071726	0.934780673
2.945071726	0.934780673
2.945071726	0.934780673
2.945071726	0.934780673
2.945071726	0.934780673
3.325735677	-0.984787915
3.366685014	-1.092032594
3.366685014	-1.092032594
3.366685014	-1.092032594
3.366685014	-1.092032594
3.366685014	-1.092032594
3.366685014	-1.092032594
3.366685014	-1.092032594
3.366685014	-1.092032594
3.366685014	-1.092032594
3.366685014	-1.092032594
3.366685014	-1.092032594
3.541395386	-1.144648099
3.541395386	-1.144648099
3.541395386	-1.144648099
3.541395386	-1.144648099
3.541395386	-1.144648099
3.541395386	-1.144648099
3.541395386	-1.144648099
3.541395386	-1.144648099
3.541395386	-1.144648099
3.541395386	-1.144648099
3.541395386	-1.144648099
3.541395386	-1.144648099
3.541395386	-1.144648099
3.541395386	-1.144648099
6.204615165	-0.939260842
3.541395386	-1.144648099
3.108665093	-0.929885497
3.108665093	-0.929885497
3.108665093	-0.929885497
3.108665093	-0.929885497
6.204615165	-0.939260842
3.108665093	-0.929885497
3.108665093	-0.929885497
3.108665093	-0.929885497
3.108665093	-0.929885497

2.917364205	-0.938431151
2.917364205	-0.938431151
2.50868676	-2.284897364
2.50868676	-2.284897364
3.440951371	1.066054738
3.440951371	1.066054738
3.440951371	1.066054738
3.440951371	1.066054738
3.440951371	1.066054738
3.440951371	1.066054738
3.440951371	1.066054738
6.204615165	-0.939260842
2.892886394	-0.934841325
2.989233776	-0.9776677
2.989233776	-0.9776677
2.989233776	-0.9776677
2.989233776	-0.9776677
2.989233776	-0.9776677
2.989233776	-0.9776677
2.989233776	-0.9776677
2.989233776	-0.9776677
2.989233776	-0.9776677
4.407790888	1.145351386
2.989233776	-0.9776677
2.989233776	-0.9776677
5.355626106	1.20614307
5.355626106	1.20614307
5.849465933	1.301821736
6.479888542	1.486199493
7.493252898	1.331705216
7.493252898	1.331705216
7.493252898	1.331705216
7.493252898	1.331705216
7.493252898	1.331705216
7.493252898	1.331705216
7.493252898	1.331705216
7.493252898	1.331705216
7.493252898	1.331705216
7.493252898	1.331705216
7.493252898	1.331705216
7.493252898	1.331705216
7.493252898	1.331705216
7.493252898	1.331705216
7.493252898	1.331705216
7.493252898	1.331705216
7.493252898	1.331705216
7.493252898	1.331705216

7.493252898	1.331705216
7.493252898	1.331705216
7.493252898	1.331705216
7.493252898	1.331705216
7.493252898	1.331705216
7.493252898	1.331705216
7.493252898	1.331705216
7.493252898	1.331705216
7.493252898	1.331705216
7.493252898	1.331705216
7.493252898	1.331705216
7.493252898	1.331705216
7.493252898	1.331705216
7.493252898	1.331705216
7.493252898	1.331705216
7.493252898	1.331705216
7.493252898	1.331705216
7.493252898	1.331705216
7.493252898	1.331705216
7.493252898	1.331705216
2.533725521	1.098284796
2.533725521	1.098284796
2.533725521	1.098284796
2.533725521	1.098284796
2.533725521	1.098284796
2.533725521	1.098284796
2.533725521	1.098284796
2.533725521	1.098284796
2.533725521	1.098284796
2.533725521	1.098284796
7.246893935	1.366699889
10.12898614	2.22891102
10.12898614	2.22891102
10.12898614	2.22891102
10.12898614	2.22891102
10.12898614	2.22891102
10.12898614	2.22891102
10.12898614	2.22891102
5.078261916	1.631756201
5.078261916	1.631756201
5.078261916	1.631756201
5.078261916	1.631756201
5.078261916	1.631756201
5.078261916	1.631756201
5.078261916	1.631756201
5.078261916	1.631756201
5.078261916	1.631756201

2.998310964	-1.01187887
2.98348829	-0.937878288
3.20015091	-0.918115033
3.20015091	-0.918115033
3.20015091	-0.918115033
5.493789747	1.676718744
3.20015091	-0.918115033
3.20015091	-0.918115033
3.20015091	-0.918115033
3.20015091	-0.918115033
3.20015091	-0.918115033
3.372787468	1.157496503
3.372787468	1.157496503
3.372787468	1.157496503
6.204615165	-0.939260842
3.372787468	1.157496503
3.372787468	1.157496503
3.372787468	1.157496503
3.372787468	1.157496503
3.372787468	1.157496503
3.372787468	1.157496503
3.372787468	1.157496503
2.550401029	-0.988504361
2.550401029	-0.988504361
2.550401029	-0.988504361
2.550401029	-0.988504361
2.550401029	-0.988504361
2.550401029	-0.988504361
2.550401029	-0.988504361
2.603470282	-0.905899084
2.676121784	-1.443006615
4.413791352	-1.506762581
4.413791352	-1.506762581
4.413791352	-1.506762581
4.413791352	-1.506762581
4.413791352	-1.506762581
4.413791352	-1.506762581
4.413791352	-1.506762581
4.413791352	-1.506762581
4.413791352	-1.506762581
4.413791352	-1.506762581
4.413791352	-1.506762581
4.413791352	-1.506762581
3.552393402	-1.493296513
3.552393402	-1.493296513
3.552393402	-1.493296513
3.552393402	-1.493296513

5.493789747	1.676718744
3.552393402	-1.493296513
3.552393402	-1.493296513
3.552393402	-1.493296513
3.552393402	-1.493296513
3.552393402	-1.493296513
3.552393402	-1.493296513
3.552393402	-1.493296513
3.552393402	-1.493296513
3.552393402	-1.493296513
3.552393402	-1.493296513
3.552393402	-1.493296513
3.552393402	-1.493296513
3.552393402	-1.493296513
2.534728431	-1.451662024
4.326759136	-0.932085986
2.699562687	-0.934014171
2.699562687	-0.934014171
2.699562687	-0.934014171
2.989182781	-2.602963759
2.989182781	-2.602963759
2.989182781	-2.602963759
2.989182781	-2.602963759
2.989182781	-2.602963759
2.989182781	-2.602963759
2.989182781	-2.602963759
2.998191975	-1.006651708
2.998191975	-1.006651708
2.998191975	-1.006651708
2.998191975	-1.006651708
2.998191975	-1.006651708
2.998191975	-1.006651708
2.998191975	-1.006651708
2.998191975	-1.006651708
2.998191975	-1.006651708
2.998191975	-1.006651708
2.998191975	-1.006651708
2.998191975	-1.006651708
2.998191975	-1.006651708
2.998191975	-1.006651708
2.998191975	-1.006651708
2.998191975	-1.006651708
2.998191975	-1.006651708
2.998191975	-1.006651708
2.998191975	-1.006651708
2.550622009	-1.20889443
2.550622009	-1.20889443

2.550622009	-1.20889443
2.550622009	-1.20889443
2.550622009	-1.20889443
2.550622009	-1.20889443
2.550622009	-1.20889443
2.550622009	-1.20889443
2.550622009	-1.20889443
3.757446061	-2.189680297
3.757446061	-2.189680297
3.757446061	-2.189680297
3.757446061	-2.189680297
4.836832511	-0.979373349
4.836832511	-0.979373349
4.836832511	-0.979373349
4.836832511	-0.979373349
4.836832511	-0.979373349
4.836832511	-0.979373349
4.836832511	-0.979373349
4.836832511	-0.979373349
4.836832511	-0.979373349
4.836832511	-0.979373349
4.836832511	-0.979373349
4.836832511	-0.979373349
4.836832511	-0.979373349
4.836832511	-0.979373349
4.836832511	-0.979373349
4.836832511	-0.979373349
4.836832511	-0.979373349
4.836832511	-0.979373349
2.69527907	-1.363270987
4.407790888	1.145351386
2.69527907	-1.363270987
2.69527907	-1.363270987
2.69527907	-1.363270987
2.69527907	-1.363270987
2.69527907	-1.363270987
2.69527907	-1.363270987
2.69527907	-1.363270987
3.304130609	-2.01100636
6.204615165	-0.939260842
2.753447867	-0.964094616
4.37579975	2.015176396
4.37579975	2.015176396
4.37579975	2.015176396
5.046304774	1.003314389
6.006871853	1.186563944
6.006871853	1.186563944

6.006871853	1.186563944
6.006871853	1.186563944
6.006871853	1.186563944
6.006871853	1.186563944
6.006871853	1.186563944
6.006871853	1.186563944
3.412733895	-1.543298969
6.204615165	-0.939260842
3.412733895	-1.543298969
3.412733895	-1.543298969
3.412733895	-1.543298969
3.412733895	-1.543298969
3.401225924	1.743385708
3.401225924	1.743385708
3.401225924	1.743385708
3.401225924	1.743385708
3.767169191	1.355072662
3.401225924	1.743385708
3.401225924	1.743385708
3.401225924	1.743385708
3.401225924	1.743385708
3.401225924	1.743385708
3.401225924	1.743385708
3.401225924	1.743385708
3.401225924	1.743385708
3.401225924	1.743385708
3.401225924	1.743385708
3.401225924	1.743385708
3.401225924	1.743385708
3.401225924	1.743385708
3.767169191	1.355072662
2.775749872	-1.027380861
2.775749872	-1.027380861
2.775749872	-1.027380861
2.775749872	-1.027380861
2.854639815	-0.994240731
2.854639815	-0.994240731
2.887990832	-1.367359524
2.887990832	-1.367359524
2.887990832	-1.367359524
2.887990832	-1.367359524
2.887990832	-1.367359524
2.887990832	-1.367359524
2.887990832	-1.367359524
2.887990832	-1.367359524
3.396534344	1.737427183
3.396534344	1.737427183

3.396534344	1.737427183
3.396534344	1.737427183
4.25271376	1.306320576
4.25271376	1.306320576
4.25271376	1.306320576
4.25271376	1.306320576
4.25271376	1.306320576
4.25271376	1.306320576
4.25271376	1.306320576
4.25271376	1.306320576
4.25271376	1.306320576
4.25271376	1.306320576
3.435239882	-1.080698059
3.435239882	-1.080698059
3.435239882	-1.080698059
3.435239882	-1.080698059
3.435239882	-1.080698059
3.435239882	-1.080698059
3.435239882	-1.080698059
3.435239882	-1.080698059
3.435239882	-1.080698059
3.435239882	-1.080698059
3.435239882	-1.080698059
3.435239882	-1.080698059
3.435239882	-1.080698059
3.43022533	-1.053493701
6.204615165	-0.939260842
6.204615165	-0.939260842
6.204615165	-0.939260842
6.204615165	-0.939260842
6.204615165	-0.939260842
6.204615165	-0.939260842
6.204615165	-0.939260842
6.204615165	-0.939260842
6.204615165	-0.939260842
6.204615165	-0.939260842
6.204615165	-0.939260842
6.204615165	-0.939260842
6.204615165	-0.939260842
6.204615165	-0.939260842
3.284191393	-0.924398915
3.360106602	-0.925768606
3.360106602	-0.925768606
3.360106602	-0.925768606
3.360106602	-0.925768606
3.360106602	-0.925768606
3.360106602	-0.925768606

3.360106602	-0.925768606
3.360106602	-0.925768606
3.360106602	-0.925768606
3.360106602	-0.925768606
3.90837556	-0.930160375
6.204615165	-0.939260842
4.251727848	-0.917297411
4.251727848	-0.917297411
4.251727848	-0.917297411
4.251727848	-0.917297411
4.251727848	-0.917297411
4.251727848	-0.917297411
4.251727848	-0.917297411
4.251727848	-0.917297411
4.251727848	-0.917297411
4.251727848	-0.917297411
2.644453616	-1.015957574
2.644453616	-1.015957574
2.644453616	-1.015957574
3.059012535	-1.238544507
3.059012535	-1.238544507
3.059012535	-1.238544507
3.059012535	-1.238544507
3.059012535	-1.238544507
3.059012535	-1.238544507
3.059012535	-1.238544507
3.059012535	-1.238544507
3.059012535	-1.238544507
3.059012535	-1.238544507
3.059012535	-1.238544507
3.059012535	-1.238544507
3.059012535	-1.238544507
3.059012535	-1.238544507
3.059012535	-1.238544507
3.059012535	-1.238544507
3.059012535	-1.238544507
4.249297065	-2.184424571
4.249297065	-2.184424571
4.249297065	-2.184424571
4.249297065	-2.184424571
2.617341041	-1.236163541
2.617341041	-1.236163541
4.326759136	-0.932085986
3.552427399	-0.963250524
3.552427399	-0.963250524
3.552427399	-0.963250524
3.552427399	-0.963250524

3.552427399	-0.963250524
3.552427399	-0.963250524
3.552427399	-0.963250524
3.552427399	-0.963250524
3.552427399	-0.963250524
3.552427399	-0.963250524
3.552427399	-0.963250524
3.552427399	-0.963250524
4.326759136	-0.932085986
3.552427399	-0.963250524
3.552427399	-0.963250524
3.489091065	-2.116535582
3.559447771	-2.492484414
3.559447771	-2.492484414
3.559447771	-2.492484414
3.559447771	-2.492484414
3.559447771	-2.492484414
3.559447771	-2.492484414
3.559447771	-2.492484414
3.559447771	-2.492484414
3.660350746	-1.567040593
3.660350746	-1.567040593
3.660350746	-1.567040593
3.660350746	-1.567040593
3.660350746	-1.567040593
3.660350746	-1.567040593
3.660350746	-1.567040593
3.660350746	-1.567040593
3.660350746	-1.567040593
3.660350746	-1.567040593
3.660350746	-1.567040593
3.660350746	-1.567040593
3.660350746	-1.567040593
3.660350746	-1.567040593
3.660350746	-1.567040593
3.660350746	-1.567040593
3.660350746	-1.567040593
3.660350746	-1.567040593
3.660350746	-1.567040593
3.660350746	-1.567040593
3.660350746	-1.567040593
5.631137462	1.553606257
5.631137462	1.553606257
5.631137462	1.553606257
5.631137462	1.553606257
5.631137462	1.553606257
5.631137462	1.553606257

5.631137462	1.553606257
5.631137462	1.553606257
2.901810596	-1.157463595
2.901810596	-1.157463595
2.901810596	-1.157463595
2.901810596	-1.157463595
2.901810596	-1.157463595
3.097786066	1.272978695
5.493789747	1.676718744
3.097786066	1.272978695
3.014833486	-0.91893312
3.014833486	-0.91893312
3.014833486	-0.91893312
3.014833486	-0.91893312
3.014833486	-0.91893312
3.014833486	-0.91893312
3.014833486	-0.91893312
3.014833486	-0.91893312
3.014833486	-0.91893312
3.77634837	-1.064313965
3.77634837	-1.064313965
3.77634837	-1.064313965
3.77634837	-1.064313965
3.77634837	-1.064313965
6.204615165	-0.939260842
3.77634837	-1.064313965
3.77634837	-1.064313965
2.687748744	-1.079173173
2.687748744	-1.079173173
2.687748744	-1.079173173
2.687748744	-1.079173173
2.687748744	-1.079173173
2.687748744	-1.079173173
6.204615165	-0.939260842
2.61288744	-1.142735555
3.624466955	-1.006941609
3.101117768	-1.654263005
3.101117768	-1.654263005
3.101117768	-1.654263005
3.101117768	-1.654263005
3.101117768	-1.654263005
3.535955873	-1.740335816
3.726831799	-1.784383523
3.726831799	-1.784383523
3.726831799	-1.784383523
3.726831799	-1.784383523
3.726831799	-1.784383523

3.726831799	-1.784383523
3.726831799	-1.784383523
3.726831799	-1.784383523
3.726831799	-1.784383523
3.726831799	-1.784383523
3.512684954	-1.904008087
3.148203557	1.184661154
3.819541506	1.429803402
3.819541506	1.429803402
3.819541506	1.429803402
3.819541506	1.429803402
3.819541506	1.429803402
3.819541506	1.429803402
3.819541506	1.429803402
3.819541506	1.429803402
3.819541506	1.429803402
3.819541506	1.429803402
3.819541506	1.429803402
3.819541506	1.429803402
3.819541506	1.429803402
3.819541506	1.429803402
3.819541506	1.429803402
3.819541506	1.429803402
3.819541506	1.429803402
3.819541506	1.429803402
3.819541506	1.429803402
3.819541506	1.429803402
3.819541506	1.429803402
3.819541506	1.429803402
3.819541506	1.429803402
3.819541506	1.429803402
3.819541506	1.429803402
3.819541506	1.429803402
3.819541506	1.429803402
3.819541506	1.429803402
3.819541506	1.429803402
3.819541506	1.429803402
3.819541506	1.429803402
3.819541506	1.429803402
3.819541506	1.429803402
3.819541506	1.429803402
3.819541506	1.429803402
9.794303073	4.144870298
9.794303073	4.144870298
9.794303073	4.144870298
9.794303073	4.144870298
9.794303073	4.144870298

9.794303073	4.144870298
4.385284901	1.74118651
4.385284901	1.74118651
4.385284901	1.74118651
4.385284901	1.74118651
4.385284901	1.74118651
4.385284901	1.74118651
4.385284901	1.74118651
8.570174569	3.5854313
4.407790888	1.145351386
4.407790888	1.145351386
4.407790888	1.145351386
11.90741806	6.779108666
4.994867375	1.847796518
5.493789747	1.676718744
5.493789747	1.676718744
5.493789747	1.676718744
5.493789747	1.676718744
5.493789747	1.676718744
5.493789747	1.676718744
5.493789747	1.676718744
5.493789747	1.676718744
5.493789747	1.676718744
5.493789747	1.676718744
6.204615165	-0.939260842
5.255709043	1.73508887
3.767169191	1.355072662
3.767169191	1.355072662
3.767169191	1.355072662
3.767169191	1.355072662
3.767169191	1.355072662
3.767169191	1.355072662
3.767169191	1.355072662
3.767169191	1.355072662
4.407790888	1.145351386
3.767169191	1.355072662
6.204615165	-0.939260842
3.767169191	1.355072662
3.767169191	1.355072662
3.554280234	1.348459062
3.251792291	1.091665988

Supplementary Table s8:The candidate genes in the selection sweep of white cashmere goa cashmere goats(YNBB,GZB)

Gene	Chr	Bin_start	Bin_end	Gene_Start	Gene_end	ZFst
**DLG1**	chr1	71190001	71340001	71247382	71513739	4.077795841
**MELTF**	chr1	71190001	71340001	71209817	71236775	4.077795841
**DSCR3**	chr1	149200001	149350001	149344317	149378412	7.13579866
**TTC3**	chr1	149200001	149350001	149193226	149320070	7.13579866
**MAP2**	chr2	38530001	38680001	38304673	38603165	4.585173759
**ABI2**	chr2	44180001	44330001	44270238	44375595	4.118960347
**RAPH1**	chr2	44180001	44330001	44165839	44269635	4.118960347
**ARHGAP15**	chr2	82980001	83130001	82654446	83365942	4.683102607
**TLK1**	chr2	110870001	111020001	110847293	111023597	3.682082085
**EIF2B3**	chr3	19630001	19780001	19591301	19712010	4.647552384
**HECTD3**	chr3	19630001	19780001	19724747	19732726	4.647552384
**UROD**	chr3	19630001	19780001	19735233	19738693	4.647552384
**ZSWIM5**	chr3	19630001	19780001	19738773	19797291	4.647552384
**MIER1**	chr3	42510001	42660001	42524645	42592531	6.301259252
**SLC35D1**	chr3	42510001	42660001	42606930	42654238	6.301259252
**WDR78**	chr3	42510001	42660001	42418935	42512922	6.301259252
**DLD**	chr4	71290001	71440001	71309867	71338255	5.85101858
**LAMB1**	chr4	71290001	71440001	71228918	71305310	5.85101858
**A4GALT**	chr5	112530001	112680001	112509158	112533604	4.411586744
**ARFGAP3**	chr5	112530001	112680001	112591791	112642935	4.411586744
**OC1086360[illegible]**	chr5	112530001	112680001	112564962	112571285	4.411586744
**PACSIN2**	chr5	112530001	112680001	112659474	112771854	4.411586744
**LIMCH1**	chr6	60700001	60850001	60794267	61154327	5.006638638
**UCHL1**	chr6	60700001	60850001	60715873	60727733	5.006638638
**KIT**	chr6	70650001	70800001	70711232	70793908	6.418703432
**OC10216853[illegible]**	chr6	85400001	85550001	85372781	85417336	3.79432632
**OC10217567[illegible]**	chr6	85400001	85550001	85418698	85462330	3.79432632
**OC10863319[illegible]**	chr6	85400001	85550001	85478280	85511521	3.79432632
**C6H4orf22**	chr6	95410001	95560001	95489192	96199327	7.88154538
**FGF5**	chr6	95410001	95560001	95418992	95440124	7.88154538
**OC10218583[illegible]**	chr7	670001	820001	784018	785066	3.542595109
**OC10863641[illegible]**	chr7	670001	820001	792204	793583	3.542595109
**CSF1R**	chr7	48970001	49120001	49005729	49037330	5.331314099
**HMGXB3**	chr7	48970001	49120001	49031724	49099624	5.331314099
**SLC26A2**	chr7	48970001	49120001	49103643	49133638	5.331314099
**TIGD6**	chr7	48970001	49120001	49099687	49105290	5.331314099
**NR3C1**	chr7	56030001	56180001	56055609	56173859	3.993747325
**AK3**	chr8	39630001	39780001	39635494	39656272	4.275663078
**CDC37L1**	chr8	39630001	39780001	39660101	39678648	4.275663078
**OC10863655[illegible]**	chr8	39630001	39780001	39713646	39733463	4.275663078
**PLPP6**	chr8	39630001	39780001	39700133	39703413	4.275663078
**SLC1A1**	chr8	39630001	39780001	39770036	39845247	4.275663078
**SPATA6L**	chr8	39630001	39780001	39734093	39756809	4.275663078

**LOC10217060[illegible]**	chr8	48300001	48450001	48103346	48358321	6.770352314
**ZFAND5**	chr8	48300001	48450001	48393269	48404753	6.770352314
**UBAP2**	chr8	75030001	75180001	75124772	75238885	4.991784611
**UBE2R2**	chr8	75030001	75180001	75023162	75123568	4.991784611
**SPACA1**	chr9	49090001	49240001	49173871	49199828	4.589731479
**WDR72**	chr10	46300001	46450001	46414060	46635609	7.339901688
**ATP5S**	chr10	59800001	59950001	59820875	59831656	8.16018786
**CDKL1**	chr10	59800001	59950001	59763696	59820944	8.16018786
**L2HGDH**	chr10	59800001	59950001	59831705	59883022	8.16018786
**SOS2**	chr10	59800001	59950001	59890326	59991899	8.16018786
**MYO9A**	chr10	82460001	82610001	82335575	82577050	3.77472812
**NR2E3**	chr10	82460001	82610001	82590036	82594865	3.77472812
**BIRC6**	chr11	14750001	14900001	14814336	15024500	5.654912281
**YIPF4**	chr11	14750001	14900001	14738946	14782522	5.654912281
**ATAD2B**	chr11	74680001	74830001	74782987	74910341	4.49953004
**FAM228B**	chr11	74680001	74830001	74637060	74685661	4.49953004
**FKBP1B**	chr11	74680001	74830001	74698136	74708853	4.49953004
**MFSD2B**	chr11	74680001	74830001	74729851	74742391	4.49953004
**SF3B6**	chr11	74680001	74830001	74685654	74693957	4.49953004
**TP53I3**	chr11	74680001	74830001	74678510	74685183	4.49953004
**UBXN2A**	chr11	74680001	74830001	74745868	74769527	4.49953004
**WDCP**	chr11	74680001	74830001	74711378	74726672	4.49953004
**MYCBP2**	chr12	33740001	33890001	33616605	33885679	3.765612678
**ARHGAP12**	chr13	32640001	32790001	32767221	32884670	5.861998544
**KIF5B**	chr13	32640001	32790001	32665942	32713201	5.861998544
**TFAP2C**	chr13	58580001	58730001	58657523	58667140	6.894591642
**FABP5**	chr14	37420001	37570001	37555681	37561958	3.87398285
**LOC10650286**	chr14	37420001	37570001	37565973	37578551	3.87398285
**ANGPTL7**	chr16	40820001	40970001	40944001	40950549	7.210068794
**MTOR**	chr16	40820001	40970001	40889820	41015393	7.210068794
**UBIAD1**	chr16	40820001	40970001	40869569	40881667	7.210068794
**LOC10217072[illegible]**	chr19	34260001	34410001	34401249	34407219	4.023455378
**MIR33B**	chr19	34260001	34410001	34294280	34294373	4.023455378
**RAI1**	chr19	34260001	34410001	34296405	34400733	4.023455378
**SREBF1**	chr19	34260001	34410001	34288792	34295549	4.023455378
**LOC10863849[illegible]**	chr21	830001	980001	828399	835280	4.44993375
**DUSP22**	chr23	50001	200001	111611	159290	8.136425561
**IRF4**	chr23	50001	200001	189741	204470	8.136425561
**LOC10218055**	chr23	50001	200001	103134	111587	8.136425561
**GDF10**	chr28	3470001	3620001	3460297	3473327	6.31818497
**GDF2**	chr28	3470001	3620001	3482208	3486651	6.31818497
**LOC10217750**	chr28	3470001	3620001	3521579	3535635	6.31818497
**LOC10863333[illegible]**	chr28	3470001	3620001	3549189	3563373	6.31818497
**RBP3**	chr28	3470001	3620001	3494829	3504269	6.31818497
**ZNF488**	chr28	3470001	3620001	3511701	3521545	6.31818497
**GPR174**	chrX1	2430001	2580001	2445642	2450303	7.203812286
**CHM**	chrX1	5380001	5530001	5218529	5465965	8.47547856

**ZDHHC15**	chrX1	7490001	7640001	7543624	7656270	10.43542284
**ERCC6L**	chrX1	9690001	9840001	9758235	9789589	8.272432095
**PIN4**	chrX1	9690001	9840001	9797484	9806164	8.272432095
**RPS4X**	chrX1	9690001	9840001	9710947	9715286	8.272432095
GBE1	chr1	28550001	28700001	27823980	28134705	3.602363403
TRNAS-GGA	chr1	29730001	29880001	30237432	30237503	4.845461054
LOC10863683[illegible]	chr1	50960001	51110001	51225661	51369420	5.766762895
BDH1	chr1	71190001	71340001	71702243	71739306	4.077795841
CEP19	chr1	71190001	71340001	70953940	70960688	4.077795841
FBXO45	chr1	71190001	71340001	70833297	70847080	4.077795841
LOC10650198[illegible]	chr1	71190001	71340001	71739387	71740951	4.077795841
LOC10863560[illegible]	chr1	71190001	71340001	71071562	71085250	4.077795841
NCBP2	chr1	71190001	71340001	71133340	71140105	4.077795841
NRROS	chr1	71190001	71340001	70890387	70916589	4.077795841
PAK2	chr1	71190001	71340001	70990569	71073583	4.077795841
PIGX	chr1	71190001	71340001	70957571	70985213	4.077795841
PIGZ	chr1	71190001	71340001	71144173	71175164	4.077795841
RNF168	chr1	71190001	71340001	70765125	70782692	4.077795841
SENP5	chr1	71190001	71340001	71085339	71133963	4.077795841
SMCO1	chr1	71190001	71340001	70784055	70790921	4.077795841
WDR53	chr1	71190001	71340001	70820545	70833184	4.077795841
APOD	chr1	71240001	71390001	71801594	71815214	4.007461921
LOC10650207[illegible]	chr1	71240001	71390001	71796710	71801248	4.007461921
ACAP2	chr1	71400001	71550001	71889654	72051523	3.570252188
PPP1R2	chr1	71400001	71550001	71839912	71861039	3.570252188
CCDC50	chr1	75940001	76090001	75702789	75778927	3.845600679
GMNC	chr1	75940001	76090001	76146215	76153710	3.845600679
IL1RAP	chr1	75940001	76090001	76348425	76502431	3.845600679
LOC10650209[illegible]	chr1	75940001	76090001	75696854	75700886	3.845600679
OSTN	chr1	75940001	76090001	75832686	75869245	3.845600679
TRNAY-GUA	chr1	75940001	76090001	75729168	75729239	3.845600679
UTS2B	chr1	75940001	76090001	75811187	75826115	3.845600679
CCDC39	chr1	86180001	86330001	86026741	86083878	5.039951434
DNAJC19	chr1	86180001	86330001	85751161	85756951	5.039951434
FXR1	chr1	86180001	86330001	85759719	85832648	5.039951434
LOC10217473[illegible]	chr1	86180001	86330001	86008316	86009287	5.039951434
PEX5L	chr1	86180001	86330001	86709401	86990660	5.039951434
TTC14	chr1	86180001	86330001	86086130	86095985	5.039951434
DYRK1A	chr1	149200001	149350001	149441823	149586835	7.13579866
HLCS	chr1	149200001	149350001	148908000	149101181	7.13579866
KCNJ6	chr1	149200001	149350001	149701472	149797540	7.13579866
LOC10650247[illegible]	chr1	149200001	149350001	148833940	148856302	7.13579866
LOC10863638[illegible]	chr1	149200001	149350001	149442265	149444359	7.13579866
LOC10863660[illegible]	chr1	149200001	149350001	148937056	148939730	7.13579866
PIGP	chr1	149200001	149350001	149181657	149193145	7.13579866
RIPPLY3	chr1	149200001	149350001	149137812	149154486	7.13579866
SIM2	chr1	149200001	149350001	148856421	148906415	7.13579866

LOC10863776[illegible]	chr2	38490001	38640001	38096326	38103292	3.757885269
LOC10219100[illegible]	chr2	38530001	38680001	38809543	38811982	4.585173759
UNC80	chr2	38530001	38680001	38057219	38272802	4.585173759
PTH2R	chr2	38620001	38770001	39142168	39237314	3.52863191
CARF	chr2	44180001	44330001	44630924	44682273	4.118960347
CD28	chr2	44180001	44330001	43971113	44007195	4.118960347
CTLA4	chr2	44180001	44330001	43834650	43898636	4.118960347
ICA1L	chr2	44180001	44330001	44715754	44771738	4.118960347
ICOS	chr2	44180001	44330001	43742923	43767248	4.118960347
LOC10217656[illegible]	chr2	44180001	44330001	44390930	44434022	4.118960347
NBEAL1	chr2	44180001	44330001	44445261	44596606	4.118960347
WDR12	chr2	44180001	44330001	44682367	44708633	4.118960347
FAM117B	chr2	44200001	44350001	44772823	44849910	3.721402786
BMPR2	chr2	44430001	44580001	44895151	45051641	3.520324427
GTDC1	chr2	82980001	83130001	83524315	83969888	4.683102607
KYNU	chr2	82980001	83130001	82455732	82573836	4.683102607
DCAF17	chr2	110870001	111020001	111270166	111296211	3.682082085
GAD1	chr2	110870001	111020001	110670201	110709366	3.682082085
GORASP2	chr2	110870001	111020001	110759622	110790395	3.682082085
LOC10086113[illegible]	chr2	110870001	111020001	111347663	111380487	3.682082085
LOC10650279[illegible]	chr2	110870001	111020001	110586596	110589425	3.682082085
METTL8	chr2	110870001	111020001	111185392	111270066	3.682082085
MYO3B	chr2	110870001	111020001	110071275	110525479	3.682082085
SP5	chr2	110870001	111020001	110577471	110649747	3.682082085
BEST4	chr3	19630001	19780001	19533282	19537923	4.647552384
BTBD19	chr3	19630001	19780001	19559512	19564955	4.647552384
C3H1orf228	chr3	19630001	19780001	19416581	19475118	4.647552384
HPDL	chr3	19630001	19780001	20038094	20039756	4.647552384
KIF2C	chr3	19630001	19780001	19497048	19515005	4.647552384
LOC10218365[illegible]	chr3	19630001	19780001	20200128	20203776	4.647552384
LOC10650197[illegible]	chr3	19630001	19780001	20011606	20034411	4.647552384
LOC10650197[illegible]	chr3	19630001	19780001	19351455	19376896	4.647552384
LOC10863564[illegible]	chr3	19630001	19780001	19492744	19496898	4.647552384
MMACHC	chr3	19630001	19780001	20203755	20209102	4.647552384
MUTYH	chr3	19630001	19780001	20040325	20052659	4.647552384
PLK3	chr3	19630001	19780001	19552113	19557154	4.647552384
PTCH2	chr3	19630001	19780001	19570536	19586439	4.647552384
RNF220	chr3	19630001	19780001	19134220	19396425	4.647552384
RPS8	chr3	19630001	19780001	19528475	19531122	4.647552384
TCTEX1D4	chr3	19630001	19780001	19557154	19559385	4.647552384
TESK2	chr3	19630001	19780001	20056153	20195032	4.647552384
TMEM53	chr3	19630001	19780001	19398934	19416381	4.647552384
TOE1	chr3	19630001	19780001	20052442	20056227	4.647552384
C3H1orf141	chr3	42510001	42660001	42689958	42739898	6.301259252
IL12RB2	chr3	42510001	42660001	42898126	42970364	6.301259252
IL23R	chr3	42510001	42660001	42777642	42839433	6.301259252

INSL5	chr3	42510001	42660001	42399363	42402829	6.301259252
LOC10217925[illegible]	chr3	42510001	42660001	42142579	42143074	6.301259252
SERBP1	chr3	42510001	42660001	42979045	42995109	6.301259252
SGIP1	chr3	42510001	42660001	42109904	42342473	6.301259252
TCTEX1D1	chr3	42510001	42660001	42350053	42378791	6.301259252
BCAP29	chr4	71290001	71440001	71630040	71677966	5.85101858
CBLL1	chr4	71290001	71440001	71501625	71518069	5.85101858
COG5	chr4	71290001	71440001	71695962	71978448	5.85101858
DUS4L	chr4	71290001	71440001	71679995	71695871	5.85101858
GPR22	chr4	71290001	71440001	71759431	71765488	5.85101858
LAMB4	chr4	71290001	71440001	71082894	71201291	5.85101858
NRCAM	chr4	71290001	71440001	70733249	71043018	5.85101858
SLC26A3	chr4	71290001	71440001	71471442	71497737	5.85101858
SLC26A4	chr4	71290001	71440001	71541010	71598634	5.85101858
HBP1	chr4	71570001	71720001	71979140	72010036	5.561603305
PRKAR2B	chr4	71570001	71720001	72019339	72129472	5.561603305
PIK3CG	chr4	71820001	71970001	72257537	72291605	5.430610264
AHR	chr4	94490001	94640001	94675280	94726287	4.224409435
SNX13	chr4	94490001	94640001	94080605	94228482	4.224409435
VSTM2A	chr4	119610001	119760001	119990280	120015338	6.90345848
**KITLG**	chr5	18400001	18550001	18044632	18151924	5.451347894
TRNAK-UUU	chr5	18400001	18550001	18931313	18931385	5.451347894
LOC10216900[illegible]	chr5	112490001	112640001	112064706	112073351	3.662421734
LOC10217733[illegible]	chr5	112490001	112640001	112046093	112073327	3.662421734
BIK	chr5	112530001	112680001	112857123	112876056	4.411586744
LOC10217242[illegible]	chr5	112530001	112680001	112386168	112391658	4.411586744
LOC10217364[illegible]	chr5	112530001	112680001	112448192	112473179	4.411586744
MCAT	chr5	112530001	112680001	112879941	112895630	4.411586744
NFAM1	chr5	112530001	112680001	112285233	112321727	4.411586744
POLDIP3	chr5	112530001	112680001	112423180	112445045	4.411586744
RRP7A	chr5	112530001	112680001	112412807	112419720	4.411586744
SCUBE1	chr5	112530001	112680001	112949603	113076465	4.411586744
SERHL2	chr5	112530001	112680001	112391941	112412844	4.411586744
TCF20	chr5	112530001	112680001	112076058	112171316	4.411586744
TSPO	chr5	112530001	112680001	112906645	112919429	4.411586744
TTLL12	chr5	112530001	112680001	112921455	112939130	4.411586744
TTLL1	chr5	112530001	112680001	112793919	112835018	4.411586744
APBB2	chr6	60700001	60850001	60285849	60665198	5.006638638
NSUN7	chr6	60700001	60850001	60236339	60284745	5.006638638
PHOX2B	chr6	60700001	60850001	61216690	61220345	5.006638638
KDR	chr6	70650001	70800001	71115093	71162022	6.418703432
LOC10650220[illegible]	chr6	70650001	70800001	71112345	71114702	6.418703432
LOC10863627[illegible]	chr6	70650001	70800001	70941596	70959636	6.418703432
PDGFRA	chr6	70650001	70800001	70285928	70334333	6.418703432
LAGE3	chr6	85400001	85550001	85840653	85841323	3.79432632
LOC10216852[illegible]	chr6	85400001	85550001	85677862	85734370	3.79432632
LOC10216984[illegible]	chr6	85400001	85550001	85878003	85901026	3.79432632

LOC10217196	chr6	85400001	85550001	85100990	85116854	3.79432632
LOC10217243	chr6	85400001	85550001	85137434	85152951	3.79432632
LOC10217308	chr6	85400001	85550001	85181313	85204348	3.79432632
LOC10217355	chr6	85400001	85550001	85246765	85270440	3.79432632
LOC10218314	chr6	85400001	85550001	85556243	85588921	3.79432632
LOC10650395	chr6	85400001	85550001	85319850	85353942	3.79432632
LOC10863324	chr6	85400001	85550001	85029807	85052189	3.79432632
SULT1B1	chr6	85400001	85550001	85775305	85812886	3.79432632
TRNAH-GUG	chr6	85400001	85550001	85872259	85872330	3.79432632
CSN1S1	chr6	85410001	85560001	85978463	85995270	3.621360812
ANTXR2	chr6	95410001	95560001	95056743	95230556	7.88154538
PRDM8	chr6	95410001	95560001	95336875	95356821	7.88154538
TRNAG-CCC	chr6	95410001	95560001	95616042	95616114	7.88154538
CAMK4	chr7	670001	820001	80527	352060	3.542595109
SLC25A46	chr7	670001	820001	913717	942491	3.542595109
TMEM232	chr7	670001	820001	970691	1229535	3.542595109
WDR36	chr7	670001	820001	469008	513272	3.542595109
ARHGEF37	chr7	48970001	49120001	49434451	49493265	5.331314099
ARSI	chr7	48970001	49120001	48771935	48779194	5.331314099
CAMK2A	chr7	48970001	49120001	48785985	48851816	5.331314099
CD74	chr7	48970001	49120001	48668848	48676702	5.331314099
CDX1	chr7	48970001	49120001	48887463	48904695	5.331314099
CSNK1A1	chr7	48970001	49120001	49514129	49562875	5.331314099
LOC10863634	chr7	48970001	49120001	48616701	48647168	5.331314099
LOC10863645	chr7	48970001	49120001	49397699	49417138	5.331314099
MIR378	chr7	48970001	49120001	49342624	49342733	5.331314099
NDST1	chr7	48970001	49120001	48527503	48611610	5.331314099
PDE6A	chr7	48970001	49120001	49146863	49224055	5.331314099
PDGFRB	chr7	48970001	49120001	48915000	48952725	5.331314099
PPARGC1B	chr7	48970001	49120001	49224063	49345243	5.331314099
RPS14	chr7	48970001	49120001	48647020	48650971	5.331314099
SLC6A7	chr7	48970001	49120001	48859670	48880389	5.331314099
TCOF1	chr7	48970001	49120001	48677826	48716462	5.331314099
ARHGAP26	chr7	56030001	56180001	56232839	56713995	3.993747325
CHSY3	chr7	86730001	86880001	86320405	86608841	4.602990304
HINT1	chr7	86730001	86880001	87299552	87306155	4.602990304
LYRM7	chr7	86740001	86890001	87312006	87337065	4.310861113
ACSL6	chr7	88170001	88320001	88024364	88089819	3.553575073
CSF2	chr7	88170001	88320001	88163476	88165829	3.553575073
FNIP1	chr7	88170001	88320001	87745848	87868406	3.553575073
IL5	chr7	88170001	88320001	88684078	88686626	3.553575073
IRF1	chr7	88170001	88320001	88630486	88637730	3.553575073
LOC10218911	chr7	88170001	88320001	88358467	88394609	3.553575073
LOC10219050	chr7	88170001	88320001	88148538	88150765	3.553575073
LOC10650231	chr7	88170001	88320001	88166964	88168905	3.553575073
LOC10863638	chr7	88170001	88320001	88557879	88636200	3.553575073
LOC10863638	chr7	88170001	88320001	88537122	88559426	3.553575073

MEIKIN	chr7	88170001	88320001	87869145	88020686	3.553575073
PDLIM4	chr7	88170001	88320001	88413050	88427472	3.553575073
RAD50	chr7	88170001	88320001	88702532	88815957	3.553575073
SLC22A4	chr7	88170001	88320001	88444408	88487123	3.553575073
SLC22A5	chr7	88170001	88320001	88511443	88537601	3.553575073
GLIS3	chr8	39630001	39780001	40049592	40544666	4.275663078
INSL6	chr8	39630001	39780001	39210866	39227365	4.275663078
**JAK2**	**chr8**	**39630001**	**39780001**	**39245187**	**39360411**	**4.275663078**
RCL1	chr8	39630001	39780001	39520371	39583801	4.275663078
ABHD17B	chr8	48300001	48450001	47856656	47901320	6.770352314
ALDH1A1	chr8	48300001	48450001	48842634	48898290	6.770352314
C8H9orf85	chr8	48300001	48450001	47901641	47965410	6.770352314
GDA	chr8	48300001	48450001	48163890	48299507	6.770352314
TMC1	chr8	48300001	48450001	48656137	48790796	6.770352314
TRNAC-ACA	chr8	48300001	48450001	48452463	48452534	6.770352314
TRNAS-AGA	chr8	48300001	48450001	48491343	48491414	6.770352314
LOC10863660	chr8	67610001	67760001	67546369	67550356	5.85940893
DNAJA1	chr8	75020001	75170001	74588289	74599055	4.706388417
AQP3	chr8	75030001	75180001	74981478	74987380	4.991784611
AQP7	chr8	75030001	75180001	74925841	74942904	4.991784611
B4GALT1	chr8	75030001	75180001	74661494	74711790	4.991784611
BAG1	chr8	75030001	75180001	74814221	74826505	4.991784611
C8H9orf24	chr8	75030001	75180001	75524268	75539047	4.991784611
CHMP5	chr8	75030001	75180001	74826490	74840085	4.991784611
DCAF12	chr8	75030001	75180001	75279762	75315413	4.991784611
DNAI1	chr8	75030001	75180001	75600294	75669575	4.991784611
FAM219A	chr8	75030001	75180001	75539143	75600107	4.991784611
KIAA1161	chr8	75030001	75180001	75514875	75522929	4.991784611
KIF24	chr8	75030001	75180001	75427275	75497830	4.991784611
LOC10216918	chr8	75030001	75180001	75382353	75382839	4.991784611
LOC10218885	chr8	75030001	75180001	74712045	74742654	4.991784611
NFX1	chr8	75030001	75180001	74850386	74915589	4.991784611
NOL6	chr8	75030001	75180001	74996129	75008583	4.991784611
NUDT2	chr8	75030001	75180001	75498047	75508885	4.991784611
SMU1	chr8	75030001	75180001	74602402	74634128	4.991784611
SPINK4	chr8	75030001	75180001	74799650	74807172	4.991784611
UBAP1	chr8	75030001	75180001	75361372	75427272	4.991784611
AKIRIN2	chr9	49090001	49240001	49535238	49559370	4.589731479
CNR1	chr9	49090001	49240001	49058598	49089157	4.589731479
ORC3	chr9	49090001	49240001	49563207	49636451	4.589731479
RARS2	chr9	49090001	49240001	49636516	49709248	4.589731479
TRNAE-UUC	chr9	49090001	49240001	49452430	49452502	4.589731479
EPM2A	chr9	69560001	69710001	69975816	70097860	4.546992488
UTRN	chr9	69560001	69710001	68700661	69257114	4.546992488
FBXO30	chr9	69570001	69720001	70139832	70157217	4.46087228
UNC13C	chr10	46300001	46450001	46857881	47536542	7.339901688
ARF6	chr10	59800001	59950001	60176242	60180754	8.16018786

ATL1	chr10	59800001	59950001	59534244	59620451	8.16018786
DNAAF2	chr10	59800001	59950001	60362975	60373391	8.16018786
KLHDC1	chr10	59800001	59950001	60292061	60331139	8.16018786
KLHDC2	chr10	59800001	59950001	60273935	60288929	8.16018786
LOC10863699	chr10	59800001	59950001	60096105	60123543	8.16018786
MAP4K5	chr10	59800001	59950001	59620555	59737329	8.16018786
MGAT2	chr10	59800001	59950001	60373610	60376239	8.16018786
NEMF	chr10	59800001	59950001	60219279	60273568	8.16018786
NIN	chr10	59800001	59950001	59358669	59461224	8.16018786
POLE2	chr10	59800001	59950001	60333137	60362906	8.16018786
SAV1	chr10	59800001	59950001	59504372	59533641	8.16018786
VCPKMT	chr10	59800001	59950001	59992012	59998534	8.16018786
LRR1	chr10	59810001	59960001	60380171	60390404	7.436483937
RPL36AL	chr10	59810001	59960001	60376270	60377558	7.436483937
RPS29	chr10	59830001	59980001	60400653	60402483	6.701406427
ARIH1	chr10	82460001	82610001	81939607	82041082	3.77472812
CELF6	chr10	82460001	82610001	82164246	82194306	3.77472812
GRAMD2	chr10	82460001	82610001	82266642	82301212	3.77472812
HEXA	chr10	82460001	82610001	82112311	82143840	3.77472812
LOC10217412	chr10	82460001	82610001	82079094	82093970	3.77472812
LOC10217439	chr10	82460001	82610001	82063046	82067055	3.77472812
PARP6	chr10	82460001	82610001	82205875	82232562	3.77472812
PKM	chr10	82460001	82610001	82238790	82265545	3.77472812
SENP8	chr10	82460001	82610001	82315289	82335360	3.77472812
THSD4	chr10	82460001	82610001	82620551	83285979	3.77472812
DPY30	chr11	14750001	14900001	14524697	14538756	5.654912281
LOC10863710	chr11	14750001	14900001	15031517	15032456	5.654912281
LTBP1	chr11	14750001	14900001	15324388	15780520	5.654912281
MEMO1	chr11	14750001	14900001	14404200	14515722	5.654912281
NLRC4	chr11	14750001	14900001	14669557	14716302	5.654912281
SLC30A6	chr11	14750001	14900001	14620539	14666163	5.654912281
SPAST	chr11	14750001	14900001	14556017	14608600	5.654912281
TTC27	chr11	14750001	14900001	15041424	15219539	5.654912281
FAM228A	chr11	74680001	74830001	74624305	74636502	4.49953004
ITSN2	chr11	74680001	74830001	74499640	74622540	4.49953004
KLHL29	chr11	74680001	74830001	74938480	75270624	4.49953004
NCOA1	chr11	74680001	74830001	74153980	74372125	4.49953004
PFN4	chr11	74680001	74830001	74669150	74674491	4.49953004
ACOD1	chr12	33740001	33890001	33959349	33966931	3.765612678
CLN5	chr12	33740001	33890001	33920524	33931904	3.765612678
FBXL3	chr12	33740001	33890001	33896229	33919253	3.765612678
KCTD12	chr12	33740001	33890001	34023639	34029762	3.765612678
LOC10863727	chr12	33740001	33890001	33515996	33519521	3.765612678
SCEL	chr12	33740001	33890001	33247740	33372142	3.765612678
CACNB2	chr13	32640001	32790001	31915665	32336714	5.861998544
EPC1	chr13	32640001	32790001	32467923	32564799	5.861998544
NSUN6	chr13	32640001	32790001	32338249	32416247	5.861998544

ZEB1	chr13	32640001	32790001	33123676	33323855	5.861998544
ADRA1D	chr13	49740001	49890001	50264597	50293882	6.179858141
HAO1	chr13	49740001	49890001	50024601	50086221	6.179858141
SMOX	chr13	49740001	49890001	50305402	50340974	6.179858141
SPO11	chr13	58540001	58690001	58104357	58119153	3.523494115
AURKA	chr13	58580001	58730001	58845806	58862526	6.894591642
BMP7	chr13	58580001	58730001	58174093	58260125	6.894591642
CASS4	chr13	58580001	58730001	58791766	58829365	6.894591642
CSTF1	chr13	58580001	58730001	58833053	58845722	6.894591642
FAM210B	chr13	58580001	58730001	58864336	58875132	6.894591642
FKBP1A	chr13	58580001	58730001	59038273	59061754	6.894591642
GCNT7	chr13	58580001	58730001	58749848	58761271	6.894591642
LOC10217180(	chr13	58580001	58730001	58963406	58973585	6.894591642
LOC10217207	chr13	58580001	58730001	58980392	58991467	6.894591642
LOC10217846	chr13	58580001	58730001	58731400	58732833	6.894591642
MC3R	chr13	58580001	58730001	58935095	58936185	6.894591642
NSFL1C	chr13	58580001	58730001	59000052	59021299	6.894591642
RTFDC1	chr13	58580001	58730001	58735902	58783189	6.894591642
SDCBP2	chr13	58580001	58730001	59090093	59109110	6.894591642
SNPH	chr13	58580001	58730001	59109662	59153612	6.894591642
C13H20orf202	chr13	58770001	58920001	59230537	59243636	5.191682105
LOC10217320	chr13	58770001	58920001	59204147	59213351	5.191682105
PSMF1	chr13	58770001	58920001	59259919	59302429	5.191682105
RAD21L1	chr13	58770001	58920001	59165671	59201265	5.191682105
TMEM74B	chr13	58770001	58920001	59244717	59249746	5.191682105
FABP9	chr14	37420001	37570001	37386749	37389927	3.87398285
LOC10086127	chr14	37420001	37570001	37370685	37375203	3.87398285
LOC10218033(	chr14	37420001	37570001	37401185	37406098	3.87398285
LOC10218113(	chr14	37420001	37570001	37261248	37304883	3.87398285
LOC10218398	chr14	37420001	37570001	37319293	37328976	3.87398285
MAL2	chr14	37420001	37570001	37054286	37083765	3.87398285
NOV	chr14	37420001	37570001	37205654	37213987	3.87398285
PAG1	chr14	37420001	37570001	37722835	37882011	3.87398285
ZNF704	chr14	37420001	37570001	37966963	38224513	3.87398285
ASPH	chr14	54670001	54820001	54919151	55109204	5.115941071
CLVS1	chr14	54670001	54820001	55108124	55272244	5.115941071
NKAIN3	chr14	54670001	54820001	54129546	54655604	5.115941071
CLCN6	chr16	40780001	40930001	40326558	40359834	5.273845382
MTHFR	chr16	40790001	40940001	40359958	40374676	6.15818825
AGTRAP	chr16	40820001	40970001	40414580	40434456	7.210068794
C16H1orf127	chr16	40820001	40970001	41145834	41161708	7.210068794
C16H1orf167	chr16	40820001	40970001	40375150	40397444	7.210068794
CASZ1	chr16	40820001	40970001	41300131	41455846	7.210068794
DISP3	chr16	40820001	40970001	40642918	40700187	7.210068794
DRAXIN	chr16	40820001	40970001	40450639	40462803	7.210068794
EXOSC10	chr16	40820001	40970001	41023287	41045517	7.210068794
FBXO2	chr16	40820001	40970001	40518717	40525066	7.210068794

LOC102183714	chr16	40820001	40970001	40489918	40510465	7.210068794
LOC102184360	chr16	40820001	40970001	40513567	40518656	7.210068794
LOC106502956	chr16	40820001	40970001	40793627	40798534	7.210068794
MAD2L2	chr16	40820001	40970001	40484534	40489828	7.210068794
MASP2	chr16	40820001	40970001	41080217	41096199	7.210068794
SRM	chr16	40820001	40970001	41062640	41066649	7.210068794
TARDBP	chr16	40820001	40970001	41097063	41103700	7.210068794
CPE	chr17	69480001	69630001	69249530	69400252	8.940987597
KLHL2	chr17	69480001	69630001	69018320	69180171	8.940987597
MSMO1	chr17	69480001	69630001	69190106	69207149	8.940987597
TLL1	chr17	69790001	69940001	70288534	70463775	3.940318406
ALKBH5	chr19	34260001	34410001	34083409	34101930	4.023455378
ATPAF2	chr19	34260001	34410001	34184989	34197673	4.023455378
COPS3	chr19	34260001	34410001	34532194	34550031	4.023455378
DRC3	chr19	34260001	34410001	34198124	34214173	4.023455378
DRG2	chr19	34260001	34410001	34158391	34167225	4.023455378
EPN2	chr19	34260001	34410001	33782295	33835695	4.023455378
FAM83G	chr19	34260001	34410001	33895436	33924274	4.023455378
FLCN	chr19	34260001	34410001	34562388	34578706	4.023455378
FLII	chr19	34260001	34410001	34048218	34060204	4.023455378
GID4	chr19	34260001	34410001	34170501	34184804	4.023455378
GRAP	chr19	34260001	34410001	33858368	33879666	4.023455378
LLGL1	chr19	34260001	34410001	34060165	34075010	4.023455378
LOC102173120	chr19	34260001	34410001	34813392	34819216	4.023455378
LOC106503231	chr19	34260001	34410001	34702981	34708025	4.023455378
MED9	chr19	34260001	34410001	34469592	34475211	4.023455378
MIEF2	chr19	34260001	34410001	34043363	34048134	4.023455378
MPRIP	chr19	34260001	34410001	34594212	34689199	4.023455378
MYO15A	chr19	34260001	34410001	34106198	34152663	4.023455378
NT5M	chr19	34260001	34410001	34515432	34528305	4.023455378
PEMT	chr19	34260001	34410001	34427348	34463596	4.023455378
PLD6	chr19	34260001	34410001	34584886	34587673	4.023455378
PRPSAP2	chr19	34260001	34410001	33938685	33961376	4.023455378
RASD1	chr19	34260001	34410001	34466190	34468560	4.023455378
SHMT1	chr19	34260001	34410001	33985506	34012237	4.023455378
SLC5A10	chr19	34260001	34410001	33879678	33935296	4.023455378
SMCR8	chr19	34260001	34410001	34012105	34021030	4.023455378
TNFRSF13B	chr19	34260001	34410001	34749523	34772452	4.023455378
TOP3A	chr19	34260001	34410001	34021122	34041435	4.023455378
USP22	chr19	34260001	34410001	34778451	34797475	4.023455378
MKRN3	chr21	700001	850001	368224	369765	4.082146393
LOC102175755	chr21	830001	980001	1128292	1135469	4.44993375
LOC102188534	chr21	830001	980001	1135590	1283333	4.44993375
LOC106503337	chr21	830001	980001	1346787	1351080	4.44993375
MAGEL2	chr21	830001	980001	418902	423047	4.44993375
NDN	chr21	830001	980001	463679	465342	4.44993375
SNRPN	chr21	830001	980001	1109040	1115092	4.44993375

SNURF	chr21	830001	980001	1091977	1113163	4.44993375
ADAMTSL3	chr21	23100001	23250001	23559613	23931139	6.221830607
EFL1	chr21	23100001	23250001	23389905	23501720	6.221830607
LOC10218457	chr21	23100001	23250001	22654945	22773169	6.221830607
LOC10863845	chr21	23100001	23250001	22816979	22829042	6.221830607
LOC10863847	chr21	23100001	23250001	22879566	22880445	6.221830607
LOC10863851	chr21	23100001	23250001	23501768	23503984	6.221830607
LOC10863853	chr21	23100001	23250001	22853361	22858753	6.221830607
MEX3B	chr21	23100001	23250001	23314666	23317865	6.221830607
SAXO2	chr21	23100001	23250001	23508086	23519714	6.221830607
BNC1	chr21	23630001	23780001	24191923	24220410	4.600504274
SH3GL3	chr21	23630001	23780001	23947019	24053896	4.600504274
EXOC2	chr23	50001	200001	237311	359937	8.136425561
LOC10863344	chr23	50001	200001	567430	569485	8.136425561
FOXQ1	chr23	70001	220001	641272	643823	5.461001975
FOXF2	chr23	120001	270001	691668	696627	4.410447314
FOXC1	chr23	270001	420001	780584	784092	4.16598774
GMDS	chr23	270001	420001	792203	1217301	4.16598774
LOC10217961	chr23	270001	420001	765292	769039	4.16598774
MAPK8	chr28	3460001	3610001	2941175	3039589	5.084099176
ANTXRL	chr28	3470001	3620001	3727694	3772919	6.31818497
FAM35A	chr28	3470001	3620001	3966919	4070554	6.31818497
FRMPD2	chr28	3470001	3620001	3087917	3155034	6.31818497
GPRIN2	chr28	3470001	3620001	3917031	3935587	6.31818497
LOC10217243	chr28	3470001	3620001	3836198	3843754	6.31818497
LOC10217462	chr28	3470001	3620001	3937188	3948104	6.31818497
LOC10217649	chr28	3470001	3620001	3784690	3801075	6.31818497
LOC10218847	chr28	3470001	3620001	3948193	3957398	6.31818497
LOC10863413	chr28	3470001	3620001	3167684	3194936	6.31818497
PTPN20	chr28	3470001	3620001	3207633	3233696	6.31818497
TRNAA-AGC	chr28	3470001	3620001	3823011	3823082	6.31818497
ATRX	chrX1	2430001	2580001	2844617	3079823	7.203812286
CYSLTR1	chrX1	2430001	2580001	2639169	2709712	7.203812286
LOC10217871	chrX1	2430001	2580001	2763653	2793750	7.203812286
LOC10218404	chrX1	2430001	2580001	2403912	2428110	7.203812286
LOC10218423	chrX1	2430001	2580001	2184806	2204285	7.203812286
LPAR4	chrX1	2430001	2580001	2087559	2099113	7.203812286
CYLC1	chrX1	5380001	5530001	5770546	5816316	8.47547856
MIR361	chrX1	5380001	5530001	5296302	5296397	8.47547856
TRNAW-CCA	chrX1	5380001	5530001	5909913	5909985	8.47547856
ABCB7	chrX1	7490001	7640001	7666353	7809522	10.43542284
HDX	chrX1	7490001	7640001	7167355	7192928	10.43542284
KIAA2022	chrX1	7490001	7640001	7875317	8084555	10.43542284
MAGEE2	chrX1	7490001	7640001	7358153	7360444	10.43542284
RPS6KA6	chrX1	7490001	7640001	7095036	7148417	10.43542284
TRNAC-GCA	chrX1	7490001	7640001	8014720	8014791	10.43542284
NAP1L2	chrX1	9680001	9830001	9253597	9256075	8.272432095

CITED1	chrX1	9690001	9840001	9674906	9680321	8.272432095
FGF16	chrX1	9690001	9840001	10034151	10043086	8.272432095
HDAC8	chrX1	9690001	9840001	9335339	9343047	8.272432095
LOC10863431[illegible]	chrX1	9690001	9840001	10190999	10195135	8.272432095
LOC10863434[illegible]	chrX1	9690001	9840001	10068848	10075883	8.272432095
LOC10863435[illegible]	chrX1	9690001	9840001	9621510	9654343	8.272432095
NHSL2	chrX1	9690001	9840001	9845027	9866306	8.272432095
PHKA1	chrX1	9690001	9840001	9352165	9527318	8.272432095
RGAG4	chrX1	9690001	9840001	9866637	9871155	8.272432095

ıts(IMC,LNC) and black

log 2 (θπ ratio)
1.519133746
1.519133746
1.220067334
1.220067334
1.44606391
0.831762887
0.831762887
2.895711311
0.896106212
5.444823593
5.444823593
5.444823593
5.444823593
0.695709022
0.695709022
0.695709022
0.689512446
0.689512446
1.438761099
1.438761099
1.438761099
1.438761099
0.979381613
0.979381613
1.269078511
0.983364532
0.983364532
0.983364532
-5.892952164
-5.892952164
1.869047665
1.869047665
1.689495915
1.689495915
1.689495915
1.689495915
0.465154886
1.256629115
1.256629115
1.256629115
1.256629115
1.256629115
1.256629115

0.799970839
0.799970839
0.696009002
0.696009002
0.451627735
1.225058174
2.062164527
2.062164527
2.062164527
2.062164527
0.82252203
0.82252203
3.241322629
3.241322629
0.452310108
0.452310108
0.452310108
0.452310108
0.452310108
0.452310108
0.452310108
0.452310108
0.581443334
0.544320516
0.544320516
1.848139163
0.868863963
0.868863963
0.420092778
0.420092778
0.420092778
0.49551399
0.49551399
0.49551399
0.49551399
0.533246485
1.59447982
1.59447982
1.59447982
2.913213984
2.913213984
2.913213984
2.913213984
2.913213984
2.913213984
0.747097385
2.522883125

0.753121315
0.901394004
0.901394004
0.901394004
0.540637055
0.676332558
1.904745972
1.519133746
1.519133746
1.519133746
1.519133746
1.519133746
1.519133746
1.519133746
1.519133746
1.519133746
1.519133746
1.519133746
1.519133746
1.519133746
1.519133746
2.422088645
2.422088645
2.33243566
2.33243566
0.41356136
0.41356136
0.41356136
0.41356136
0.41356136
0.41356136
0.41356136
0.628265047
0.628265047
0.628265047
0.628265047
0.628265047
0.628265047
1.220067334
1.220067334
1.220067334
1.220067334
1.220067334
1.220067334
1.220067334
1.220067334
1.220067334

1.703640266
1.542400338
1.44606391
1.44606391
0.445404986
0.831762887
0.831762887
0.831762887
0.831762887
0.831762887
0.831762887
0.831762887
0.831762887
1.125952097
0.695849086
2.895711311
2.895711311
0.896106212
0.896106212
0.896106212
0.896106212
0.896106212
0.896106212
0.896106212
0.896106212
5.444823593
5.444823593
5.444823593
5.444823593
5.444823593
5.444823593
5.444823593
5.444823593
5.444823593
5.444823593
5.444823593
5.444823593
5.444823593
5.444823593
5.444823593
5.444823593
5.444823593
5.444823593
5.444823593
0.695709022
0.695709022
0.695709022

0.695709022
0.695709022
0.695709022
0.695709022
0.695709022
0.689512446
0.689512446
0.689512446
0.689512446
0.689512446
0.689512446
0.689512446
0.689512446
0.689512446
1.349252948
1.349252948
0.454013285
0.741763151
0.741763151
0.588026912
2.824501837
2.824501837
0.490304594
0.490304594
1.438761099
1.438761099
1.438761099
1.438761099
1.438761099
1.438761099
1.438761099
1.438761099
1.438761099
1.438761099
1.438761099
1.438761099
1.438761099
0.979381613
0.979381613
0.979381613
1.269078511
1.269078511
1.269078511
1.269078511
0.983364532
0.983364532
0.983364532

0.983364532
0.983364532
0.983364532
0.983364532
0.983364532
0.983364532
0.983364532
0.983364532
0.983364532
1.367069834
-5.892952164
-5.892952164
-5.892952164
1.869047665
1.869047665
1.869047665
1.869047665
1.689495915
1.689495915
1.689495915
1.689495915
1.689495915
1.689495915
1.689495915
1.689495915
1.689495915
1.689495915
1.689495915
1.689495915
1.689495915
1.689495915
1.689495915
1.689495915
0.465154886
1.300898854
1.300898854
1.329492762
0.639018307
0.639018307
0.639018307
0.639018307
0.639018307
0.639018307
0.639018307
0.639018307
0.639018307
0.639018307

0.639018307
0.639018307
0.639018307
0.639018307
0.639018307
1.256629115
1.256629115
1.256629115
1.256629115
0.799970839
0.799970839
0.799970839
0.799970839
0.799970839
0.799970839
0.799970839
1.043592334
0.652970977
0.696009002
0.696009002
0.696009002
0.696009002
0.696009002
0.696009002
0.696009002
0.696009002
0.696009002
0.696009002
0.696009002
0.696009002
0.696009002
0.696009002
0.696009002
0.696009002
0.696009002
0.696009002
0.696009002
0.451627735
0.451627735
0.451627735
0.451627735
0.451627735
2.052384367
2.052384367
2.213053386
1.225058174
2.062164527

2.062164527
2.062164527
2.062164527
2.062164527
2.062164527
2.062164527
2.062164527
2.062164527
2.062164527
2.062164527
2.062164527
2.062164527
1.554576324
1.554576324
1.208801374
0.82252203
0.82252203
0.82252203
0.82252203
0.82252203
0.82252203
0.82252203
0.82252203
0.82252203
0.82252203
3.241322629
3.241322629
3.241322629
3.241322629
3.241322629
3.241322629
3.241322629
3.241322629
0.452310108
0.452310108
0.452310108
0.452310108
0.452310108
0.581443334
0.581443334
0.581443334
0.581443334
0.581443334
0.581443334
0.544320516
0.544320516
0.544320516

0.544320516
0.620295215
0.620295215
0.620295215
0.507530025
1.848139163
1.848139163
1.848139163
1.848139163
1.848139163
1.848139163
1.848139163
1.848139163
1.848139163
1.848139163
1.848139163
1.848139163
1.848139163
1.848139163
1.848139163
1.502285353
1.502285353
1.502285353
1.502285353
1.502285353
0.868863963
0.868863963
0.868863963
0.868863963
0.868863963
0.868863963
0.868863963
0.868863963
0.868863963
0.983812908
0.983812908
0.983812908
0.478324923
0.551944071
0.420092778
0.420092778
0.420092778
0.420092778
0.420092778
0.420092778
0.420092778
0.420092778

0.420092778
0.420092778
0.420092778
0.420092778
0.420092778
0.420092778
0.420092778
1.096762359
1.096762359
1.096762359
0.51668781
0.49551399
0.49551399
0.49551399
0.49551399
0.49551399
0.49551399
0.49551399
0.49551399
0.49551399
0.49551399
0.49551399
0.49551399
0.49551399
0.49551399
0.49551399
0.49551399
0.49551399
0.49551399
0.49551399
0.49551399
0.49551399
0.49551399
0.49551399
0.49551399
0.49551399
0.49551399
0.49551399
0.49551399
0.49551399
0.824422412
0.533246485
0.533246485
0.533246485
0.533246485
0.533246485
0.533246485

0.533246485
0.479996211
0.479996211
0.479996211
0.479996211
0.479996211
0.479996211
0.479996211
0.479996211
0.479996211
0.428499799
0.428499799
1.59447982
1.59447982
1.137823382
1.014177925
0.775201697
0.775201697
0.775201697
2.742224133
2.913213984
2.913213984
2.913213984
2.913213984
2.913213984
2.913213984
2.913213984
2.913213984
2.913213984
2.913213984
2.913213984
0.747097385
0.747097385
0.747097385
0.747097385
0.747097385
0.747097385
2.522883125
2.522883125
2.522883125
0.753121315
0.753121315
0.753121315
0.753121315
0.753121315
0.753121315
0.772385717

0.901394004
0.901394004
0.901394004
0.901394004
0.901394004
0.901394004
0.901394004
0.901394004
0.901394004

ary Table s9: Variant analysis of DENND1A an

#GeneName	GeneId	TranscriptIc	BioType	nts_impact_	nts_impact_	impact_MC	impact_M	ct_3_prime_
DENND1A	gene12218	_01805574	otein_codi	0	0	0	645	0
DENND1A	gene12218	_01805574	otein_codi	0	0	0	645	0
DENND1A	gene12218	_01805574	otein_codi	0	0	0	645	0
DENND1A	gene12218	_01805574	otein_codi	0	0	0	645	0
LHX2	gene12219	_0013142	otein_codi	0	0	0	15	0

R_prematu	ct_5_prime_	downstrea	t_initiator_	effect_intro	fect_misse	coding_tra	on_coding_t	splice_ac
0	0	3	0	0	0	0	635	0
0	0	3	0	0	0	0	635	0
0	0	3	0	0	0	0	635	0
0	0	3	0	0	0	0	635	0
0	0	1	0	0	0	0	11	0

ect_splice_d	ct_splice_re	s_effect_sta	_effect_stop	s_effect_sto	ct_stop_ret	ct_synony	rt_upstream_gene_varia
0	0	0	0	0	0	0	7
0	0	0	0	0	0	0	7
0	0	0	0	0	0	0	7
0	0	0	0	0	0	0	7
0	0	0	0	0	0	0	3

ant

Supplementary Table s10: Cashmere yield and Genotype

DNA ID	Yield(kg)	FGF5	LXH2	Earmark	Age(years)	Gender
HXR2061	0.26	del/del	del/del	ER00858	3	F
HXR2169	0.39	del/del	del/del	ER17886	3	F
HXR2802	0.2	del/del	del/del	ER18223	3	F
HXR2315	0.21	del/del	del/del	ER25253	3	F
HXR2227	0.3	del/del	del/del	ER32517	3	F
HXR2791	0.35	del/del	del/del	ER18225	2	F
HXR2779	0.18	del/del	del/del	ER05034	3	F
HXR2798	0.28	del/del	del/del	ER18265	3	F
HXR2190	0.31	del/del	del/del	ER17955	2	F
HXR1926	0.4	del/del	del/del	ER17927	3	F
HXR2788	0.23	del/del	del/del	ER18275	2	F
HXR2063	0.23	del/del	del/del	ER04686	3	F
HXR2219	0.29	del/del	del/del	ER32528	3	F
HXR2163	0.37	del/del	del/del	ER17890	3	F
HXR2346	0.5	del/del	del/del	ER23963	3	F
HXR2482	0.31	del/del	del/del	ER39431	2	F
HXR2827	0.21	del/del	del/del	ER05005	3	F
HXR1957	0.26	del/del	del/del	ER17832	3	F
HXR2787	0.39	del/del	del/del	ER18233	3	F
HXR2444	0.29	del/del	del/del	ER17660	2	F
HXR2476	0.38	del/del	del/del	ER17690	3	F
HXR1454	0.23	del/del	del/del	ER01832	3	F
HXR2082	0.23	del/del	del/del	ER19136	3	F
HXR2090	0.48	del/del	del/del	ER18568	3	F
HXR3125	0.2	del/del	del/del	ER28381	2	F
HXR1067	0.39	del/del	del/del	ER00715	3	F
HXR0736	0.41	del/del	del/del	ER00734	3	F
HXR0550	0.31	del/del	del/del	ER04035	3	F
HXR1927	0.36	del/del	del/del	ER17834	3	F
HXR1821	0.38	del/del	del/del	ER18152	3	F
HXR0716	0.41	del/del	del/del	ER41142	3	F
HXR1717	0.45	del/del	del/del	ER29449	3	F
HXR1933	0.5	del/del	del/del	ER17940	3	F
HXR0690	0.51	del/del	del/del	ER42534	2	F
HXR3139	0.19	del/del	del/del	ER28609	2	F
HXR3214	0.21	del/del	del/del	ER36241	2	F
HXR1143	0.24	del/del	del/del	ER05392	2	F
HXR1477	0.24	del/del	del/del	ER01831	3	F
HXR0960	0.28	del/del	del/del	ER29103	2	F
HXR1798	0.31	del/del	del/del	ER18148	3	F
HXR1130	0.33	del/del	del/del	ER05393	2	F
HXR1068	0.42	del/del	del/del	ER00748	3	F
HXR0979	0.45	del/del	del/del	ER29638	3	F
HXR1189	0.33	del/del	del/del	ER41103	2	F
HXR1392	0.34	del/del	del/del	ER09249	2	F
HXR1237	0.29	del/del	del/del	ER04315	3	F
HXR0954	0.38	del/del	del/del	ER29756	3	F
HXR1398	0.44	del/del	del/del	ER10903	3	F

HXR0825	0.31	del/del	del/del	ER02032	3	F
HXR1812	0.34	del/del	del/del	ER18140	2	F
HXR1046	0.32	del/del	del/del	ER09390	2	F
HXR1833	0.32	del/del	del/del	ER17474	2	F
HXR0879	0.4	del/del	del/del	ER12253	3	F
HXR1581	0.42	del/del	del/del	ER00744	3	F
HXR0900	0.22	del/del	del/del	ER12036	3	F
HXR0889	0.26	del/del	del/del	ER12335	2	F
HXR1830	0.26	del/del	del/del	ER17475	2	F
HXR1841	0.33	del/del	del/del	ER17456	2	F
HXR0850	0.38	del/del	del/del	ER05260	3	F
HXR1100	0.43	del/del	del/del	ER12119	3	F
HXR0888	0.45	del/del	del/del	ER12147	3	F
HXR1121	0.3	del/del	del/del	ER05400	2	F
HXR1129	0.42	del/del	del/del	ER05311	2	F
HXR0186	0.13	del/del	del/del	ER24960	2	F
HXR0370	0.59	del/del	del/del	ER41130	3	F
HXR2781	0.15	del/del	del/del	ER05069	3	F
HXR0981	0.45	del/del	del/del	ER29622	3	F
HXR2752	0.15	del/del	del/del	ER05003	3	F
HXR1321	0.53	del/del	del/del	ER12349	3	F
HXR1630	0.34	del/del	del/del	ER32088	3	F
HXR1319	0.87	del/del	del/del	ER12236	3	F
HXR0775	0.85	del/del	del/del	ER12273	2	F
HXR1092	0.76	del/del	del/del	ER12159	2	F
HXR1583	0.76	del/del	del/del	ER00738	2	F
HXR1574	0.71	del/del	del/del	ER00751	3	F
HXR0765	0.66	del/del	del/del	ER12096	3	F
HXR0909	0.62	del/del	del/del	ER12346	2	F
HXR0882	0.62	del/del	del/del	ER12243	3	F
HXR0750	0.62	del/del	del/del	ER12124	3	F
HXR1802	0.61	del/del	del/del	ER18824	2	F
HXR0971	0.59	del/del	del/del	ER29612	2	F
HXR3135	0.58	del/del	del/del	ER28367	3	F
HXR0880	0.58	del/del	del/del	ER12050	3	F
HXR0864	0.57	del/del	del/del	ER12116	2	F
HXR0770	0.57	del/del	del/del	ER12098	3	F
HXR1559	0.56	del/del	del/del	ER00703	3	F
HXR0970	0.56	del/del	del/del	ER29619	2	F
HXR0792	0.56	del/del	del/del	ER02028	3	F
HXR1387	0.56	del/del	del/del	ER09251	3	F
HXR1199	0.56	del/del	del/del	ER41095	2	F
HXR0777	0.56	del/del	del/del	ER12265	2	F
HXR0881	0.55	del/del	del/del	ER12274	3	F
HXR1313	0.54	del/del	del/del	ER12169	3	F
HXR1318	0.53	del/del	del/del	ER12302	2	F
HXR0836	0.52	del/del	del/del	ER12358	2	F
HXR1796	0.52	del/del	del/del	ER18581	2	F
HXR0949	0.51	del/del	del/del	ER29610	3	F
HXR1158	0.19	del/del	del/del	ER41707	N	N

HXR1928	0.16	del/del	del/del	ER17930	3	F
HXR2815	0.14	del/del	del/del	ER05058	3	F
HXR2776	0.14	del/del	del/del	ER05066	3	F
HXR2762	0.13	del/del	del/del	ER05048	2	F
HXR2766	0.13	del/del	del/del	ER05036	2	F
HXR0851	0.13	del/del	del/del	ER12306	3	F
HXR1627	0.13	del/del	del/del	ER34217	3	F
HXR2893	0.11	del/del	del/del	ER39352	2	F
HXR2771	0.3	del/del	del/+	ER05041	2	F
HXR2806	0.24	del/del	del/+	ER18281	3	F
HXR1939	0.26	del/del	del/+	ER17912	3	F
HXR2196	0.31	del/del	del/+	ER17851	3	F
HXR2006	0.36	del/del	del/+	ER39427	2	F
HXR2769	0.2	del/del	del/+	ER05042	2	F
HXR2200	0.24	del/del	del/+	ER32664	3	F
HXR1937	0.38	del/del	del/+	ER17947	3	F
HXR2370	0.23	del/del	del/+	ER17824	3	F
HXR2210	0.19	del/del	del/+	ER32085	3	F
HXR2166	0.21	del/del	del/+	ER17849	3	F
HXR2758	0.22	del/del	del/+	ER05049	3	F
HXR2606	0.26	del/del	del/+	ER39329	2	F
HXR2573	0.28	del/del	del/+	ER29446	3	F
HXR2612	0.27	del/del	del/+	ER39035	3	N
HXR0963	0.37	del/del	del/+	ER29748	2	F
HXR2091	0.43	del/del	del/+	ER19150	2	F
HXR2372	0.43	del/del	del/+	ER17914	2	F
HXR1571	0.25	del/del	del/+	ER02014	3	F
HXR0962	0.48	del/del	del/+	ER29640	3	F
HXR3120	0.34	del/del	del/+	ER28359	3	F
HXR3162	0.14	del/del	del/+	ER28744	3	F
HXR1438	0.22	del/del	del/+	ER04143	2	F
HXR1309	0.25	del/del	del/+	ER41289	3	F
HXR1070	0.33	del/del	del/+	ER02034	3	F
HXR1378	0.47	del/del	del/+	ER10986	3	F
HXR1069	0.23	del/del	del/+	ER02021	3	F
HXR1326	0.29	del/del	del/+	ER12216	3	F
HXR0887	0.3	del/del	del/+	ER12083	3	F
HXR1233	0.33	del/del	del/+	ER05320	3	F
HXR1123	0.35	del/del	del/+	ER04320	2	F
HXR1192	0.35	del/del	del/+	ER41389	3	F
HXR1236	0.35	del/del	del/+	ER04313	3	F
HXR0941	0.37	del/del	del/+	ER29745	3	F
HXR1091	0.43	del/del	del/+	ER12268	3	F
HXR0937	0.45	del/del	del/+	ER12152	3	F
HXR0739	0.27	del/del	del/+	ER02040	3	F
HXR0925	0.26	del/del	del/+	ER12157	3	F
HXR1555	0.27	del/del	del/+	ER00713	3	F
HXR1122	0.42	del/del	del/+	ER05395	3	F
HXR0878	0.51	del/del	del/+	ER12066	2	F
HXR1566	0.29	del/del	del/+	ER00741	3	F

HXR0867	0.35	del/del	del/+	ER12182	3	F
HXR1082	0.36	del/del	del/+	ER12354	2	F
HXR0875	0.37	del/del	del/+	ER12086	3	F
HXR1557	0.38	del/del	del/+	ER00773	2	F
HXR1769	0.42	del/del	del/+	ER39374	2	F
HXR0837	0.43	del/del	del/+	ER12244	3	F
HXR1317	0.5	del/del	del/+	ER00704	2	F
HXR1560	0.23	del/del	del/+	ER00701	2	F
HXR1111	0.27	del/del	del/+	ER05333	3	F
HXR1572	0.36	del/del	del/+	ER00729	3	F
HXR0127	0.29	del/del	del/+	ER24958	3	F
HXR1104	0.75	del/del	del/+	ER12173	3	F
HXR1314	0.66	del/del	del/+	ER00737	2	F
HXR0806	0.6	del/del	del/+	ER02019	3	F
HXR1103	0.58	del/del	del/+	ER12295	3	F
HXR2804	0.55	del/del	del/+	ER18238	3	F
HXR0781	0.54	del/del	del/+	ER12123	3	F
HXR1248	0.19	del/del	del/+	ER04184	3	F
HXR1093	0.18	del/del	del/+	ER12114	3	F
HXR1308	0.18	del/del	del/+	ER41234	3	F
HXR2811	0.17	del/del	del/+	ER05033	2	F
HXR0812	0.16	del/del	del/+	ER02009	3	F
HXR1112	0.16	del/del	del/+	ER01833	2	F
HXR2777	0.14	del/del	del/+	ER05039	2	F
HXR1468	0.13	del/del	del/+	ER01816	3	F
HXR1133	0.12	del/del	del/+	ER01828	3	F
HXR2199	0.21	del/del	+/+	ER32073	3	F
HXR1924	0.36	del/del	+/+	ER17847	3	F
HXR2234	0.28	del/del	+/+	ER23978	2	F
HXR2639	0.25	del/del	+/+	ER39375	2	F
HXR2619	0.35	del/del	+/+	ER39506	3	F
HXR2814	0.21	del/del	+/+	ER05063	2	F
HXR1792	0.25	del/del	+/+	ER18611	2	F
HXR1459	0.37	del/del	+/+	ER09237	2	F
HXR2363	0.43	del/del	+/+	ER25613	3	F
HXR1304	0.42	del/del	+/+	ER41270	3	F
HXR1079	0.43	del/del	+/+	ER12264	3	F
HXR1591	0.2	del/del	+/+	ER34183	2	F
HXR1653	0.18	del/del	+/+	ER32087	3	F
HXR1651	0.12	del/del	+/+	ER32083	3	F
HXR2395	0.38	del/+	del/del	ER17808	3	F
HXR2261	0.13	del/+	del/del	ER23721	3	F
HXR1974	0.22	del/+	del/del	ER35861	3	F
HXR2168	0.19	del/+	del/del	ER17941	3	F
HXR2551	0.24	del/+	del/del	ER39332	2	F
HXR0439	0.39	del/+	del/del	ER09612	3	F
HXR3226	0.26	del/+	del/del	ER37570	3	F
HXR3196	0.32	del/+	del/del	ER37571	3	F
HXR3165	0.3	del/+	del/del	ER28716	3	F
HXR0890	0.34	del/+	del/del	ER12250	2	F

HXR0760	0.42	del/+	del/del	ER12160	3	F
HXR1466	0.32	del/+	del/del	ER01814	3	F
HXR0766	0.33	del/+	del/del	ER12315	3	F
HXR1828	0.2	del/+	del/del	ER18132	2	F
HXR0844	0.28	del/+	del/del	ER12270	3	F
HXR0835	0.47	del/+	del/del	ER00777	2	F
HXR0866	0.49	del/+	del/del	ER12179	2	F
HXR1787	0.28	del/+	del/del	ER39533	3	F
HXR0904	0.19	del/+	del/del	ER12263	3	F
HXR1993	0.17	del/+	del/del	ER35872	3	F
HXR2179	0.22	del/+	del/+	ER17870	3	F
HXR2160	0.26	del/+	del/+	ER17904	3	F
HXR2794	0.41	del/+	del/+	ER18570	3	F
HXR2367	0.31	del/+	del/+	ER25606	3	F
HXR2379	0.34	del/+	del/+	ER17692	2	F
HXR2170	0.32	del/+	del/+	ER17833	3	F
HXR2193	0.22	del/+	del/+	ER17843	3	F
HXR1200	0.21	del/+	del/+	ER41331	3	F
HXR0758	0.35	del/+	del/+	ER12320	2	F
HXR1196	0.21	del/+	del/+	ER41334	3	F
HXR1136	0.24	del/+	del/+	ER05322	3	F
HXR1128	0.27	del/+	del/+	ER01818	3	F
HXR0870	0.35	del/+	del/+	ER12117	3	F
HXR1108	0.35	del/+	del/+	ER05304	2	F
HXR1097	0.4	del/+	del/+	ER00759	3	F
HXR0936	0.42	del/+	del/+	ER12094	3	F
HXR0892	0.44	del/+	del/+	ER12178	3	F
HXR0883	0.48	del/+	del/+	ER12149	3	F
HXR1110	0.48	del/+	del/+	ER05306	3	F
HXR0877	0.26	del/+	del/+	ER12343	3	F
HXR2377	0.18	del/+	del/+	ER17804	3	F
HXR2368	0.17	del/+	del/+	ER17813	3	F
HXR2783	0.44	del/+	+/+	ER05062	2	F
HXR1569	0.19	del/+	+/+	ER01480	3	F
HXR2770	0.16	+/+	del/del	ER04617	3	F
HXR1389	0.15	+/+	del/+	ER10921	3	F
HXR0746	0.35	+/+	+/+	ER00722	3	F

**Surveyor**
kjg
bsc
kjg
bsc
kjg
kjg
bsc
kjg
bsc
bsc
kjg
kjg
kjg
bsc
bsc
kjg
bsc
bsc
kjg
kjg
kjg
bsc
kjg
kjg
bsc
kjg
kjg
bsc
bsc
kjg
kjg
kjg
bsc
kjg
bsc
kjg
kjg
bsc
bsc
kjg
kjg
kjg
kjg
bsc
kjg
kjg
kjg
kjg

kjg
kjg
kjg
kjg
kjg
kjg
kjg
kjg
kjg
kjg
kjg
kjg
kjg
kjg
kjg
kjg
kjg
bsc
kjg
bsc
kjg
kjg
kjg
kjg
kjg
kjg
kjg
kjg
kjg
kjg
kjg
kjg
kjg
bsc
kjg
kjg
kjg
kjg
kjg
kjg
kjg
bsc
kjg
kjg
kjg
kjg
kjg
kjg
kjg
kjg

bsc
bsc
bsc
bsc
bsc
kjg
kjg
kjg
bsc
kjg
bsc
bsc
kjg
bsc
bsc
bsc
kjg
kjg
bsc
bsc
kjg
kjg
kjg
kjg
kjg
kjg
kjg
kjg
bsc
kjg
bsc
bsc
kjg
kjg
kjg
kjg
kjg
kjg
kjg
bsc
kjg
kjg
kjg
kjg
kjg
kjg
kjg
kjg
kjg
kjg

kjg
kjg
kjg
bsc
kjg
kjg
kjg
kjg
kjg
kjg
kjg
kjg
kjg
kjg
kjg
kjg
kjg
kjg
kjg
bsc
bsc
kjg
bsc
bsc
bsc
bsc
kjg
bsc
bsc
kjg
kjg
bsc
kjg
kjg
bsc
bsc
kjg
kjg
kjg
kjg
kjg
kjg
kjg
bsc
kjg
bsc
bsc
bsc
kjg
kjg

kjg
bsc
kjg
kjg
kjg
bsc
kjg
kjg
kjg
kjg
bsc
bsc
kjg
bsc
kjg
bsc
bsc
bsc
kjg
bsc
kjg
bsc
kjg
kjg
kjg
kjg
kjg
kjg
kjg
kjg
kjg
kjg
bsc
kjg
bsc
kjg
kjg

Supplementary Table s11: Cashmere diameter and Gnotype

Sample	Diameter（um)	FGF5	LHX2	Earmark	Age(years)
HXR3749	15.27	del/del	del/del	ER23538	3
HXR3752	14.74	del/del	del/del	ER23590	2
HXR3176	13.81	del/+	del/+	ER33342	2
HXR3149	14.85	del/del	del/del	ER18250	2
HXR3144	14.09	del/del	del/del	ER39747	2
HXR3581	16.47	del/del	del/del	ER36633	3
HXR3152	14.05	del/del	del/del	ER39559	3
HXR2445	16.22	del/del	del/del	ER39385	3
HXR3750	14.07	del/+	del/+	ER36710	2
HXR3738	14.01	del/+	del/+	ER36649	3
HXR3352	15.39	del/del	del/del	ER39686	3
HXR3745	14.7	del/del	del/del	ER39836	2
HXR3739	13.3	del/del	del/del	ER35291	3
HXR3138	14.07	del/+	del/+	ER27653	2
HXR3315	17.06	del/del	del/del	ER36346	3
HXR3288	14.85	del/del	del/del	ER36219	2
HXR3742	13.94	del/+	del/+	ER35365	3
HXR3748	14.71	del/del	del/del	ER37737	3
HXR2457	15.68	del/del	del/del	ER37786	3
HXR1484	13.87	del/+	del/+	ER32926	3
HXR2531	14.24	del/del	del/del	ER35389	3
HXR3257	13.91	del/del	del/del	ER33278	2
HXR3665	14.09	del/del	del/del	ER32916	3
HXR3103	15.74	del/del	del/del	ER33130	3
HXR3119	15.22	del/del	del/del	ER33149	3
HXR3401	15.56	+/+	+/+	ER28693	2
HXR3334	14.05	del/del	del/del	ER30207	3
HXR3083	13.83	del/del	del/del	ER36642	3
HXR3669	16.75	del/del	del/del	ER28684	3
HXR3151	13.68	del/del	del/del	ER33626	3
HXR3680	14.67	del/+	del/+	ER33106	3
HXR3110	15.53	del/+	del/+	ER30132	3
HXR3142	15.35	del/del	del/del	ER30105	3
HXR3376	16.57	del/del	del/del	ER28755	2
HXR3548	15.11	del/+	del/+	ER33711	3
HXR3082	15.42	del/del	del/del	ER30140	2
HXR3715	13.28	del/del	del/del	ER30146	2
HXR3565	14.1	del/del	del/del	ER32831	3
HXR3080	15.12	del/del	del/del	ER27038	3
HXR3161	14.23	del/+	del/+	ER29307	2
HXR3170	14.07	del/del	del/del	ER36396	3
HXR3564	14.93	del/+	del/+	ER31568	3
HXR3604	14.04	del/del	del/del	ER28741	3
HXR3134	15.54	del/del	del/del	ER33307	2
HXR3117	14.97	del/del	del/del	ER33138	2
HXR3222	14.19	del/del	del/del	ER36258	3
HXR3158	14.1	del/del	del/del	ER35126	3
HXR3636	13.6	del/del	del/del	ER30074	3

HXR3156	15.95	del/del	del/del	ER33777	2
HXR3706	13.95	del/del	del/del	ER33110	3
HXR3092	14.22	del/del	del/del	ER32977	3
HXR3107	15.09	del/del	del/del	ER36342	2
HXR3385	14.04	del/del	del/del	ER30133	3
HXR3373	14.53	del/del	del/del	ER35132	3
HXR3155	13.18	del/del	del/del	ER35371	3
HXR3681	14.24	del/del	del/del	ER36676	3
HXR3606	15.32	del/del	del/del	ER36199	3
HXR3612	14.98	del/del	del/del	ER33667	3
HXR3407	13.49	del/del	del/del	ER35992	3
HXR3381	13.52	del/del	del/del	ER35974	3
HXR3705	14.71	del/+	del/+	ER33686	3
HXR3619	14.28	del/del	del/del	ER32938	2
HXR3095	14.76	del/del	del/del	ER36218	3
HXR3403	14.1	del/del	del/del	ER35139	3
HXR3224	14.17	del/del	del/del	ER37473	3
HXR3357	13.02	del/+	del/+	ER33424	2
HXR3545	15.72	del/del	del/del	ER37157	2
HXR3114	15.46	del/del	del/del	ER29723	3
HXR3195	13.45	del/del	del/del	ER33841	2
HXR3690	13.04	del/del	del/del	ER32896	3
HXR3201	15.45	del/del	del/del	ER35965	3
HXR3252	16.09	del/del	del/del	ER37482	3
HXR3356	13.98	del/+	del/+	ER32957	3
HXR3194	13.73	del/del	del/del	ER36599	3
HXR3314	14.78	del/del	del/del	ER35308	3
HXR3379	14.27	del/del	del/del	ER32946	2
HXR3459	13.55	del/del	del/del	ER33608	3
HXR3611	13.61	del/del	del/del	ER36578	3
HXR3589	13.82	del/del	del/del	ER30155	3
HXR3175	13.31	del/del	del/del	ER33283	2
HXR3181	15.95	del/del	del/del	ER37469	3
HXR3337	14.27	+/+	+/+	ER32955	3
HXR3311	15.15	del/del	del/del	ER35366	3
HXR3206	13.47	del/del	del/del	ER32964	3
HXR3363	13.78	del/del	del/del	ER37534	3
HXR3569	15.89	del/del	del/del	ER36373	3
HXR3653	14.19	del/+	del/+	ER35994	3
HXR3320	14.45	del/del	del/del	ER36289	3
HXR3448	14.06	del/del	del/del	ER36183	3
HXR3384	13.74	del/del	del/del	ER31449	3
HXR3291	14.16	del/del	del/del	ER36404	3
HXR3672	14.12	del/del	del/del	ER35130	3
HXR3213	13.99	del/del	del/del	ER36294	3
HXR3264	16.74	del/del	del/del	ER36344	2
HXR3346	13.49	del/del	del/del	ER33445	3
HXR3622	13.96	del/+	del/+	ER37779	3
HXR3277	15.82	del/del	del/del	ER27428	3
HXR3228	15.71	del/del	del/del	ER36271	3

HXR3247	15.98	del/del	del/del	ER36284	2
HXR3338	14.92	del/del	del/del	ER36699	3
HXR3639	13.91	del/del	del/del	ER32842	3
HXR3321	12.85	del/del	del/del	ER26193	2
HXR3391	14.17	del/+	del/+	ER29713	3
HXR3367	15.16	NaN	NaN	ER36480	3
HXR3347	15.71	del/+	del/+	ER35924	3
HXR3628	14.05	del/del	del/del	ER35121	3
HXR3280	13.9	del/del	del/del	ER29705	3
HXR3483	14.27	del/del	del/del	ER36216	3
HXR3618	15.28	del/del	del/del	ER35315	3
HXR3306	16.14	del/+	del/+	ER35393	3
HXR3260	15.97	del/del	del/del	ER37481	3
HXR3290	15.27	del/del	del/del	ER30492	3
HXR3431	13.49	del/del	del/del	ER33368	2
HXR3212	15.5	del/del	del/del	ER30999	2
HXR3269	15.21	del/del	del/del	ER33350	3
HXR3716	14.98	del/del	del/del	ER33871	2
HXR3325	14.56	del/del	del/del	ER30970	2
HXR3655	15.06	del/+	del/+	ER34086	3
HXR3249	15.21	del/del	del/del	ER28563	3
HXR3259	14.96	del/+	del/+	ER34079	3
HXR3366	14.23	del/del	del/del	ER34510	3
HXR3230	17.1	del/+	del/+	ER39958	2
HXR3626	13.23	del/+	del/+	ER34408	2
HXR3319	15.37	del/del	del/del	ER33816	3
HXR3707	14.25	del/del	del/del	ER30384	3
HXR3266	14.27	del/del	del/del	ER34016	3
HXR3236	14.13	del/del	del/del	ER34017	2
HXR3422	14.92	del/del	del/del	ER33875	2
HXR3293	14.19	del/+	del/+	ER34006	2
HXR3393	13.75	del/del	del/del	ER30459	3
HXR3268	15.28	del/+	del/+	ER29789	3
HXR3730	15.26	del/+	del/+	ER34005	2
HXR3425	13.96	del/+	del/+	ER27133	2
HXR3503	15.37	del/del	del/del	ER34003	2
HXR3241	16.29	del/del	del/del	ER33357	3
HXR3454	17.24	del/del	del/del	ER34004	2
HXR3714	14.06	del/+	del/+	ER30640	3
HXR3392	15.02	del/del	del/del	ER34529	3
HXR3426	16.31	del/+	del/+	ER33878	3
HXR3645	16.17	del/del	del/del	ER33356	2
HXR3262	14.06	del/del	del/del	ER34019	2
HXR3734	14.64	del/del	del/del	ER33348	2
HXR3317	14.26	del/del	del/del	ER35452	3
HXR3255	15	del/del	del/del	ER34044	2
HXR3332	14.91	del/del	del/del	ER30100	3
HXR3427	15.32	del/del	del/del	ER31209	3
HXR3736	13.69	del/del	del/del	ER35463	3
HXR3467	14.02	del/del	del/del	ER30241	2

HXR3396	14.91	del/del	del/del	ER28603	2
HXR3336	13.99	del/del	del/del	ER31224	3
HXR3343	14.44	del/+	del/+	ER33384	3
HXR3499	14.61	del/del	del/del	ER30703	3
HXR3272	13.89	del/del	del/del	ER30242	2
HXR3479	14.39	del/del	del/del	ER30083	3
HXR3254	14.87	del/del	del/del	ER34001	2
HXR3186	15.23	del/del	del/del	ER35480	3
HXR3284	15.94	del/+	del/+	ER27132	2
HXR2351	14.01	del/+	del/+	ER30038	3
HXR3472	14.65	del/del	del/del	ER29565	3
HXR3686	13.25	del/del	del/del	ER34013	2
HXR3525	14.75	del/del	del/del	ER29938	3
HXR3733	13.49	del/del	del/del	ER35485	3
HXR3221	15	del/del	del/del	ER34528	3
HXR3558	13.57	del/del	del/del	ER30955	2
HXR3512	14.71	del/del	del/del	ER29560	3
HXR3718	14.27	del/del	del/del	ER30277	3
HXR3735	15.69	del/+	del/+	ER37700	3
HXR3722	14.3	del/del	del/del	ER30993	3
HXR3726	12.94	del/del	del/del	ER29658	3
HXR3737	13.55	del/del	del/del	ER28209	2
HXR3741	13.79	del/del	del/del	ER30051	3
HXR3132	13.83	del/del	del/del	ER28207	3
HXR3128	13.86	del/del	del/del	ER30560	3
HXR3101	14.87	del/del	del/del	ER29535	3
HXR3615	13.68	del/del	del/del	ER30725	3
HXR3532	13.79	del/del	del/del	ER34049	2
HXR3183	14.9	del/del	del/del	ER30946	3
HXR3547	12.8	del/del	del/del	ER33393	2
HXR3670	13.52	del/del	del/del	ER29642	2
HXR3140	14.55	del/del	del/del	ER29586	3
HXR3112	15.7	del/del	del/del	ER30977	3
HXR3552	14.66	del/del	del/del	ER29555	2
HXR3660	13.98	del/+	del/+	ER33346	2
HXR3097	15.05	del/del	del/del	ER35431	3
HXR3113	13.89	del/del	del/del	ER34023	2
HXR3100	13.58	del/+	del/+	ER31222	3
HXR3387	13.87	del/del	del/del	ER30574	2
HXR3232	13.54	del/del	del/del	ER34055	2
HXR3751	13.52	del/del	del/del	ER30901	2
HXR3555	13.53	del/del	del/del	ER30906	2
HXR3189	13.8	del/del	del/del	ER30709	3
HXR3709	14.99	del/del	del/del	ER29876	2
HXR3755	13.86	del/+	del/+	ER29561	3
HXR3300	13.27	del/del	del/del	ER35432	3
HXR3182	14.33	del/del	del/del	ER29566	3
HXR3324	13.49	del/del	del/del	ER26384	3
HXR3530	14.99	del/+	del/+	ER27700	2
HXR3369	13.82	del/+	del/+	ER28611	3

HXR3551	13.51	del/del	del/del	ER26716	2
HXR3187	15.03	del/del	del/del	ER13105	3
HXR3326	13.8	del/del	del/del	ER27920	2
HXR3331	14.46	del/+	del/+	ER17812	3
HXR3372	13.57	del/del	del/del	ER04385	3
HXR3286	14.8	del/del	del/del	ER39359	3
HXR3434	14.3	del/del	del/del	ER28672	2
HXR3754	14.56	del/del	del/del	ER27646	3
HXR3531	14.68	del/del	del/del	ER26244	3
HXR3245	14.93	del/del	del/del	ER26053	2
HXR3333	13.29	del/del	del/del	ER26531	3
HXR3541	14.78	del/del	del/del	ER27041	3
HXR3701	14.03	del/del	del/del	ER26198	2
HXR3143	14.12	del/del	del/del	ER26142	3
HXR3188	14.15	del/del	del/del	ER26422	2
HXR3178	13.83	del/del	del/del	ER26624	2
HXR3218	14.52	del/del	del/del	ER26523	3
HXR3542	13.4	del/del	del/del	ER27721	3
HXR3374	13.98	del/del	del/del	ER36953	3
HXR3238	15.47	del/del	del/del	ER28726	2
HXR3534	13.79	del/+	del/+	ER26060	2
HXR3538	14.95	del/del	del/del	ER27236	2
HXR3190	13.81	del/del	del/del	ER28384	2
HXR3362	15.03	del/del	del/del	ER28230	2
HXR3378	13.75	del/del	del/del	ER26577	2
HXR3398	13.67	del/del	del/del	ER37550	2
HXR3674	13.86	del/del	del/del	ER27286	2
HXR3410	14.46	del/del	del/del	ER37662	3
HXR3485	13.3	del/del	del/del	ER35292	3
HXR3327	13.48	del/del	del/del	ER36992	2
HXR3520	13.17	del/+	del/+	ER36930	3
HXR3447	14.25	del/del	del/del	ER26596	2
HXR3671	14.17	+/+	+/+	ER28747	3
HXR3437	14.59	del/del	del/del	ER37417	3
HXR3480	13.47	+/+	+/+	ER35516	3
HXR3527	14.82	del/del	del/del	ER37001	3
HXR3282	14.09	del/del	del/del	ER37129	3
HXR3518	15.22	del/del	del/del	ER35320	3
HXR3445	13.48	del/+	del/+	ER36948	3
HXR3219	13.74	del/del	del/del	ER37120	3
HXR3684	14.29	del/+	del/+	ER36618	2
HXR3438	13.83	del/del	del/del	ER36423	3
HXR3220	13.89	del/del	del/del	ER37006	3
HXR3675	15.56	del/del	del/del	ER25260	3
HXR3510	14.45	del/+	del/+	ER36917	2
HXR3743	15.28	del/del	del/del	ER28126	3
HXR3516	14.63	del/del	del/del	ER27695	2
HXR3420	13.7	del/del	del/del	ER26556	3
HXR3478	13.27	del/del	del/del	ER26748	3
HXR3428	14.04	del/del	del/del	ER27780	3

HXR3446	14.87	del/+	del/+	ER27072	3
HXR3469	13.66	del/del	del/del	ER27186	3
HXR3513	14.85	+/+	+/+	ER27188	3
HXR3484	13.3	del/+	del/+	ER27519	2
HXR3413	13.84	del/+	del/+	ER36984	3
HXR3720	13.76	del/del	del/del	ER37633	3
HXR3329	13.32	del/del	del/del	ER26058	2
HXR3539	13.79	del/del	del/del	ER37080	3
HXR3526	14.92	del/+	del/+	ER36965	3
HXR3414	13.26	del/del	del/del	ER28399	3
HXR3481	13.82	del/del	del/del	ER37553	3
HXR3505	14.56	del/del	del/del	ER26357	2
HXR3517	13.8	del/del	del/del	ER35939	3
HXR3486	14.86	del/del	del/del	ER35430	3
HXR3500	12.85	del/del	del/del	ER36950	3
HXR3271	13.85	del/del	del/del	ER36418	3
HXR0988	12.98	del/del	del/del	ER37794	3
HXR3506	14.32	del/del	del/del	ER12472	3
HXR3239	14.37	del/+	del/+	ER36352	2
HXR1387	14.06	del/del	del/del	ER09251	3
HXR0851	13.76	del/del	del/del	ER12306	3
HXR0777	13.92	del/del	del/del	ER12265	2
HXR1313	13.76	del/del	del/del	ER12169	3
HXR0824	14.13	NaN	NaN	ER05259	3
HXR0806	13.2	del/+	del/del	ER02019	3
HXR0745	14.08	+/+	del/del	ER00726	3
HXR0781	13.8	del/+	del/del	ER12123	3
HXR1314	14.06	del/+	del/del	ER00737	2
HXR0775	13.88	del/del	del/del	ER12273	2
HXR0792	13.32	del/del	del/del	ER02028	3
HXR0761	13.61	NaN	NaN	ER12185	3
HXR1092	14	del/del	del/del	ER12159	2
HXR0770	13.29	del/del	del/del	ER12098	3
HXR1583	13.92	del/del	del/del	ER00738	2
HXR1370	13.12	del/+	NaN	ER10946	3
HXR1318	13.22	del/del	del/del	ER12302	2
HXR1389	13.54	del/+	+/+	ER10921	3
HXR1248	13.98	del/+	del/del	ER04184	3
HXR1103	13.92	del/+	del/del	ER12295	3
HXR1802	13.79	del/del	del/del	ER18824	2
HXR0882	13.8	del/del	del/del	ER12243	3
HXR1574	13.19	del/del	del/del	ER00751	3
HXR1093	13.88	del/+	del/del	ER12114	3
HXR1796	13.96	del/del	del/del	ER18581	2
HXR1319	14.1	del/del	del/del	ER12236	3
HXR0904	13.28	del/del	del/+	ER12263	3
HXR1569	14.05	+/+	del/+	ER01480	3
HXR1104	13.78	del/+	del/del	ER12173	3
HXR0864	13.71	del/del	del/del	ER12116	2
HXR0881	13.8	del/del	del/del	ER12274	3

HXR0836	14.01	del/del	del/del	ER12358	2
HXR0880	13.58	del/del	del/del	ER12050	3
HXR0909	13.66	del/del	del/del	ER12346	2
HXR0902	13.34	NaN	NaN	ER12355	3
HXR1834	14.13	NaN	del/del	ER17461	3
HXR2607	14.05	NaN	NaN	ER39539	2
HXR2377	14.01	del/+	del/+	ER17804	3
HXR2368	13.89	del/+	del/+	ER17813	3
HXR2804	13.3	del/+	del/del	ER18238	3
HXR1559	13.88	del/del	del/del	ER00703	3
HXR0750	13.67	del/del	del/del	ER12124	3
HXR0765	13.91	del/del	del/del	ER12096	3
HXR2068	16.12	del/del	NaN	ER18730	3
HXR0812	13.2	del/+	del/del	ER02009	3
HXR0798	13.6	NaN	del/del	ER12045	3
HXR1627	14.08	del/del	del/del	ER34217	3
HXR1468	13.29	del/+	del/del	ER01816	3
HXR1653	14.17	+/+	del/del	ER32087	3
HXR1651	14.07	+/+	del/del	ER32083	3
HXR0971	13.71	del/del	del/del	ER29612	2
HXR1308	13.66	del/+	del/del	ER41234	3
HXR1199	13.66	del/del	del/del	ER41095	2
HXR0949	13.87	del/del	del/del	ER29610	3
HXR1133	13.28	del/+	del/del	ER01828	3
HXR1112	13.46	del/+	del/del	ER01833	2
HXR2243	13.92	del/del	del/del	ER23972	2
HXR2815	12.54	del/del	del/del	ER05058	3
HXR2811	14.05	del/+	del/del	ER05033	2
HXR2766	12.45	del/del	del/del	ER05036	2
HXR2770	12.37	del/del	+/+	ER04617	3
HXR2893	15.18	del/del	del/del	ER39352	2
HXR2762	14	del/del	del/del	ER05048	2
HXR2777	12.92	del/+	del/del	ER05039	2
HXR2749	14.04	del/del	del/del	ER05071	2
HXR1993	14.18	del/del	del/+	ER35872	3
HXR2826	12.69	del/del	del/+	ER05047	2
HXR2764	13.11	NaN	del/del	ER05054	3
HXR3135	15.27	del/del	del/del	ER28367	3
HXR3168	13.91	del/del	del/del	ER28749	3
HXR2776	12.79	del/del	del/del	ER05066	3
HXR0970	13.68	del/del	del/del	ER29619	2
HXR1158	13.76	del/del	del/del	ER41707	N
HXR1928	13.9	del/del	del/del	ER17930	3
HXR0127	13.53	del/del	del/+	ER24958	3
HXR0186	13.09	del/del	del/del	ER24960	2
HXR0370	13.42	del/del	del/del	ER41130	3
HXR1387	14.06	del/del	del/del	ER09251	3
HXR1392	13.72	del/del	del/del	ER09249	2
HXR1046	13.39	del/del	del/del	ER09390	2
HXR3162	14.08	del/del	del/+	ER28744	3

HXR1459	13.59	del/del	+/+	ER09237	2
HXR0439	14.05	del/+	del/del	ER09612	3
HXR0690	13.98	del/del	del/del	ER42534	2
HXR0550	14.15	del/del	del/del	ER04035	3
HXR0716	14.12	del/del	del/del	ER41142	3
HXR0851	13.76	del/del	del/del	ER12306	3
HXR0739	13.35	del/del	del/+	ER02040	3
HXR0777	13.92	del/del	del/del	ER12265	2
HXR0825	13.69	del/del	del/del	ER02032	3
HXR1313	13.76	del/del	del/del	ER12169	3
HXR0806	13.2	del/del	del/+	ER02019	3
HXR0745	14.08	del/del	+/+	ER00726	3
HXR0781	13.8	del/del	del/+	ER12123	3
HXR0766	14	del/+	del/del	ER12315	3
HXR1812	13.38	del/del	del/del	ER18140	2
HXR1314	14.06	del/del	del/+	ER00737	2
HXR0758	13.43	del/+	del/+	ER12320	2
HXR1237	13.58	del/del	del/del	ER04315	3
HXR0775	13.88	del/del	del/del	ER12273	2
HXR0792	13.32	del/del	del/del	ER02028	3
HXR1092	14	del/del	del/del	ER12159	2
HXR1398	13.56	del/del	del/del	ER10903	3
HXR0770	13.29	del/del	del/del	ER12098	3
HXR0760	13.72	del/+	del/del	ER12160	3
HXR1583	13.92	del/del	del/del	ER00738	2
HXR1378	13.51	del/del	del/+	ER10986	3
HXR1070	14.38	del/del	del/+	ER02034	3
HXR1798	14.02	del/del	del/del	ER18148	3
HXR1143	13.66	del/del	del/del	ER05392	2
HXR0746	13.77	+/+	+/+	ER00722	3
HXR1091	12.86	del/del	del/+	ER12268	3
HXR0890	13.04	del/+	del/del	ER12250	2
HXR0937	13.58	del/del	del/+	ER12152	3
HXR1326	13.13	del/del	del/+	ER12216	3
HXR1318	13.22	del/del	del/del	ER12302	2
HXR1123	14.01	del/del	del/+	ER04320	2
HXR1130	13.68	del/del	del/del	ER05393	2
HXR1321	12.86	del/del	del/del	ER12349	3
HXR0887	13.47	del/del	del/+	ER12083	3
HXR1069	13.36	del/del	del/+	ER02021	3
HXR1068	13.13	del/del	del/del	ER00748	3
HXR1236	13.48	del/del	del/+	ER04313	3
HXR1233	14.13	del/del	del/+	ER05320	3
HXR1389	13.54	+/+	del/+	ER10921	3
HXR1248	13.98	del/del	del/+	ER04184	3
HXR1129	13.74	del/del	del/del	ER05311	2
HXR1103	13.92	del/del	del/+	ER12295	3
HXR1802	13.79	del/del	del/del	ER18824	2
HXR1079	14.15	del/del	+/+	ER12264	3
HXR1572	14.03	del/del	del/+	ER00729	3

HXR1121	14.04	del/del	del/del	ER05400	2
HXR1560	13.57	del/del	del/+	ER00701	2
HXR1111	14.07	del/del	del/+	ER05333	3
HXR1787	13.74	del/+	del/del	ER39533	3
HXR0882	13.8	del/del	del/del	ER12243	3
HXR1581	13.19	del/del	del/del	ER00744	3
HXR0883	13.92	del/+	del/+	ER12149	3
HXR1574	13.19	del/del	del/del	ER00751	3
HXR1110	13.87	del/+	del/+	ER05306	3
HXR1108	14	del/+	del/+	ER05304	2
HXR1093	13.88	del/del	del/+	ER12114	3
HXR1796	13.96	del/del	del/del	ER18581	2
HXR0844	13.91	del/+	del/del	ER12270	3
HXR0878	13.68	del/del	del/+	ER12066	2
HXR0879	13.02	del/del	del/del	ER12253	3
HXR1122	13.58	del/del	del/+	ER05395	3
HXR1833	14.07	del/del	del/del	ER17474	2
HXR1136	13.66	del/+	del/+	ER05322	3
HXR0892	13.63	del/+	del/+	ER12178	3
HXR1555	13.09	del/del	del/+	ER00713	3
HXR1828	13.84	del/+	del/del	ER18132	2
HXR0866	13.85	del/+	del/del	ER12179	2
HXR1097	13.26	del/+	del/+	ER00759	3
HXR0925	13.57	del/del	del/+	ER12157	3
HXR0870	14.16	del/+	del/+	ER12117	3
HXR0936	14.12	del/+	del/+	ER12094	3
HXR1319	14.1	del/del	del/del	ER12236	3
HXR0904	13.28	del/+	del/del	ER12263	3
HXR0889	13.98	del/del	del/del	ER12335	2
HXR1566	13.68	del/del	del/+	ER00741	3
HXR1569	14.05	del/+	+/+	ER01480	3
HXR1082	14.02	del/del	del/+	ER12354	2
HXR0888	13.78	del/del	del/del	ER12147	3
HXR1104	13.78	del/del	del/+	ER12173	3
HXR1317	14.02	del/del	del/+	ER00704	2
HXR1830	13.91	del/del	del/del	ER17475	2
HXR0875	12.84	del/del	del/+	ER12086	3
HXR0864	13.71	del/del	del/del	ER12116	2
HXR0850	14.04	del/del	del/del	ER05260	3
HXR0881	13.8	del/del	del/del	ER12274	3
HXR0867	13.89	del/del	del/+	ER12182	3
HXR0836	14.01	del/del	del/del	ER12358	2
HXR0900	13.1	del/del	del/del	ER12036	3
HXR0837	14.15	del/del	del/+	ER12244	3
HXR1769	12.94	del/del	del/+	ER39374	2
HXR1841	13.66	del/del	del/del	ER17456	2
HXR1100	13.64	del/del	del/del	ER12119	3
HXR0880	13.58	del/del	del/del	ER12050	3
HXR0909	13.66	del/del	del/del	ER12346	2
HXR0877	13.33	del/+	del/+	ER12343	3

HXR2551	13.88	del/+	del/del	ER39332	2
HXR2444	13.99	del/del	del/del	ER17660	2
HXR2612	13.39	del/del	del/+	ER39035	3
HXR2476	13.53	del/del	del/del	ER17690	3
HXR2606	13.97	del/del	del/+	ER39329	2
HXR2370	13.11	del/del	del/+	ER17824	3
HXR2377	14.01	del/+	del/+	ER17804	3
HXR2482	13.85	del/del	del/del	ER39431	2
HXR2619	13.77	del/del	+/+	ER39506	3
HXR2639	13.81	del/del	+/+	ER39375	2
HXR2368	13.89	del/+	del/+	ER17813	3
HXR2379	13.81	del/+	del/+	ER17692	2
HXR2787	13.86	del/del	del/del	ER18233	3
HXR2791	13.47	del/del	del/del	ER18225	2
HXR2804	13.3	del/del	del/+	ER18238	3
HXR2802	13.86	del/del	del/del	ER18223	3
HXR2395	14.07	del/+	del/del	ER17808	3
HXR2063	14.52	del/del	del/del	ER04686	3
HXR2794	13.62	del/+	del/+	ER18570	3
HXR2788	12.54	del/del	del/del	ER18275	2
HXR2006	14.05	del/del	del/+	ER39427	2
HXR2061	14.06	del/del	del/del	ER00858	3
HXR2806	13.42	del/del	del/+	ER18281	3
HXR2798	14.74	del/del	del/del	ER18265	3
HXR0736	13.37	del/del	del/del	ER00734	3
HXR1559	13.88	del/del	del/del	ER00703	3
HXR1571	13.14	del/del	del/+	ER02014	3
HXR1067	14.07	del/del	del/del	ER00715	3
HXR0750	13.67	del/del	del/del	ER12124	3
HXR2082	14.67	del/del	del/del	ER19136	3
HXR0765	13.91	del/del	del/del	ER12096	3
HXR2372	13.89	del/del	del/+	ER17914	2
HXR0812	13.2	del/del	del/+	ER02009	3
HXR2090	15.79	del/del	del/del	ER18568	3
HXR1792	13.92	del/del	+/+	ER18611	2
HXR2091	13.91	del/del	del/+	ER19150	2
HXR1821	14.13	del/del	del/del	ER18152	3
HXR1627	14.08	del/del	del/del	ER34217	3
HXR1468	13.29	del/del	del/+	ER01816	3
HXR1466	13.3	del/+	del/del	ER01814	3
HXR1653	14.17	del/del	+/+	ER32087	3
HXR0954	13.75	del/del	del/del	ER29756	3
HXR1651	14.07	del/del	+/+	ER32083	3
HXR1200	13.52	del/+	del/+	ER41331	3
HXR0981	13.03	del/del	del/del	ER29622	3
HXR1477	13.08	del/del	del/del	ER01831	3
HXR1309	13.33	del/del	del/+	ER41289	3
HXR1304	14	del/del	+/+	ER41270	3
HXR0971	13.71	del/del	del/del	ER29612	2
HXR1308	13.66	del/del	del/+	ER41234	3

HXR0941	14.18	del/del	del/+	ER29745	3
HXR1189	13.1	del/del	del/del	ER41103	2
HXR0979	14.19	del/del	del/del	ER29638	3
HXR1192	14.05	del/del	del/+	ER41389	3
HXR1199	13.66	del/del	del/del	ER41095	2
HXR0949	13.87	del/del	del/del	ER29610	3
HXR1133	13.28	del/del	del/+	ER01828	3
HXR1591	13.51	del/del	+/+	ER34183	2
HXR1112	13.46	del/del	del/+	ER01833	2
HXR1128	13.7	del/+	del/+	ER01818	3
HXR1196	13.88	del/+	del/+	ER41334	3
HXR0835	13.95	del/+	del/del	ER00777	2
HXR1630	13.59	del/del	del/del	ER32088	3
HXR1557	14.1	del/del	del/+	ER00773	2
HXR2243	13.92	del/del	del/del	ER23972	2
HXR2573	14.24	del/del	del/+	ER29446	3
HXR2346	14.2	del/del	del/del	ER23963	3
HXR2783	13.51	del/+	+/+	ER05062	2
HXR2815	12.54	del/del	del/del	ER05058	3
HXR2814	14.04	del/del	+/+	ER05063	2
HXR2210	13.19	del/del	del/+	ER32085	3
HXR2827	14.12	del/del	del/del	ER05005	3
HXR2758	12.53	del/del	del/+	ER05049	3
HXR2227	14.23	del/del	del/del	ER32517	3
HXR2811	14.05	del/del	del/+	ER05033	2
HXR2315	14.09	del/del	del/del	ER25253	3
HXR2766	12.45	del/del	del/del	ER05036	2
HXR2770	12.37	+/+	del/del	ER04617	3
HXR2367	13.44	del/+	del/+	ER25606	3
HXR2234	13.82	del/del	+/+	ER23978	2
HXR1974	13.97	del/+	del/del	ER35861	3
HXR2769	13.91	del/del	del/+	ER05042	2
HXR2893	15.18	del/del	del/del	ER39352	2
HXR2219	13.74	del/del	del/del	ER32528	3
HXR2752	12.62	del/del	del/del	ER05003	3
HXR2762	14	del/del	del/del	ER05048	2
HXR2781	12.54	del/del	del/del	ER05069	3
HXR2777	12.92	del/del	del/+	ER05039	2
HXR2749	14.04	del/del	del/del	ER05071	2
HXR1993	14.18	del/+	del/del	ER35872	3
HXR2771	13.88	del/del	del/+	ER05041	2
HXR2826	12.69	del/+	del/del	ER05047	2
HXR2199	13.39	del/del	+/+	ER32073	3
HXR2779	13.7	del/del	del/del	ER05034	3
HXR3135	15.27	del/del	del/del	ER28367	3
HXR3165	14.11	del/+	del/del	ER28716	3
HXR3168	13.91	del/del	del/del	ER28749	3
HXR3214	14.75	del/del	del/del	ER36241	2
HXR2776	12.79	del/del	del/del	ER05066	3
HXR3125	14.97	del/del	del/del	ER28381	2

HXR2363	14.06	del/del	+/+	ER25613	3
HXR0970	13.68	del/del	del/del	ER29619	2
HXR1454	14.15	del/del	del/del	ER01832	3
HXR0963	13.64	del/del	del/+	ER29748	2
HXR1717	13.63	del/del	del/del	ER29449	3
HXR0962	14.1	del/del	del/+	ER29640	3
HXR1158	13.76	del/del	del/del	ER41707	N
HXR1438	13.79	del/del	del/+	ER04143	2
HXR0960	13.37	del/del	del/del	ER29103	2
HXR1928	13.9	del/del	del/del	ER17930	3
HXR2166	13.85	del/del	del/+	ER17849	3
HXR1957	13.73	del/del	del/del	ER17832	3
HXR2170	13.8	del/+	del/+	ER17833	3
HXR2168	13.71	del/+	del/del	ER17941	3
HXR1941	12.69	del/del	+/+	ER17861	3
HXR1937	13.19	del/del	del/+	ER17947	3
HXR2200	13.88	del/del	del/+	ER32664	3
HXR2163	13.13	del/del	del/del	ER17890	3
HXR1939	13.72	del/del	del/+	ER17912	3
HXR2160	13.27	del/+	del/+	ER17904	3
HXR2196	13.02	del/del	del/+	ER17851	3
HXR1924	12.93	del/del	+/+	ER17847	3
HXR2179	13.13	del/+	del/+	ER17870	3
HXR2169	12.98	del/del	del/del	ER17886	3
HXR2261	13.8	del/+	del/del	ER23721	3
HXR1926	14.92	del/del	del/del	ER17927	3
HXR2190	13.76	del/del	del/del	ER17955	2
HXR3139	14.78	del/del	del/del	ER28609	2
HXR3225	13.58	del/del	+/+	ER37554	2
HXR2193	13.37	del/+	del/+	ER17843	3
HXR3226	14.24	del/+	del/del	ER37570	3
HXR1927	12.88	del/del	del/del	ER17834	3
HXR1933	13.84	del/del	del/del	ER17940	3

Gender	Surveyor
F	Test bsc
F	Test bsc
F	Test bsc
F	kjg
F	kjg
F	kjg
F	kjg
F	kjg
F	kjg
F	kjg
F	kjg
F	kjg
F	kjg
F	kjg
F	kjg
F	kjg
F	kjg
F	Testbsc
F	Testbsc
F	kjg
F	kjg
F	kjg
F	kjg
F	kjg
F	kjg
F	kjg
F	kjg
F	kjg
F	kjg
F	kjg
F	kjg
F	kjg
F	kjg
F	kjg
F	kjg
F	kjg
F	kjg
F	kjg
F	Testbsc
F	kjg
F	kjg
F	kjg
F	kjg
F	kjg
F	kjg
F	kjg
F	kjg
F	kjg

F	kjg
F	kjg
F	kjg
F	kjg
F	kjg
F	kjg
F	kjg
F	kjg
F	kjg
F	kjg
F	kjg
F	kjg
F	kjg
F	kjg
F	kjg
F	kjg
F	kjg
F	kjg
F	kjg
F	kjg
F	kjg
F	kjg
F	kjg
F	kjg
F	kjg
F	kjg
F	kjg
F	kjg
F	kjg
F	kjg
F	kjg
F	kjg
F	kjg
F	kjg
F	kjg
F	kjg
F	kjg
F	kjg
F	kjg
F	kjg
F	kjg
F	kjg
F	kjg
F	kjg
F	kjg
F	kjg
F	kjg
F	Testbsc
F	kjg
F	kjg

F	kjg
F	kjg
F	kjg
F	Testbsc
F	kjg
F	kjg
F	kjg
F	kjg
F	kjg
F	kjg
F	kjg
F	kjg
F	kjg
F	Testbsc
F	Testbsc
F	Testbsc
F	Testbsc
F	Testbsc
F	Testbsc
F	Testbsc
F	kjg
F	Testbsc
F	Testbsc
F	Testbsc
F	Testbsc
F	Testbsc
F	Testbsc
F	Testbsc
F	Testbsc
F	Testbsc
F	Testbsc
F	Testbsc
F	Testbsc
F	Testbsc
F	kjg
F	Testbsc
F	Testbsc
F	Testbsc
F	Testbsc
F	Testbsc
F	Testbsc
F	Testbsc
F	Testbsc
F	Testbsc
F	Testbsc
F	Testbsc
F	Testbsc
F	Testbsc
F	Testbsc
F	Testbsc

F	Testbsc
F	Testbsc
F	Testbsc
F	Testbsc
F	Testbsc
F	Testbsc
F	Testbsc
F	Testbsc
F	kjg
F	Testbsc
F	Testbsc
F	Testbsc
F	Testbsc
F	Testbsc
F	Testbsc
F	Testbsc
F	Testbsc
F	Testbsc
F	Testbsc
F	Testbsc
F	Testbsc
F	Testbsc
F	Testbsc
F	Testbsc
F	Testbsc
F	Testbsc
F	Testbsc
F	Testbsc
F	Testbsc
F	Testbsc
F	Testbsc
F	Testbsc
F	Testbsc
F	Testbsc
F	Testbsc
F	Testbsc
F	Testbsc
F	Testbsc
F	Testbsc
F	Testbsc
F	Testbsc
F	Testbsc
F	Testbsc
F	Testbsc
F	Testbsc
F	Testbsc
M	Testbsc
F	bsc
F	kjg
F	bsc

F	bsc
F	kjg
F	kjg
F	kjg
F	kjg
F	kjg
F	kjg
F	bsc
F	bsc
F	bsc
F	bsc
F	bsc
F	bsc
F	kjg
F	kjg
F	kjg
F	bsc
F	kjg
F	bsc
F	kjg
F	bsc
F	kjg
F	bsc
F	bsc
F	bsc
F	bsc
F	kjg
F	bsc
F	kjg
F	bsc
F	bsc
F	bsc
F	kjg
F	kjg
F	bsc
F	kjg
F	kjg
F	kjg
F	bsc
F	kjg
F	kjg
F	kjg
F	kjg
F	bsc
F	bsc
F	kjg
F	kjg
F	bsc
F	bsc
F	kjg

F	kjg
F	kjg
F	kjg
F	bsc
F	bsc
F	bsc
F	bsc
F	kjg
M	bsc
F	bsc
F	bsc
F	bsc
F	kjg
F	bsc
F	bsc
F	kjg
F	bsc
F	bsc
F	kjg
F	kjg
F	kjg
F	kjg
F	kjg
F	kjg
F	kjg
F	kjg
F	kjg
F	kjg
F	kjg
F	kjg
F	kjg
F	kjg
F	kjg
F	kjg
F	kjg
F	kjg
F	kjg
F	kjg
F	kjg
F	kjg
F	kjg
F	kjg
F	kjg
F	kjg
F	kjg
F	kjg
F	kjg
F	kjg
F	kjg
F	kjg

F	kjg
F	kjg
F	kjg
F	kjg
F	kjg
F	kjg
F	kjg
F	kjg
F	kjg
F	kjg
F	kjg
F	kjg
F	kjg
F	kjg
F	kjg
F	kjg
F	bsc
F	kjg
F	kjg
F	kjg
F	bsc
F	bsc
F	kjg
F	bsc
F	bsc
F	bsc
F	bsc
F	bsc
F	bsc
F	bsc
F	kjg
F	bsc
F	bsc
F	bsc
F	kjg
F	bsc
F	bsc
F	bsc
F	kjg
F	bsc
F	kjg
N	kjg
F	bsc
F	kjg
F	kjg
F	kjg
F	kjg
F	kjg
F	kjg
F	kjg

F	kjg
F	bsc
F	kjg
F	bsc
F	kjg
F	kjg
F	kjg
F	kjg
F	kjg
F	kjg
F	kjg
F	kjg
F	kjg
F	kjg
F	kjg
F	kjg
F	kjg
F	kjg
F	kjg
F	kjg
F	kjg
F	kjg
F	kjg
F	kjg
F	kjg
F	kjg
F	kjg
F	kjg
F	kjg
F	kjg
F	kjg
F	kjg
F	kjg
F	kjg
F	kjg
F	kjg
F	kjg
F	kjg
F	kjg
F	kjg
F	kjg
F	kjg
F	kjg
F	kjg
F	kjg
F	kjg
F	kjg
F	kjg
F	kjg
F	kjg

F	kjg
F	kjg
F	kjg
F	kjg
F	kjg
F	kjg
F	kjg
F	kjg
F	kjg
F	kjg
F	kjg
F	kjg
F	kjg
F	kjg
F	kjg
F	kjg
F	kjg
F	kjg
F	kjg
F	kjg
F	kjg
F	kjg
F	kjg
F	kjg
F	kjg
F	kjg
F	kjg
F	kjg
F	kjg
F	kjg
F	kjg
F	kjg
F	kjg
F	kjg
F	kjg
F	kjg
F	kjg
F	kjg
F	kjg
F	kjg
F	kjg
F	kjg
F	kjg
F	kjg
F	kjg
F	kjg
F	kjg
F	kjg
F	kjg
F	kjg

F	kjg
F	kjg
N	kjg
F	kjg
F	kjg
F	kjg
F	kjg
F	kjg
F	kjg
F	kjg
F	kjg
F	kjg
F	kjg
F	kjg
F	kjg
F	kjg
F	kjg
F	kjg
F	kjg
F	kjg
F	kjg
F	kjg
F	kjg
F	kjg
F	kjg
F	kjg
F	kjg
F	kjg
F	kjg
F	kjg
F	kjg
F	kjg
F	kjg
F	kjg
F	kjg
F	kjg
F	kjg
F	kjg
F	bsc
F	bsc
F	kjg
F	kjg
F	kjg
F	bsc
F	kjg
F	bsc
F	bsc
F	bsc
F	kjg
F	bsc

F	kjg
F	bsc
F	kjg
F	bsc
F	bsc
F	kjg
F	bsc
F	kjg
F	bsc
F	bsc
F	bsc
F	bsc
F	kjg
F	bsc
F	bsc
F	kjg
F	bsc
F	bsc
F	bsc
F	bsc
F	kjg
F	bsc
F	bsc
F	kjg
F	bsc
F	bsc
F	bsc
F	bsc
F	bsc
F	bsc
F	kjg
F	bsc
F	kjg
F	kjg
F	bsc
F	bsc
F	bsc
F	bsc
F	bsc
F	kjg
F	bsc
F	bsc
F	kjg
F	bsc
F	bsc
F	kjg
F	kjg
F	kjg
F	bsc
F	bsc

F	bsc
F	kjg
F	bsc
F	kjg
F	kjg
F	kjg
N	kjg
F	bsc
F	bsc
F	bsc
F	bsc
F	bsc
F	bsc
F	bsc
F	bsc
F	bsc
F	bsc
F	bsc
F	bsc
F	bsc
F	bsc
F	bsc
F	bsc
F	bsc
F	kjg
F	bsc
F	bsc
F	bsc
F	bsc
F	bsc
F	bsc
F	bsc
F	bsc

Figure

Click here to access/download;Figure;Fig. 1.tif

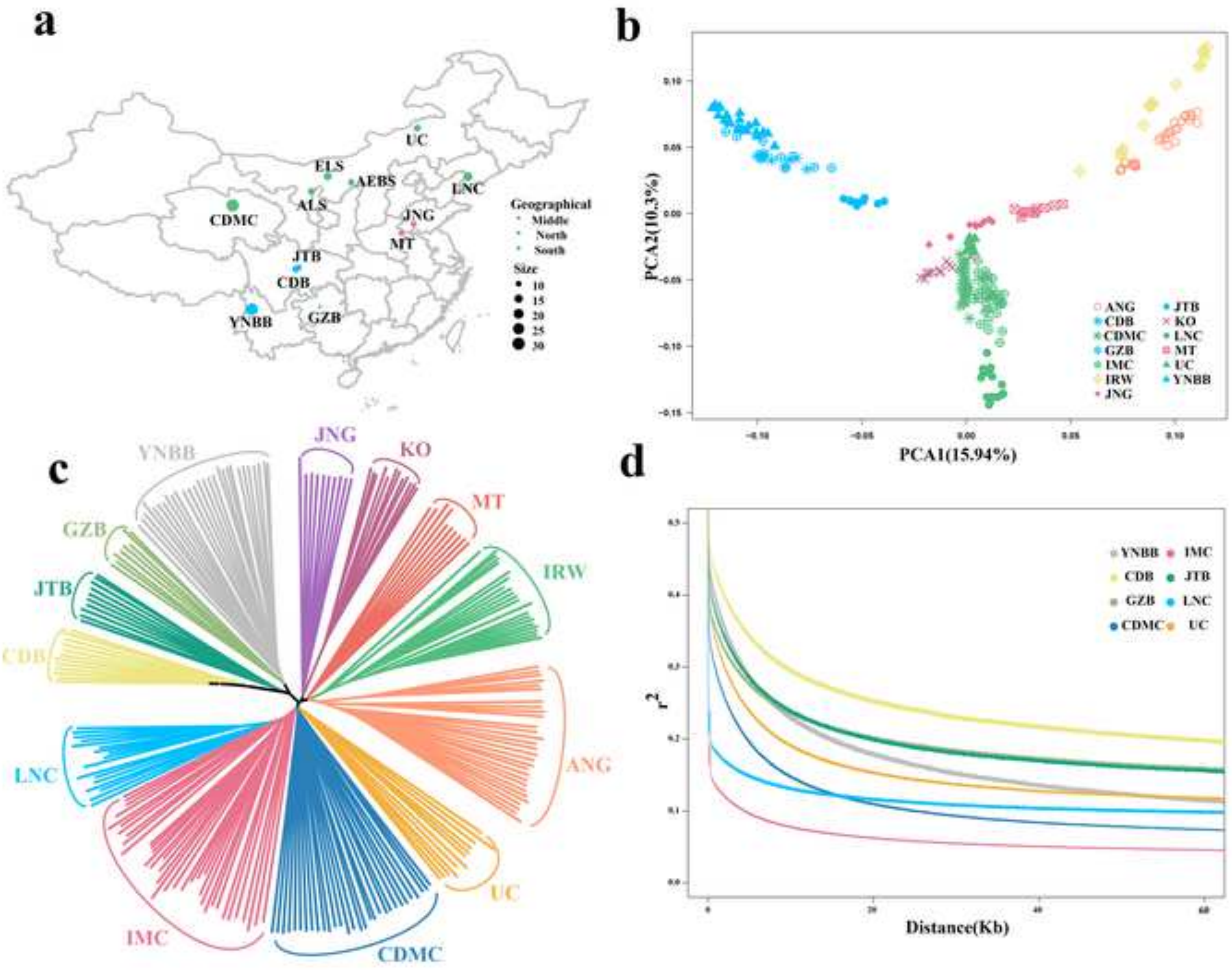

Figure
Click here to access/download;Figure;Fig. 1a.tiff

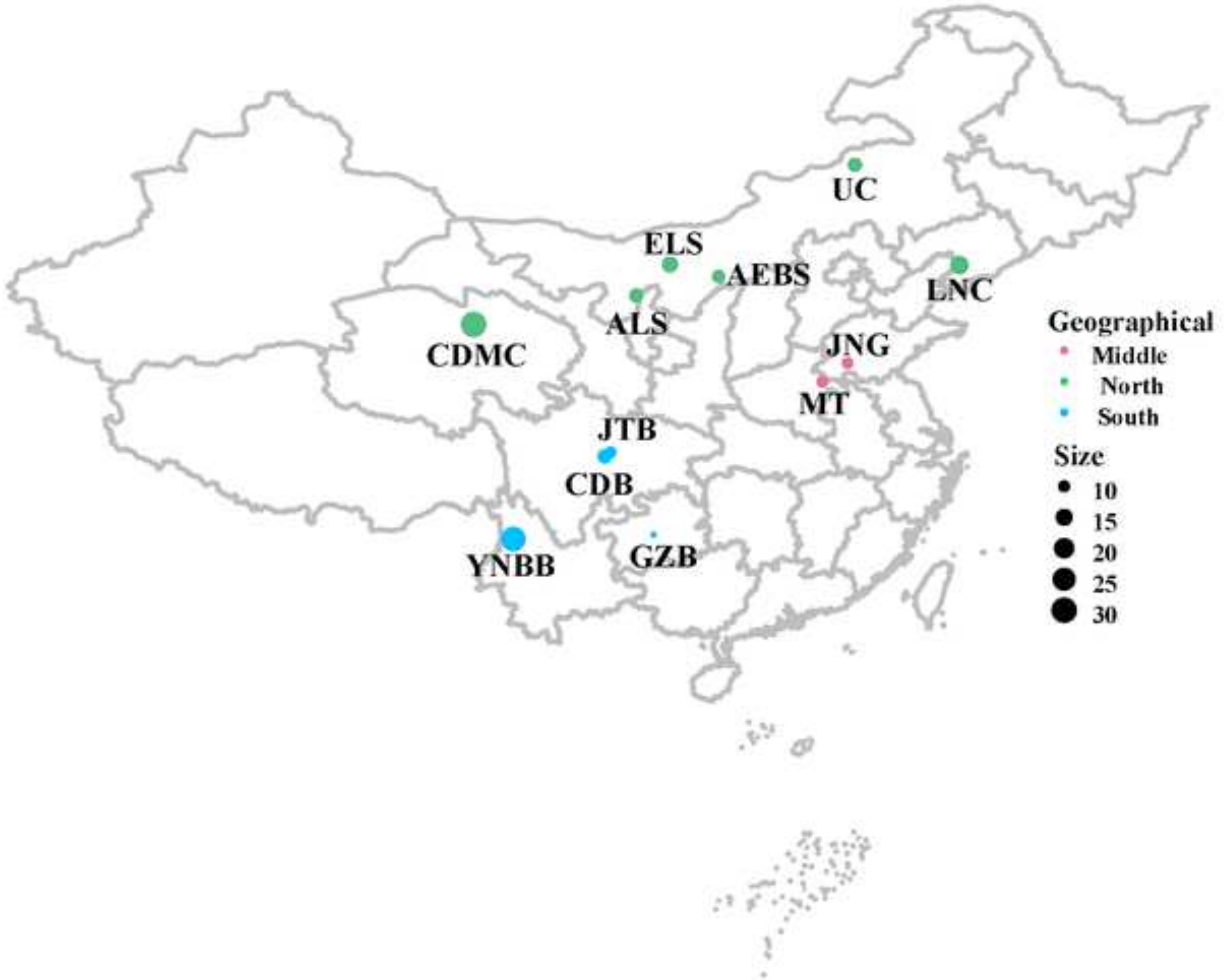

Click here to access/download;Figure;Fig. 1b.tiff

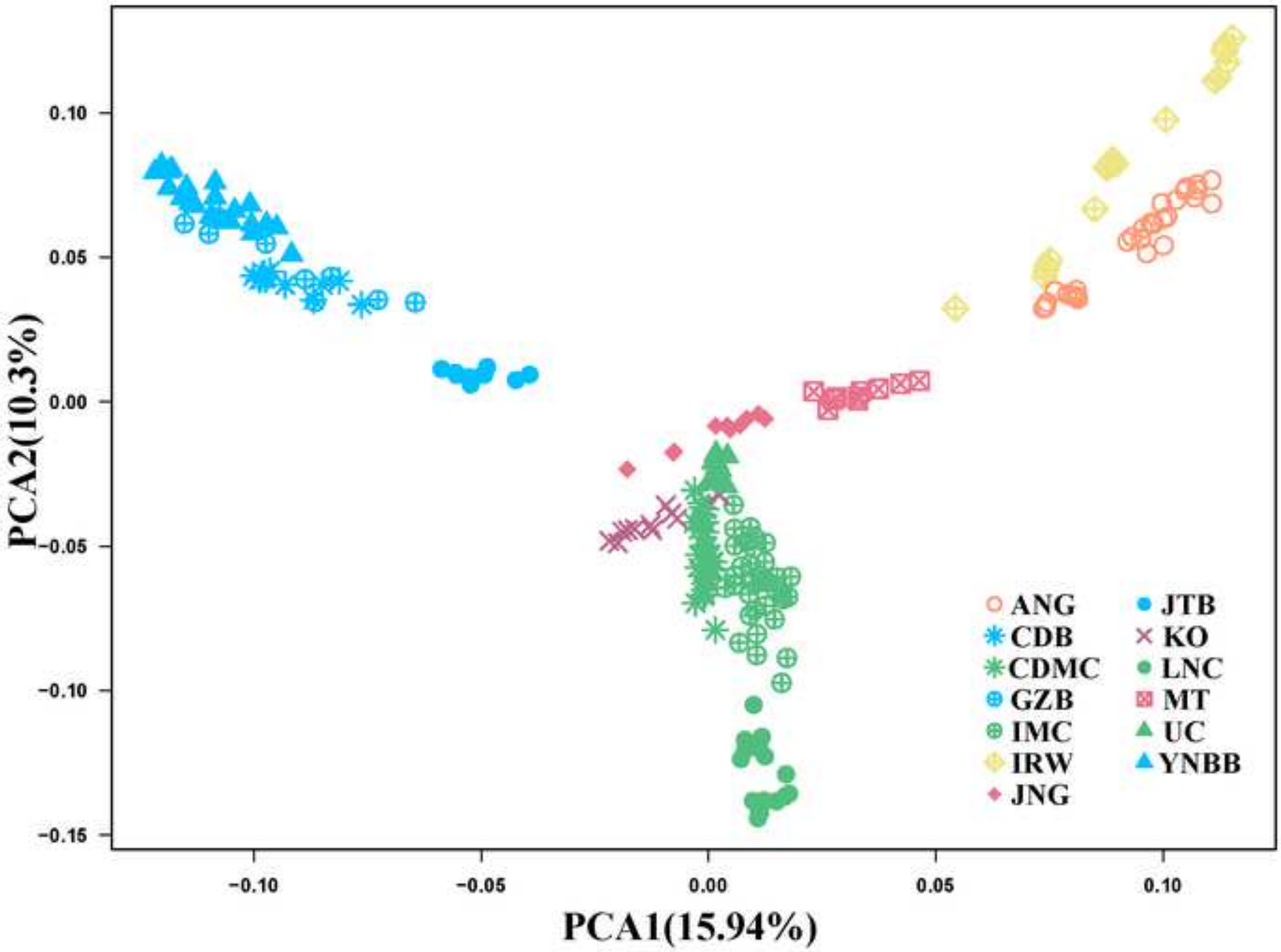

Figure Click here to access/download;Figure;Fig. 1c.tif

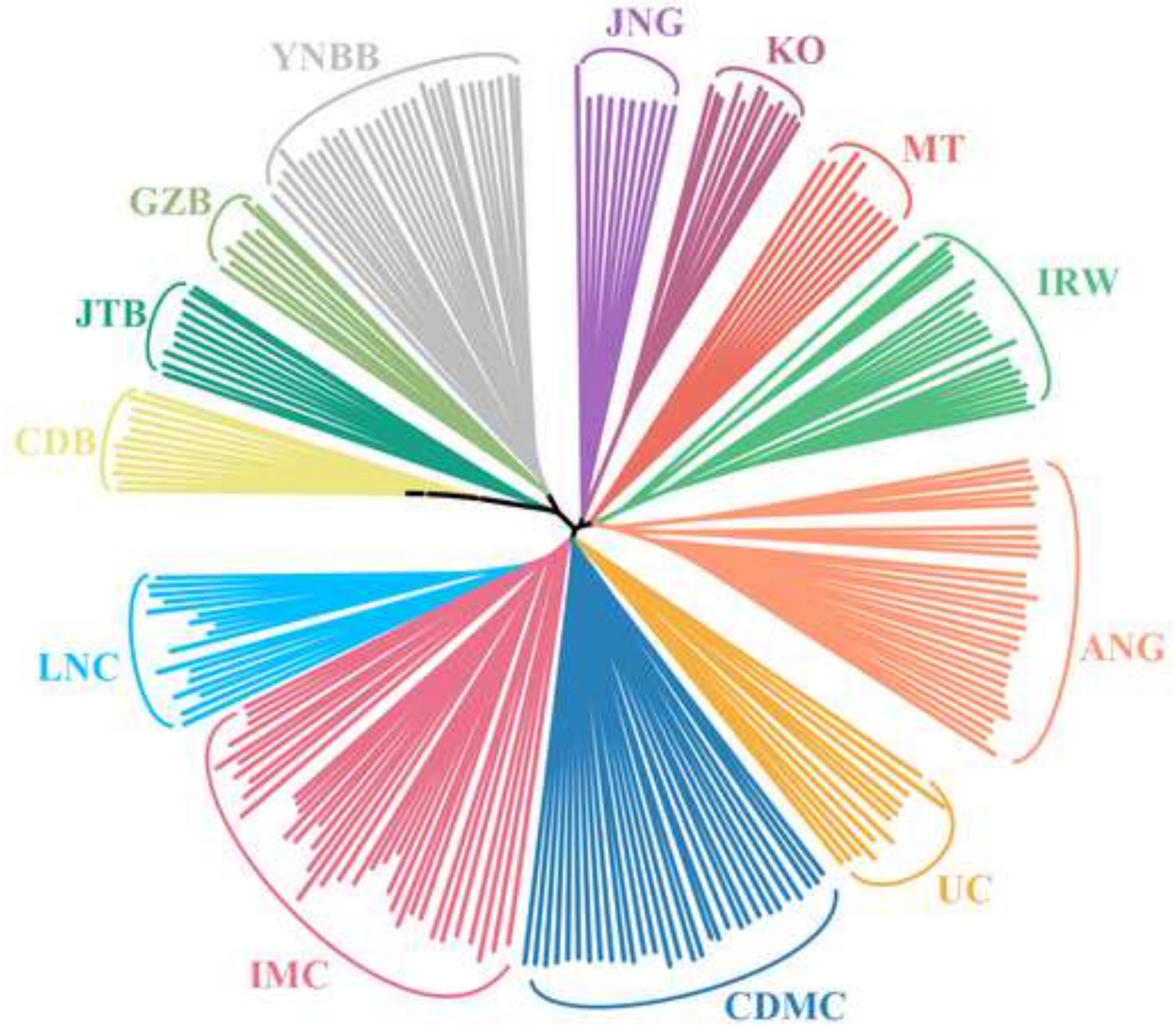

Figure
Click here to access/download;Figure;Fig. 1d.tiff

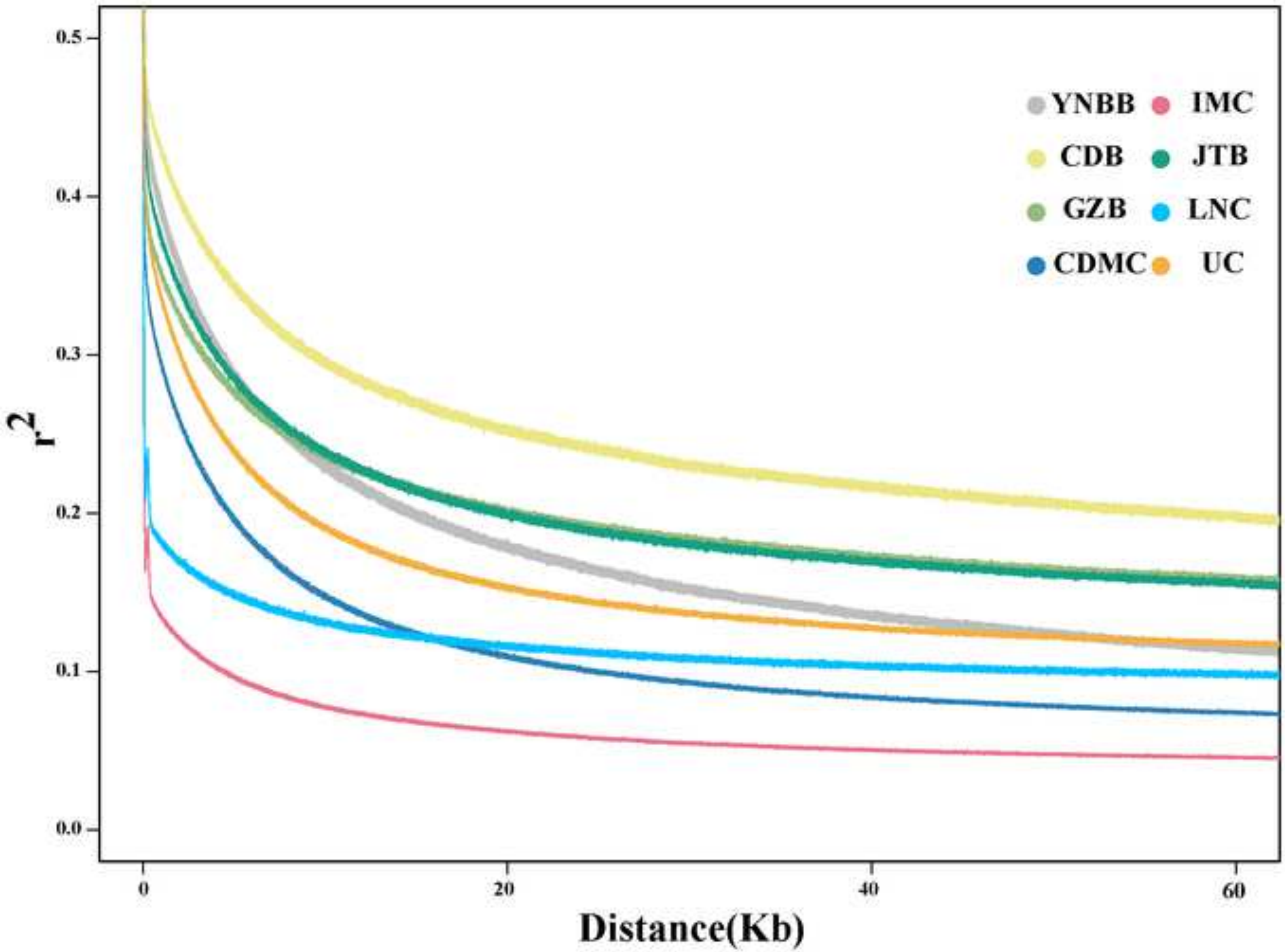

Figure
Click here to access/download;Figure;Fig. 2.tif

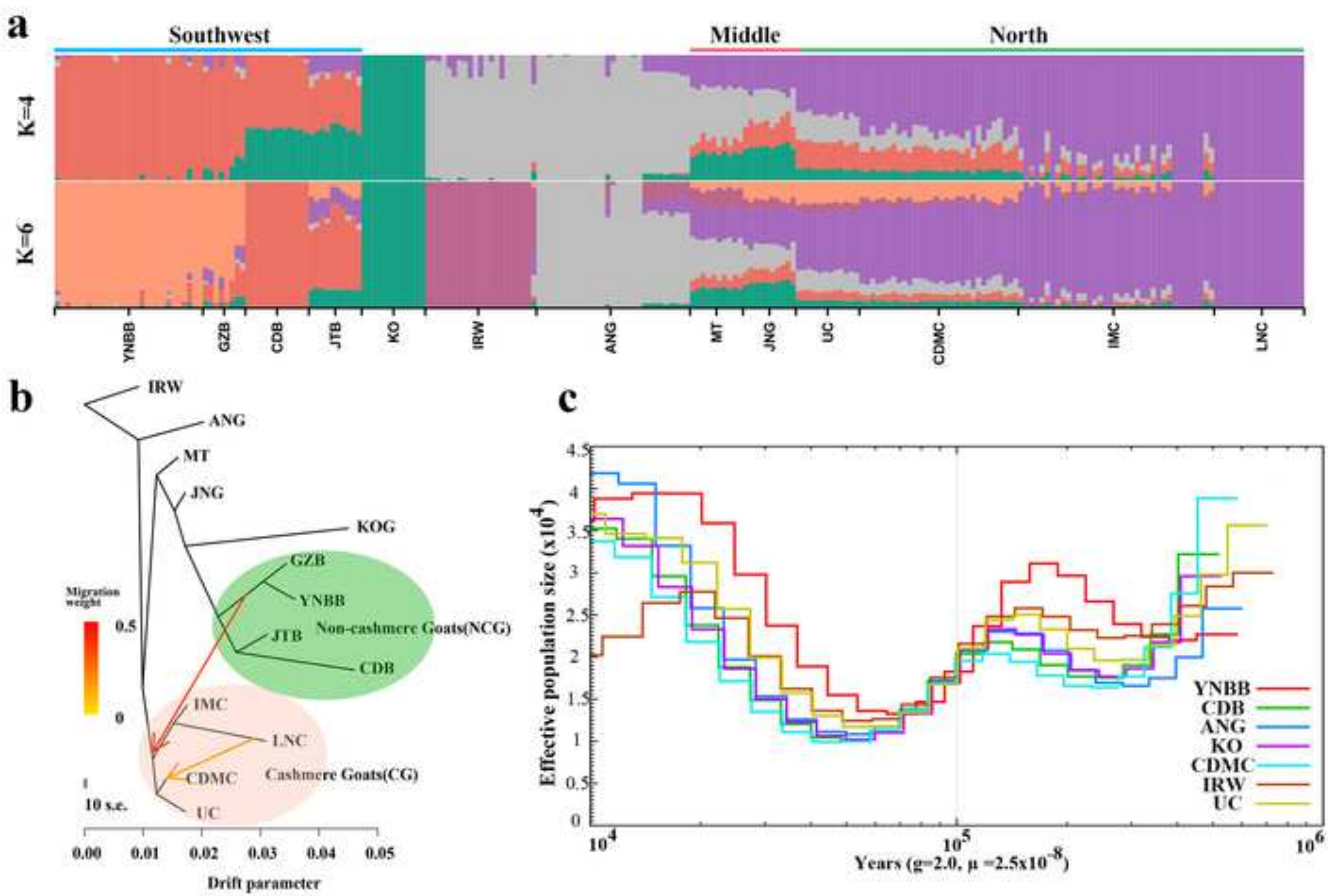

Figure

Click here to access/download;Figure;Fig. 2a.tif

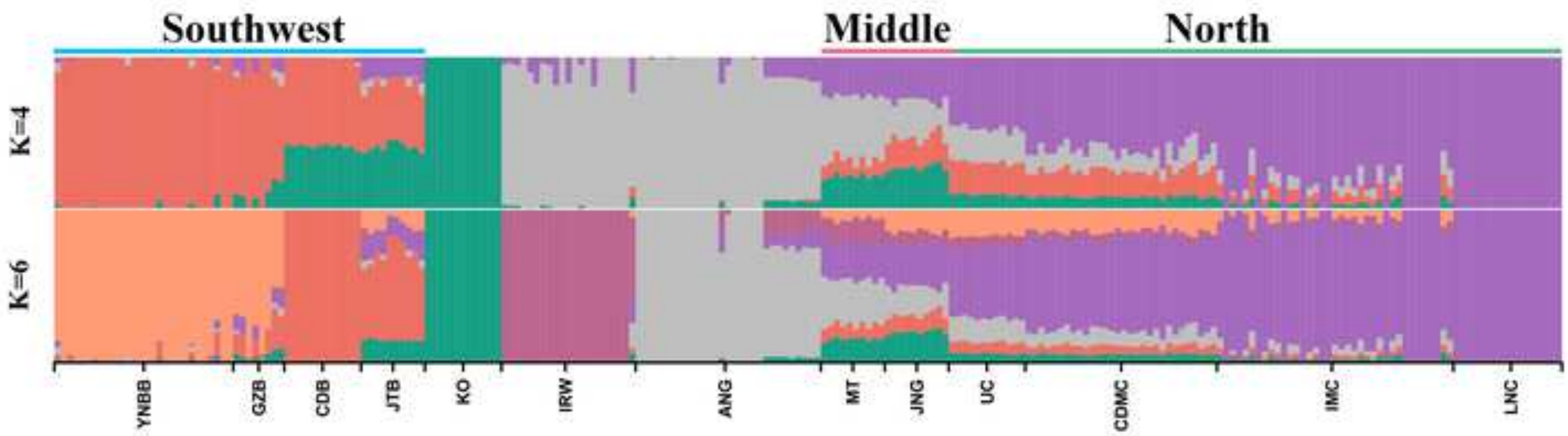

 Click here to access/download;Figure;Fig. 2b.tiff

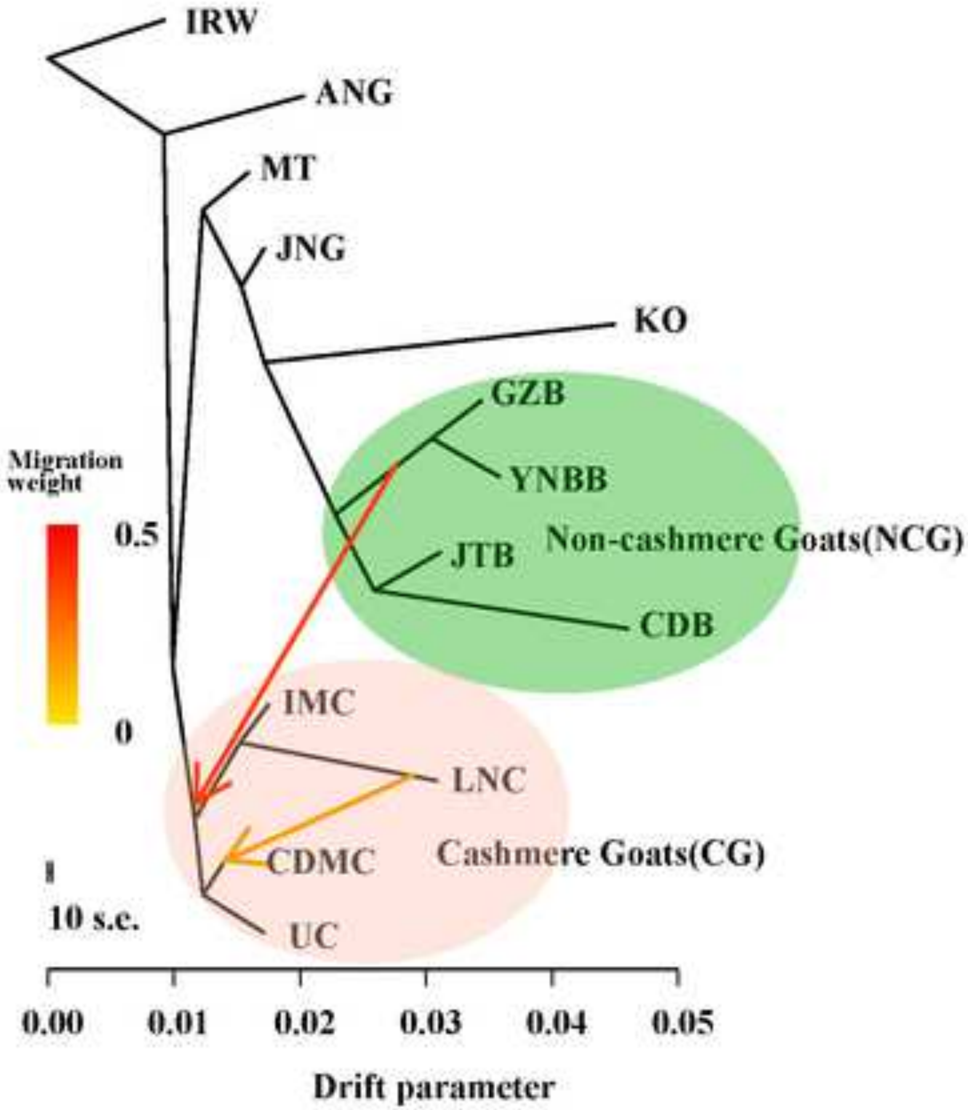

Click here to access/download;Figure;Fig. 2c.tif

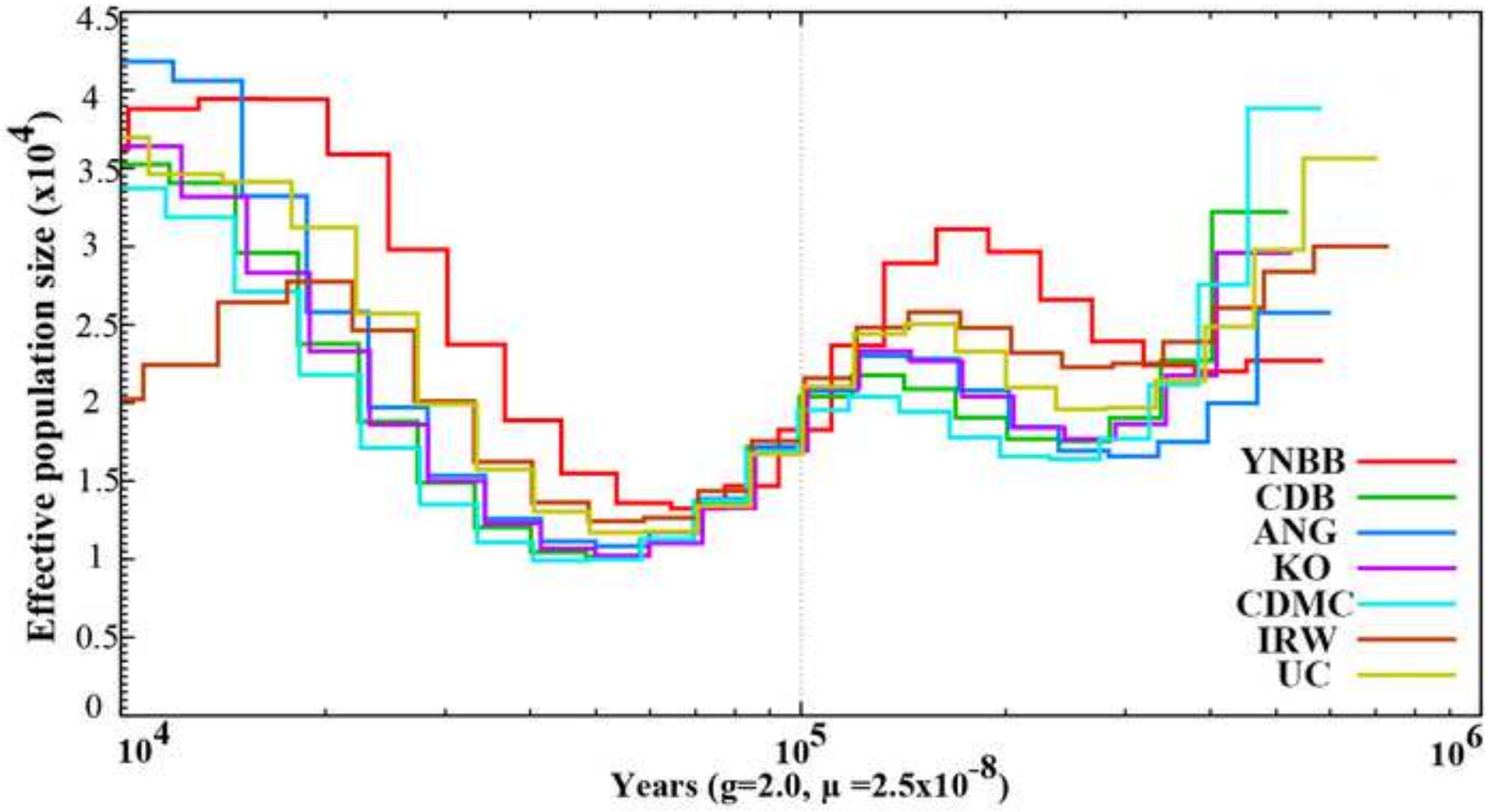

Figure

Click here to access/download;Figure;Fig. 3.jpg

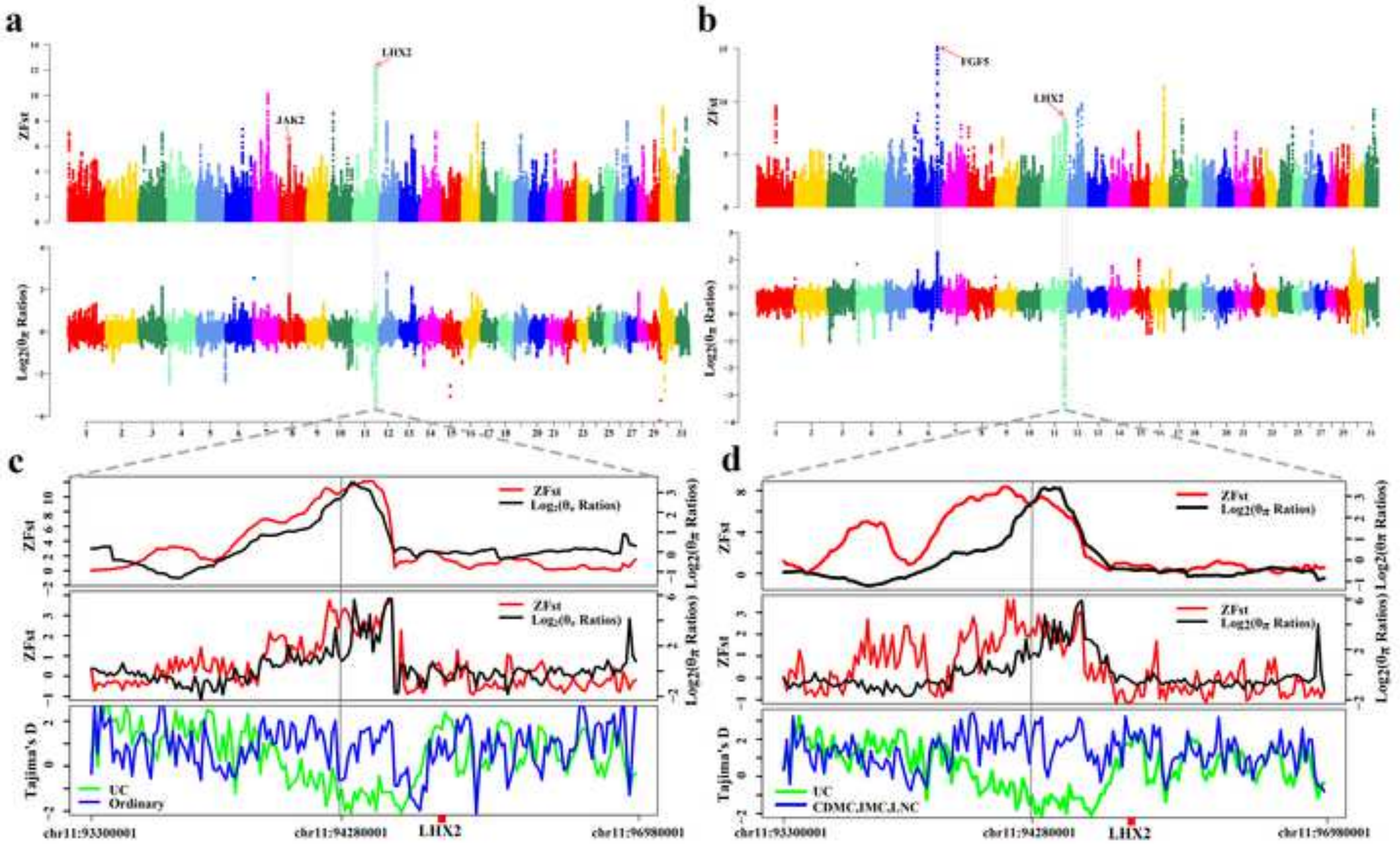

Click here to access/download;Figure;Fig. 3a.tiff

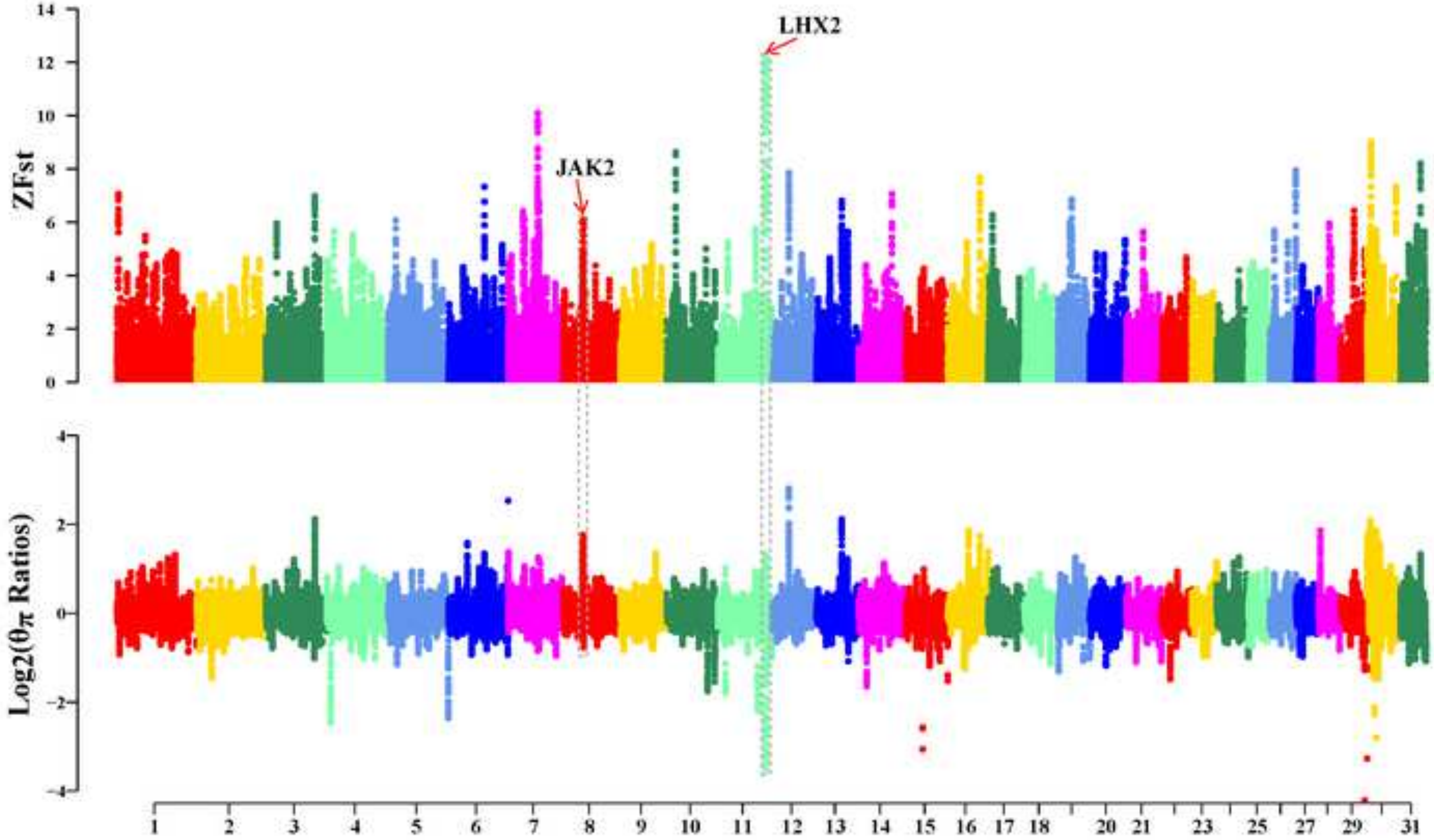

 Click here to access/download;Figure;Fig. 3b.tif

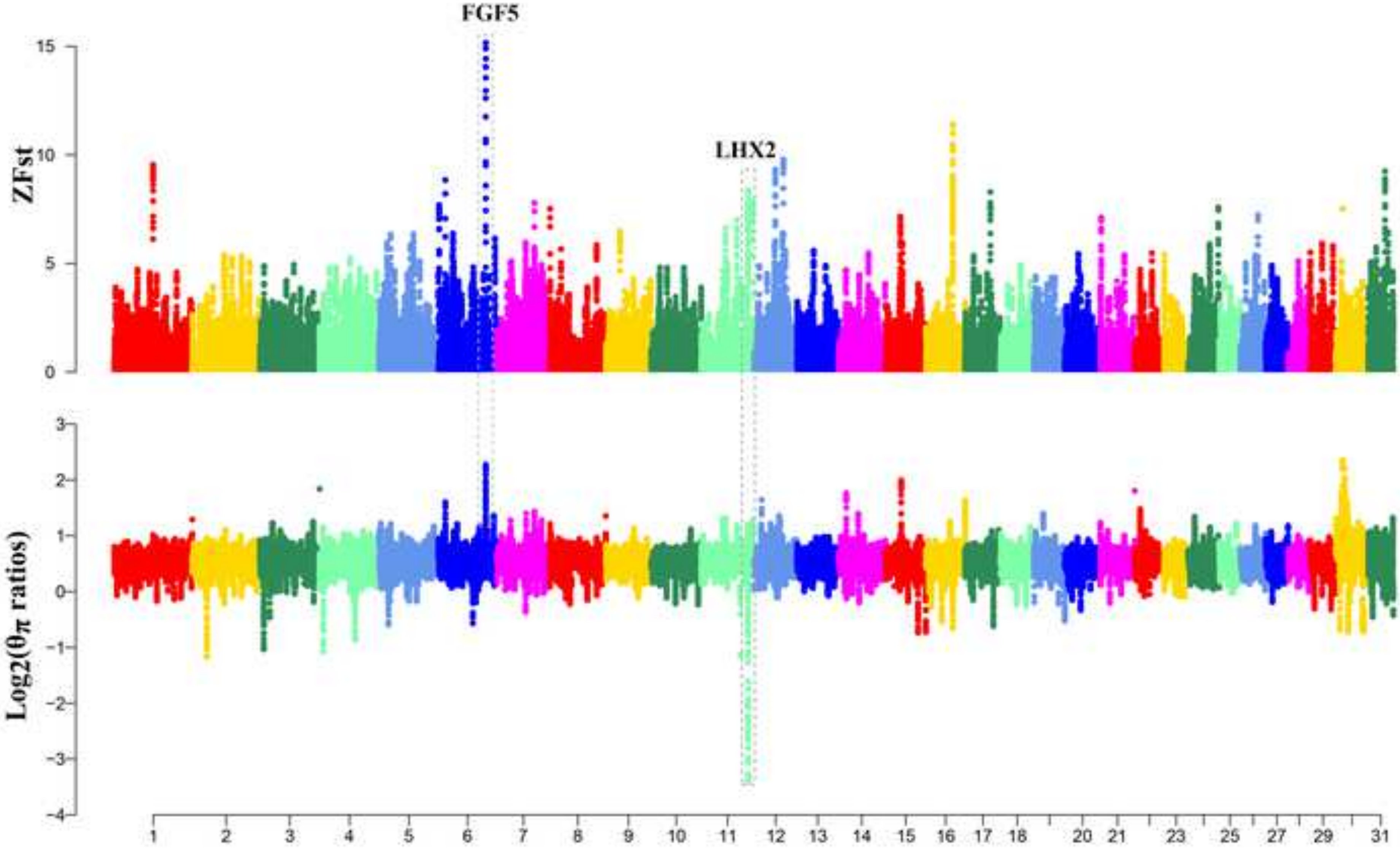

Figure

Click here to access/download;Figure;Fig. 3c.tif

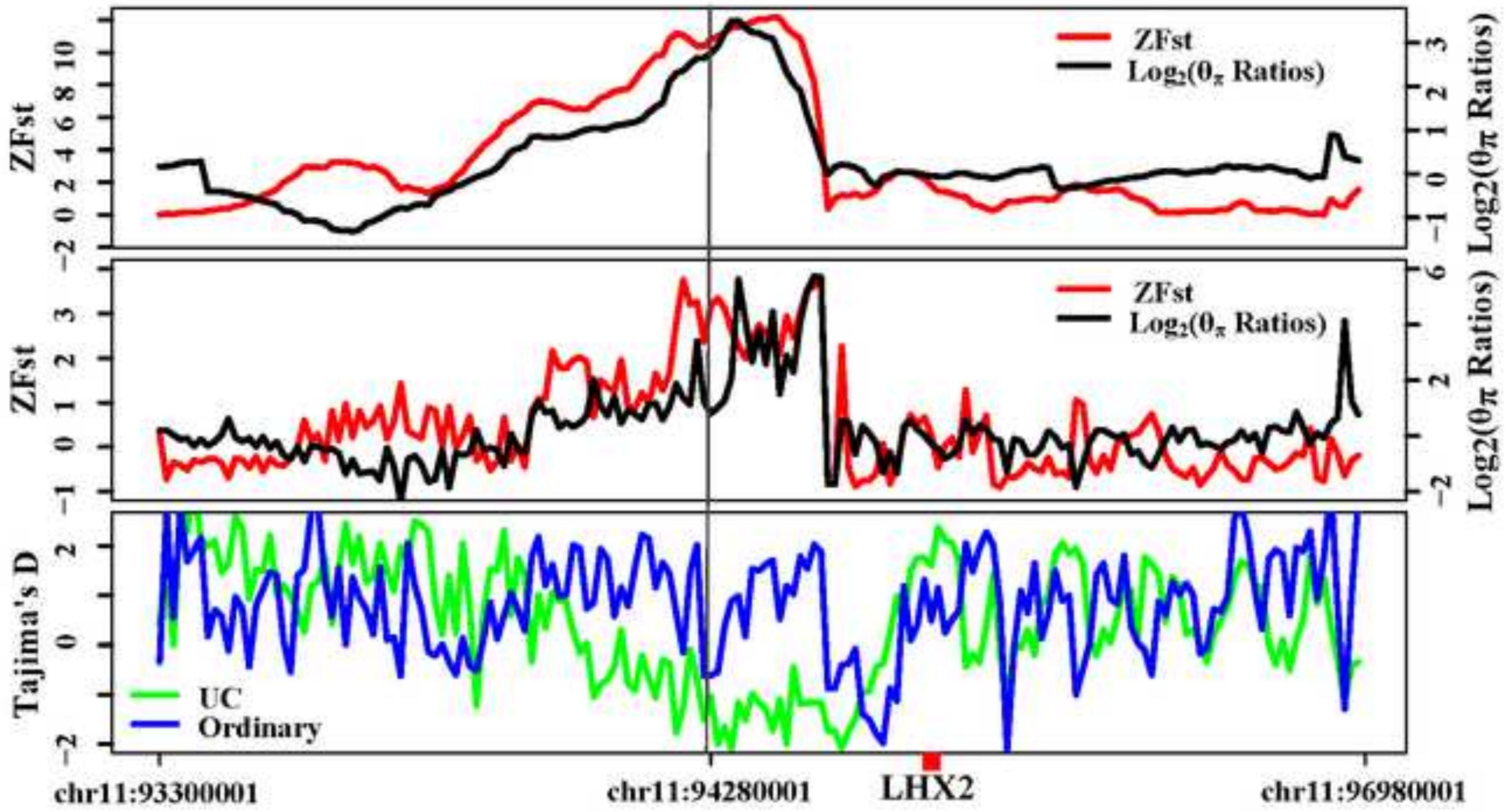

Click here to access/download;Figure;Fig. 3d.tiff

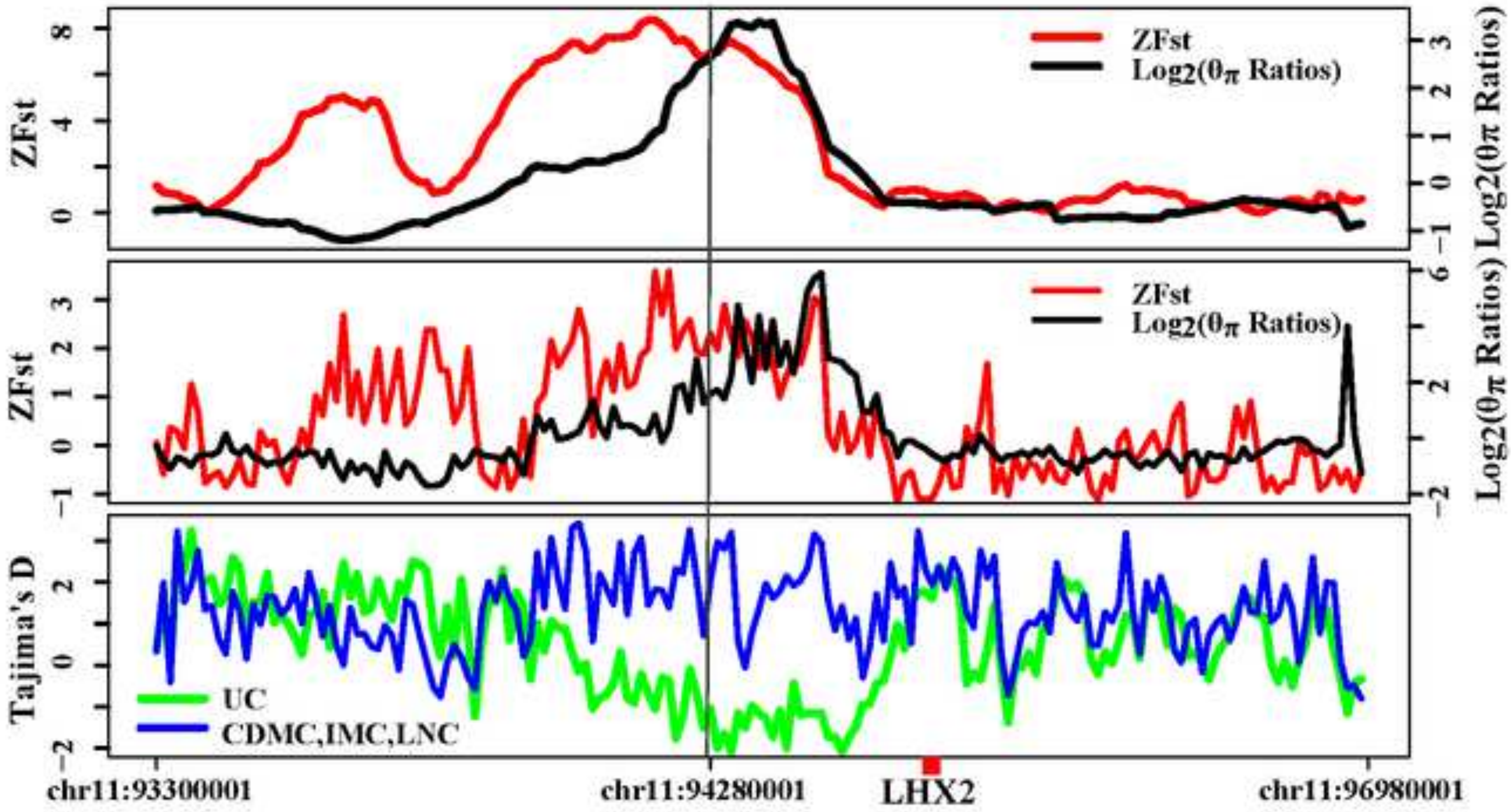

Figure
Click here to access/download;Figure;Fig. 4.tiff

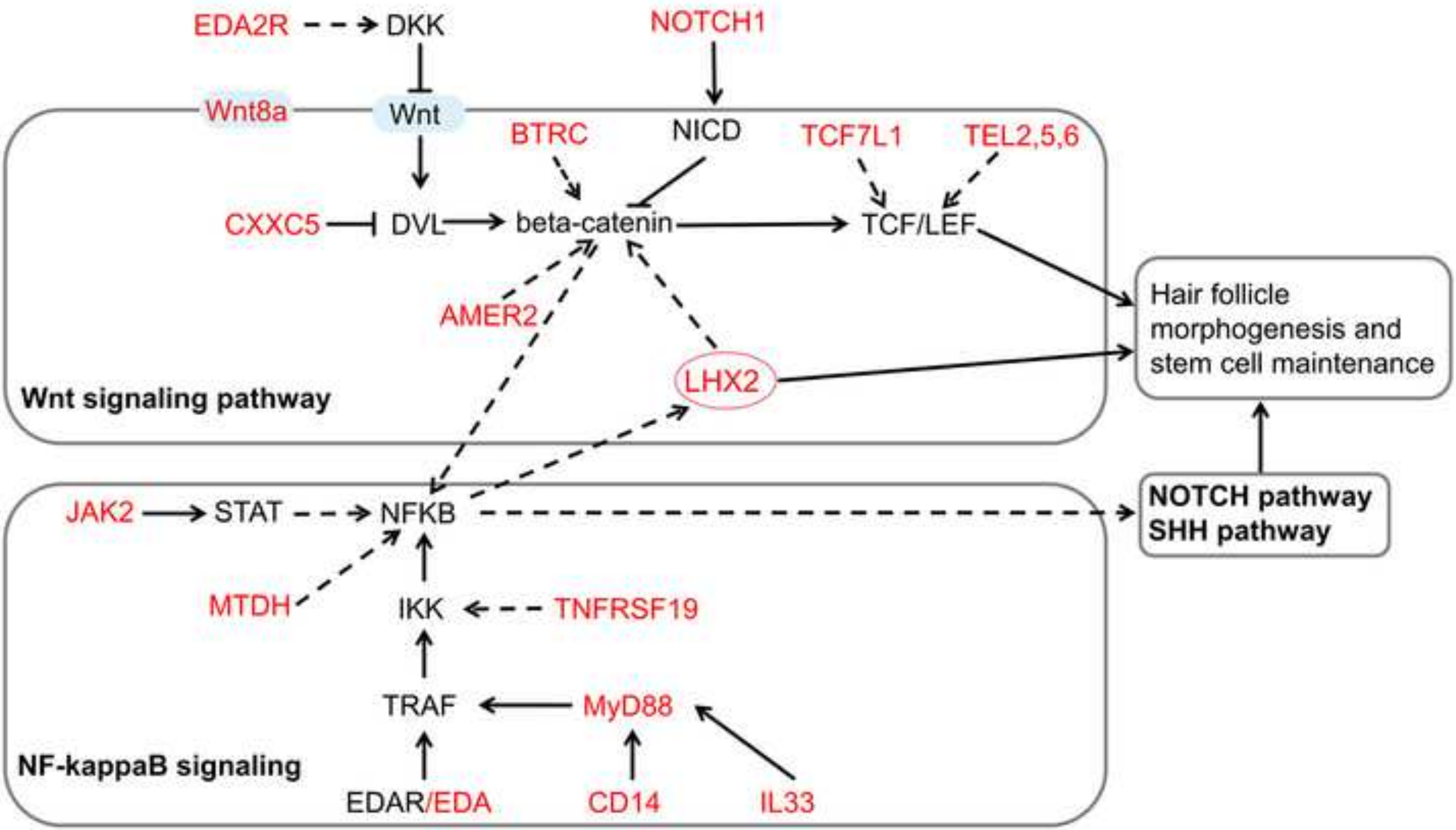

Click here to access/download;Figure;Fig. 5.tif

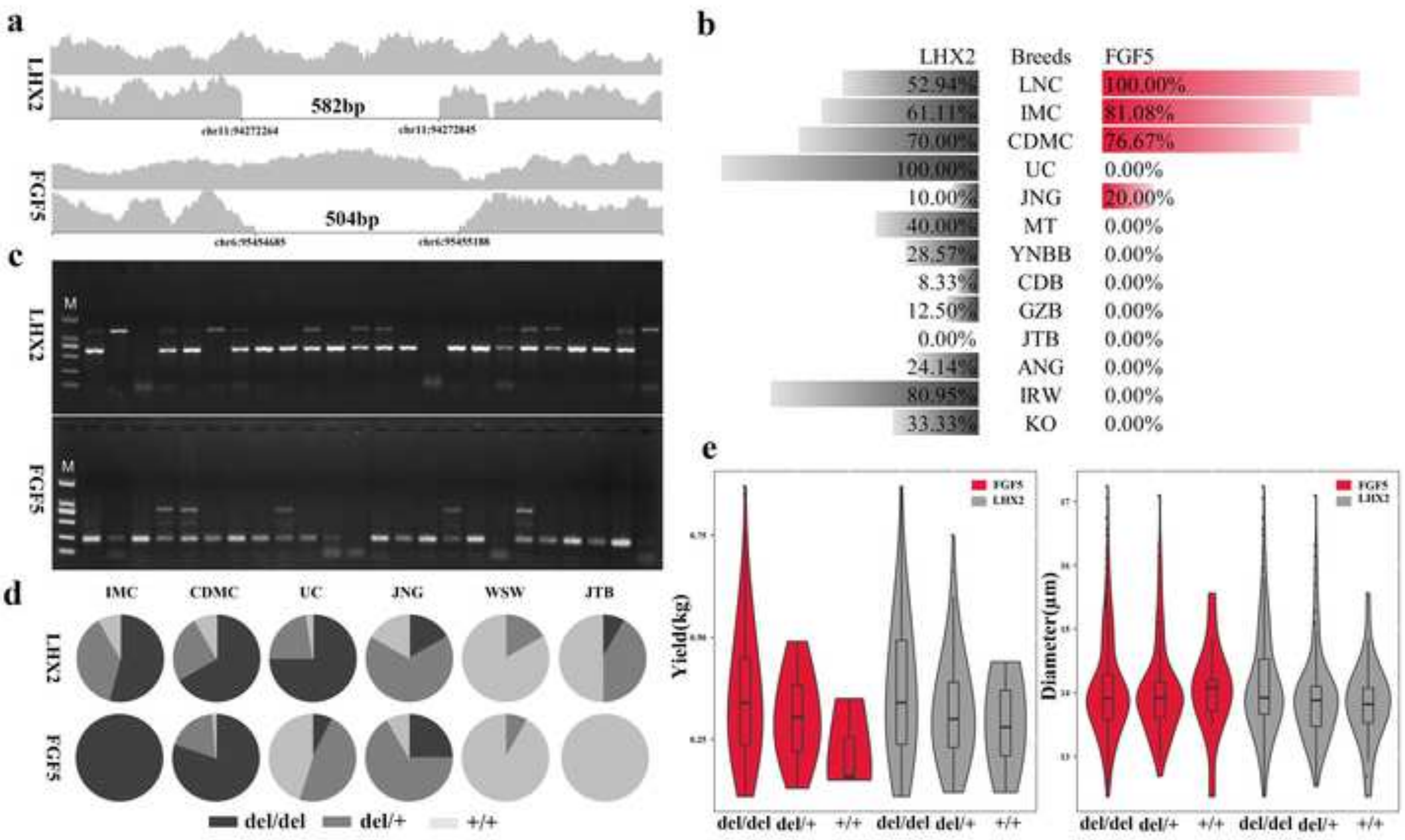

Figure

Click here to access/download;Figure;Fig. 5a.tif

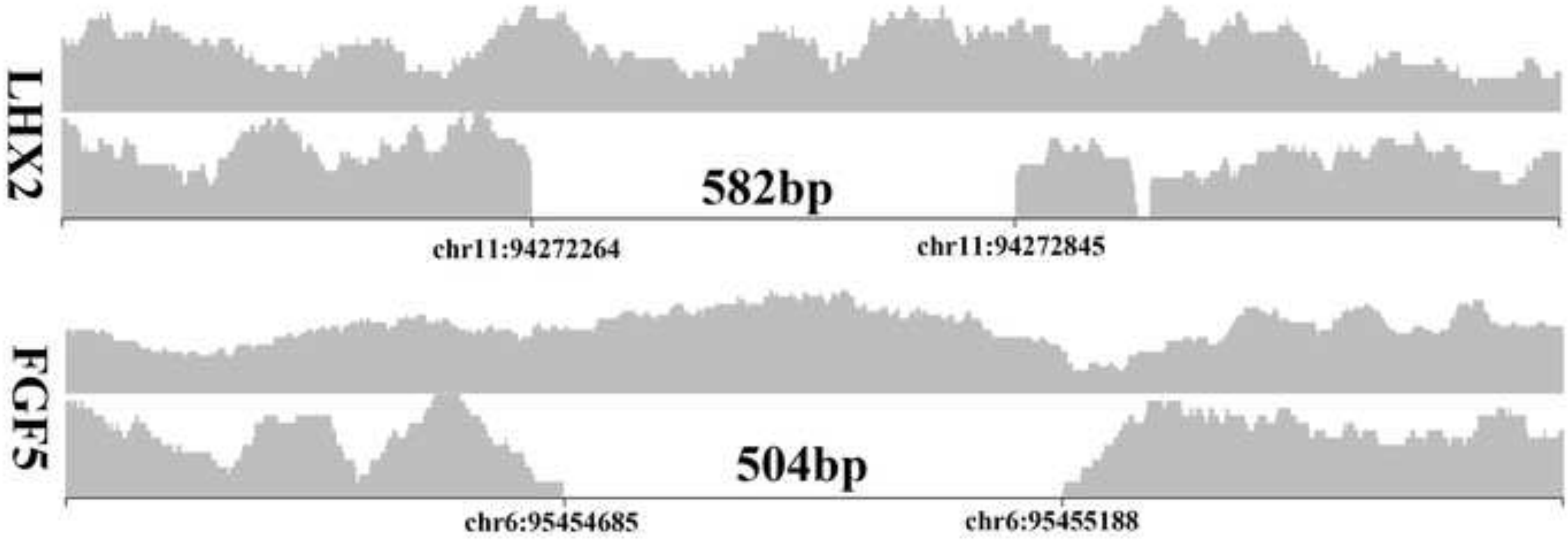

Figure Click here to access/download;Figure;Fig. 5b.tif

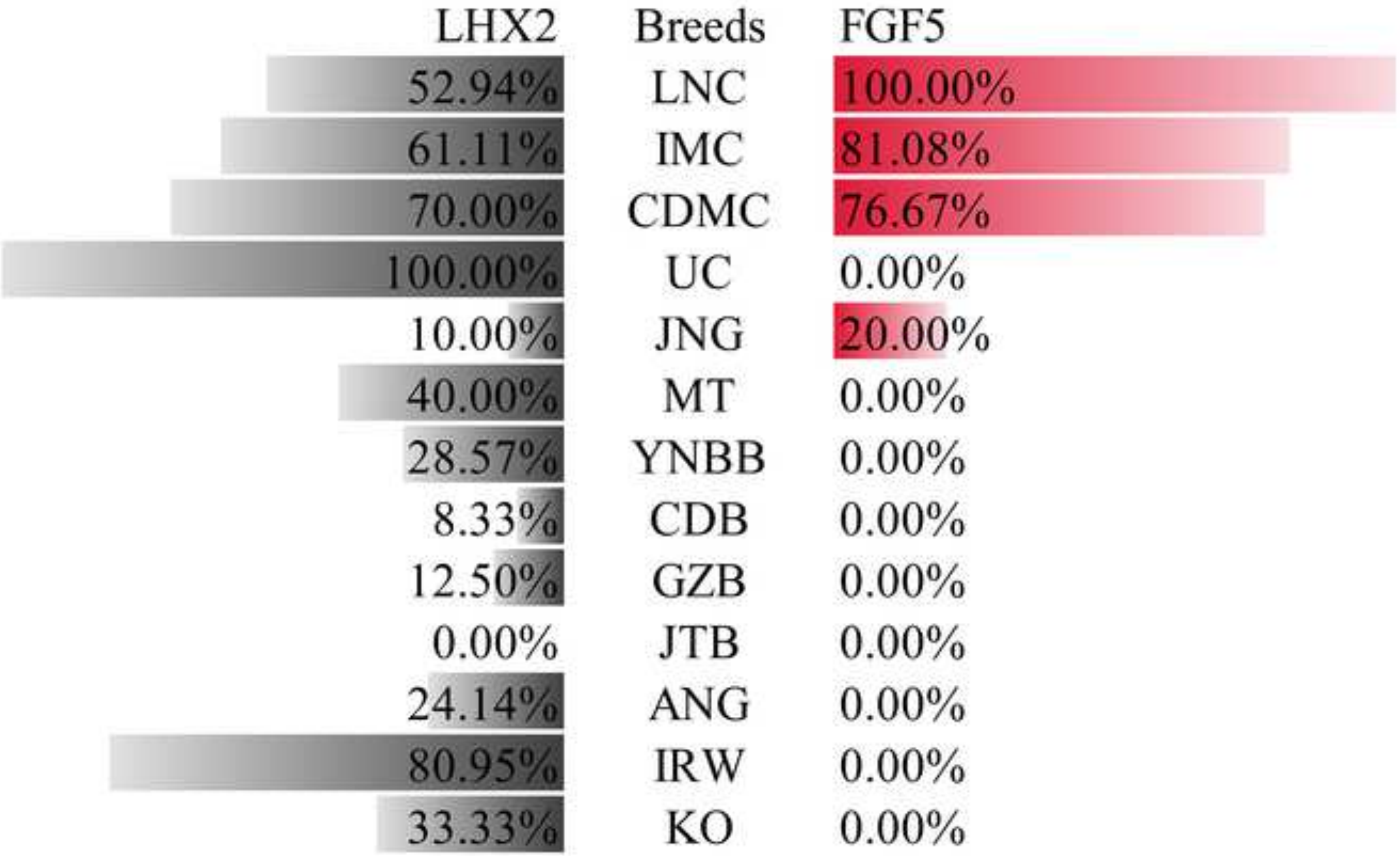

Click here to access/download;Figure;Fig. 5c.tif

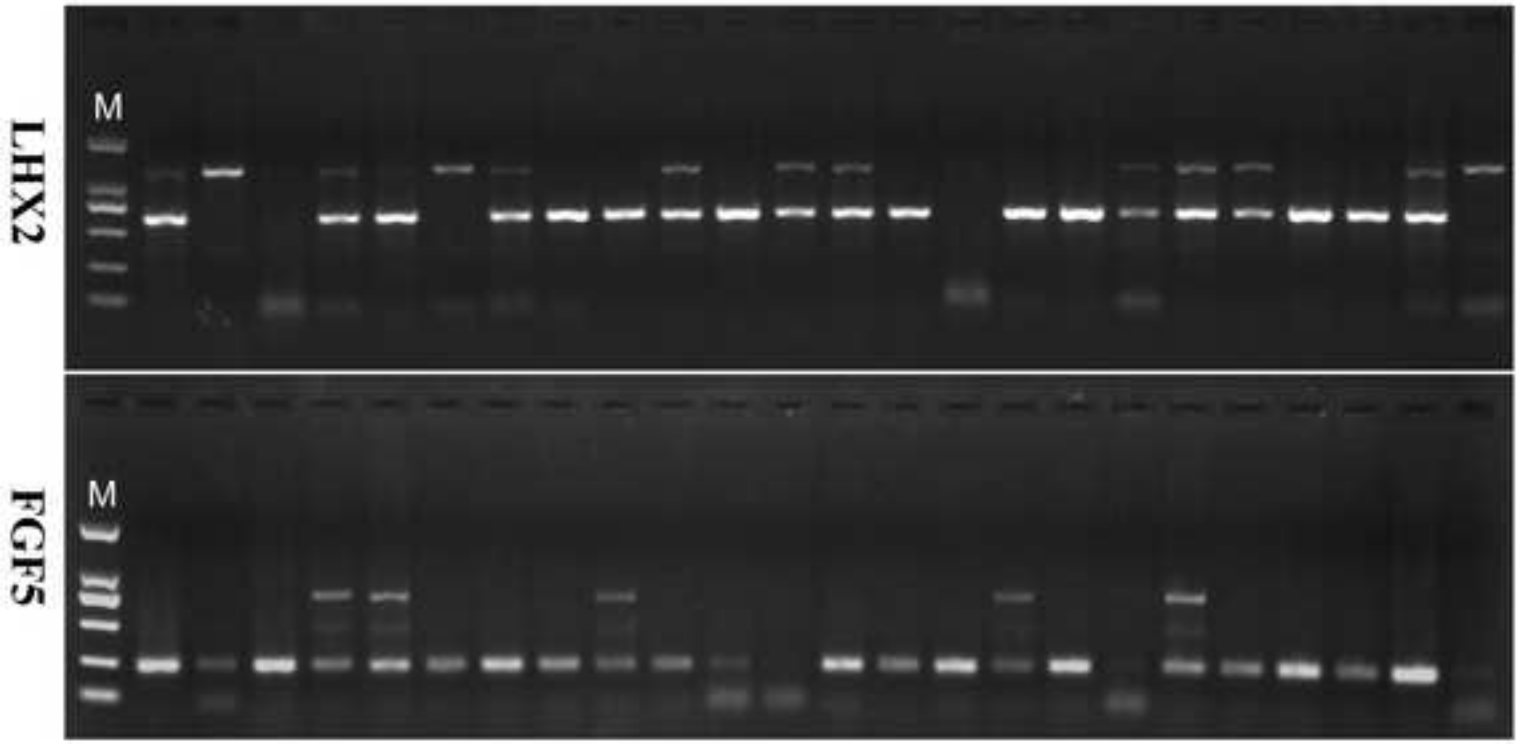

Click here to access/download;Figure;Fig. 5d.tif

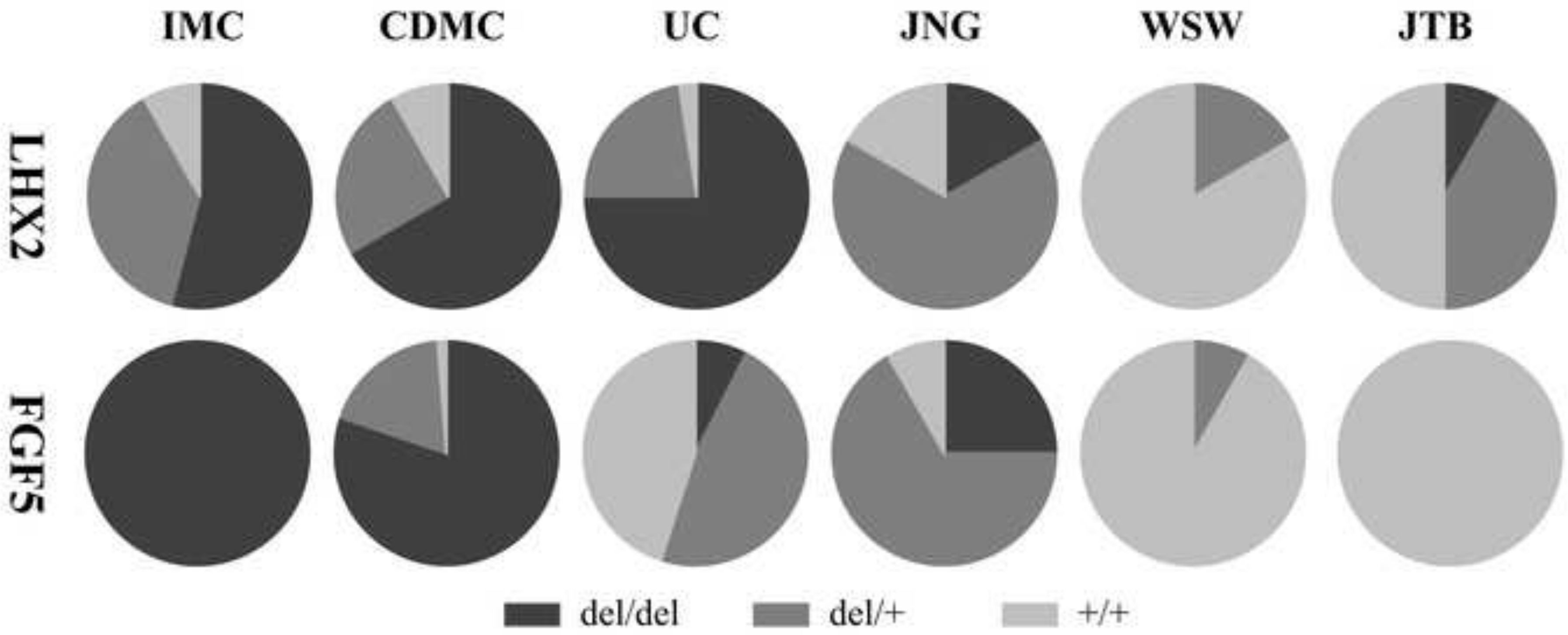

Click here to access/download;Figure;Fig. 5e.tiff

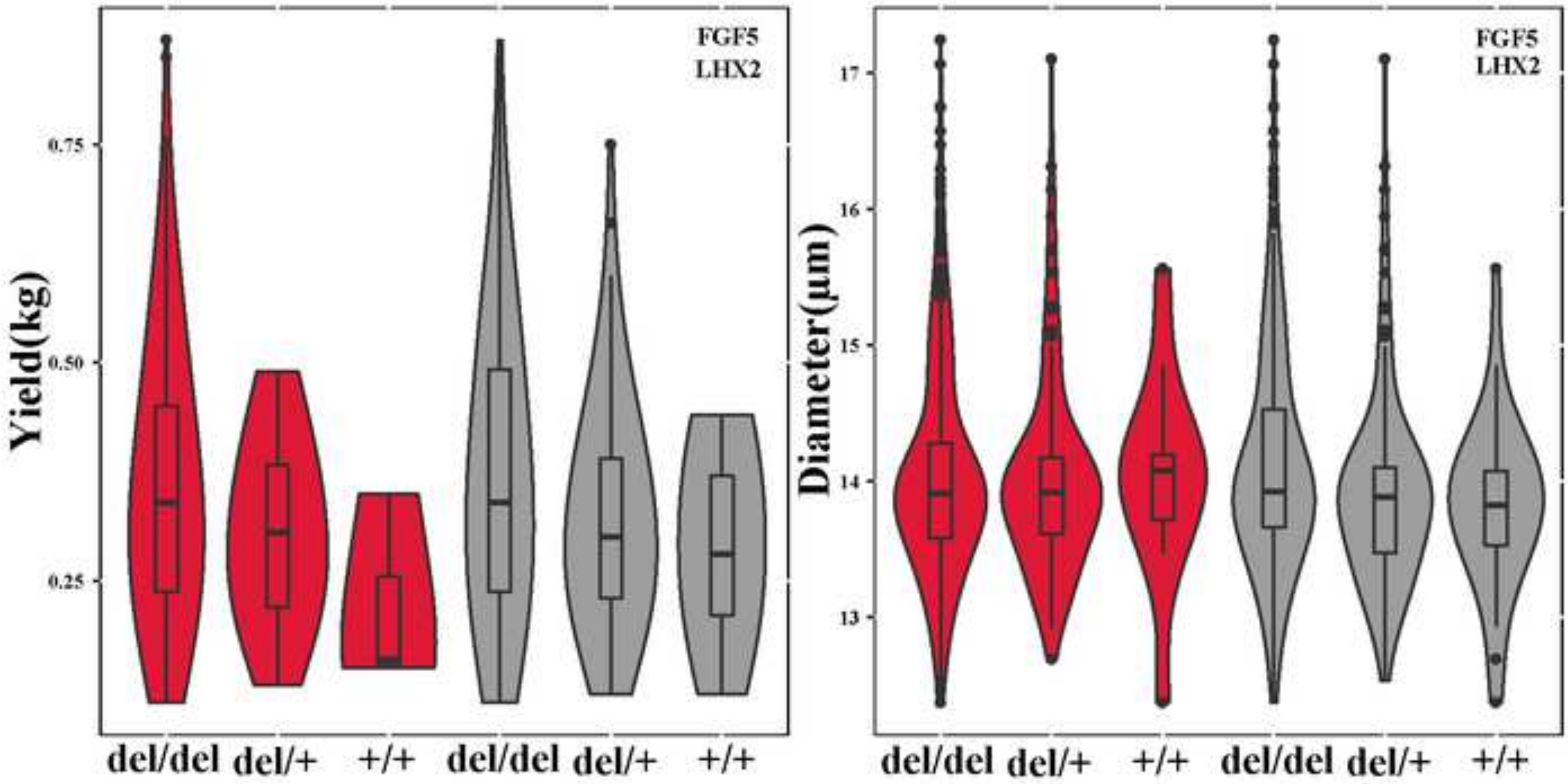

Figure

Click here to access/download;Figure;Fig. 6.tif

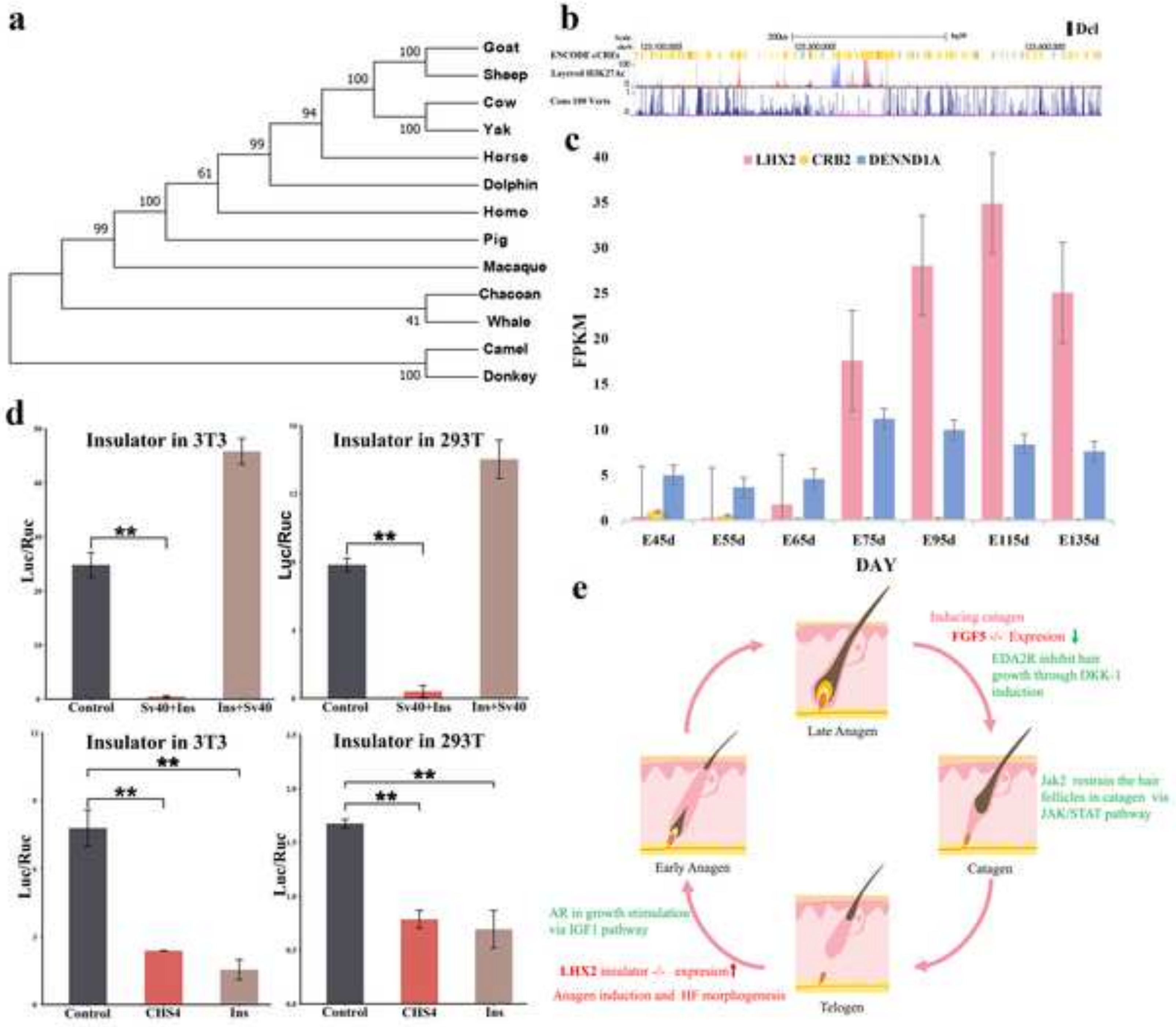

Figure Click here to access/download;Figure;Fig. 6a.tiff

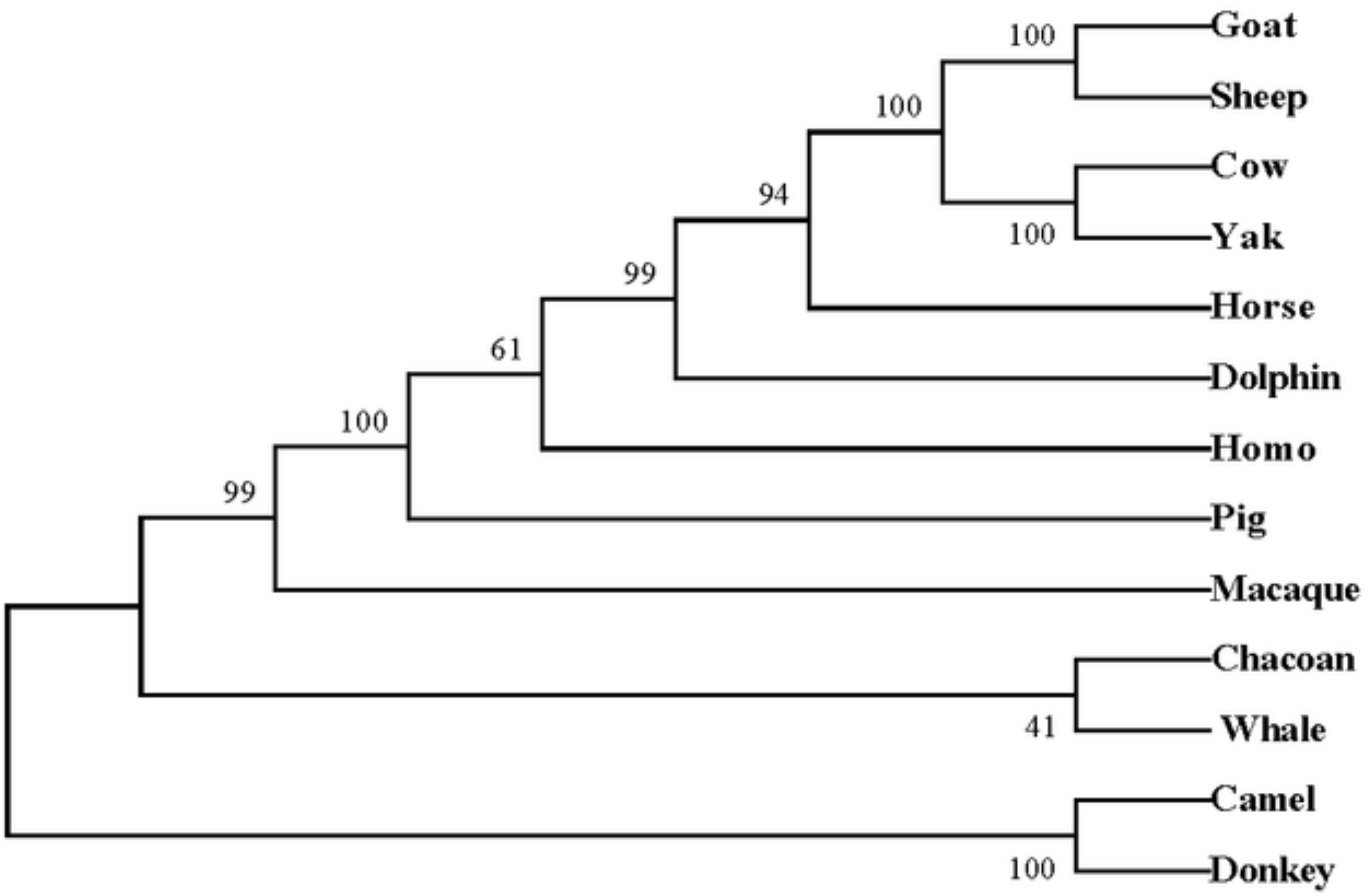

Click here to access/download;Figure;Fig. 6b.tiff

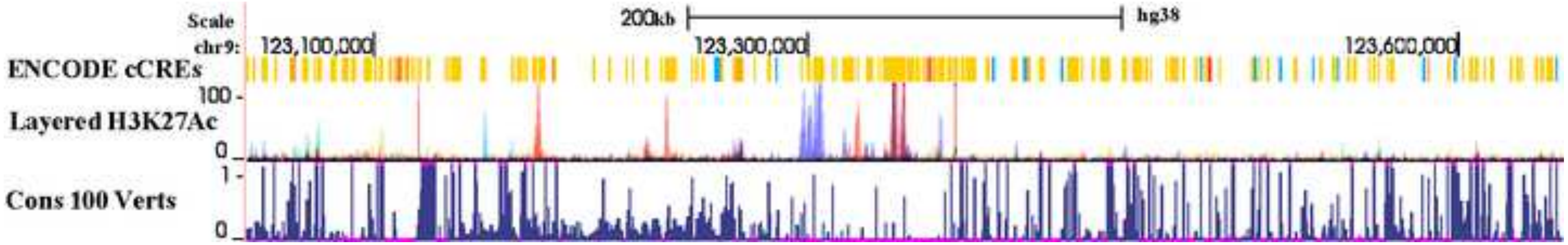

Click here to access/download;Figure;Fig. 6c.tiff

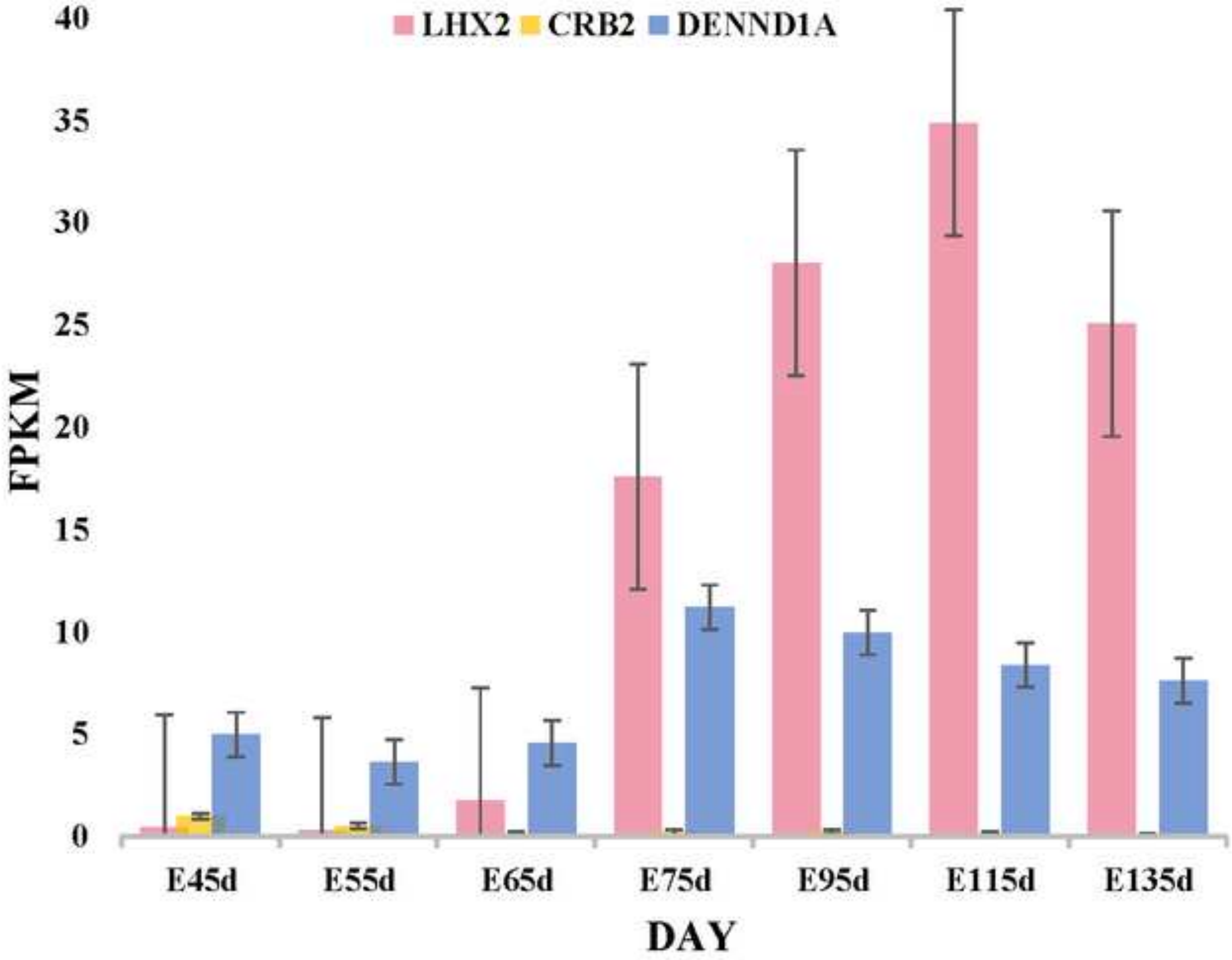

Figure Click here to access/download;Figure;Fig. 6d.tif

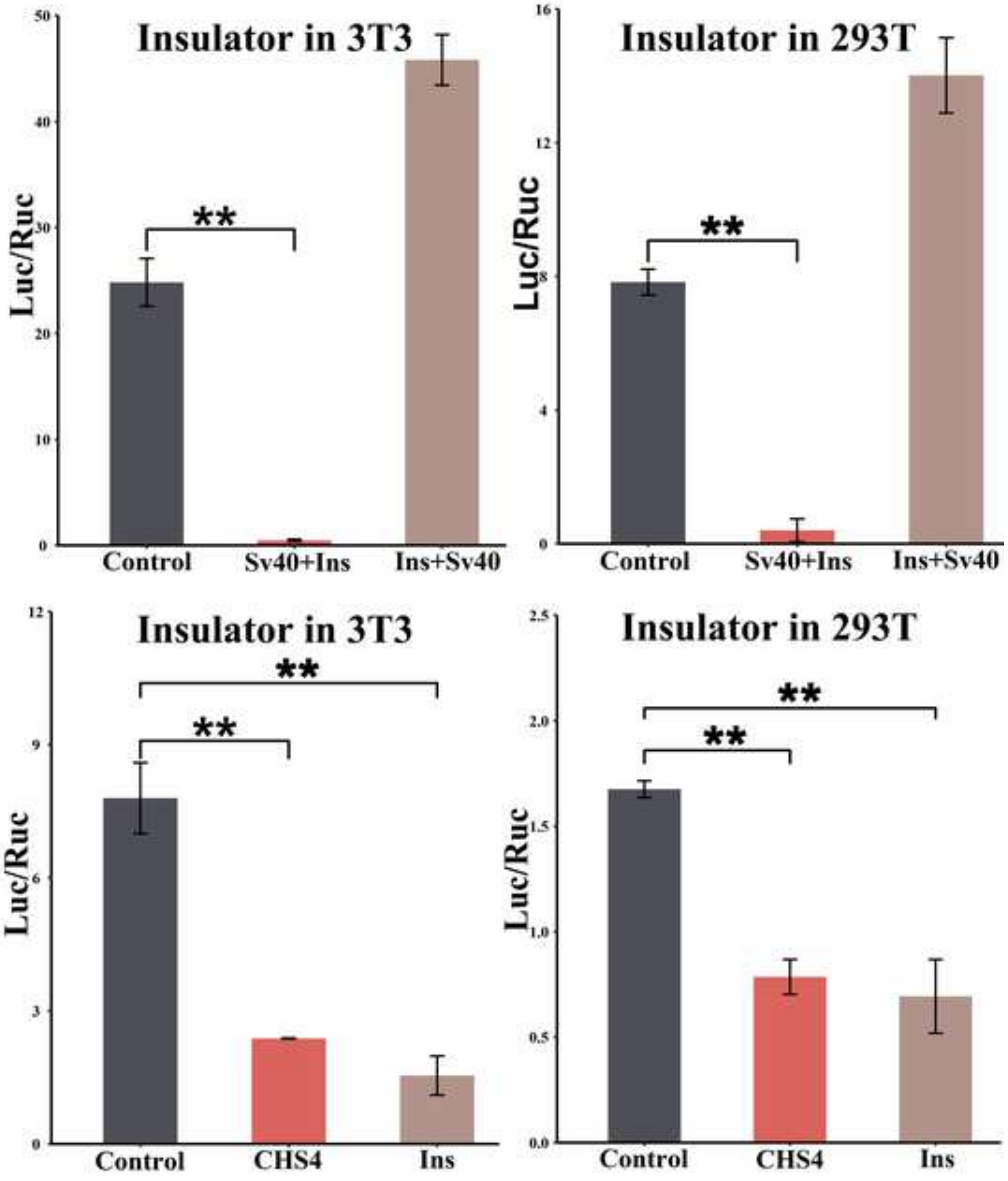

Figure Click here to access/download;Figure;Fig. 6e.tif

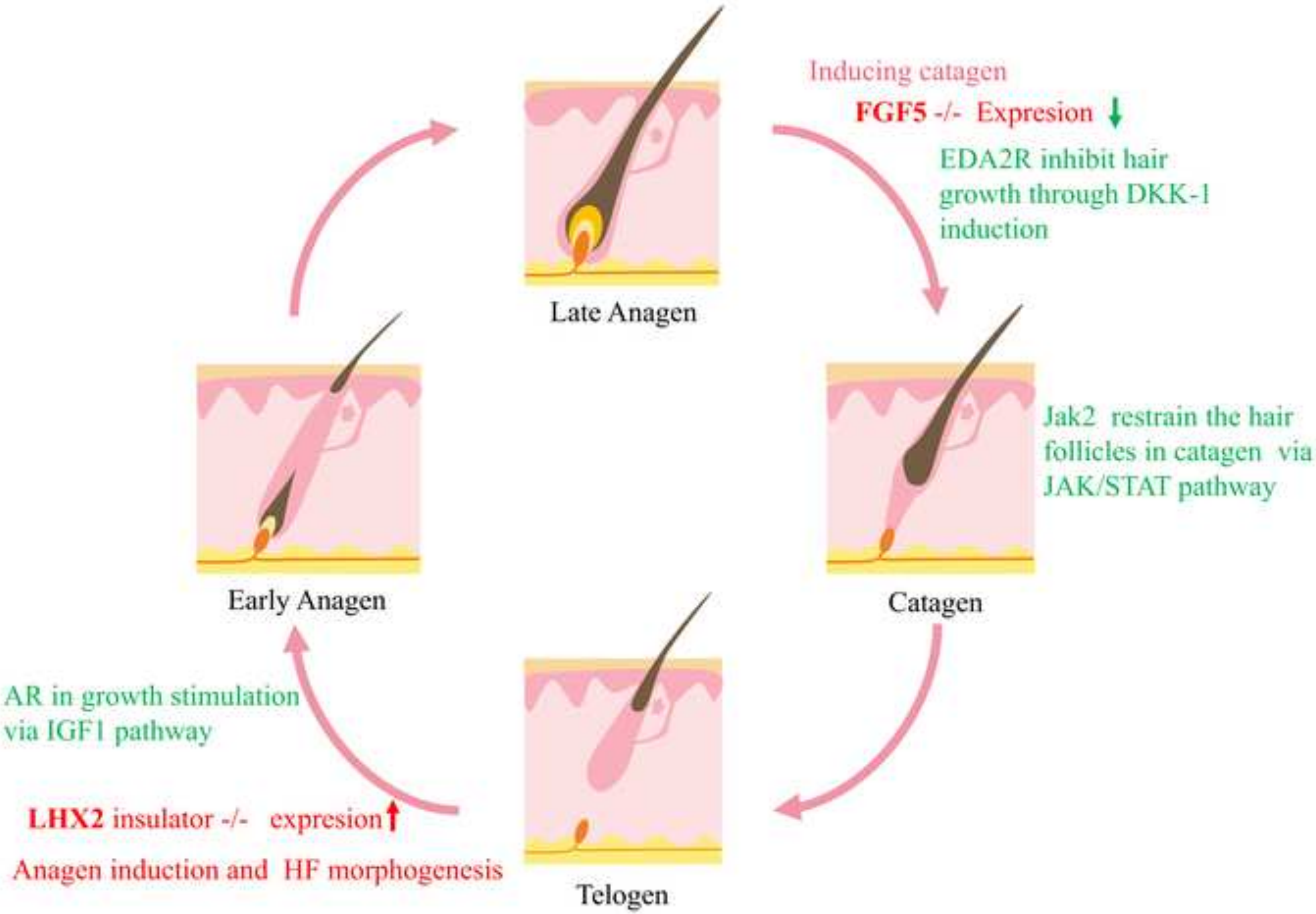

Click here to access/download
Supplementary Material
Revised-Supplementary.docx
