# Supplementary material for: Selection signatures in goats reveal a novel deletion mutant underlying cashmere yield and diameter
Source: Gigascience. 2022 Nov 3;11:giac107. doi: 10.1093/gigascience/giac107 (PMC9633279; doi:10.1093/gigascience/giac107)
Supplement: giac107_Supplemental_Files [file giac107_supplemental_files.zip › Revised-Supplementary.docx]

**Selection signatures in goats reveal a novel deletion mutant underlying cashmere yield and diameter**

**Content**

[**Supplementary Notes 1** 4](#_Toc106080422)

[**1.1 Sample information and sequencing** 4](#_Toc106080423)

[1.2 Quality control and reads mapping 4](#_Toc106080424)

[**Supplementary Notes 2**: **Population structure and phylogenetic analysis** 5](#_Toc106080425)

[2.1 Geographic distribution mapping 5](#_Toc106080426)

[2.2 Principal component analysis (PCA) 5](#_Toc106080427)

[2.3 Population admixture 5](#_Toc106080428)

[2.4 Phylogenetic tree 6](#_Toc106080429)

[2.5 LD decay 6](#_Toc106080430)

[**Supplementary Notes 3:** **Gene flows and effective population size** 6](#_Toc106080431)

[3.1 Gene flow 7](#_Toc106080432)

[3.2 Effective population size 7](#_Toc106080433)

[**Supplementary Notes 4:** **Selective sweep analysis** 7](#_Toc106080434)

[4.1 Genome-Wide Selective Sweep between cashmere and ordinary goat 8](#_Toc106080435)

[4.2 Genome-Wide Selective Sweep for hair color 8](#_Toc106080436)

[Supplementary Figures 10](#_Toc106080437)

[Supplementary Tables 30](#_Toc106080438)

[Supplementary sequence 1 45](#_Toc106080439)

[Supplementary sequence 2 48](#_Toc106080440)

[**Supplementary Reference** 49](#_Toc106080441)

**Supplementary Figure List**

[Supplementary Fig. 1 PCA analysis. 10](#_Toc106045908)

[Supplementary Fig. 2 Results of genetic structure analysis of 13 goat breeds. 11](#_Toc106045909)

[Supplementary Fig. 3 Results of genetic structure analysis of 10 Chinese goat breeds. 12](#_Toc106045910)

[Supplementary Fig. 4 Phylogenetic tree of 10 Chinese native goat breeds. 13](#_Toc106045911)

[Supplementary Fig. 5 Inferred goat tree of mixture events deduced by TreeMix. 14](#_Toc106045912)

[Supplementary Fig. 6 Bar graph of enriched terms across positively selected genes with GO term enrichment analysis. 14](#_Toc106045913)

[Supplementary Fig. 7 Analysis of the signatures of positive selection in the genome of cashmere and ordinary goat breeds. 15](#_Toc106045914)

[Supplementary Fig. 8 Analysis of the signatures of positive selection in the genome of goat breeds with white and black coat color. 16](#_Toc106045915)

[Supplementary Fig. 9 Analysis of the signatures of positive selection in the genome of goat breeds with white and brown coat color. 17](#_Toc106045916)

[Supplementary Fig. 10 Ratios and ZFst values around the *ASIP* locus. 18](#_Toc106045917)

[Supplementary Fig. 11 CNVs at the *KIT* locus. 19](#_Toc106045918)

[Supplementary Fig. 12 CNVs at the ASIP locus. 20](#_Toc106045919)

[Supplementary Fig. 13 Visualization of whole genome sequencing data identifies 582 bp deletion in the ancient goat. 20](#_Toc106045920)

[Supplementary Fig. 14 Visualization of Whole Genome Sequencing Data identifies 582 bp deletion near *LHX2* in the ibex goats. 21](#_Toc106045921)

[Supplementary Fig. 15 The pattern of SNP genotypes near DEL among 13 goat breeds. 21](#_Toc106045922)

[Supplementary Fig. 16 Distribution diagram of cashmere yield data 22](#_Toc106045923)

[Supplementary Fig. 17 Distribution of cashmere diameter measurements 23](#_Toc106045924)

[Supplementary Fig. 18 The expression of *LHX2*, *JAK2* and *Notch1* in different days (from 45 d to 135 d) of fetal skin. 24](#_Toc106045925)

[Supplementary Fig. 19 Schematic drawing of the construction of DNA plasmids. 25](#_Toc106045926)

[Supplementary Fig. 20 The expression of *LHX2* and *AR* in different months of a year. 26](#_Toc106045927)

[Supplementary Fig. 21 The expression of *JAK2* and *EDA2R* as well as *FGF5* in different months of a year. 27](#_Toc106045928)

[Supplementary Fig. 22 The PCR identification of deletions in sheep. 28](#_Toc106045929)

[Supplementary Fig. 23 The expression of *STK2* and *NOTCH1* as well as *TCF7L1* in different months of a year. 29](#_Toc106045930)

**Supplementary Table List**

[Supplementary Table 1 Overview of sequencing information of 120 goats 30](#_Toc106081189)

[Supplementary Table 2 Sequencing information of 95 published goats used in this study 33](#_Toc106081190)

[Supplementary Table 3 Sequencing information of Wild goats used in this study 37](#_Toc106081191)

[Supplementary Table 4 Sequencing platforms for 236 goats of 13 goat breeds. 38](#_Toc106081192)

[Supplementary Table 5 The genotype frequencies of the homozygous 582bp deletion (-/-) at *LHX2* locus for 15 goat breeds, obtained from whole genome sequencing. 39](#_Toc106081193)

[Supplementary Table 6 The genotype frequencies of the homozygous 504bp deletion (-/-) at FGF5 locus for 15 goat breeds, obtained from whole genome sequencing. 40](#_Toc106081194)

[Supplementary Table 7 The frequencies of the 582 bp deletion near *LHX2* with PCR amplification. 41](#_Toc106081195)

[Supplementary Table 8 The frequencies of the 504 bp deletion near *FGF5* with PCR amplification. 42](#_Toc106081196)

[Supplementary Table 9 Published ancient samples used in this study. 43](#_Toc106081197)

[Supplementary Table 10 Samples for PSMC analysis 44](#_Toc106081198)

[Supplementary Table 11 Primers used for amplification of deletion sequence 44](#_Toc106081199)

**Supplementary Notes 1**

**1.1 Sample information and sequencing**

All 120 goats of 8 breeds were used in this study, which includes 30 Chaidamu Cashmere Goats (CDMC) from Chaidamu City of Qinghai Province; 11 Ujumqin Cashmere goats (UC) from Ujimqin Banner of Inner Mongolia Province; 8 Guizhou Black goats (GZB) from Guiyang City of Guizhou Province; 11 Yunnan Black Bone Goats (YNBB) from Lanping County of Yunnan Province; 10 Matou Goats (MT) from Shangqiu City of Henan Province; 10 Jining Grey Goats(JNG) from Jining City of Shandong Province; 10 Jintang black goat(JTB) from Jintang County of Sichuan Province; 12 Chengdu brown goat(CDB) from Chengdu City of Sichuan Province.

Firstly, ear tissues or blood of goats were collected on-site and stored in alcohol sampling tubes or blood collection tubes; then genomic DNA was extracted by commercial kits, and samples with DNA integrity and concentration > 50ng/ul were used for library construction. Next, the standard circularization step of DNBSEQ was carried out to make DNA nanoballs. Finally, the libraries were sequenced using BGIseq500 with PE100^1^.

## 1.2 Quality control and reads mapping

Quality control of all raw reads was performed using SOAPnuke1.5.0 (https://github.com/BGI-flexlab/SOAPnuke), with the following criteria: quality value ≤20; low-quality bases >30%; and N bases >5%. Clean reads were then mapped to the reference genome ARS1 (https://www.ncbi.nlm.nih.gov/genome/10731?genome_assembly_id=281266) using BWA-0.7.12^2^ (https://sourceforge.net/projects/bio-bwa/) with default parameters. In the mapping process, a BAM file index was built using SamTools (https://github.com/samtools/samtools/releases/), followed by the bam file being sorted and removed for duplicated reads with picard-tools-1.105 (https://github.com/broadinstitute/picard/releases).

**1.3 Variant calling and annotation**

After mapping, the “HaplotypeCaller”, “CombineGVCFs” and “GenotypeGVCFs” in GATK4 were used to detect SNPs and indels with default parameters. The output VCF File was then screened for SNPs using "SelectVariants function" of GATK4. We further reserved the SNPs using "VariantFiltration" of GATK4 using the following criteria: QD < 2.0; FS > 60.0; MQ < 40.0; and ReadPosRankSum < -8.0. Next, we used VCFtools to obtain high-quality SNPs combined with population information. SNPs that met at least one of the following standards were excluded: (i) minor allele frequency (MAF) <0.05; (ii) SNP call rate <90%; and (iii) P < 0.000001 for the Hardy–Weinberg equilibrium. Only the loci of two alleles were retained for the subsequent analyses. Finally, ∼13 million SNPs were remained for further analysis.

**Supplementary Notes 2**: **Population structure and phylogenetic analysis**

### 2.1 Geographic distribution mapping

First, we obtained the longitude and dimension data of 10 goat sampling sites through Google map; then used package "mapdata" and "ggrepel in R for mapping; finally, the Photoshop software was used for organization and optimization.

### 2.2 Principal component analysis (PCA)

The PLINK was used for principle component analysis (PCA) with 120 goats based on the filtered SNPs using function "--vcf vcf --out pca –pca --chr-set 29 --allow-extra-chr". The contribution of PCA1 and PCA2 was 15.94% and 10.3% respectively. The PCA further reveals that the difference between North China (cashmere goats, JNG and MT) and South China (JTB, CDB, YNBB and GZB) is greater than that between cashmere goats and ordinary goats (**Supplementary Fig. 1**).

### 2.3 Population admixture

Population structure was inferred using a Bayesian-based approach via the software package Admixture_linux-1.23 (https://dalexander.github.io/admixture/index.html); then, the cross validation was performed to choose the optimal *k* value. The analysis process includes the following three steps:

1. Converting VCF format to PLINK format. The Linux command line was “vcftools --vcf vcf --plink --out goat.plink”;

(2) Using PLINK for further filtering with Linux command line “plink --noweb --file plink --geno 0.05 --maf 0.05 --hwe 0.0001 --chr-set 29 --make-bed --out QC”; after this step we get the corresponding bed file.

(3) Using ADMIXTURE v1.23 for population structure analysis with k values ranged from 3 to 7. Five-fold cross-validation was used to evaluate the fitness. For each k, we ran the ADMIXTURE for 20 times and calculated the corresponding cross-validation errors with the Linux command line “admixture --cv QC.bed $k | tee log${k}.out”; then the results were plotted using R.

### 2.4 Phylogenetic tree

The VCF files were converted into a matrix using VCF2Dis-1.09 (https://github.com/BGI-shenzhen/VCF2Dis), and tree files were formed using PHYLIPNEW-3.69.650 (https://evolution.genetics.washington.edu/phylip.html). Then a phylogenetic tree based on genetic distance was constructed via the neighbor-joining method by iTOL^3^ (http://itol.embl.de). The Linux command line of converting to a matrix is "VCF2Dis -InPut vcf -OutPut p_dis.mat"; the Linux command line of constructing a phylogenetic tree is "PHYLIPNEW-3.69.650/bin/fneighbor -datafile p_dis.mat -outfile goat.tree.txt -matrixtype s -treetype n -outtreefile goat.n.tree.tre”. The neighbor-joining (NJ) tree revealed strong clustering of the Chinese native goat into three genetic groups that presented a low level of genetic differentiation, which recapitulated the finding in the PCA (**Supplementary Fig. 4**).

### 2.5 LD decay

PopLDdecay (https://github.com/BGI-shenzhen/PopLDdecay) was used to calculate the r^2^ of linkage disequilibrium (LD) with command "pLDdecay -InVCF vcf -MaxDist 300 -OutStat LDdecay".

**Supplementary Notes 3:** **Gene flows and effective population size**

### 3.1 Gene flow

A population-level admixture analysis was carried out with TreeMix (version 1.12) program (Pickrell and Pritchard 2012). It uses genome-wide SNP sites to infer the Maximum Likelihood (ML) tree for the 12 goat breeds (236 individuals) and an outgroup (Bezoars), with the command '-i input -bootstrap -k 500 -root IRW -o output'. The covariance matrix of the ML tree was estimated to reflect the correspondence between the ML tree and the SNP data and to identify pairs of populations that showed poor fits in the ML tree. When the ML tree did not fully describe the data, an admixture analysis was performed by allowing migration events. The poor-fit populations were regarded as candidates for adding potential migration edges. Migration events are gradually added to the ML trees until the model can explain 98% of the variation in breeds. The command was '-i input -bootstrap -k 500 -m migration events -o output'. The maximum-likelihood (ML) tree without migration events inferred from the TreeMix analysis divided the 236 goats into three clusters, which is similar to the population structuring patterns identified from the phylogenetic tree, PCA and genetic structure analysis (**Supplementary Fig. 5**). Migration events were detected among the three clusters when potential migration edges were added to the ML tree (**Supplementary Fig. 5**).

### 3.2 Effective population size

PSMC software was used to infer historical dynamics of effective population size and the divergence timing of goats based on seven samples from four lineages with a high sequencing depth^4^. The whole-genome diploid consensus sequences for each sample were generated by SAMtools and BCFtools with the parameter C50. Sites with sequencing depths < 10 and > 100 (vcfutils.pl vcf2fq -d 10 -D 100) were removed to reduce the probability of false positives. The PSMC parameter (psmc -N25 -t15 -r5 -p "4+25*2+4+6") was used to infer the historical effective population size, where the parameters of generation time and mutation rate were set to 2.0 and 2.5e^-8^, respectively.

**Supplementary Notes 4:** **Selective sweep analysis**

Fst and θπ were performed to evaluate fixation and differentiation. High-quality SNPs were chosen to calculate Fst using VCFtools based on sliding 150kb windows with a 10kb step length. Fst values were then standardized as ZFst by calculating Z-scores according to the formula x*=(−x)/σ⁠. We also calculated the θ_π_ ratios and log_2_-transformed, namely Log_2_(θ_π_ Ratios). We annotated genes functions for those windows with larger ZFst and Log_2_(θ_π_ Ratios) values (top 1%) ^5,6^. Gene ontology and KEGG analyses were performed using Metascape^7^ and compared to the human genome as background.

### 4.1 Genome-Wide Selective Sweep between cashmere and ordinary goat

We performed selective sweep analysis with genome sequences of 84 cashmere goats (IMC, CDMC, LNC) and 58 ordinary goats (YNBB, GZB, JTB and GZB) We choose those windows with top 1% of statistic (ZFst>4.042 and log_2_(θ_π_ Ratios)> 1.348) for candidate windows. Finally, we identified 141 candidate genes in and around these windows (500kb from both sides) (Supplementary Table s6 and Supplementary Fig. 6).

### 4.2 Genome-Wide Selective Sweep for hair color

First, we scanned for selection signatures for the white coat group (CDMC, UC, IMC, LNC) and the black coat group (YNBB, GZB). We identified signature regions harboring *KIT*, *KITG* and *IRF4*(Supplementary Fig.7 and Supplementary Table s7) and 100 kb copy number variants (CNVs) downstream the *KIT* (chr6: 70,859,258-70,959,918) (Supplementary Fig. 10) , which is consistent with the previously report ^8^. We next searched for the selection signature in the white coat group (LNC, IMC, CDMC and UC) and the brown coat group (CDM),We also identified a striking signature harboring *ASIP*, *AHCY* and *ITCH* (Supplementary Fig. 8-9 and Supplementary Table s8) and ~154 kb CNV partly overlapping with *ASIP* (chr13:63,334,008-63,380,258) (Supplementary Fig. 11), which is consistent with the previously reported^8^.

**Supplementary Notes 5: Deletion of breakpoints of LHX2 and FGF5**

To detect the heterozygous genotype of goats more accurately, we designed PCR primers to detect the deletions in *LHX2* and *FGF5* (Supplementary Table 8 and Supplementary Table 9). Also, we used the same primers to detect the deletions in several sheep breeds (Hu sheep, Ujumqin sheep, and Australian white sheep) (Supplementary Fig. 21).

## Supplementary Figures


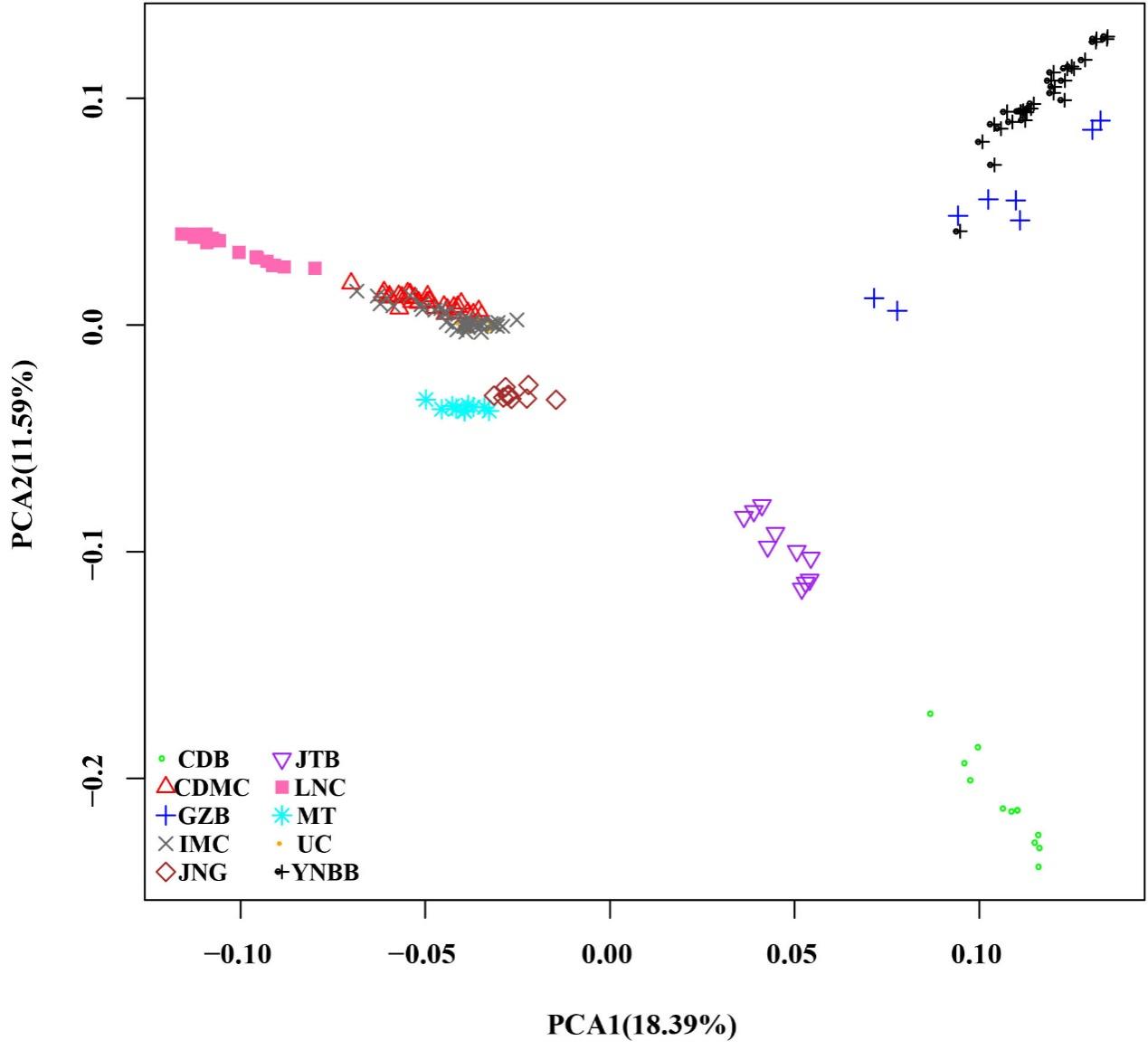


Supplementary Fig. 1 PCA analysis.

Principal components 1 and 2 of 10 local native goat breeds. The horizontal axis represents principal component 1 (PCA1), and the vertical axis represents principal component 2 (PCA2). Four cashmere goat breeds (CDMC, IMC, UC, LNC) were gathered; JNG and MTG breeds are close to the cashmere goats; The JTB, CDB, YNBB and GZB in Southwest China were all far from the cashmere goats and were divided into two small groups.


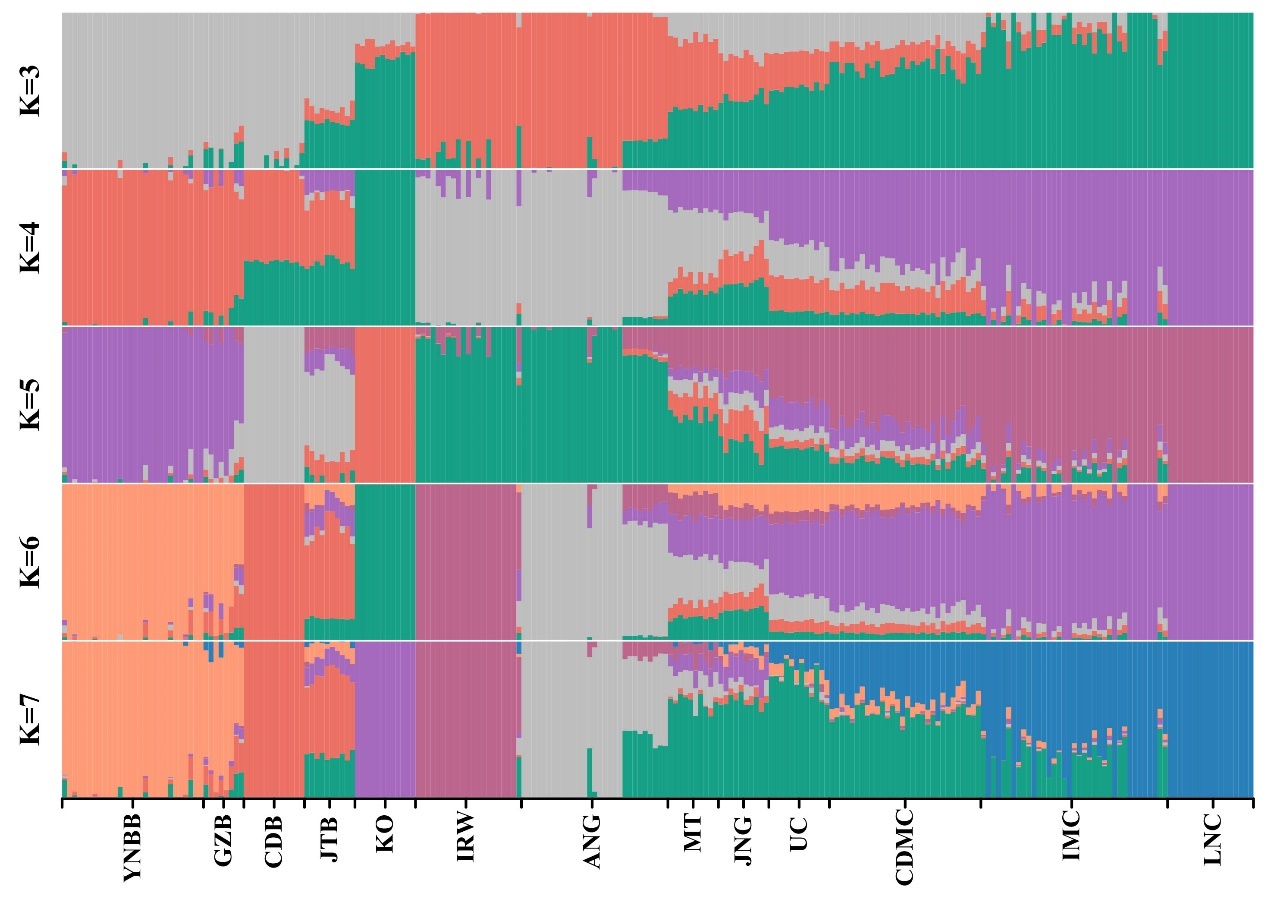


Supplementary Fig. 2 Results of genetic structure analysis of 13 goat breeds.

The length of each colored segment represents the proportion of the individual's genome inferred from K=3-7 ancestral populations. For the abbreviations of the individuals see supplementary table 3-5.


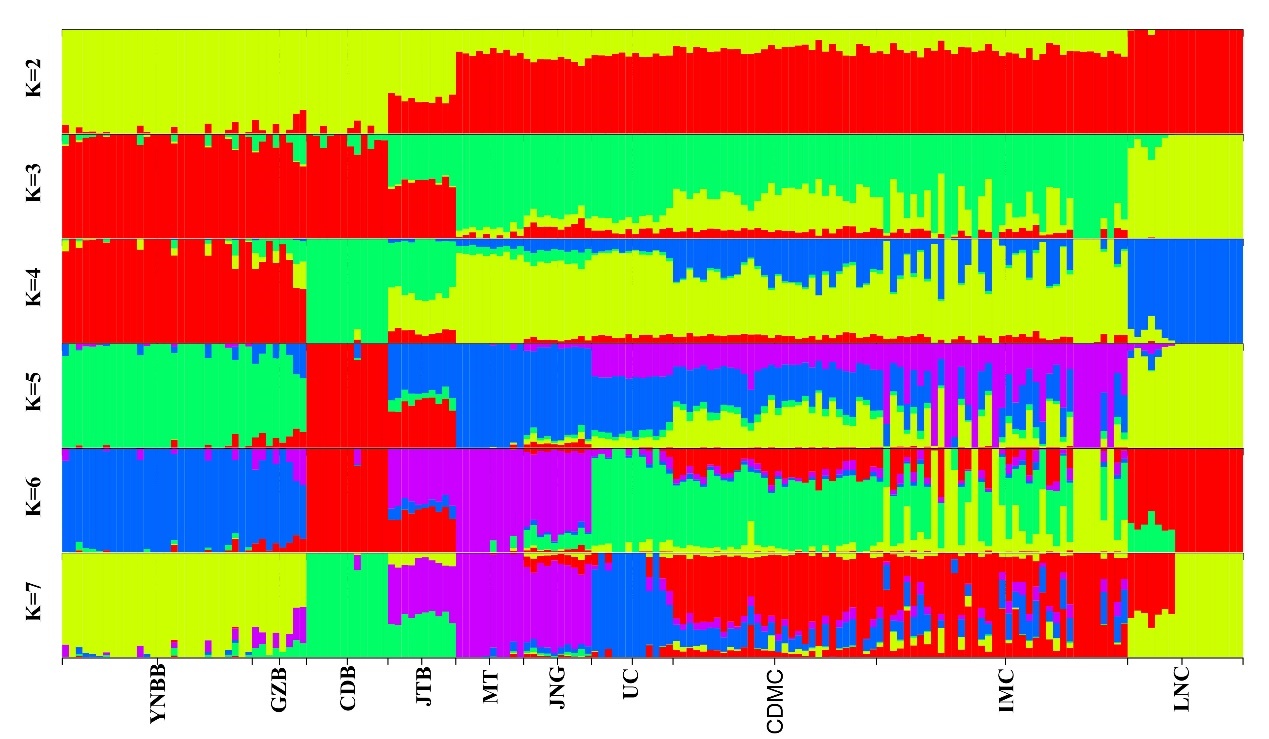


Supplementary Fig. 3 Results of genetic structure analysis of 10 Chinese goat breeds.

The length of each colored segment represents the proportion of the individual's genome inferred from K=2-7 ancestral populations. For the abbreviations of the individuals see supplementary table 3-4.


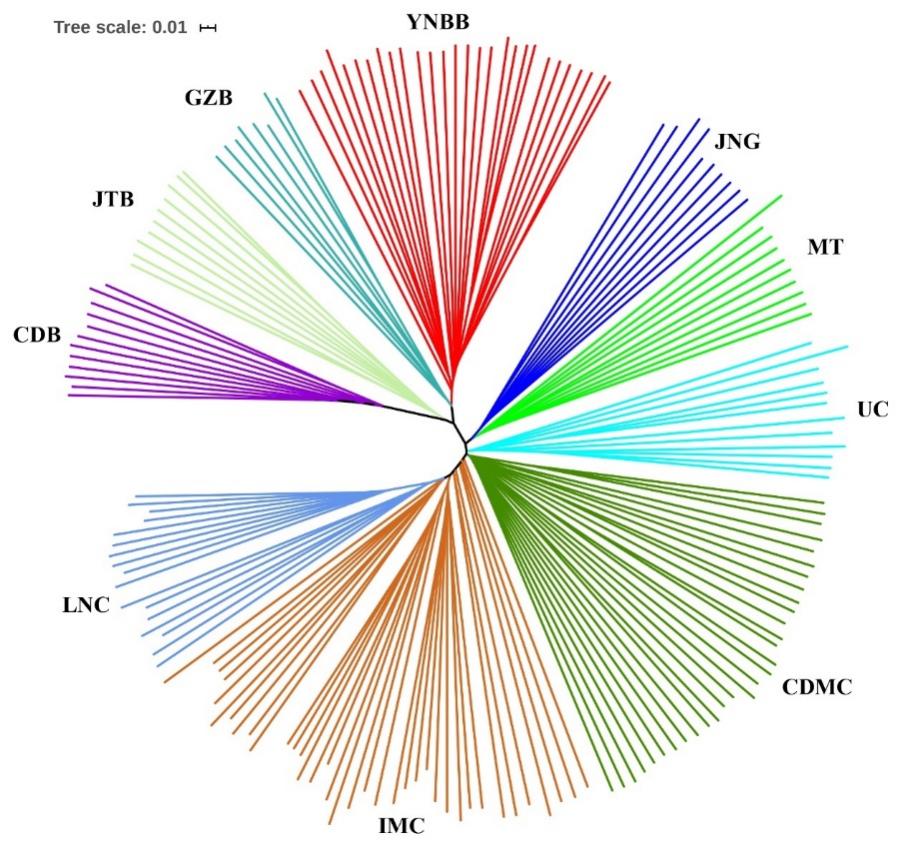


Supplementary Fig. 4 Phylogenetic tree of 10 Chinese native goat breeds.

Each color represents a breed, and each line represents an individual. Ten local goat breeds were clustered into two groups: four goat breeds in southwest China (CDB, JTB, GZB and YNBB) clustered for one group; LNC, IMC, CDMC, UC, MT and JNG gathered for another, JNG and MTG have clustered separately close to the cashmere goat subgroup.


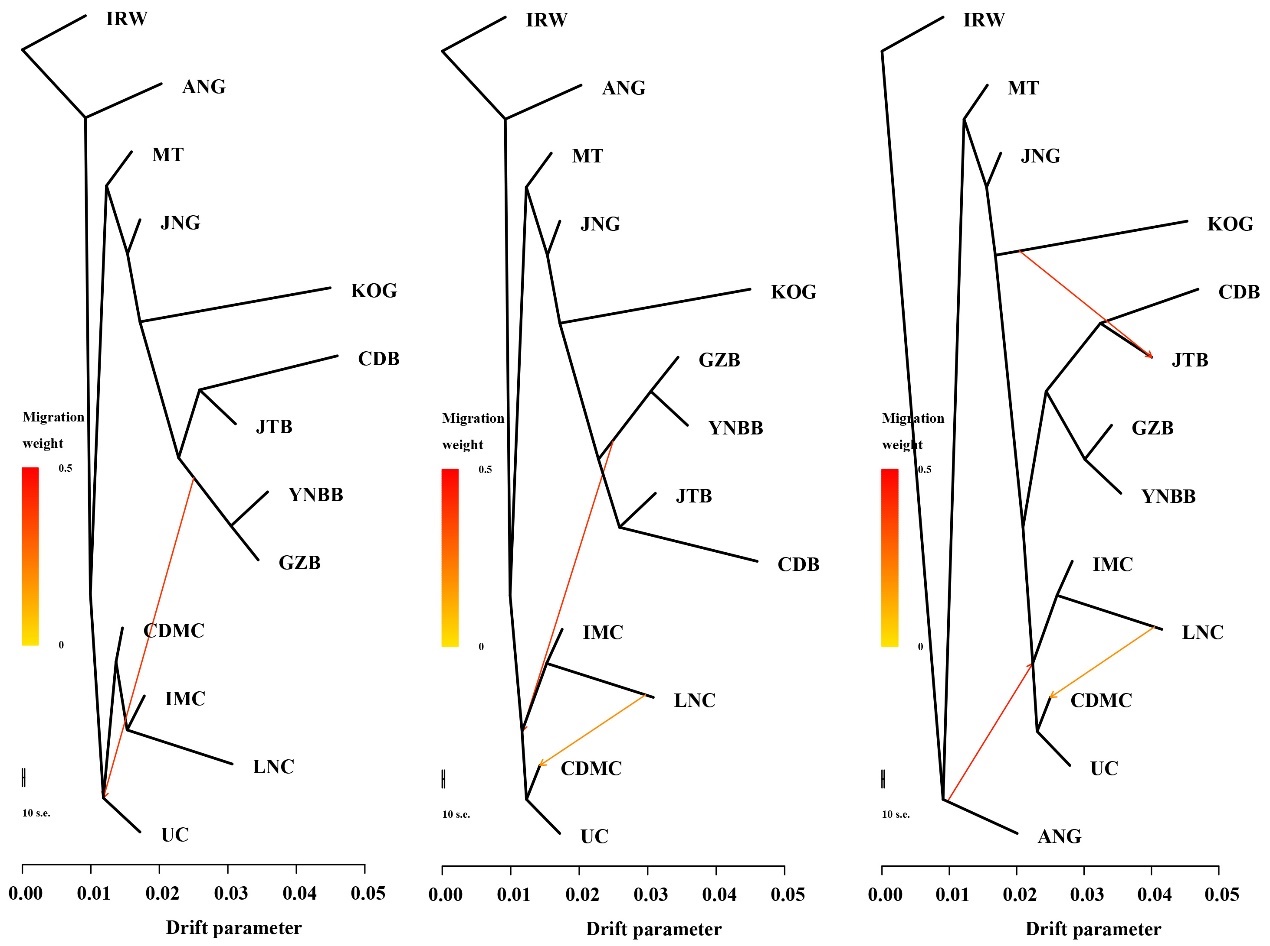


Supplementary Fig. 5 Inferred goat tree of mixture events deduced by TreeMix.

IRW samples were assigned as "outgroup" to root the tree. The graph inferred for the goat populations, allowing one, two and three migration events. Migration arrows are colored according to their weight. Horizontal branch lengths are proportional to the amount of genetic drift that has occurred on each branch. The scale bar shows ten times the average standard error of the entries in the sample covariance matrix.


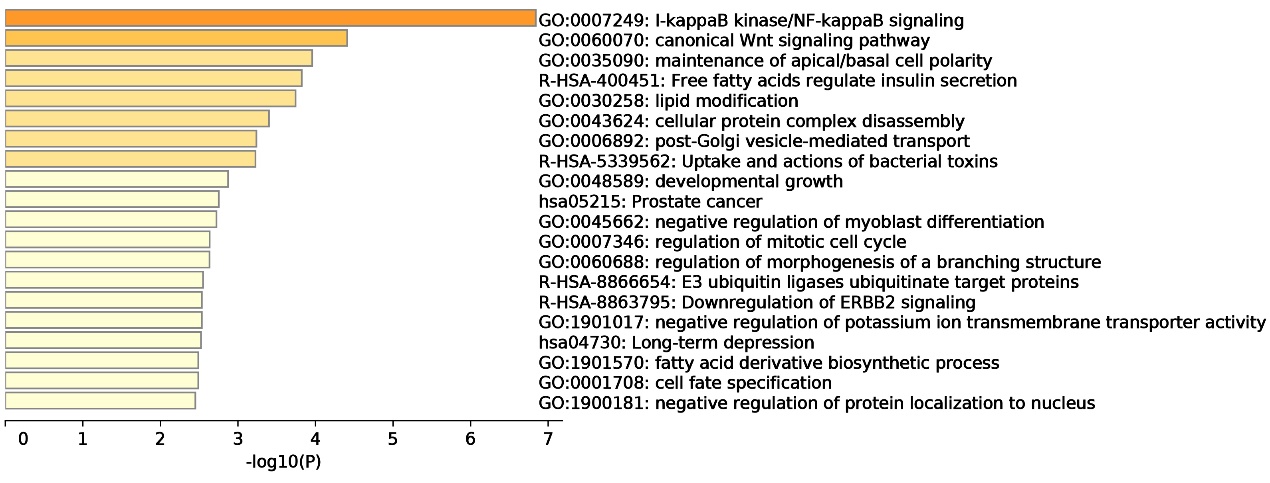


Supplementary Fig. 6 Bar graph of enriched terms across positively selected genes with GO term enrichment analysis.

X-axis represents the p-values.


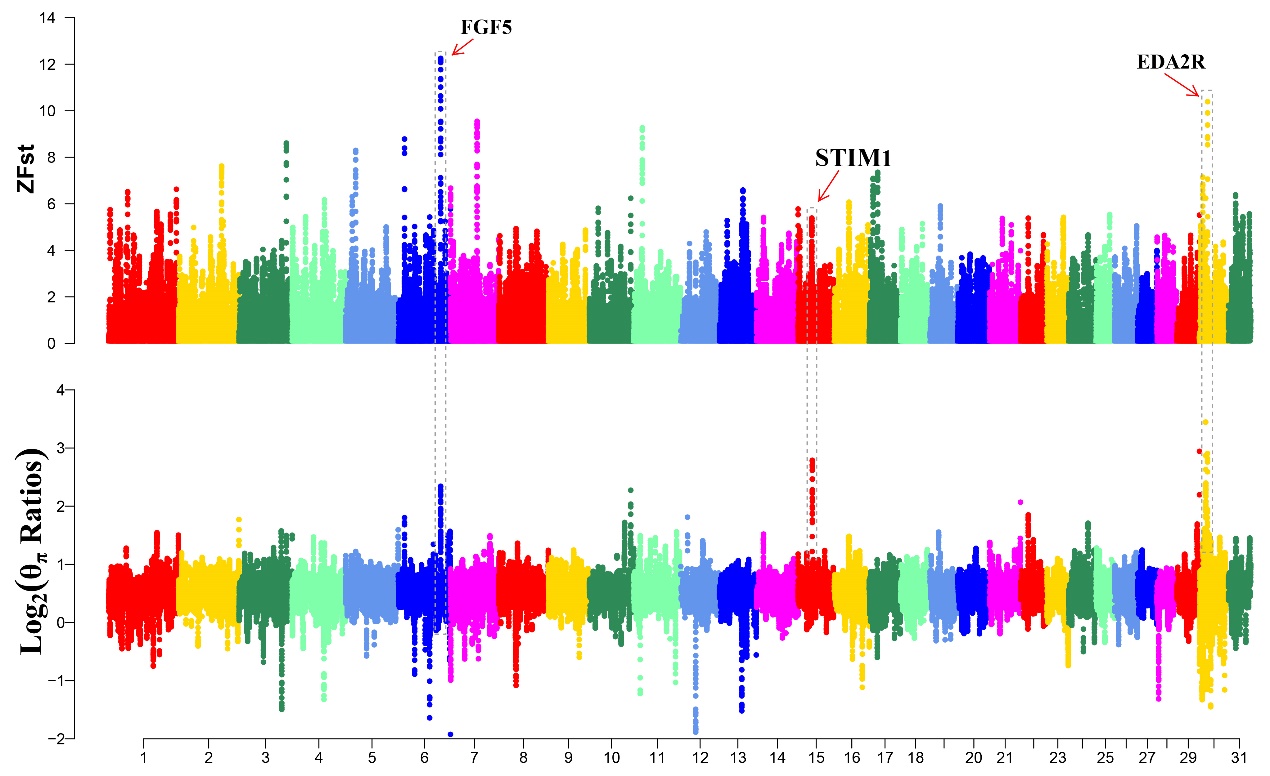


Supplementary Fig. 7 Analysis of the signatures of positive selection in the genome of cashmere and ordinary goat breeds.

Manhattan plot of the genome-wide distribution of pairwise ZFst and log_2_(θ_π_ Ratios) between cashmere goats (CDMC, IMC, LNC) and ordinary goats (YNBB, GZB, JTB, CDB) using a 150 kb window size and a 10 kb step size. The key candidate genes *FGF5, STIM1, KIT* related to cashmere were annotated at the significant signal. Red arrows and names indicate the top three candidate genes.


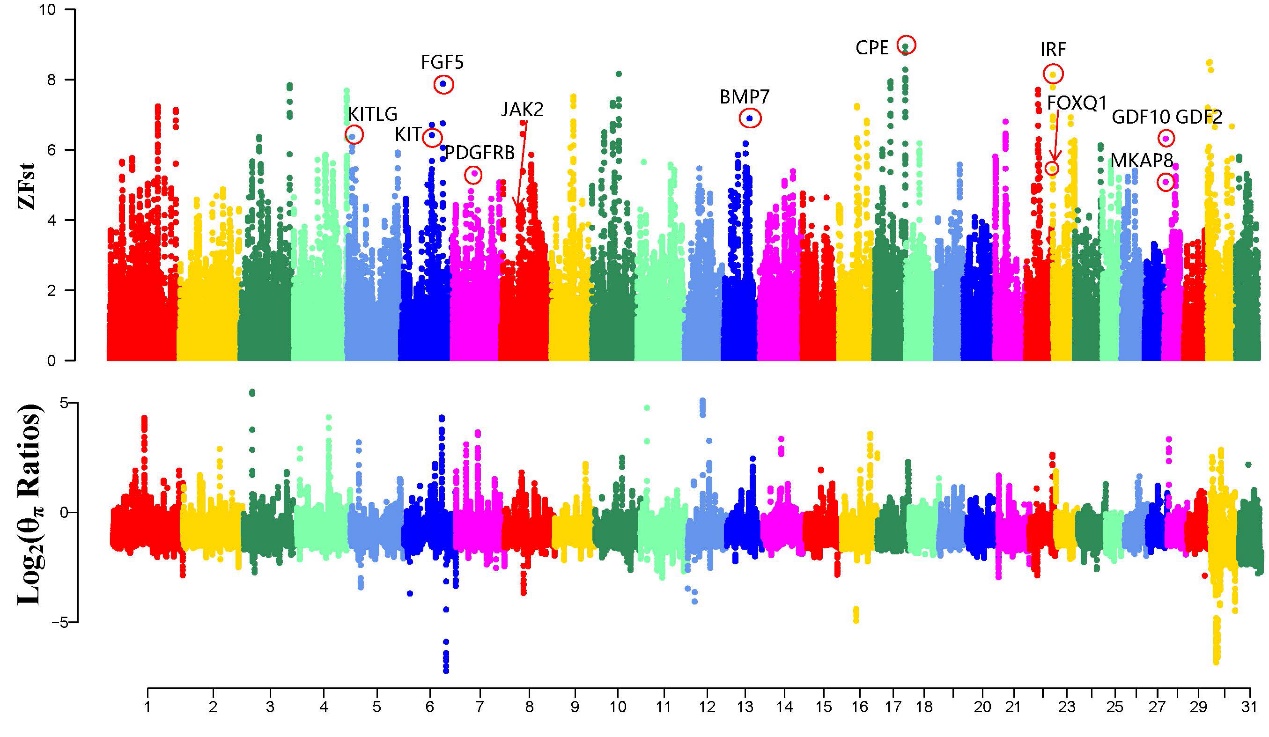


Supplementary Fig. 8 Analysis of the signatures of positive selection in the genome of goat breeds with white and black coat color.

Manhattan plot of the genome-wide distribution of pairwise ZFst and log_2_(θ_π_ Ratios) between white coat color goats (UC, CDMC, IMC, LNC) and black coat color goats (YNBB, GZB) using a 150 kb window size and a 10 kb step size. The key candidate genes *KIT*, *KITLG*, *FGF5*, *JAK2*, *FOXQ1* related to cashmere were annotated at the significant signal. Red circles and names indicate the top three candidate genes.


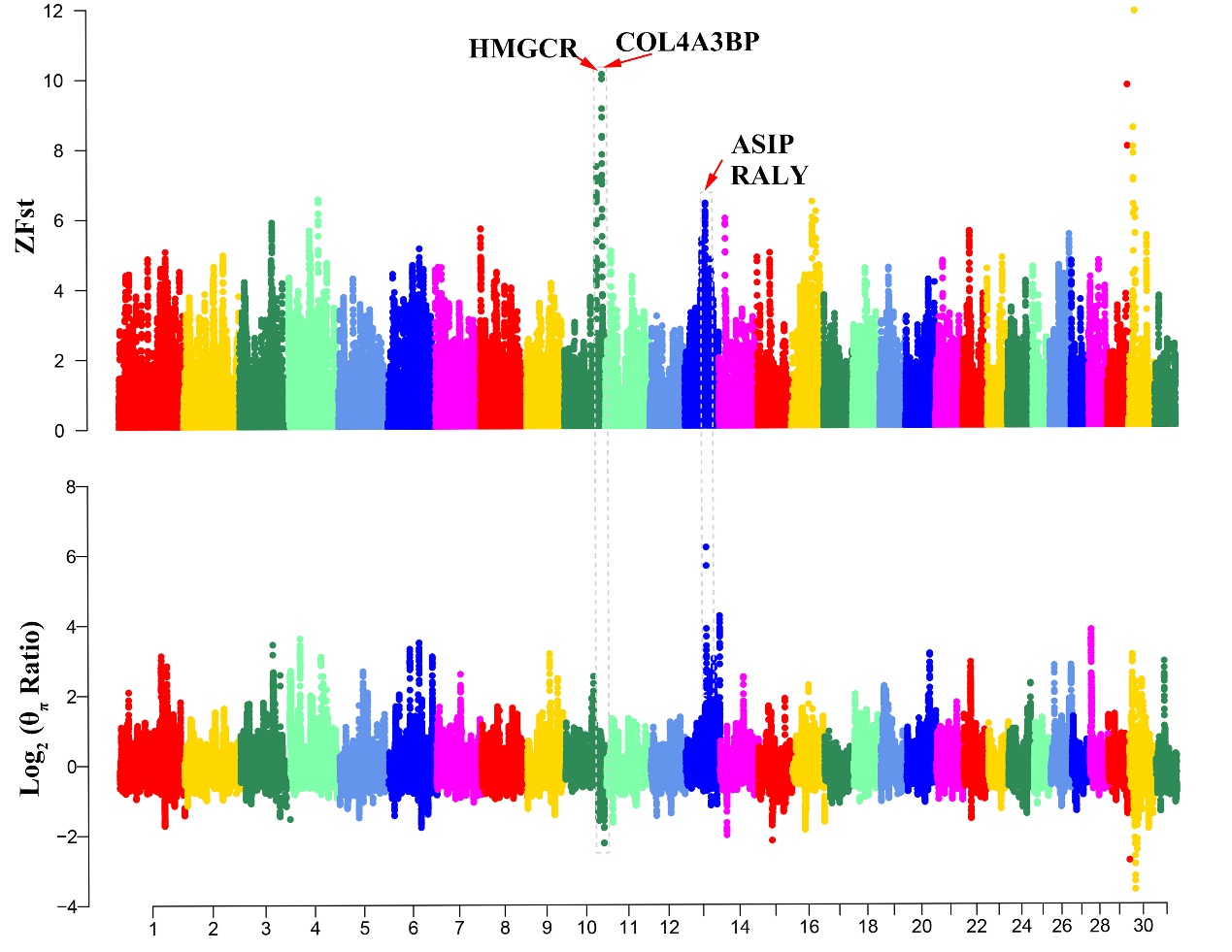


Supplementary Fig. 9 Analysis of the signatures of positive selection in the genome of goat breeds with white and brown coat color.

Manhattan plot of the genome-wide distribution of pairwise ZFst and log_2_(θ_π_ Ratios) between white coat color goats (UC, CDMC, IMC, LNC) and brown coat color goats (CDB) using a 150 kb window size and a 10 kb step size. The key candidate genes *ASIP*, *RALY*, *HMGCR*, *COL4A3BP* related to cashmere color were annotated at the significant signal. Red arrows and names indicate the top three candidate genes.


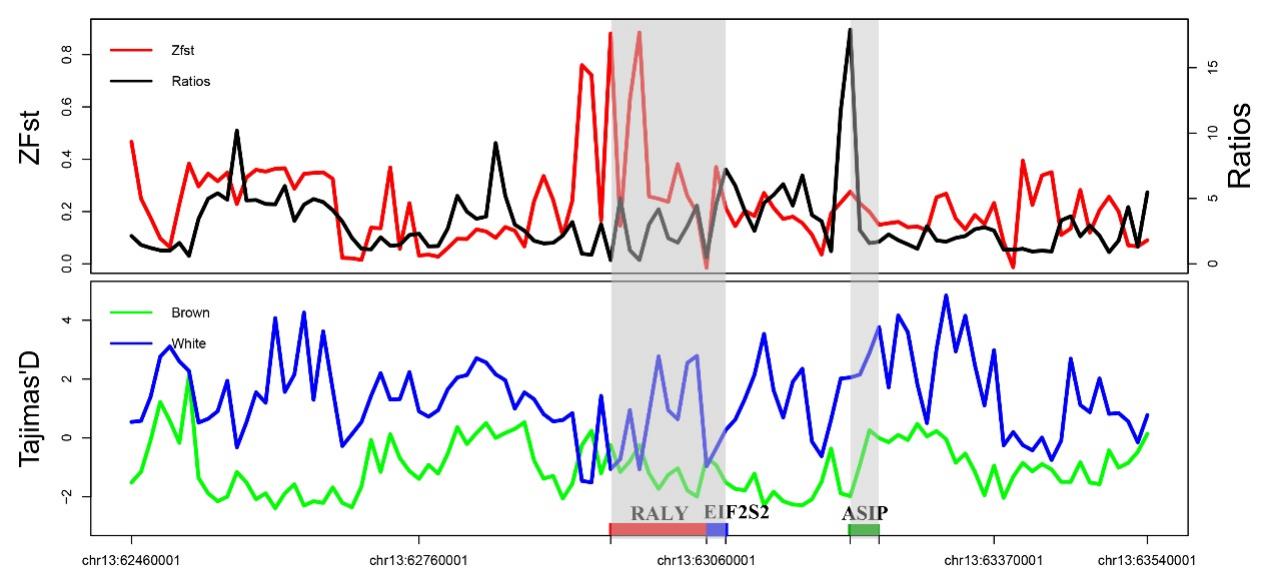


Supplementary Fig. 10 Ratios and ZFst values around the *ASIP* locus.

The black and red lines represent θπ Ratios and ZFst values, respectively. Tajima's D values around the *RALY*, *EIF2S2*, *ASIP* locus. The green and blue lines represent the brown coat color goats (CDB) and the white coat color goats (CDMC, IMC, UC, LNC).


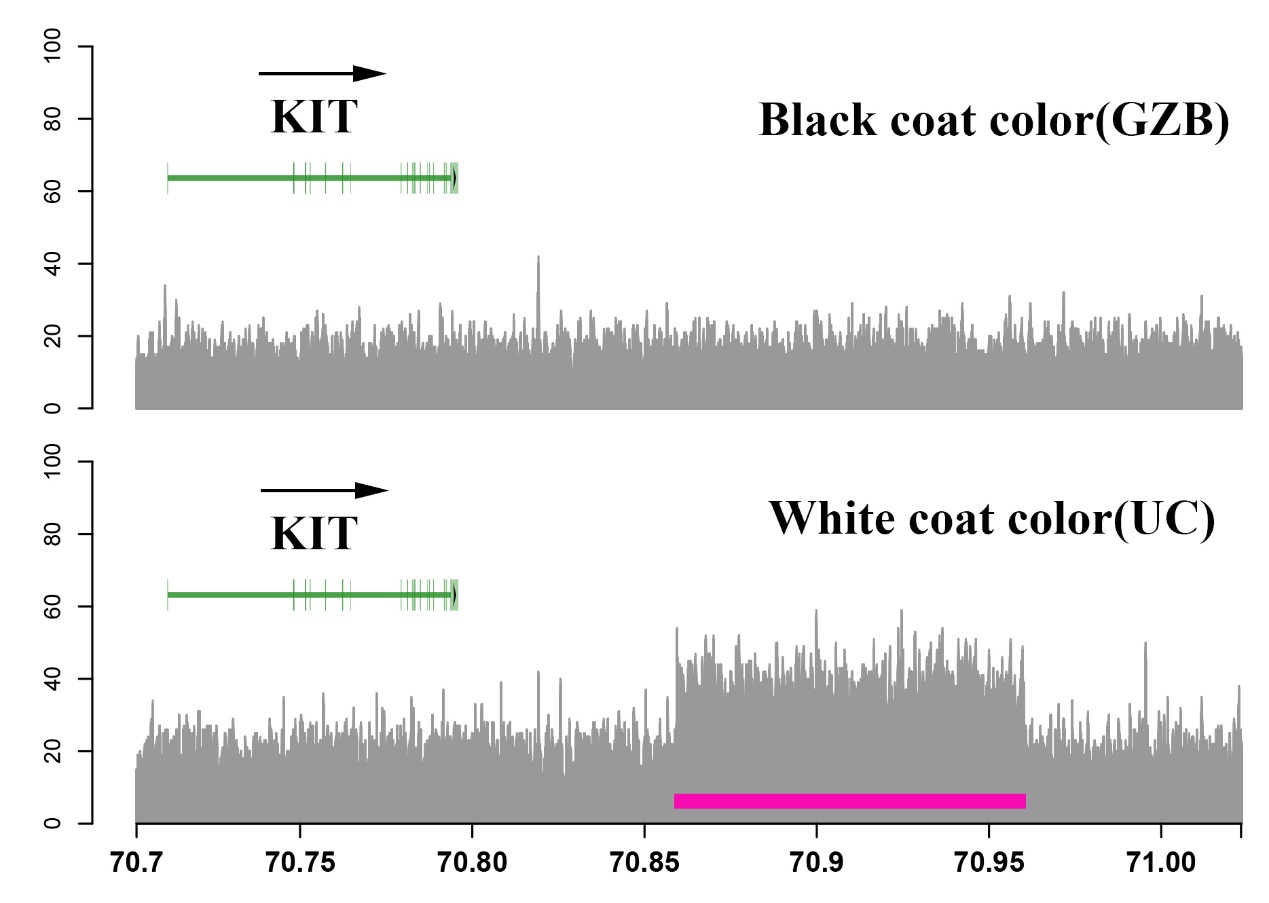


Supplementary Fig. 11 CNVs at the *KIT* locus.

The black coat color goat (GZB) does not show copy number variation (upper panel); however, in the white coat color goat (UC), the coverage plot shows a ~100 kb duplicated CNV (chr6:70859258-70959918) downstream of the *KIT* gene (lower panel).


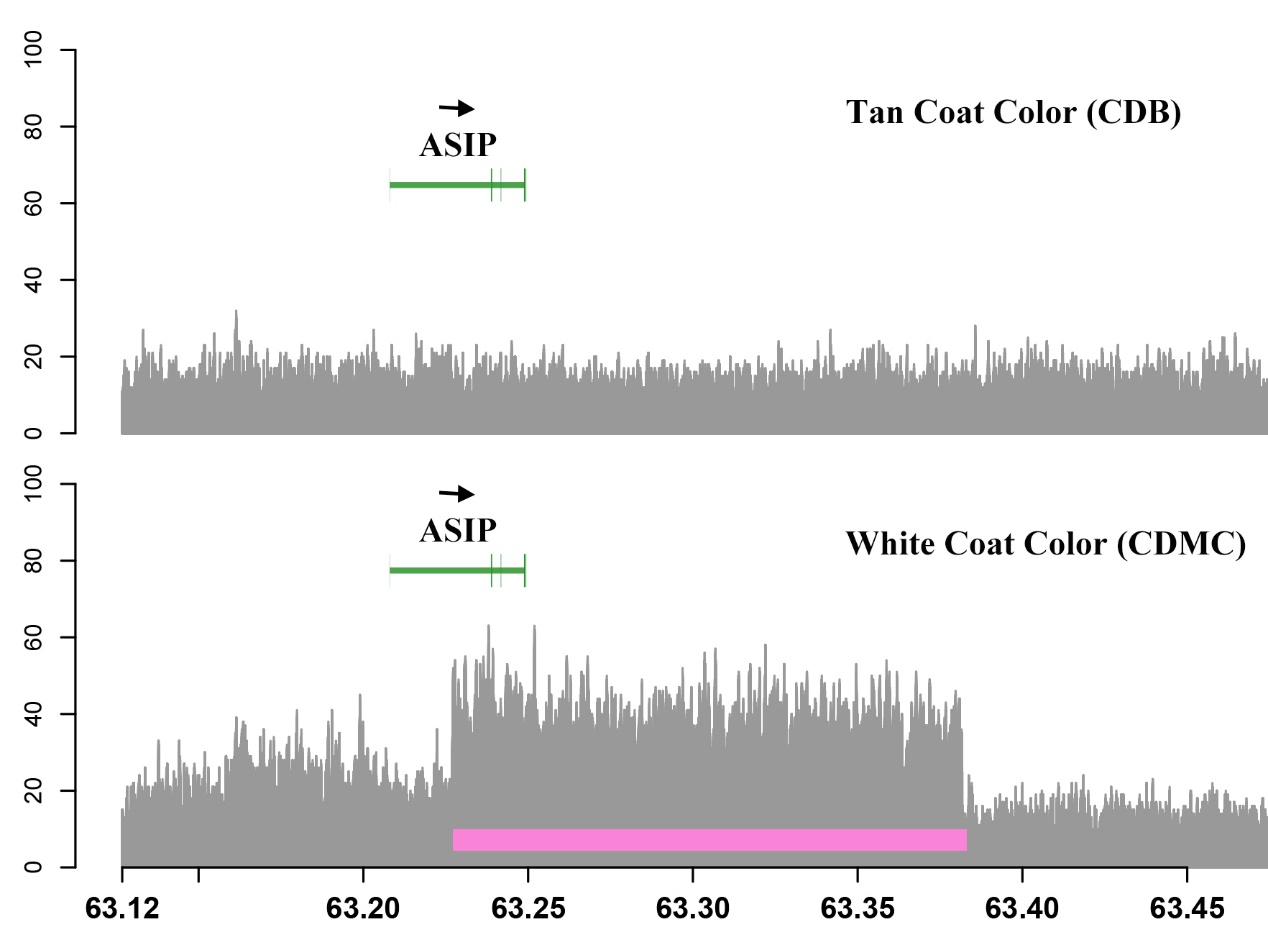


Supplementary Fig. 12 CNVs at the ASIP locus.

The black coat color goat (CDB) does not show any copy number variation. However, in the tan coat color goat (CDMC), the coverage plot shows a duplication of ~154 kb(chr13:63226824-63381501) downstream of the *KIT* gene.


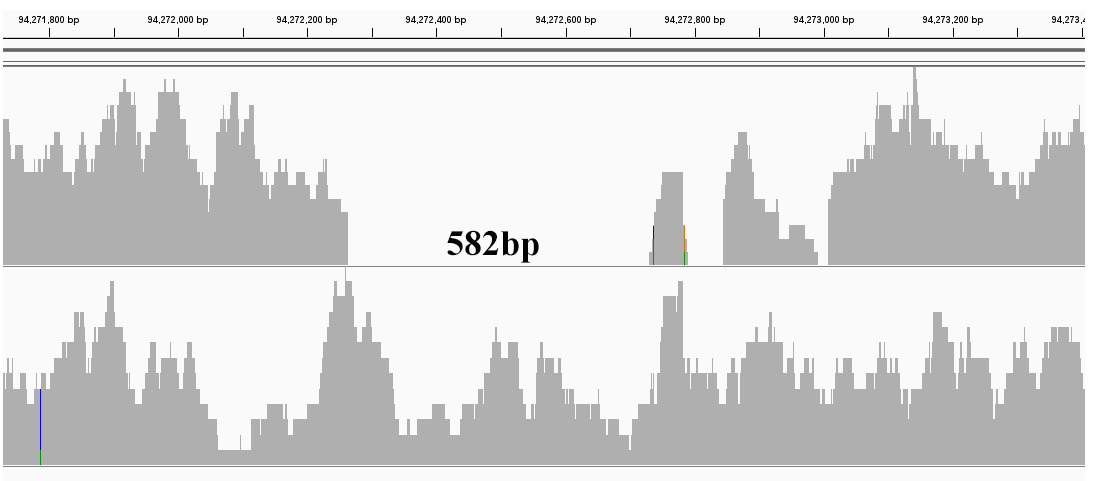


Supplementary Fig. 13 Visualization of whole genome sequencing data identifies 582 bp deletion in the ancient goat.

BAM files were visually inspected using the Integrative Genomics Viewer (IGV).


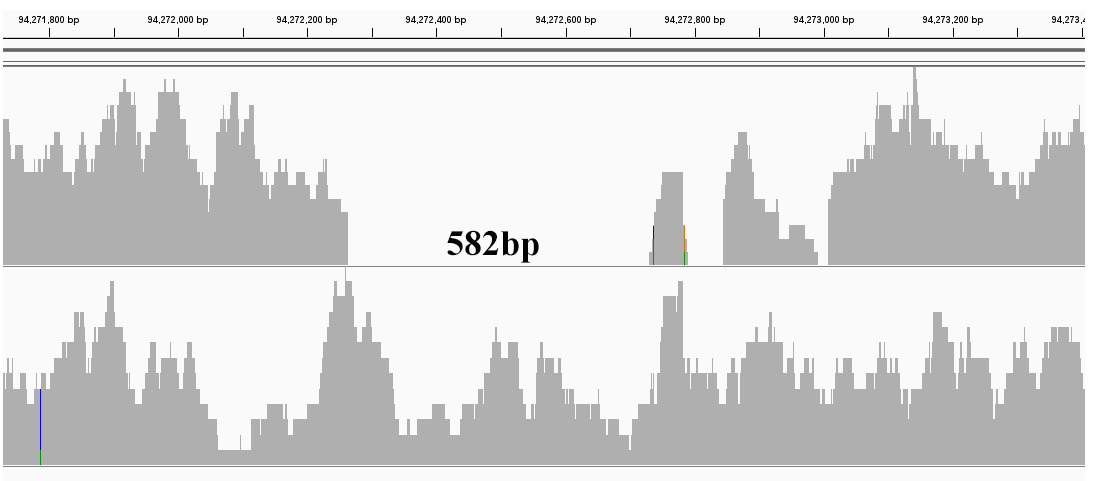


Supplementary Fig. 14 Visualization of Whole Genome Sequencing Data identifies 582 bp deletion near *LHX2* in the ibex goats.

Upper panel represents ibex goat carries 582bp deletion and lower panel not carries.


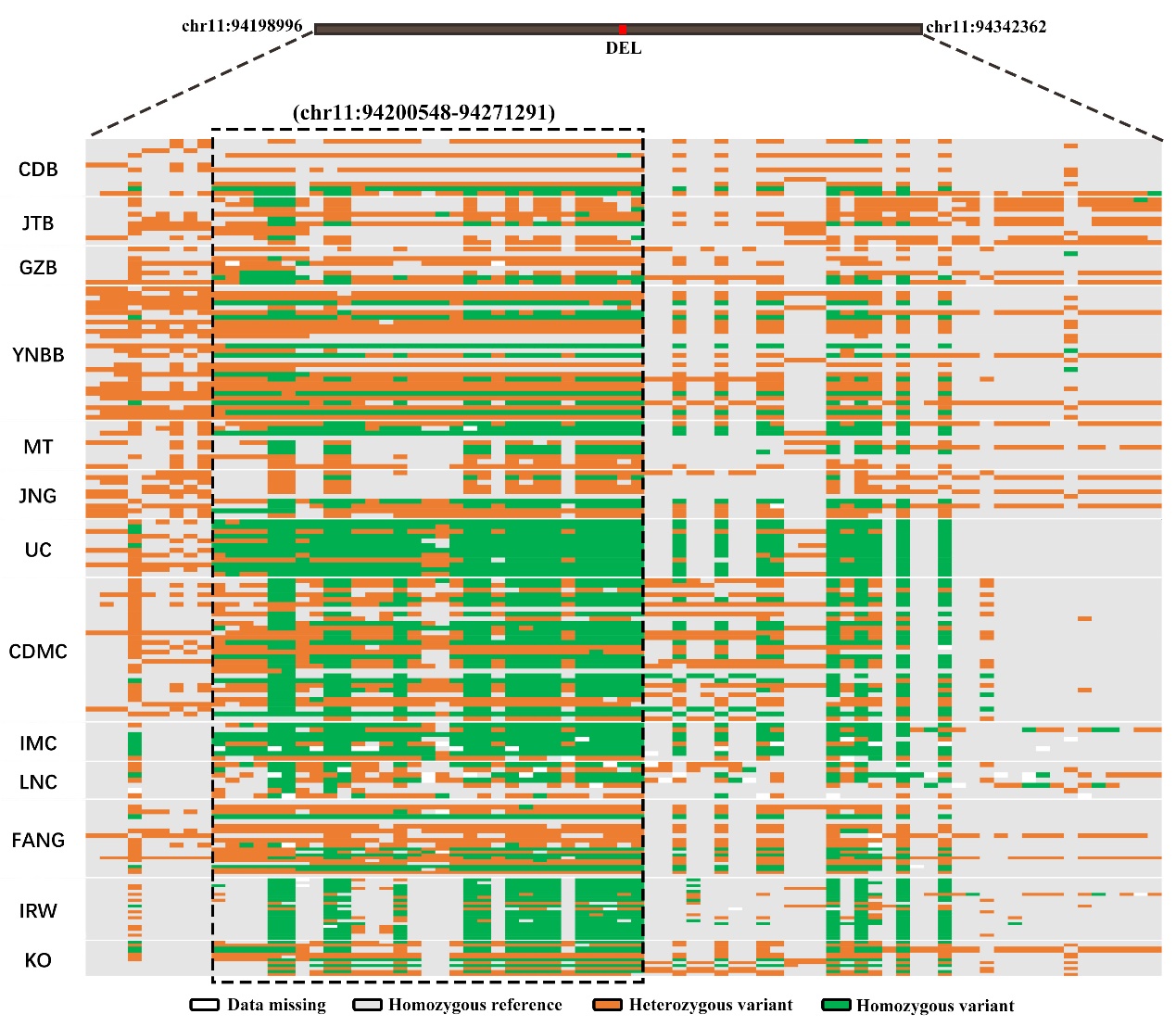


Supplementary Fig. 15 The pattern of SNP genotypes near DEL among 13 goat breeds.


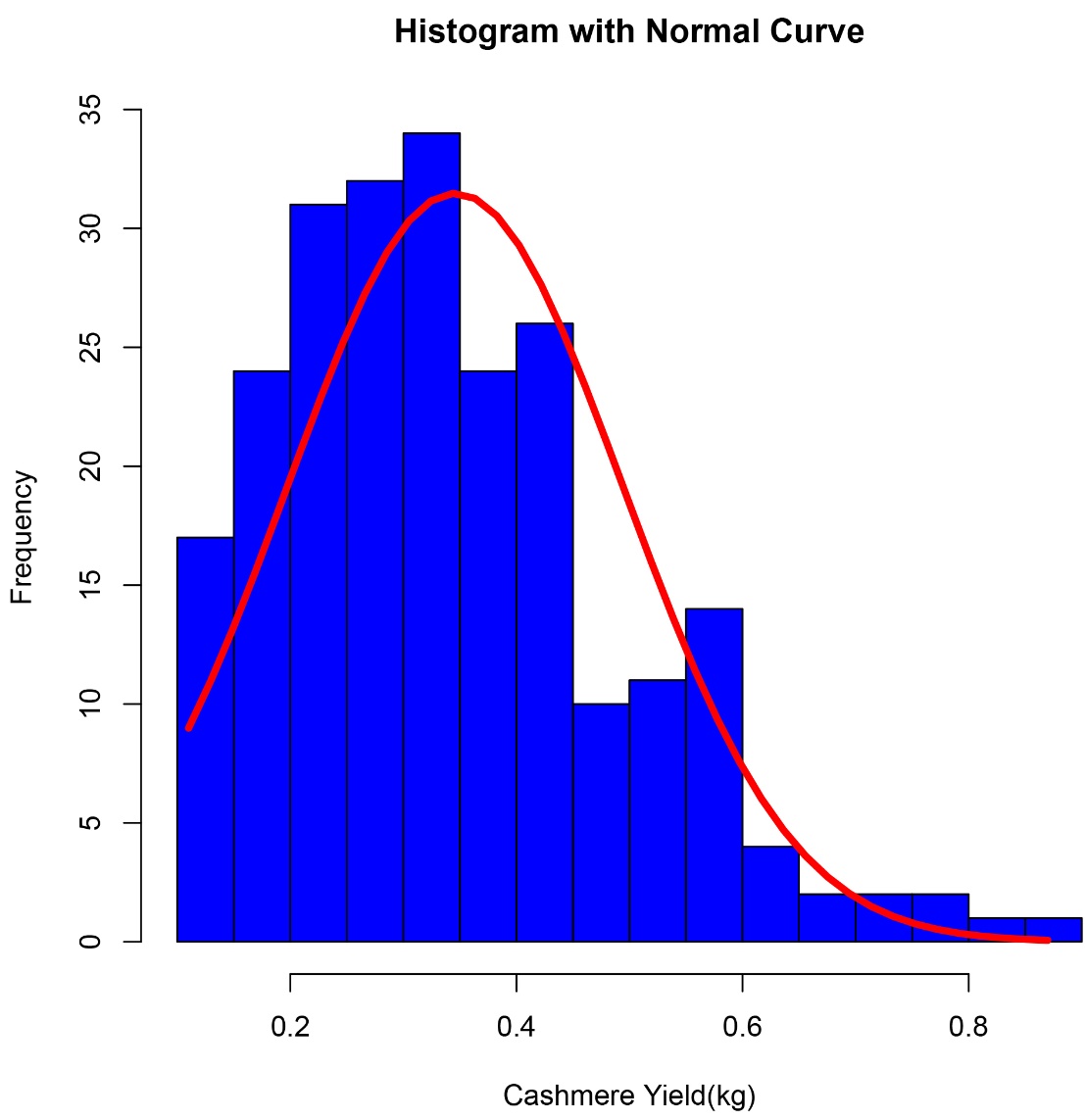


Supplementary Fig. 16 Distribution diagram of cashmere yield data


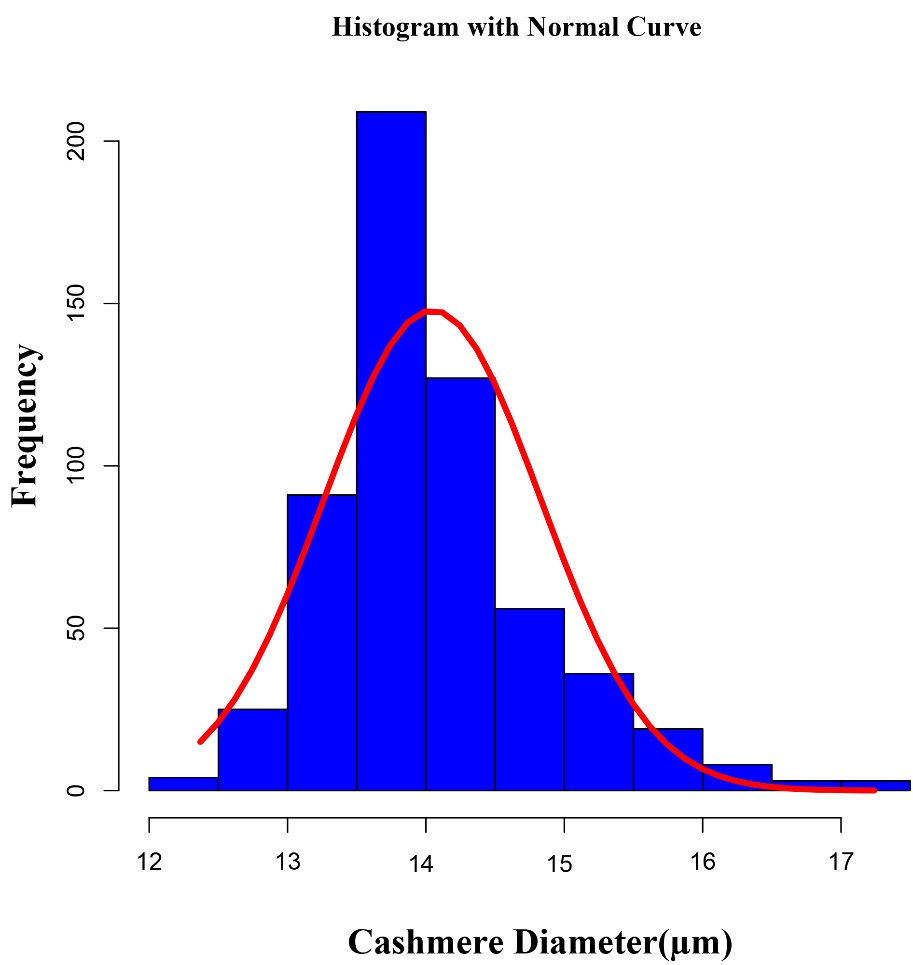


Supplementary Fig. 17 Distribution of cashmere diameter measurements


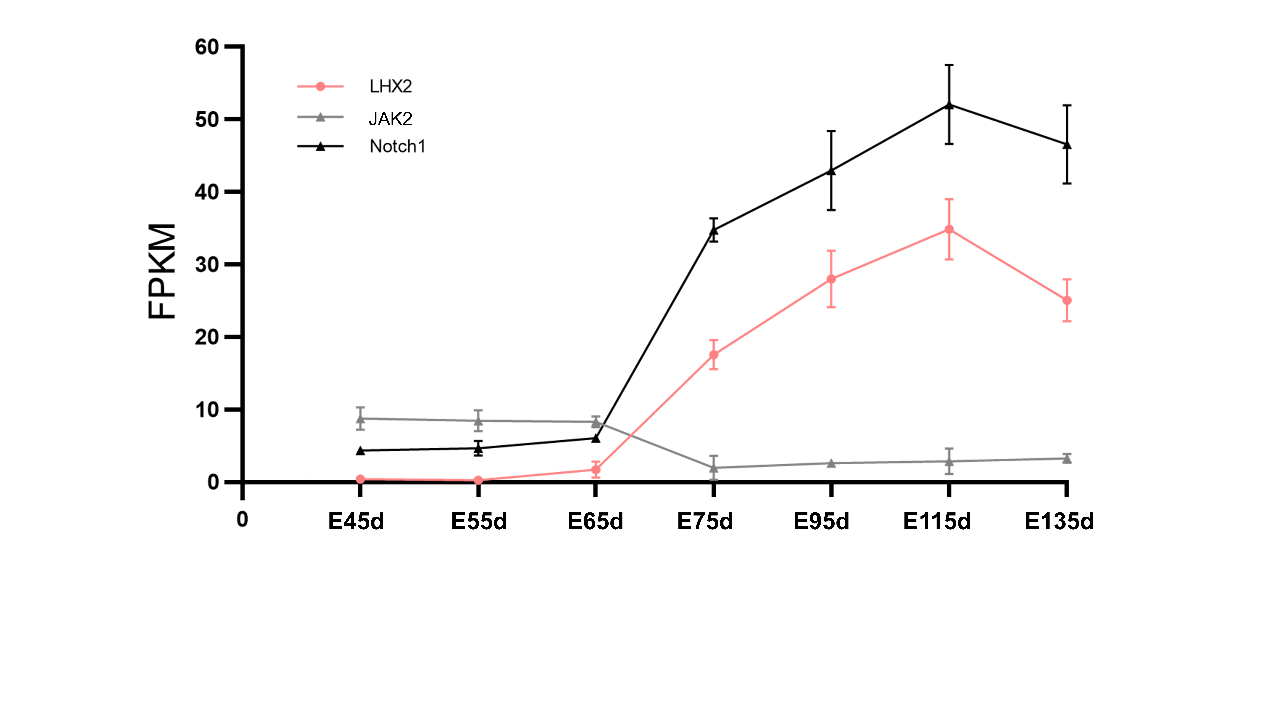


Supplementary Fig. 18 The expression of *LHX2*, *JAK2* and *Notch1* in different days (from 45 d to 135 d) of fetal skin.

E represents days of gestation. The expression data is downloaded from the published data^9^.


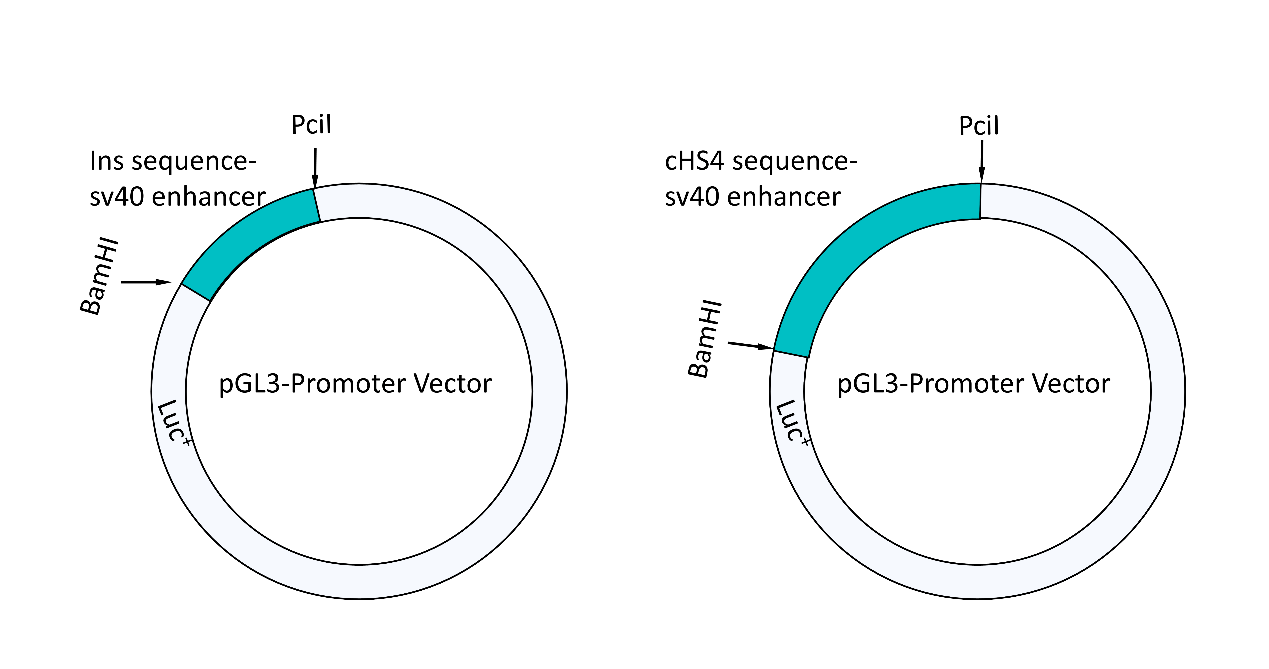


Supplementary Fig. 19 Schematic drawing of the construction of DNA plasmids.

The 796 bp DNA fragment (including 532 bp deletion sequence and some flanking sequence as well as SV40 enhancer, named Ins) and the 1444 bp DNA fragment （including 1206 bp cHS4 sequence and SV40 enhancer, named cSH4) were synthesized and subsequently inserted downstream and upstream of the SV40 promoter in pGL3 plasmid.


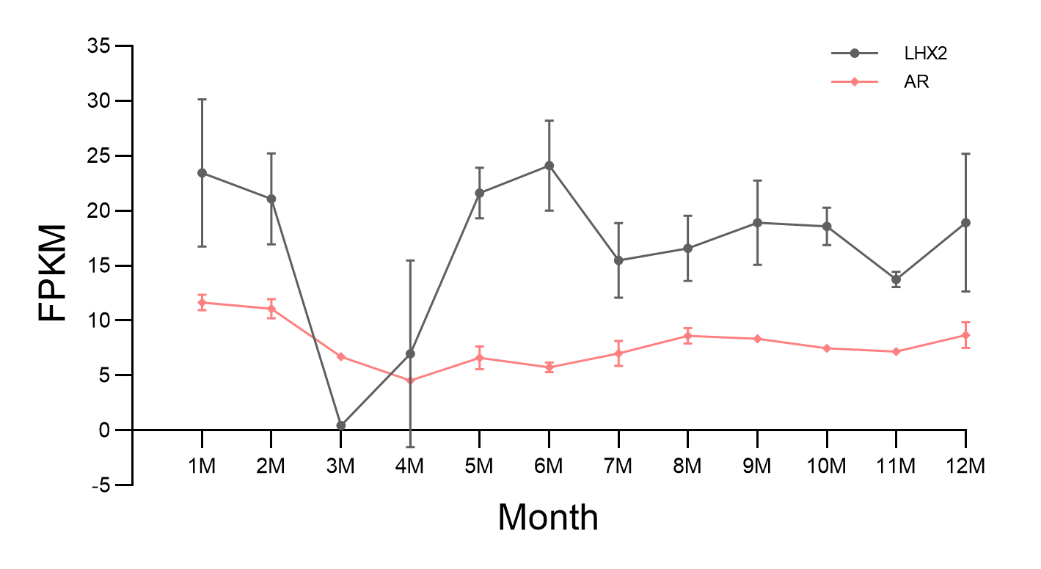


Supplementary Fig. 20 The expression of *LHX2* and *AR* in different months of a year.

The expression data is downloaded from the NCBI SRA database^10^ (PRJNA470971).


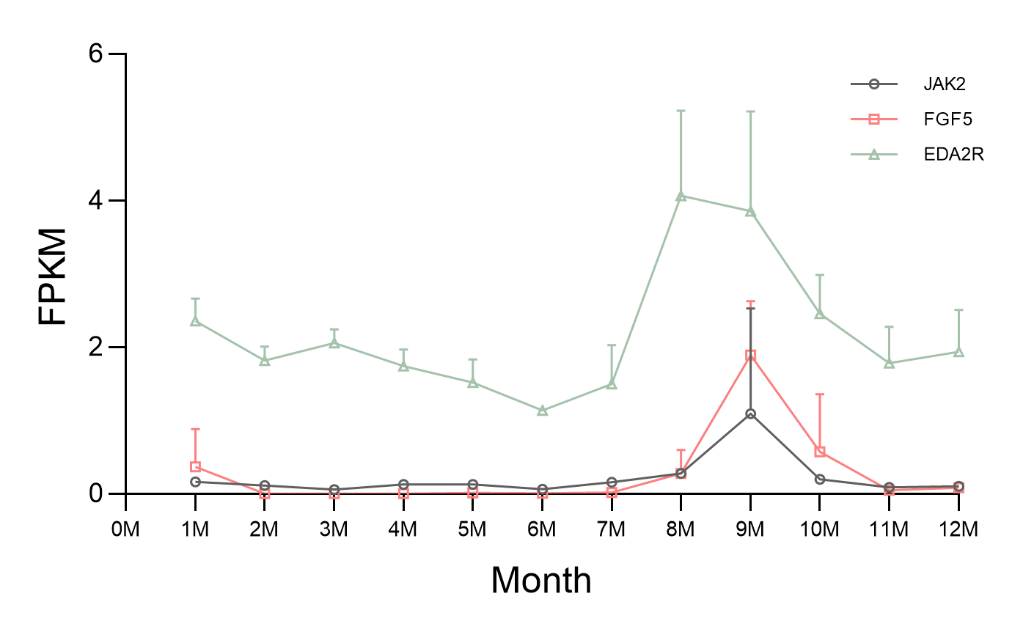


Supplementary Fig. 21 The expression of *JAK2* and *EDA2R* as well as *FGF5* in different months of a year.

The expression data is downloaded from the NCBI SRA database^10^ (PRJNA470971).


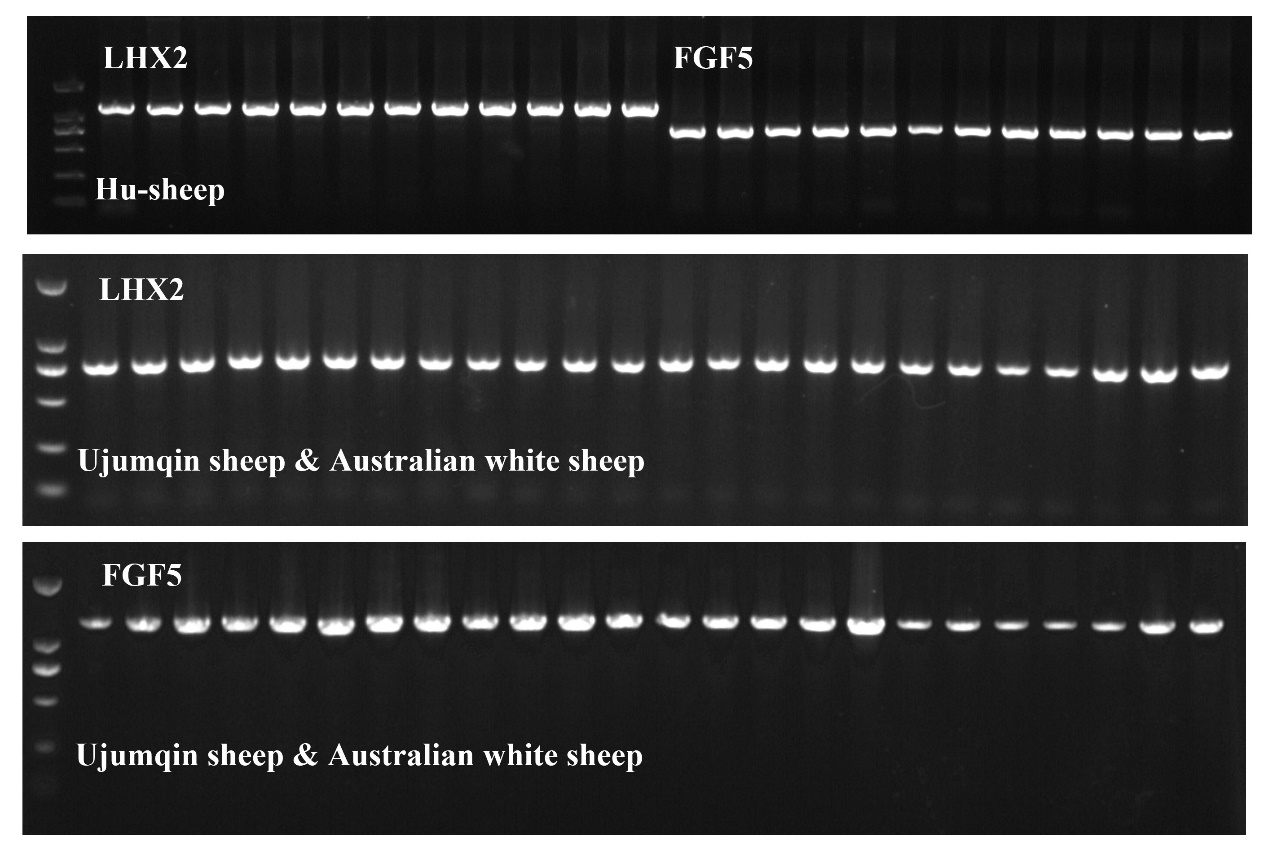


Supplementary Fig. 22 The PCR identification of deletions in sheep.


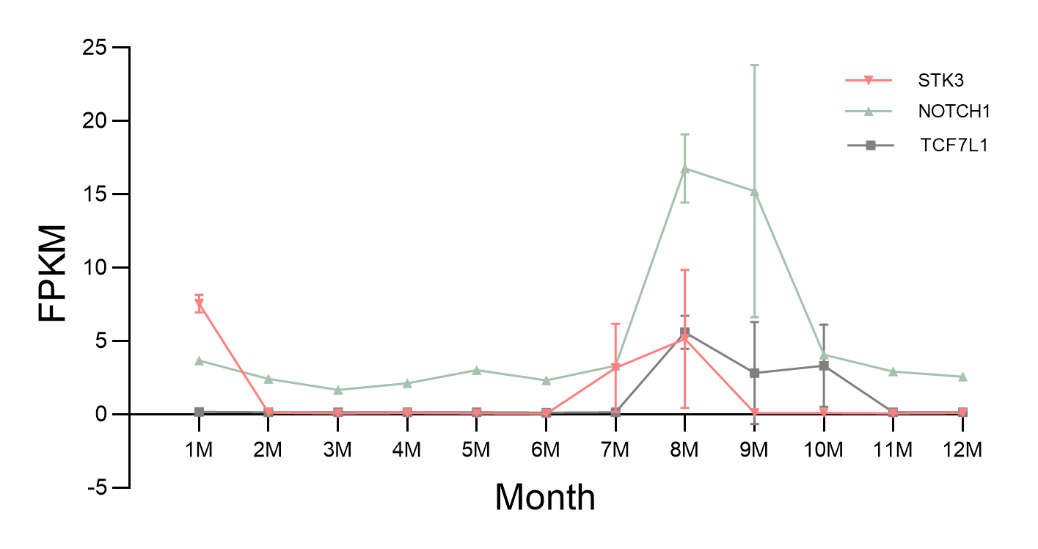


Supplementary Fig. 23 The expression of *STK2* and *NOTCH1* as well as *TCF7L1* in different months of a year.

The expression data is downloaded from the NCBI SRA database^10^ (PRJNA470971).

## Supplementary Tables

| Supplementary Table 1 Overview of sequencing information of 120 goats | | | | | | |
| --- | --- | --- | --- | --- | --- | --- |
| **Abbreviation** | **Sampling location** | **ID** | **Size_MB** | **Depth** | **Coverage** | **Mapping_rate(%)** |
| CDMC | Chaidamu, Qinghai | HXR0157 | 24722.84 | 8.09 | 99.65% | 99.19 |
|  |  | HXR0014 | 27535.99 | 9.33 | 99.70% | 99.21 |
|  |  | HXR0138 | 28576.42 | 9.43 | 99.72% | 99.2 |
|  |  | HXR0044 | 29417.06 | 10.04 | 99.71% | 99.22 |
|  |  | HXR1167 | 29859.92 | 10.18 | 99.68% | 99.36 |
|  |  | HXR0147 | 31318.85 | 10.51 | 99.72% | 99.19 |
|  |  | HXR0047 | 31448.91 | 10.46 | 99.73% | 99.2 |
|  |  | HXR1188 | 32735.31 | 11.07 | 99.71% | 99.28 |
|  |  | HXR1166 | 32867.20 | 11.27 | 99.71% | 99.3 |
|  |  | HXR171 | 34456.79 | 11.82 | 99.72% | 99.26 |
|  |  | HXR0981 | 34904.03 | 11.86 | 99.69% | 99.24 |
|  |  | HXR1321 | 30607.06 | 10.28 | 99.54% | 99.17 |
|  |  | HXR1750 | 26659.97 | 9.09 | 99.55% | 99.22 |
|  |  | HXR2752 | 28146.61 | 9.53 | 99.60% | 99.22 |
|  |  | HXR0908 | 28744.20 | 9.94 | 99.66% | 99.27 |
|  |  | HXR1630 | 33845.47 | 11.68 | 99.72% | 99.09 |
|  |  | HXR3386 | 30860.89 | 10.67 | 99.65% | 99.25 |
|  |  | HXR3388 | 30735.56 | 10.6 | 99.65% | 99.27 |
|  |  | HXR0022 | 26253.44 | 8.7 | 99.58% | 99.24 |
|  |  | HXR0045 | 29850.22 | 10.09 | 99.63% | 99.3 |
|  |  | HXR0126 | 28236.77 | 9.4 | 99.67% | 99.27 |
|  |  | HXR0170 | 28779.79 | 9.6 | 99.65% | 99.28 |
|  |  | HXR0186 | 27155.72 | 9.06 | 99.64% | 99.25 |
|  |  | HXR0370 | 33842.07 | 11.2 | 99.73% | 99.18 |
|  |  | HXR0711 | 34055.78 | 11.64 | 99.71% | 99.27 |
|  |  | HXR097 | 30425.80 | 10.06 | 99.68% | 99.26 |
|  |  | HXR0127 | 36488.06 | 12.6 | 99.72% | 99.27 |
|  |  | HXR0176 | 29880.23 | 10.19 | 99.65% | 99.28 |
|  |  | HXR2781 | 35434.27 | 12.17 | 99.71% | 99.28 |
|  |  | HXR3030 | 31065.93 | 10.6 | 99.68% | 99.17 |
| UC | Ujumqin,Inner Mongolia | WMG0010 | 29808.94 | 10.22 | 99.68% | 99.22 |
|  |  | WMG0013 | 25158.27 | 8.53 | 99.58% | 99.19 |
|  |  | WMG0016 | 32235.68 | 10.99 | 99.61% | 99.33 |
|  |  | WMG0017 | 32123.61 | 11.03 | 99.70% | 99.23 |
|  |  | WMG0004 | 45279.43 | 15.93 | 99.76% | 99.23 |
|  |  | WMG0005 | 37758.79 | 13 | 99.73% | 99.25 |
|  |  | WMG0006 | 43441.20 | 15.16 | 99.73% | 99.22 |
|  |  | WMG0007 | 35369.19 | 12.18 | 99.73% | 99.23 |
|  |  | WMG0008 | 39552.82 | 13.77 | 99.76% | 99.24 |
|  |  | WMG0009 | 30251.41 | 10.41 | 99.71% | 99.21 |
|  |  | WMG0011 | 29613.21 | 10.01 | 99.70% | 99.23 |
|  |  | WMG0012 | 39691.25 | 13.55 | 99.74% | 99.24 |
| CDB | Chengdu,Sichuan | CMS0019 | 28356.30 | 9.82 | 99.66% | 99.25 |
|  |  | CMS0084 | 34839.14 | 12.21 | 99.69% | 99.27 |
|  |  | CMS0177 | 34653.57 | 12.04 | 99.69% | 99.4 |
|  |  | CMS0330 | 32826.68 | 11.55 | 99.67% | 99.38 |
|  |  | CMS0037 | 31909.39 | 10.83 | 99.69% | 99.28 |
|  |  | CMS0088 | 39151.30 | 13.21 | 99.70% | 99.26 |
|  |  | CMS0100 | 28772.64 | 9.75 | 99.61% | 99.39 |
|  |  | CMS0406 | 27437.51 | 9.34 | 99.63% | 99.28 |
|  |  | CMS0026 | 33172.37 | 10.83 | 99.66% | 99.3 |
|  |  | CMS0053 | 31938.17 | 10.64 | 99.67% | 99.32 |
|  |  | CMS0065 | 25939.66 | 8.68 | 99.58% | 99.28 |
|  |  | CMS0431 | 28635.63 | 9.36 | 99.60% | 99.33 |
| GZB | Guiyang,Guizhou | GHS0004 | 16639.80 | 5.39 | 98.76% | 98.98 |
|  |  | GHS0013 | 20755.17 | 6.93 | 99.46% | 98.99 |
|  |  | GHS0010 | 24577.96 | 8.2 | 99.60% | 98.56 |
|  |  | GHS0001 | 26279.23 | 8.89 | 99.65% | 98.88 |
|  |  | GHS0027 | 27464.94 | 9.22 | 99.68% | 99.18 |
|  |  | GHS0017 | 27865.07 | 9.4 | 99.64% | 99.1 |
|  |  | GHS0026 | 29457.34 | 9.86 | 99.70% | 99.22 |
|  |  | GHS0025 | 37752.37 | 13.01 | 99.75% | 99.18 |
| JTB | Jintang,Sichuan | JTY0038 | 22812.64 | 7.84 | 99.56% | 99.2 |
|  |  | JTY0076 | 25960.19 | 8.93 | 99.66% | 99.23 |
|  |  | JTY0042 | 26355.43 | 9.26 | 99.65% | 99.25 |
|  |  | JTY0001 | 27932.10 | 9.67 | 99.62% | 99.24 |
|  |  | JTY0045 | 29104.36 | 9.92 | 99.68% | 99.21 |
|  |  | JTY0022 | 30724.89 | 10.59 | 99.70% | 99.03 |
|  |  | JTY0062 | 30799.82 | 10.83 | 99.71% | 99.21 |
|  |  | JTY0012 | 32126.81 | 10.9 | 99.71% | 99.1 |
|  |  | JTY0034 | 32642.38 | 11.22 | 99.72% | 99.09 |
|  |  | JTY0050 | 35249.33 | 11.95 | 99.75% | 99.22 |
| YNBB | Lanping,Yunnan | BBG97 | 68903.47 | 24.77 | 99.68% | 99.49 |
|  |  | BBG274 | 53166.99 | 19 | 99.64% | 99.54 |
|  |  | BBG3101 | 67090.35 | 23.88 | 99.72% | 99.62 |
|  |  | BBG6106 | 57484.93 | 20.78 | 99.69% | 99.56 |
|  |  | BBG7111 | 57943.70 | 20.91 | 99.67% | 99.53 |
|  |  | BBG8111 | 57948.10 | 20.75 | 99.70% | 99.51 |
|  |  | BBG974 | 56834.47 | 20.32 | 99.69% | 99.47 |
|  |  | BBG10101 | 52573.69 | 18.74 | 99.69% | 99.52 |
|  |  | BBG12142 | 67912.64 | 24.3 | 99.68% | 99.61 |
|  |  | BBG1674 | 55152.12 | 19.84 | 99.65% | 99.55 |
|  |  | BBG1790 | 61289.37 | 21.93 | 99.69% | 99.62 |
|  |  | BBG1897 | 69885.29 | 24.88 | 99.72% | 99.54 |
|  |  | BBG26101 | 55940.49 | 20.26 | 99.70% | 99.46 |
|  |  | BBG28106 | 67676.59 | 24.27 | 99.68% | 99.35 |
|  |  | BBG31107 | 74517.03 | 26.94 | 99.68% | 99.54 |
|  |  | BBG3674 | 58077.22 | 20.82 | 99.66% | 99.61 |
|  |  | BBG39133 | 67997.18 | 24.34 | 99.71% | 99.64 |
|  |  | BBG8094 | 59123.55 | 21.11 | 99.68% | 99.52 |
|  |  | BBG8166 | 59744.89 | 21.79 | 99.70% | 99.6 |
|  |  | BBG8266 | 56908.26 | 20.7 | 99.70% | 99.55 |
|  |  | BBG8396 | 67991.46 | 24.94 | 99.71% | 99.53 |
|  |  | BBG8990 | 59468.12 | 21.62 | 99.70% | 99.57 |
|  |  | BBG90100 | 64959.42 | 23.48 | 99.69% | 99.66 |
|  |  | BBG9195 | 59900.10 | 21.55 | 99.68% | 99.54 |
|  |  | BBG93108 | 64680.66 | 11.98 | 99.54% | 99.67 |
|  |  | BBG9590 | 55525.50 | 20.04 | 99.70% | 99.51 |
|  |  | BBG9896 | 60156.38 | 21.58 | 99.70% | 99.63 |
|  |  | BBG99111 | 61696.89 | 22.19 | 99.69% | 99.51 |
| JNG | Jining,Shandong | GRG43 | 80147.53 | 27.97 | 99.69% | 98.74 |
|  |  | GRG45 | 77785.36 | 26.92 | 99.57% | 98.73 |
|  |  | GRG47 | 70586.37 | 24.5 | 99.55% | 98.74 |
|  |  | GRG77 | 67499.73 | 23.42 | 99.64% | 98.76 |
|  |  | GRG76 | 58886.59 | 20.43 | 99.10% | 98.65 |
|  |  | GRG75 | 62095.07 | 21.69 | 99.58% | 98.79 |
|  |  | GRG15 | 74124.89 | 26 | 99.70% | 98.62 |
|  |  | GRG16 | 64099.62 | 23.07 | 99.68% | 98.74 |
|  |  | GRG17 | 58185.73 | 20.13 | 99.66% | 98.66 |
|  |  | GRG18 | 66355.72 | 22.34 | 99.65% | 98.53 |
| MT | Shangqiu,Henan | MTG0003 | 33073.79 | 12.05 | 99.71% | 99.3 |
|  |  | MTG0005 | 31190.80 | 11.44 | 99.71% | 99.29 |
|  |  | MTG0006 | 32001.89 | 11.67 | 99.72% | 99.29 |
|  |  | MTG0007 | 36583.23 | 13.23 | 99.72% | 99.3 |
|  |  | MTG0012 | 27846.19 | 10.16 | 99.62% | 99.3 |
|  |  | MTG0013 | 28180.39 | 10.31 | 99.67% | 99.26 |
|  |  | MTG0001 | 28026.49 | 10.39 | 99.66% | 99.34 |
|  |  | MTG0002 | 23833.58 | 8.79 | 99.58% | 99.28 |
|  |  | MTG0009 | 28787.71 | 10.5 | 99.67% | 99.3 |
|  |  | MTG0010 | 27156.25 | 9.93 | 99.62% | 99.29 |

| Supplementary Table 2 Sequencing information of 95 published goats used in this study | | | | | |
| --- | --- | --- | --- | --- | --- |
| **Abbreviation** | **Sampling location** | **SRR** | **ID** | **Size_Mb** | **Depth(X)** |
| IMC | Inner Mongolia | SRR4052649 | IMCG02 | 13352.12 | 5.15 |
|  |  | SRR4096727 |  |  |  |
|  |  | SRR4052677 | IMCG05 | 10378.24 | 5.92 |
|  |  | SRR4096728 |  |  |  |
|  |  | SRR4053244 | IMCG25 | 20533.36 | 7.12 |
|  |  | SRR4096730 |  |  |  |
|  |  | SRR4053410 | IMCG28 | 16367.42 | 6.06 |
|  |  | SRR4096705 |  |  |  |
|  |  | SRR4053460 | IMCG29 | 19661.99 | 6.95 |
|  |  | SRR4096706 |  |  |  |
|  |  | SRR4053519 | IMCG30 | 17400.57 | 6.37 |
|  |  | SRR4096707 |  |  |  |
|  |  | SRR4053642 | IMCG43 | 10480.34 | 4.17 |
|  |  | SRR4096720 |  |  |  |
|  |  | SRR4051874 | IMCG21 | 18138.44 | 6.33 |
|  |  | SRR4101814 |  |  |  |
|  |  | SRR4101803 | IMCG37 | 12121.82 | 4.73 |
|  |  | SRR4051950 |  |  |  |
|  |  | SRR4101805 | IMCG39 | 14024.62 | 5.1 |
|  |  | SRR4051959 |  |  |  |
|  |  | SRR4051960 | IMCG47 | 28440.14 | 9.08 |
|  |  | SRR4051961 |  |  |  |
|  |  | SRR4051919 | IMCG311 | 15150.43 | 5.29 |
|  |  | SRR4101817 |  |  |  |
|  |  | SRR4051941 | IMCG35 | 13081.32 | 5.05 |
|  |  | SRR4101820 |  |  |  |
|  |  | SRR4101821 | IMCG36 | 11200.72 | 4.55 |
|  |  | SRR4051947 |  |  |  |
|  |  | SRR4101735 | IMCG101 | 13212.90 | 4.64 |
|  |  | SRR4052046 |  |  |  |
|  |  | SRR4101737 | IMCG29 | 19661.99 | 6.95 |
|  |  | SRR4052247 |  |  |  |
|  |  | SRR4052353 | IMCG04 | 21710.11 | 7.55 |
|  |  | SRR4101744 |  |  |  |
|  |  | SRR4101745 | IMCG051 | 16082.35 | 4.36 |
|  |  | SRR4052354 |  |  |  |
|  |  | SRR4101749 | IMCG141 | 15530.00 | 5.54 |
|  |  | SRR4052120 |  |  |  |
|  |  | SRR4052177 | IMCG19 | 18957.64 | 6.5 |
|  |  | SRR4101750 |  |  |  |
|  |  | SRR4101751 | IMCG213 | 13769.74 | 5.3 |
|  |  | SRR4052202 |  |  |  |
|  |  | SRR4101747 | IMCG121 | 12505.18 | 4.68 |
|  |  | SRR4052075 |  |  |  |
|  |  | SRR4101753 | IMCG27 | 15395.76 | 5.75 |
|  |  | SRR4052224 |  |  |  |
|  |  | SRR4052234 | IMCG28 | 16367.42 | 6.06 |
|  |  | SRR4101754 |  |  |  |
|  |  | SRR4051772 | IMCG10 | 36865.38 | 11.57 |
|  |  | SRR4096722 |  |  |  |
|  |  | SRR4051866 | IMCG13 | 28479.47 | 8.98 |
|  |  | SRR4101802 |  |  |  |
|  |  | SRR4051917 | IMCG11 | 24991.59 | 12.38 |
|  |  | SRR4101816 |  |  |  |
|  |  | SRR4051928 | IMCG14 | 31556.88 | 10.34 |
|  |  | SRR4101818 |  |  |  |
|  |  | SRR4051939 | IMCG15 | 24113.19 | 8.04 |
|  |  | SRR4101819 |  |  |  |
|  |  | SRR4051964 | IMCG12 | 39729.48 | 12.8 |
|  |  | SRR4101808 |  |  |  |
|  |  | SRR4052103 | IMCG313 | 30156.49 | 9.86 |
|  |  | SRR4101748 |  |  |  |
|  |  | SRR4052272 | IMCG01 | 26447.13 | 8.33 |
|  |  | SRR4101738 |  |  |  |
|  |  | SRR4052346 | IMCG33 | 29215.81 | 9.71 |
|  |  | SRR4101741 |  |  |  |
|  |  | SRR4052349 | IMCG55 | 36349.01 | 11.9 |
|  |  | SRR4101742 |  |  |  |
|  |  | SRR4052525 | IMCG111 | 37597.81 | 8.58 |
|  |  | SRR4096703 |  |  |  |
|  |  | SRR4052590 | IMCG31 | 28631.05 | 9.39 |
|  |  | SRR4096725 |  |  |  |
|  |  | SRR4053363 | IMCG710 | 23137.77 | 7.63 |
|  |  | SRR4096704 |  |  |  |
| LNC | Gaizhou, Liaoning | SRR4064255 | LNC18 | 19120.07 | 7.23 |
|  |  | SRR4107102 |  |  |  |
|  |  | SRR4064259 | LNC21 | 18442.79 | 6.96 |
|  |  | SRR4107114 |  |  |  |
|  |  | SRR4067780 | LNC24 | 25718.73 | 9.33 |
|  |  | SRR4107116 |  |  |  |
|  |  | SRR4067798 | LNC33 | 25739.53 | 9.34 |
|  |  | SRR4107119 |  |  |  |
|  |  | SRR4068049 | LNC37 | 22317.17 | 7.98 |
|  |  | SRR4107104 |  |  |  |
|  |  | SRR4068051 | LNC38 | 17313.81 | 6.45 |
|  |  | SRR4107105 |  |  |  |
|  |  | SRR4068053 | LNC47 | 26501.94 | 9.35 |
|  |  | SRR4107107 |  |  |  |
|  |  | SRR4068056 | LNC52 | 25383.53 | 9.16 |
|  |  | SRR4107110 |  |  |  |
|  |  | SRR4068057 | LNC54 | 22658.95 | 8.16 |
|  |  | SRR4107111 |  |  |  |
|  |  | SRR4068058 | LNC56 | 24913.93 | 8.91 |
|  |  | SRR4107112 |  |  |  |
|  |  | SRR4067779 | LNC22 | 11526.85 | 4.75 |
|  |  | SRR4107115 |  |  |  |
|  |  | SRR4067781 | LNC31 | 14464.54 | 5.68 |
|  |  | SRR4107117 |  |  |  |
|  |  | SRR4067799 | LNC34 | 13730.86 | 5.37 |
|  |  | SRR4107120 |  |  |  |
|  |  | SRR4067800 | LNC35 | 12068.03 | 4.93 |
|  |  | SRR4107121 |  |  |  |
|  |  | SRR4067801 | LNC36 | 10564.92 | 4.46 |
|  |  | SRR4107122 |  |  |  |
|  |  | SRR4068052 | LNC39 | 15629.86 | 5.53 |
|  |  | SRR4107106 |  |  |  |
|  |  | SRR4068054 | LNC48 | 11129.55 | 4.47 |
|  |  | SRR4107108 |  |  |  |
| KO | Korean | SRR1265926 | KOG06 | 45009.41 | 14.92 |
|  |  | SRR1265927 | KOG07 | 44747.49 | 14.91 |
|  |  | SRR1265928 | KOG08 | 45333.81 | 14.94 |
|  |  | SRR1265929 | KOG09 | 44544.50 | 14.76 |
|  |  | SRR1265930 | KOG10 | 43077.48 | 14.39 |
|  |  | SRR1265931 | KOG11 | 45720.98 | 15.29 |
|  |  | SRR1265932 | KOG12 | 44948.16 | 15.11 |
|  |  | SRR1265933 | KOG13 | 46550.37 | 15.46 |
|  |  | SRR1265934 | KOG14 | 47704.28 | 15.73 |
|  |  | SRR1265935 | KOG15 | 45500.75 | 15.26 |
|  |  | SRR1265936 | KOG16 | 43953.58 | 14.59 |
|  |  | SRR1265937 | KOG17 | 43054.14 | 14.38 |
| ANG | Evry, France;South Africa;Madagascar | ERR4133570 | FANG0001 | 41597.54 | 13.93 |
|  |  | ERR4133551 | FANG0002 | 35697.88 | 11.49 |
|  |  | ERR4133594 | FANG0003 | 18590.68 | 6.53 |
|  |  | ERR4133600 | FANG0004 | 33849.04 | 11.46 |
|  |  | ERR4133602 | FANG0005 | 31924.81 | 10.83 |
|  |  | ERR4133549 | FANG0006 | 37378.87 | 10.28 |
|  |  | ERR4133646 | FANG0007 | 34118.43 | 11.55 |
|  |  | ERR4133649 | FANG0008 | 29679.00 | 10.08 |
|  |  | ERR4133643 | FANG0009 | 37437.20 | 12.86 |
|  |  | ERR4133597 | FANG0010 | 16980.35 | 6.01 |
|  |  | ERR4133588 | FANG0011 | 39509.40 | 13.07 |
|  |  | ERR4133619 | FANG0012 | 18814.75 | 6.54 |
|  |  | ERR4133615 | FANG0013 | 39342.96 | 13.72 |
|  |  | ERR4133622 | FANG0014 | 33621.92 | 11.7 |
|  |  | ERR4133550 | FANG0015 | 42170.35 | 13.13 |
|  |  | ERR4133591 | FANG0016 | 32655.34 | 11.11 |
|  |  | ERR4133618 | FANG0017 | 40490.15 | 13.93 |
|  |  | ERR4133606 | FANG0018 | 35121.24 | 11.88 |
|  |  | ERR4133625 | FANG0019 | 37295.04 | 13.03 |
|  |  | ERR4133628 | FANG0020 | 34410.02 | 12.12 |
|  |  | ERR3281402 | MANG0027 | 103932.50 | 29.9 |
|  |  | ERR3281403 | MANG0028 | 68239.26 | 22.11 |
|  |  | ERR3281404 | MANG0029 | 80376.99 | 25.78 |
|  |  | ERR3281405 | MANG0030 | 79312.28 | 25.62 |
|  |  | ERR3281406 | MANG0031 | 62641.33 | 19.91 |
|  |  | ERR3281407 | MANG0033 | 76354.83 | 21.87 |
|  |  | ERR4133525 | ZANG0374 | 41438.84 | 14.24 |
|  |  | ERR4133528 | ZANG0388 | 34554.79 | 11.94 |
|  |  | ERR4133517 | ZANG0397 | 31028.87 | 10.73 |

Supplementary Table 3 Sequencing information of Wild goats used in this study

| **Abbreviation** | **Sampling location** | SRR | **ID** | **Size_MB** | **depth** |
| --- | --- | --- | --- | --- | --- |
| IRW | Alamout, Iranian | ERR470100 | IRWG01 | 45083.46 | 15.74 |
|  |  | ERR470104 | IRWG04 | 43442.83 | 15.49 |
|  |  | ERR470106 | IRWG06 | 45847.73 | 15.35 |
|  |  | ERR340426 | IRWG26 | 37967.84 | 13.72 |
|  |  | ERR340328 | IRWG28 | 19454.66 | 7.24 |
|  |  | ERR340329 | IRWG29 | 17013.47 | 6.22 |
|  |  | ERR340330 | IRWG30 | 37395.69 | 13.64 |
|  |  | ERR340331 | IRWG31 | 34581.11 | 12.73 |
|  |  | ERR340333 | IRWG33 | 19084.62 | 7 |
|  |  | ERR340334 | IRWG34 | 37587.13 | 13.58 |
|  |  | ERR340335 | IRWG35 | 19737.88 | 7.26 |
|  |  | ERR340336 | IRWG36 | 19252.59 | 7.13 |
|  |  | ERR340338 | IRWG38 | 34183.70 | 12.48 |
|  |  | ERR340340 | IRWG40 | 36536.11 | 13.22 |
|  |  | ERR340341 | IRWG41 | 15342.29 | 5.67 |
|  |  | ERR340342 | IRWG42 | 19017.87 | 7.03 |
|  |  | ERR340343 | IRWG43 | 21705.31 | 7.97 |
|  |  | ERR340344 | IRWG44 | 33823.57 | 12.31 |
|  |  | ERR340345 | IRWG45 | 35354.22 | 12.85 |
|  |  | ERR340347 | IRWG47 | 37973.28 | 13.76 |
|  |  | ERR340348 | IRWG48 | 32400.27 | 11.56 |
| BS | Central Asia | SRR5803211 | BS1 | 40343.35 | 14.58 |
|  |  | SRR5803192 | BS5 | 34274.71 | 12.13 |
|  |  | SRR5803193 | BS8 | 48985.98 | 17.08 |
| FAG | Central Asia | SRR5803200 | FAG02 | 27401.58 | 9.71 |
|  |  | SRR5803204 | FAG05 | 90588.81 | 11.5 |
|  |  | SRR5803207 | FAG04 | 32469.98 | 31.11 |

BS (Capra sibirica)

FAG (Capra falconeri)

Supplementary Table 4 Sequencing platforms for 236 goats of 13 goat breeds.

| **Breed or Species** | **Abbreviation** | **Sequencing Platform** | **Length** | **Sample Size** |
| --- | --- | --- | --- | --- |
| Chaidamu Cashmere Goat | CDMC | BGISEQ-500 | 100 | 30 |
| Inner Mongolia Cashmere Goat | IMC | Illumina HiSeq2000 | 100 | 37 |
| Ujumqin Cashmere Goat | UC | BGISEQ-500 | 100 | 12 |
| Liaoning Cashmere Goat | LNC | Illumina HiSeq2000 | 100 | 17 |
| Iranian Wild Goat | IRW | Illumina HiSeq2000 | 100 | 21 |
| Matou Goat | MT | BGISEQ-500 | 100 | 10 |
| Korean native goat | KO | Illumina HiSeq2000 | 100 | 12 |
| Jining Grey Goats | JNG | Illumina Hiseq 2500 | 150 | 10 |
| Jintang Black Goat | JTB | BGISEQ-500 | 100 | 10 |
| Guizhou Black Goat | GZB | BGISEQ-500 | 100 | 8 |
| Yunnan Black Bone Goat | YNBB | Illumina Hiseq 2500 | 150 | 28 |
| Angora goat | ANG | Illumina HiSeq 4000 | 150 | 29 |
| Chengdu Brown Goat | CDB | BGISEQ-500 | 100 | 12 |

Supplementary Table 5 The genotype frequencies of the homozygous 582bp deletion (-/-) at *LHX2* locus for 15 goat breeds, obtained from whole genome sequencing.

| **Breed** | **LHX2(del/del)** | **Size** | **Genotype Frequency** |
| --- | --- | --- | --- |
| CDMC | 21 | 30 | 70.00% |
| YNBB | 8 | 28 | 28.57% |
| CDB | 1 | 12 | 8.33% |
| JNG | 1 | 10 | 10.00% |
| GZB | 1 | 8 | 12.50% |
| IMC | 22 | 36 | 61.11% |
| IRW | 17 | 21 | 80.95% |
| JTB | 0 | 10 | 0.00% |
| KO | 4 | 12 | 33.33% |
| LNC | 9 | 17 | 52.94% |
| MT | 4 | 10 | 40.00% |
| UC | 12 | 12 | 100.00% |
| ANG | 7 | 29 | 24.14% |
| BS&FAG | 1 | 6 | 16.66% |
| AG | 7 | 21 | 33.33% |

Supplementary Table 6 The genotype frequencies of the homozygous 504bp deletion (-/-) at FGF5 locus for 15 goat breeds, obtained from whole genome sequencing.

| **Breed** | **FGF5(del/del)** | **Size** | **Genotype Frequency** |
| --- | --- | --- | --- |
| CDMC | 23 | 30 | 76.67% |
| YNBB | 0 | 28 | 0% |
| CDB | 0 | 12 | 0% |
| JNG | 2 | 10 | 20% |
| GZB | 0 | 8 | 0% |
| IMC | 30 | 37 | 81.08% |
| IRW | 0 | 21 | 0% |
| JTB | 0 | 10 | 0% |
| KO | 0 | 12 | 0% |
| LNC | 17 | 17 | 100% |
| MT | 0 | 10 | 0% |
| UC | 0 | 12 | 0% |
| ANG | 1 | 29 | 3.45% |
| BS&FAG | 0 | 6 | 0% |
| AG | 0 | 21 | 0% |

Supplementary Table 7 The frequencies of the 582 bp deletion near *LHX2* with PCR amplification.

| Breed | **del/del** | **del/+** | **+/+** | **Sum** |
| --- | --- | --- | --- | --- |
| CDMC | 131(54.13%) | 91(37.6%) | 20(8.27%) | 242 |
| IMC | 8(66.67%) | 3(25%) | 1(8.33%) | 12 |
| UC | 30(75%) | 9(22.5) | 1(2.5%) | 40 |
| JNG | 2(16.67%) | 8(66.67%) | 2(16.66) | 12 |
| WSW | 0 | 2(16.67%) | 10(83.33%) | 12 |
| JTB | 1(8.33%) | 5(41.67) | 6(50%) | 12 |

The frequencies of homozygous (del/del), heterozygous (del/+) and wide type of the 582 bp deletion near *LHX2*, obtained from PCR amplification.

Supplementary Table 8 The frequencies of the 504 bp deletion near *FGF5* with PCR amplification.

| Breed | del/del | del/+ | +/+ | Sum |
| --- | --- | --- | --- | --- |
| CDMC | 194(80.16%) | 45(18.6) | 3(1.23%) | 242 |
| IMC | 12(100%) | 0 | 0 | 12 |
| UC | 3(7.5%) | 19(47.5%) | 18(45%) | 40 |
| JNG | 3(25%) | 8(66.67%) | 1(8.33%) | 12 |
| WSW | 0 | 1(8.33%) | 11(91.67%) | 12 |
| JTB | 0 | 0 | 12(100%) | 12 |

The frequencies of homozygous (del/del), heterozygous (del/+) and wide type of the 504 bp deletion near *FGF5*, obtained from PCR amplification.

Supplementary Table 9 Published ancient samples used in this study.

|  | | |
| --- | --- | --- |
| **Sample** | **Period** | **Bioproject** |
| Potterne1 | Bronze Age | PRJEB26011 |
| Qazvin1 | Bronze Age | PRJEB26011 |
| Bulak2 | Bronze Age | PRJEB26011 |
| Kohneh2 | Bronze Age | PRJEB26011 |
| Acem1 | Bronze Age | PRJEB26011 |
| Acem2 | Bronze Age | PRJEB26011 |
| Azer3 | Bronze Age | PRJEB26011 |
| Darre2 | Chalcolithic | PRJEB26011 |
| Fars4 | Chalcolithic | PRJEB26011 |
| Geor2 | IronAge-Medieval | PRJEB26011 |
| Kazbeg1 | IronAge-Medieval | PRJEB26011 |
| Azer4 | IronAge-Medieval | PRJEB26011 |
| Semnan1 | Neolithic | PRJEB26011 |
| Semnan10 | Neolithic | PRJEB26011 |
| Semnan13 | Neolithic | PRJEB26011 |
| Monjukli8 | Neolithic | PRJEB26011 |
| Blagotin3 | Neolithic | PRJEB26011 |
| Lur12 | Neolithic | PRJEB26011 |
| Blagotin1 | Neolithic | PRJEB26011 |
| Blagotin16 | Neolithic | PRJEB26011 |
| Blagotin2 | Neolithic | PRJEB26011 |

Supplementary Table 10 Samples for PSMC analysis

| **Sample** | **depth(X)** |
| --- | --- |
| BBG31107 | 26.94 |
| FANG0017 | 13.93 |
| WMG0004 | 15.93 |
| CMS0088 | 13.21 |
| HXR0127 | 12.6 |
| IRWG01 | 15.74 |
| KOG14 | 15.73 |

Supplementary Table 11 Primers used for amplification of deletion sequence

| Primer names | Sequence 5’-3' | Length (bp) |
| --- | --- | --- |
| LHX2_del_F2 | CAGTACGGAGCAAGTAAACGG | 630 bp for del/del  1214 bp for +/+  630/1214 bp for del/+ |
| LHX2_del_R2 | ACCATTCCACTTGTCCACCT |  |
| FGF5_del_F1 | ACAGCGTGTGATCTTTTCTCTG | 235 bp for del/del  742 bp for +/+  235/742 bp for del/+ |
| FGF5_del_R2 | TCTTGGTCTGGCTGTGATCA |  |

## Supplementary sequence 1

Distal enhancer-like signatures are shown in red, the CTCF binding site is displayed in bold, and the 582bp deletion sequence is underlined.

>goat:chromosome chromosome:ARS1:11:94266769:94278930:1

ATACGAGTTGCCATGACTATTCTGTTGCTTGGATTCATTCACCATCTACTCTCACCAGTTGTACTAATTAAATCTTCAGAGCTCTTCCAGGATCTCTATCGCTTTCTTTGCTTCAGAAAGTCAATCAGAGTTTATTGCCCCGAAAAGGTAGGATTCAGAGTCAACATTTACTAAATGAGTAGTATGTGTCCAGCAATGGGCTACTGGCTTTCATATCTTTCAAAAACCCTGAGGGGAGTATTAACACTACTCTCTTCTTACTGAAAAGATTAAGTTGCCTAGGGCAACATGGCTAGGGAGGATCAAGATACACAGGAAGAATTTCAACAAAACCATTATGACTTGAAGCCTGGTGCTCTCTCCTCTTGTCCTTTTTCTTGGGAAAAGCTAGCAGAAGCACGGCTGTCTGAATCATCTAGCACGGAAAACACATAAATATCAAGACAGCACAAGAGAAAAAACATGAATTTTCACAAGGTCTATATGAACTCCTAATGATATAGTCTAACCTCACATCATACTTCATTCGCTCATCAGTGACCATGCTTTCAGTTTGCAGAAATAAAAAAAAAATTTGAGTTCTTGACTTCTGACCACATAGTAGGTGCTCCAATTGTGTTAACAAAATCCACACAGATGTCTGCCAATGTGAGTGCCATTAGGGGTTTTTCAAGTATATTAAGATGTCAGTGACTTGGAAGGAAGTTATAACCAACCTAGATAGCATATTCAAAAGCAGAGACATTACTTTGCCAACTAACACCCATCTAGTCAAGGGTGTGGTTTTTCCAGTAGTCATGTATGGATGTGAGAGTTGGACTGTGAAGAAAGCTGAGTGCCGAAGAATTGATGCTTTTGAACTGTGGTGTTGGAGAAGACTCTTGAGAGTCCCTTGGACTGCAAGGAGATCCAACCAGTCCATTCTGAAGGAGATCAGTCCTGGGTGTTCTTTGGAAGGAATGATGCTAAAGCTGAAACTCCAGTACTTTGGCCACCTCATGCAAAAAGTTGACTCACTGGAAAAGACTCTGATGCTGGGAGGGGTTGGGAGCAGGAGGAAAAGGGGACGACAGAGGATGAGATGGCTGGATGGCATCACCGACTCAATGGATGAGAGTTTGAGTGAACTCCGGACAGGGAGGCCTGGCATGTTGCAATTCATGGGGTCGCAAAGAGTCGGACAAGACTGAGTGACTGAACTGAACTGAACTGACTTGAATATCACCTCTTAGTGTTTTTTGGTAGAGATCTGATTCTTAAAAGAGTCATATAAAAAAGTTATCAATCCTATAATCTAAGAAACTGACTACACCACTAAAATAATACACAAGATACTCTGCCAATGTTCTCAATGCAATGGCTGAAAAGAAACATCACCTCTCTAGATCCATTTATACAGATTTATTTAAAACTACCCTAGTGGACTACATCAGCATCCAATGGTACTGAGAGCTTAAATTACTCTTAGCTCTGTCCTGTTCTAGACTGTTGTTGCTGCTGCTGCTAAGTTGCTTCAGTCATGTCTGATTCTTTGCGACCCCATAGACTCAGAGCTCTTTCAGAAATATGGAAATAATTTTTAGTGCTAATCTGTACTCACAAATGGAGAAGGAAATGGCAACCCACTCCAGAATTCTTGCTTAGAGAATACTGTGGACGGAGGAGCCTGGTGGGCTGCTGTCCATAGGGCTGCACAGAGTCGGACACGACTGAAGCGACTTAGCACGCATGCATGCACCGGAGAAGGAAATGGCAGCCCACTCCAGTGTTCTTGACTGGATAATCCCAGGGATGGTGGAGCCTGGTGCGCTGCTGTCTATGGGGTCGCACAAAGTCGGACACGACTGAAGCAACTTGGCAGCAGCAGCAGCAGTACTCACAAATGCCTTCTAAACAAACTTAGTCAGCCTTTATTATATAGTCTCAAGATGAAAAGGACTCTATTGATGCAAAATAATCAAAGAATAAATACTGTCTTAGAGAACTGGTCCTGAATCTGGTTATGTATTAGTAATTTGGGAAACCTTAAAAAAATAGACATCCCACAGTCCCATCTTAGACTTTTTGAATTATTATTATAATAAAAATTTAAGTTCTTGGGTACTACCTCTACACATTCTGATTCAAAAGGTCTAATTTTTATTTAAATTGGAGAATAATTAGTTTACAACATTGTGATGTTTTTAGCCATACATCAACATGAATCAGCCACAGGTATACATTTGTCCCACCCCATCCTGAATCCCCTCCCACCTCCTTCCTTACCCTATTCCTCTGCGGTTGTCCCAGAGCATCAGCTTTGAGTGCCTTGCTTCATGCATCAAACTTGCACTGGTCATCTATTTTACACATAGTAATGTACATGTTTCAGTGCTATTGTCTCAAATCATCCCCTGCTCTCCTCCTCTCCCTCTGAGTCCAAAAGTTTGTTCTTTACATCTGTGTCTCCTTTACTGCCCTGCATGTAGGATCATTGGTATTGTCTTTCTAAATTCCATATATATGCGTTAATATATAGTATTTGTCTTTCTTTTCAACTTATTTCACTCTGTATAATAGGCTCCAGGTTCATCTACCTCATTAGAACTGACTCAAATGCATTCCTTTTTGTAGCTGAGAACTTAAATTTTTAATAAGCTCTCTGAGTGATTTCTAACACAACTAACTCAGGGATCAGCATTTGGGAATCACTAACAATCTGAATCCTCTGTCTAGTCCAAAACATGTGCTCAATGTGTTGAAGCAAGAAGGGCAGGAGAGGAAGGTAAGAACTAGTAGGGAGACCGTGATTGGTTGAAATCACAGCAATATTTAAAGTACTACAAATTAAAGTGTTGTTACTCTATATATTATATAGCTTATACATTCTCTATAACATCAATGTTCTTCATTCTTATAGTCTAACATGAACAAGAGAAAAGGAACTCGCACATAATTAAATGAAGAACATAGTTCAAAAAGCAGCCAAGTGTATTTACATTTAAAGACCAGAGAAAAACCACATAAATAAAACATACCAATCTTTTCTATTTCCTGCCAATGGAAGTTCCTTTCATTTATTTCTAACAGTTTTTAATATTCTCTGAGCCACAATAAAATTTTATGTGATGGAGATTAGAGAGGCTATGCTATTTGAAAGTTTGGCTGCAGAGTTTGTATTTCATGCCACTTCATTACATTTTGTTAAGGATTTCCCTCTTCACCAAAAGGGATTAGTTTTCTTCTCACTTCTTTGAATACTCTTCTTAGGTTGTTAGTGTGAACTAGGTTAATCTCTCTTTTAACATATAAAAGATGAACAAACAAAATGTTCAATTTATTTTATTTTTTTAAATGGAACACTAAATTTAGTCTTAGAATTCTCTGTTTGGTAAGTTTCCAGTTACAAGGTCTTGGCTTCCCTTGAGCACAGTTTGAAACAAAATAATGCAGTTTGTTCTGATGGCTGGAACATGCAGAGTGGAGTGTGCAGGCAAAGAGGCTCTGGCTCTGGTCCAGTATTTCAAACCTTCTTAAACTGCCACTCTCATCTAGGCTTCAAGGGCTCCAGATAAGCAAACAGGATTAGTTATTTCTAGCCCAGAAGAGCTAGAAAAGCTTTATACACATCATTTAGGGACATAGCCCACTCTACAAGTCAGGGTAGTCATGACCAGGTAGATCTGATCAGGAAAGAAGTAAAGATGAAGCTAAAGATCTGAAACATTCCCTAGTCCACAAACTGACTCTTCTTAGGTCACAAAACCAGGTGGTGCTAAGACACTGGATGCTGTGAATGGAAGTGGATAGGACAGGACTGGAGCAAGGTTGGGAAGTTCCCCATCCCTTCATGCCAACACTCAAGTGTCTACTAAGGCCACAACTGAGGGTGCTGGGGGGAAAAAGAAATACCACCCACCACTCTCCAGGCCATCAAGTCCTGCCCAAGGCACATTTCTGGGACTAATTGGAAGAAATTGACGGAGAGATATTGGGCTTTTTTTTTTTTTTTTTCCTTCTCTACATGGTGTATTTGGAGAAGGAAACAGCAACCCACTCCAGTATGCTTGCCTGGAAAATTTCATGGACAGGGAGCCTGGTGGGCTCTAGTCCATGGGGTCACAGAGTCAGACACGACTGAGTGATTAAATGACATAACATACTATTAGAAAAATAGTAATTCTTTTCATAGTTGTGGGTATCACACAAATAATTAGGTGTGGAGGCTCAATTAATTAGGTAACAGCTTTGCCACCAAATTGTGCTTCCTTCCTAAGAACTTGTTTTAAAAAGCATCTCTTAAGTGTGACAGATATTCTTAACAGTGTGCCTCTGGTTCTCGTGACAGTTCCCACACAAGGATTCTGATGTTTGTTTGAGAGGTGACACCCAATCAGTGTAACCTGGTCCCCACAGCAAGCTCTTTGAAGGGGCCAAGATTCATCTGTGTAACTCCTGGTTGTTACTCCCTTTGCTGAAGCCAAAGCCTCCCTTCCTCTGACACATCATCATAATGACCCACCTGCATGTCCTTTTCTTCTATTACGAGGGCATAACCTTGGGCAAGAGAACCCATCTCTCTGAGTGGATGGATCATTCTGTAAAATGGGGATAATATTGCTCTTCTAGGAATTCTGTTGCAATTAGATGATATTATTTCTATAAAGGTTTTATTTTAGACCTACTTGCTATCCACAAGAGTAACCAAAAAACAATCTGAGTTAAACTCAGATTTGAAAATCTGGAAGAAAAATGGGGCCTTTGTGCTAGAAGAAATCTCACTGAAGCATTTACATTAGAGAACAAGATGTAGTTAAATCTTCCCTATTTATCATTTATAAAGGAAAACAATCAATATACACTGCACAAAAAGAACAAAAAGTTAACATTTTTCTTACACAGAATATCACAACATTATTGACCGGGAAAGCCT**GAAATGAATATTACCTGACTCTGAACTAAACATGGCTGGGTTTGTCTCTATTGACACACAGTCAGTTTTATGAAACATGGCACTCTGGGATGTTCAACAGCTTTCATCTTTCAGATGCTTGATGGCTCTGAGAAGCATCAAGCCTACATCTTTATGAAACAAAATTGAGAAAAATGTGTTTTGTCAAGGTATGGAAGGCCACATCCGGATCTGTCAAGAAAGGGTCATCAAAAACTTCAGGCTACAGTATTCTTTGCATTTTCCTTCATCAAAAAGAAAATAAAAAGTCAGTACGGAGCAAGTAAACGGTAACAATTAGAAAGCTTTTA**AACTCTAAAATTGAAGAGGTCCAAAGGCTTGGATAAAAGGGATGTCTACTCAGTTACTTATCCCCAGAGAAACAACACAAAACAGATCAGGGTGTTGCTGATCCCCTATGAGGTAAAAGAAGACCTTCTCCTTCCCTAAAGAGATATCCACCATCAATTAGTAAAACTTAGAACTCAGTTAAGAATCAACTGTCTGTTTAAAAGTTTTTAAGTCAAAGTCATGTATTTATAGCCTGAAAAACAAAACAAAAAGTGTTAAAGGCTTATACTACTGACAAATAGCAGCCCATCTCATGATATCCCTTACCTTCAATCAAAAGTAATCACATTCAACACAATATTTATCTCCTTATTTTTAAAAAGTATTTAATTTAAAAATTTAAACATTAATCTTTGACTTCCCATTATAGATATTAATAATTTATCAAGAACATATACATATACCTTATGCCAGACTGTTCTAGGAGTTTTGCAACTCGTTTTATTTGACATGAGGTCACTGGTGACCAAACAAAAACTGTTTCAGTGGAGGCAGGGGCTAAATCACAACACAAGACAGAGGAGAGAATGAGAAGAGAGGAATTAGACAGTAAACAGAAGCAACTCTCTTGAGGAATTGCATTCTAAAAGGAAGAAAAGAAATGGGATAAAAGAGATGTGTTTAAGGATGTTTTGTTTTGAAGTTTGGGAGACACTATACTGTGCTAGTAAGTGCATAAGACTGTTCAGTTCAGTCGCTCAGTTGTGTCCGACTCTTTGCGACCCCATGAATTACAGCACGCCAGGCCTGTACCAGTAAAGAAAACAAAAAACAAAAAATGATGCAGGAGAGAGAAAGCTCAAGGCTGGAGGAATTCCGTAAGGTGACAAGAAGGATGCTGCAGTGTTAGCTTTCCCACAAGGCACTCCTTGGTCATCTAGCTAAACTGCAATCCCTGCTACACCAGTCGCCATCCACTCTCCTCCTCTGCTTAATTGTACTCCTTGTACTTAGAACTAGCTACTATTCTATATTTTCAACCCACCTCATTTATTGATCTACTCGACTAGAACATAAGCTTCATATGAGGACAGGGACTTTTGCCTATTTTGTTCACTGCTGTATCACCAGTACCTATGACAGTGCCACAAAAACTATTTGCTGAATGAAGTCAGGTGGACAAGTGGAATGGTTGGCCTAGACAGAATGGACAGTTCATCCACAATGGAGAAGGTGAGTGGAGGACTAGCTACCGCAGGTTGACAGGTGTGGTAGGGGCTGGTGCCACTCTCTCCTGAGCGCTTTCATTAGATAAAGATGGAATTTTAGGTAAGAAATATTTTTCCCTTAGAAGTTTAGGCACTGCTTCACTATCTTCTCGTTTCTAGAACTGCCACTGCGAAATGTTGTGCAAGTCTGATTCTTAATGTTTATACAATGACTACTTTGTCTCTATCTCTGTATTGTAAAATTACATGATAAAAAGGGCTTTTTAACTTTCAAAGATTTCCTGAAGCCAAAACCTCCCTTCCCCTGACACTACATCATAACGACCAATGTGCCTGTCTCTTCTATGACTACCACGTGGCCTTAGGCAAGTTAACCCATGTATCTGAGTGAGTGGGCCATTCTGTAAAATGGGGATAATATTGCTTTTTAGACTTTTAAAGAATTCTGAA

## Supplementary sequence 2

S/MARs sequence are shown in red, S/MARs sequence from [http://transfac.gbf.de/SMARtDB/], release 2.0^11^. The 532-bp conserved sequence is underlined.

>Ins sequence(551bp)

GAAAAACAAAACAAAAAGTGTTAAAGGCTTATACTACTGACAAATAGCAGCCCATCTCATGATATCCCTTACCTTCAATCAAAAGTAATCACATTCAACACAATATTTATCTCCTTATTTTTAAAAAGTATTTAATTTAAAAATTTAAACATTAATCTTTGACTTCCCATTATAGATATTAATAATTTATCAAGAACATATACATATACCTTATGCCAGACTGTTCTAGGAGTTTTGCAACTCGTTTTATTTGACATGAGGTCACTGGTGACCAAACAAAAACTGTTTCAGTGGAGGCAGGGGCTAAATCACAACACAAGACAGAGGAGAGAATGAGAAGAGAGGAATTAGACAGTAAACAGAAGCAACTCTCTTGAGGAATTGCATTCTAAAAGGAAGAAAAGAAATGGGATAAAAGAGATGTGTTTAAGGATGTTTTGTTTTGAAGTTTGGGAGACACTATACTGTGCTAGTAAGTGCATAAGACTGTTCAGTTCAGTCGCTCAGTTGTGTCCGACTCTTTGCGACCCCATGAATTACAGCACGCCAGG

**Supplementary Reference**

1. Huang, J. et al. A reference human genome dataset of the BGISEQ-500 sequencer. *Gigascience*. 6, 1-9 (2017).

2. Li, H. & Durbin, R. Fast and accurate short read alignment with Burrows-Wheeler transform. *Bioinformatics*. 25, 1754-1760 (2009).

3. Letunic, I. & Bork, P. Interactive Tree Of Life (iTOL): an online tool for phylogenetic tree display and annotation. *Bioinformatics*. 23, 127-128 (2007).

4. Schiffels, S. & Durbin, R. Inferring human population size and separation history from multiple genome sequences. *Nat. Genet.* 46, 919-925 (2014).

5. Lai, F. N. et al. Whole-genome scanning for the litter size trait associated genes and SNPs under selection in dairy goat (Capra hircus). *Sci Rep*. 6, 38096 (2016).

6. Wang, L. et al. Understanding the Genetic Domestication History of the Jianchang Duck by Genotyping and Sequencing of Genomic Genes Under Selection. *G3 (Bethesda)*. 10, 1469-1476 (2020).

7. Zhou, Y. et al. Metascape provides a biologist-oriented resource for the analysis of systems-level datasets. *Nat. Commun.* 10, 1523 (2019).

8. Henkel, J. et al. Selection signatures in goats reveal copy number variants underlying breed-defining coat color phenotypes. *PLoS Genet.* 15, e1008536 (2019).

9. Wu, Z. et al. Using WGCNA (weighted gene co-expression network analysis) to identify the hub genes of skin hair follicle development in fetus stage of Inner Mongolia cashmere goat. *PLoS One*. 15, e243507 (2020).

10. Yang, F. et al. Skin transcriptome reveals the periodic changes in genes underlying cashmere (ground hair) follicle transition in cashmere goats. *BMC Genomics*. 21, 392 (2020).

11. Liebich I, et al. S/MARt DB: a database on scaffold/matrix attached regions. Nucleic Acids Res. 30, 372-374 (2002).
