# Supplementary material for: Selection signatures in goats reveal a novel deletion mutant underlying cashmere yield and diameter
Source: Gigascience. 2022 Nov 3;11:giac107. doi: 10.1093/gigascience/giac107 (PMC9633279; doi:10.1093/gigascience/giac107)
Supplement: giac107_Response_to_Reviewer_Comments_Revision_1 [file giac107_response_to_reviewer_comments_revision_1.pdf]

Author's Response To Reviewer Comments

Close

Reviewer reports:
Reviewer #3: The manuscript written by Han et al. is an interesting study aiming at investigating the genetic mutations underlying cashmere yield and its diameter. In this manuscript, the authors have sequenced 120 Chinese native goats including two cashmeres (n=42) and six ordinary breeds (n=78), and also downloaded published whole-genome sequencing data from 116 individuals (five different goat breeds). The authors performed genome-wide selective sweep analyses based on the genomic variation data, and identified a 582bp deletion of LHX2 gene, which may be important for cashmere production and for its diameter.
In general, the logic of the paper is clear and the authors have done corresponding experimental verifications in both human and mice species. However, the manuscript needs to be further improved.

1: In Fig 1C, the author classified two distinct cashmere goats (UC and IMC), however in the Supplementary Table 1 there is only one group (UC). Please check this.
Re: In Supplementary table 1, a total of 120 domestic goats (include UC) were selected for genome resequencing. In Supplementary table 2 and 3, we downloaded the published whole-genome sequencing (WGS) datasets of 116 individuals (include IMC) from five breeds. Therefore, IMC sequencing information in the Supplementary Table 2.
2: Please check the below sentences and see how Supplementary Table S7 and Supplementary Table S8 would match to this paragraph!
"To further evaluate whether these two deletion variants were related to cashmere traits, we selected 235 CDMC goats with cashmere yield (Supplementary Fig. 16, Supplementary Table s7) and 581 CDMC goats with fiber diameter records (Supplementary Fig. 17, Supplementary Table s8) for association analysis".
Re: Thanks for pointing it out. It is a mistake, "Supplementary Table s7" here is corresponding to Supplementary Table s10 and "Supplementary Table s8" is corresponding to supplementary Table s11. We have modified it in the new vision of manuscript and marked in green.
3: In the "Phenotypic analysis" section (P 25). Please provide more details about the phenotypic samples that you have applied for genomic correlation analysis. Did you take phenotypic samples from the same individuals that you have sequenced in this study? If no, what is the average age of the animals? Whether the age factor is corrected as a co-variable in this analysis? The wool production and its diameter is greatly depending of the age of the animals, which finally can affect your results.
Re: Thanks very much, it is a good question. We added some details about the phenotypic samples (including ages, gender and Surveyor information) in the Supplementary Table s10 and s11. The samples include 235 CDMC goats with cashmere yield (Supplementary Fig. 16, Supplementary Table s10) and 581 CDMC goats with fiber diameter records (Supplementary Fig. 17, Supplementary Table s11). Each individual was genotyped for both 582del and the 504del with developed PCR based method rather than re-sequencing (see Materials and methods). The association results showed that the 582del and the 504del was significantly associated with cashmere production (P value = 0.0061 and P value = 0.0113 for the 582del and the 504del, respectively). The 582del was also significantly associated with fiber diameter (P value = 2.74e-04), but the 504del was not (P value =0.348).
The age of goats are 2 or 3 ages, we have evaluated the effect of ages on diameter and yield, respectively, but the correlation coefficient was quite small (0.0754 for yield and -0.069 for diameter, P value >0.05), so we removed them from linear model for avoiding over-fitting. We have modified it in the new vision of manuscript and marked in green.

4: Fig. 5e. Please add the P value, corresponding to the below sentence.
"The 582del was also significantly associated with fiber diameter (P value = 2.74e-04), but the 504del was not (Fig. 5e)."
Re: Thanks for pointing it out. We added it in the text and marked in green.

5: "FGF5 enhancer deletion may have occurred during artificial selection or secondary domestication in the Kashmir region of Afghanistan and gradually migrated north of the Mongolian Plateau." What is your robust evidence to confirm this hypothesis? When the British entered Kashmir from the India, they found

that cashmere was raised. But can we prove this is the place of origin?
RE : Thanks very much, it is a good question. In fact, the origin of the domestic cashmere goat remains uncertain and controversial. Some authors suggested that the Cashmere goat breed probably originated from the Capra falconeri, a large Capra species native to Central Asia, the Karakoram, and the Himalayas (Ganai et al., 2011). In addition, some studies also have suggested that an second domestication in Pakistan gave rise to the Cashmere breeds (Meadow 1996; Luikart et a., 2001). On other hand, Chinese cashmere goats, however, do have the distinction of growing more underwool than other double coated goats and also of having a greater proportion of white animals. Some authors suggested that the concentration of cashmere goats moved from the western end of the Himalayas to north west China during the last 150 years (Ryder 1993). Based on this evidence, our study hint that FGF5 enhancer deletion may have occurred during artificial selection or secondary domestication in the Kashmir region of Afghanistan and gradually migrated north of the Mongolian Plateau. However, this evidence is not very robust and need more research data to support.

REFERENCES

Ganai, Tasleem & Misra, Siddhartha & Sheikh, Feroz Din. 2011. Characterization and evaluation of Pashmina producing Changthangi goat of Ladakh. The Indian journal of animal sciences. 81. 592-599.
Luikart G, Gielly L, Excoffier L, Vigne JD, Bouvet J, Taberlet P. 2001. Multiple maternal origins and weak phylogeographic structure in domestic goats. Proc Natl Acad Sci U S A. 98(10):5927-5932.
Meadow, A. 1996. Animal domestication in the Middle East: a revised view from the eastern margin. Pp. 295–320 in G. Possehl, ed. Harappan civilisation. Oxford and IBH, New Delhi.
Ryder, M. L. 1993. The use of goat hair: an introductory historical review. Anthropozoologica.

Close
