# Supplementary material for: Selection signatures in goats reveal a novel deletion mutant underlying cashmere yield and diameter
Source: Gigascience. 2022 Nov 3;11:giac107. doi: 10.1093/gigascience/giac107 (PMC9633279; doi:10.1093/gigascience/giac107)
Supplement: giac107_Response_to_Reviewer_Comments_Original_Submission [file giac107_response_to_reviewer_comments_original_submission.pdf]

Author's Response To Reviewer Comments

Close

Reviewer reports:
Reviewer #1: Nicely written paper on selection signatures for Cashmere goats with detailed analysis and possible deletion
1. * How was the optimal size of K determined in admixture?
Re: The reviewer raises a good question. K is the assumed to be the population subgroup or ancestor number. In order to
2. * Please review the formatting on the manuscript, for example page 5 and page 6 figure have some formatting errors.
Re: changed, thank you.
3. * Was sample size enough for all the comparison? What was the power of study design.
Re: The referee raises good points. In population selective sweep analysis, usually more than three subgroups/subspecies
designed the sample size so that significant selection signals between populations could be detected in the genome.
4. * How the results from mouse and human cell line justified for comparison with goat?
Re: Thanks very much, it is a good question. Firstly, Insulators, first identified in Drosophila, are DNA sequence elements
illustrates the functional conservation of insulator across species. For example, chicken cSH4 insulator has been widely us
[http://transfac.gbf.de/SMARtDB/]) in the targeted sequence and found that the 551bp region is enriched in S/MAR seque
Finally, the experiment design mainly refers to the methods of published articles (Chung et al., 1993; 1997; Petrykowska
study, since the hair follicle-specific promoter and enhancer sequences of goat LHX2 gene have not been reported yet, we
after the SV40 enhancer to detect the potential biological function of this sequence, and the chick cSH4 insulator was sele
Overall, considering phylogenetic relationship between human, mouse and goat, we speculate that this element also funct
fibroblasts, respectively, and the two experimental results were consistent (Wang et al., 2020; Li et al., 2020). This also c
5. * Very good job of supplying all the codes used in the programs. Perhaps this codes or parameters can be combined to
Re: Thank you for your proposal. In order to ensure the repeatability of the project, all the codes and parameters descript
methods.

Reviewer #2: The authors raised an interesting question, hoping to discover the genetic mechanisms associated with cash
kb upstream of LHX2 is involved in regulating cashmere yield and cashmere diameter. The results are very interesting, an
1. 120 goats, are they inbred, and are there enough to get a statistically significant result? What is the statistical basis?
Re: The referee raises an interesting question. In fact, we did not calculate the inbreeding coefficient between individuals
samples of different lineages based on the pedigree records of the conservation farm, and randomly sampled among diffe
In population selective sweep analysis, more than three subgroups/subspecies are usually required, and about 10 samples
selection signals between populations could be detected in the genome.
2. The article begins to describe: 582del and 504del are both correlated with cashmere yield, but only 582del is also signi
interaction between the two genes. What is the mechanism of this interaction? How they are significantly related to cashm
Re: We are grateful to the reviewer for raising this insightful point. Firstly, the two genes have similar spatiotemporal exp
matrix cells during both morphogenesis and anagen (Hébert et al.,1994; Törnqvist et al., 2010). Secondly, LHX2 is an inte
The hair follicle (HF) goes through a cycle of growth containing four main stages: active growth (anagen), regression (cata
is highly expressed during the late anagen phase and promotes the transition from anagen to catagen. The inactivating m
deletion variants. Furthermore, the upregulated expression of the lhx2 gene maintains the function of hair follicle stem cel
enhancer of FGF5 and the insulator of LHX2. Overall, the down-regulated expression of FGF5 and the up-regulated expres
These elaborations are described in the revised manuscript.
3. On the one hand, the text mentions that the deletion sequence 582del in the upstream region of the LHX2 gene may ac
seems to be a contradiction here, please explain.
Re: Thank you for raising this point as it has helped us explain things better in the revised manuscript.
The first sentence is to describe the potential biological function of the 582bp sequence, which may act as an insulator to
LHX2 enhancer and increasing the expression level of LHX2.
We have changed this sentence to: "Luciferase assay shows that the 582bp sequence, which acts as an insulator, restrain
enhancer reduces the expression of FGF5, and thus inhibits the regression of the hair follicle." and marked in the text.
4. To confirm the insulator function of the 582del sequence, the authors synthesized a 551 bp DNA fragment and inserted
identical in length and identity, (2) using mouse and human cell lines respectively, how do we conclude that the 582del se
Re: To further explore the feature of the 582-bp sequence, we downloaded homologous sequences for six additional speci

sequence 2, Replied Sequence 1) was selected from the 582-bp sequence. However, we synthesized a 551 bp DNA fragm
sequence does not have an effect on the experimental results, we reconstructed the vector that deleted the 19 bp and did

Replied Figure 1 Multiple sequence alignment of 582 bp sequence of six species (Goat, Sheep, Cow, Yak, and Siberian mu
Replied Sequence 1
Ins sequence (551bp) was used in the dual-luciferase assay, and the 532-bp highly conserved sequence from 582-bp dele
>Ins sequence(551bp)
GAAAAACAAAACAAAAAGTGTTAAAGGCTTATACTACTGACAAATAGCAGCCCATCTCATGATATCCCTTACCTTCAATCAAAAGTAATCA
ACAAAAACTGTTTCAGTGGAGGCAGGGGCTAAATCACAACACAAGACAGAGGAGAGAATGAGAAGAGAGGAATTAGACAGTAAACAGA
ACGCCAGG

Replied Figure 2 A dual-luciferase assay using 293T cell shows the LHX2 upstream deletion sequence blocks the activity of
SV40 enhancer in the pGL3-control plasmid, and 532 represents that 532-bp sequence was inserted downstream of the SV
columns are significantly different from each other (P < 0.05).
In addition, the purpose of this selection is to reduce the length of the insulator and facilitate its application in the field of
insulator needs further experiments in the future.
Insulators, first identified in Drosophila, are DNA sequence elements that buffer the sequence they delimit from the effects
Insulators have been identified using several different approaches, including in-depth analysis of these sequences, nucleas
In our study, according to the feature of S/MAR sequences in the S/MAR transaction database (rich in AT, like ATTTTT, AA
Supplementary sequence 2), indicating that this region is a candidate insulator.
We then used the benchmark assays to identify its specific biological function. The experiment design mainly refers to the
as an insulator not restricted to erythrocytes or mammalian cells.
This result indicates that the function of insulator depends on its core sequences, including S/MAR sequences, or CTCF bin
fields (Arumugam et al., 2007; Uchida et al., 2013).
In our study, since the hair follicle-specific promoter and enhancer sequences of goat LHX2 gene have not been reported y
and after the SV40 enhancer to detect the potential biological function of this sequence, and the chick cSH4 insulator was
this element also functions as an insulator in goat cells.
On the other hand, we note that two previous experiments about the enhancer function of the 504-bp element located up
conservation of transcriptional regulatory sequences across species. Finally, the reason we did not use goat fibroblasts to
experimental results. Considering phylogenetic relationship between human, mouse and goat, we finally selected mouse 3

Reviewer #3: This manuscript resequenced 42 cashmere goats and 78 ordinary goats, performing Genome-Wide Selective
genetic resources. However, the description in some parts of the manuscript is very rough, and many attachments are mis
Re: We thank the referee for the careful assessment of our work and for considering that the topic is of great interest. We

Major concerns:
1.In the result "Plausible Causative Mutation near LHX2", you selected a lot of cashmere and fiber diameter data for assoc
Re: Yes, confounder effect may cause false positive findings. We used linear model by adding covariant to remove the con
model was expressed as, y=b0+bx+sex+batch+e, where, y was the diameter; x was the deletion coding with 0, 1 and 2 r
assumed to follow normal distribution. When performed the association between the deletion variants and the production,
2.It is found from Fig.2 a that MT and JNG contain a large number of cashmere goat pedigrees. When they are selected an
Re: Our results do show that MT and JNG contain a large number of cashmere goat pedigrees, so we did not use MT and
do selection sweep signals.
3.In Fig 3, are strongly selected loci linked to surrounding loci? Do these sites have an effect on gene expression?
Re: The referee raises a good point. These surrounding selected regions may be within a haplotype block. We found a larg
genes in the DENND1A-LHX2 region and analyzed the spatiotemporal expression patterns. We found that although these g
course we cannot rule out the possibility that there are some loci in other regions that affect the expression of surrounding

4.Fig 3c & d, the horizontal and vertical coordinates of your two graphs are the same, but the trends of the graphs are dif
Re: In Figure 3c, the analysis is based on UC and ordinary goats (YNBB, GZB, JTB and CDB) populations, while in Fig 3d, t
description of the figure legend and also the corresponding content in the paper.

Minor concerns:
5.In the abstract, "Luciferase assay shows that the deletion, which acts as an insulator, restrains the expression of LHX2 b
expression of FGF5, inhibiting the regression".
The conclusion is inconsistent, please clarify the logic of the paper and draw the correct conclusion.

Re: The first sentence is to describe the potential biological function of the 582bp sequence, which may act as an insulator
on LHX2 enhancer and increasing the expression level of LHX2. We have changed this sentence to:” "Luciferase assay sho
cashmere fiber growth at the anagen stage, while deletion of the FGF5 enhancer reduces the expression of FGF5, and thus
6."Luciferase assay shows that the deletion, which acts as an insulator, restrains the expression of LHX2 by interfering its
These two sentences are easy to confuse us, and it will make people understand that two insulators are found on LHX2, o
Re: Thanks for pointing it out, we've revised it: “Luciferase assay shows that the 582bp sequence, which acts as an insula
7.There are inconsistencies in the sample names in the paper. For example,you use IRWG in the front of the sentence and
(1) In the STRUCTURE analysis, When K = 4, we observed five separate clusters: IRWG and ANG in west Asia, YNBB, GZB
subgroups: the Yunnan-Kweichow Plateau group including YNBB and GZB goats, and the Chengdu Plain group including C
(2)More interestingly, we found that the 582del has a high frequency in the IRWG population (80.9 %), while the 504del w
(3)10 JTB from Jintang County of Sichuan Province; 12 CDB from Chengdu City of Sichuan Province".."
To further evaluate whether these two deletion variants were related to cashmere
(4)traits, we selected 235 CDMC goatswith cashmere yield (Supplementary Fig. 22, Supplementary Table s8) and 581 CD
(5)Fig 2b, "KOG".
Re: All have been changed in the new version of the MS, thank you very much.
8."We inspected all variants within exons to identify the potential causal mutation around the DENND1A-LHX2 locus; howe
Please put the information of the relevant sites in the attachment, only one sentence will make the paper unconvincing.
Re: Thank you for your advice. We previously annotated mutation locus in the DENND1A-LHX2 gene region using snpEFF
9."Analysis of the 582del deletion region using the BLAST program revealed that it is not a highly conserved element but
"582del deletion" is a repetition.
Re: Thank you for your proposal. We have revised it.

REFERENCES

Alonso L, Fuchs E. 2006. The hair cycle. J Cell Sci. 119:391–393.
Arumugam PI, Scholes J, Perelman N, Xia P, Yee JK, Malik P. 2007. Improved human beta-globin expression from self-ina
Barkess G, West AG. 2012. Chromatin insulator elements: establishing barriers to set heterochromatin boundaries. Epiger
Burgess-Beusse B, Farrell C, Gaszner M, Litt M, Mutskov V, Recillas-Targa F, Simpson M, West A, Felsenfeld G. 2002. The
Chung JH, Bell AC, Felsenfeld G. 1997. Characterization of the chicken beta-globin insulator. Proc Natl Acad Sci U S A. 94:
Chung JH, Whiteley M, Felsenfeld G. 1993. A 5' element of the chicken beta-globin domain serves as an insulator in huma
Engel N, Bartolomei MS. 2003. Mechanisms of insulator function in gene regulation and genomic imprinting. Int Rev Cytol
Folgueras AR, Guo X, Pasolli HA, Stokes N, Polak L, Zheng D, Fuchs E. 2013. Architectural niche organization by LHX2 is li
Hébert JM, Rosenquist T, Götz J, Martin GR. 1994. FGF5 as a regulator of the hair growth cycle: evidence from targeted a
Higgins CA, Petukhova L, Harel S, Ho YY, Drill E, Shapiro L, Wajid M, Christiano AM. 2014. FGF5 is a crucial regulator of h
Lee, A. P.S. Brenner and B. Venkatesh. 2011. Mouse transgenesis identifies conserved functional enhancers and cis-regula
Li, Y., S. Song, X. Liu, Y. Zhang, D. Wang, X. He, Q. Zhao, Y. Pu, W. Guan, Y. Ma and L. Jiang. 2020. Deletion of an enha
Liebich I, Bode J, Frisch M, Wingender E. 2002. S/MARt DB: a database on scaffold/matrix attached regions. Nucleic Acids
Petrykowska HM, Vockley CM, Elnitski L. 2008. Detection and characterization of silencers and enhancer-blockers in the g
Qiu Q, Wang L, Wang K, Yang Y, Ma T, Wang Z, Zhang X, Ni Z, Hou F, Long R, Abbott R, Lenstra J, Liu J. 2015. Yak whole
Rubin CJ, Megens HJ, Martinez Barrio A, Maqbool K, Sayyab S, Schwochow D, Wang C, Carlborg Ö, Jern P, Jørgensen CB,
Seth A, Culverwell J, Walkowicz M, Toro S, Rick JM, Neuhauss SC, Varga ZM, Karlstrom RO. 2006. belladonna/(Ihx2) is re
Törnqvist G, Sandberg A, Hägglund AC, Carlsson L. 2010. Cyclic expression of lhx2 regulates hair formation. PLoS Genet.
Uchida N, Hanawa H, Yamamoto M, Shimada T. 2013. The chicken hypersensitivity site 4 core insulator blocks promoter i
Villemure J.F. Savard N. Belmaaza A. 2001. Promoter suppression in cultured mammalian cells can be blocked by the chic
Wang, M. Yang, S. Hu, Y. Li, Z. Yang, M. Gong, Y. Hou, T. Lan, K. Wu, Y. Chen, Y. Jiang, and X. Wang. 2020. Ancient Gen
Watson BA, Feenstra JM, Van Arsdale JM, Rai-Bhatti KS, Kim DJH, Coggins AS, Mattison GL, Yoo S, Steinman ED, Pira CU,
Yang J, Li WR, Lv FH, He SG, Tian SL, Peng WF, Sun YW, Zhao YX, Tu XL, Zhang M, Xie XL, Wang YT, Li JQ, Liu YG, Shen

Supplementary data

Replied Table 1 Raw data of firefly luciferase activity and Renilla luciferase activity

Group	Fluc	Rluc	Ratio
control-1	380743008	34958396	10.89132
control-2	254579744	28838640	8.827731
control-3	283508960	32223302	8.798259
csh4-1	42606228	22212086	1.918155

csh4-2 38121500 19651264 1.939901
csh4-3 35589416 20236980 1.758633
csh4-4 40099180 21899328 1.831069
ins-1 78732288 16043951 4.907288
ins-2 41620224 12153245 3.424618
ins-3 62319672 18844526 3.307044
ins-4 61497032 16724342 3.677097
532-1 46733104 11156289 4.188947
532-2 56791240 14014137 4.052425
532-3 62227168 12360058 5.034537
532-4 77523304 15243126 5.085788

Replied Table 2 Effect of insulator sequence on activity of enhancer inhibition
Group diff lwr upr p adj
ctl-ins 6.036 4.271897 7.800103 0.00002**
ctl-csh4 7.630333 5.86623 9.394437 0.0000034**
ctl-532 4.782 3.017897 6.546103 0.0001118**
ins-532 -1.254 -3.0181 0.510103 0.1828874
csh4-ins -1.59433 -3.35844 0.16977 0.0772422
csh4-532 -2.84833 -4.61244 -1.08423 0.0037634**
Note: Asterisks represent statistical significance

Close
