# Supplementary material for: Selection signatures in goats reveal a novel deletion mutant underlying cashmere yield and diameter
Source: Gigascience. 2022 Nov 3;11:giac107. doi: 10.1093/gigascience/giac107 (PMC9633279; doi:10.1093/gigascience/giac107)
Supplement: giac107_Response_to_Reviewer_Comments_Revision_2 [file giac107_response_to_reviewer_comments_revision_2.pdf]

Author's Response To Reviewer Comments

Close

Reviewer reports:
Reviewer #3: The author has answered most of the questions well, but the answer to the fifth question is still very unclear:

"FGF5 enhancer deletion may have occurred during artificial selection or secondary domestication in the Kashmir region of Afghanistan and gradually migrated north of the Mongolian Plateau." What is your robust evidence to confirm this hypothesis? When the British entered Kashmir from the India, they found that cashmere was raised. But can we prove this is the place of origin?
RE : Thanks very much, it is a good question. In fact, the origin of the domestic cashmere goat remains uncertain and controversial. Some authors suggested that the Cashmere goat breed probably originated from the Capra falconeri, a large Capra species native to Central Asia, the Karakoram, and the Himalayas (Ganai et al., 2011). In addition, some studies also have suggested that a second domestication in Pakistan gave rise to the Cashmere breeds (Meadow 1996; Luikart et a., 2001). On other hand, Chinese cashmere goats, however, do have the distinction of growing more underwool than other double coated goats and also of having a greater proportion of white animals. Some authors suggested that the concentration of cashmere goats moved from the western end of the Himalayas to north west China during the last 150 years (Ryder 1993). Based on this evidence, our study hint that FGF5 enhancer deletion may have occurred during artificial selection or secondary domestication in the Kashmir region of Afghanistan and gradually migrated north of the Mongolian Plateau. However, this evidence is not very robust and need more research data to support.

Comments : There is no good connection between the author's cited references and the speculation. Did the authors detect this deletion of FGF5 in Capra falconeri and ancient samples? It is suggested that the author remove Fig.7 and emphasize in the sentence corresponding to the description that this result is only speculation and there are other possibilities.

Re: Yes, we did. The results showed that the deletion allele of FGF5 was not found in the Iranian Wild Goat (0/21), Capra falconeri and Capra sibirica (0/6) as well as ancient goats(0/21) (Supplementary Table 6).
Thanks very much for your suggestion. We removed Fig.7 and modified the corresponding descriptions in the text.

Close
