# Supplementary material for: Selection signatures in goats reveal a novel deletion mutant underlying cashmere yield and diameter
Source: Gigascience. 2022 Nov 3;11:giac107. doi: 10.1093/gigascience/giac107 (PMC9633279; doi:10.1093/gigascience/giac107)
Supplement: giac107_Response_to_Reviewer_Comments_Revision_3 [file giac107_response_to_reviewer_comments_revision_3.pdf]

Author's Response To Reviewer Comments

Close

1 GigaScience allows 3 joint first authors at most and 2 corresponding authors at most, please make sure the numbers meet our requirement.
Reply: We have revised the manuscript as requested.Yes, * represents Joint first author, and # represents Corresponding author.
2 Note that figures must be submitted as separate image files, not as part of the submitted manuscript file.
Reply: We have revised the manuscript as requested.At the time of this manuscript submission, the pictures were uploaded as required.
3 URL link shouldn't be included in the main text. please cite it as a reference.
Reply: Urls were removed from the manuscript and references were added.
4 Please add details here.
Reply: Reference were adde
5 List of abreferences were addedbreviations
Reply: A list of abbreviations was added to the manuscript and sorted.
6 Please add details here if available
Reply: Fund information has been refined in the manuscript.
7 All references, including URLs, must be numbered consecutively, in square brackets, in the order in which they are cited in the text, followed by any in tables or legends.
Reply: We have revised all the references in the manuscript according to the requirements of the GigaScience format.

Close
