# Supplementary material for: Selection signatures in goats reveal a novel deletion mutant underlying cashmere yield and diameter
Source: Gigascience. 2022 Nov 3;11:giac107. doi: 10.1093/gigascience/giac107 (PMC9633279; doi:10.1093/gigascience/giac107)
Supplement: giac107_Reviewer_2_Report_Original_Submission — Yixue Li -- 3/10/2022 Reviewed [file giac107_reviewer_2_report_original_submission.pdf]

Reviewer Report

Title: Selection signatures in goats reveal a novel deletion mutant underlying cashmere yield and diameter

Version: Original Submission **Date:** 3/10/2022

Reviewer name: Yixue Li

Reviewer Comments to Author:

The authors raised an interesting question, hoping to discover the genetic mechanisms associated with cashmere traits for breed improvement. The authors sequenced 120 native Chinese goats, including 2 cashmere goat breeds and 6 common goat breeds. Through analysis, the authors found and believe they confirmed that a 582 bp deletion at 367 kb upstream of LHX2 is involved in regulating cashmere yield and cashmere diameter. The results are very interesting, and if they convincingly answer the questions below, acceptance for publication is recommended.

1. 120 goats, are they inbred, and are there enough to get a statistically significant result? What is the statistical basis?
2. The article begins to describe: 582del and 504del are both correlated with cashmere yield, but only 582del is also significantly correlated with fiber diameter, while 504del has no significant correlation with fiber diameter. Then he added: The interaction effect between 582del and 504del was significantly correlated with cashmere fiber diameter, indicating an interaction between the two genes. What is the mechanism of this interaction? How they are significantly related to cashmere fiber diameter, further elaboration is needed.
3. On the one hand, the text mentions that the deletion sequence 582del in the upstream region of the LHX2 gene may act as an insulator, preventing the function of the LHX2 enhancer. Later, it is mentioned that the deletion of the LHX2 insulator 582del increases the expression of LHX2 and promotes the growth of cashmere fibers during the growth period. There seems to be a contradiction here, please explain.
4. To confirm the insulator function of the 582del sequence, the authors synthesized a 551 bp DNA fragment and inserted it into the pGL3 plasmid downstream and upstream of the SV40 promoter, and then co-transfected human 293T cells and mouse 3T3 cells, and thus confirmed the insulator function of the 582del sequence. Here: (1) the two sequences are not identical in length and identity, (2) using mouse and human cell lines respectively, how do we conclude that the 582del sequence will have the same function in goat? What is the experimental logic and biological logic here? Hopefully a convincing explanation can be given.

Methods

Are the methods appropriate to the aims of the study, are they well described, and are necessary controls included? Choose an item.

Conclusions

Are the conclusions adequately supported by the data shown? Choose an item.

Reporting Standards

Does the manuscript adhere to the journal's guidelines on minimum standards of reporting? Choose an item.

Choose an item.

Statistics

Are you able to assess all statistics in the manuscript, including the appropriateness of statistical tests used? Choose an item.

Quality of Written English

Please indicate the quality of language in the manuscript: Choose an item.

Declaration of Competing Interests

Please complete a declaration of competing interests, considering the following questions:

- Have you in the past five years received reimbursements, fees, funding, or salary from an organisation that may in any way gain or lose financially from the publication of this manuscript, either now or in the future?
- Do you hold any stocks or shares in an organisation that may in any way gain or lose financially from the publication of this manuscript, either now or in the future?
- Do you hold or are you currently applying for any patents relating to the content of the manuscript?
- Have you received reimbursements, fees, funding, or salary from an organization that holds or has applied for patents relating to the content of the manuscript?
- Do you have any other financial competing interests?
- Do you have any non-financial competing interests in relation to this paper?

If you can answer no to all of the above, write 'I declare that I have no competing interests' below. If your reply is yes to any, please give details below.

I declare that I have no competing interestsã€,

I agree to the open peer review policy of the journal. I understand that my name will be included on my report to the authors and, if the manuscript is accepted for publication, my named report including any attachments I upload will be posted on the website along with the authors' responses. I agree for my

report to be made available under an Open Access Creative Commons CC-BY license (http://creativecommons.org/licenses/by/4.0/). I understand that any comments which I do not wish to be included in my named report can be included as confidential comments to the editors, which will not be published.

Choose an item.

To further support our reviewers, we have joined with Publons, where you can gain additional credit to further highlight your hard work (see: https://publons.com/journal/530/gigascience). On publication of this paper, your review will be automatically added to Publons, you can then choose whether or not to claim your Publons credit. I understand this statement.

Yes Choose an item.
