# Supplementary material for: Selection signatures in goats reveal a novel deletion mutant underlying cashmere yield and diameter
Source: Gigascience. 2022 Nov 3;11:giac107. doi: 10.1093/gigascience/giac107 (PMC9633279; doi:10.1093/gigascience/giac107)
Supplement: giac107_Reviewer_3_Report_Original_Submission — Yu Jiang, Ph.D -- 3/15/2022 Reviewed [file giac107_reviewer_3_report_original_submission.pdf]

Reviewer Report

Title: Selection signatures in goats reveal a novel deletion mutant underlying cashmere yield and diameter

Version: Original Submission **Date:** 3/15/2022

Reviewer name: Yu Jiang, Ph.D

Reviewer Comments to Author:

This manuscript resequenced 42 cashmere goats and 78 ordinary goats, performing Genome-Wide Selective Sweeps and then a 582 bp deletion in the thirteenth intron region of DENND1A upstream of LHX2 was found to increase cashmere yield. This discovery provides resources for the development of the wool industry and the enrichment of animal genetic resources. However, the description in some parts of the manuscript is very rough, and many attachments are missing.
Major concerns:
1.In the result "Plausible Causative Mutation near LHX2", you selected a lot of cashmere and fiber diameter data for association analysis and get Fig 5e, but your results may have high false positives. The author should demonstrate that the deletion is significantly associated with phenotype after excluding factors such as gender, age and so on.
2.It is found from Fig.2 a that MT and JNG contain a large number of cashmere goat pedigrees. When they are selected and analyzed with cashmere goat samples, the results may be affected by this mixing. The author should consider the particularity of these samples when using them. Perform subsequent analysis.
3.In Fig 3, are strongly selected loci linked to surrounding loci? Do these sites have an effect on gene expression?
4.Fig 3c & d, the horizontal and vertical coordinates of your two graphs are the same, but the trends of the graphs are different. Please mark clearly what the two graphs describe in the legend and the paper.
Minor concerns:
5.In the abstract, "Luciferase assay shows that the deletion, which acts as an insulator, restrains the expression of LHX2 by interfering its upstream enhancers", but in the result, "Therefore, the deletion of the LHX2 insulator increases the expression of LHX2 and promotes cashmere fiber growth at the anagen stage, while deletion of the FGF5 enhancer reduces the expression of FGF5, inhibiting the regression". The conclusion is inconsistent, please clarify the logic of the paper and draw the correct conclusion.
6."Luciferase assay shows that the deletion, which acts as an insulator, restrains the expression of LHX2 by interfering its upstream enhancers. Our study discovers a novel insulator of the LHX2 involved in regulating cashmere production and diameter."
These two sentences are easy to confuse us, and it will make people understand that two insulators are found on LHX2, one to suppress expression and one to regulate cashmere production and diameter, and it is recommended to modify.
7.There are inconsistencies in the sample names in the paper. For example,you use IRWG in the front of

the sentence and IRW in the back,please unify the name. At the same time, the paper contains many spelling and symbol errors, such as "mddle", "goatswith", symbol repetition and so on.
(1)In the STRUCTURE analysis, When K = 4, we observed five separate clusters:
IRWG and ANG in west Asia, YNBB, GZB, JTB and CDB in southwest China;
cashmere goat in north China; MT and JNG in mddle east China; and Korean goats in
south Korea. At K = 6, goats in the southwest China further split into two geographic
subgroups: the Yunnan-Kweichow Plateau group including YNBB and GZB goats, and the Chengdu Plain group including CDM and JTB goats. Two west Asian goats (IRW and ANG) were also separated .
(2)More interestingly, we found that the 582del has a high frequency in the IRWG population (80.9 %), while the 504del was absent, which is also consistent with previous research.
(3)10 JTB from Jintang County of Sichuan Province; 12 CDB from Chengdu City of Sichuan Province".."
To further evaluate whether these two deletion variants were related to cashmere
(4)traits, we selected 235 CDMC goatswith cashmere yield (Supplementary Fig. 22, Supplementary Table s8) and 581 CDMC goats with fiber diameter records (Supplementary Fig. 23, Supplementary Table s9) for association analysis.
(5)Fig 2b, "KOG".
8."We inspected all variants within exons to identify the potential causal mutation around the DENND1A-LHX2 locus; however, no coding variants were found. "
Please put the information of the relevant sites in the attachment, only one sentence will make the paper unconvincing.
9."Analysis of the 582del deletion region using the BLAST program revealed that it is not a highly conserved element but was found in the genomes of primate and ungulate species. "
"582del deletion" is a repetition.

Methods

Are the methods appropriate to the aims of the study, are they well described, and are necessary controls included? Choose an item.

Conclusions

Are the conclusions adequately supported by the data shown? Choose an item.

Reporting Standards

Does the manuscript adhere to the journal's guidelines on minimum standards of reporting? Choose an item.

Choose an item.

Statistics

Are you able to assess all statistics in the manuscript, including the appropriateness of statistical tests used? Choose an item.

Quality of Written English

Please indicate the quality of language in the manuscript: Choose an item.

Declaration of Competing Interests

Please complete a declaration of competing interests, considering the following questions:

- Have you in the past five years received reimbursements, fees, funding, or salary from an organisation that may in any way gain or lose financially from the publication of this manuscript, either now or in the future?
- Do you hold any stocks or shares in an organisation that may in any way gain or lose financially from the publication of this manuscript, either now or in the future?
- Do you hold or are you currently applying for any patents relating to the content of the manuscript?
- Have you received reimbursements, fees, funding, or salary from an organization that holds or has applied for patents relating to the content of the manuscript?
- Do you have any other financial competing interests?
- Do you have any non-financial competing interests in relation to this paper?

If you can answer no to all of the above, write 'I declare that I have no competing interests' below. If your reply is yes to any, please give details below.

I declare that I have no competing interests.

I agree to the open peer review policy of the journal. I understand that my name will be included on my report to the authors and, if the manuscript is accepted for publication, my named report including any attachments I upload will be posted on the website along with the authors' responses. I agree for my report to be made available under an Open Access Creative Commons CC-BY license (http://creativecommons.org/licenses/by/4.0/). I understand that any comments which I do not wish to be included in my named report can be included as confidential comments to the editors, which will not be published.

Choose an item.

To further support our reviewers, we have joined with Publons, where you can gain additional credit to further highlight your hard work (see: https://publons.com/journal/530/gigascience). On publication of this paper, your review will be automatically added to Publons, you can then choose whether or not to claim your Publons credit. I understand this statement.

Yes Choose an item.
