# Supplementary material for: Selection signatures in goats reveal a novel deletion mutant underlying cashmere yield and diameter
Source: Gigascience. 2022 Nov 3;11:giac107. doi: 10.1093/gigascience/giac107 (PMC9633279; doi:10.1093/gigascience/giac107)
Supplement: giac107_Reviewer_3_Report_Revision_1 — Yu Jiang, Ph.D -- 6/20/2022 Reviewed [file giac107_reviewer_3_report_revision_1.pdf]

Reviewer Report

Title: Selection signatures in goats reveal a novel deletion mutant underlying cashmere yield and diameter

Version: Revision 1 **Date:** 6/20/2022

Reviewer name: Yu Jiang, Ph.D

Reviewer Comments to Author:

The manuscript written by Han et al. is an interesting study aiming at investigating the genetic mutations underlying cashmere yield and its diameter. In this manuscript, the authors have sequenced 120 Chinese native goats including two cashmeres (n=42) and six ordinary breeds (n=78), and also downloaded published whole-genome sequencing data from 116 individuals (five different goat breeds). The authors performed genome-wide selective sweep analyses based on the genomic variation data, and identified a 582bp deletion of LHX2 gene, which may be important for cashmere production and for its diameter.
In general, the logic of the paper is clear and the authors have done corresponding experimental verifications in both human and mice species. However, the manuscript needs to be further improved.
1: In Fig 1C, the author classified two distinct cashmere goats (UC and IMC), however in the Supplementary Table 1 there is only one group (UC). Please check this.
2: Please check the below sentences and see how Supplementary Table S7 and Supplementary Table S8 would match to this paragraph!
"To further evaluate whether these two deletion variants were related to cashmere traits, we selected 235 CDMC goats with cashmere yield (Supplementary Fig. 16, Supplementary Table s7) and 581 CDMC goats with fiber diameter records (Supplementary Fig. 17, Supplementary Table s8) for association analysis".
3: In the "Phenotypic analysis" section (P 25). Please provide more details about the phenotypic samples that you have applied for genomic correlation analysis. Did you take phenotypic samples from the same individuals that you have sequenced in this study? If no, what is the average age of the animals? Whether the age factor is corrected as a co-variable in this analysis? The wool production and its diameter is greatly depending of the age of the animals, which finally can affect your results.
4: Fig. 5e. Please add the P value, corresponding to the below sentence.
"The 582del was also significantly associated with fiber diameter (P value = 2.74e-04), but the 504del was not (Fig. 5e)."
5: "FGF5 enhancer deletion may have occurred during artificial selection or secondary domestication in the Kashmir region of Afghanistan and gradually migrated north of the Mongolian Plateau." What is your robust evidence to confirm this hypothesis? When the British entered Kashmir from the India, they found that cashmere was raised. But can we prove this is the place of origin?

Methods

Are the methods appropriate to the aims of the study, are they well described, and are necessary controls included? Choose an item.

Conclusions

Are the conclusions adequately supported by the data shown? Choose an item.

Reporting Standards

Does the manuscript adhere to the journal's guidelines on minimum standards of reporting? Choose an item.

Choose an item.

Statistics

Are you able to assess all statistics in the manuscript, including the appropriateness of statistical tests used? Choose an item.

Quality of Written English

Please indicate the quality of language in the manuscript: Choose an item.

Declaration of Competing Interests

Please complete a declaration of competing interests, considering the following questions:

- Have you in the past five years received reimbursements, fees, funding, or salary from an organisation that may in any way gain or lose financially from the publication of this manuscript, either now or in the future?
- Do you hold any stocks or shares in an organisation that may in any way gain or lose financially from the publication of this manuscript, either now or in the future?
- Do you hold or are you currently applying for any patents relating to the content of the manuscript?
- Have you received reimbursements, fees, funding, or salary from an organization that holds or has applied for patents relating to the content of the manuscript?
- Do you have any other financial competing interests?
- Do you have any non-financial competing interests in relation to this paper?

If you can answer no to all of the above, write 'I declare that I have no competing interests' below. If your reply is yes to any, please give details below.

I declare that I have no competing interests

I agree to the open peer review policy of the journal. I understand that my name will be included on my report to the authors and, if the manuscript is accepted for publication, my named report including any attachments I upload will be posted on the website along with the authors' responses. I agree for my

report to be made available under an Open Access Creative Commons CC-BY license (http://creativecommons.org/licenses/by/4.0/). I understand that any comments which I do not wish to be included in my named report can be included as confidential comments to the editors, which will not be published.

Choose an item.

To further support our reviewers, we have joined with Publons, where you can gain additional credit to further highlight your hard work (see: https://publons.com/journal/530/gigascience). On publication of this paper, your review will be automatically added to Publons, you can then choose whether or not to claim your Publons credit. I understand this statement.

Yes Choose an item.
