# Supplementary material for: Selection signatures in goats reveal a novel deletion mutant underlying cashmere yield and diameter
Source: Gigascience. 2022 Nov 3;11:giac107. doi: 10.1093/gigascience/giac107 (PMC9633279; doi:10.1093/gigascience/giac107)
Supplement: giac107_Reviewer_3_Report_Revision_2 — Yu Jiang, Ph.D -- 7/4/2022 Reviewed [file giac107_reviewer_3_report_revision_2.pdf]

Reviewer Report

Title: Selection signatures in goats reveal a novel deletion mutant underlying cashmere yield and diameter

Version: Revision 2 **Date:** 7/4/2022

Reviewer name: Yu Jiang, Ph.D

Reviewer Comments to Author:

The author has answered most of the questions well, but the answer to the fifth question is still very unclear:
"FGF5 enhancer deletion may have occurred during artificial selection or secondary domestication in the Kashmir region of Afghanistan and gradually migrated north of the Mongolian Plateau." What is your robust evidence to confirm this hypothesis? When the British entered Kashmir from the India, they found that cashmere was raised. But can we prove this is the place of origin?
REï¼šThanks very much, it is a good question. In fact, the origin of the domestic cashmere goat remains uncertain and controversial. Some authors suggested that the Cashmere goat breed probably originated from the Capra falconeri, a large Capra species native to Central Asia, the Karakoram, and the Himalayas (Ganai et al., 2011). In addition, some studies also have suggested that a second domestication in Pakistan gave rise to the Cashmere breeds (Meadow 1996; Luikart et a., 2001). On other hand, Chinese cashmere goats, however, do have the distinction of growing more underwool than other double coated goats and also of having a greater proportion of white animals. Some authors suggested that the concentration of cashmere goats moved from the western end of the Himalayas to north west China during the last 150 years (Ryder 1993). Based on this evidence, our study hint that FGF5 enhancer deletion may have occurred during artificial selection or secondary domestication in the Kashmir region of Afghanistan and gradually migrated north of the Mongolian Plateau. However, this evidence is not very robust and need more research data to support.
There is no good connection between the author's cited references and the speculation. Did the authors detect this deletion of FGF5 in Capra falconeri and ancient samples? It is suggested that the author remove Fig.7 and emphasize in the sentence corresponding to the description that this result is only speculation and there are other possibilities.

Methods

Are the methods appropriate to the aims of the study, are they well described, and are necessary controls included? Choose an item.

Conclusions

Are the conclusions adequately supported by the data shown? Choose an item.

Reporting Standards

Does the manuscript adhere to the journal's guidelines on minimum standards of reporting? Choose an item.

Choose an item.

Statistics

Are you able to assess all statistics in the manuscript, including the appropriateness of statistical tests used? Choose an item.

Quality of Written English

Please indicate the quality of language in the manuscript: Choose an item.

Declaration of Competing Interests

Please complete a declaration of competing interests, considering the following questions:

- Have you in the past five years received reimbursements, fees, funding, or salary from an organisation that may in any way gain or lose financially from the publication of this manuscript, either now or in the future?
- Do you hold any stocks or shares in an organisation that may in any way gain or lose financially from the publication of this manuscript, either now or in the future?
- Do you hold or are you currently applying for any patents relating to the content of the manuscript?
- Have you received reimbursements, fees, funding, or salary from an organization that holds or has applied for patents relating to the content of the manuscript?
- Do you have any other financial competing interests?
- Do you have any non-financial competing interests in relation to this paper?

If you can answer no to all of the above, write 'I declare that I have no competing interests' below. If your reply is yes to any, please give details below.

I declare that I have no competing interests

I agree to the open peer review policy of the journal. I understand that my name will be included on my report to the authors and, if the manuscript is accepted for publication, my named report including any attachments I upload will be posted on the website along with the authors' responses. I agree for my report to be made available under an Open Access Creative Commons CC-BY license (http://creativecommons.org/licenses/by/4.0/). I understand that any comments which I do not wish to be included in my named report can be included as confidential comments to the editors, which will not be published.

Choose an item.

To further support our reviewers, we have joined with Publons, where you can gain additional credit to further highlight your hard work (see: https://publons.com/journal/530/gigascience). On publication of this paper, your review will be automatically added to Publons, you can then choose whether or not to claim your Publons credit. I understand this statement.

Yes Choose an item.
